# Supplementary material for: Histone demethylase KDM2A recruits HCFC1 and E2F1 to orchestrate male germ cell meiotic entry and progression
Source: EMBO J. 2024 Aug 19;43(19):4197–227. doi: 10.1038/s44318-024-00203-4 (PMC11448500; doi:10.1038/s44318-024-00203-4)
Supplement: Supplementary file 1 — Appendix [file 44318_2024_203_MOESM1_ESM.pdf]

Appendix

Histone demethylase KDM2A recruits HCFC1 and E2F1 to orchestrate male germ cell meiotic entry and progression

By Shenglei Feng et al.

Contents:

Appendix Figure S1. KDM2A regulates gene expression of Stra8 and Meiosin. …………………..2

Appendix Figure S2. KDM2A is essential for normal deposition of H3K36me2/3. ……………….. 4

Appendix Figure S3. KDM2A cooperates with HCFC1 and E2F1 in germ cells. …………………. 6

Appendix Figure S4. KDM2A and E2F1 regulate the expression of downregulated genes. ……. 8

Appendix Table S1. Primer sequences are used in this study. ……………………………………..10

Appendix Table S2. Antibodies used in this study. ……………… …………………………………12

Appendix Figure S1

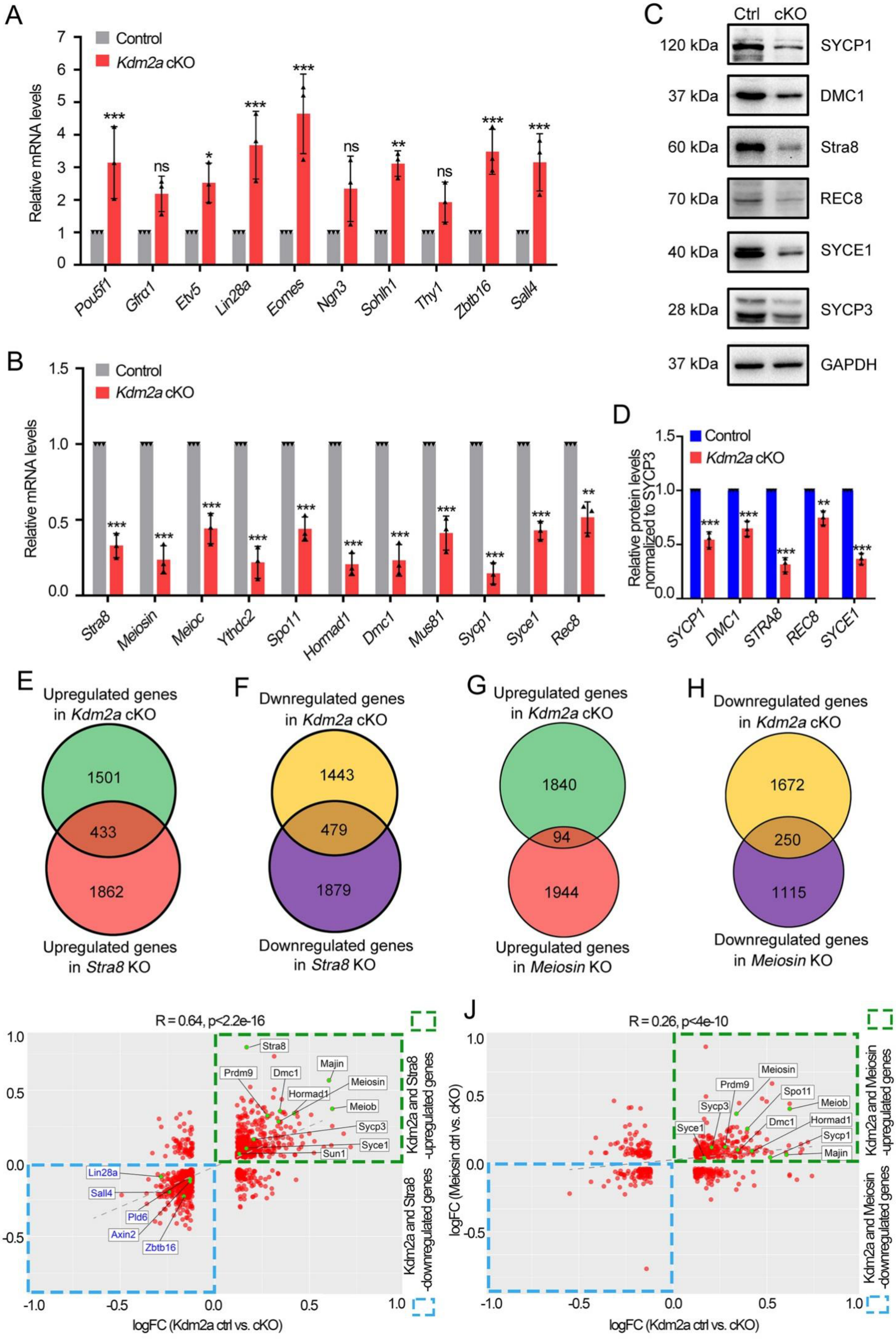

Appendix Figure S1. KDM2A regulates gene expression of *Stra8* and *Meiosin.*

(**A-B**) RT-qPCR analyses the upregulated genes are related to SSC development (A) and the downregulated genes are related to meiosis (B) in *Kdm2a* cKO c-KIT^+ differentiating spermatogonia cells from RNA-seq data. Data were presented as the mean ± SD. n = 3. *P < 0.05; **P < 0.01; ***P < 0.001.

(**C-D**) WB analysis of protein levels of the selected downregulated genes that are associated with meiosis-related genes in *Kdm2a* cKO c-KIT^+ cells identified from RNA-seq data. GAPDH served as a loading control. The quantifications were normalized to SYCP3 (D). Data were presented as the mean ± SD. n = 3. **P < 0.01; ***P < 0.001.

(**E-F**) Venn diagrams showing the overlap among the upregulated transcripts (E) and downregulated transcripts (F) from our RNA-seq data together with upregulated transcripts (E) and downregulated transcripts (F) from the RNA-seq database after deletion S*tra8*.

(**G-H**) Venn diagrams showing the overlap among the upregulated genes (G) and downregulated genes (H) from our RNA-seq data together with upregulated genes (G) and downregulated genes (H) from the RNA-seq database after deletion *Meiosin*.

(**I-J**) Distribution of log (FC, fold change) values for DGEs in *Kdm2a* cKO c-KIT^+ cells. The genes involved in indicated signaling pathways and co-regulated by *Kdm2a* with *Stra8* (I) and *Meiosin* (J) are marked in red.

Appendix Figure S2

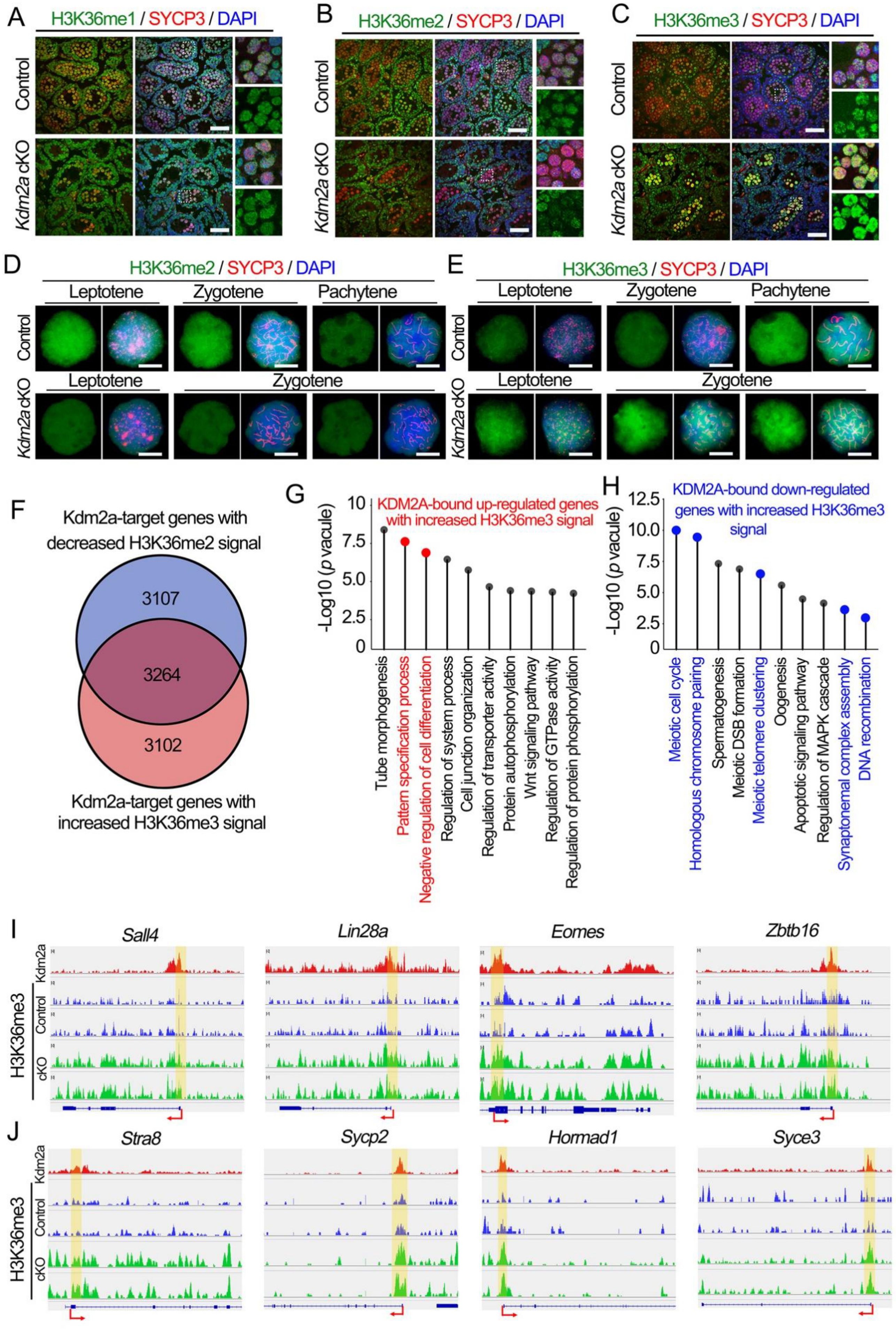

Appendix Figure S2. KDM2A is essential for normal deposition of H3K36me2/3.

(**A-C**) Co-Immunofluorescent staining of SYCP3 with H3K36me1 (A), H3K36me2 (B), and H3K36me3 (C) on testis sections from control and *Kdm2a* cKO mice at P14, respectively. Nuclei were stained with DAPI. Enlarged images are shown on the right panel. Scale bars = 50 μm.

(**D-E**) Co-Immunofluorescent staining of SYCP3 with H3K36me2 (D) and H3K36me2 (E) on spermatocyte spreads from control and *Kdm2a* cKO testes at P18. Nuclei were stained with DAPI. Scale bars = 5 μm.

(**F**) Venn diagrams showing overlap of *Kdm2a*-bound genes with decreased H3K36me2 signal genes (6371), and *Kdm2a*-bound genes with increased H3K36me3 signal genes (6366).

(**G-H**) GO analyses of the upregulated genes (G) and downregulated genes(H) with increased H3K36me3 signal in *Kdm2a*-bound genes.

(**I-J**) Genome browser tracks depicting reads accumulation of H3K36me3 on representative genes in control and *Kdm2a* cKO c-KIT-positive cells using IGV software.

Appendix Figure S3

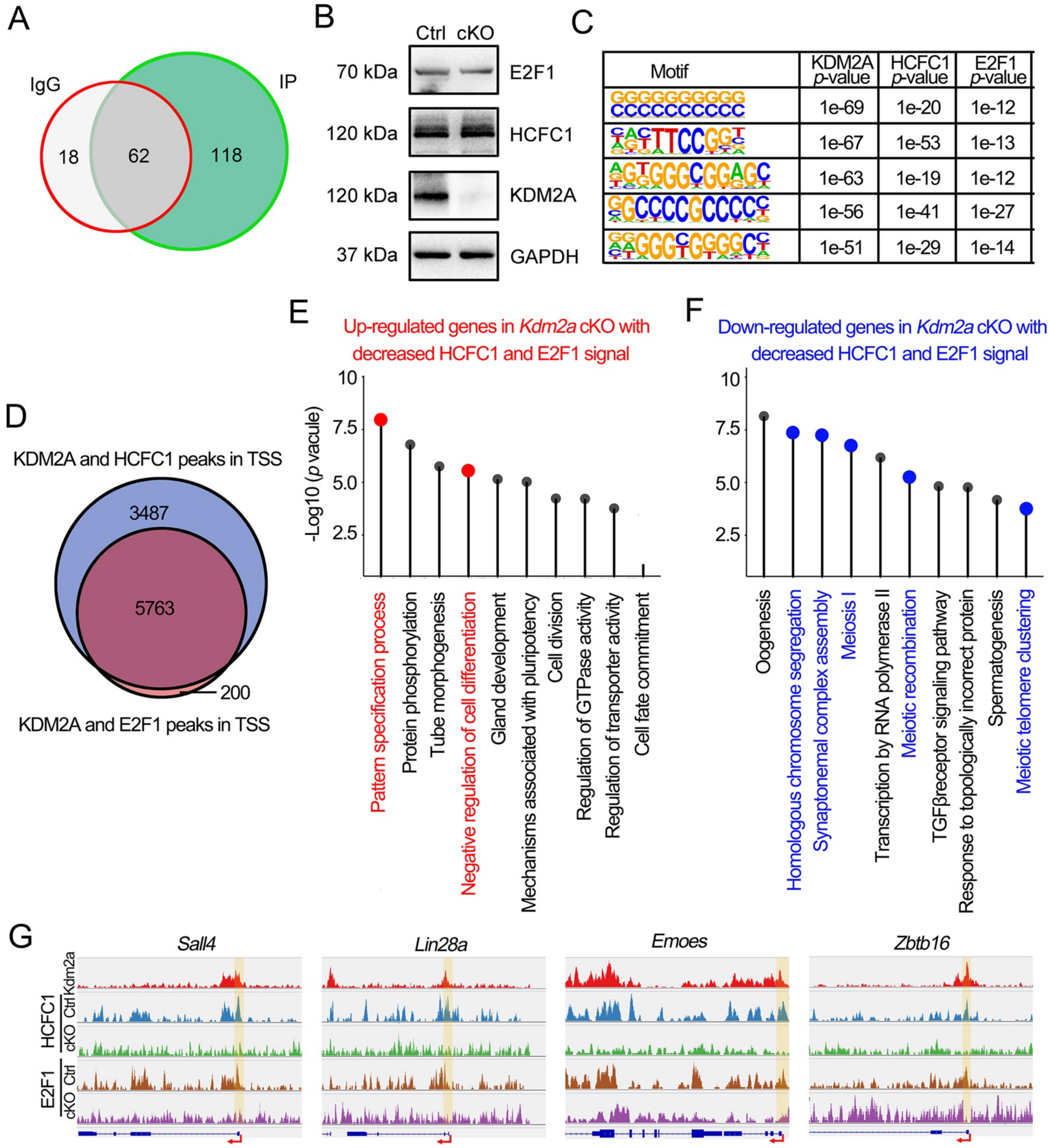

Appendix Figure S3. KDM2A cooperates with HCFC1 and E2F1 in germ cells.

(**A**) Venn diagrams representing the overlap among KDM2A-interacting partners from IP and IgG by IP-MS data.

(**B**) The interacting protein (E2F1 and HCFC1) expression levels from control and *Kdm2a* cKO c-KIT^{+} cells were determined by WB. GAPDH was used as a loading control.

(**C**) Top five ranked sequence motifs enriched in ChIP-seq data from KDM2A (left), HCFC1 (middle), and E2F1 (right) with E values are shown.

(**D**) Venn diagram representing the overlap between HCFC1-bound TSS and E2F- bound TSS from Kdm2a cKO c-KIT-positive cells.

(**E**) GO analyses of the upregulated genes in *Kdm2a* cKO c-KIT-positive cells with decreased HCFC1 and E2F1 signals.

(**F**) GO analyses of the downregulated genes of *Kdm2a* cKO c-KIT-positive cells with decreased HCFC1 and E2F1 signals.

(**G**) Genome browser tracks depicting reads accumulation of HCFC1 and E2F1 on representative genes in control and *Kdm2a* cKO c-KIT-positive cells using IGV software.

Appendix Figure S4

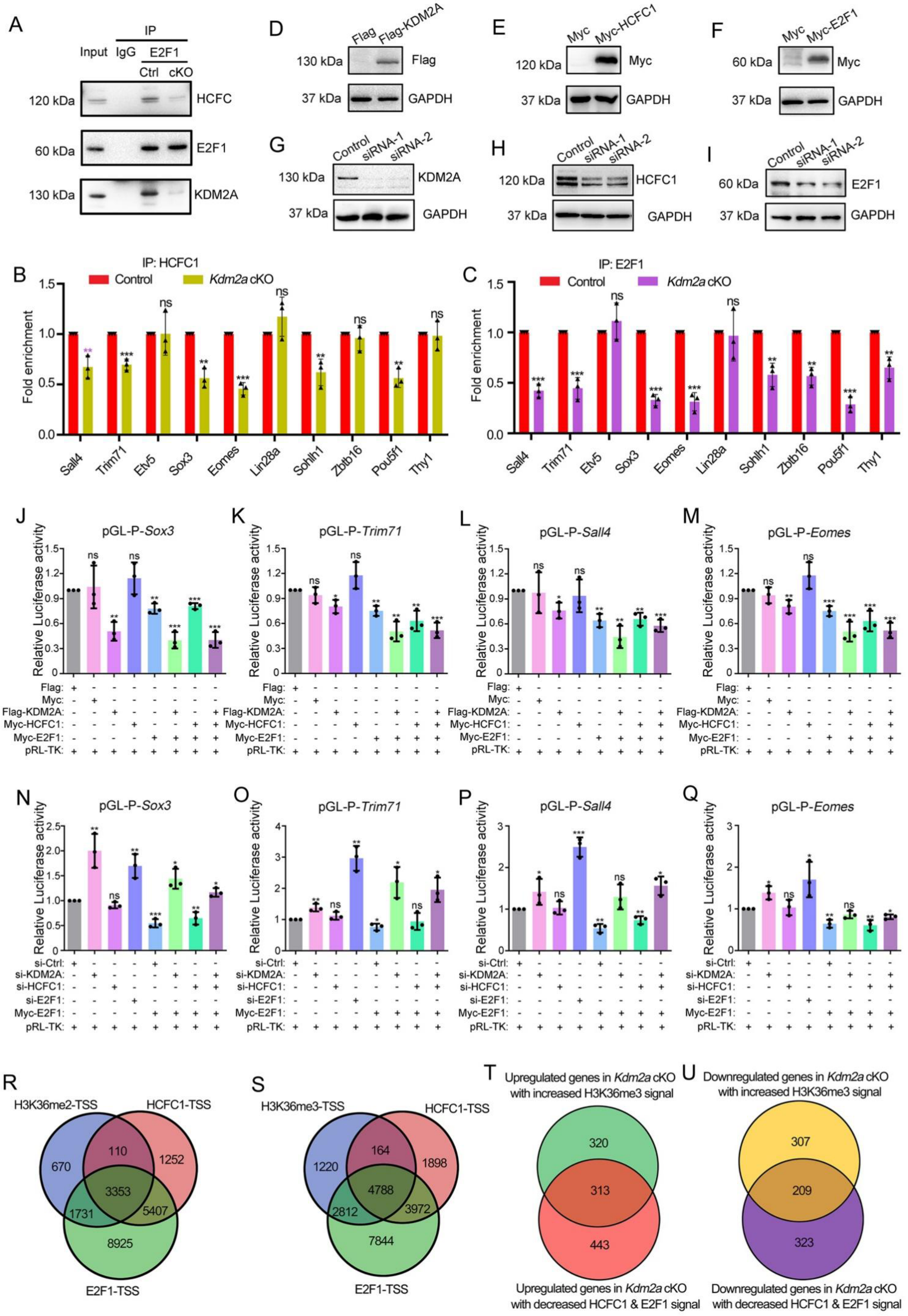

Appendix Figure S4. KDM2A and E2F1 regulate the expression of downregulated genes.

(**A**) IP analysis was performed with anti-E2F1 anbody. The protein levels of immunoprecipitated KDM2A, HCFC1 and E2F1 in WT and *Kdm2a* cKO c-KIT positive cells were checked by WB assay.

(**B-C**) RIP-qPCR analyses of the association of the selected gene mRNAs with HCFC1(L) and E2F1(M) in control and *Kdm2a* cKO c-KIT-positive cells are shown. Biologically independent mice (n = 3) were examined in three separate experiments. Data were presented as mean ± SD. ns, not significant, $^{**}P < 0.01$, $^{***}P < 0.001$.

(**D-F**) Immunoblotting analyses the overexpression of KDM2A (A), HCFC1 (B), and E2F1 (C) were transfected into HEK293T cells, respectively. GAPDH served as a loading control.

(**G-I**) Immunoblotting analyses the knockdown of KDM2A (E), HCFC1 (F), and E2F1 (G) were transfected into HEK293T cells, respectively. GAPDH served as a loading control.

(**J-M**) Luciferase reporter assays show that when *Kdm2a* and *E2f1* were overexpressed in different combinations, the luciferase activity of the *Sox3* (N), *Trim71* (O), *Sall4* (P), and *Eomes* (Q) promoter regions in HEK293T cells was significantly increased. Data were presented as mean ± SD. n = 3. ns, not significant, $^{*}P < 0.05$, $^{**}P < 0.01$, $^{***}P < 0.001$.

(**N-Q**) Luciferase reporter assays show that when *Kdm2a* and *E2f1* were knocked down in different combinations, the luciferase activity of the *Sox3* (R), *Trim71* (S), *Sall4* (T), and *Eomes* (U) promoter regions in HEK293T cells was remarkable decreased. Data were presented as mean ± SD. n = 3. ns, not significant, $^{*}P < 0.05$, $^{**}P < 0.01$, $^{***}P < 0.001$.

(**R**) Venn diagram showing overlap in the H3K36me2 TSS, HCFC1 TSS, and E2F1 TSS of *Kdm2a* cKO c-KIT-positive cells.

(**S**) Venn diagram showing overlap in the H3K36me3 TSS, HCFC1 TSS, and E2F1 TSS of *Kdm2a* cKO c-KIT-positive cells.

(**T**) Venn diagram showing the overlap of upregulated genes with increased H3K36me3 signal and upregulated genes with decreased HCFC1&E2F1 signal in *Kdm2a* cKO c-KIT-positive cells.

(**U**) Venn diagram showing the overlap of downregulated genes with increased H3K36me3 signal and downregulated genes with decreased HCFC1&E2F1 signal in *Kdm2a* cKO c-KIT-positive cells.

Appendix Table S1.

**Primer sequences are used in this study.**		
Target	**Sequence (5' to 3')**	**Application**
STRA8GFP-cre	F: ACTCCAAGCACTGGGCAGAA	Genotyping
	R1: GCCACCATAGCAGCATCAAA	
	R2: CGTTTACGTCGCCGTCCAG	
KDM2A-loxp	F: CAACCTGACCCTAATTGTACAGC	Genotyping
	R: CCTGGTGTGGCTAAACTCT	
Gapdh	F: AGGTCGGTGTGAACGGATTTG	qPCR
	R: GGGGTCGTTGATGGCAACA	
Stra8	F: AGGCTGTTGGACCAGATGCT	RT-qPCR
	R: CATGTGCAGAGATGATGCTGT	
Stra8	F: CTGCGACTACAGCTATGATCCACC	CHIP-qPCR
	R: AGCCCCTTTCTAAAGTATGCAACAGG	
Meiosin	F: CCCGTGAAGCCCAAAGACAGC	RT-qPCR
	R: GGTCTCCCCATCGTCGTCCTCA	
Meiosin	F: ACTTTGTTTGCAGCTACAGGAACTGGCAT	CHIP-qPCR
	R: CTCCTGTGTCCCCATCCGACCCTT	
SPO11	F: GATGCAACATTTCAGCGGCTCC	RT-qPCR
	R: CACATTATCTCGATGCCGTAGGGA	
SPO11	F: TCCCTTCTCCCATACCTATCCCCA	CHIP-qPCR
	R: AGGGGAAATAATACTTGCAAAGCTCT	
SYCP1	F: TCCTTCACAAAATATATCTCGGCTT	RT-qPCR
	R: AACATCAAATTCAAAGGCTGTTT	
SYCP1	F: ATGAAACACTCTATAACACGCACCAG	CHIP-qPCR
	R: GTTCTCTACCAAGTCTGCGCTCA	
Meioc	F: CCCATCTAGAGTTGATCGGTT	RT-qPCR
	R: AACAATTTCATCCTTTCTACGTGA	
Ythdc2	F: TTCCCTTTCAGTTTGCACACC	RT-qPCR
	R: TGGCATACAACACTTCATCAAGG	
Hormad1	F: CAAAACAAGAAGCGGAAA	RT-qPCR
	R: GCACACCTTCAAATAATACAGC	
Dmc1	F: CATTCTGGCTCACGCTTCCAC	RT-qPCR
	R: TGTACCAATTCCCCACCTACTCC	
Mus81	F: ACACCGTACGCAAGCTACACGTTGGA	RT-qPCR
	R: AAGGCTAAGGTTGTGGACAGACCCAT	
Syce1	F: CCTGCTCCACGGGCCTGA	RT-qPCR
	R: CACTGGCTTTCTTTTTTGGTAAGTCC	
Pou5f1	F: CCAGACCACCATCTGTCGCTTCGAG	RT-qPCR
	R: TCCAGACTCCACCTCACACGGTT	
Gfra1	F: CTGAGAATGAGATTCCCACACACGTT	RT-qPCR
	R: TGAACACCATCACCGGCAGT	
Etv5	F:GGCATGGAATTTAAGCTCATAGAACCGGAA	RT-qPCR
	R:TAAGCAGGGTTGTCTTCAAAGTGAGTCAGA	

Lin28a	F: AGTTCACCTTTAAGAAGTCTGCCAAGGGTC<br>R: AGCTTGCATTCCTTGGCATGATGGTCT	RT-qPCR
Eomes	F: ACCTTCTCAGAGACACAGTTCATCGC<br>R: TTTGAACGCCGTACCGACCTCC	RT-qPCR
Ngn3	F: CACTGCTGCTTGACACTGACCC<br>R: AATTGGAACTGAGCACTTCGTGG	RT-qPCR
Sohlh1	F: CGGGGACCCTGAATCTTCCGGCAT<br>R: CAGGGTCTCCGATGAAGCTTGGCTCT	RT-qPCR
Thy1	F: CTTGGCACCATGAACCCAG<br>R: ACGTGCTTCCTCTTCTCTCGG	RT-qPCR
Zbtb16	F: CAACCGCACCTTCCCCAGCCACACG<br>R: CCAACTGGTGCTTGAGGCTGAACT	RT-qPCR
Sall4	F: CCCCAGCATGCCCTCGGGTA<br>R: AGCACTCAGCGCGGCCAT	RT-qPCR
Meioc	F: ATCTCCCAACCACCGCTACCAAGCC<br>R: AAGGAGCAGTTAGCCGACTTCGCCCTA	ChIP-qPCR
Dmc1	F: AAAACTATCCACGGGAGATAGGT<br>R: CTGTTTCCTTTTCCCCTTAGATGG	ChIP-qPCR
Cpeb1	F: CTCCTTGAATGTGATTTGGGCACT<br>R: GTCTCAAACAAGATATGCCGCTGA	ChIP-qPCR
Tex15	F: TCAGCCTCTAAAGTGATGAGATTACAAGC<br>R: ATCCTATAAACCCTTTCTCTATAGCCAT	ChIP-qPCR
Zfp541	F: AAATAAATAAGCCAGTCCTGCAGTTC<br>R: GGACACCCTCCAGTATAGATCCC	ChIP-qPCR
Stra8	F: AGACAAGGTGGTGAGAACTGAG<br>R: ACTTAGCTTTCTCTCGTGTAAGGAC	pGL4.10 Plasmid construction
Meiosin	F: TGCAGGTCACAAAAGAGAATAAGGAAC<br>R: TCCCGACGCGCAGACCGAG	pGL4.10 Plasmid construction
Spo11	F: ACACCGCAATGCTCACAC<br>R: CGAACCTTTCCGCGCTCT	pGL4.10 Plasmid construction
Sycp1	F: TTATTTTGACATGGCTGCACTCCC<br>R: CGGCTCAATTTAACGGTTGGTC	pGL4.10 Plasmid construction
KDM2A full length	F: ATGGAACCTGAAGAAGAAAGGATTCG<br>R: TTAGCTAATCTTCTGTATCAGTTTCTCAT	Overexpression plasmid construction
HCFC1 full length	F: ATGGCTTCGGCTGTGTCTCCC<br>R: TCACTGACCATCAGCCTTAGACT	overexpression plasmid construction
E2F1 full length	F: ATGGCCGTAGCCCCCGCG<br>R: TCAGAAATCCAGAGGGGTCAGGT	overexpression plasmid construction
KDM2A-siRNA-1	5'-GAACCCGAAGAAGAAAGGAUUCGUU-3'	knockdown experiment
KDM2A-siRNA-2	5'-GAGGAGGAGCGAGAGAAACUCUAUA-3'	knockdown experiment
E2F1-siRNA-1	5'-UAUCUGUACUACGCAGCUGTT-3'	knockdown experiment
E2F1-siRNA-2	5'-GGACCUUCGUAGCAUUGCATT-3'	knockdown experiment
HCFC1-siRNA-1	5'-CCCUAUCAUCACAGUGCACAATT-3'	knockdown experiment
HCFC1-siRNA-2	5'-GCCCGCAAUGAGAAGGGCUAUTT-3'	knockdown experiment
HCFC1-siRNA-3	5'-GCAACCACCAUCGGAAAUAAATT-3'	knockdown experiment
NC-siRNA	5'-UUCUCCGAACGUGUCACGUTT-3'	knockdown experiment

Appendix Table S2.

Antibodies used in this study.					
**Antibodies**	**Species**	**Applications**	**Concentration**	**Sources**	**Cat #**
DDX4/MVH	Rabbit	IF	1:400	Abcam	ab13840
SYCP3	Rabbit	IF	1:200	Abcam	ab15093
SYCP3	Mouse	IF	1:100	Santa	sc-74569
SYCP3	Mouse	WB	1:100	Santa	sc-74569
rH2AX	Mouse	IF	1:200	Abcam	ab26350
rH2AX	Rabbit	IF	1:200	Abcam	ab11174
PLZF	Mouse	IF	1:100	Santa	SC-28391
STRA8	Rabbit	IF	1:200	Milipore	ABN1656
STRA8	Rabbit	WB	1:2000	Milipore	ABN1656
Rad51	Rabbit	IF	1:400	Abcam	ab133534
c-kit	Goat	IF	1:200	R&D	AF1356
Dmc1	Rabbit	IF	1:200	Proteinech	13714-1-AP
Dmc1	Rabbit	WB	1:2000	Proteintech	13714-1-AP
Rec8	Rabbit	WB	1:1000	Proteintech	10793-1-AP
SYCE1	Rabbit	WB	1:1000	Proteintech	11063-1-AP
SYCP1	Rabbit	IF	1:200	abcam	ab15090
SYCP1	Rabbit	WB	1:1000	ABclonal	A12139
KDM2A	Rabbit	IF	1:1000	abcam	ab191387
KDM2A	Rabbit	WB	1:1000	abcam	ab191387
KDM2A	Rabbit	IP	1:100	Proteintech	24311-1-AP
H3K36me1	Rabbit	IF	1:500	ABclonal	A2364
H3K36me1	Rabbit	WB	1:1000	ABclonal	A5924
H3K36me2	Rabbit	WB	1:1000	ABclonal	A2365
H3K36me2	Rabbit	IP	1:100	Active Motif	39056
H3K36me2	Rabbit	IF	1:200	Active Motif	39056
H3K36me3	Rabbit	IF	1:500	ABclonal	A2366
H3K36me3	Rabbit	WB	1:1000	ABclonal	A2366
H3K36me3	Rabbit	IP	1:100	CST	4909
HCFC1	Rabbit	IP	1:100	CST	69690
HCFC1	Rabbit	WB	1:1000	CST	69690
E2F1	Rabbit	WB	1:1000	Proteintech	66515-1-Ig
E2F1	Rabbit	IP	1:100	Proteintech	66515-1-Ig
GAPDH	Rabbit	WB	1:5000	Proteintech	10494-1-AP
FLAG-tag	Rabbit	IP	1:100	Proteintech	20543-1-AP
Myc-tag	Rabbit	IP	1:50	Proteintech	16286-1-AP
FLAG-tag	Mouse	WB	1:3000	ABclonal	AE005
Myc-tag	Mouse	WB	1:3000	Proteintech	60003-2-Ig
Goat anti-rat IgG	Goat	WB	1:10000	Biosharp	BL002A
Goat anti-rat IgG	Goat	WB	1:10000	Thermo Fisher	62-9520
HRP Goat anti-mouse IgG	Goat	WB	1:10000	Abbkine	A21010-1
HRP Goat anti-rabbit IgG	Goat	WB	1:10000	Abbkine	A21020-1

Dylight 488 Goat anti-mouse IgG	Goat	IF	1:500	Abbkine	A23210
Dylight 594 Goat anti-rabbit IgG	Goat	IF	1:500	Abbkine	A23420
Alexa FluorTM 488 Goat anti-mouse (H+L)	Goat	IF	1:500	Invitrogen	A11029IgG
Alexa FluorTM 488 Goat anti-rabbit IgG (H+L)	Goat	IF	1:500	Invitrogen	A32731
Alexa FluorTM 594 Goat anti-rabbit IgG (H+L)	Goat	IF	1:500	Invitrogen	A11032
Cy3-conjugated Affinipure Goat anti-mouse IgG (H+L)	Goat	IF	1:300	Proteintech	SA00009-1
