# Supplementary figures and images for: Histone demethylase KDM2A recruits HCFC1 and E2F1 to orchestrate male germ cell meiotic entry and progression (part 1 of 4)
Source: EMBO J. 2024 Aug 19;43(19):4197–227. doi: 10.1038/s44318-024-00203-4 (PMC11448500; doi:10.1038/s44318-024-00203-4)

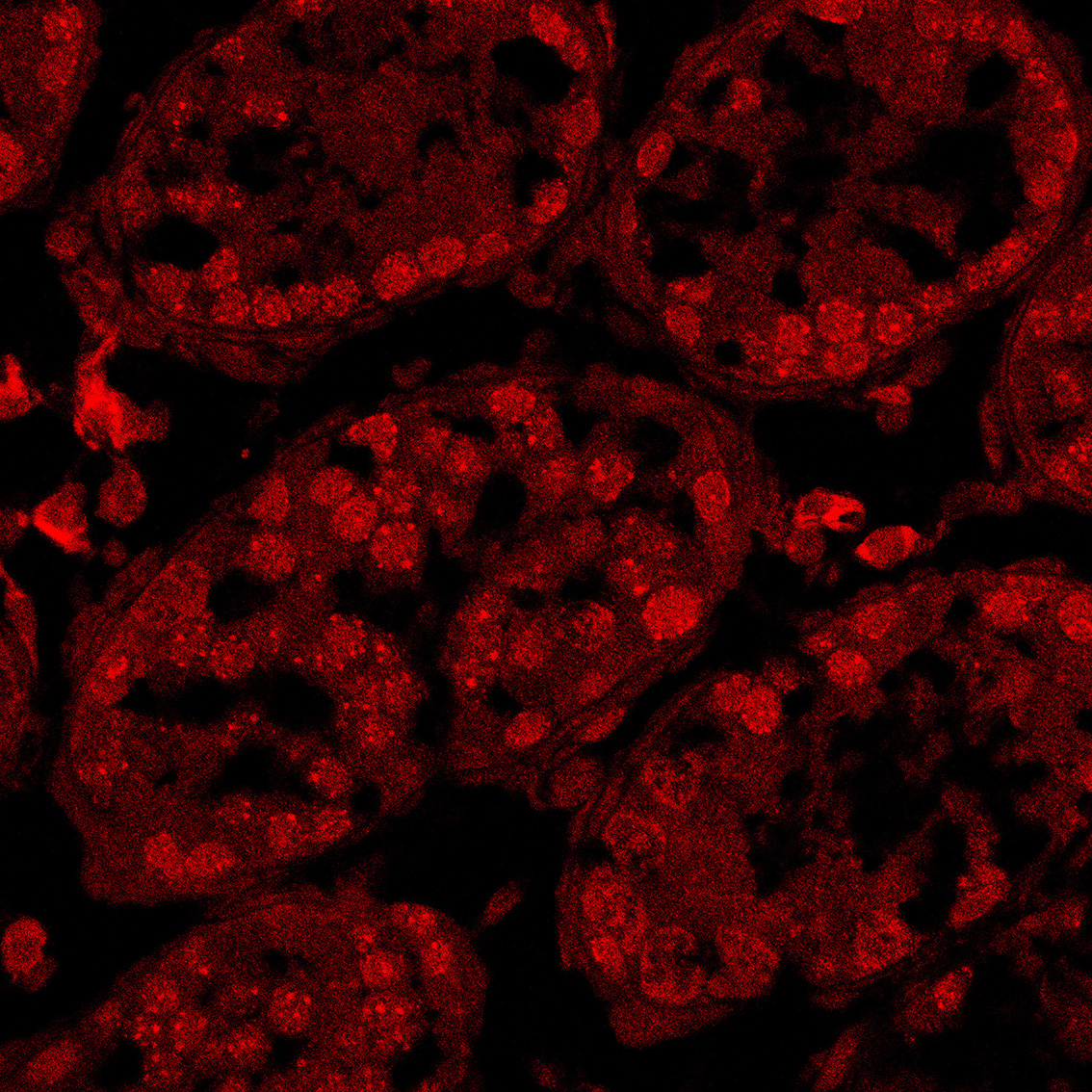

Supplement: Supplementary file 8 — Source data Fig. 1 [file 44318_2024_203_MOESM8_ESM.zip › Figure 1/Figure 1F/Kdm2a.jpg]

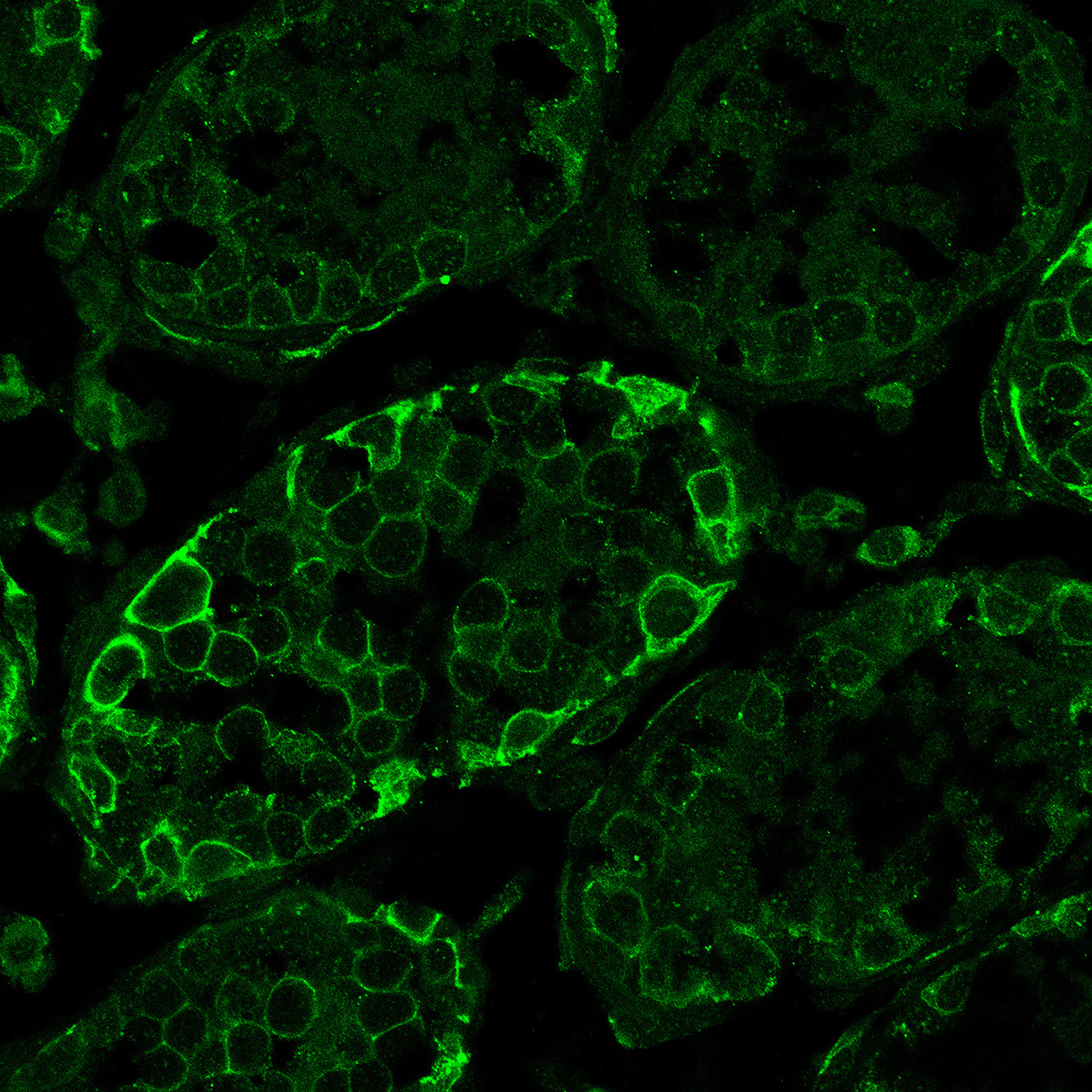

Supplement: Supplementary file 8 — Source data Fig. 1 [file 44318_2024_203_MOESM8_ESM.zip › Figure 1/Figure 1F/c-KIT.jpg]

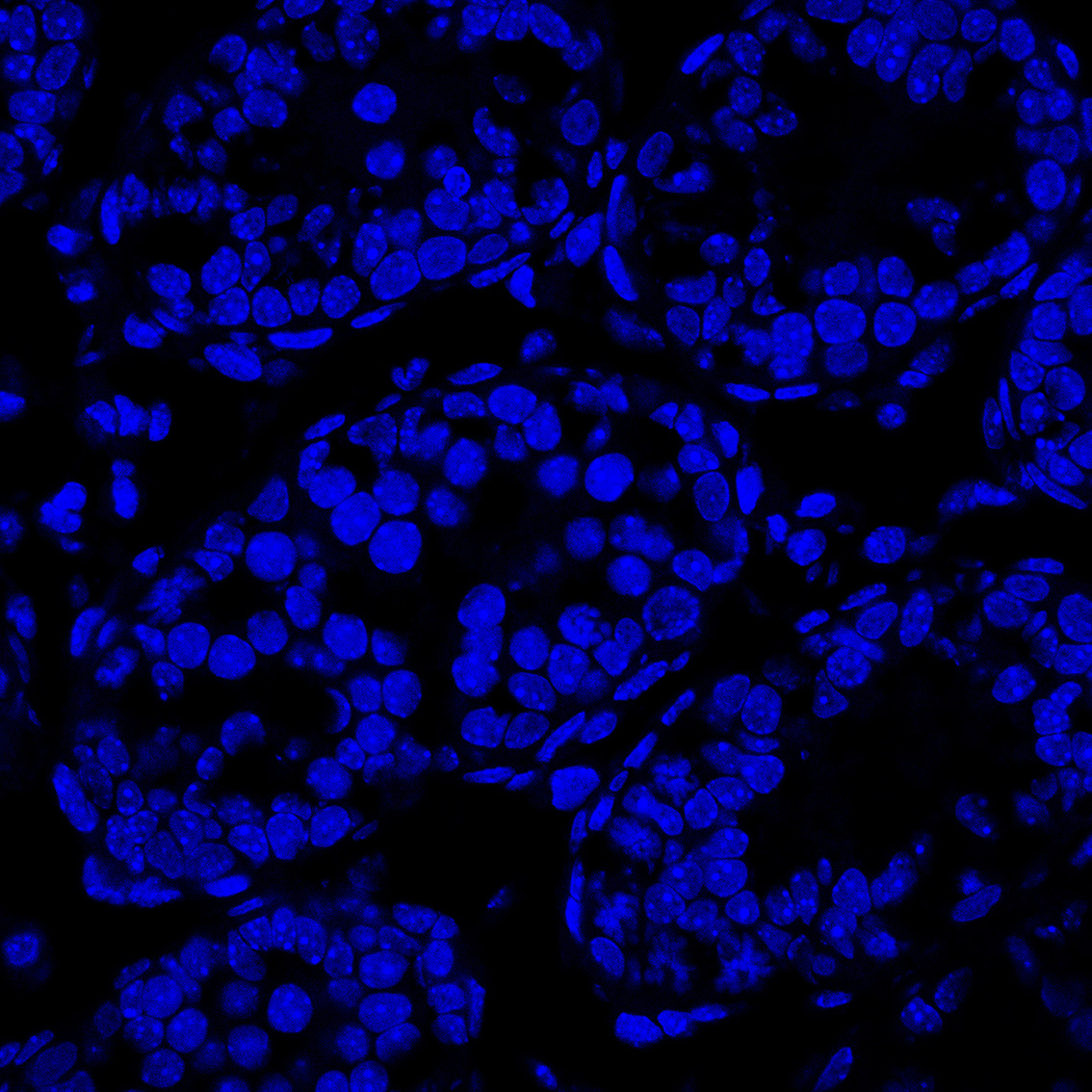

Supplement: Supplementary file 8 — Source data Fig. 1 [file 44318_2024_203_MOESM8_ESM.zip › Figure 1/Figure 1F/DAPI.jpg]

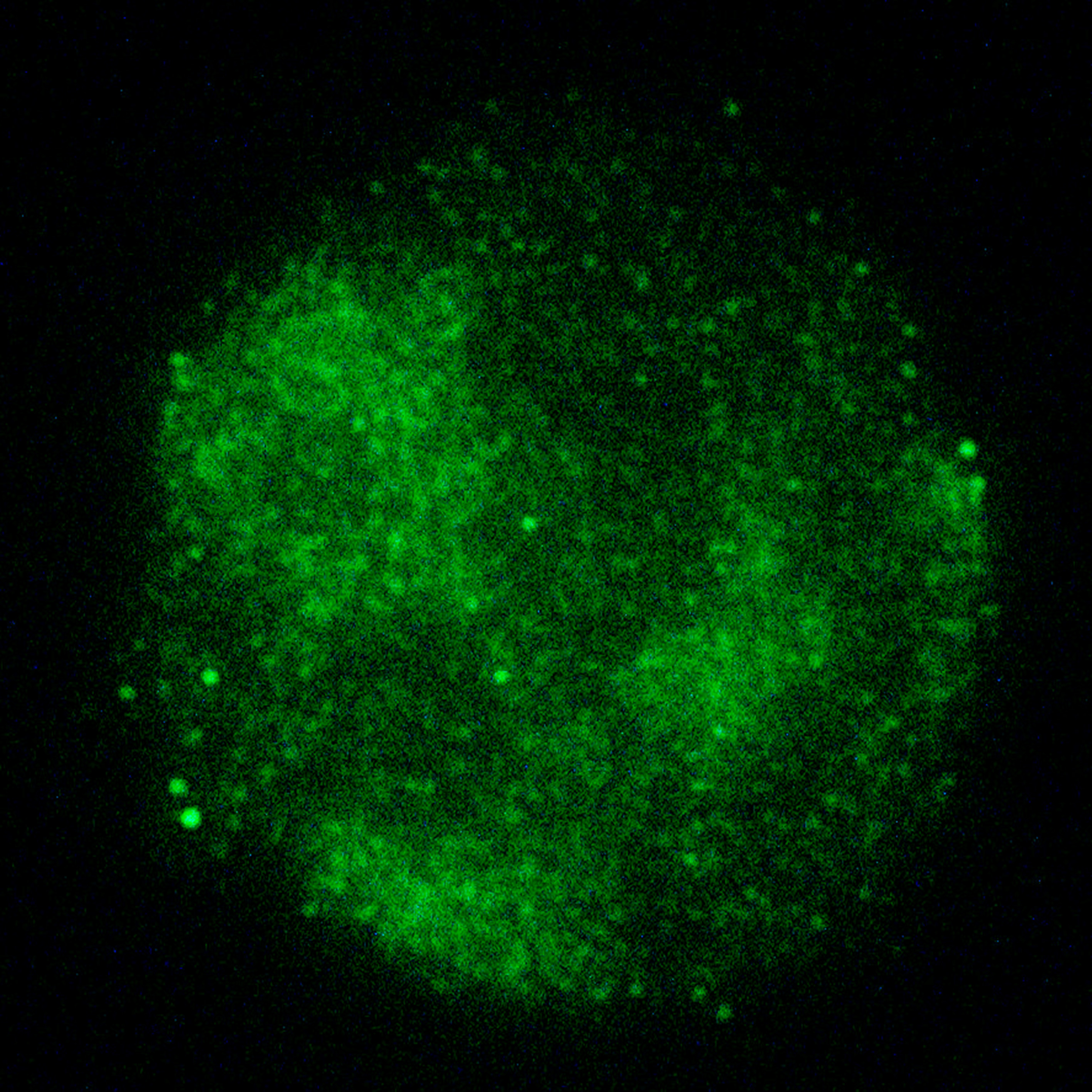

Supplement: Supplementary file 8 — Source data Fig. 1 [file 44318_2024_203_MOESM8_ESM.zip › Figure 1/Figure 1G/Zyg-KDM2A.jpg]

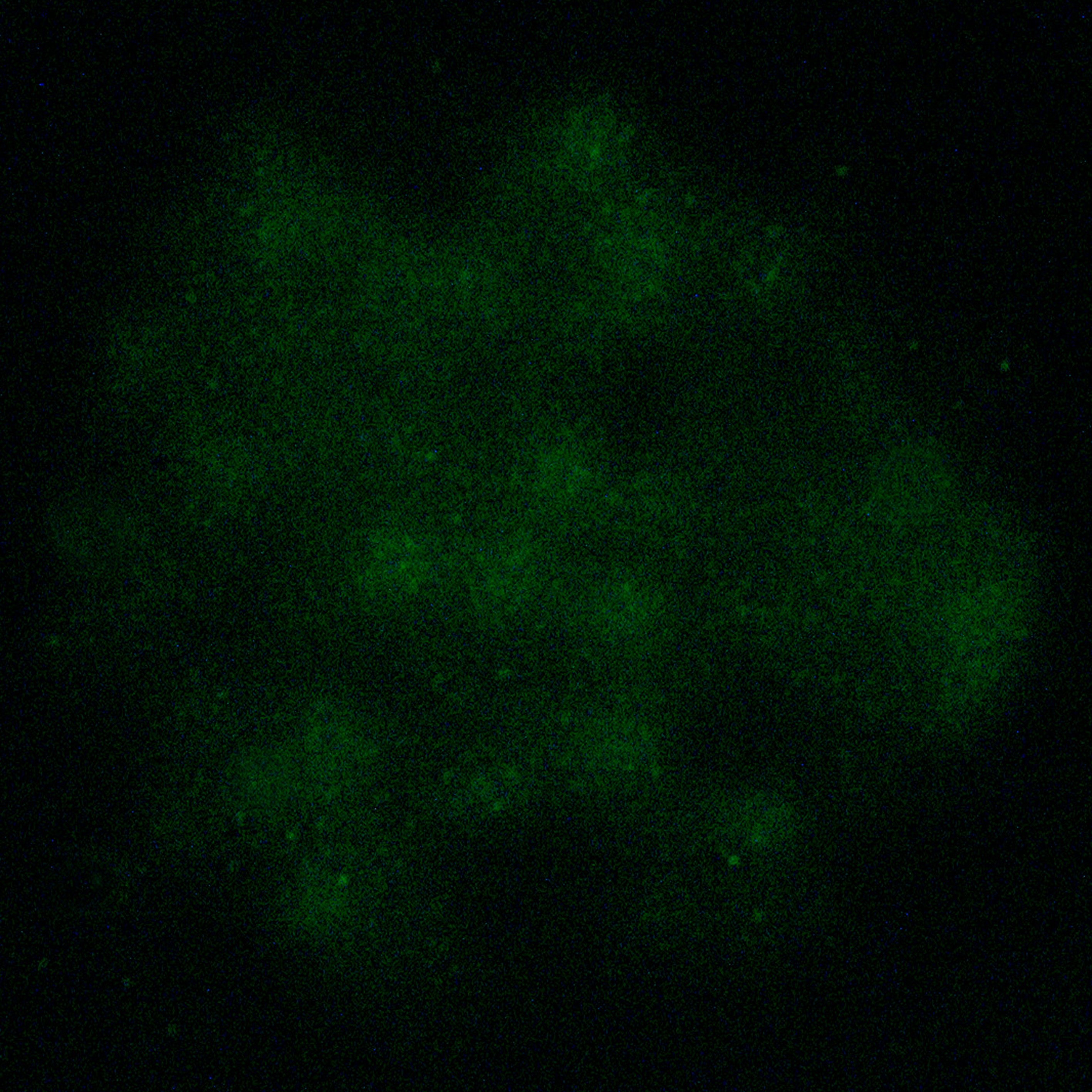

Supplement: Supplementary file 8 — Source data Fig. 1 [file 44318_2024_203_MOESM8_ESM.zip › Figure 1/Figure 1G/Pac-KDM2A.jpg]

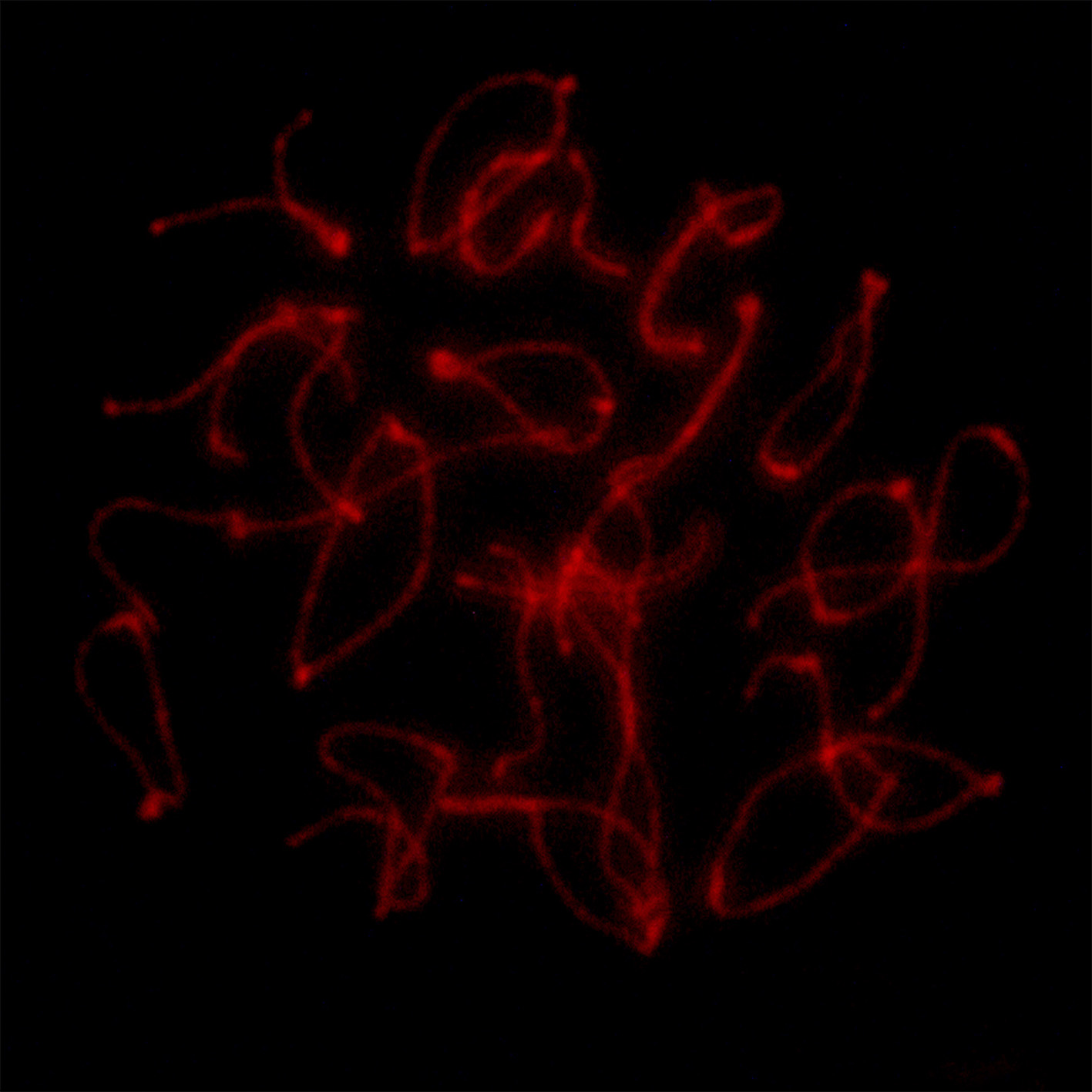

Supplement: Supplementary file 8 — Source data Fig. 1 [file 44318_2024_203_MOESM8_ESM.zip › Figure 1/Figure 1G/Dip-SYCP3.jpg]

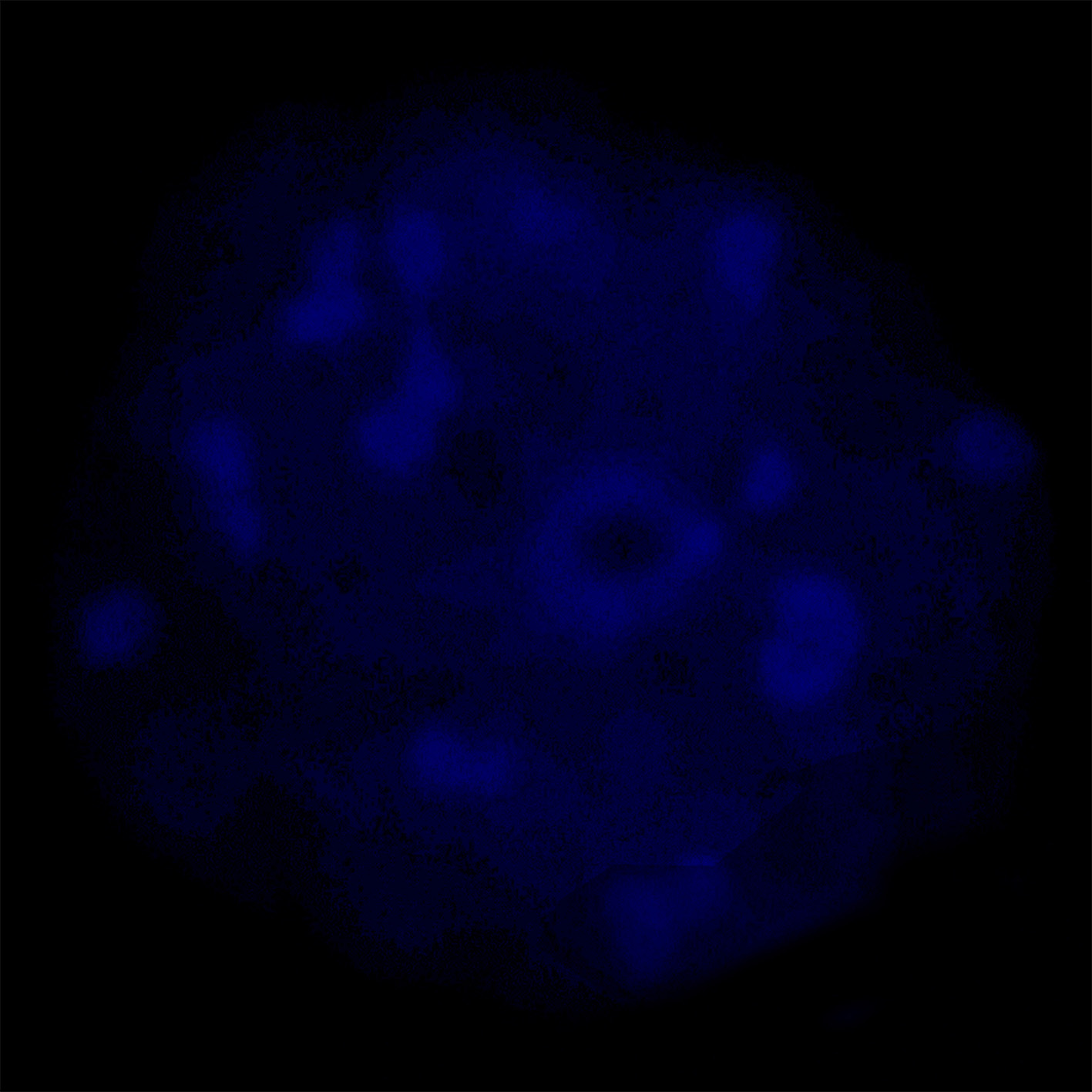

Supplement: Supplementary file 8 — Source data Fig. 1 [file 44318_2024_203_MOESM8_ESM.zip › Figure 1/Figure 1G/Dip-DAPI.jpg]

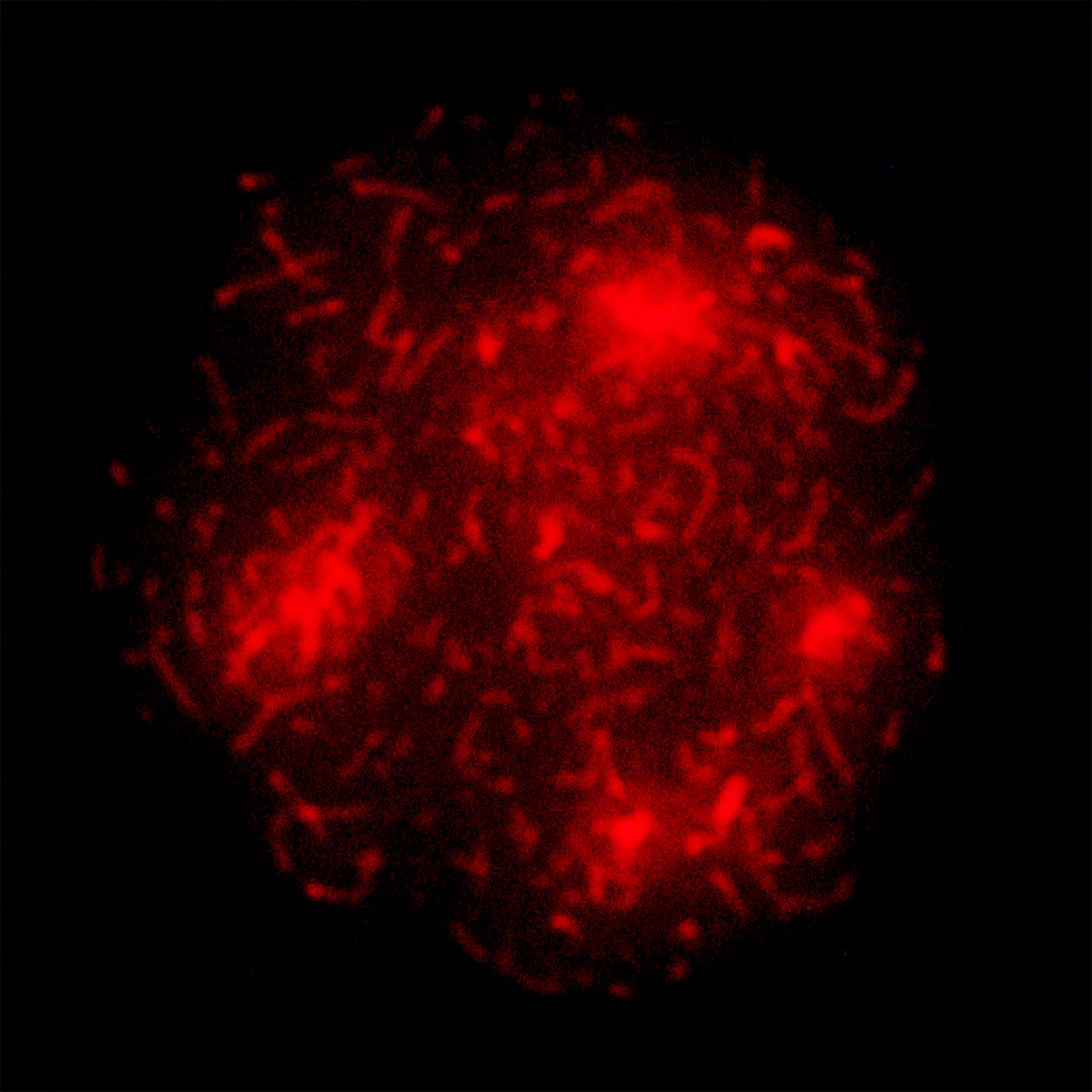

Supplement: Supplementary file 8 — Source data Fig. 1 [file 44318_2024_203_MOESM8_ESM.zip › Figure 1/Figure 1G/Lep-SYCP3.jpg]

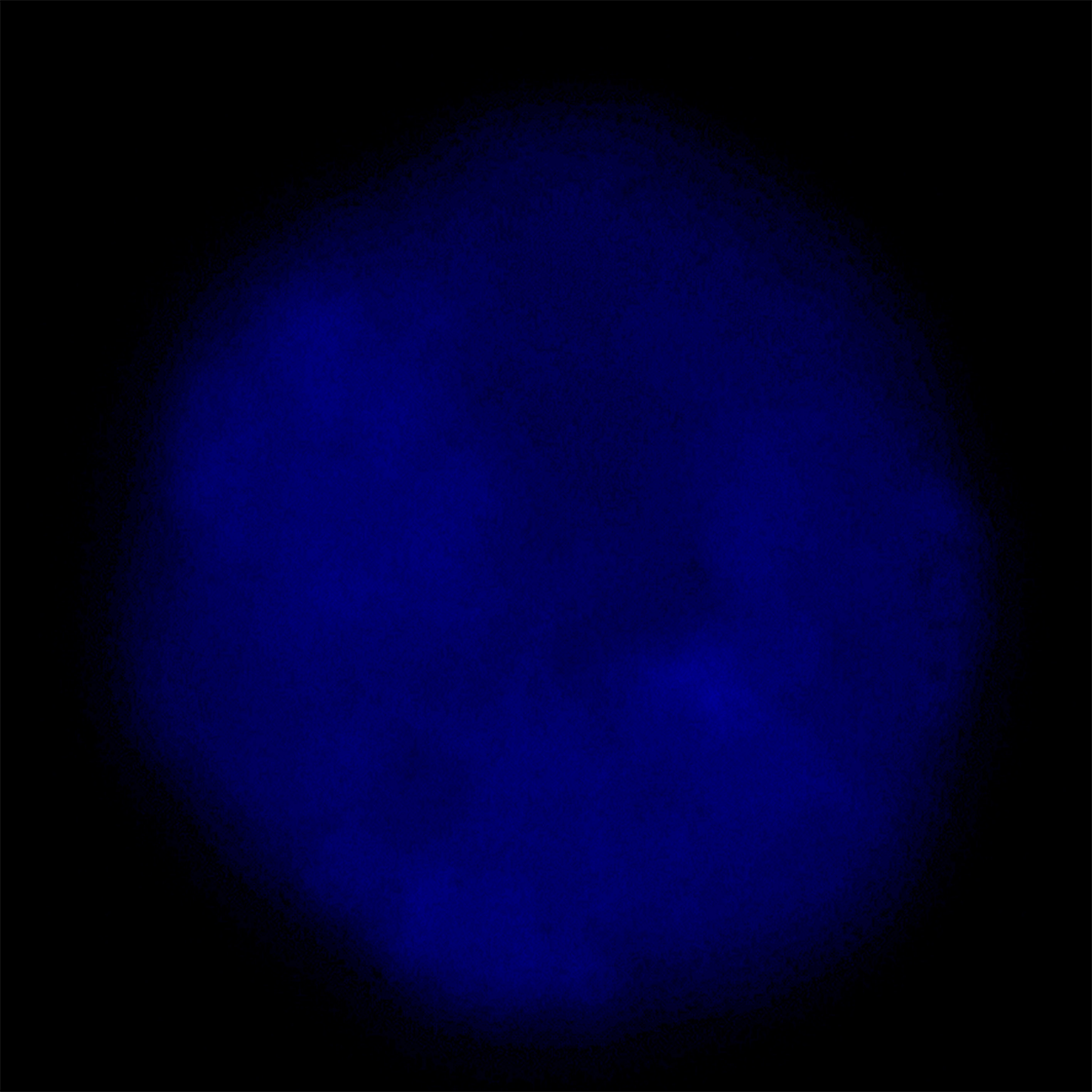

Supplement: Supplementary file 8 — Source data Fig. 1 [file 44318_2024_203_MOESM8_ESM.zip › Figure 1/Figure 1G/Zyg-DAPI.jpg]

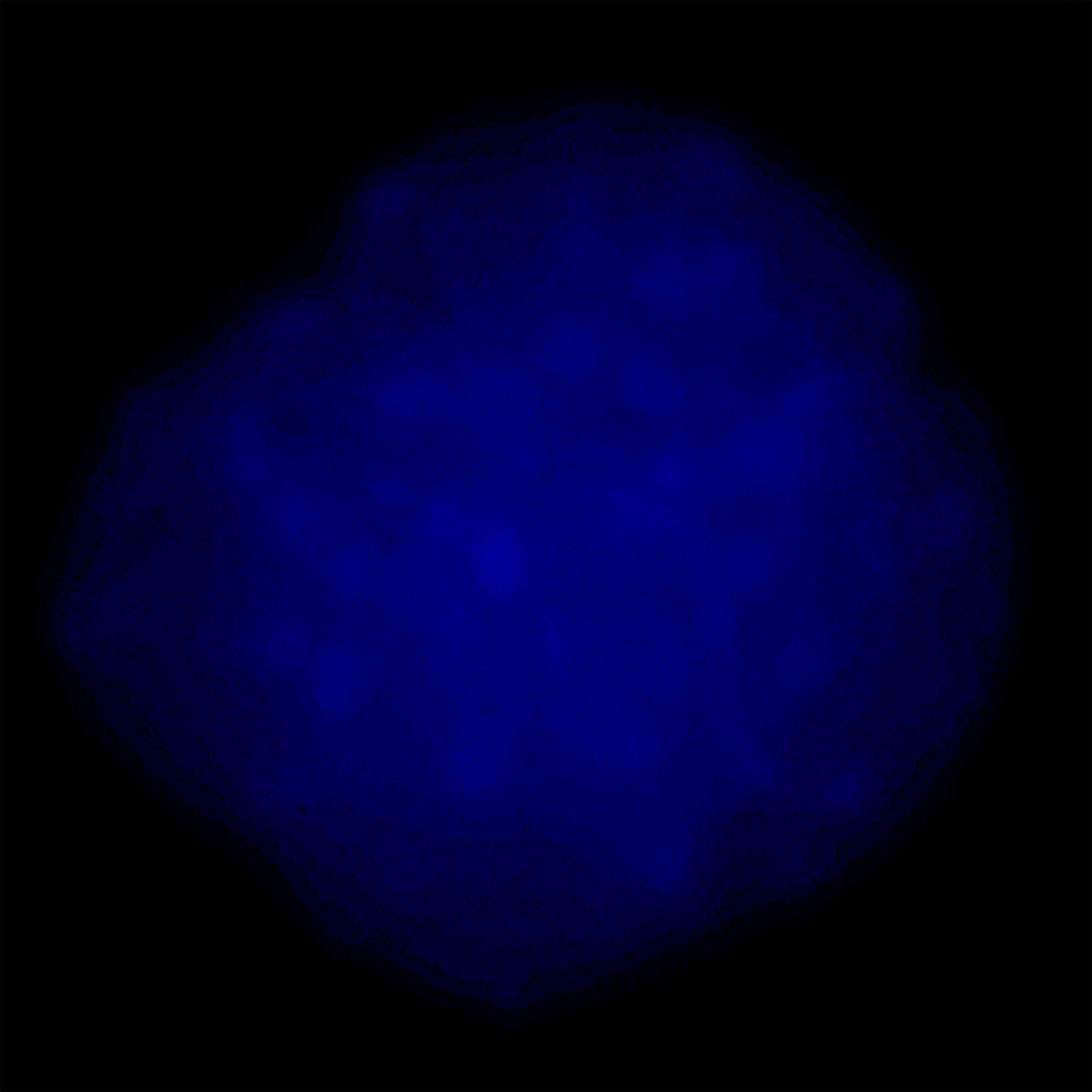

Supplement: Supplementary file 8 — Source data Fig. 1 [file 44318_2024_203_MOESM8_ESM.zip › Figure 1/Figure 1G/Prelep-DAPI.jpg]

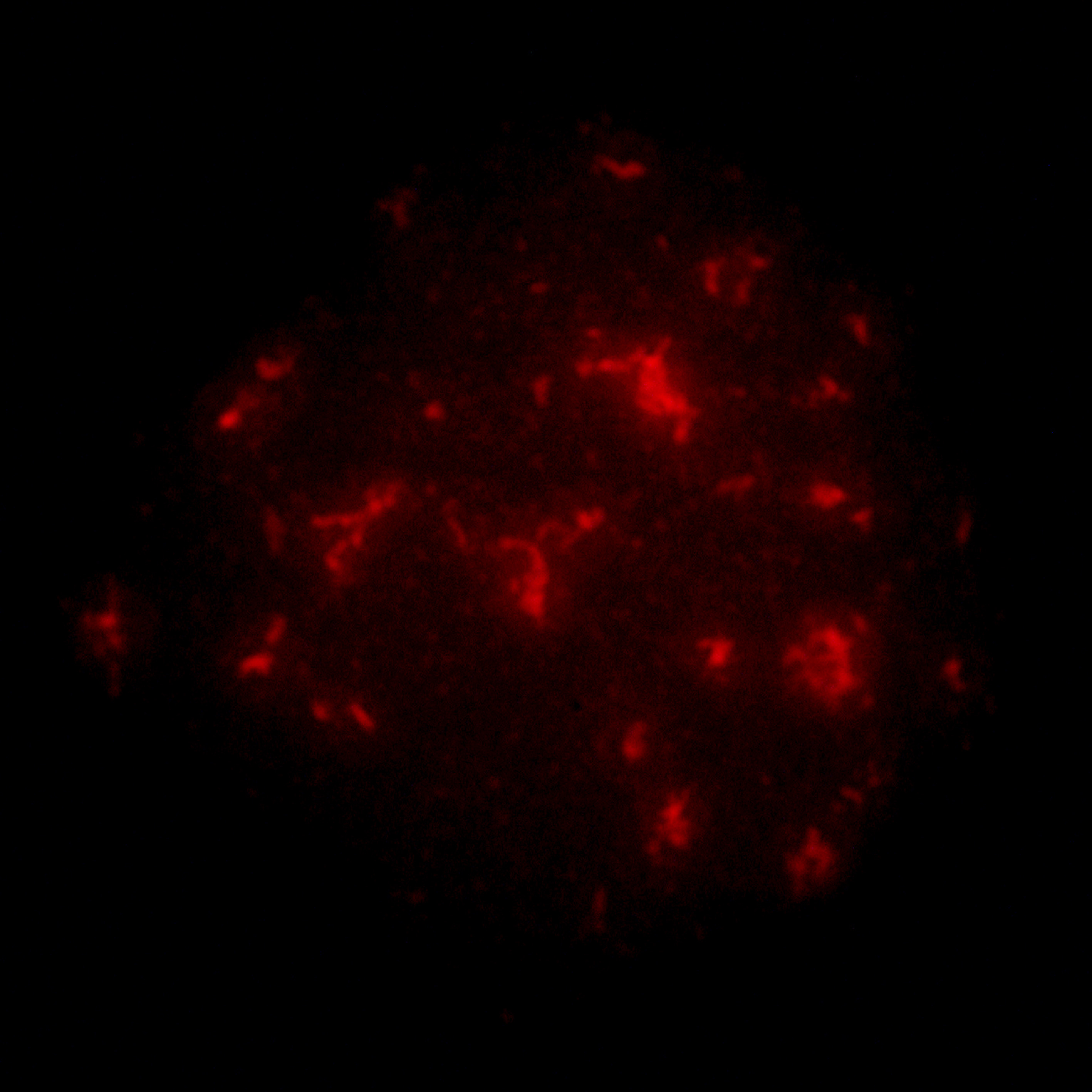

Supplement: Supplementary file 8 — Source data Fig. 1 [file 44318_2024_203_MOESM8_ESM.zip › Figure 1/Figure 1G/Prelep-SYCP3.jpg]

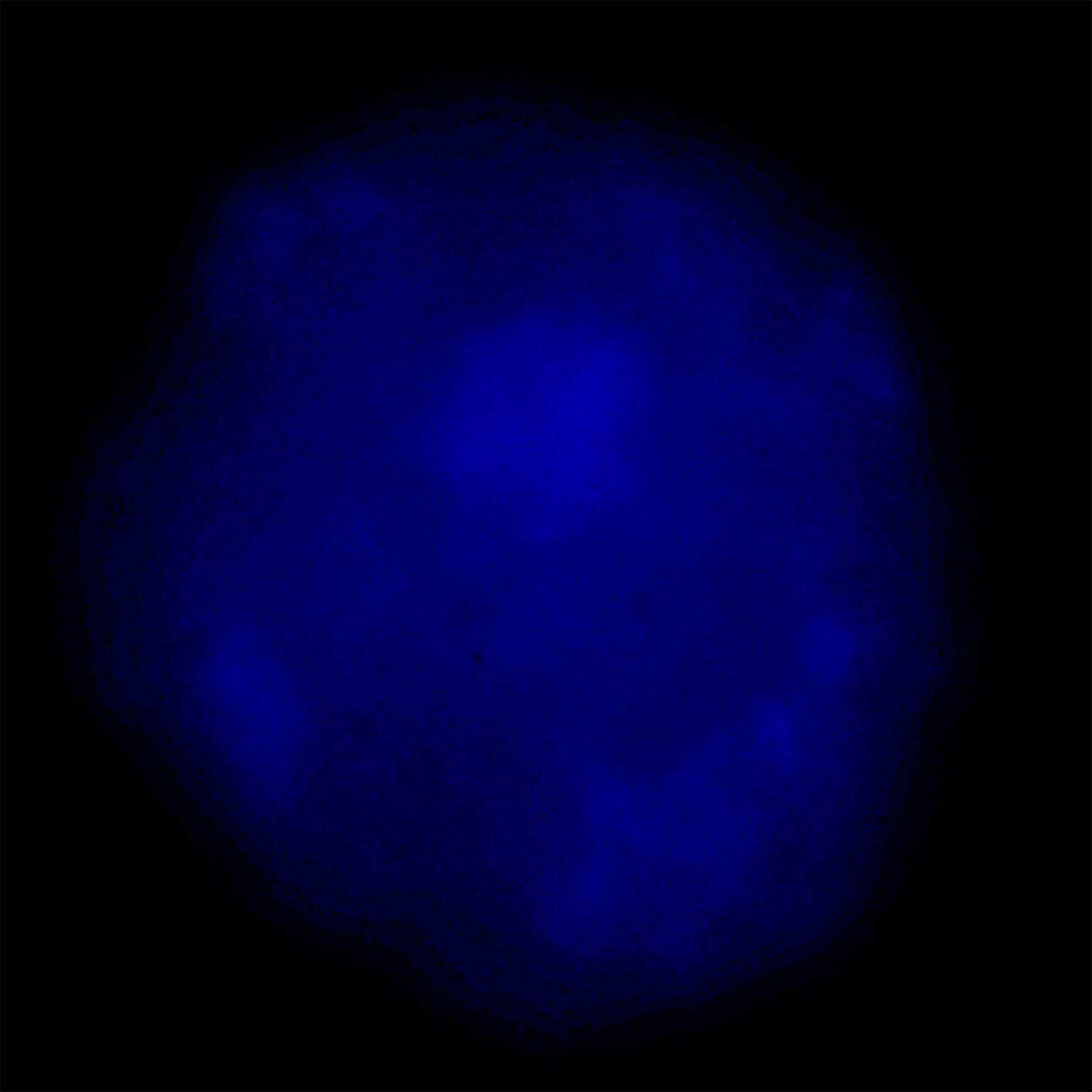

Supplement: Supplementary file 8 — Source data Fig. 1 [file 44318_2024_203_MOESM8_ESM.zip › Figure 1/Figure 1G/Lep-DAPI.jpg]

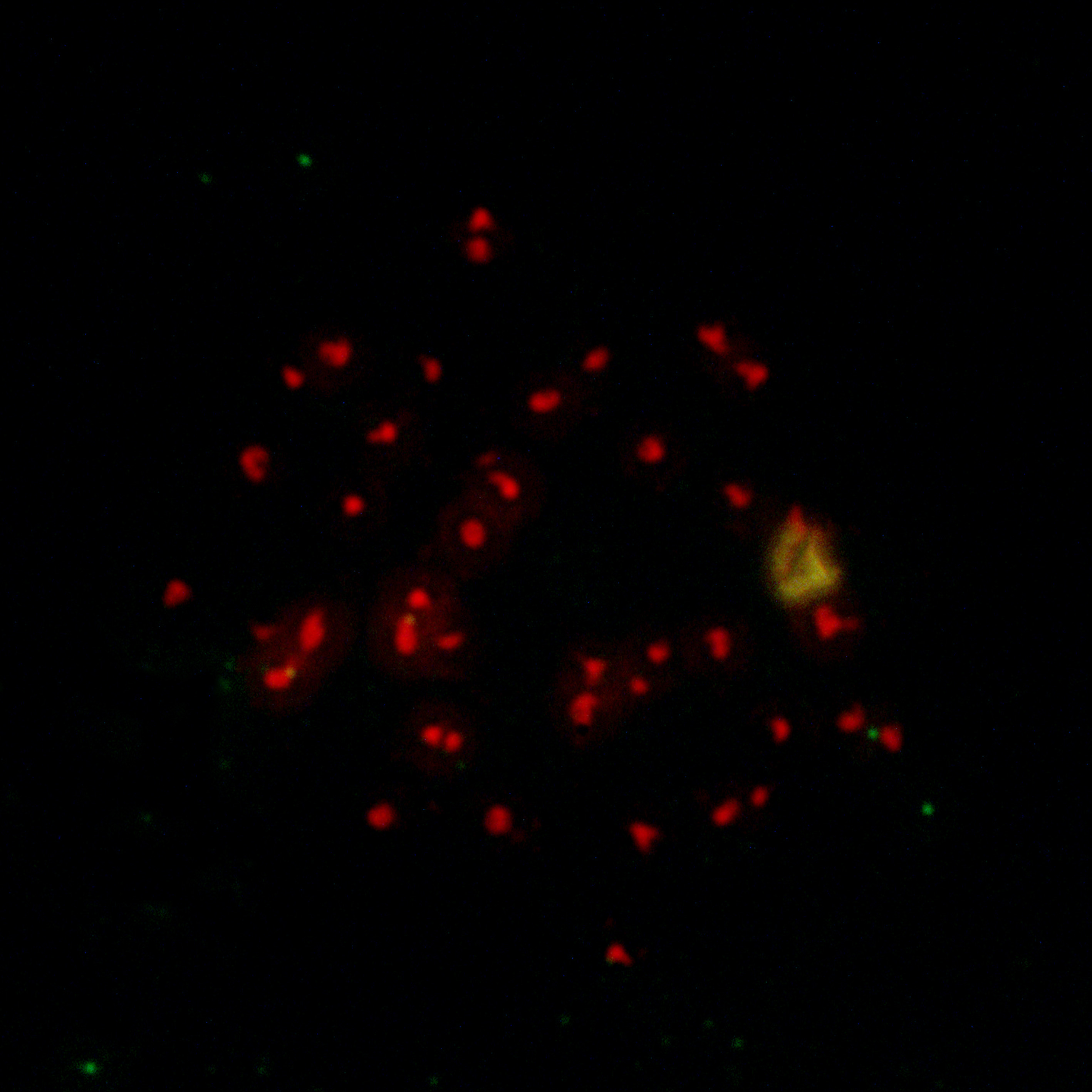

Supplement: Supplementary file 8 — Source data Fig. 1 [file 44318_2024_203_MOESM8_ESM.zip › Figure 1/Figure 1G/Meta-SYCP3.jpg]

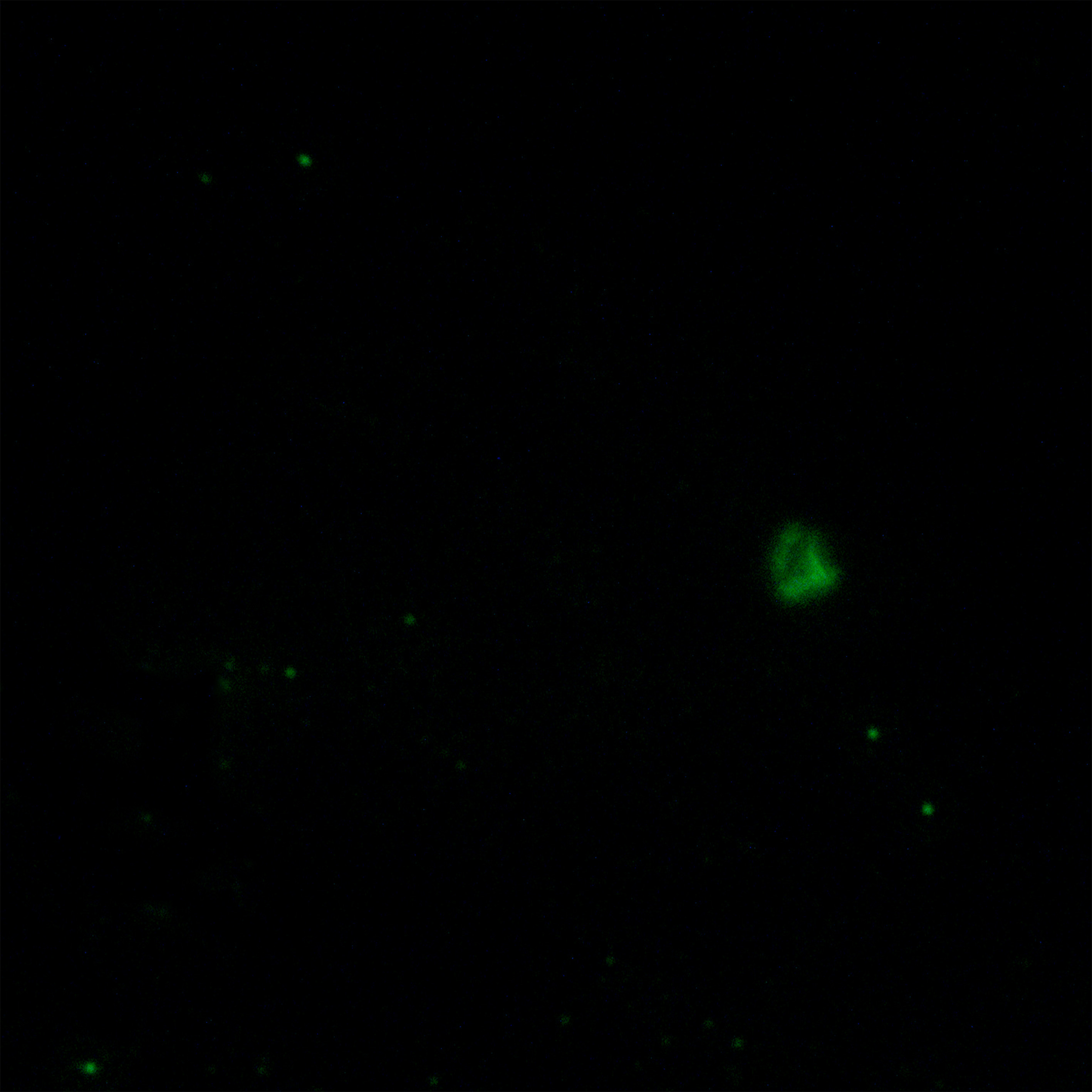

Supplement: Supplementary file 8 — Source data Fig. 1 [file 44318_2024_203_MOESM8_ESM.zip › Figure 1/Figure 1G/Meta-KDM2A.jpg]

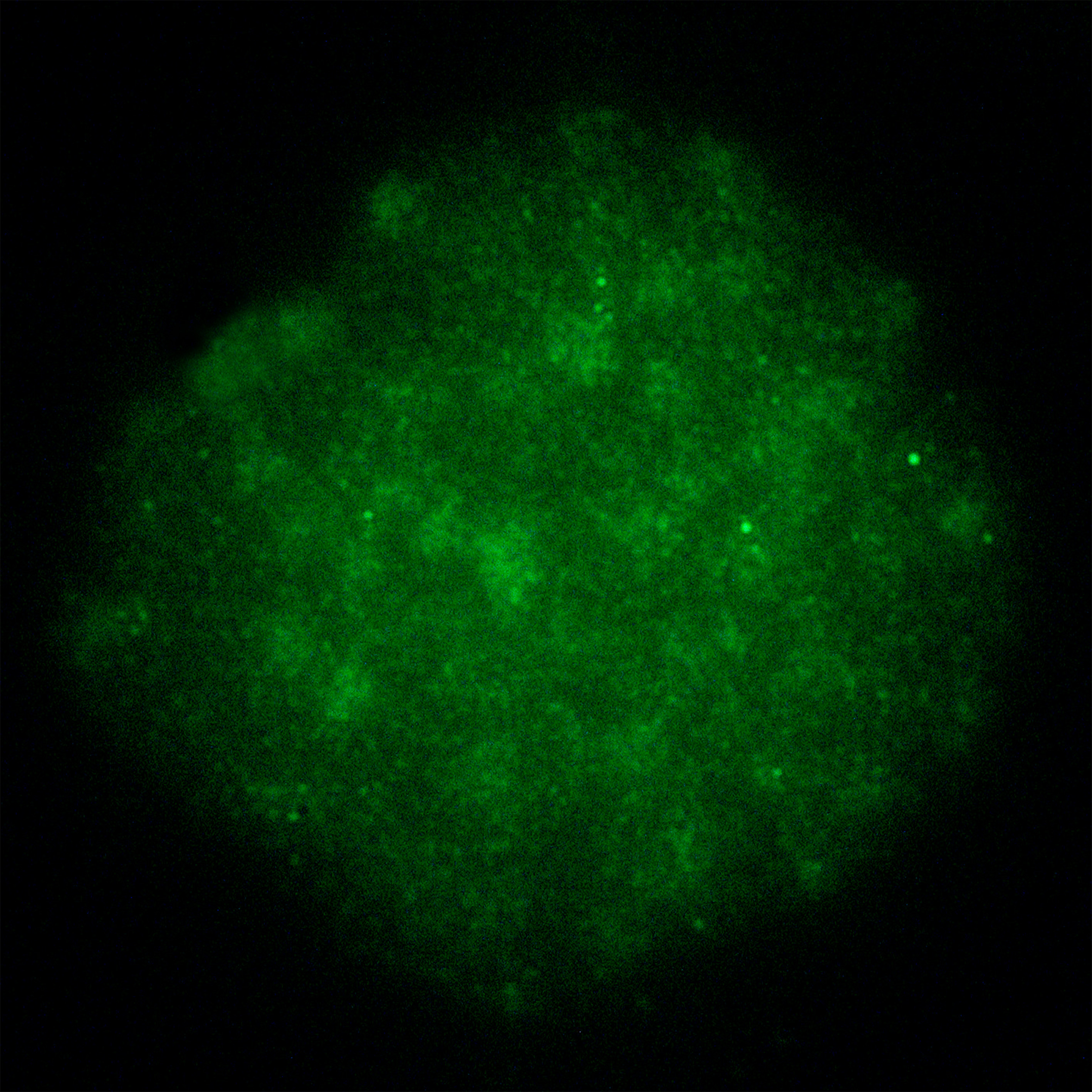

Supplement: Supplementary file 8 — Source data Fig. 1 [file 44318_2024_203_MOESM8_ESM.zip › Figure 1/Figure 1G/Prelep-KDM2A.jpg]

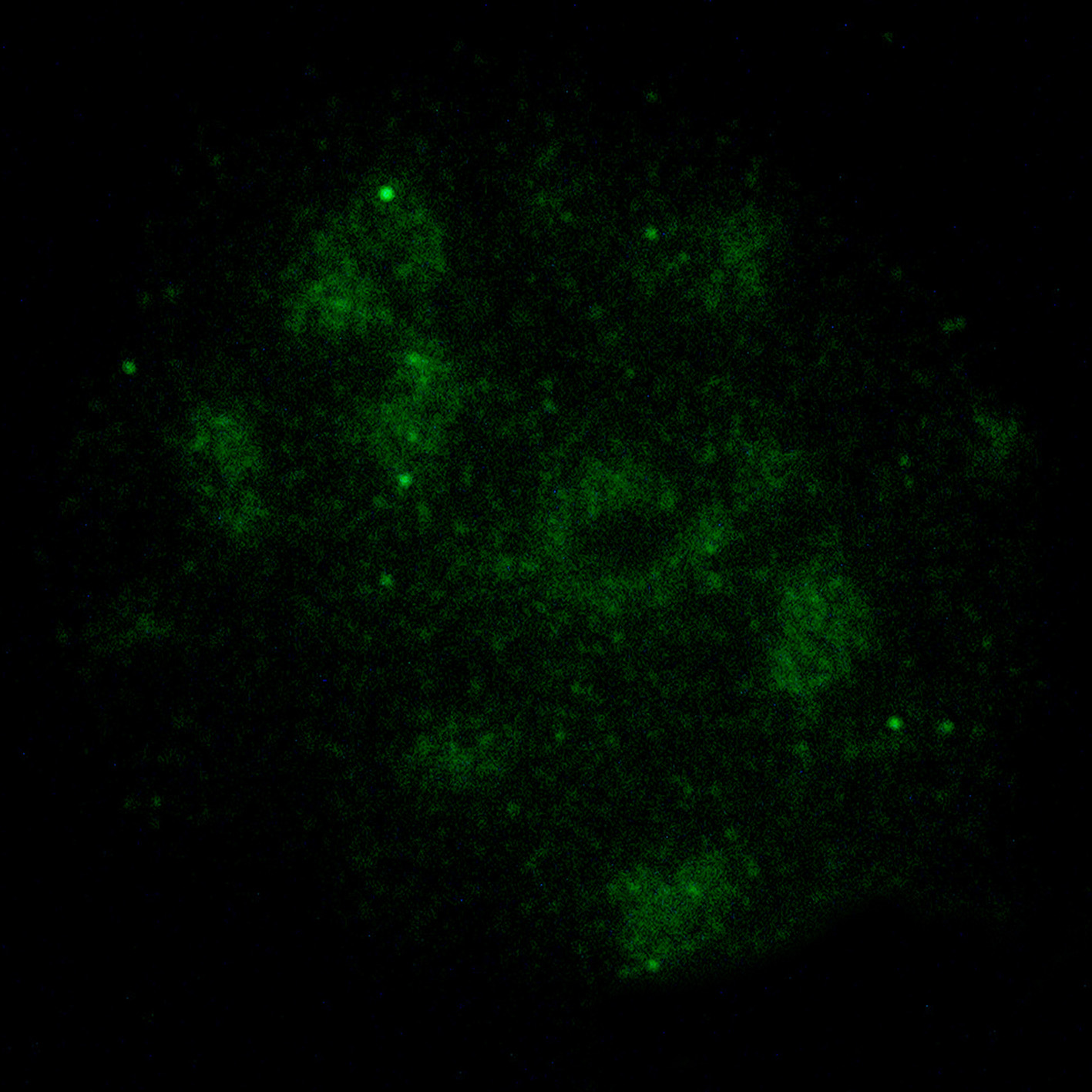

Supplement: Supplementary file 8 — Source data Fig. 1 [file 44318_2024_203_MOESM8_ESM.zip › Figure 1/Figure 1G/Dip-KDM2A.jpg]

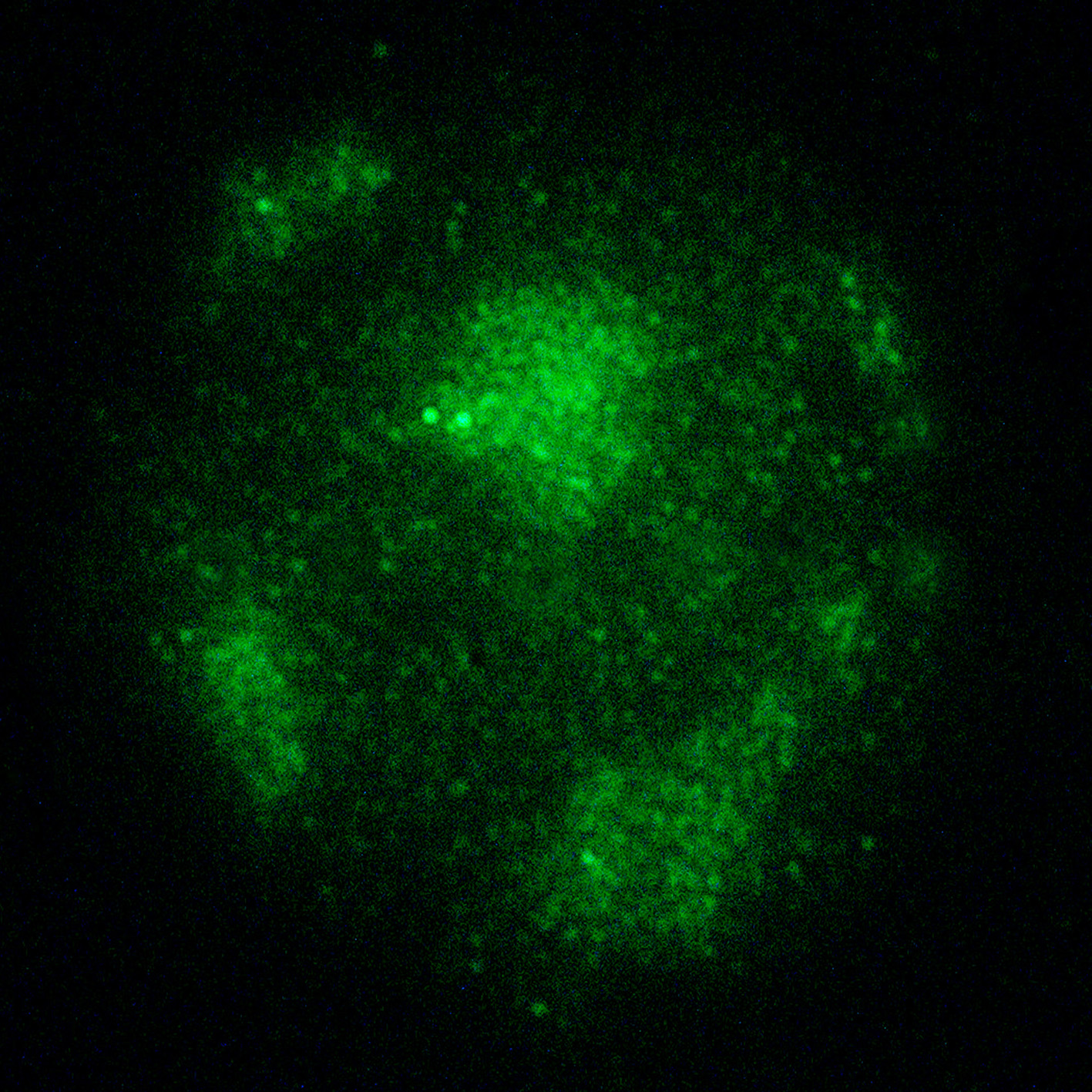

Supplement: Supplementary file 8 — Source data Fig. 1 [file 44318_2024_203_MOESM8_ESM.zip › Figure 1/Figure 1G/Lep-KDM2A.jpg]

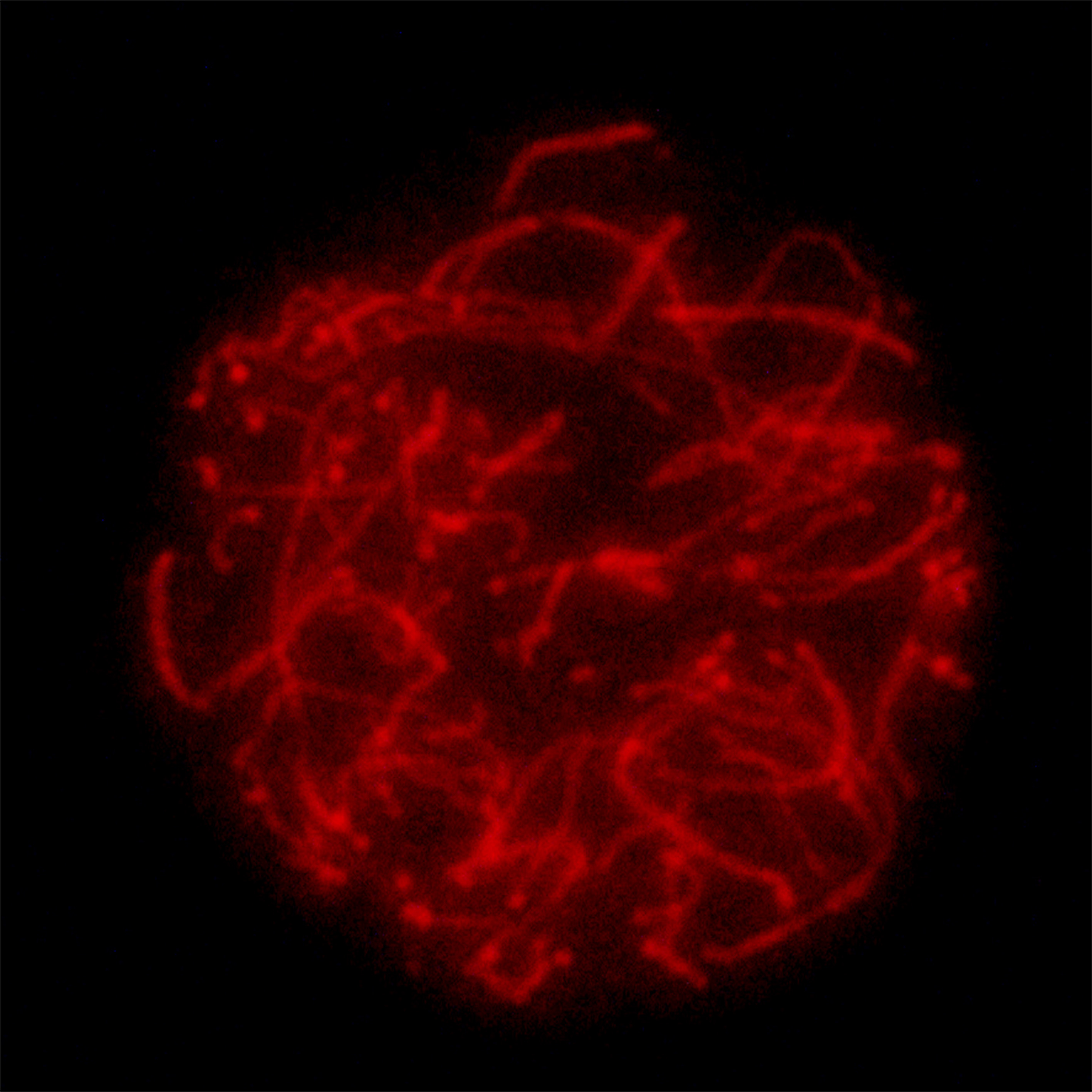

Supplement: Supplementary file 8 — Source data Fig. 1 [file 44318_2024_203_MOESM8_ESM.zip › Figure 1/Figure 1G/Zyg-SYCP3.jpg]

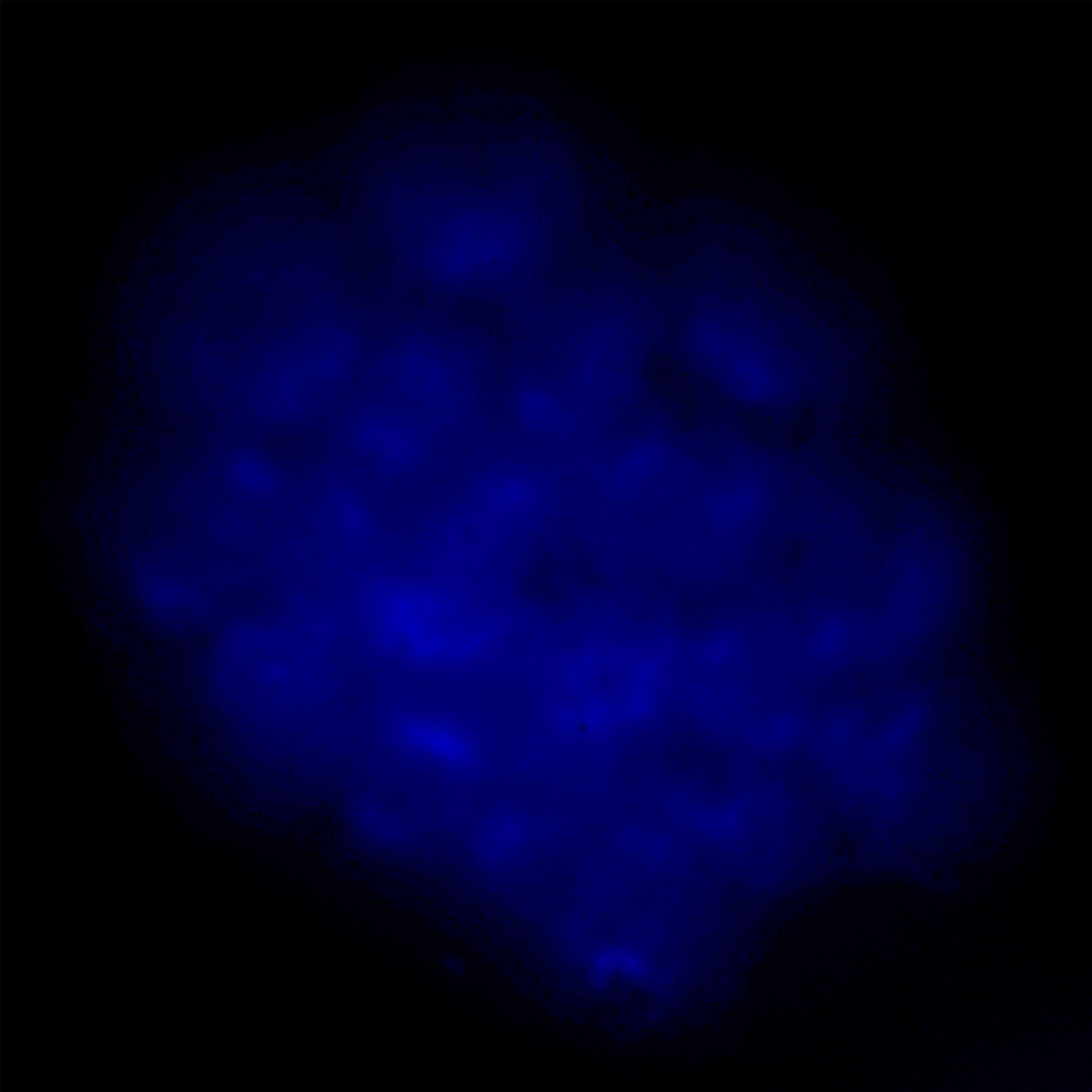

Supplement: Supplementary file 8 — Source data Fig. 1 [file 44318_2024_203_MOESM8_ESM.zip › Figure 1/Figure 1G/Meta-DAPI.jpg]

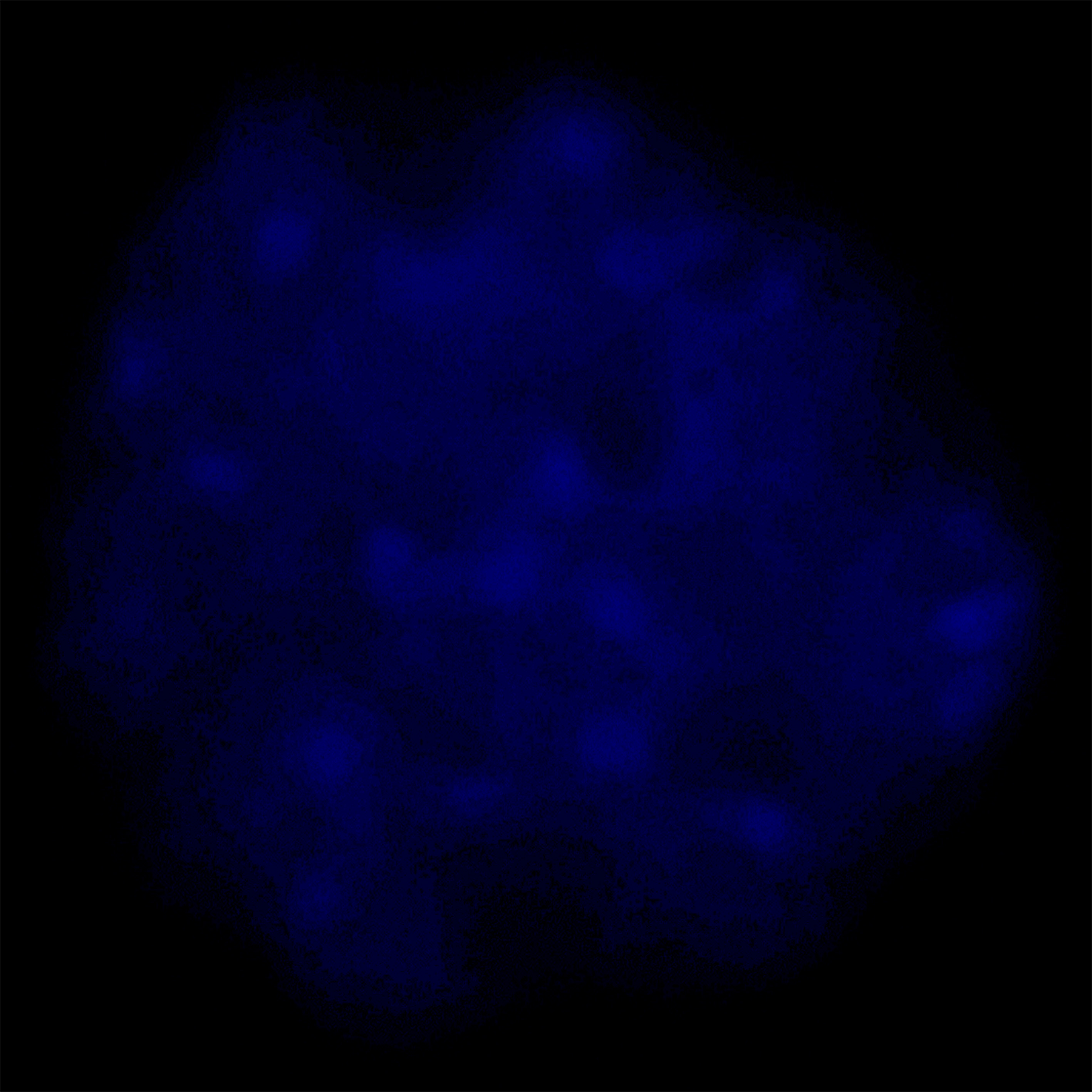

Supplement: Supplementary file 8 — Source data Fig. 1 [file 44318_2024_203_MOESM8_ESM.zip › Figure 1/Figure 1G/Pac-DAPI.jpg]

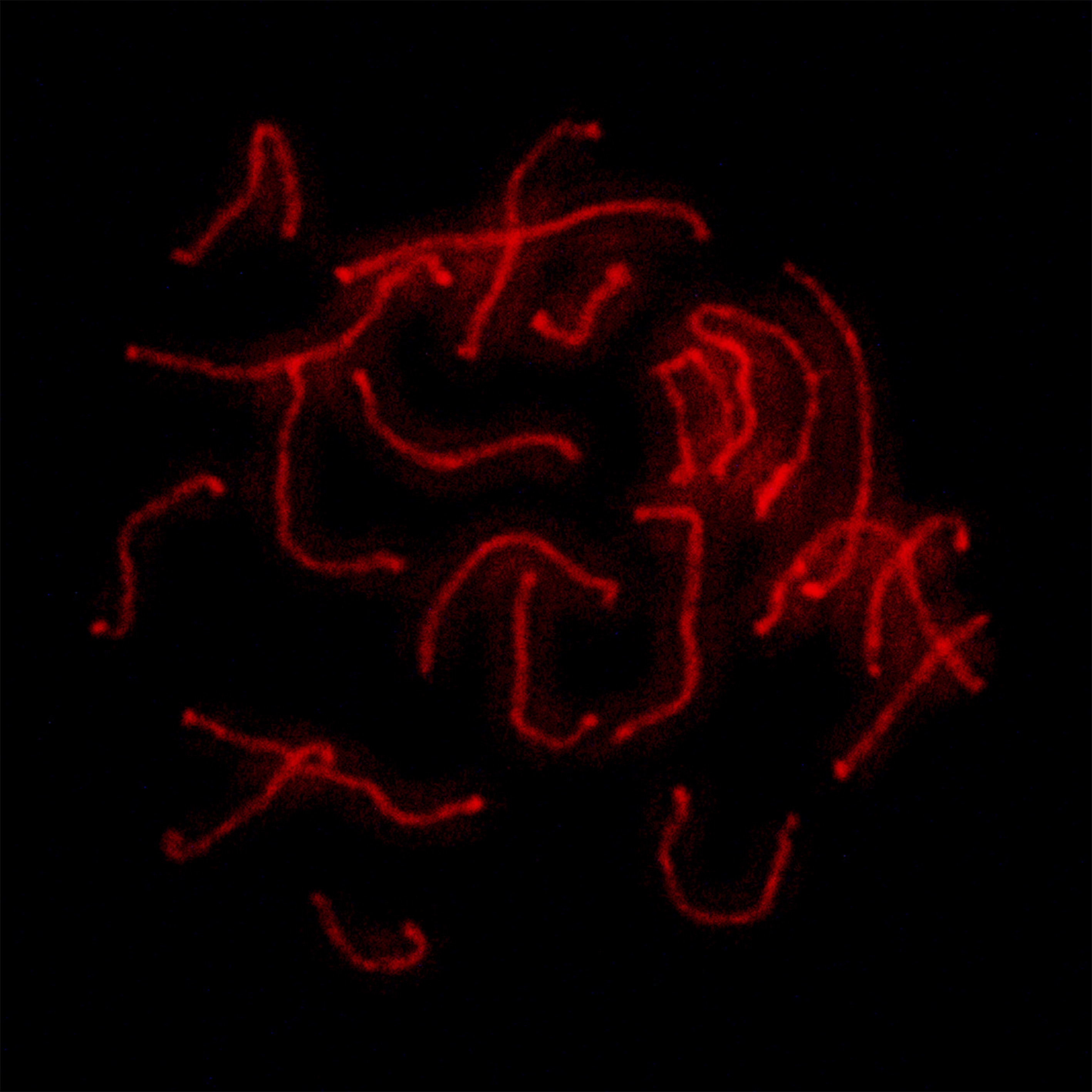

Supplement: Supplementary file 8 — Source data Fig. 1 [file 44318_2024_203_MOESM8_ESM.zip › Figure 1/Figure 1G/Pac-SYCP3.jpg]

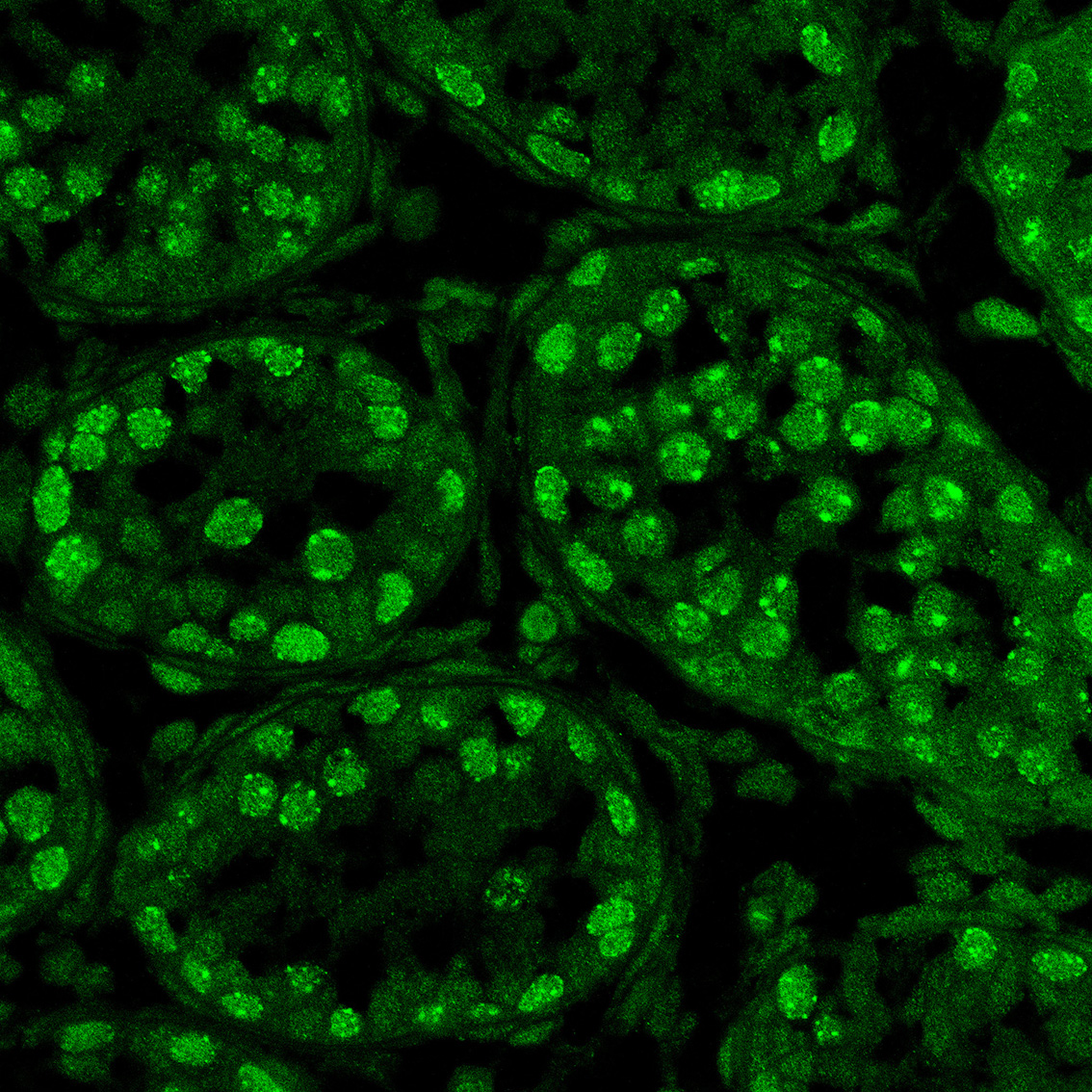

Supplement: Supplementary file 8 — Source data Fig. 1 [file 44318_2024_203_MOESM8_ESM.zip › Figure 1/Figure 1E/KDM2A.jpg]

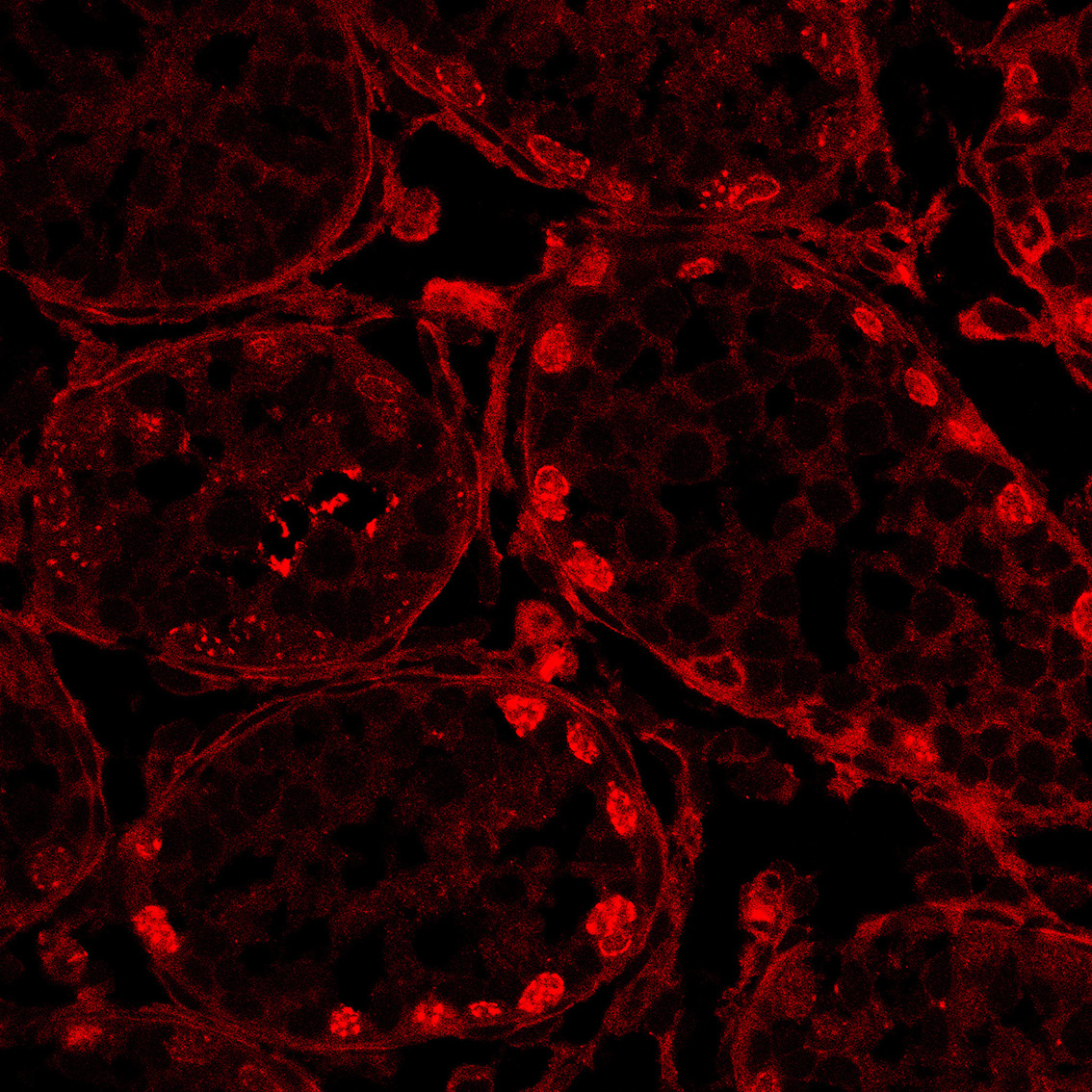

Supplement: Supplementary file 8 — Source data Fig. 1 [file 44318_2024_203_MOESM8_ESM.zip › Figure 1/Figure 1E/Sall4.jpg]

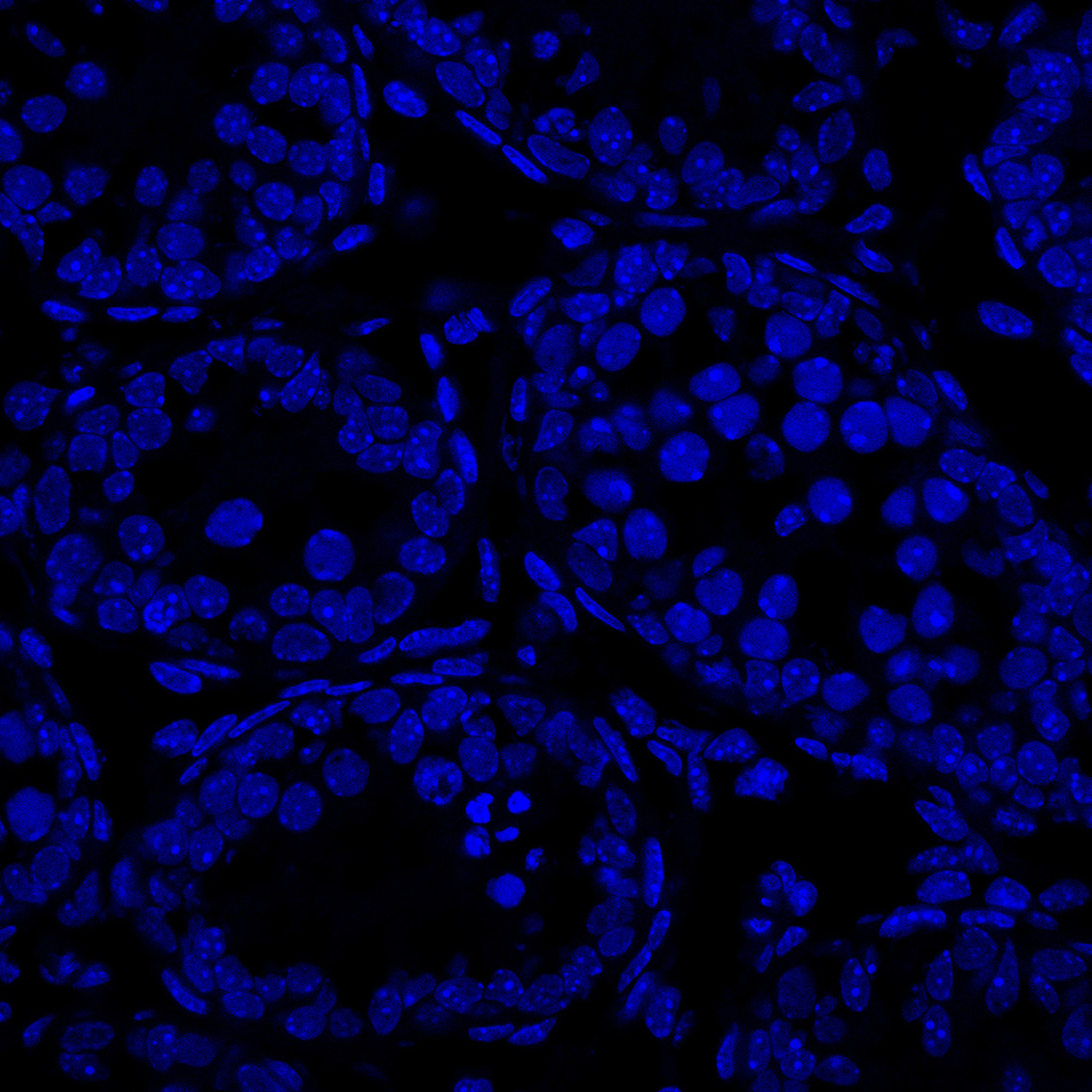

Supplement: Supplementary file 8 — Source data Fig. 1 [file 44318_2024_203_MOESM8_ESM.zip › Figure 1/Figure 1E/DAPI.jpg]

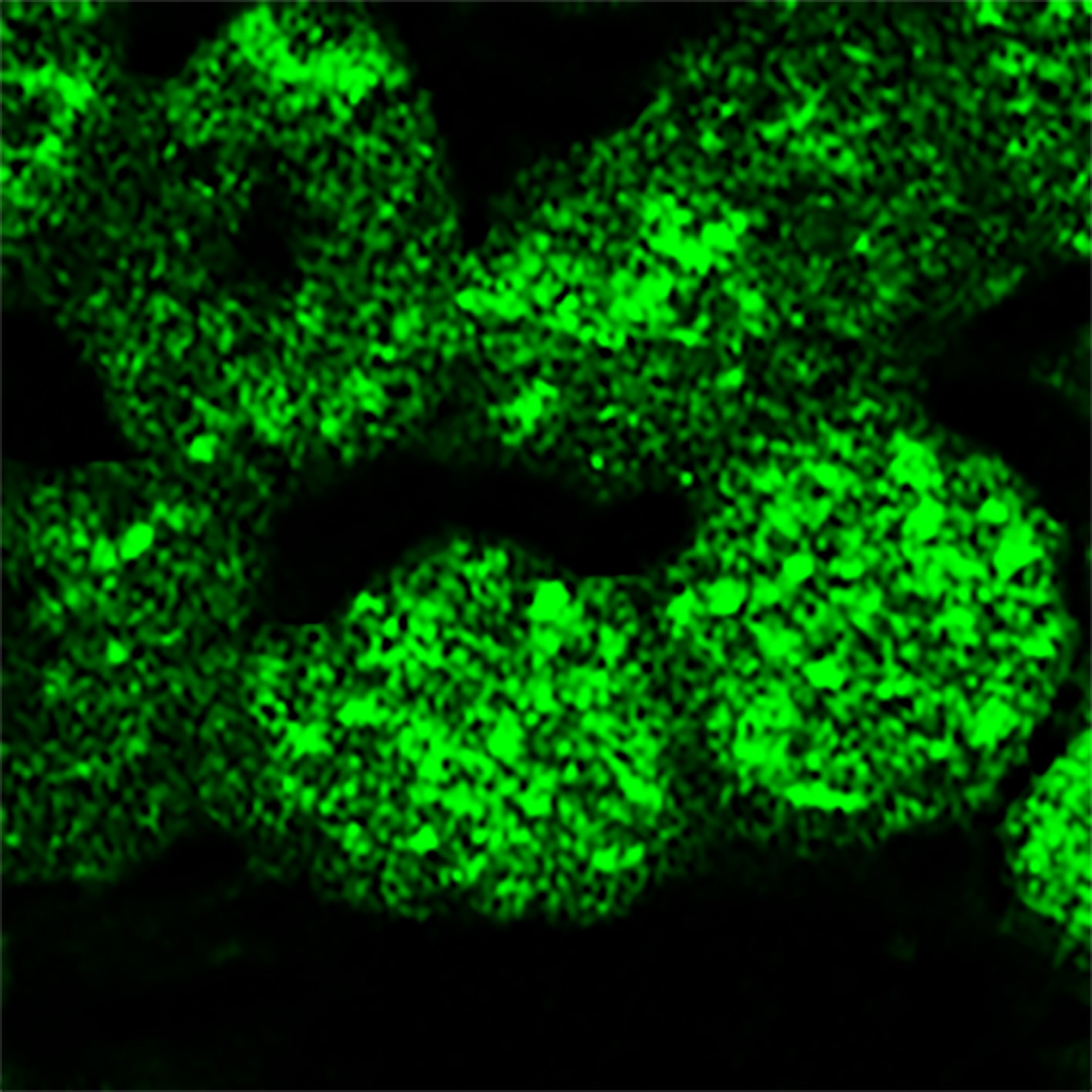

Supplement: Supplementary file 8 — Source data Fig. 1 [file 44318_2024_203_MOESM8_ESM.zip › Figure 1/Figure 1D/Leptene-KDM2A.jpg]

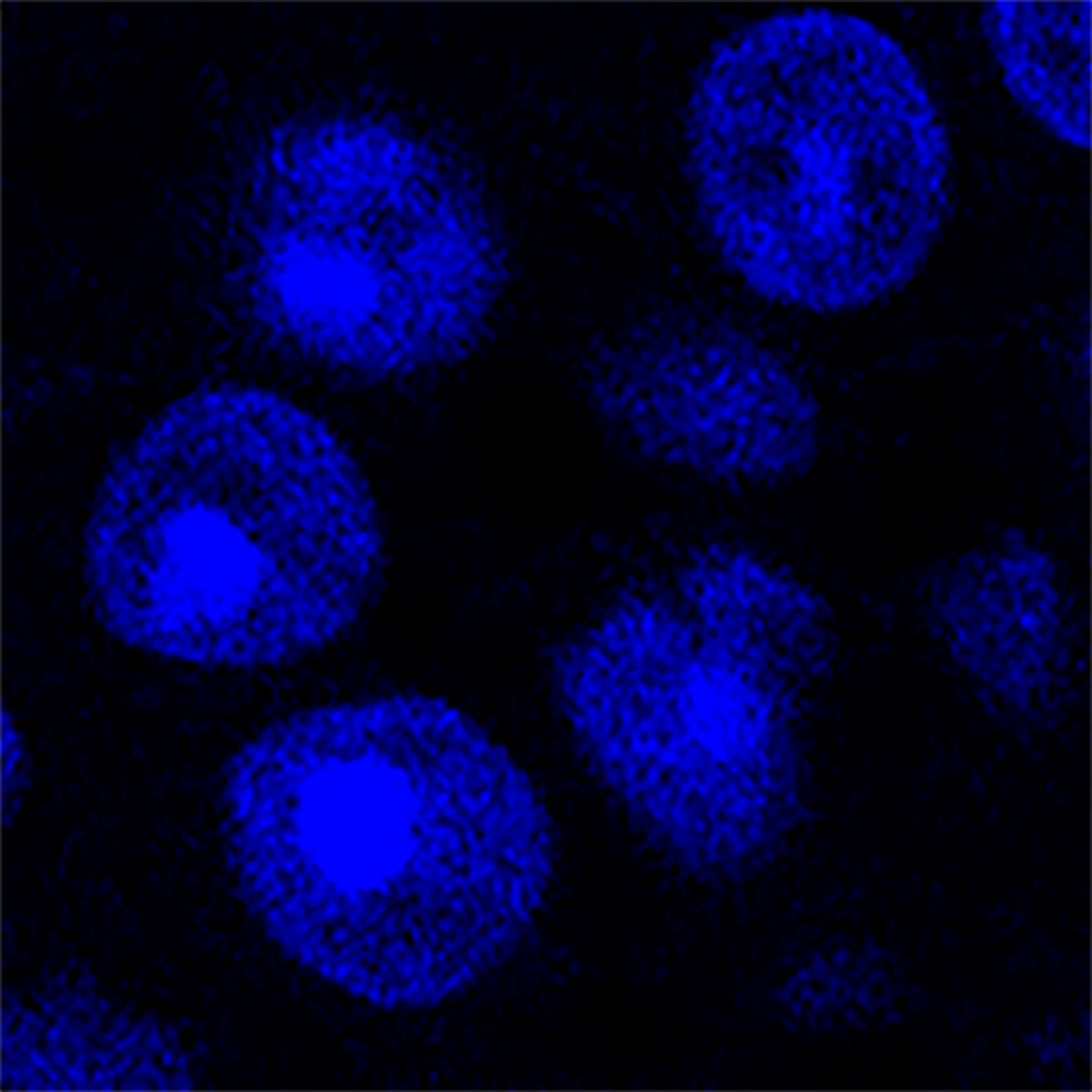

Supplement: Supplementary file 8 — Source data Fig. 1 [file 44318_2024_203_MOESM8_ESM.zip › Figure 1/Figure 1D/Elongating Spermatid (Sp.7-8)- DAPI.jpg]

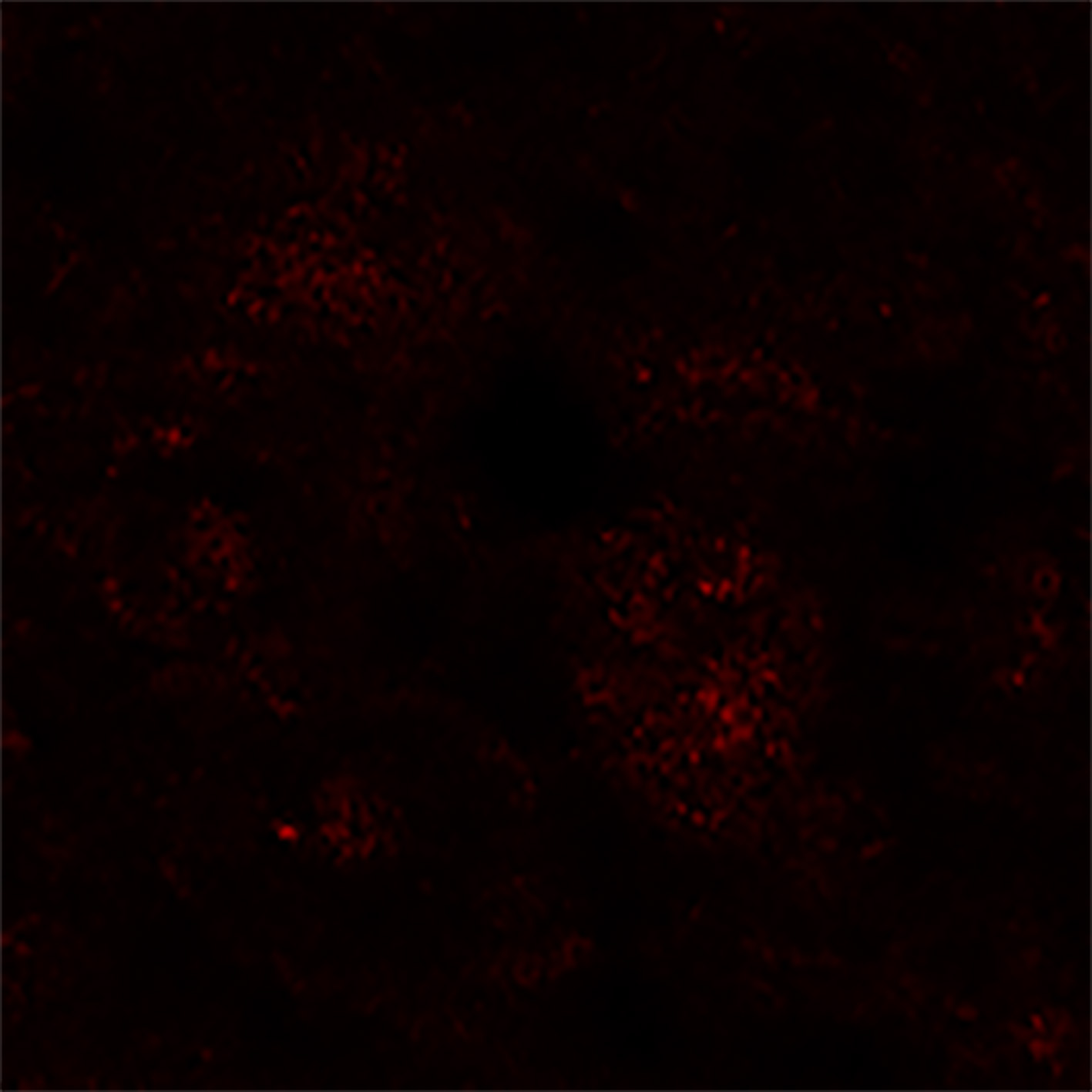

Supplement: Supplementary file 8 — Source data Fig. 1 [file 44318_2024_203_MOESM8_ESM.zip › Figure 1/Figure 1D/Elongating Spermatid (Sp.7-8) - ╬│H2AX.jpg]

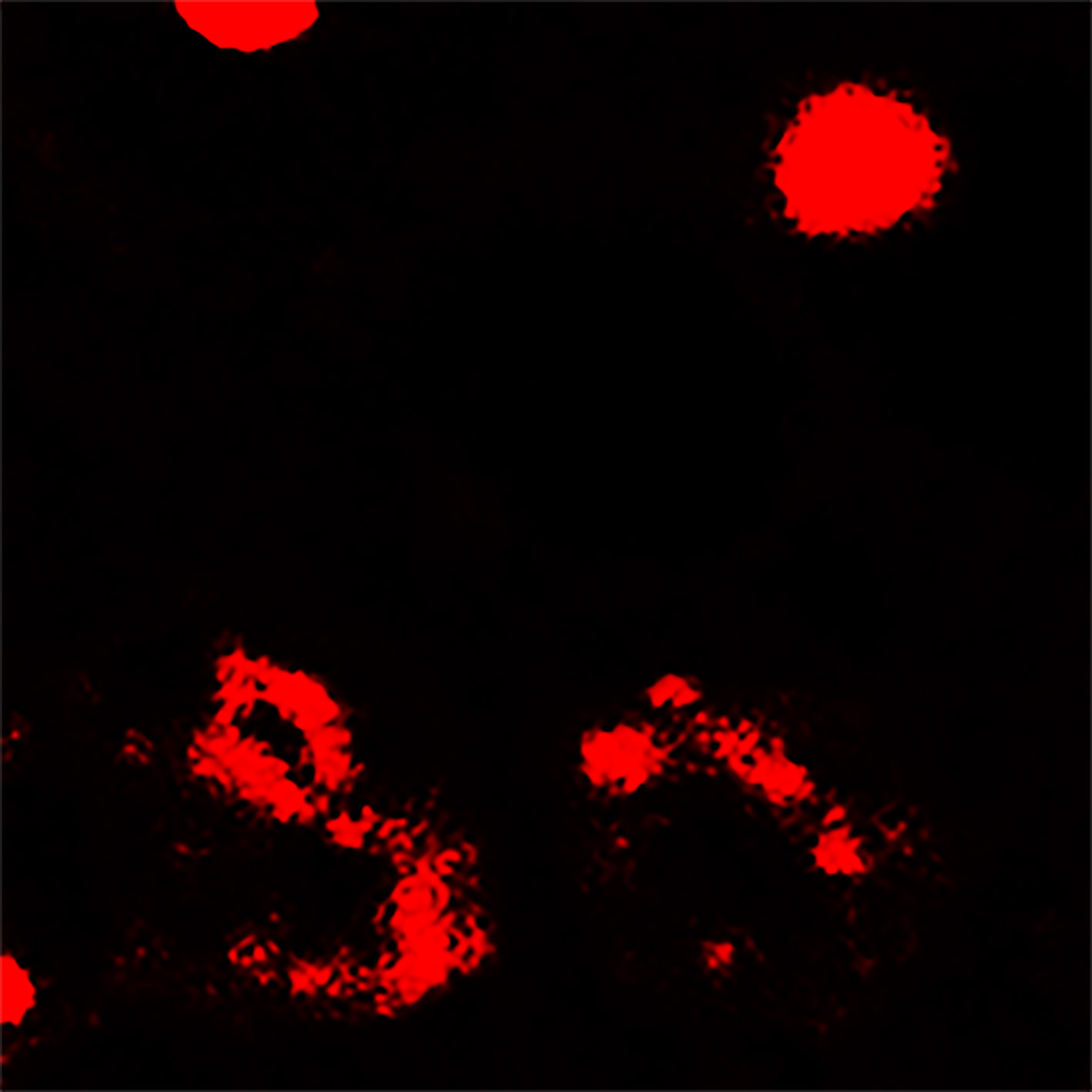

Supplement: Supplementary file 8 — Source data Fig. 1 [file 44318_2024_203_MOESM8_ESM.zip › Figure 1/Figure 1D/Zagotene- ╬│H2AX.jpg]

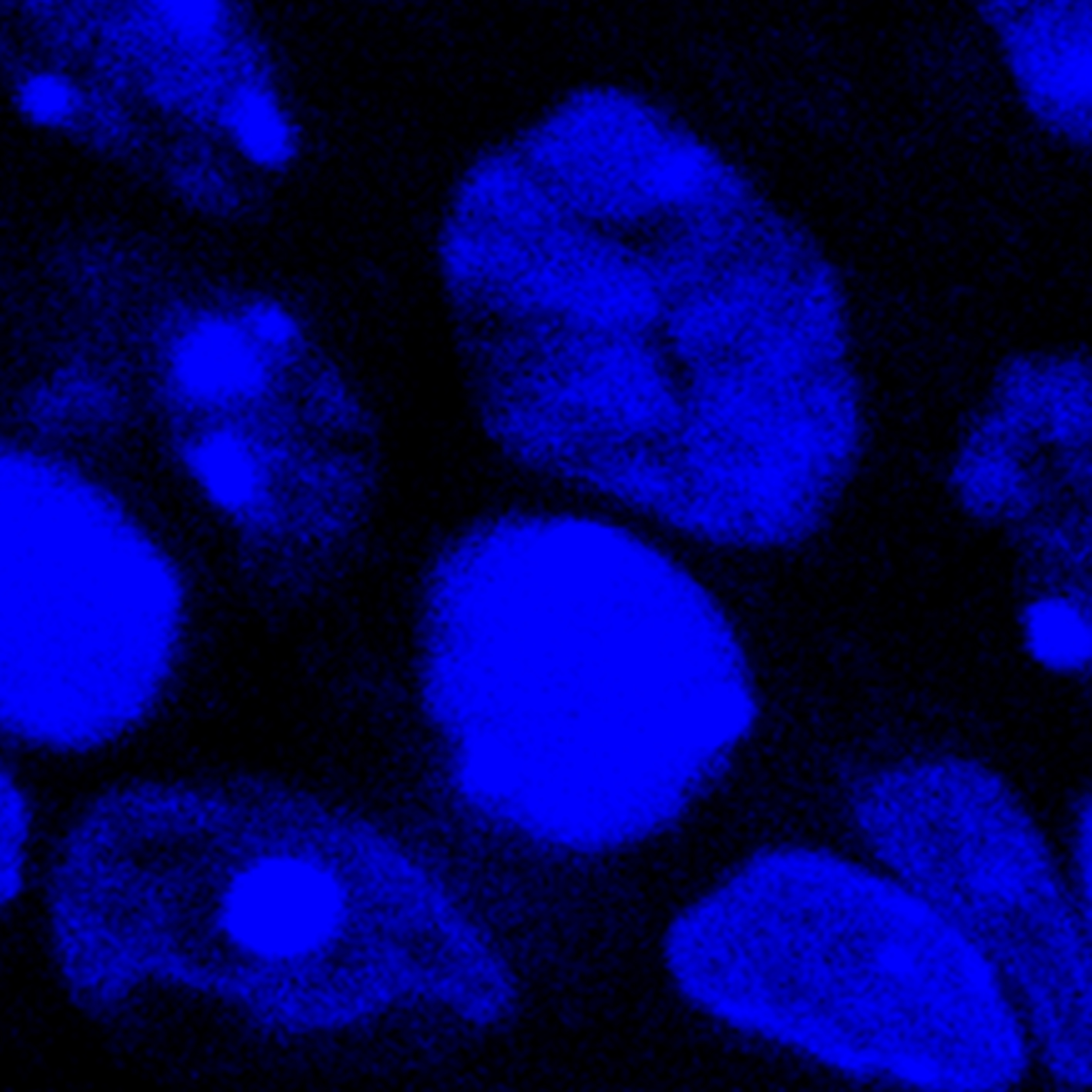

Supplement: Supplementary file 8 — Source data Fig. 1 [file 44318_2024_203_MOESM8_ESM.zip › Figure 1/Figure 1D/Diplotene- DAPI.jpg]

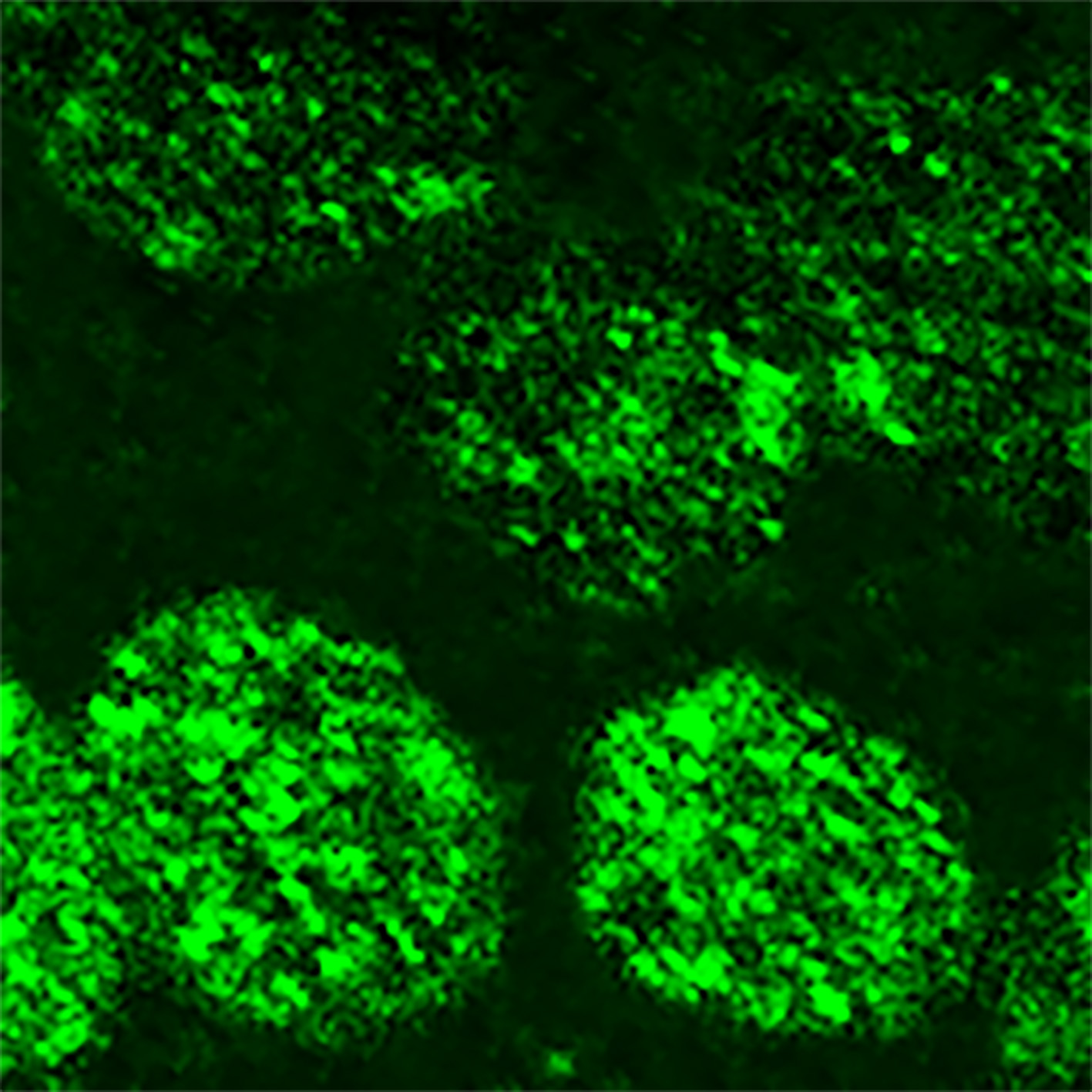

Supplement: Supplementary file 8 — Source data Fig. 1 [file 44318_2024_203_MOESM8_ESM.zip › Figure 1/Figure 1D/Zagotene- KDM2A.jpg]

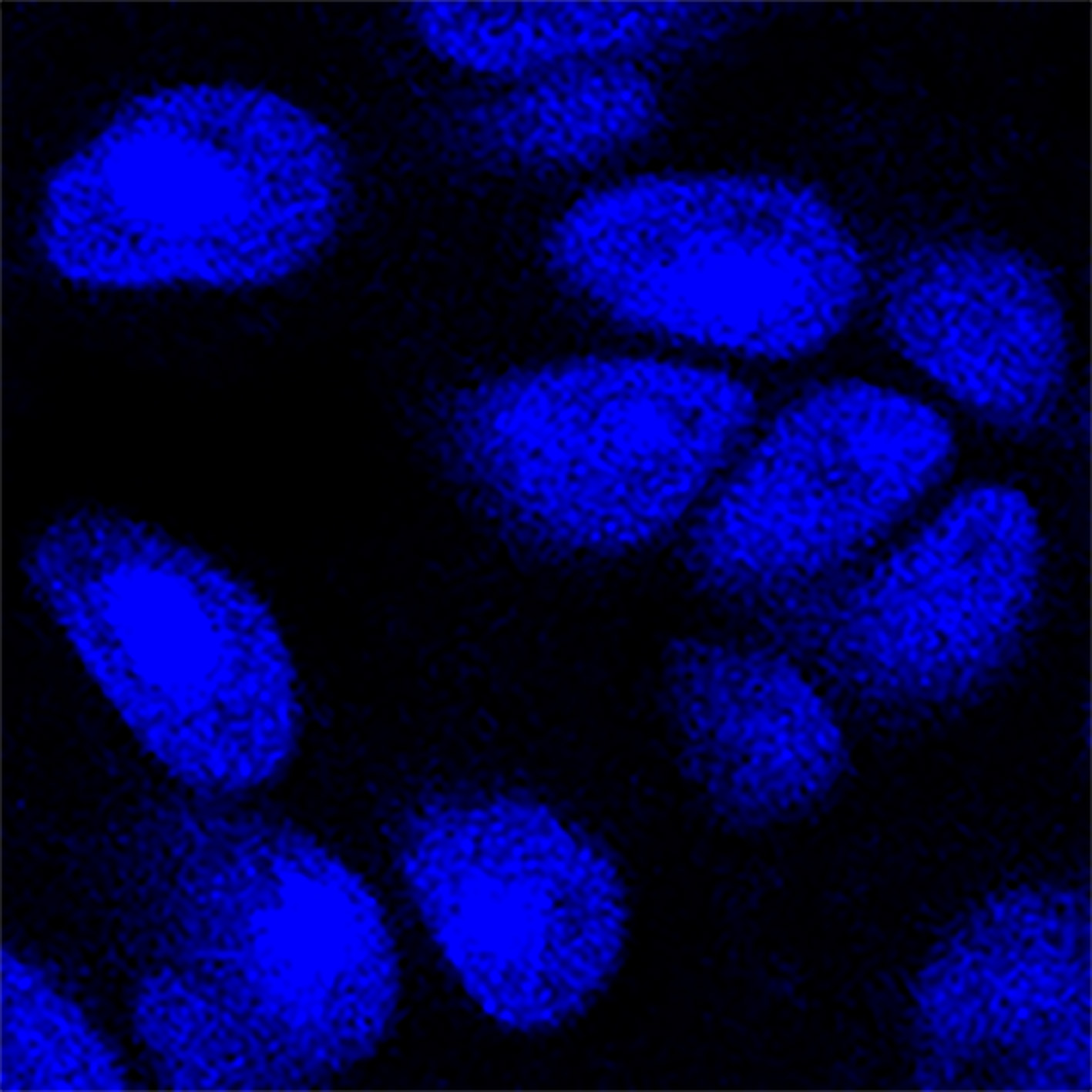

Supplement: Supplementary file 8 — Source data Fig. 1 [file 44318_2024_203_MOESM8_ESM.zip › Figure 1/Figure 1D/Elongating Spermatid (Sp.9-10)- DAPI.jpg]

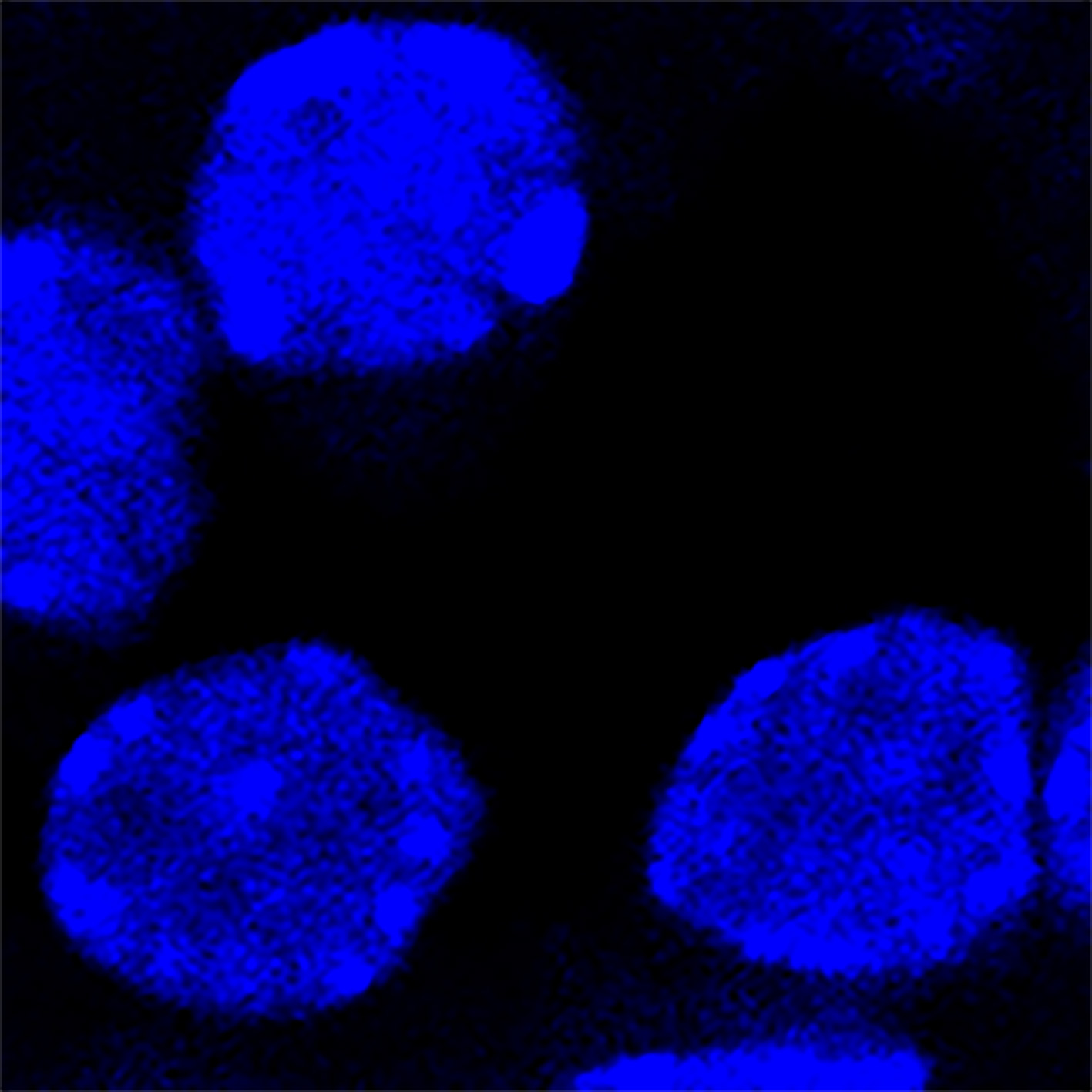

Supplement: Supplementary file 8 — Source data Fig. 1 [file 44318_2024_203_MOESM8_ESM.zip › Figure 1/Figure 1D/Spg- DAPI.jpg]

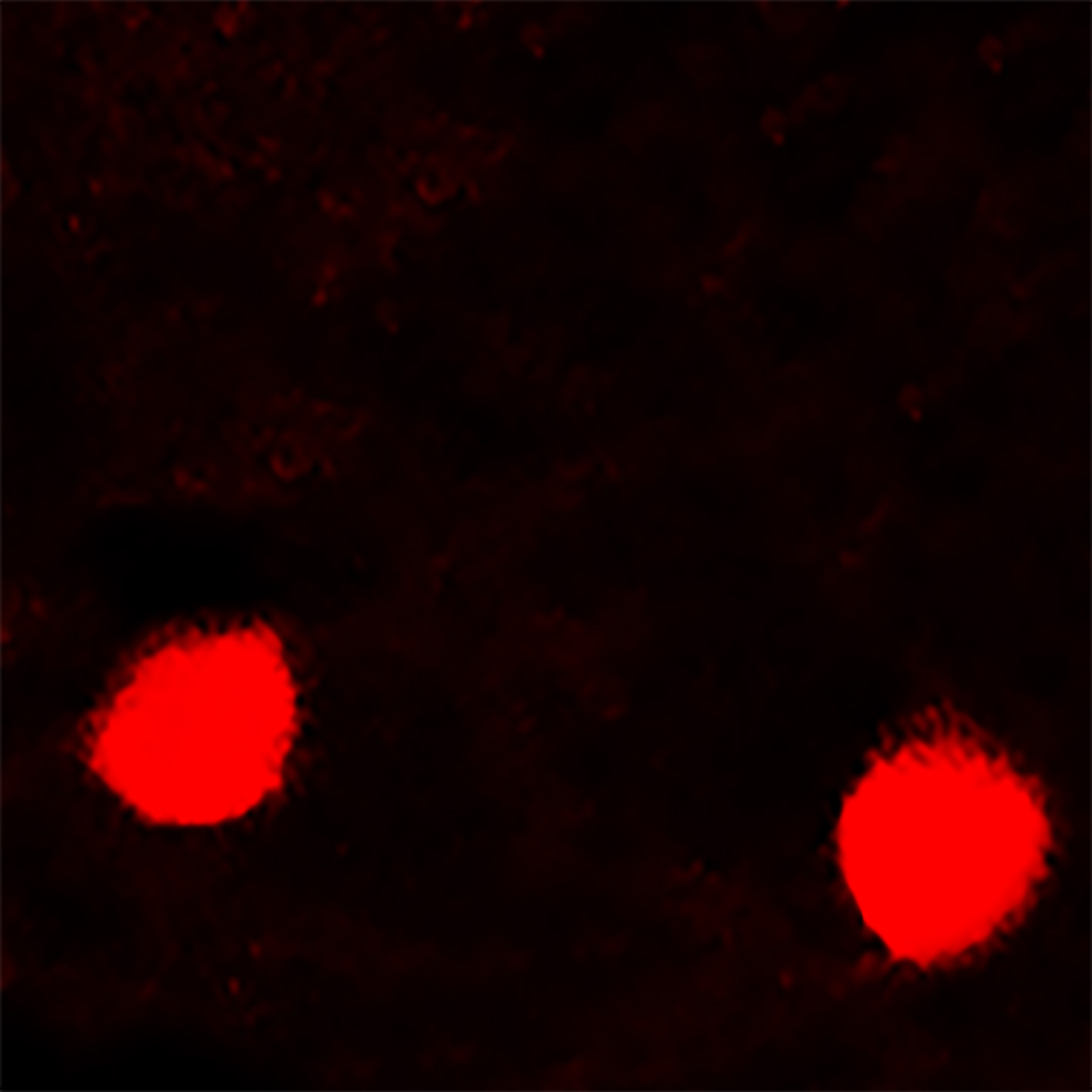

Supplement: Supplementary file 8 — Source data Fig. 1 [file 44318_2024_203_MOESM8_ESM.zip › Figure 1/Figure 1D/Pachytene (St. IV-VI) - ╬│H2AX.jpg]

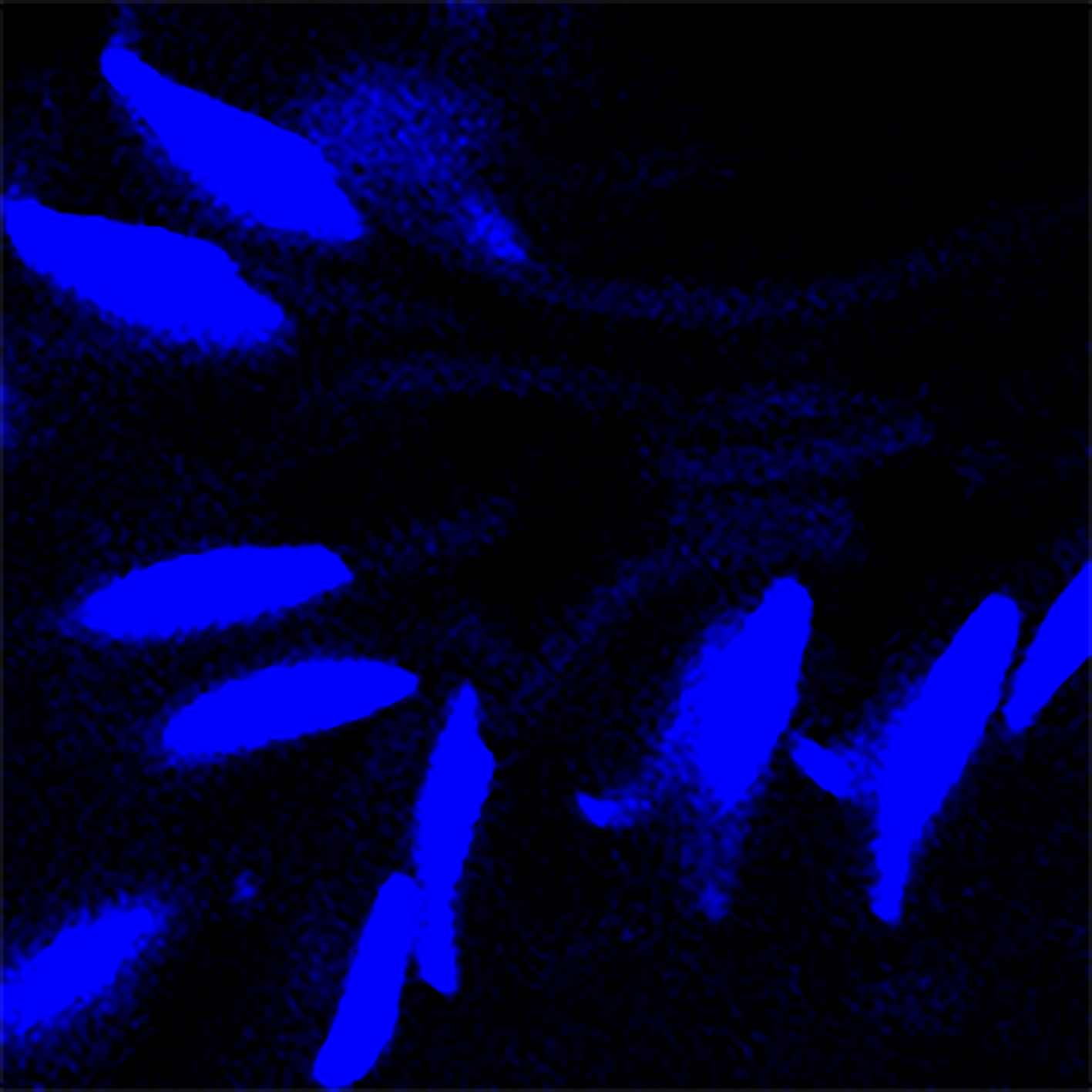

Supplement: Supplementary file 8 — Source data Fig. 1 [file 44318_2024_203_MOESM8_ESM.zip › Figure 1/Figure 1D/Elongated Spermatid (Sp.14-16) - DAPI.jpg]

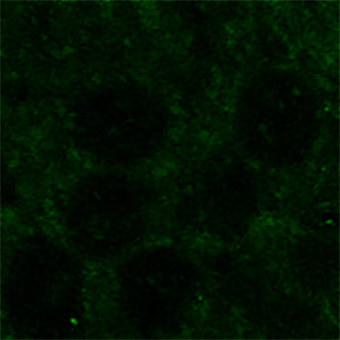

Supplement: Supplementary file 8 — Source data Fig. 1 [file 44318_2024_203_MOESM8_ESM.zip › Figure 1/Figure 1D/Round Spermatid (Sp.5-6)-KDM2A.jpg]

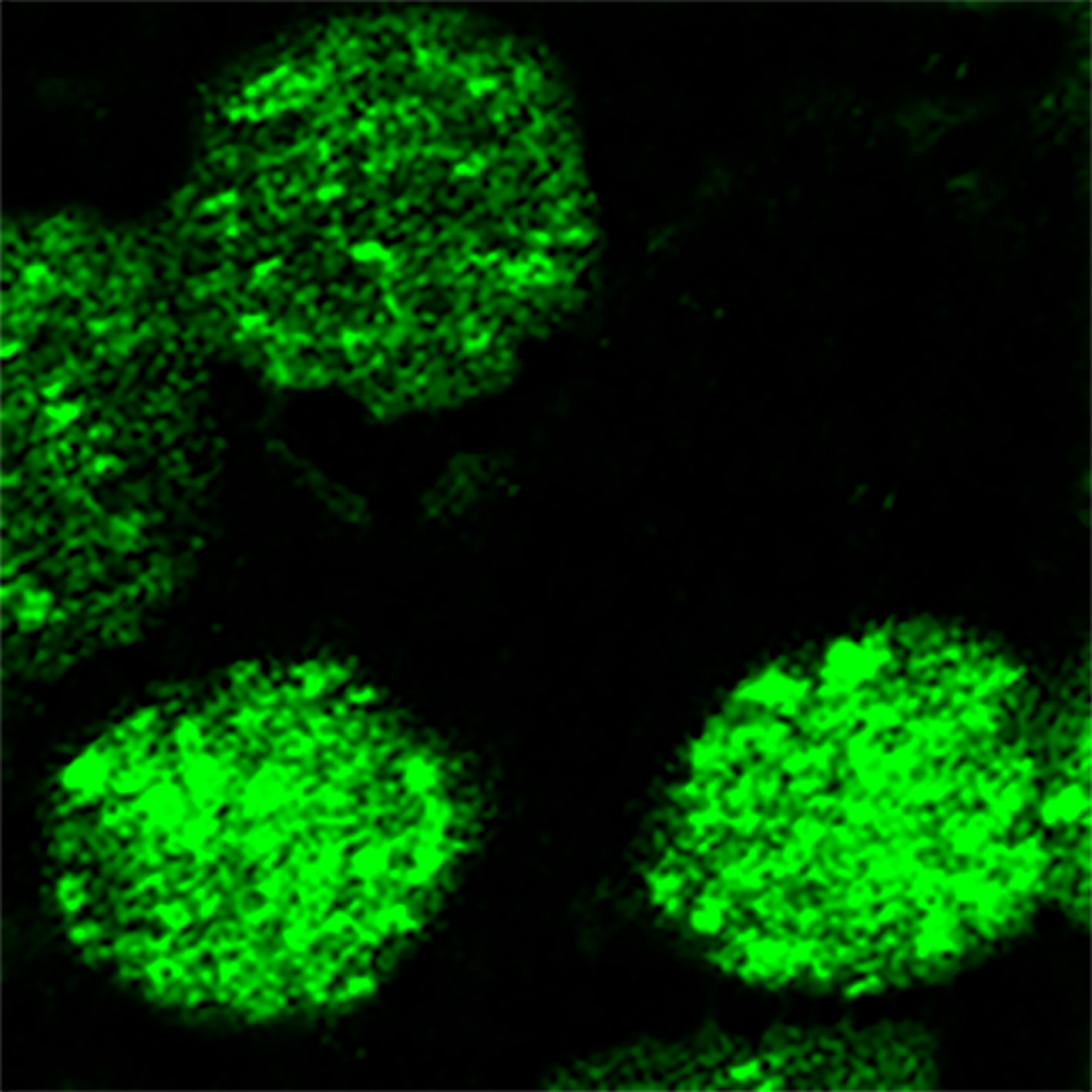

Supplement: Supplementary file 8 — Source data Fig. 1 [file 44318_2024_203_MOESM8_ESM.zip › Figure 1/Figure 1D/Spg- KDM2A.jpg]

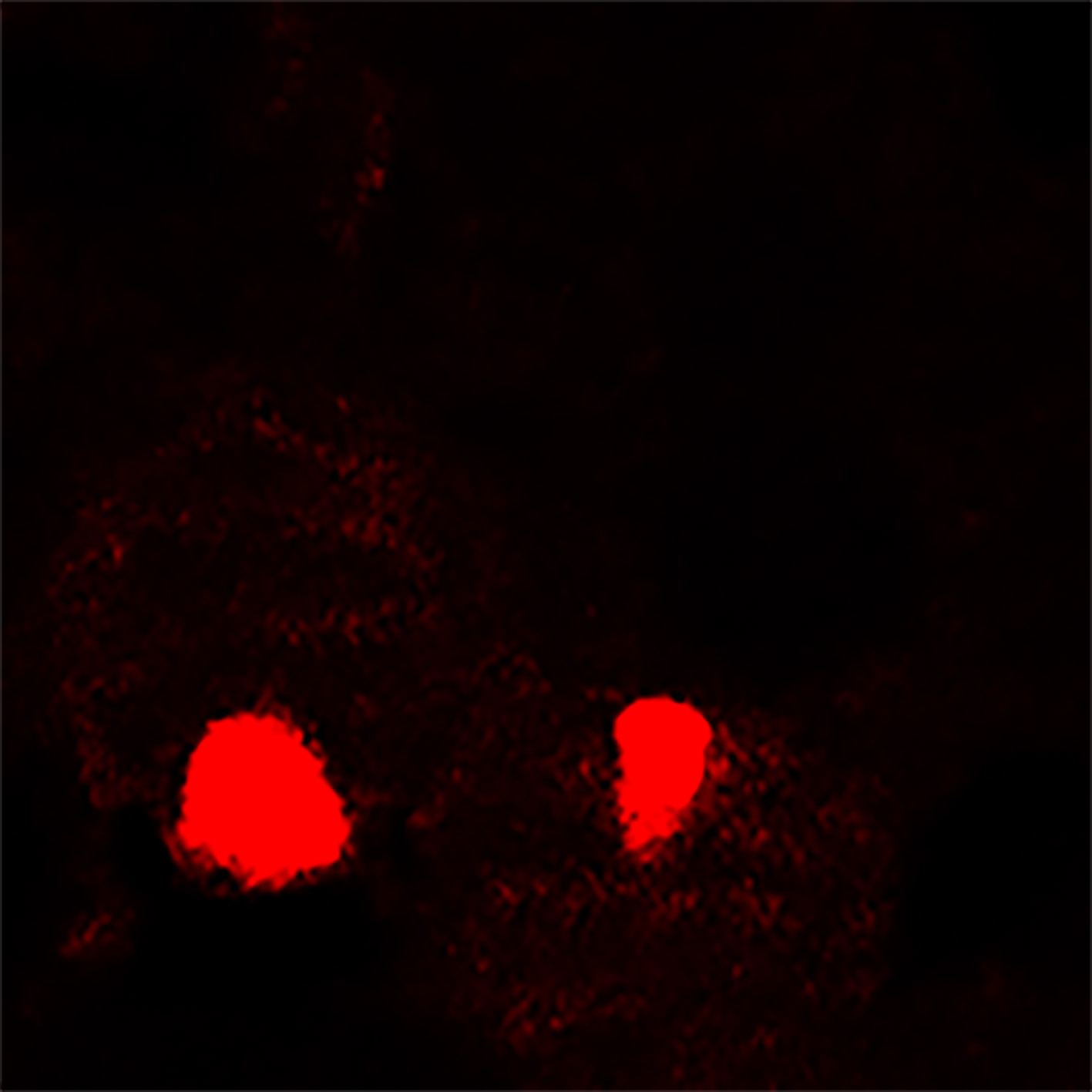

Supplement: Supplementary file 8 — Source data Fig. 1 [file 44318_2024_203_MOESM8_ESM.zip › Figure 1/Figure 1D/Pachytene (St. I-III) - ╬│H2AX.jpg]

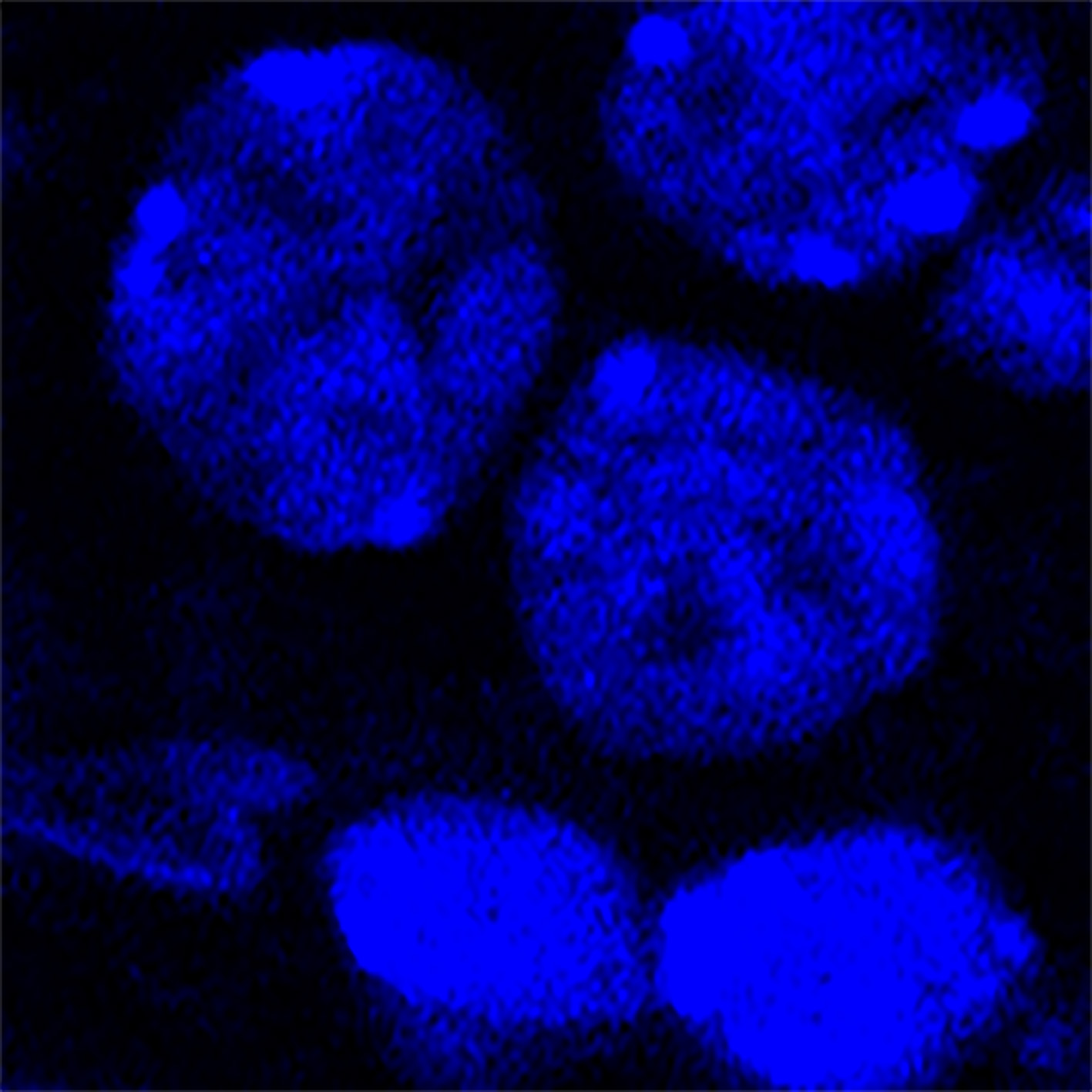

Supplement: Supplementary file 8 — Source data Fig. 1 [file 44318_2024_203_MOESM8_ESM.zip › Figure 1/Figure 1D/Pachytene (St. IX-X)- DAPI.jpg]

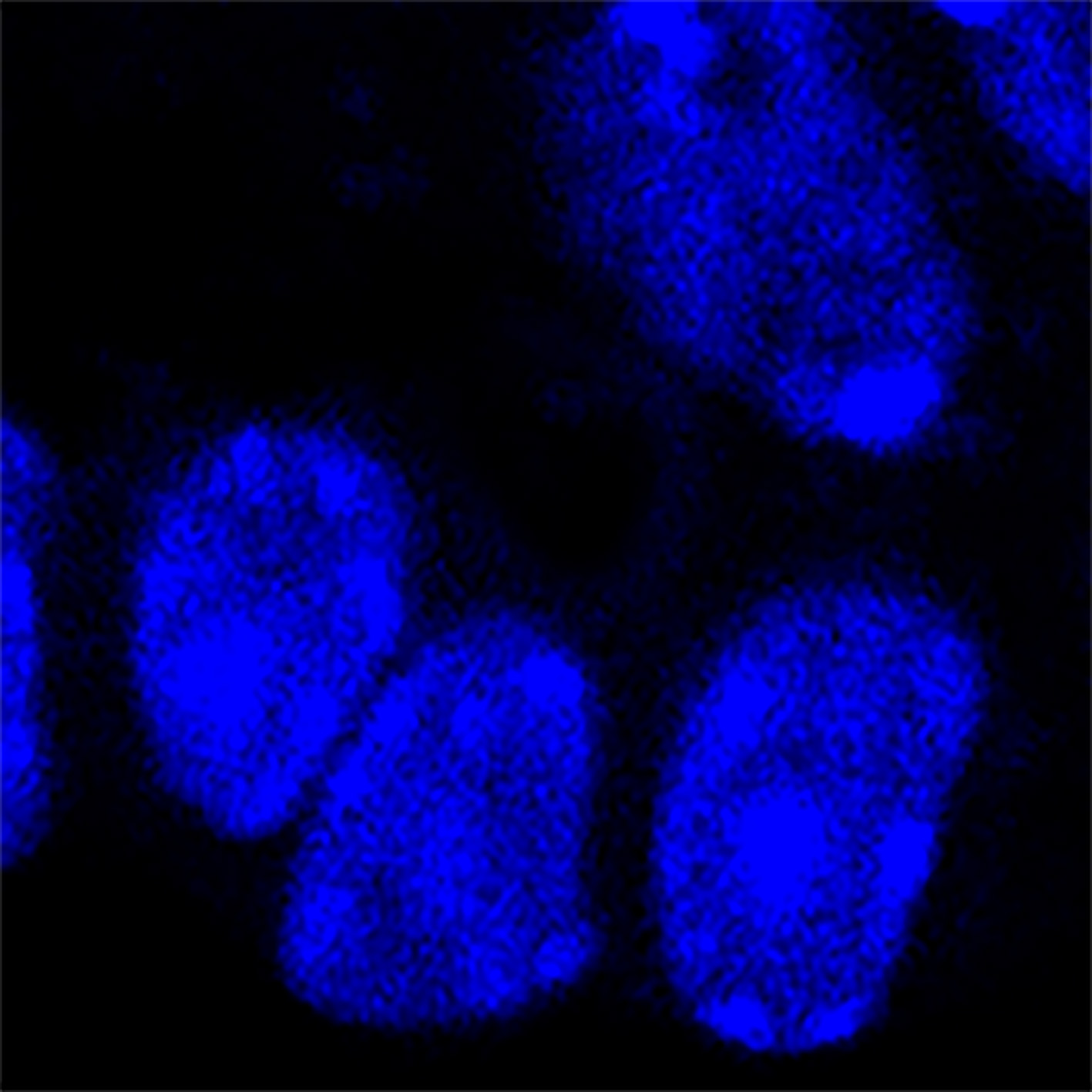

Supplement: Supplementary file 8 — Source data Fig. 1 [file 44318_2024_203_MOESM8_ESM.zip › Figure 1/Figure 1D/Pre-leptotene - DAPI.jpg]

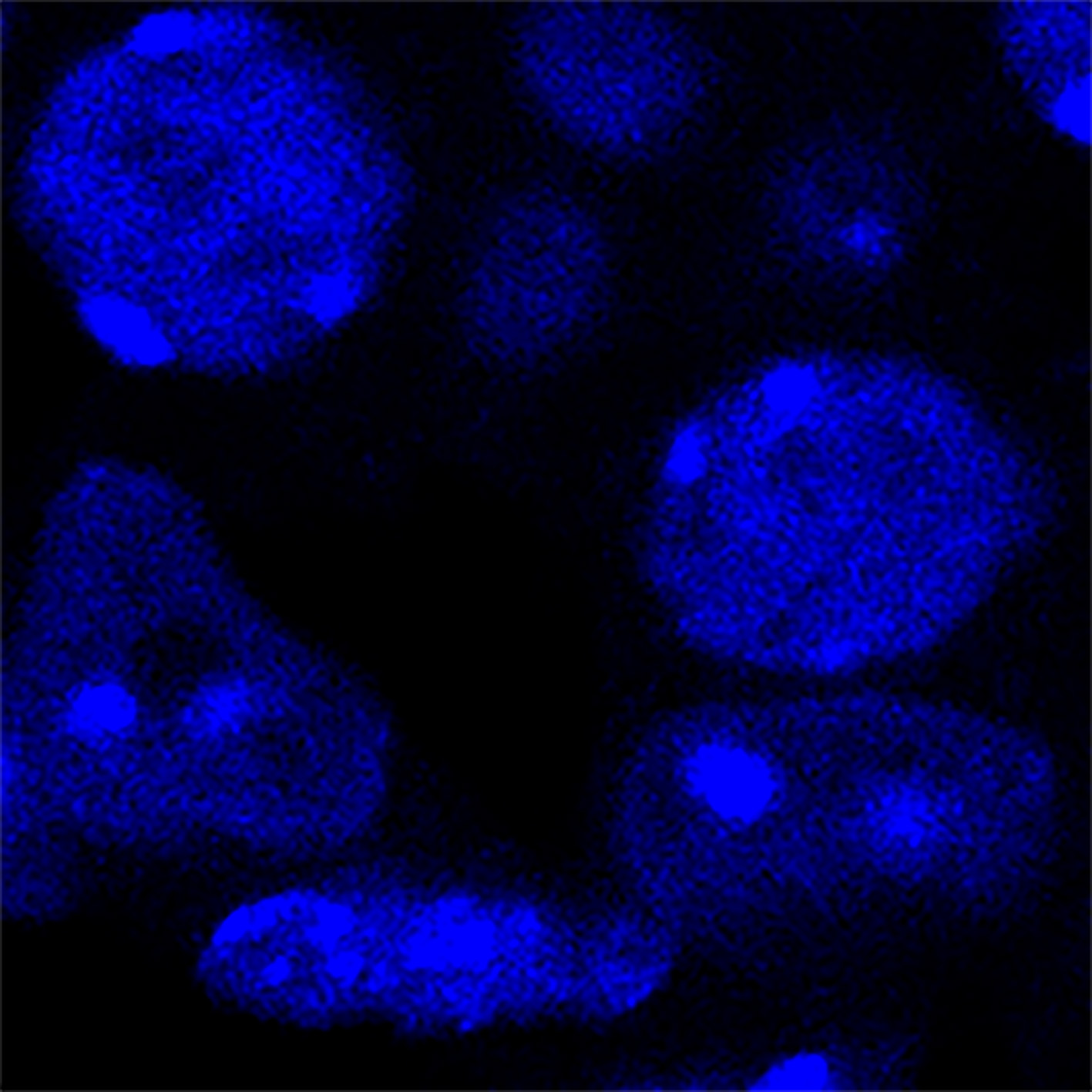

Supplement: Supplementary file 8 — Source data Fig. 1 [file 44318_2024_203_MOESM8_ESM.zip › Figure 1/Figure 1D/Sertoli cell- DAPI.jpg]

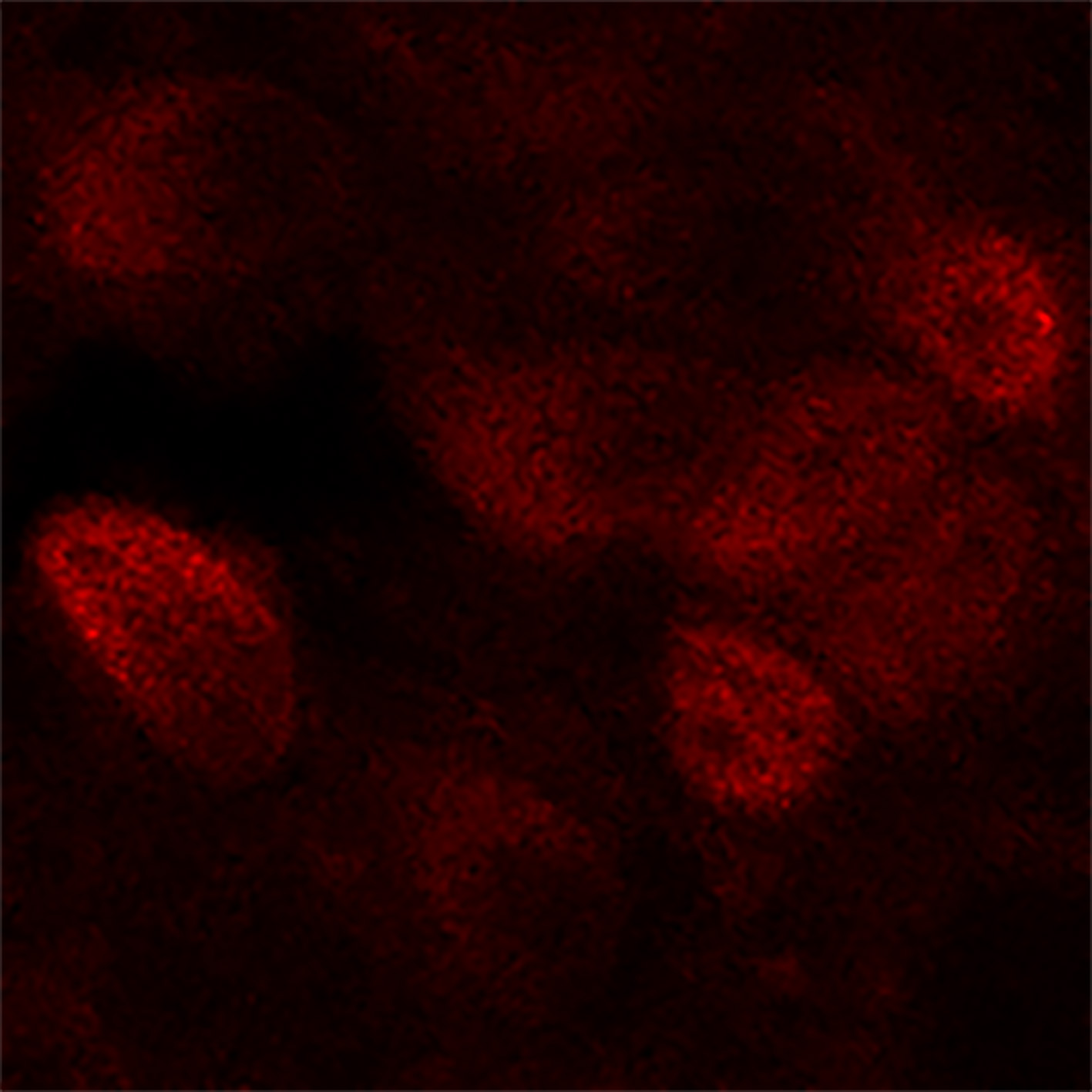

Supplement: Supplementary file 8 — Source data Fig. 1 [file 44318_2024_203_MOESM8_ESM.zip › Figure 1/Figure 1D/Elongating Spermatid (Sp.9-10) - ╬│H2AX.jpg]

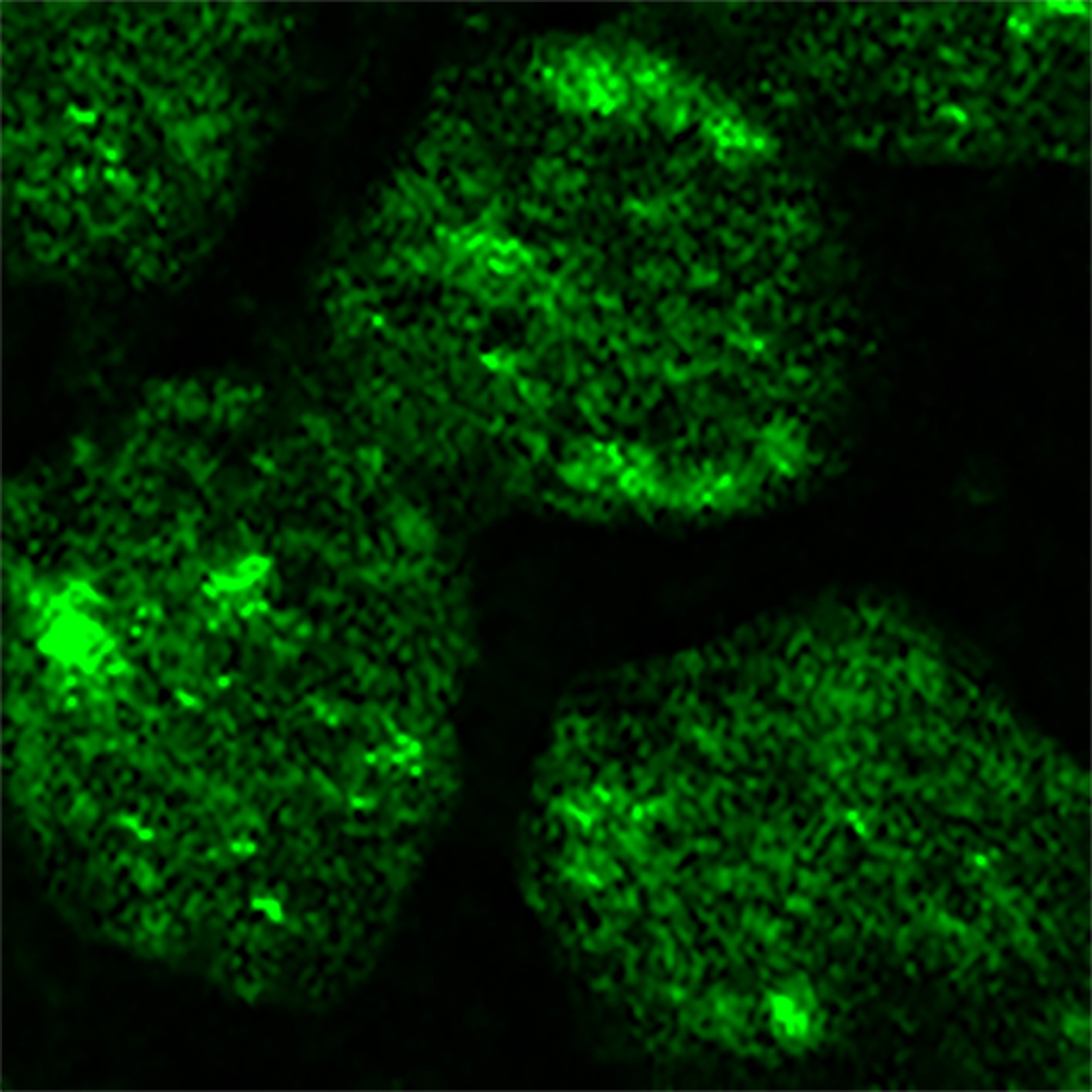

Supplement: Supplementary file 8 — Source data Fig. 1 [file 44318_2024_203_MOESM8_ESM.zip › Figure 1/Figure 1D/Pachytene (St. VII-VIII)-KDM2A.jpg]

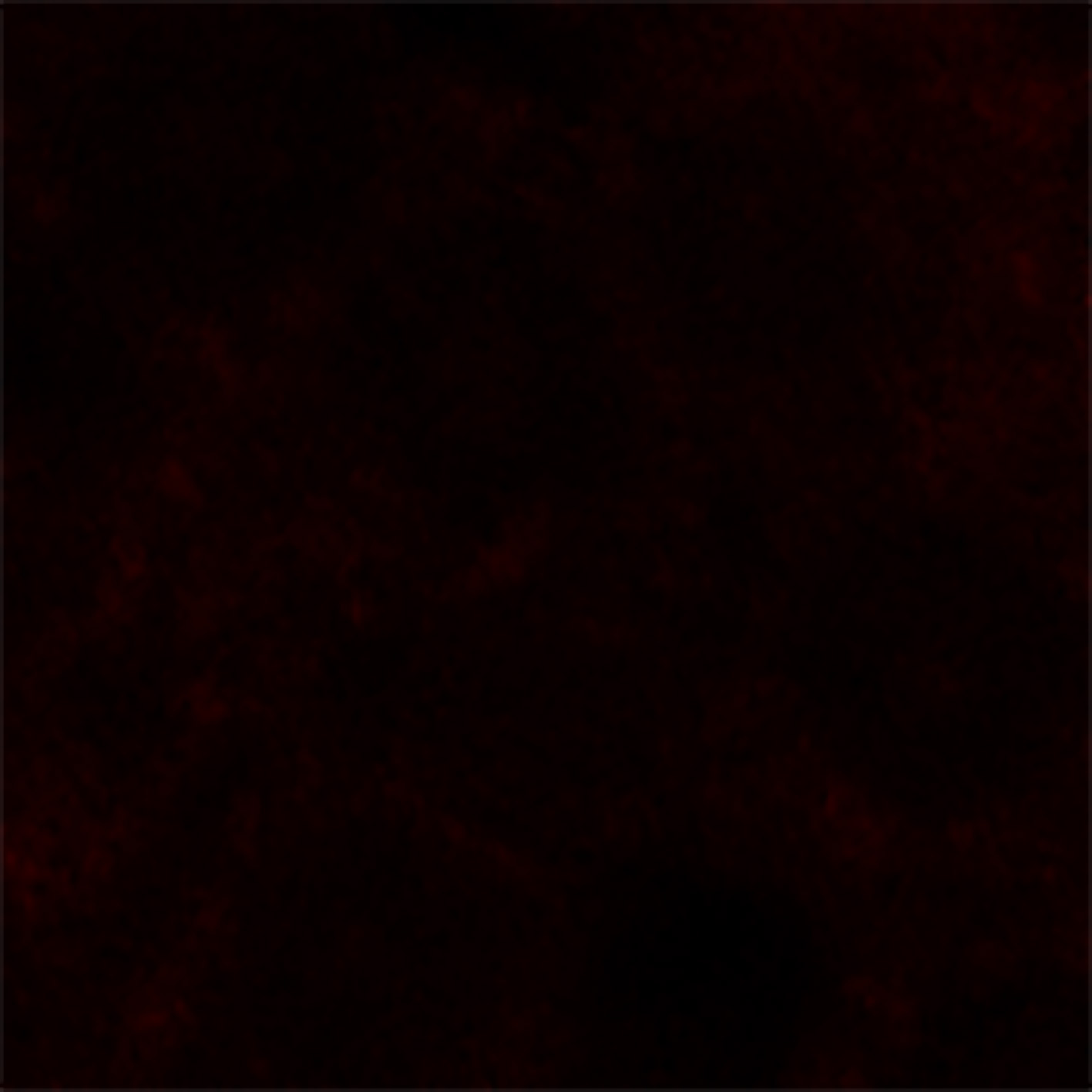

Supplement: Supplementary file 8 — Source data Fig. 1 [file 44318_2024_203_MOESM8_ESM.zip › Figure 1/Figure 1D/Round Spermatid (Sp.2-4) - ╬│H2AX.jpg]

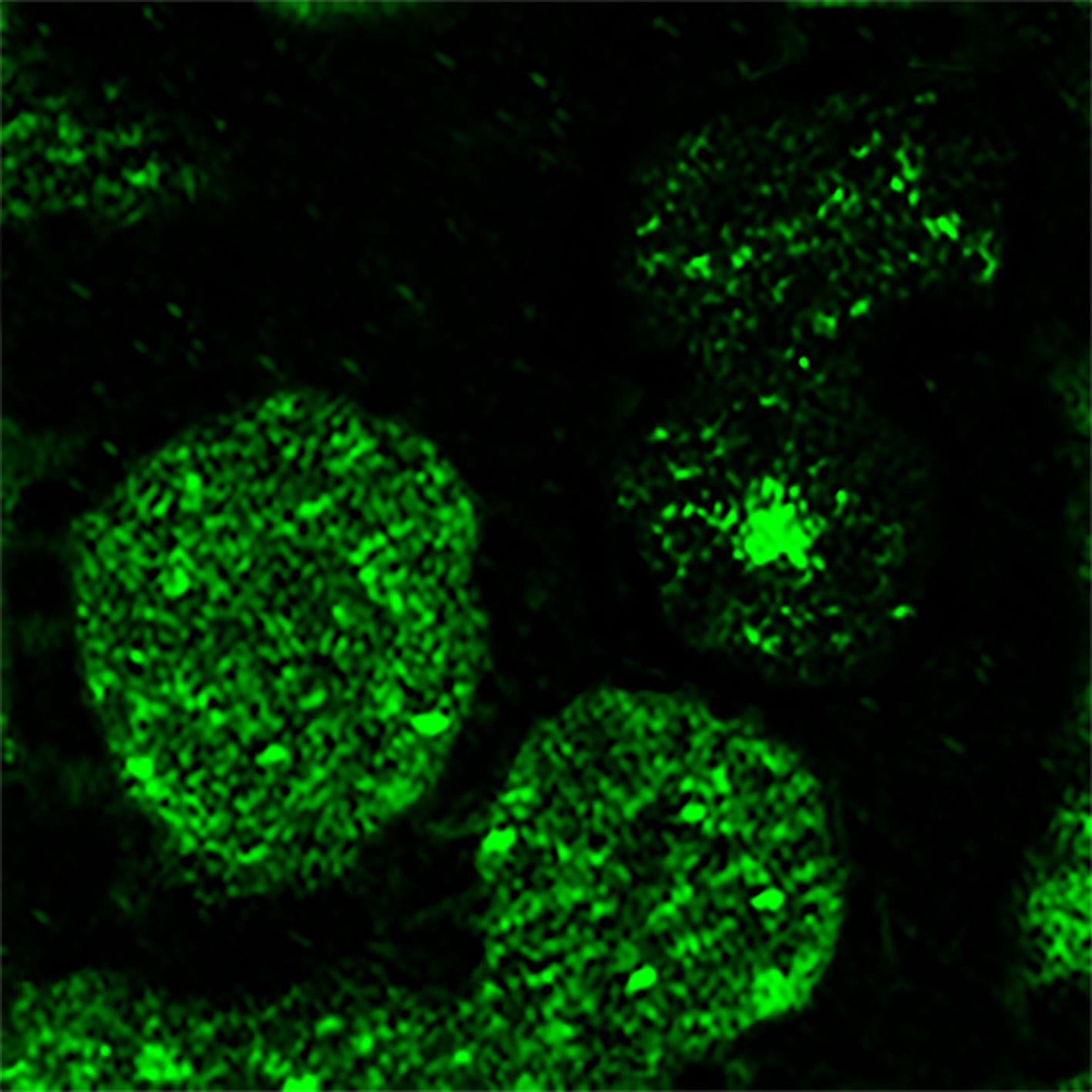

Supplement: Supplementary file 8 — Source data Fig. 1 [file 44318_2024_203_MOESM8_ESM.zip › Figure 1/Figure 1D/Pachytene (St. I-III) -KDM2A.jpg]

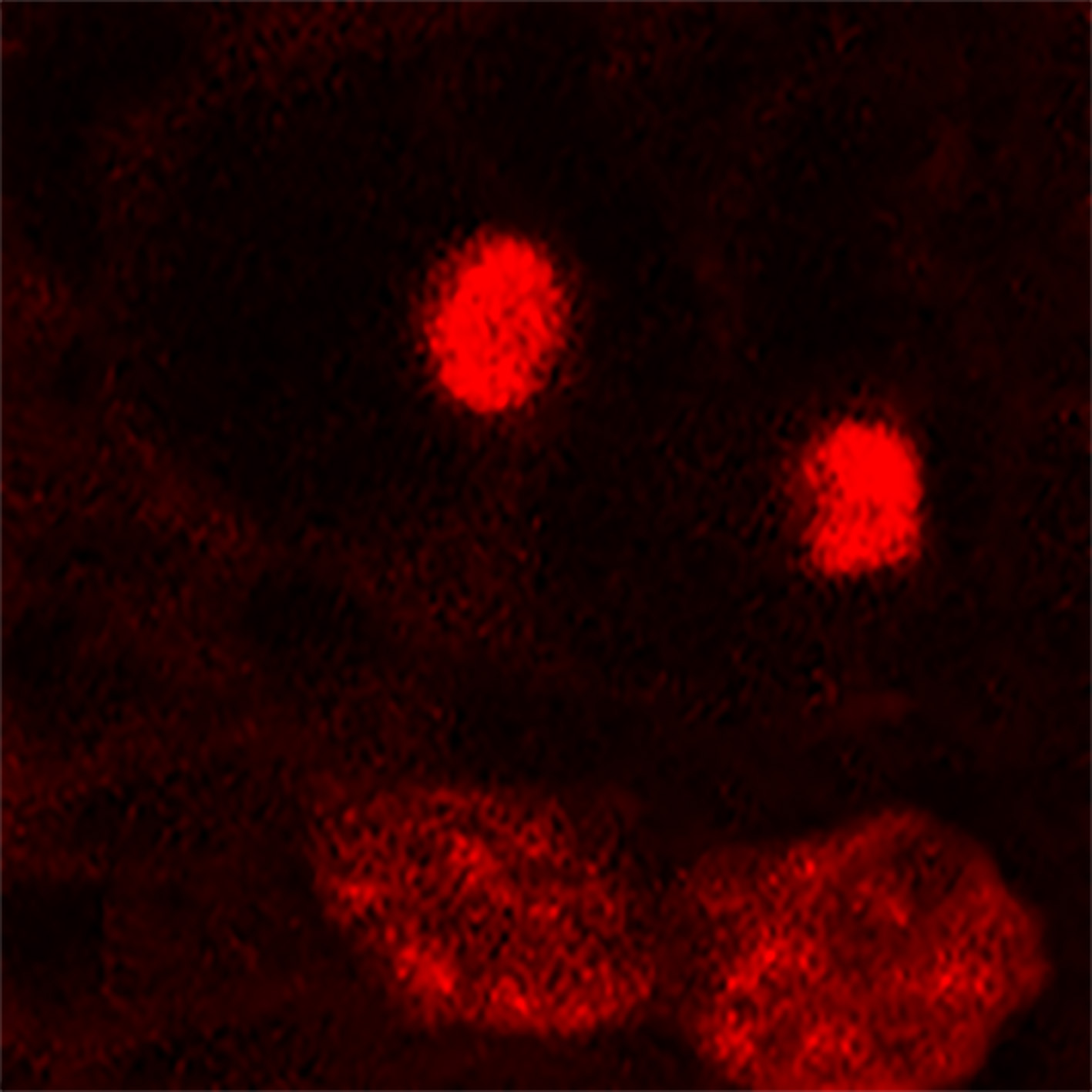

Supplement: Supplementary file 8 — Source data Fig. 1 [file 44318_2024_203_MOESM8_ESM.zip › Figure 1/Figure 1D/Pachytene (St. IX-X) - ╬│H2AX.jpg]

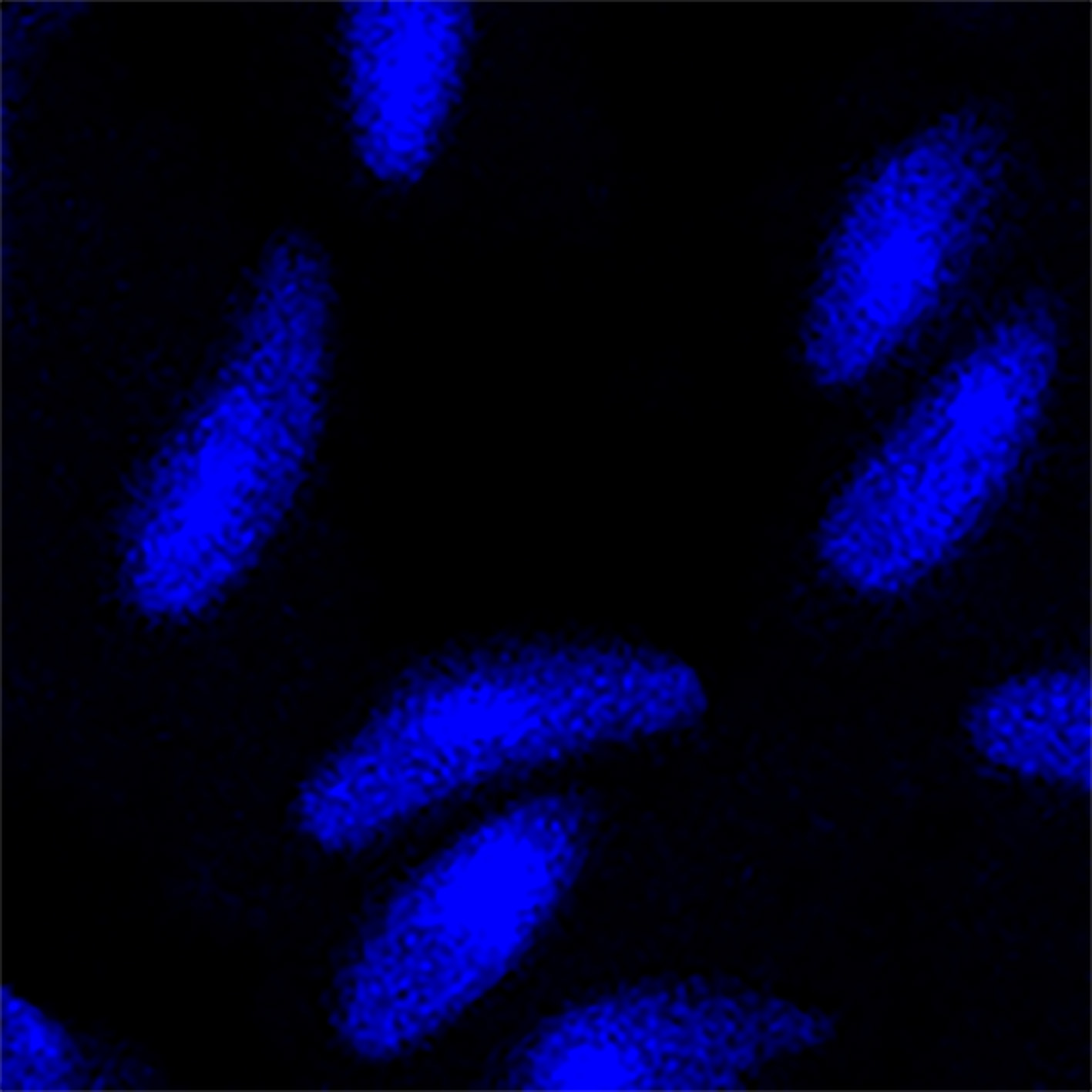

Supplement: Supplementary file 8 — Source data Fig. 1 [file 44318_2024_203_MOESM8_ESM.zip › Figure 1/Figure 1D/Elongated Spermatid (Sp.11-13)- DAPI.jpg]

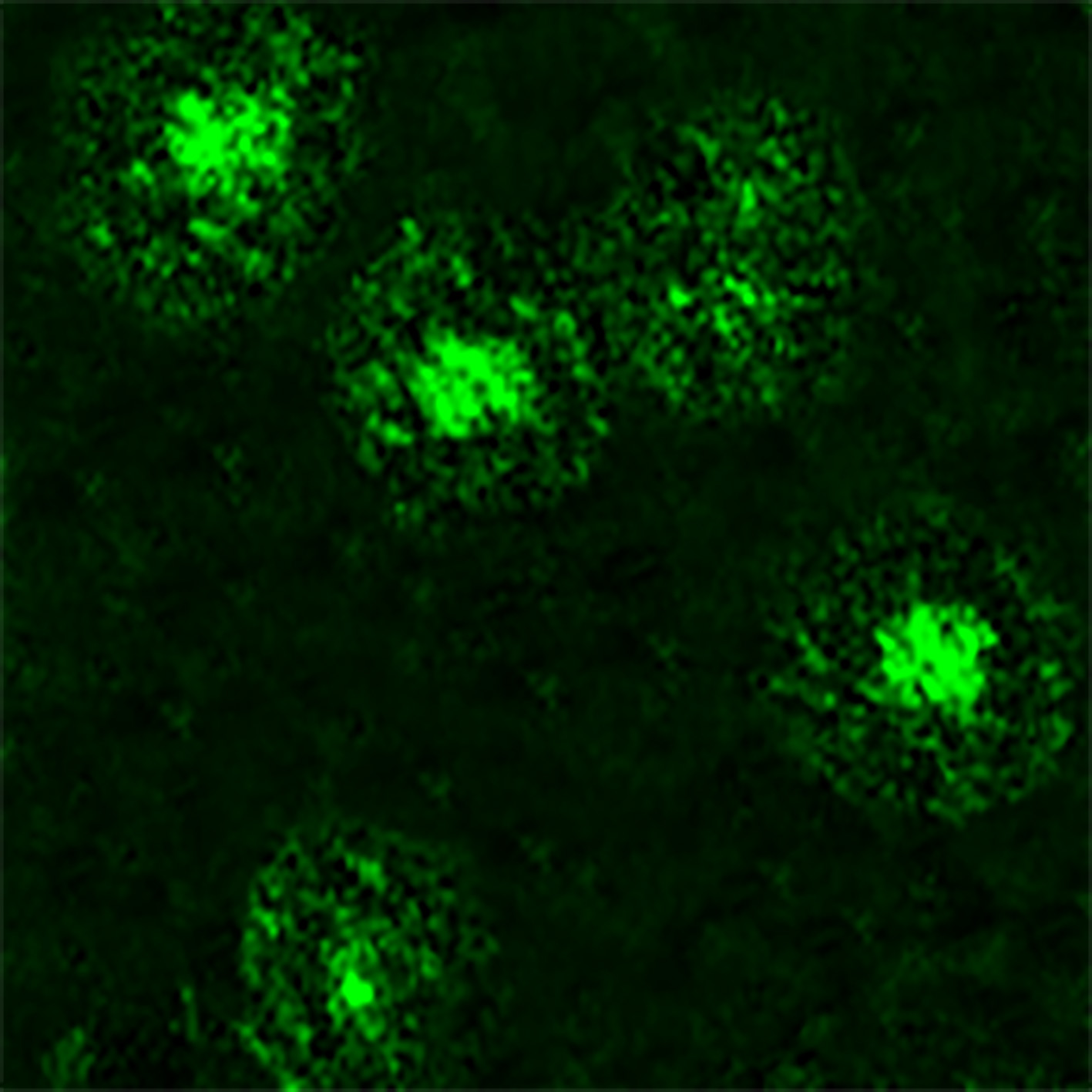

Supplement: Supplementary file 8 — Source data Fig. 1 [file 44318_2024_203_MOESM8_ESM.zip › Figure 1/Figure 1D/Round Spermatid (Sp.2-4)-KDM2A.jpg]

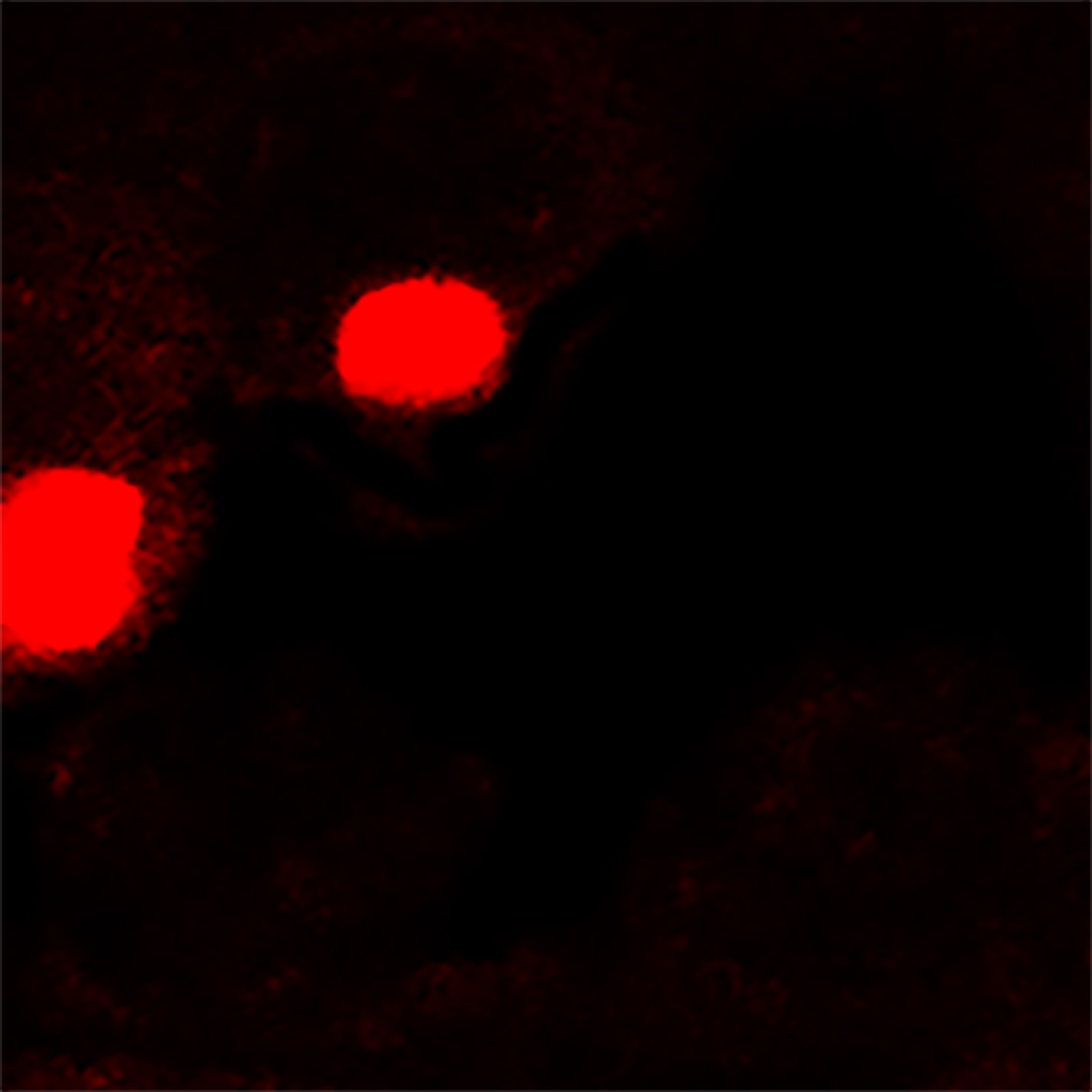

Supplement: Supplementary file 8 — Source data Fig. 1 [file 44318_2024_203_MOESM8_ESM.zip › Figure 1/Figure 1D/Spg- ╬│H2AX.jpg]

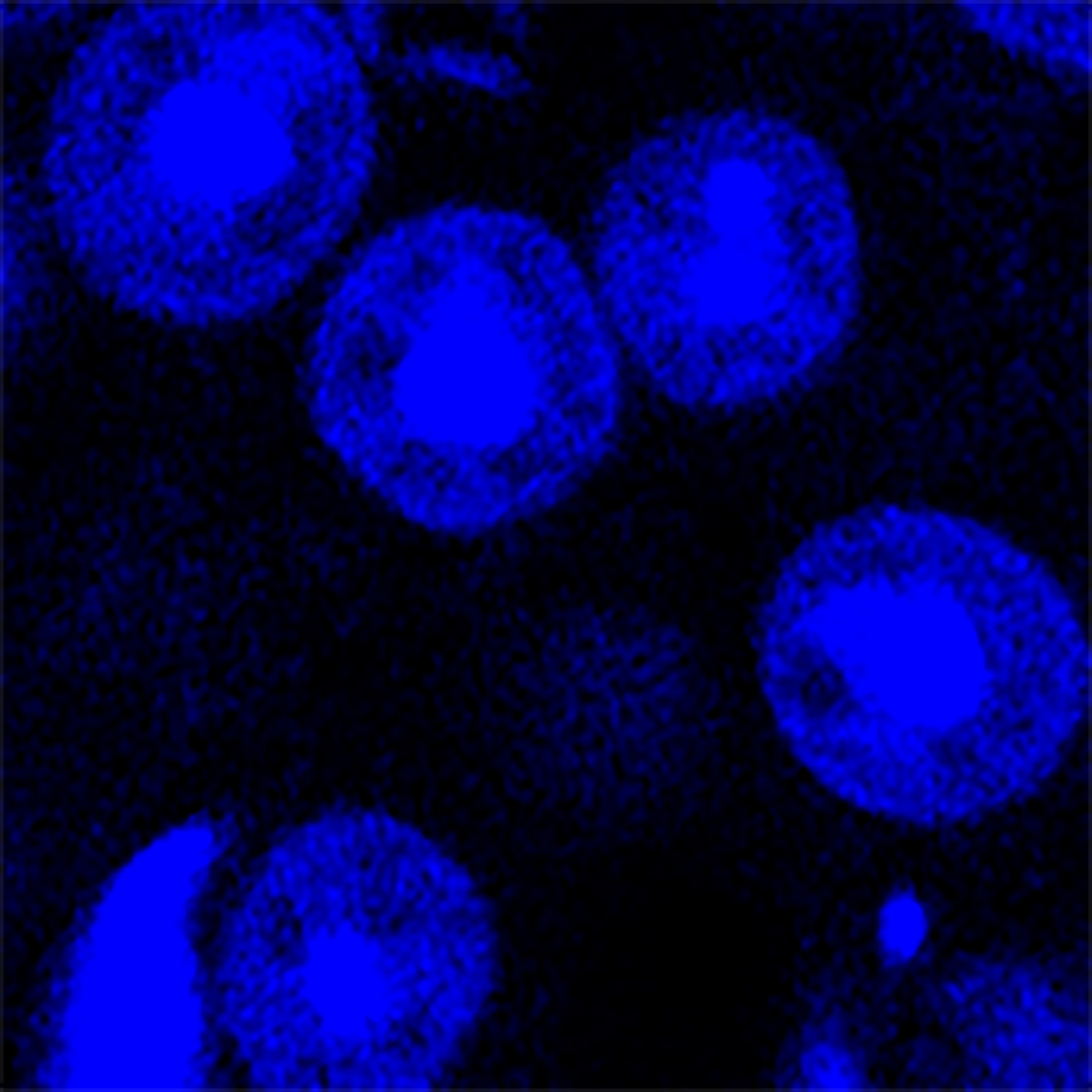

Supplement: Supplementary file 8 — Source data Fig. 1 [file 44318_2024_203_MOESM8_ESM.zip › Figure 1/Figure 1D/Round Spermatid (Sp.2-4) - DAPI.jpg]

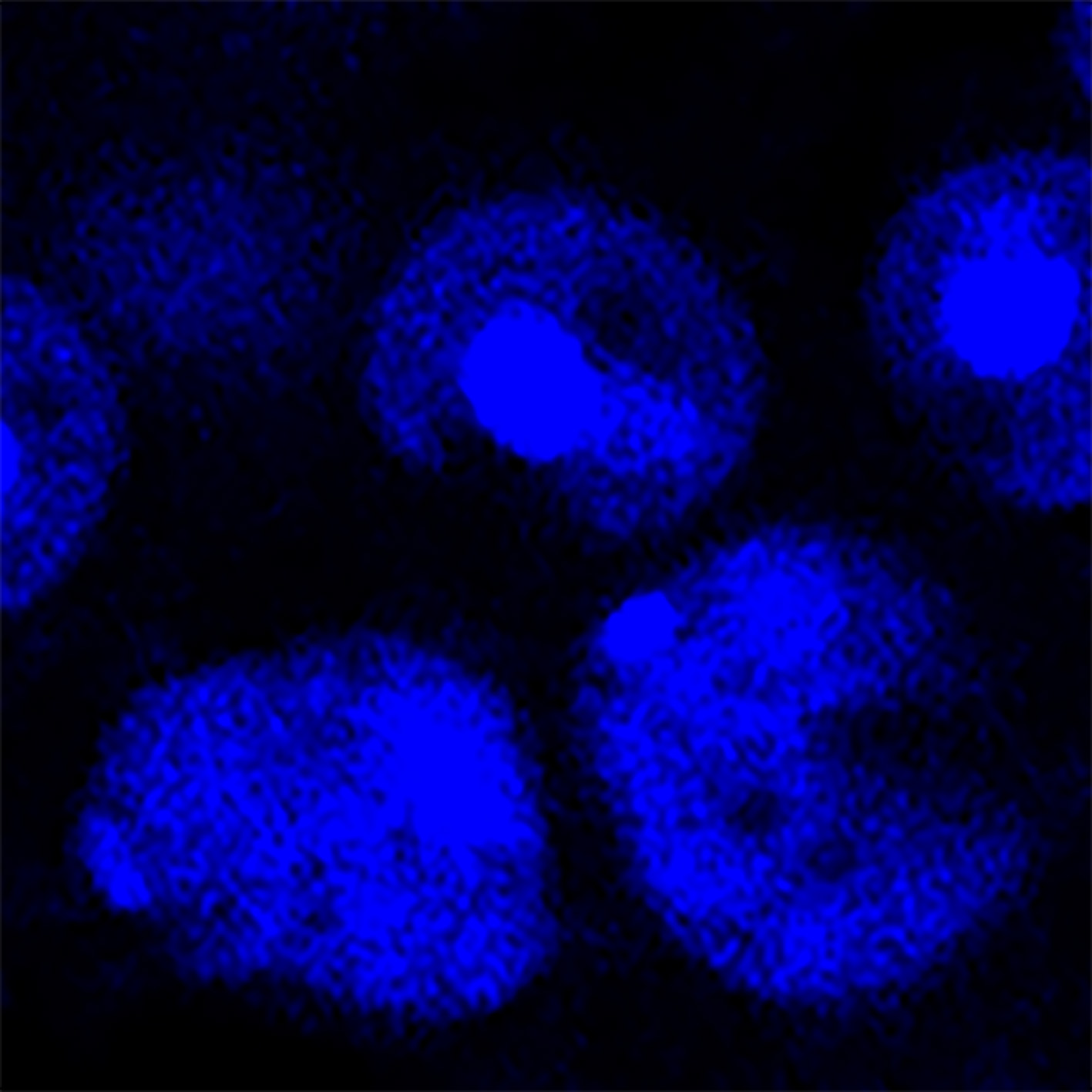

Supplement: Supplementary file 8 — Source data Fig. 1 [file 44318_2024_203_MOESM8_ESM.zip › Figure 1/Figure 1D/Pachytene (St. IV-VI) - DAPI.jpg]

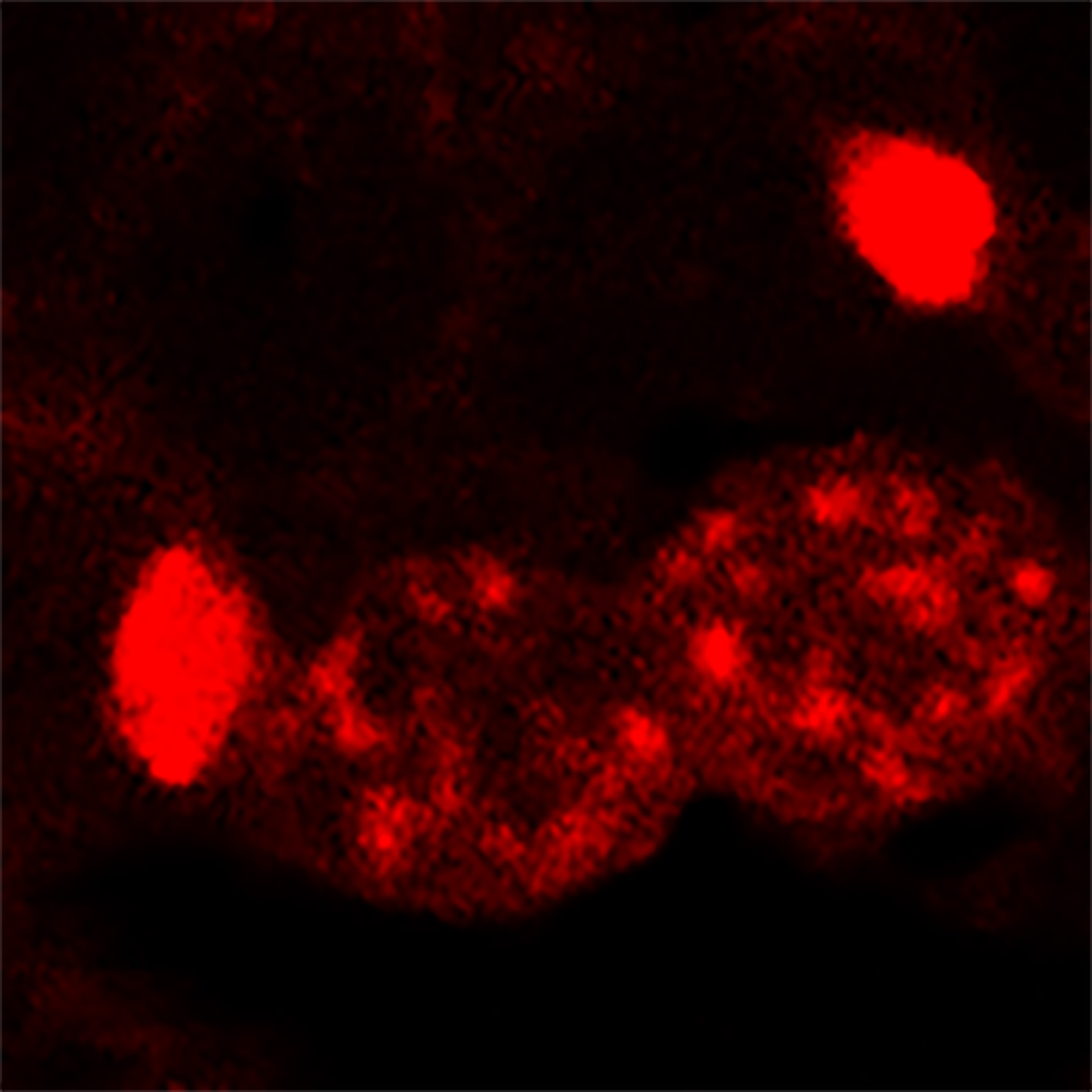

Supplement: Supplementary file 8 — Source data Fig. 1 [file 44318_2024_203_MOESM8_ESM.zip › Figure 1/Figure 1D/Leptene - ╬│H2AX.jpg]

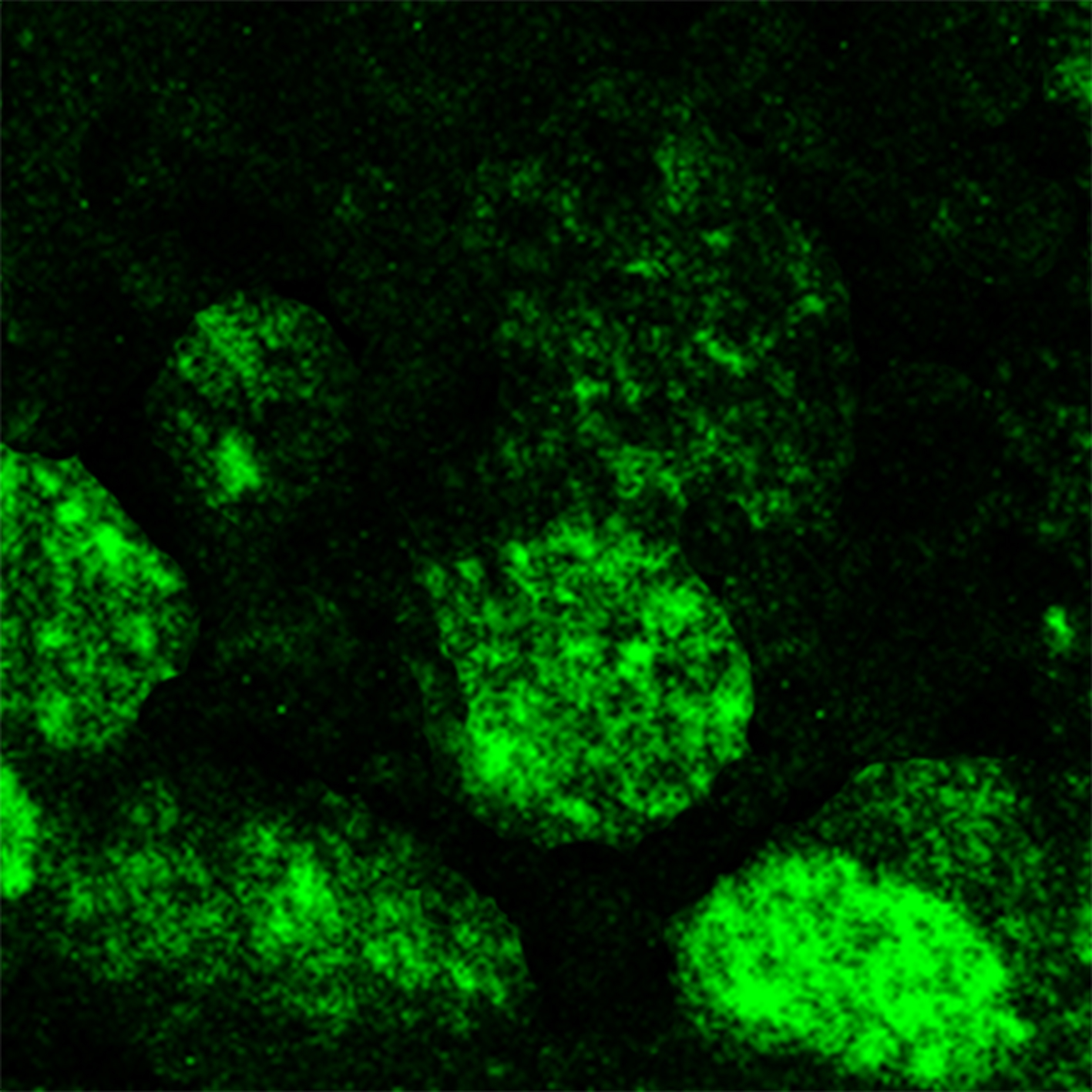

Supplement: Supplementary file 8 — Source data Fig. 1 [file 44318_2024_203_MOESM8_ESM.zip › Figure 1/Figure 1D/Diplotene-KDM2A.jpg]

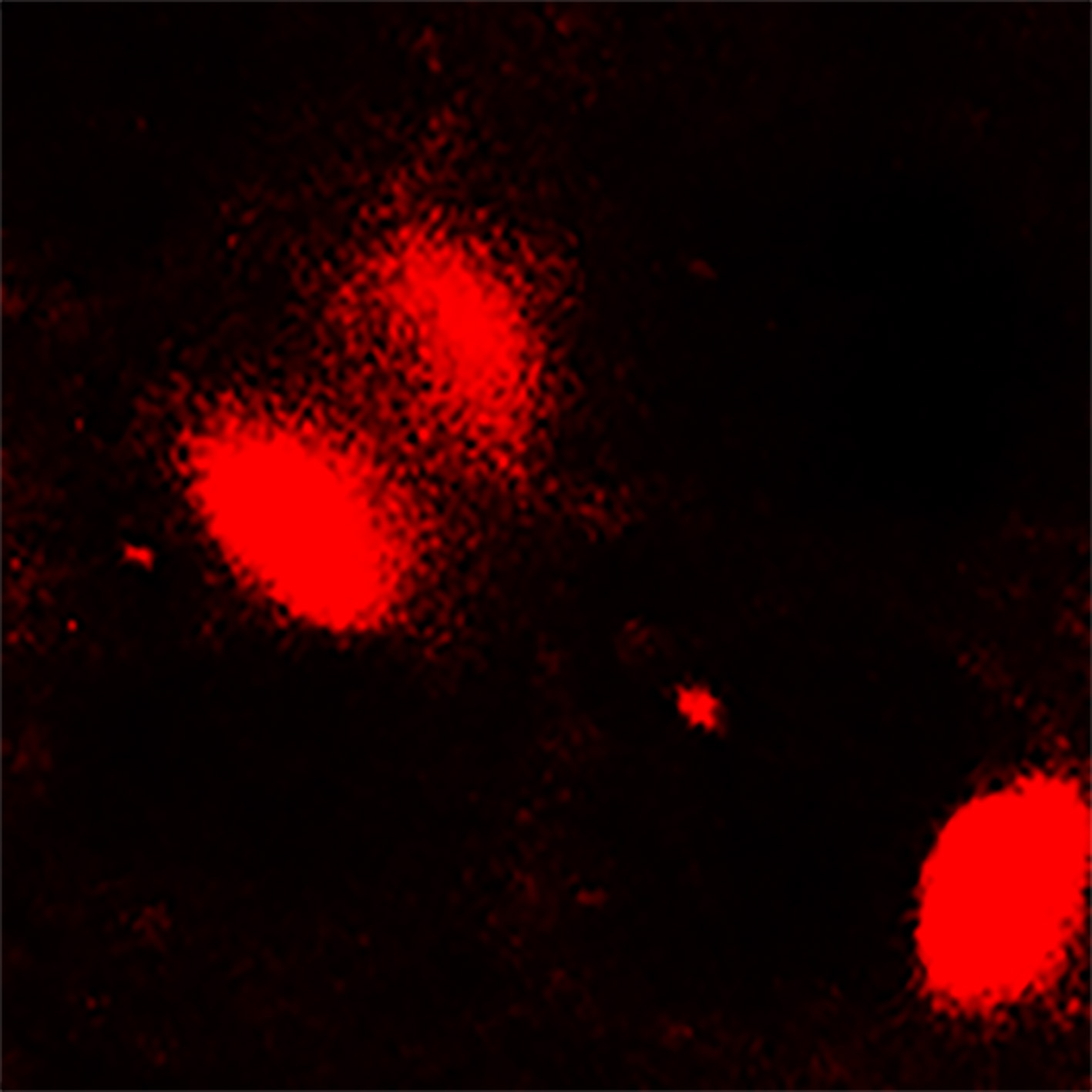

Supplement: Supplementary file 8 — Source data Fig. 1 [file 44318_2024_203_MOESM8_ESM.zip › Figure 1/Figure 1D/Pachytene (St. VII-VIII) - ╬│H2AX.jpg]

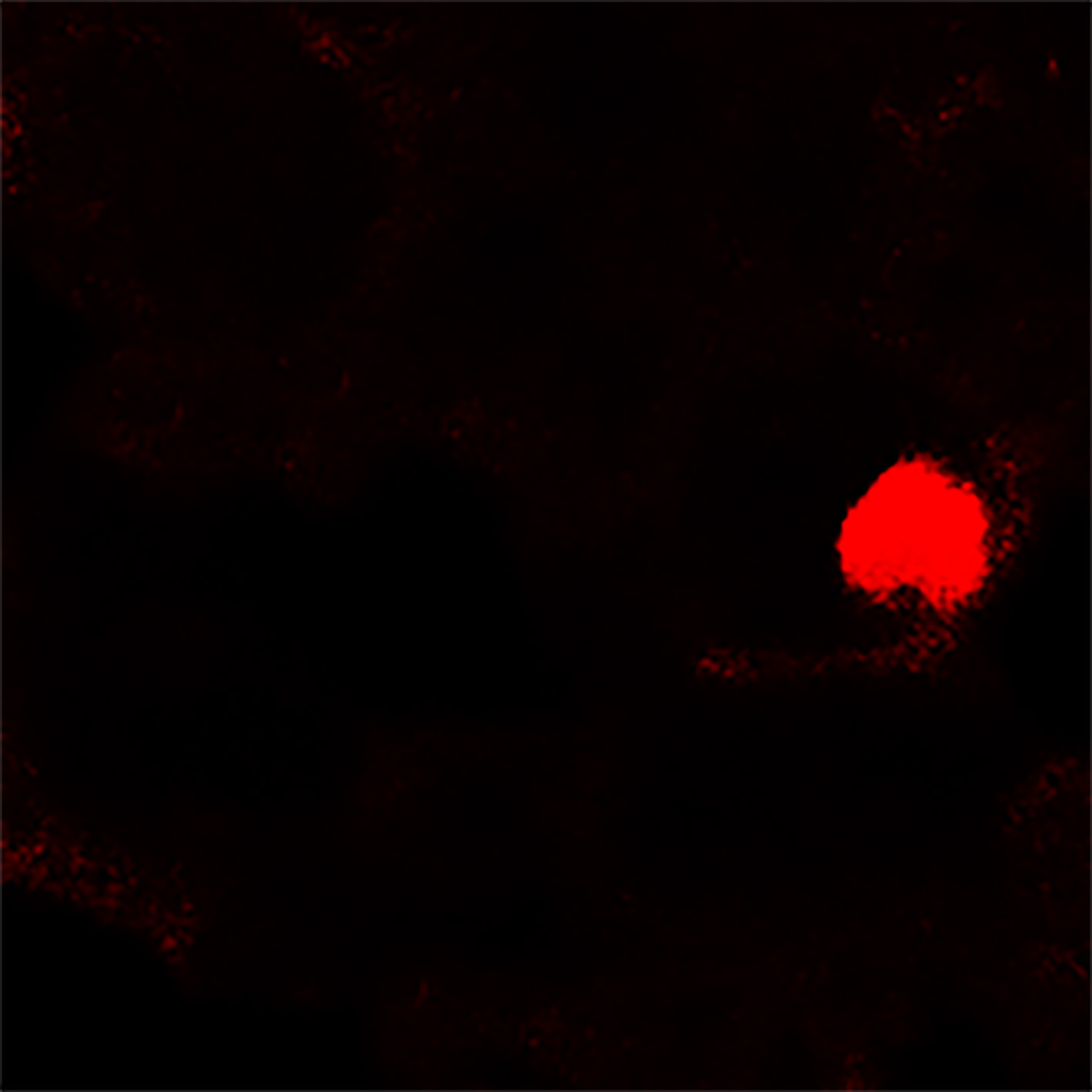

Supplement: Supplementary file 8 — Source data Fig. 1 [file 44318_2024_203_MOESM8_ESM.zip › Figure 1/Figure 1D/Sertoli cell - ╬│H2AX.jpg]

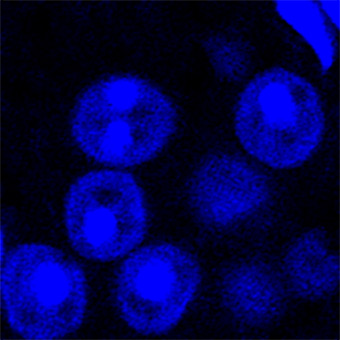

Supplement: Supplementary file 8 — Source data Fig. 1 [file 44318_2024_203_MOESM8_ESM.zip › Figure 1/Figure 1D/Round Spermatid (Sp.5-6)- DAPI.jpg]

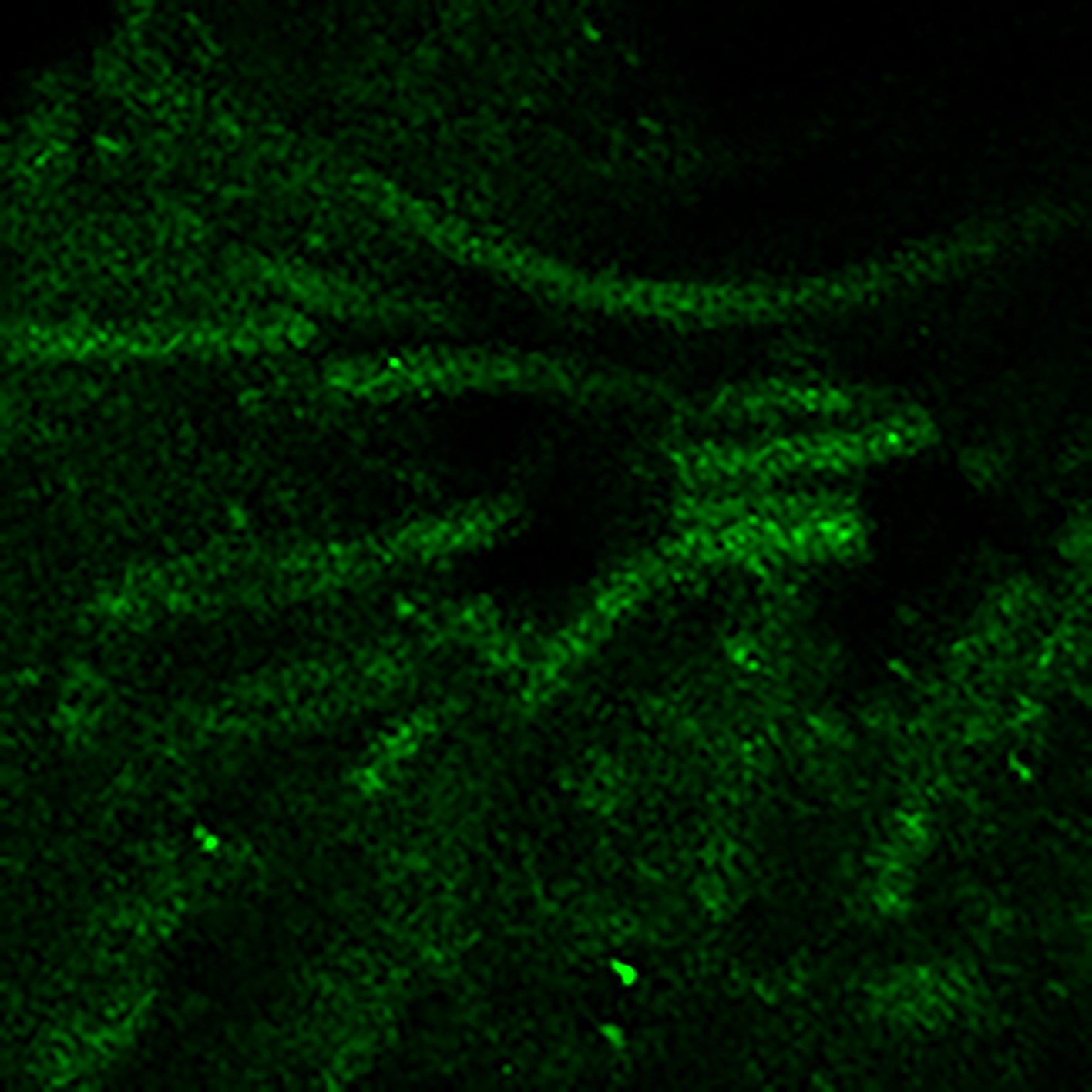

Supplement: Supplementary file 8 — Source data Fig. 1 [file 44318_2024_203_MOESM8_ESM.zip › Figure 1/Figure 1D/Elongated Spermatid (Sp.14-16) -KDM2A.jpg]

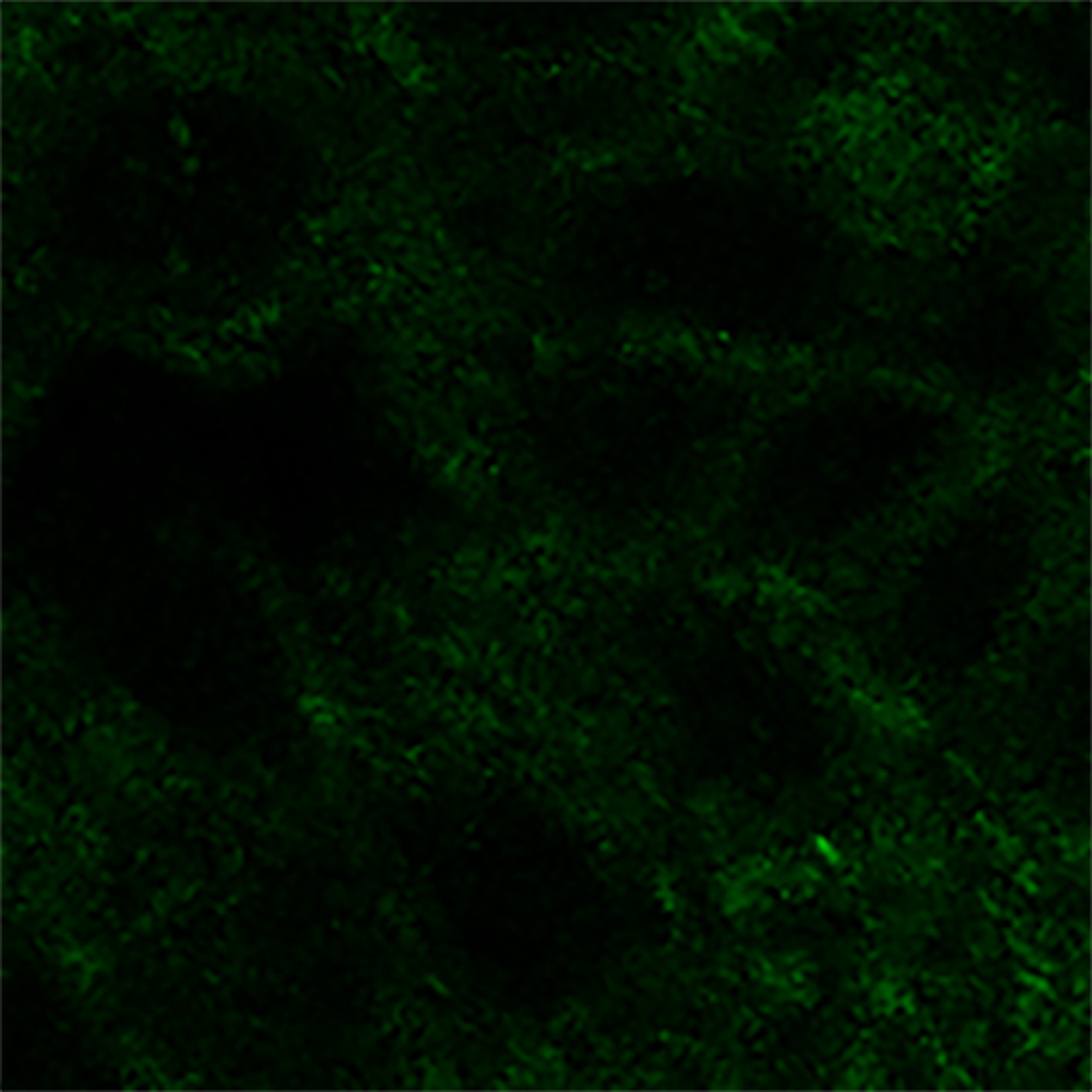

Supplement: Supplementary file 8 — Source data Fig. 1 [file 44318_2024_203_MOESM8_ESM.zip › Figure 1/Figure 1D/Elongating Spermatid (Sp.9-10)-KDM2A.jpg]

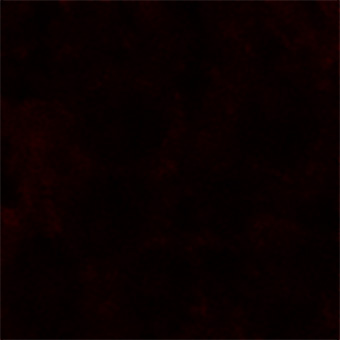

Supplement: Supplementary file 8 — Source data Fig. 1 [file 44318_2024_203_MOESM8_ESM.zip › Figure 1/Figure 1D/Round Spermatid (Sp.5-6) - ╬│H2AX.jpg]

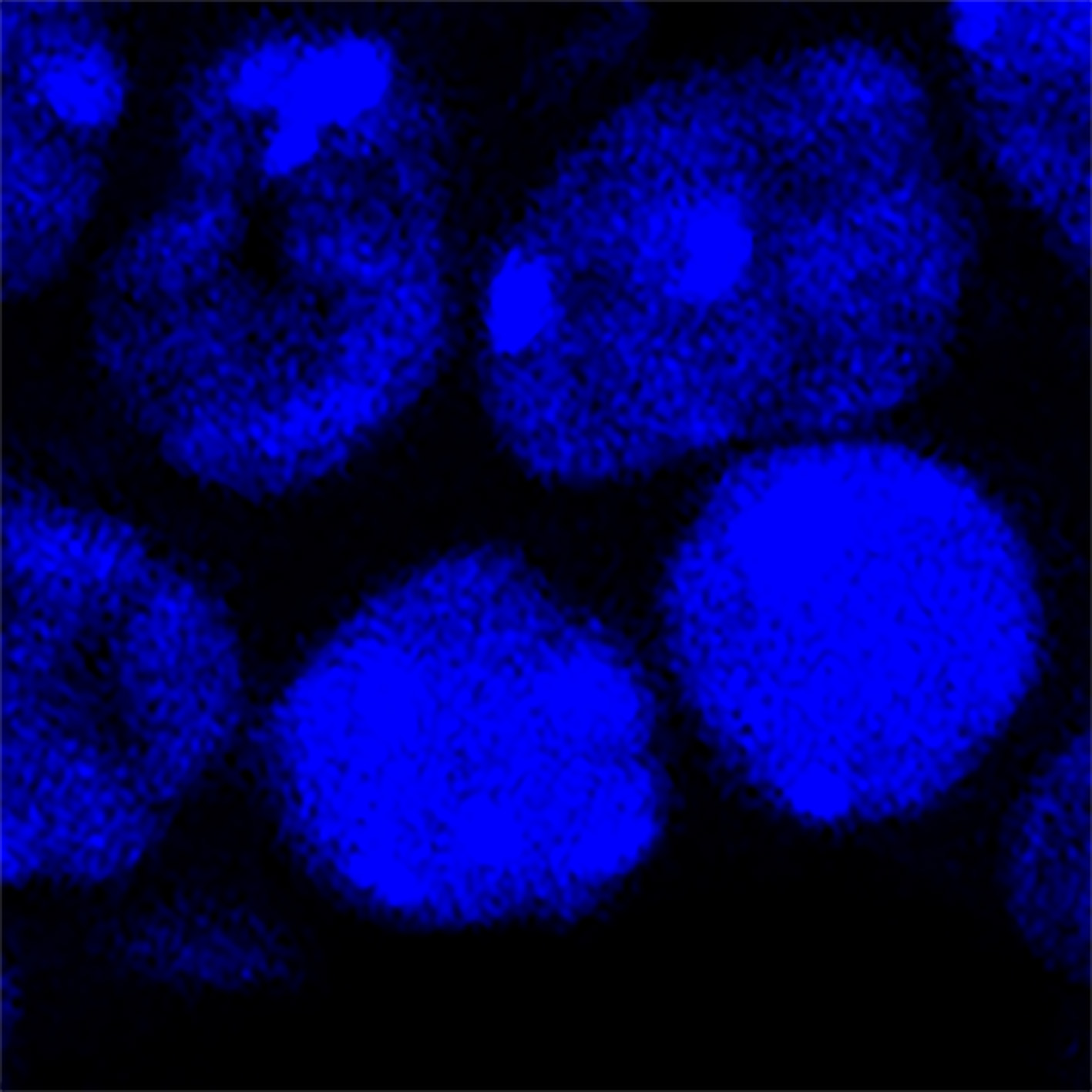

Supplement: Supplementary file 8 — Source data Fig. 1 [file 44318_2024_203_MOESM8_ESM.zip › Figure 1/Figure 1D/Leptene- DAPI.jpg]

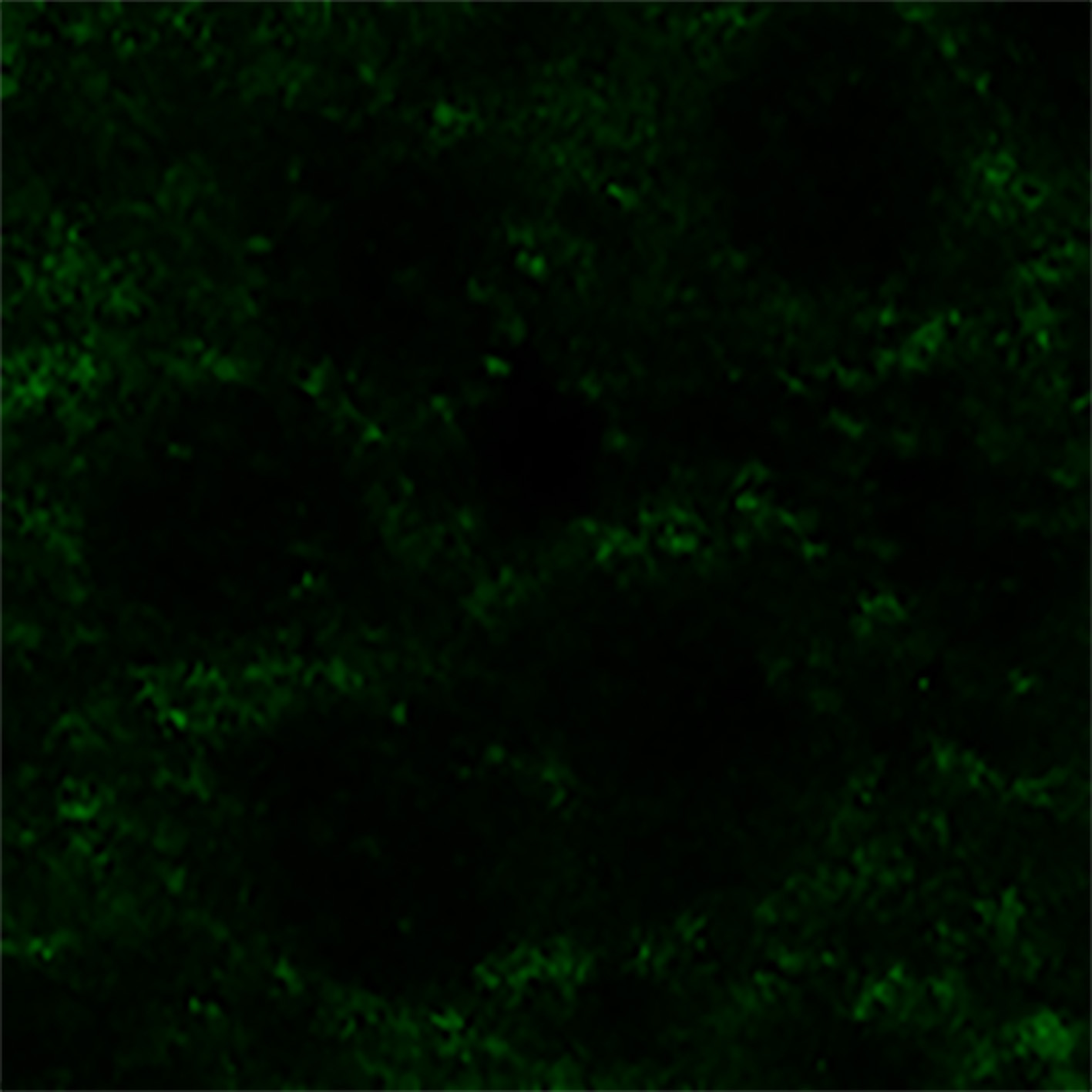

Supplement: Supplementary file 8 — Source data Fig. 1 [file 44318_2024_203_MOESM8_ESM.zip › Figure 1/Figure 1D/Elongating Spermatid (Sp.7-8)-KDM2A.jpg]

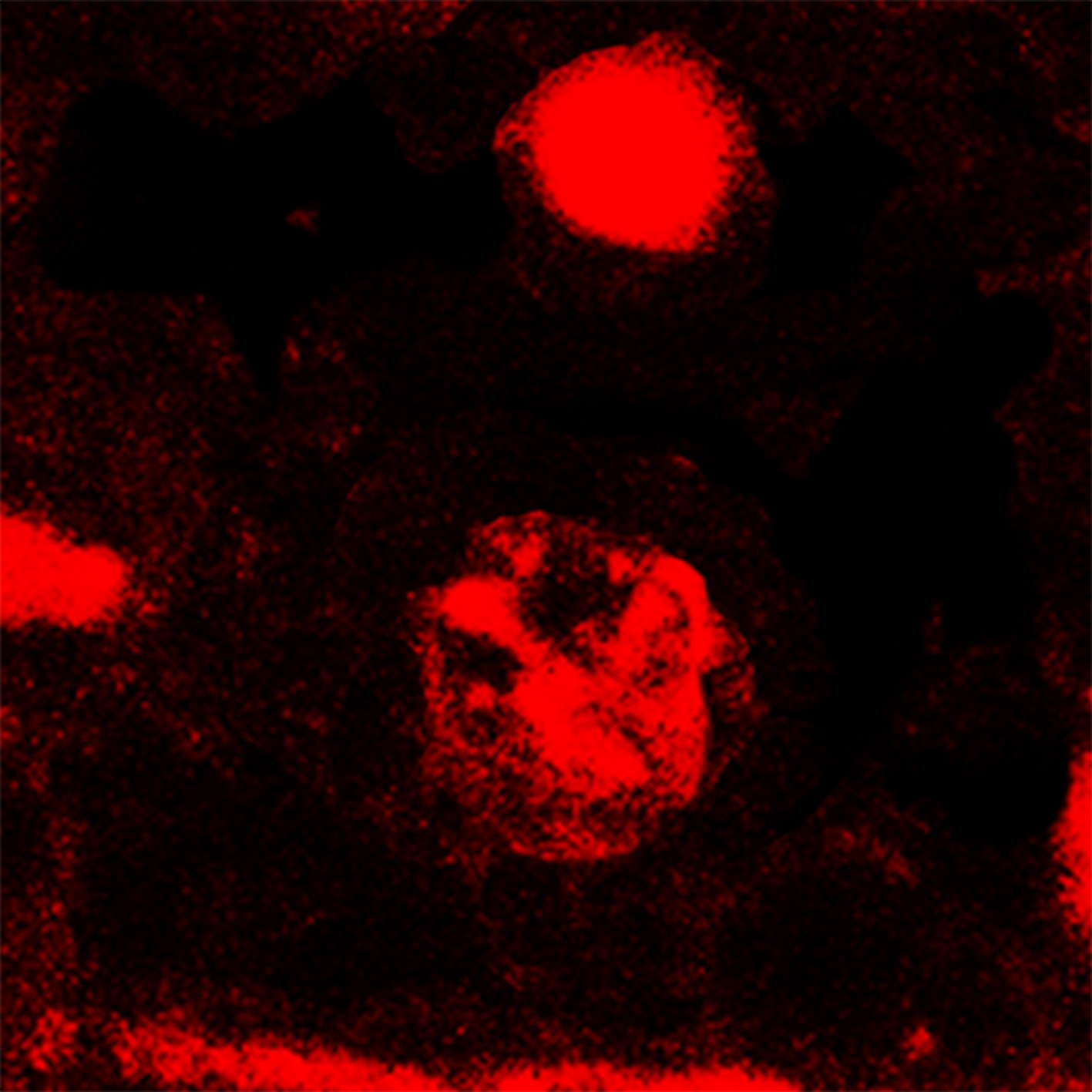

Supplement: Supplementary file 8 — Source data Fig. 1 [file 44318_2024_203_MOESM8_ESM.zip › Figure 1/Figure 1D/Diplotene - ╬│H2AX.jpg]

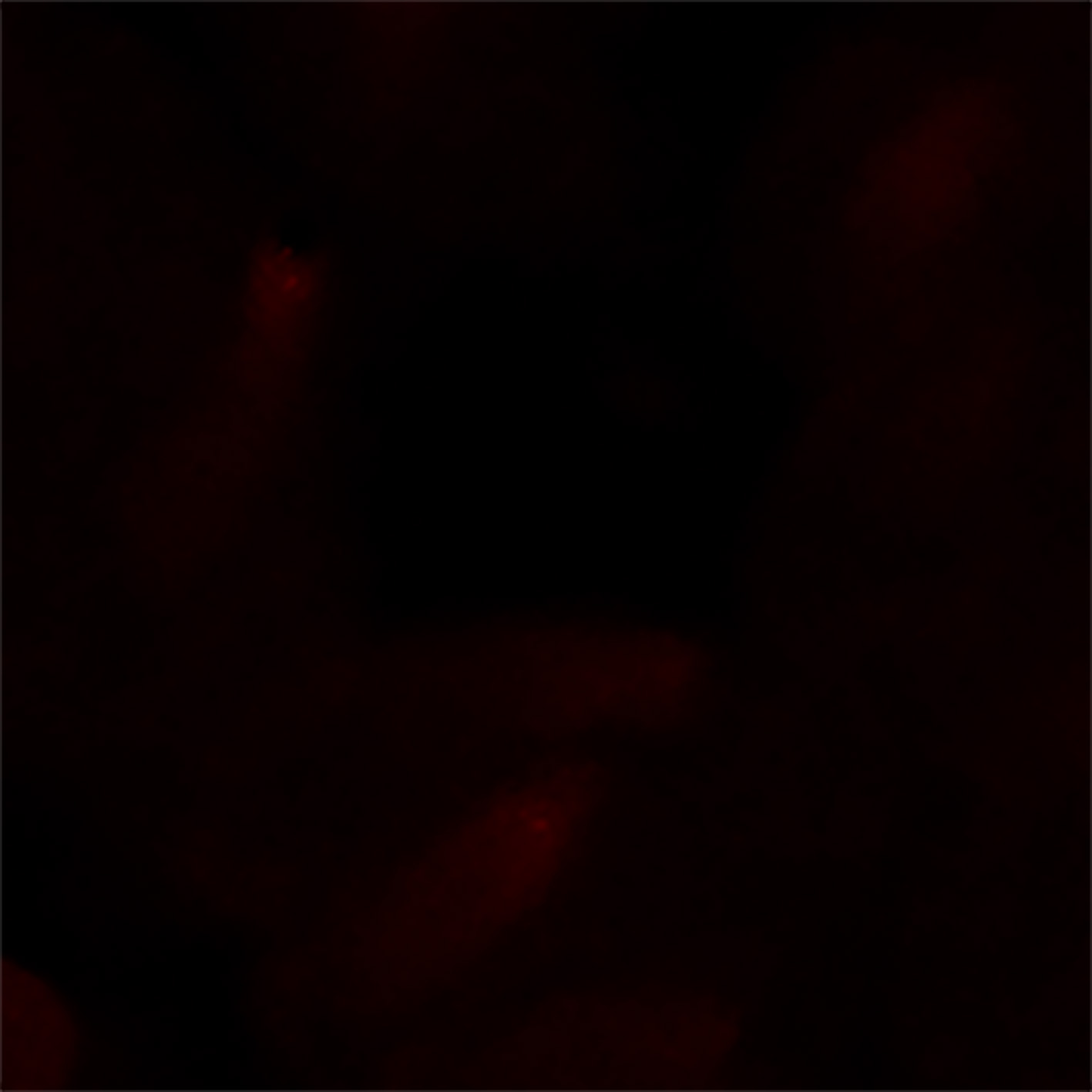

Supplement: Supplementary file 8 — Source data Fig. 1 [file 44318_2024_203_MOESM8_ESM.zip › Figure 1/Figure 1D/Elongated Spermatid (Sp.11-13) - ╬│H2AX.jpg]

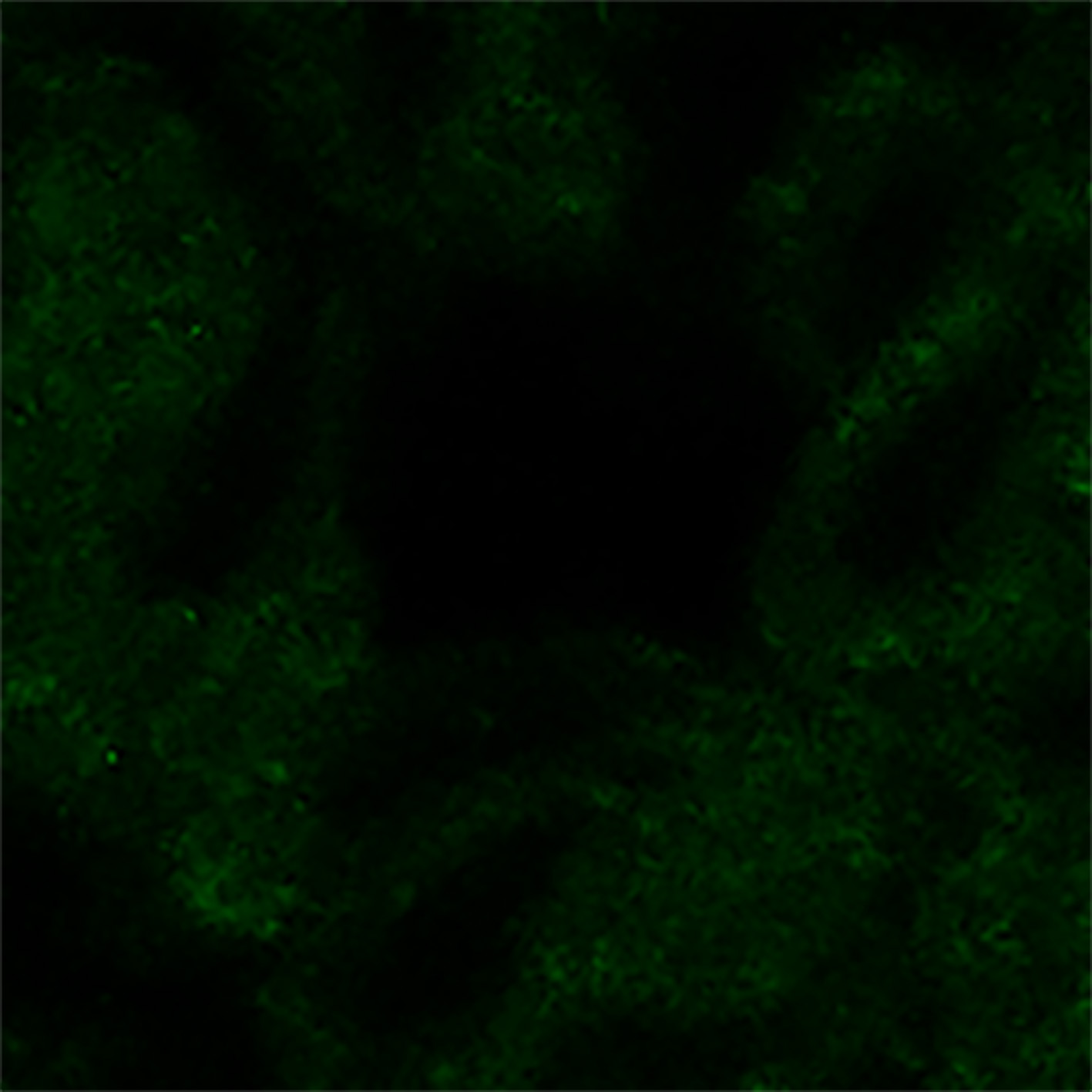

Supplement: Supplementary file 8 — Source data Fig. 1 [file 44318_2024_203_MOESM8_ESM.zip › Figure 1/Figure 1D/Elongated Spermatid (Sp.11-13)-KDM2A.jpg]

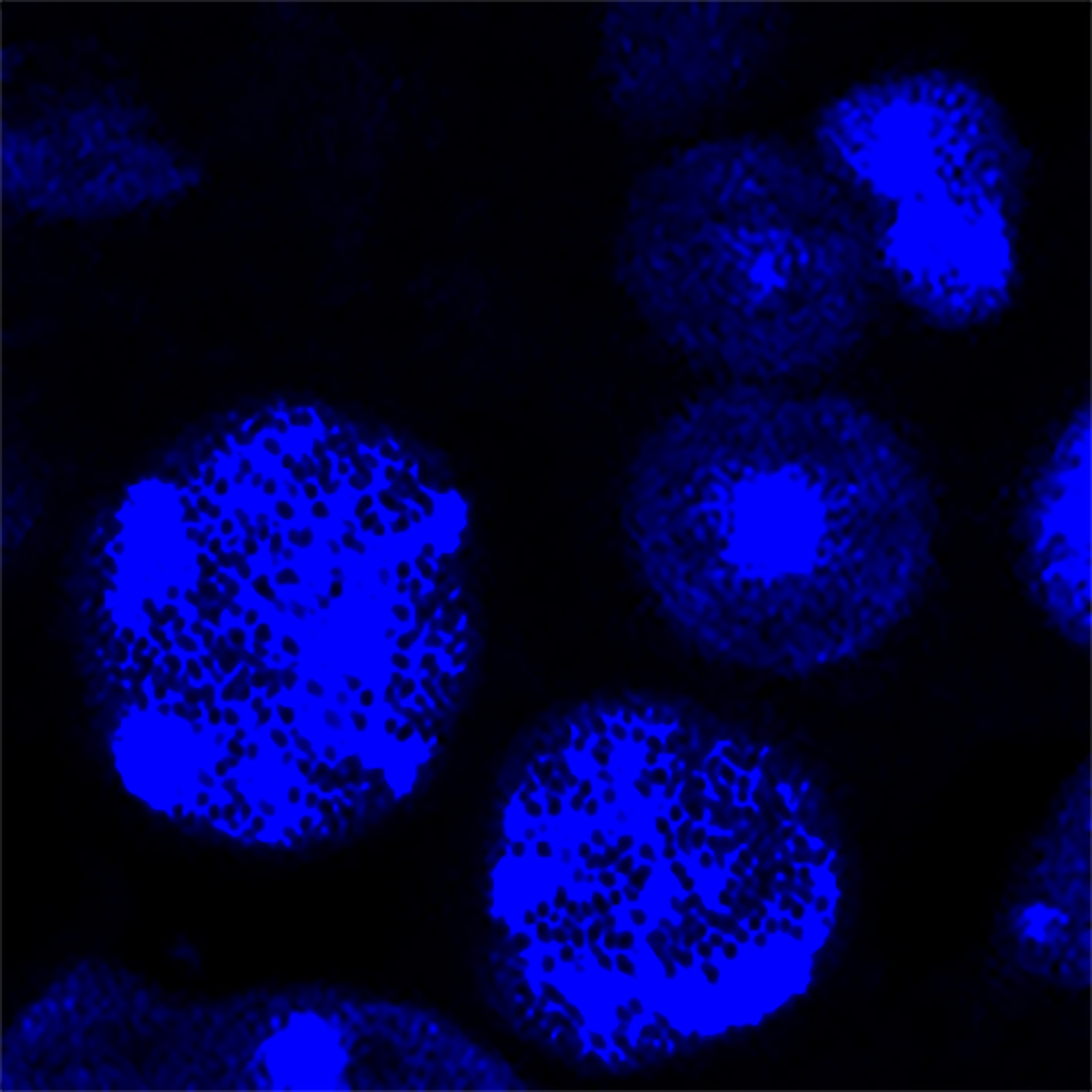

Supplement: Supplementary file 8 — Source data Fig. 1 [file 44318_2024_203_MOESM8_ESM.zip › Figure 1/Figure 1D/Pachytene (St. I-III) - DAPI.jpg]

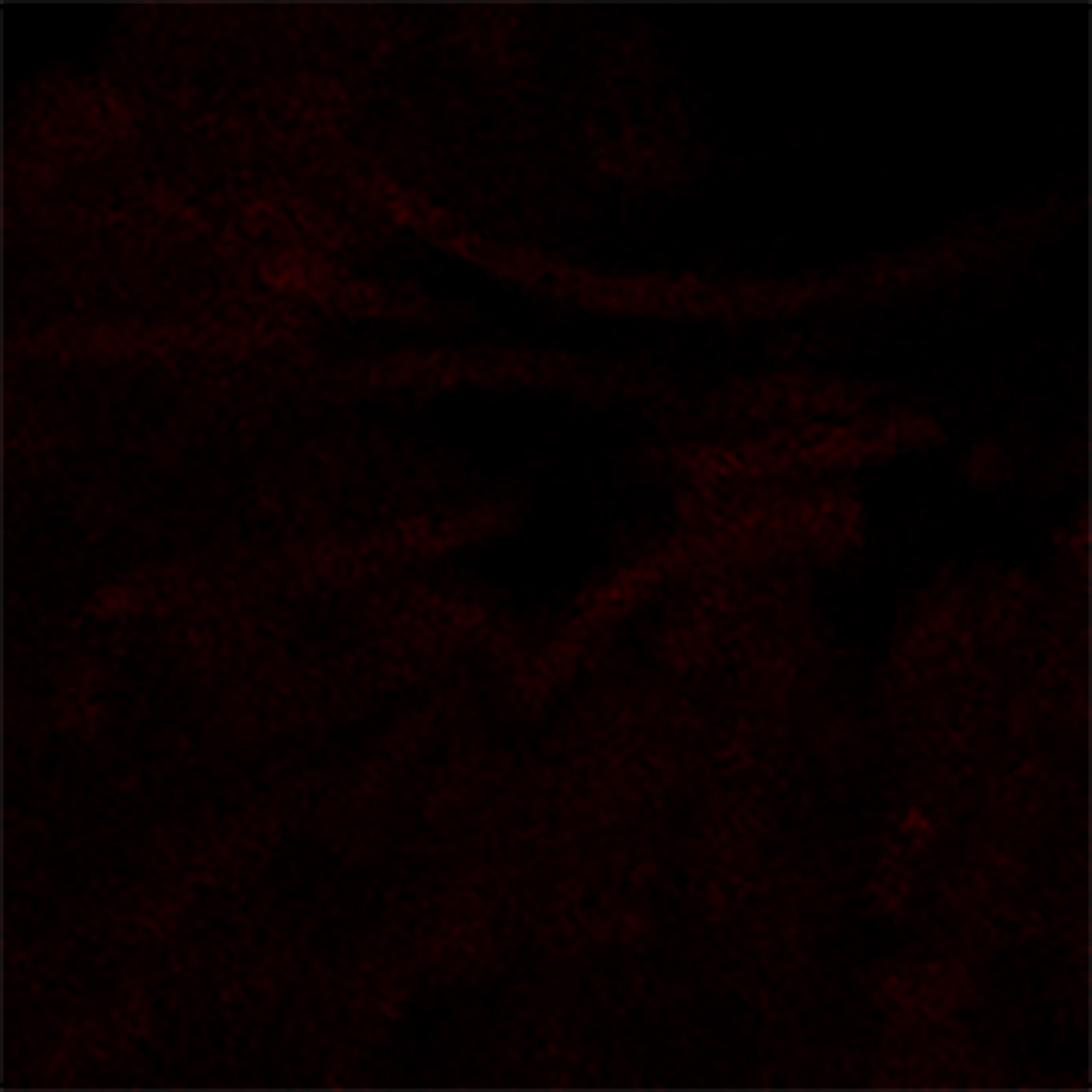

Supplement: Supplementary file 8 — Source data Fig. 1 [file 44318_2024_203_MOESM8_ESM.zip › Figure 1/Figure 1D/Elongated Spermatid (Sp.14-16) - ╬│H2AX.jpg]

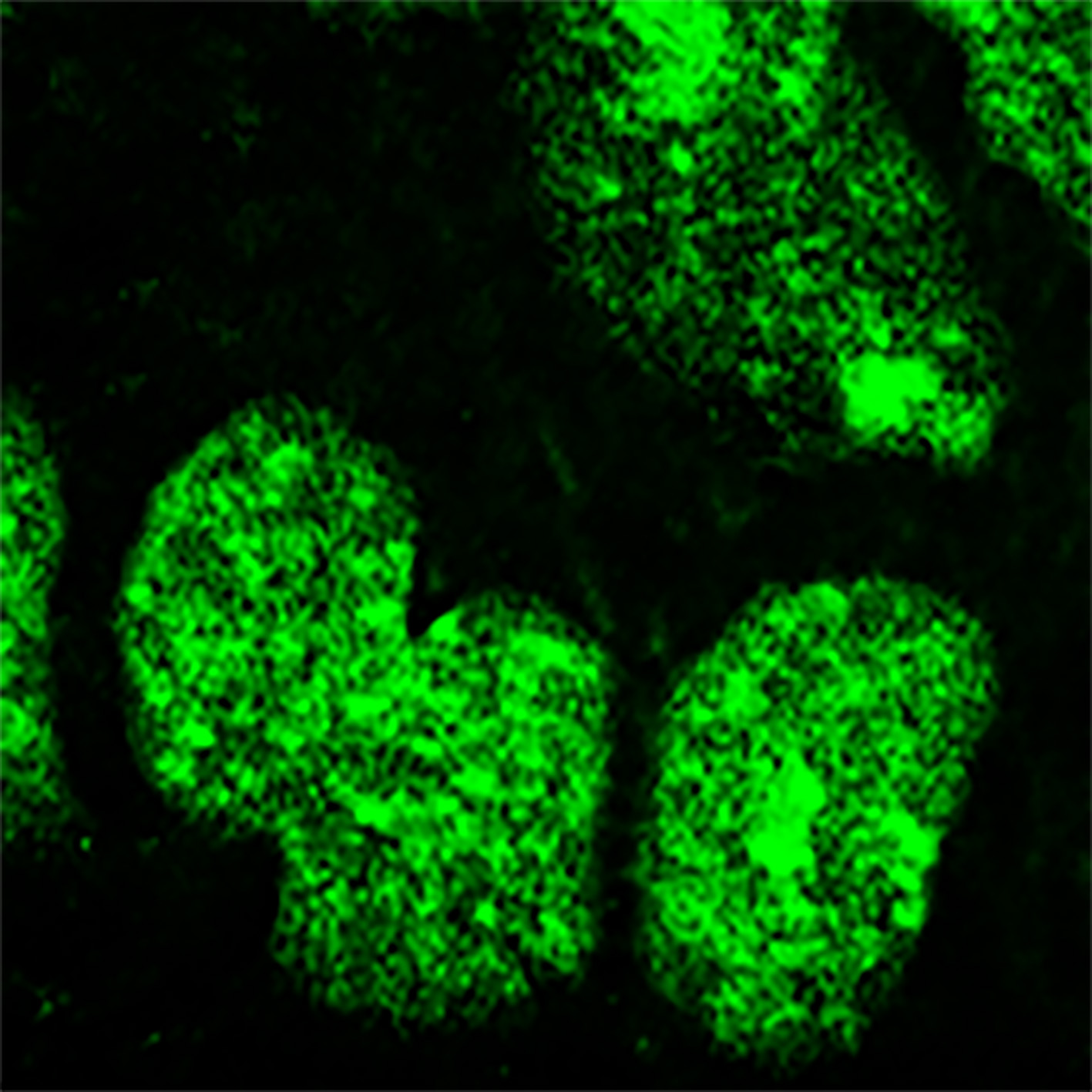

Supplement: Supplementary file 8 — Source data Fig. 1 [file 44318_2024_203_MOESM8_ESM.zip › Figure 1/Figure 1D/Pre-leptotene - KDM2A.jpg]

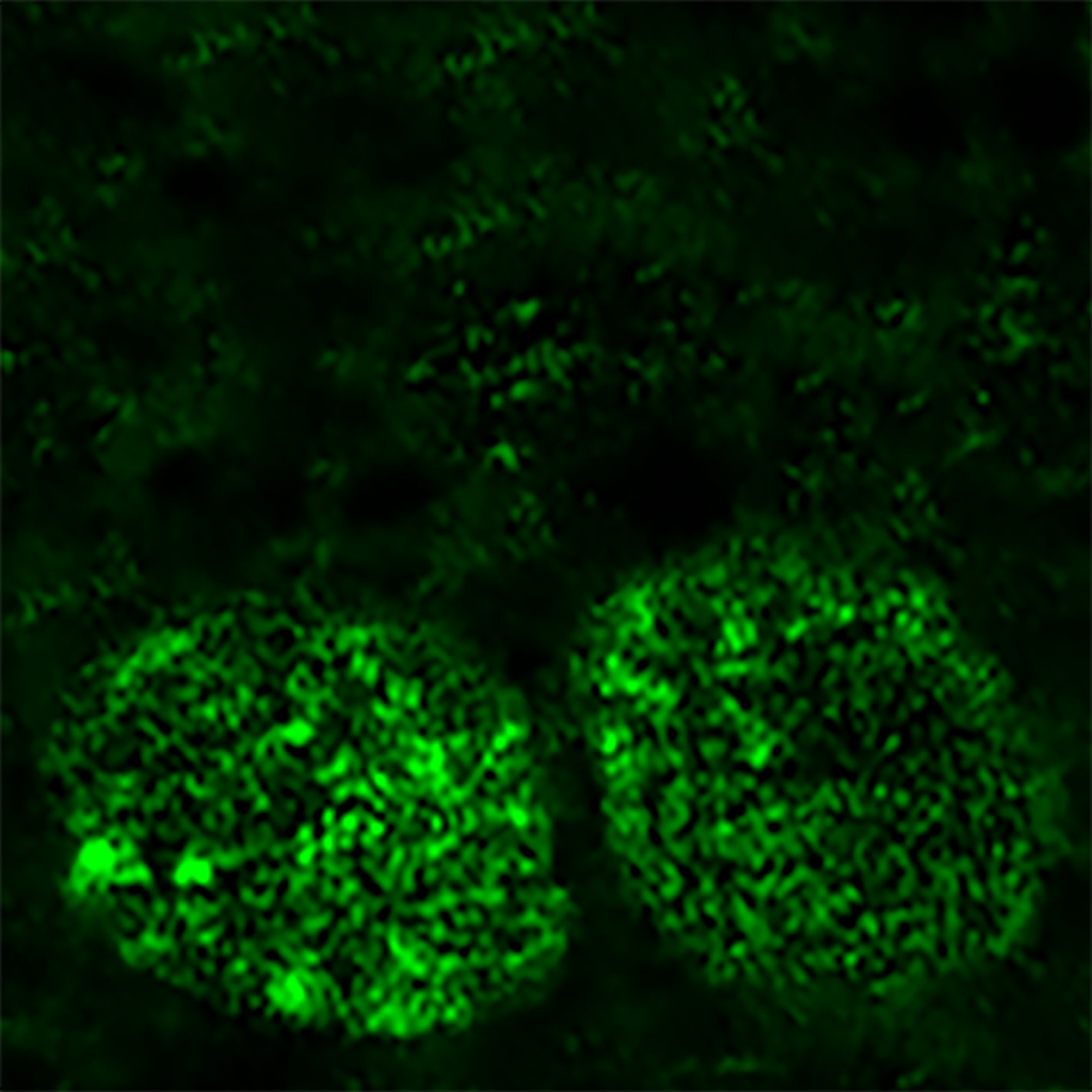

Supplement: Supplementary file 8 — Source data Fig. 1 [file 44318_2024_203_MOESM8_ESM.zip › Figure 1/Figure 1D/Pachytene (St. IV-VI) -KDM2A.jpg]

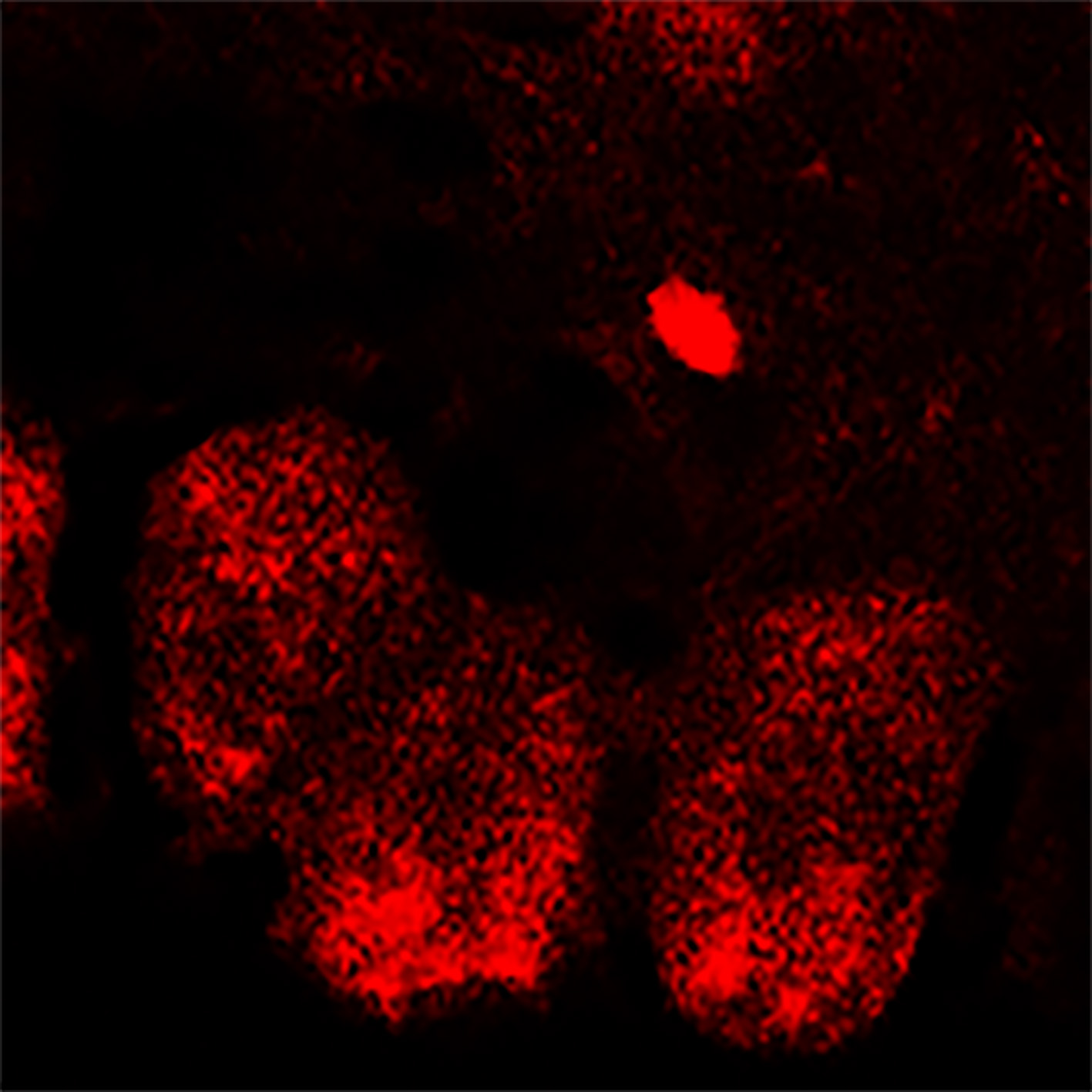

Supplement: Supplementary file 8 — Source data Fig. 1 [file 44318_2024_203_MOESM8_ESM.zip › Figure 1/Figure 1D/Pre-leptotene - ╬│H2AX.jpg]

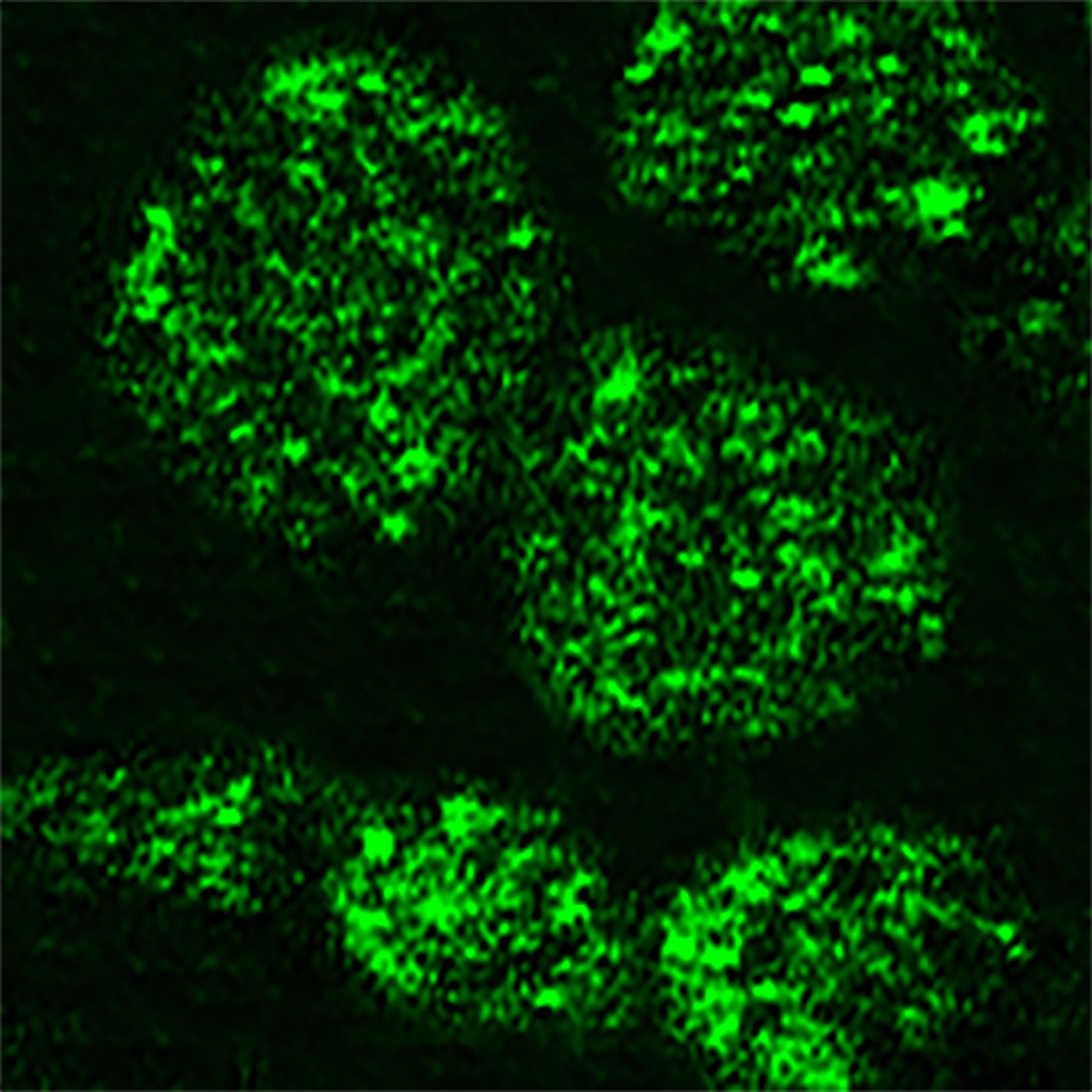

Supplement: Supplementary file 8 — Source data Fig. 1 [file 44318_2024_203_MOESM8_ESM.zip › Figure 1/Figure 1D/Pachytene (St. IX-X)-KDM2A.jpg]

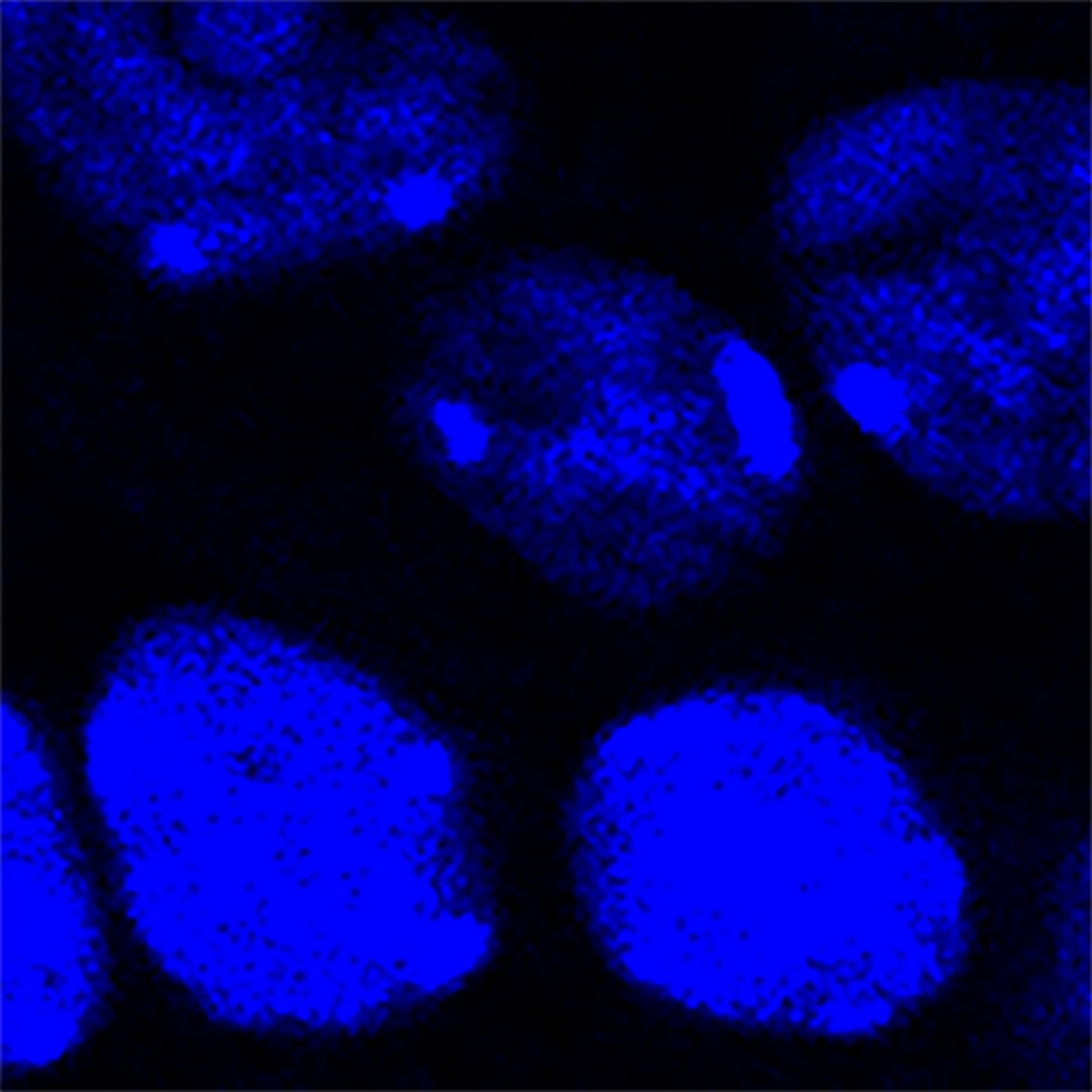

Supplement: Supplementary file 8 — Source data Fig. 1 [file 44318_2024_203_MOESM8_ESM.zip › Figure 1/Figure 1D/Zagotene- DAPI.jpg]

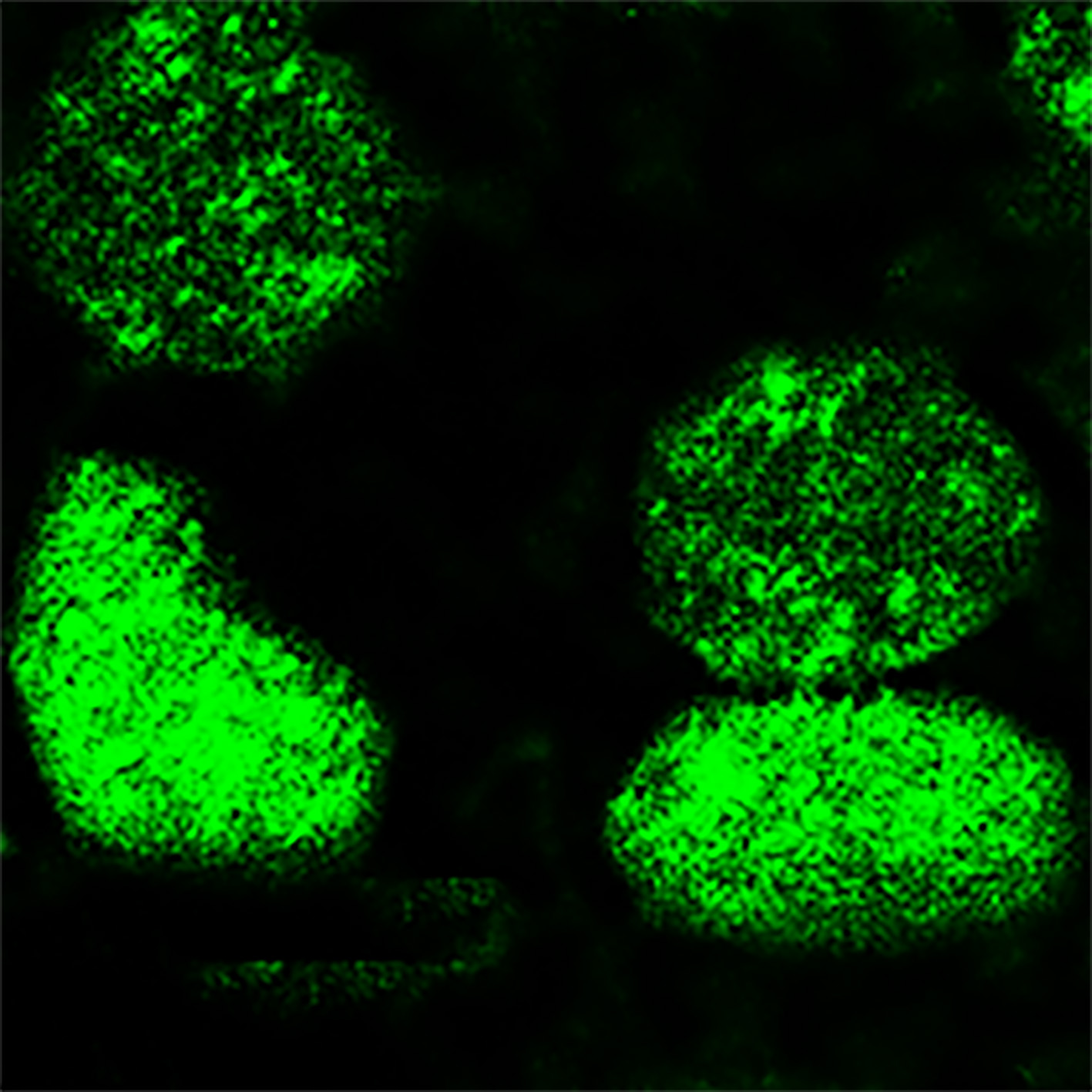

Supplement: Supplementary file 8 — Source data Fig. 1 [file 44318_2024_203_MOESM8_ESM.zip › Figure 1/Figure 1D/Sertoli cell-KDM2A.jpg]

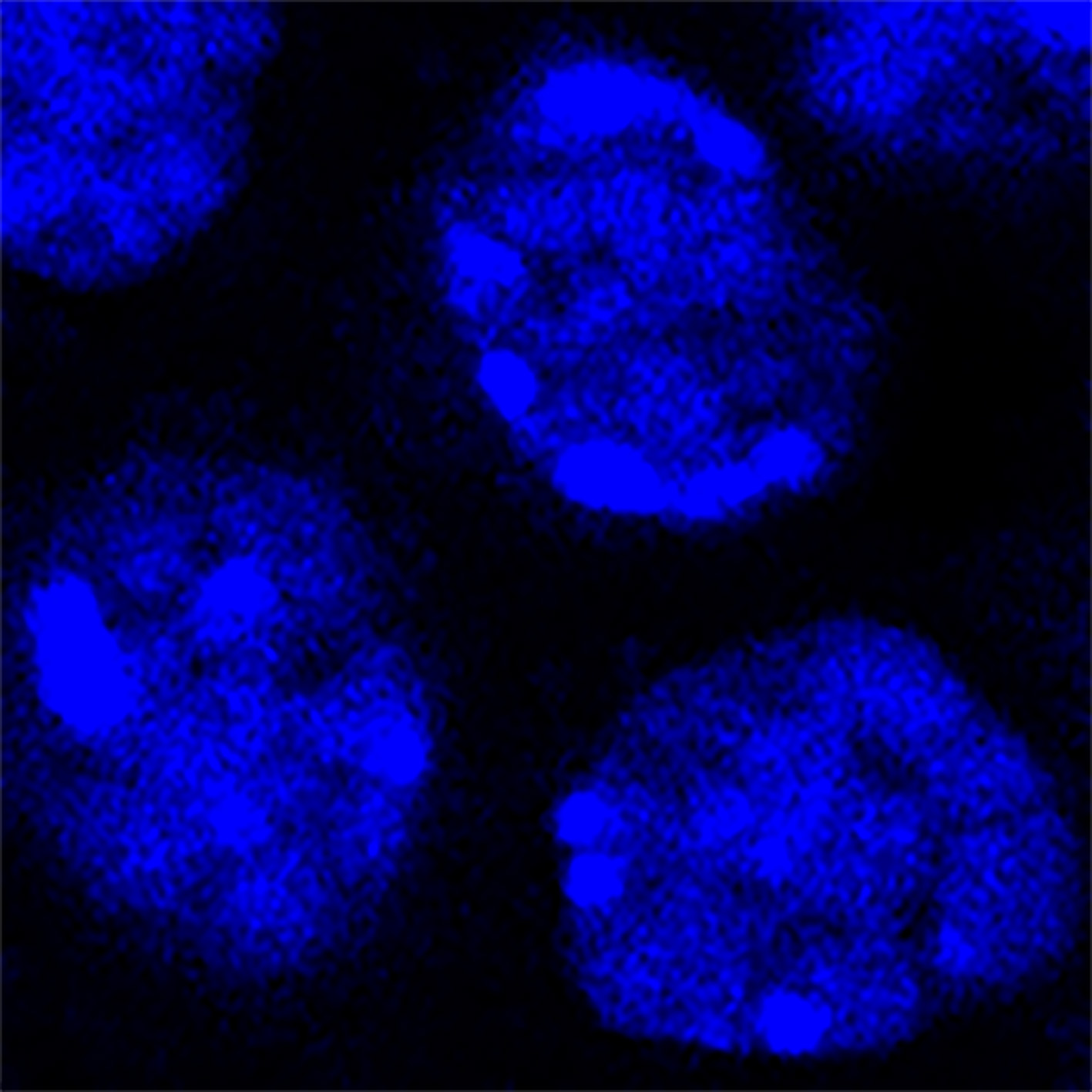

Supplement: Supplementary file 8 — Source data Fig. 1 [file 44318_2024_203_MOESM8_ESM.zip › Figure 1/Figure 1D/Pachytene (St. VII-VIII)- DAPI.jpg]

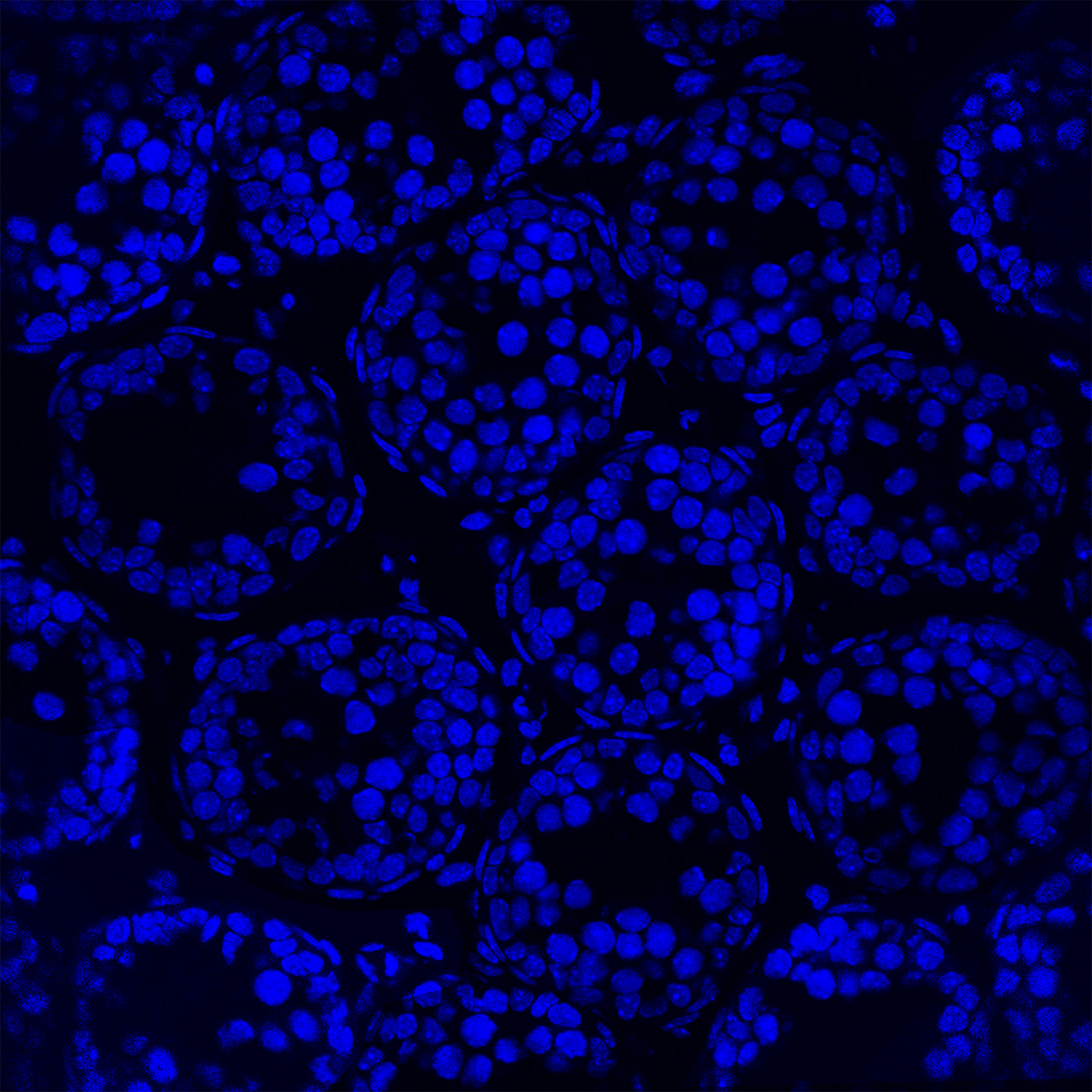

Supplement: Supplementary file 9 — Source data Fig. 2 [file 44318_2024_203_MOESM9_ESM.zip › Figure 2/Figure 2G/Ctrl-DAPI.jpg]

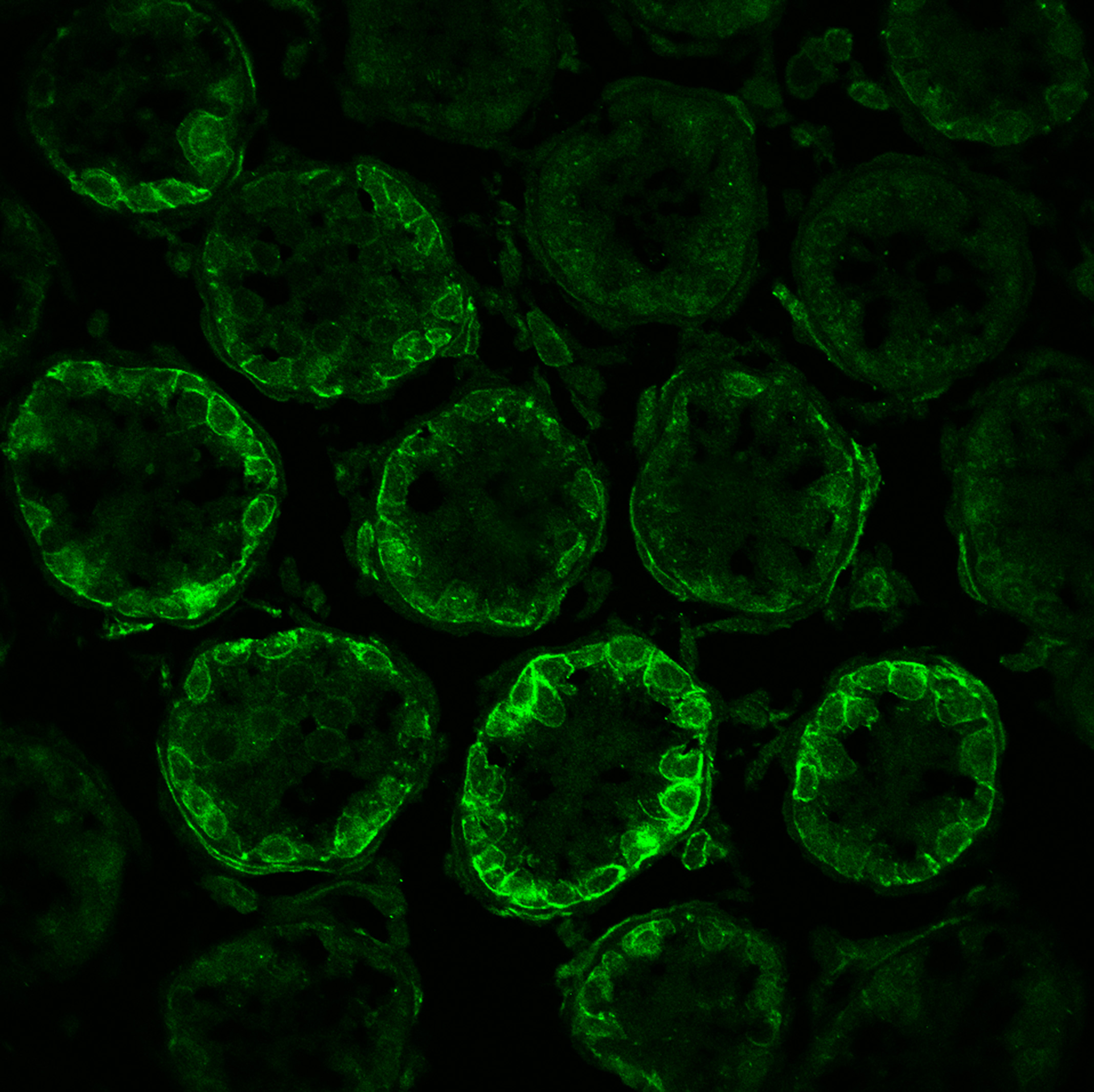

Supplement: Supplementary file 9 — Source data Fig. 2 [file 44318_2024_203_MOESM9_ESM.zip › Figure 2/Figure 2G/cKO-ckit.jpg]

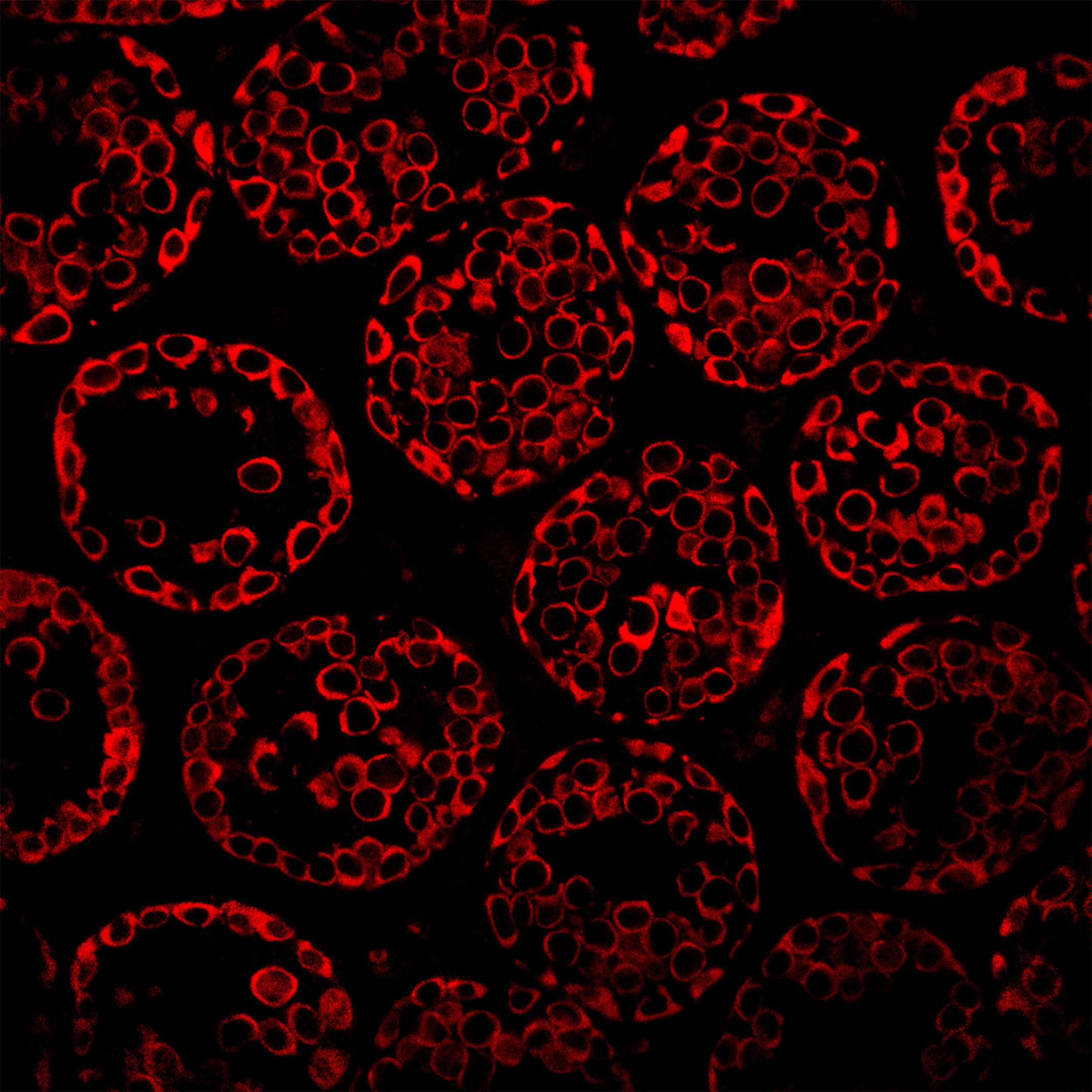

Supplement: Supplementary file 9 — Source data Fig. 2 [file 44318_2024_203_MOESM9_ESM.zip › Figure 2/Figure 2G/Ctrl-DDX4.jpg]

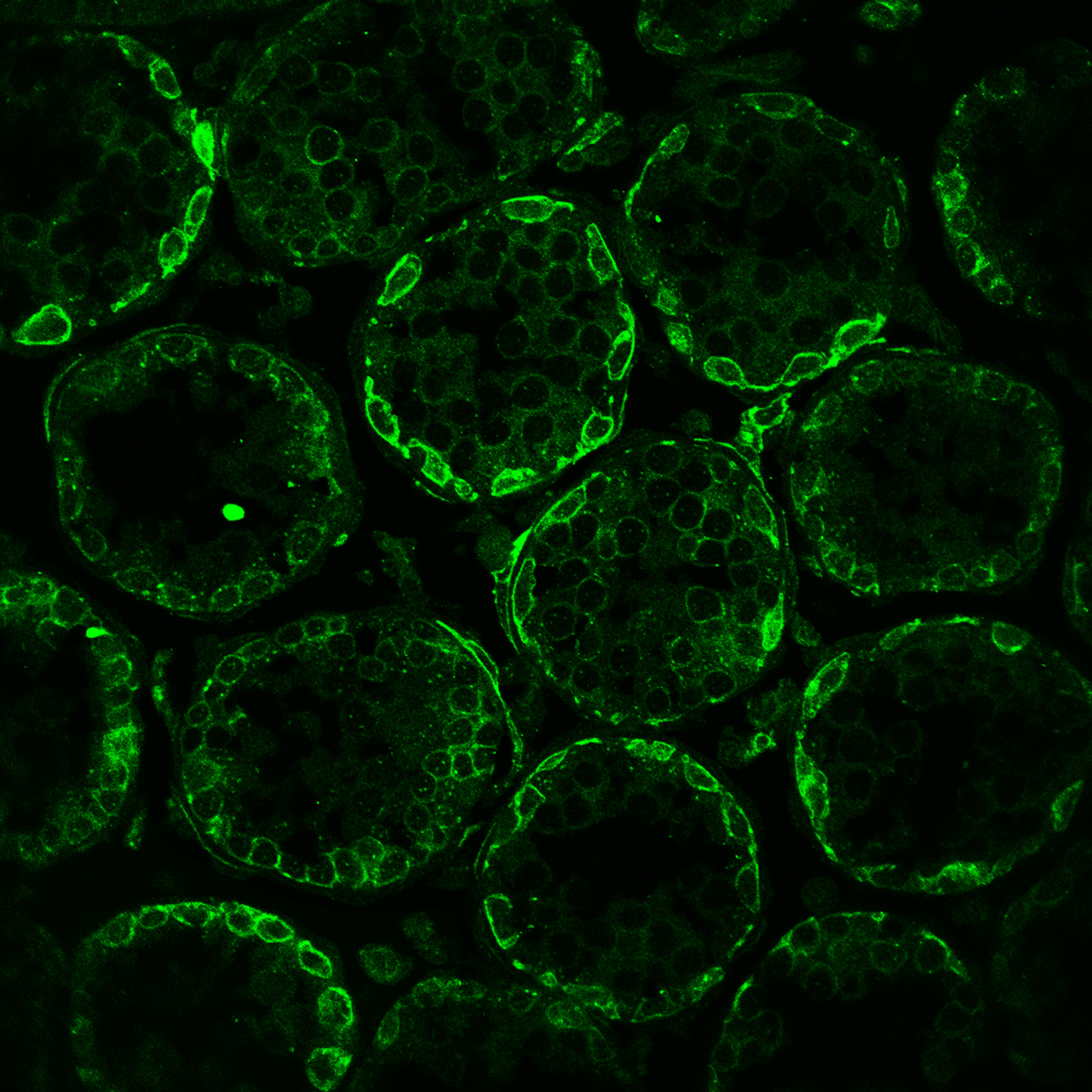

Supplement: Supplementary file 9 — Source data Fig. 2 [file 44318_2024_203_MOESM9_ESM.zip › Figure 2/Figure 2G/Ctrl-ckit.jpg]

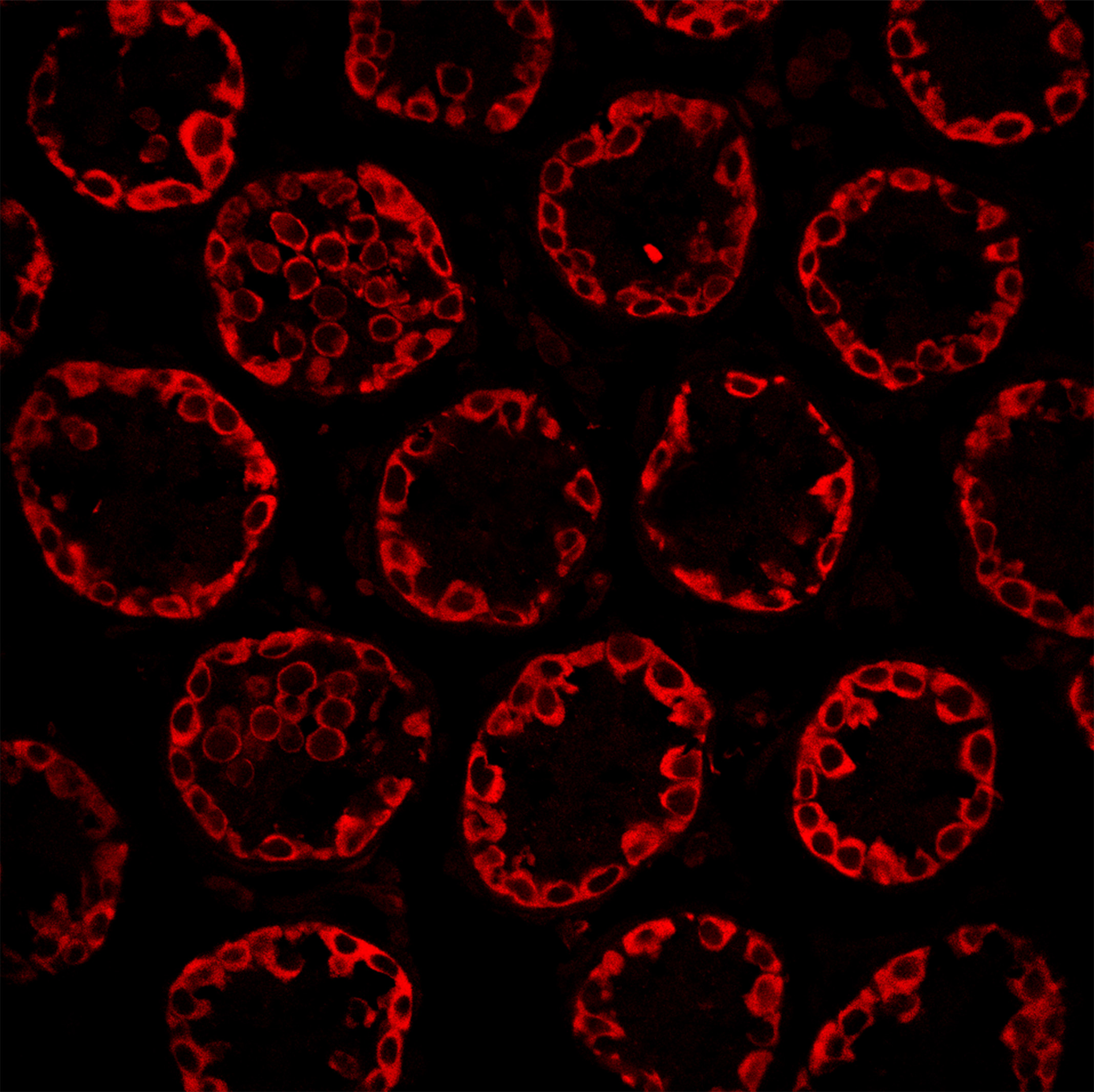

Supplement: Supplementary file 9 — Source data Fig. 2 [file 44318_2024_203_MOESM9_ESM.zip › Figure 2/Figure 2G/cKO-DDX4.jpg]

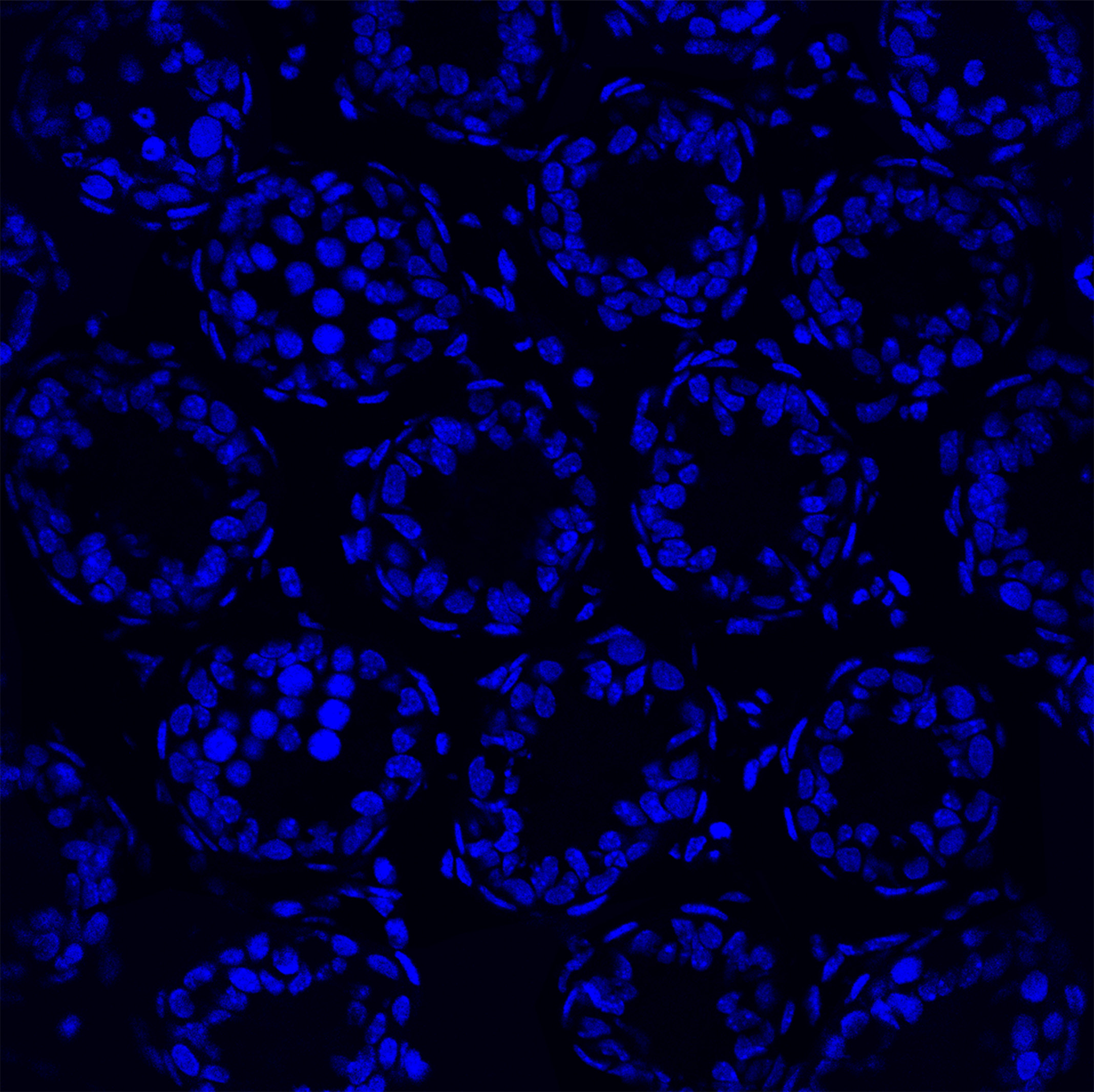

Supplement: Supplementary file 9 — Source data Fig. 2 [file 44318_2024_203_MOESM9_ESM.zip › Figure 2/Figure 2G/cKO-DAPI.jpg]

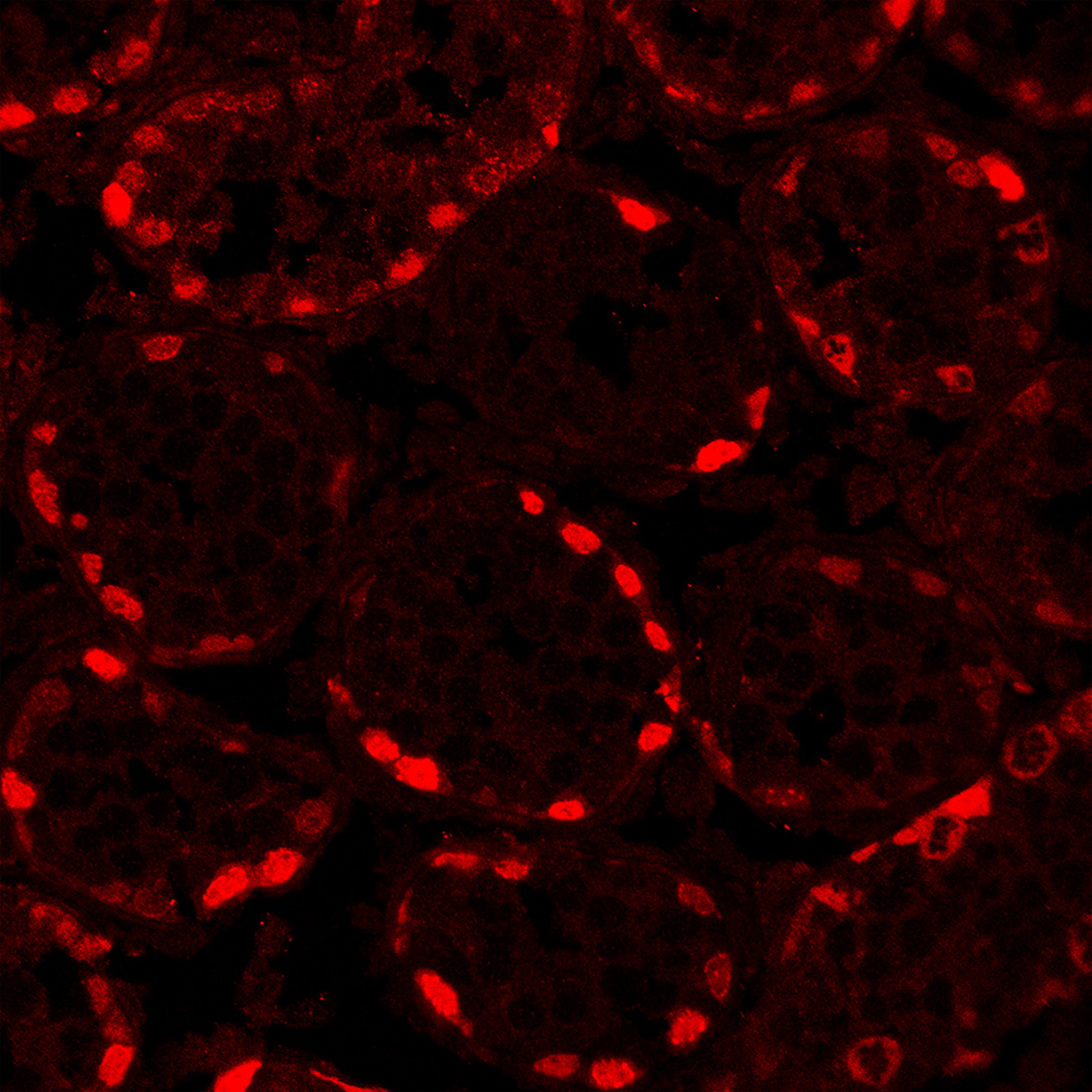

Supplement: Supplementary file 9 — Source data Fig. 2 [file 44318_2024_203_MOESM9_ESM.zip › Figure 2/Figure 2I/P10-Ctrl-PLZF.jpg]

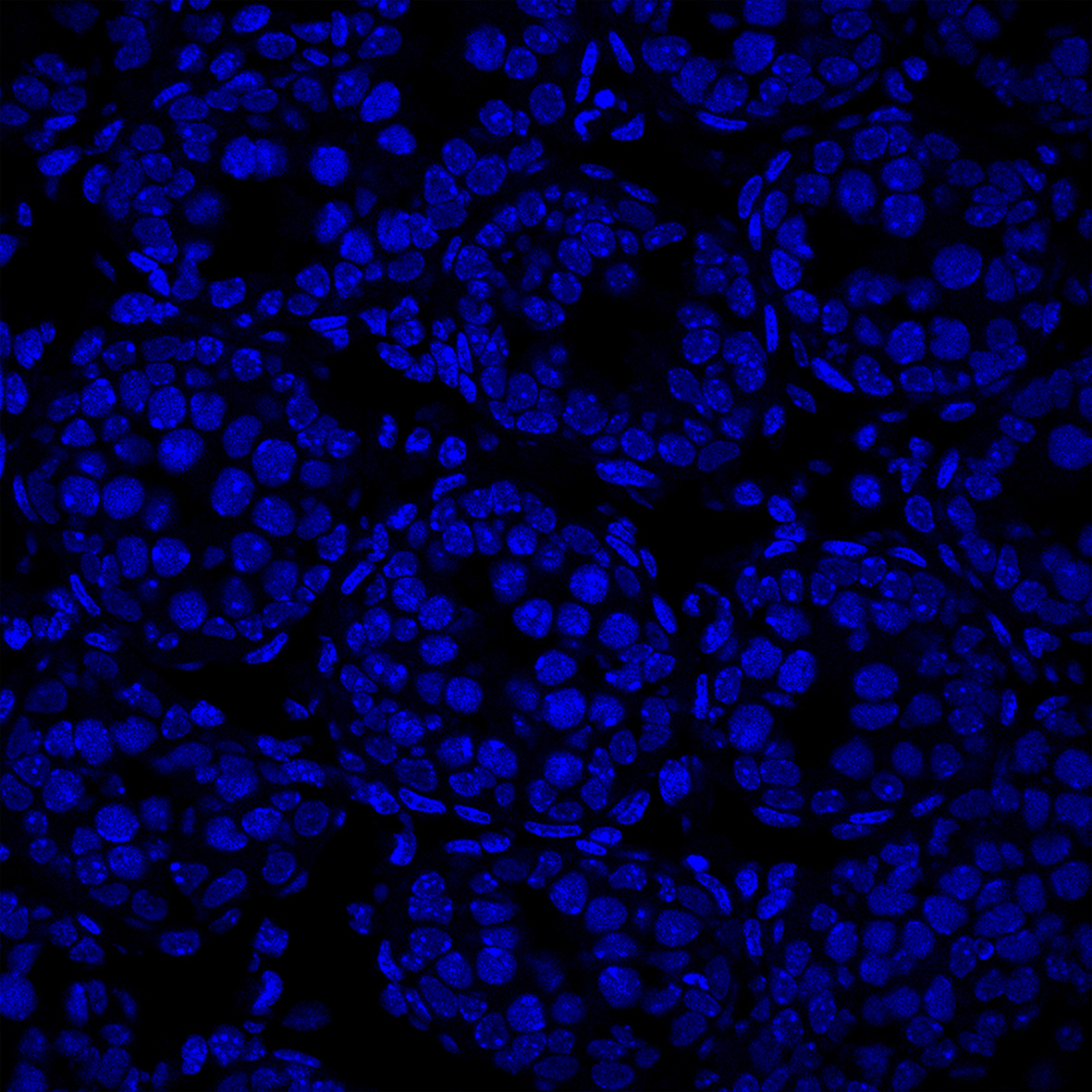

Supplement: Supplementary file 9 — Source data Fig. 2 [file 44318_2024_203_MOESM9_ESM.zip › Figure 2/Figure 2I/P10-Ctrl-DPAI.jpg]

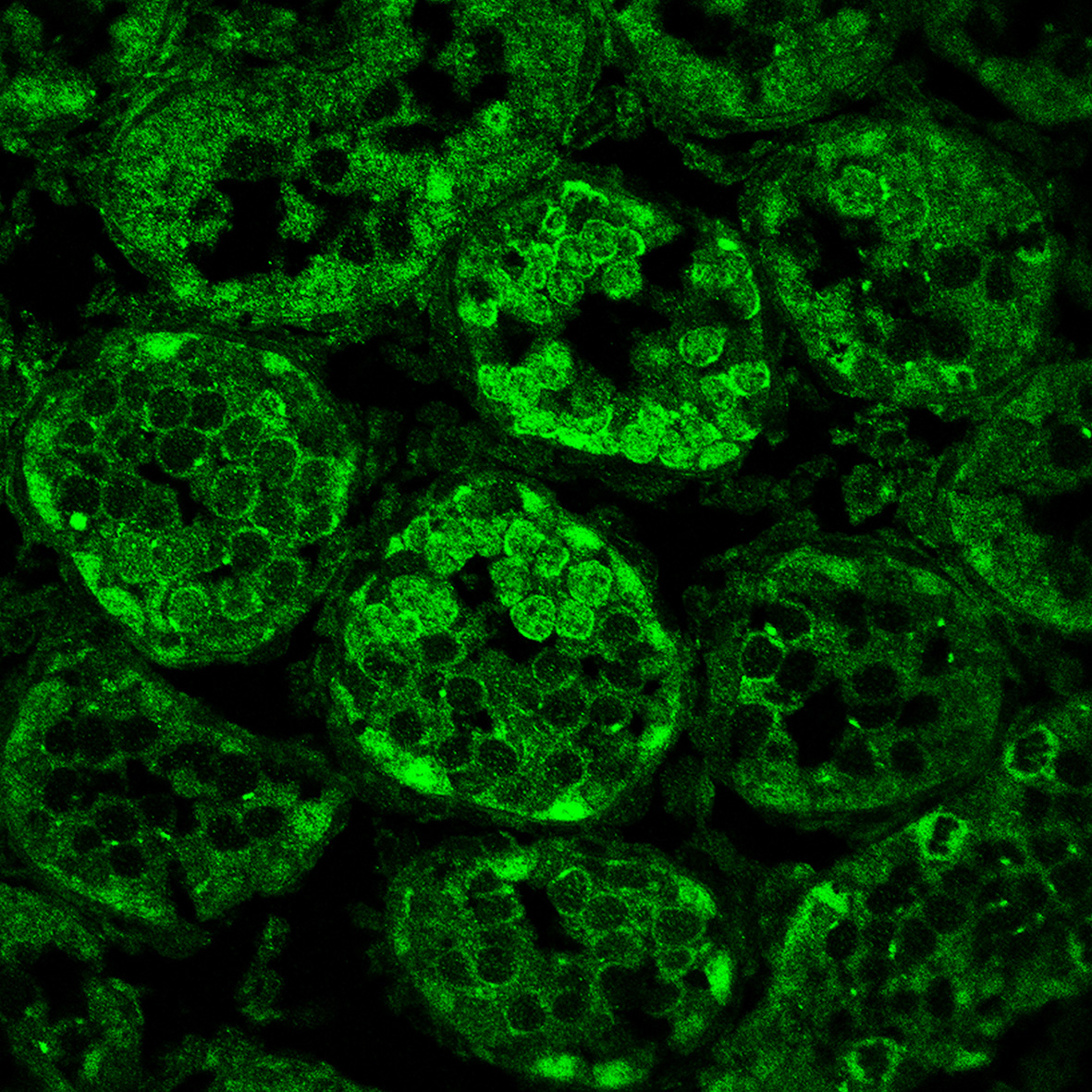

Supplement: Supplementary file 9 — Source data Fig. 2 [file 44318_2024_203_MOESM9_ESM.zip › Figure 2/Figure 2I/P10-Ctrl-STRA8.jpg]

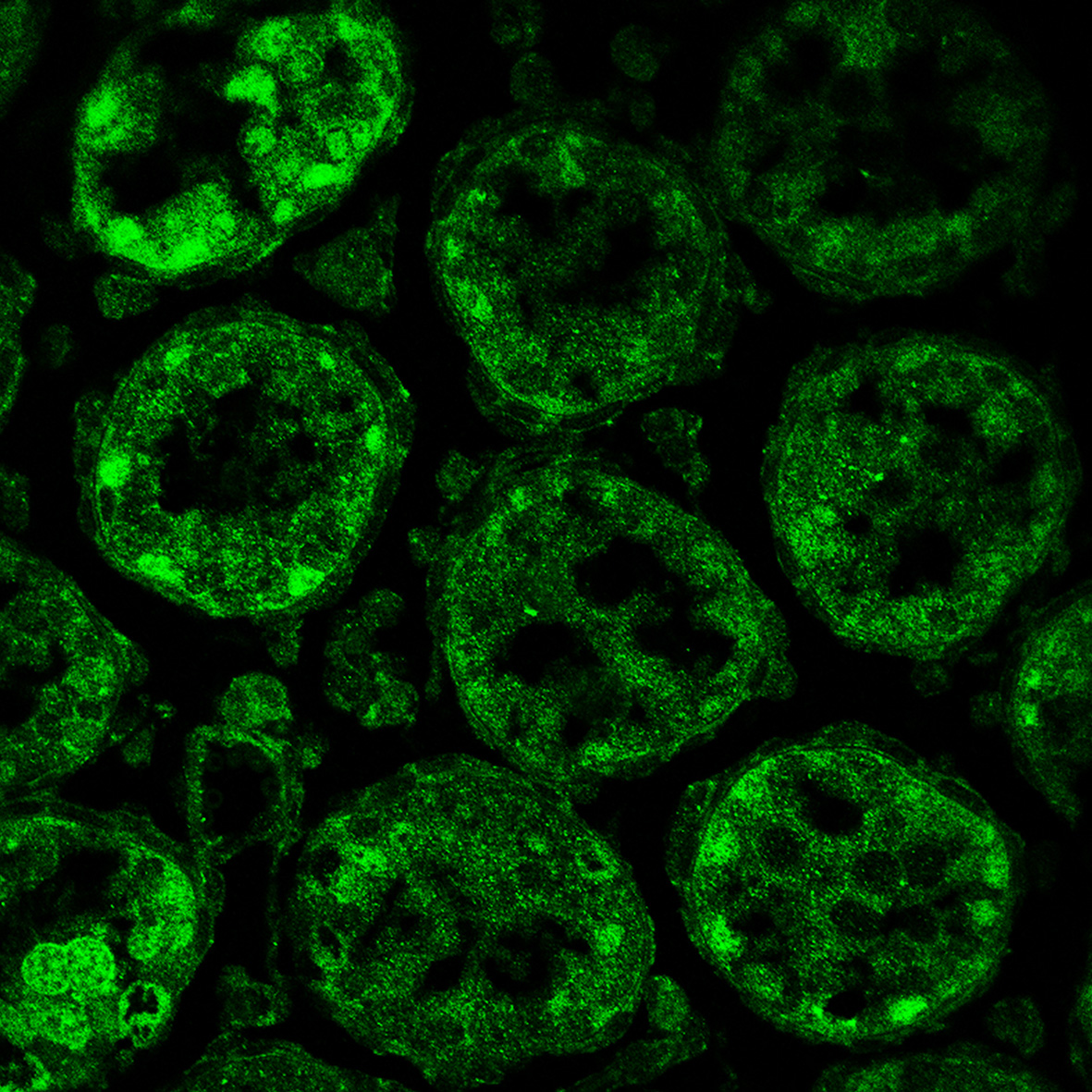

Supplement: Supplementary file 9 — Source data Fig. 2 [file 44318_2024_203_MOESM9_ESM.zip › Figure 2/Figure 2I/P10-cKO-STRA8.jpg]

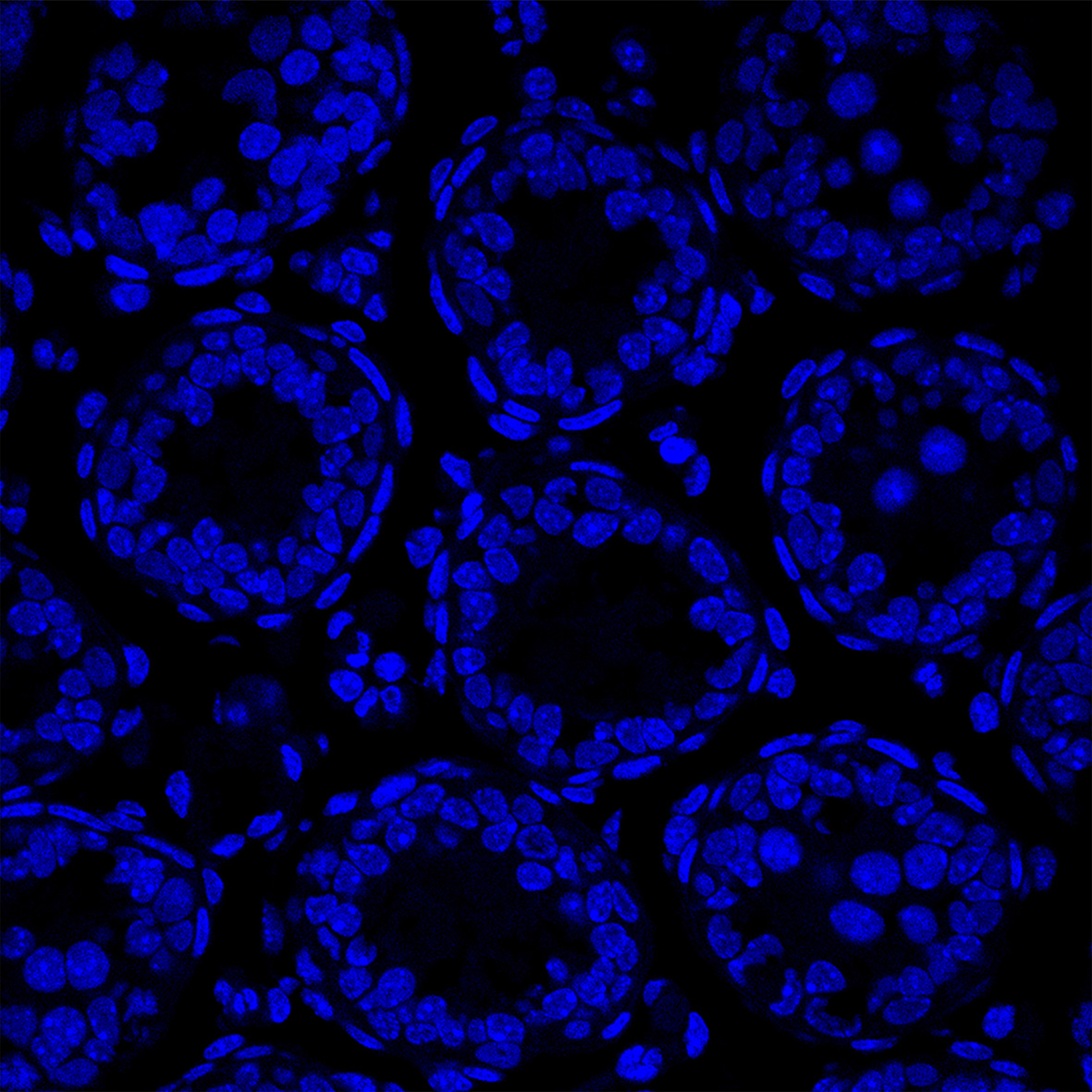

Supplement: Supplementary file 9 — Source data Fig. 2 [file 44318_2024_203_MOESM9_ESM.zip › Figure 2/Figure 2I/P10-cKO-DAPI.jpg]

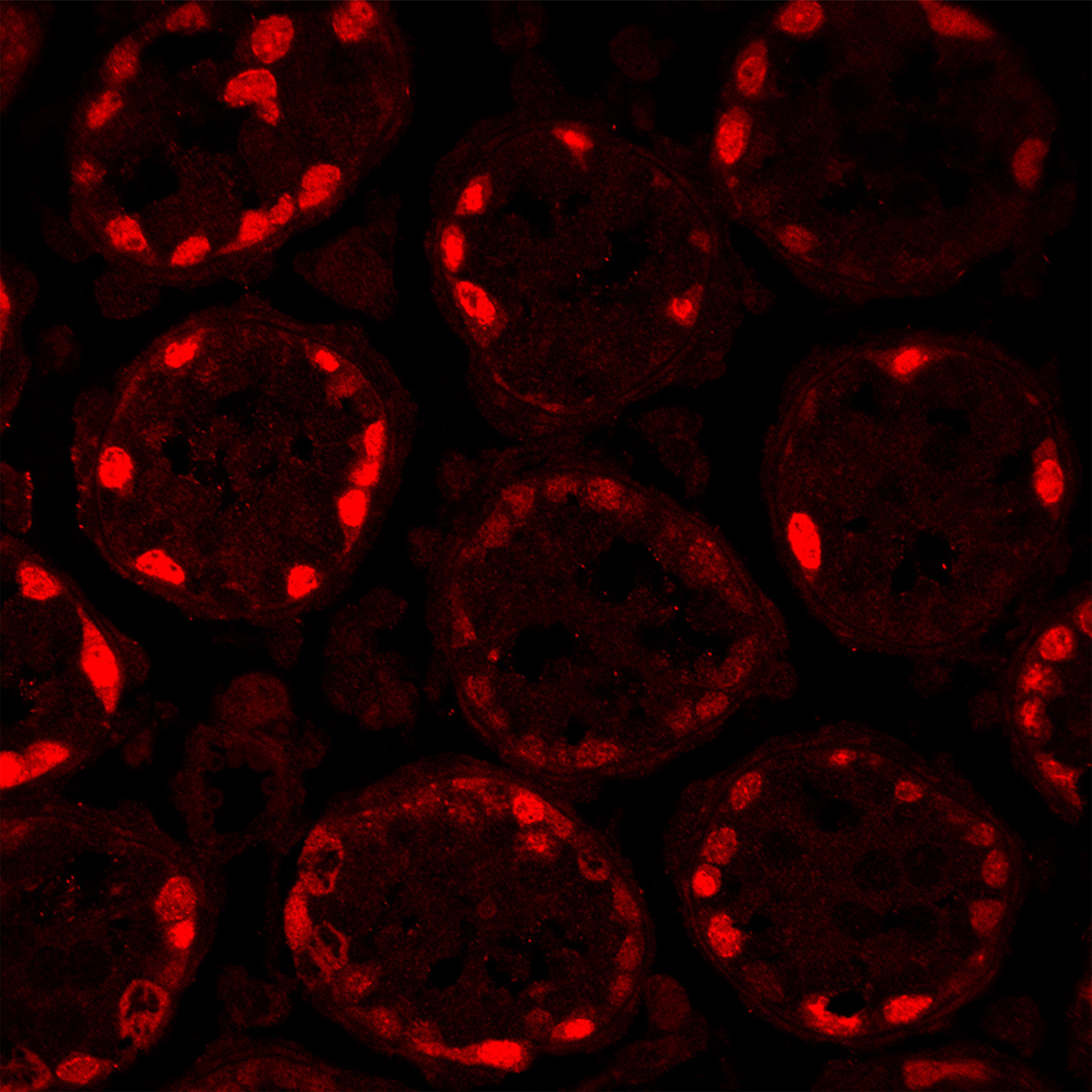

Supplement: Supplementary file 9 — Source data Fig. 2 [file 44318_2024_203_MOESM9_ESM.zip › Figure 2/Figure 2I/P10-cKO-PLZF.jpg]

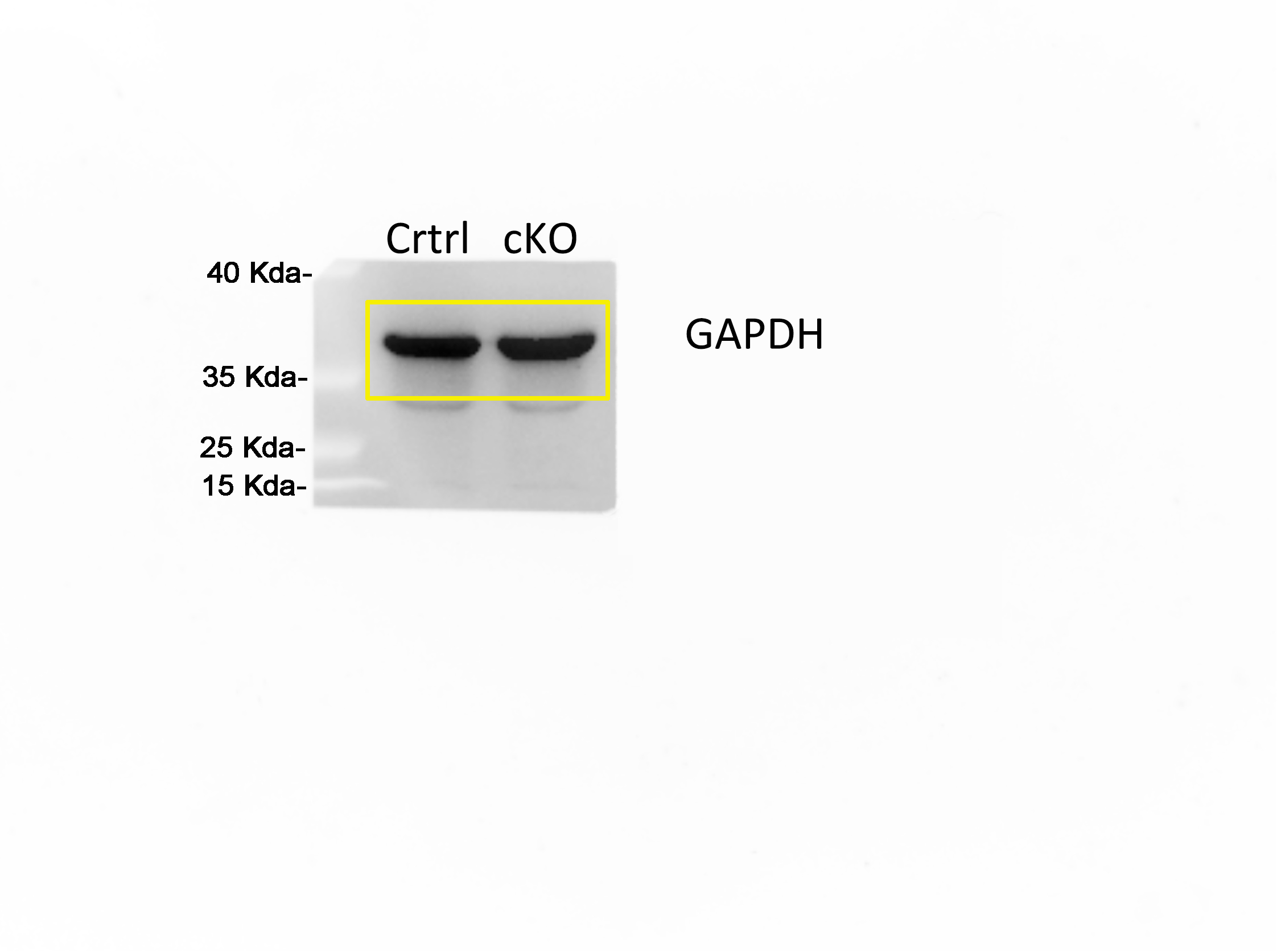

Supplement: Supplementary file 9 — Source data Fig. 2 [file 44318_2024_203_MOESM9_ESM.zip › Figure 2/Figure 2A/WB-GAPDH.jpg]

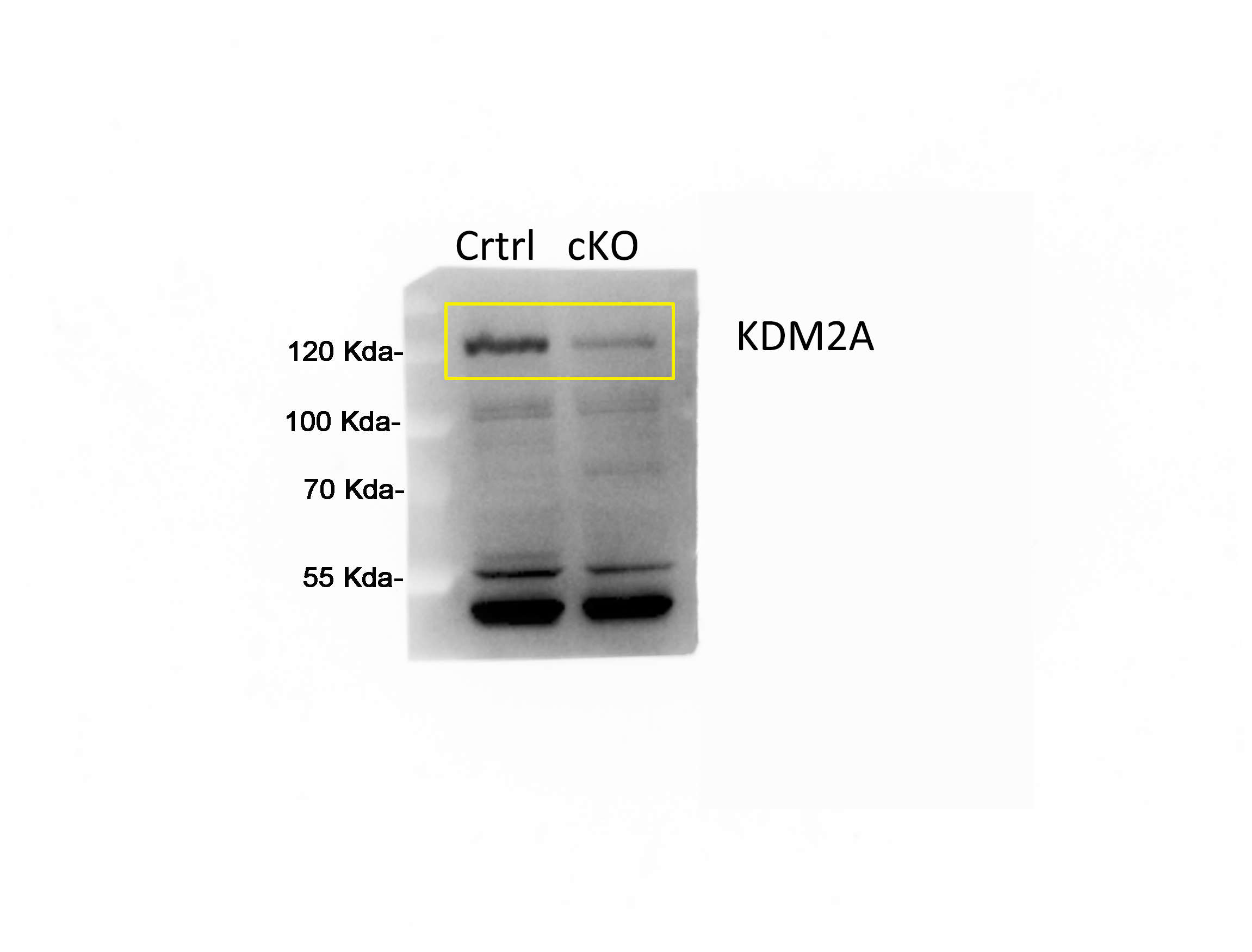

Supplement: Supplementary file 9 — Source data Fig. 2 [file 44318_2024_203_MOESM9_ESM.zip › Figure 2/Figure 2A/WB-KDM2A.jpg]

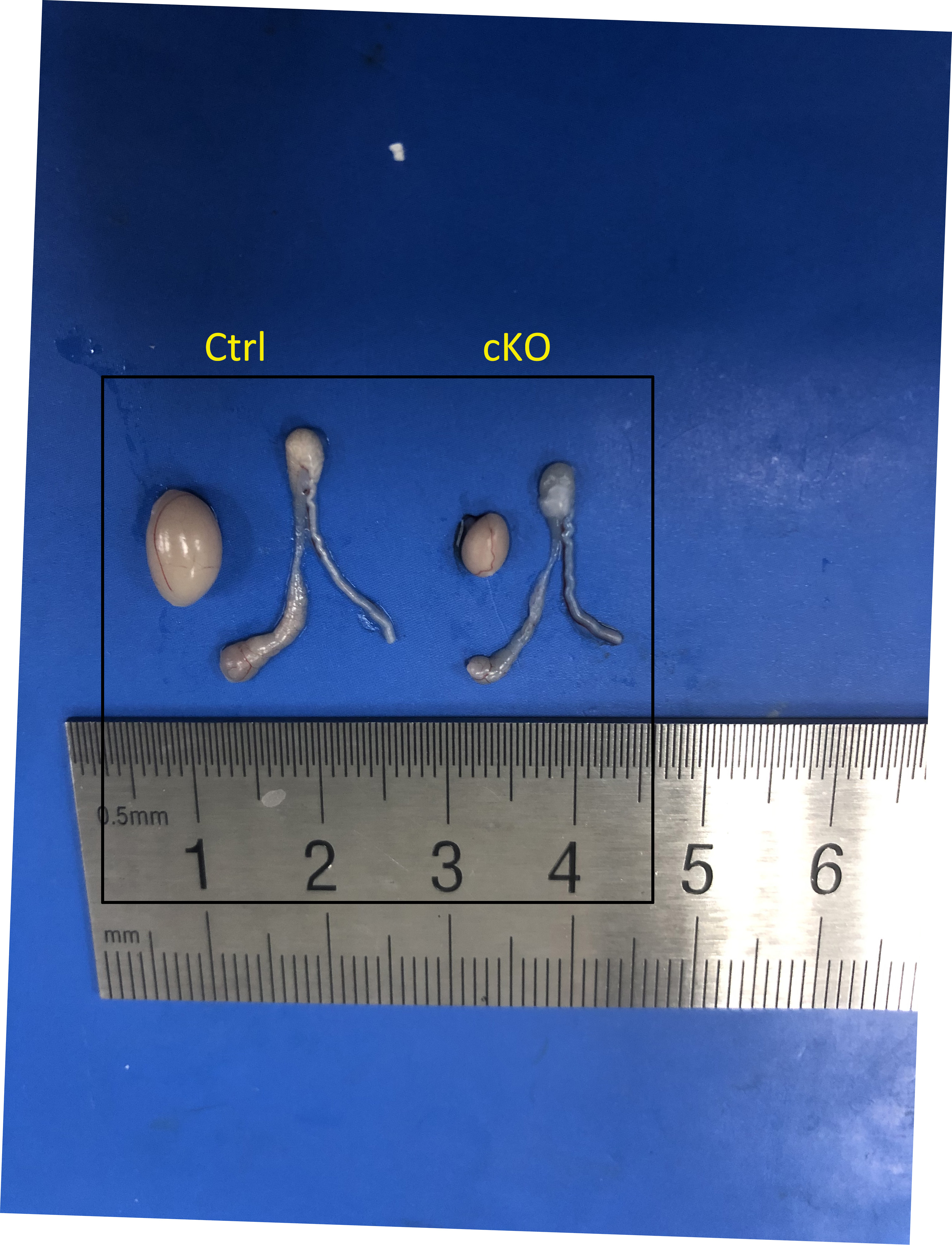

Supplement: Supplementary file 9 — Source data Fig. 2 [file 44318_2024_203_MOESM9_ESM.zip › Figure 2/Figure 2D/Testicular morphology.jpg]

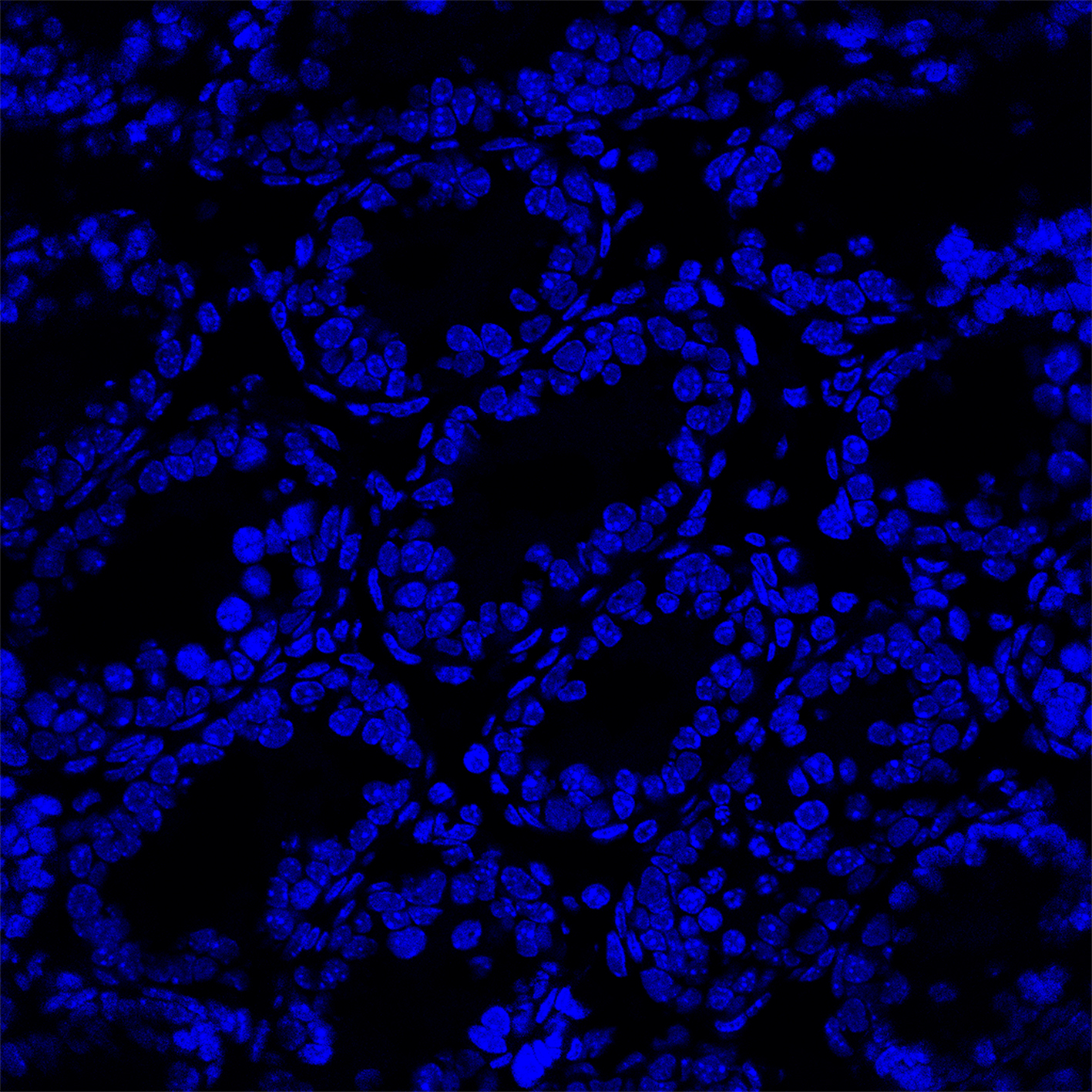

Supplement: Supplementary file 9 — Source data Fig. 2 [file 44318_2024_203_MOESM9_ESM.zip › Figure 2/Figure 2K/P14-cKO-DAPI.jpg]

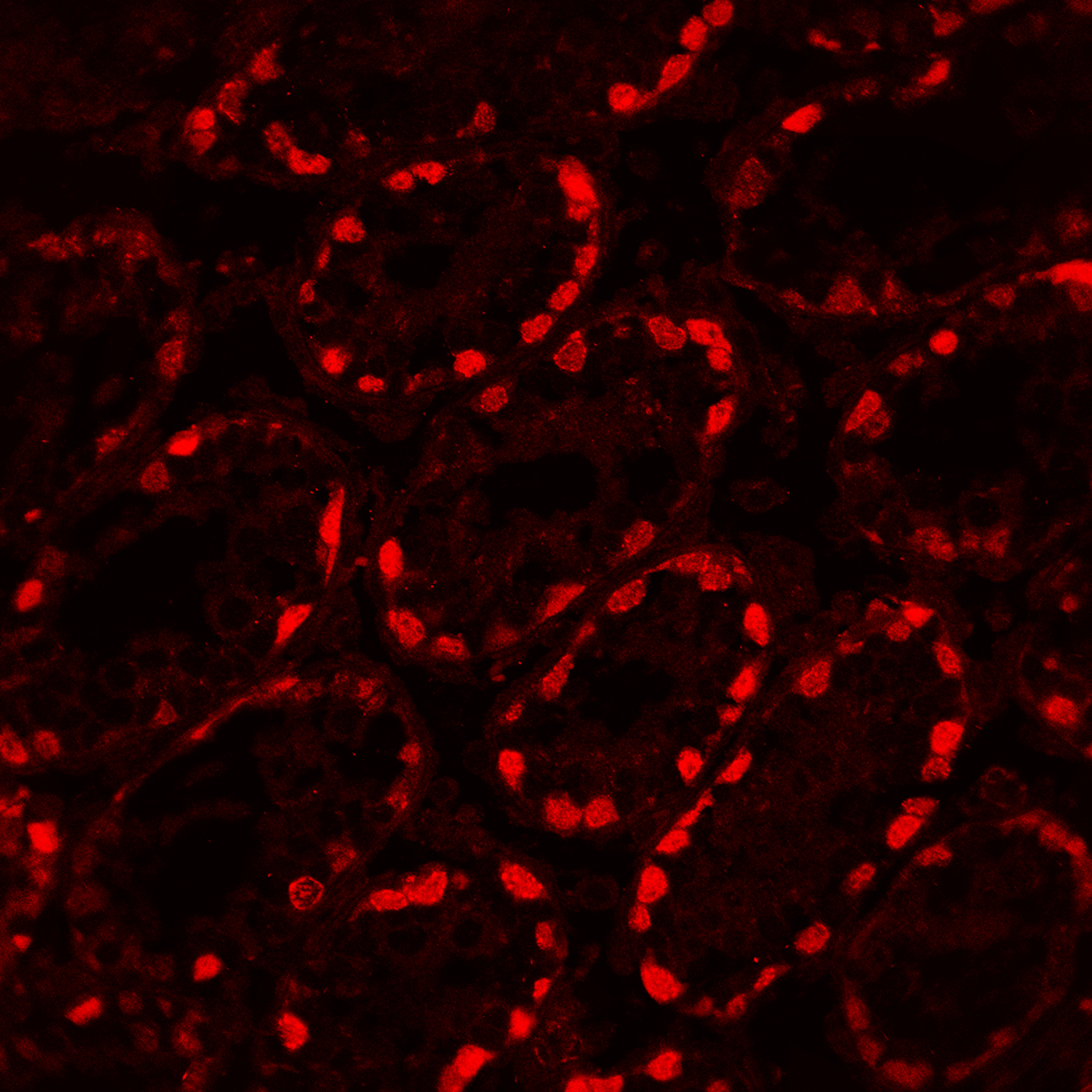

Supplement: Supplementary file 9 — Source data Fig. 2 [file 44318_2024_203_MOESM9_ESM.zip › Figure 2/Figure 2K/P14-cKO-PLZF.jpg]

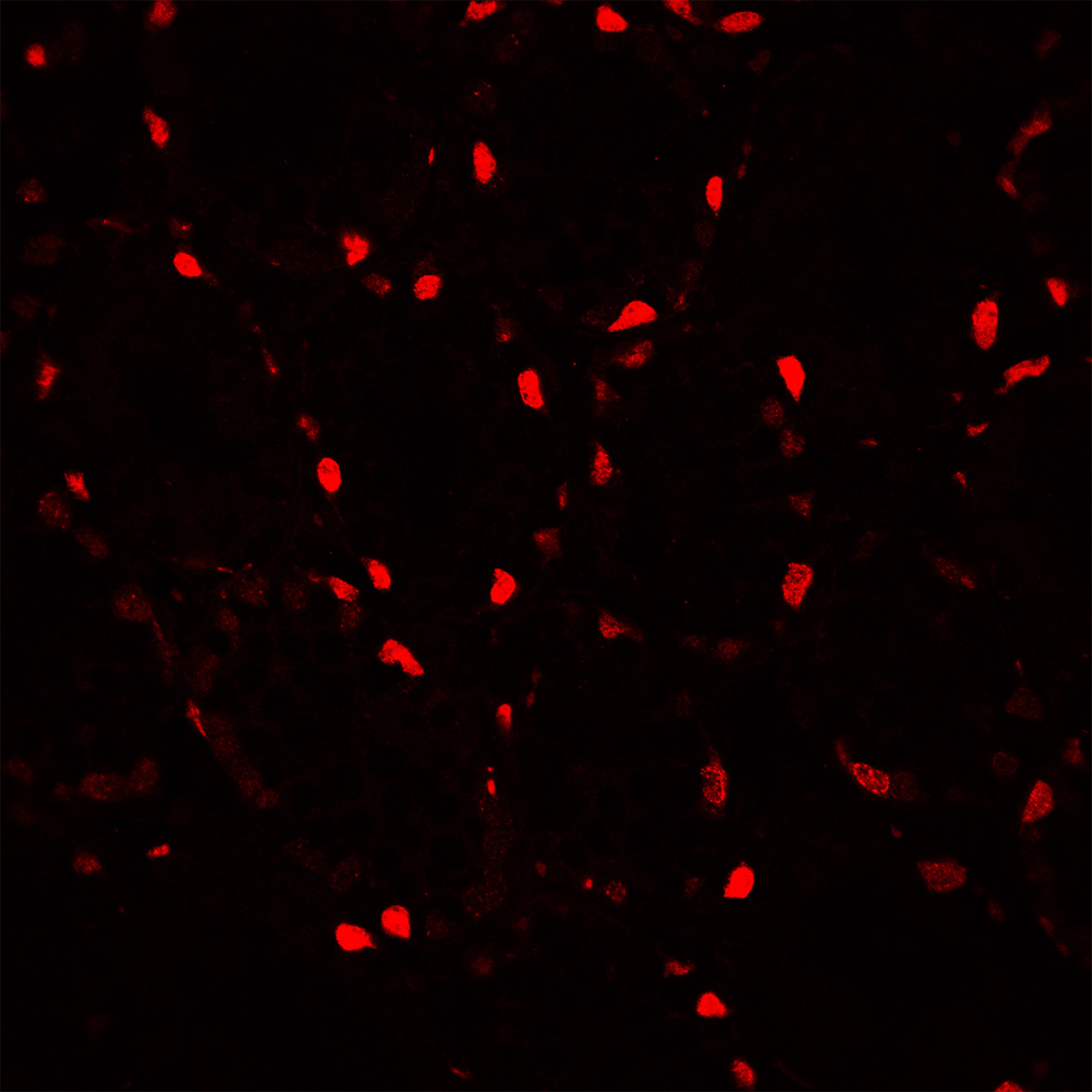

Supplement: Supplementary file 9 — Source data Fig. 2 [file 44318_2024_203_MOESM9_ESM.zip › Figure 2/Figure 2K/P14-Ctrl-PLZF.jpg]

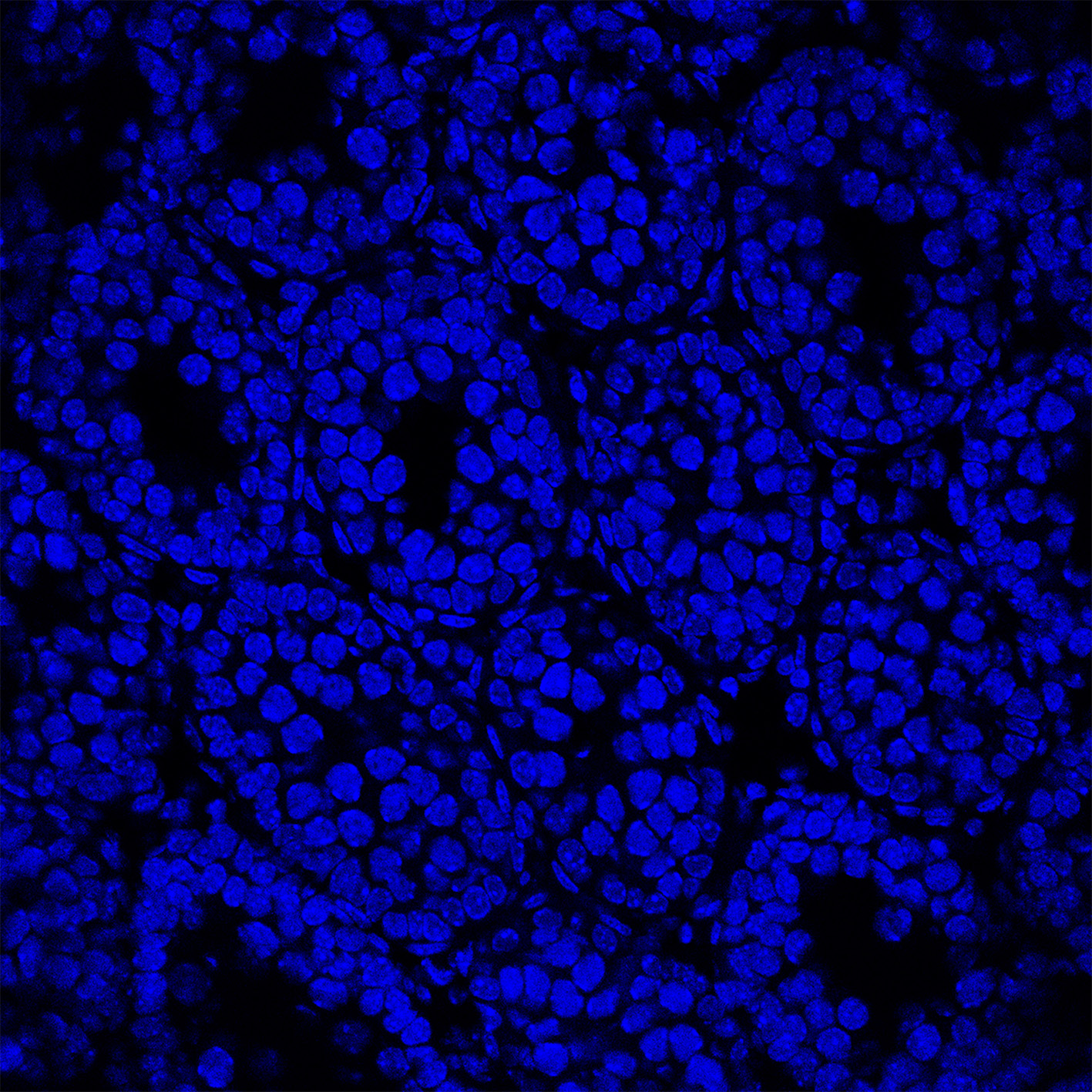

Supplement: Supplementary file 9 — Source data Fig. 2 [file 44318_2024_203_MOESM9_ESM.zip › Figure 2/Figure 2K/P14-Ctrl-DAPI.jpg]

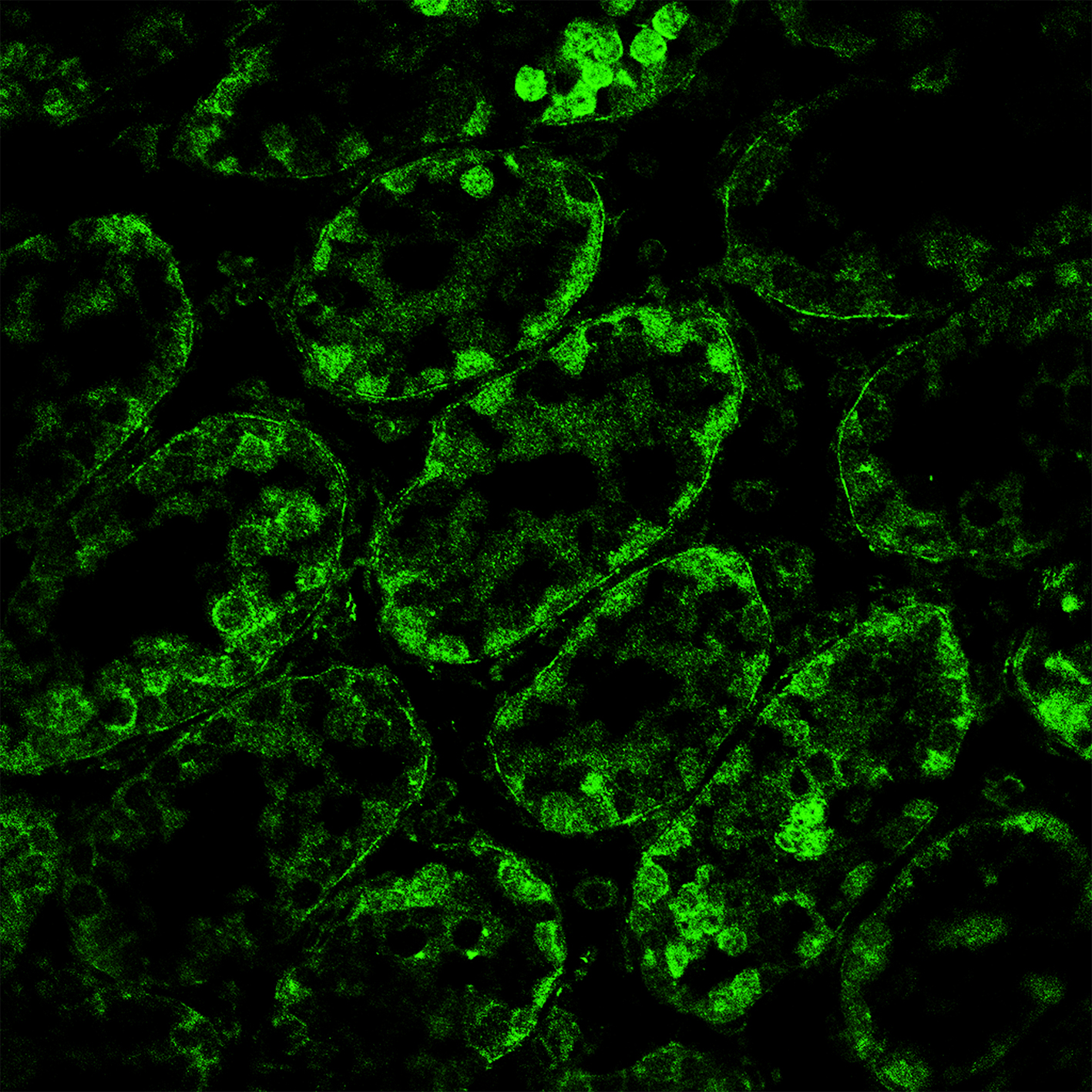

Supplement: Supplementary file 9 — Source data Fig. 2 [file 44318_2024_203_MOESM9_ESM.zip › Figure 2/Figure 2K/P14-cKO-STRA8.jpg]

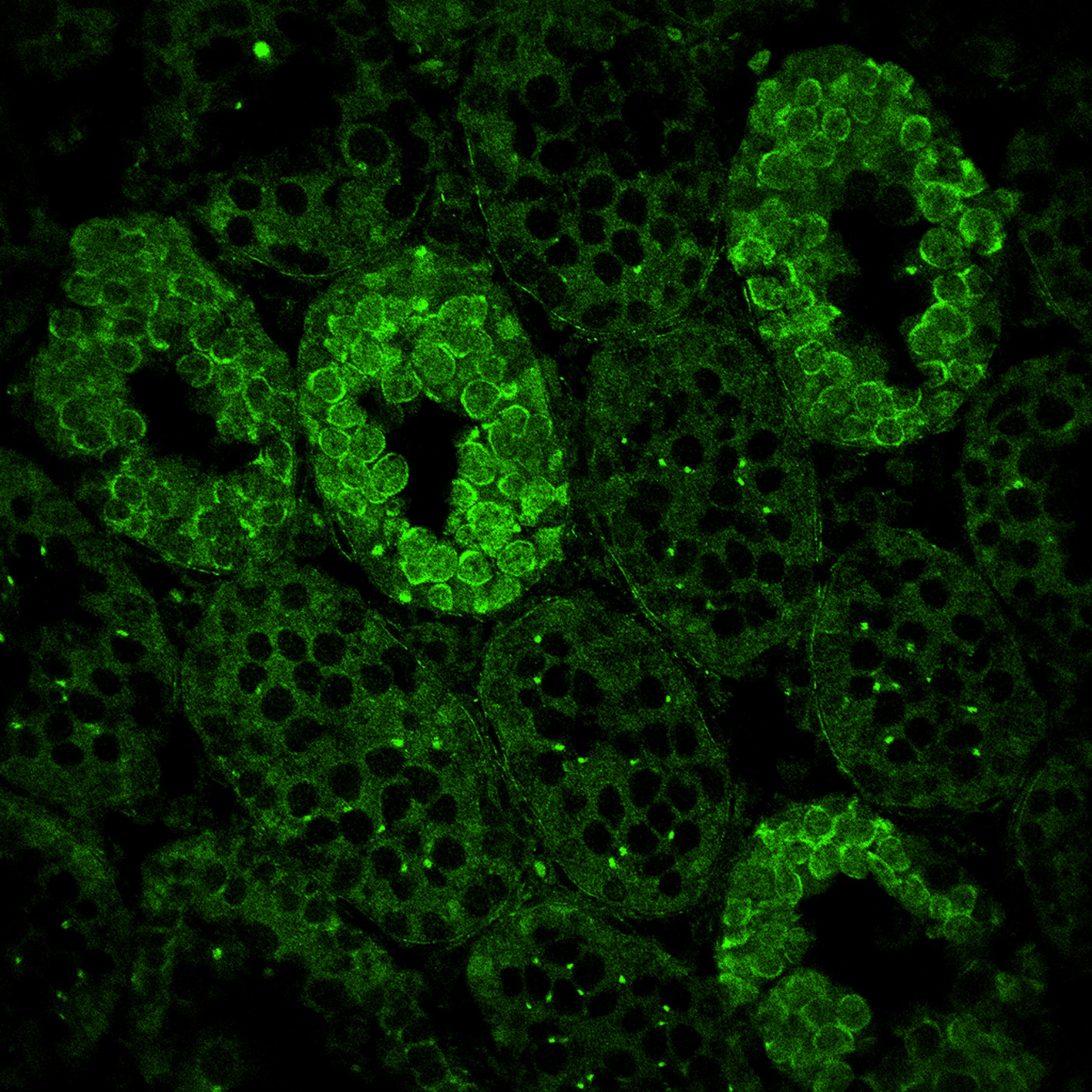

Supplement: Supplementary file 9 — Source data Fig. 2 [file 44318_2024_203_MOESM9_ESM.zip › Figure 2/Figure 2K/P14-Ctrl-STRA8.jpg]

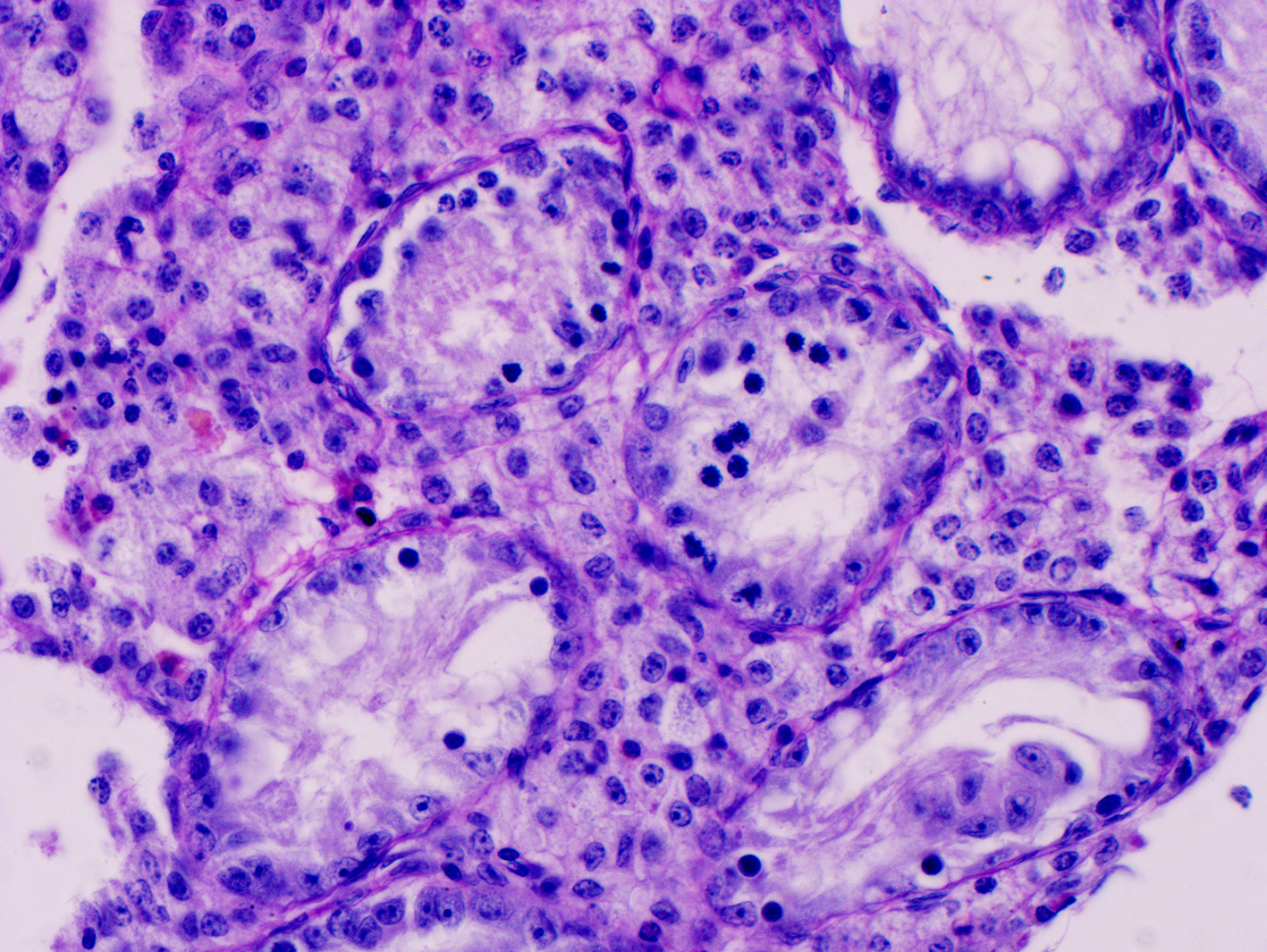

Supplement: Supplementary file 9 — Source data Fig. 2 [file 44318_2024_203_MOESM9_ESM.zip › Figure 2/Figure 2E/cKO-testis.jpg]

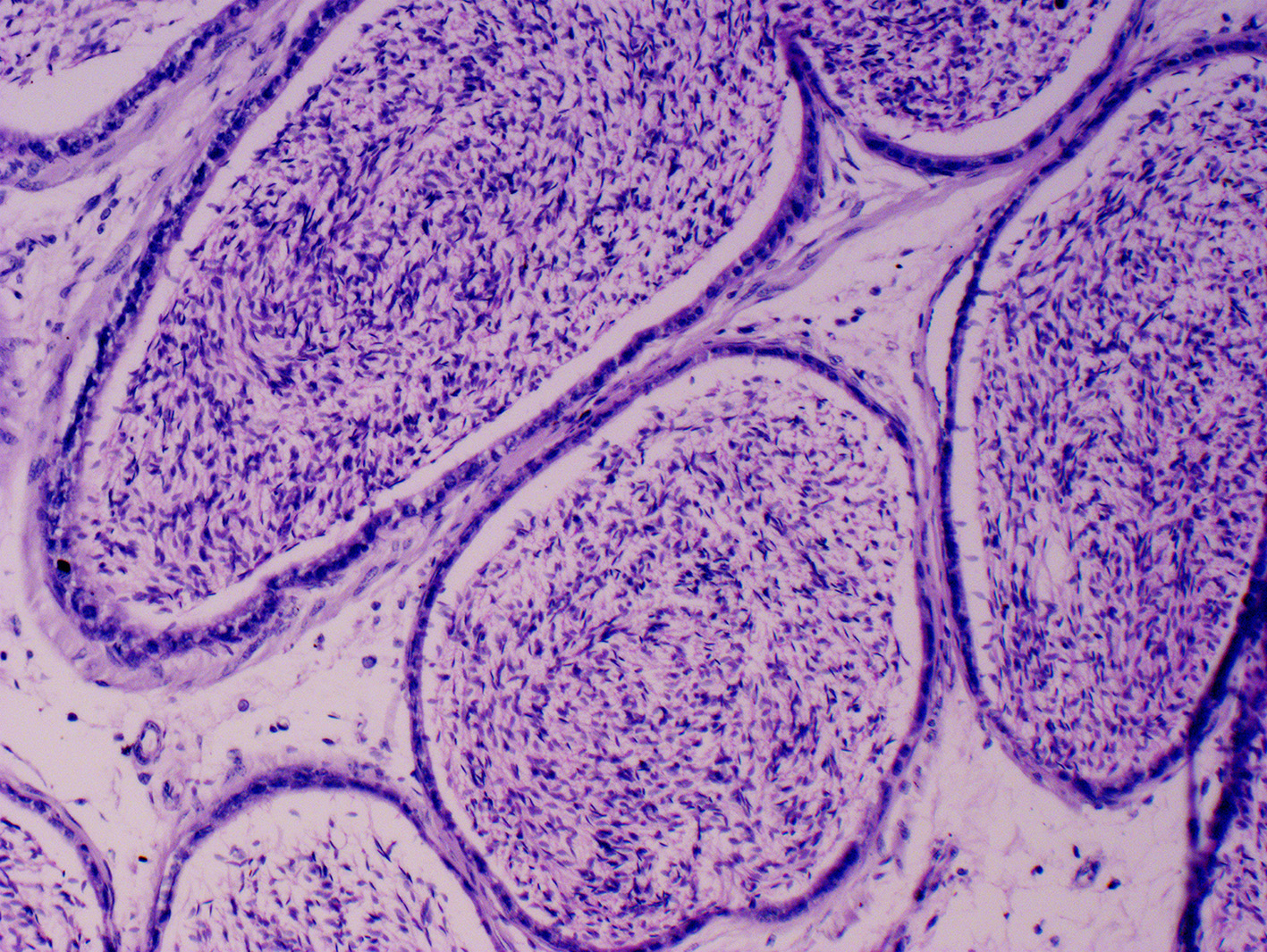

Supplement: Supplementary file 9 — Source data Fig. 2 [file 44318_2024_203_MOESM9_ESM.zip › Figure 2/Figure 2E/Ctrl-epididymis.jpg]

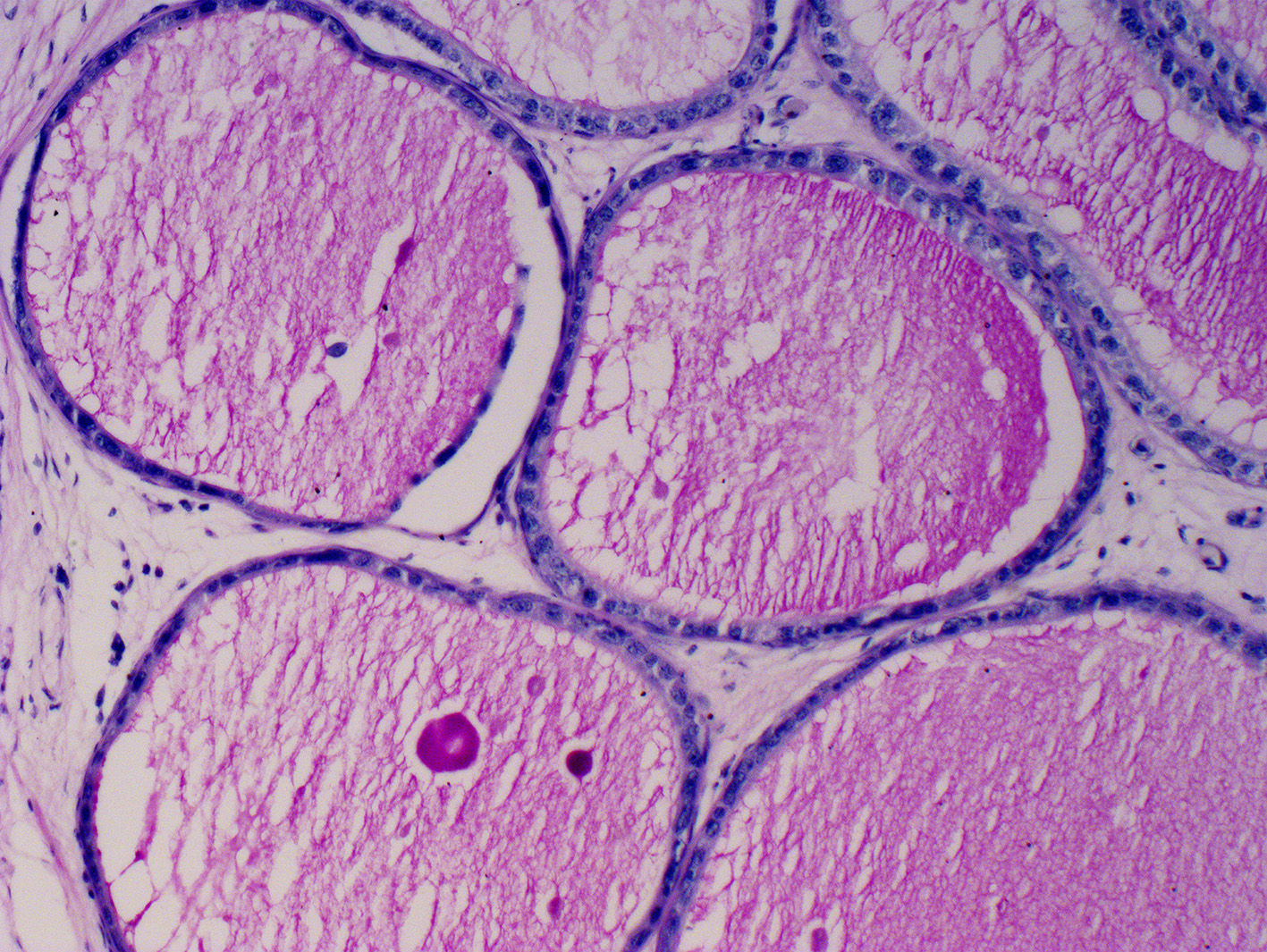

Supplement: Supplementary file 9 — Source data Fig. 2 [file 44318_2024_203_MOESM9_ESM.zip › Figure 2/Figure 2E/cKO-epididymis.jpg]

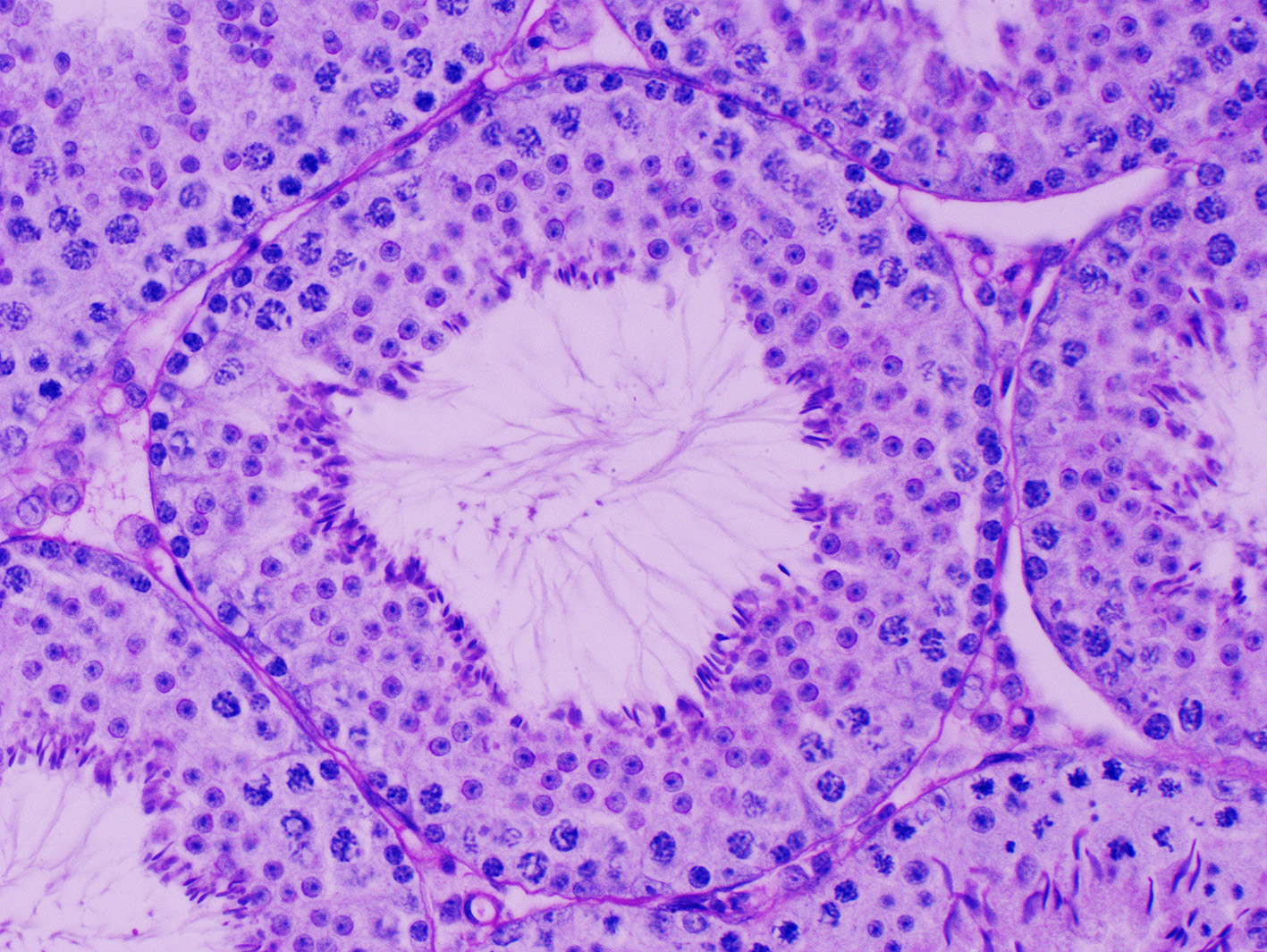

Supplement: Supplementary file 9 — Source data Fig. 2 [file 44318_2024_203_MOESM9_ESM.zip › Figure 2/Figure 2E/Ctrl-testis.jpg]

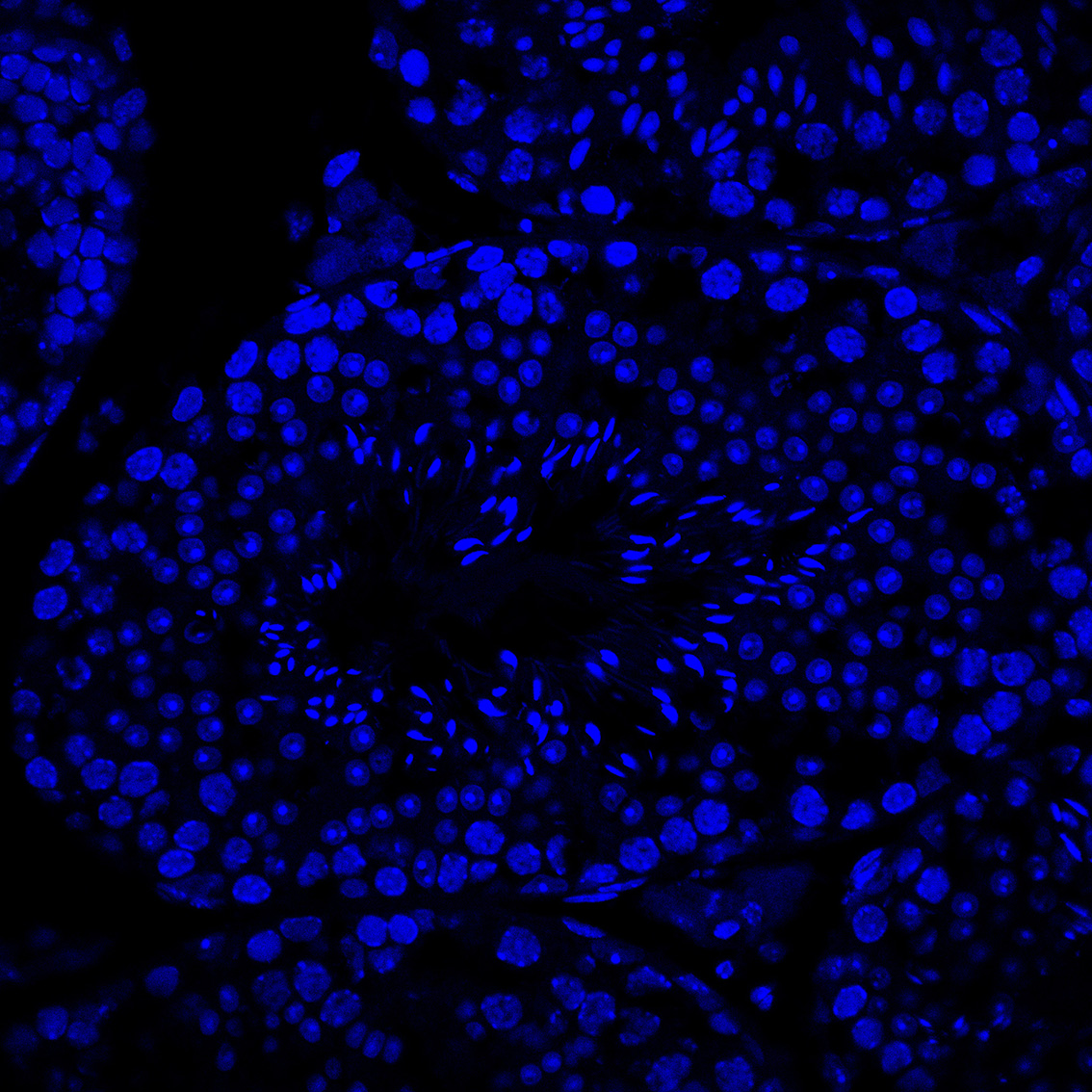

Supplement: Supplementary file 9 — Source data Fig. 2 [file 44318_2024_203_MOESM9_ESM.zip › Figure 2/Figure 2B/Ctrl-DAPI.jpg]

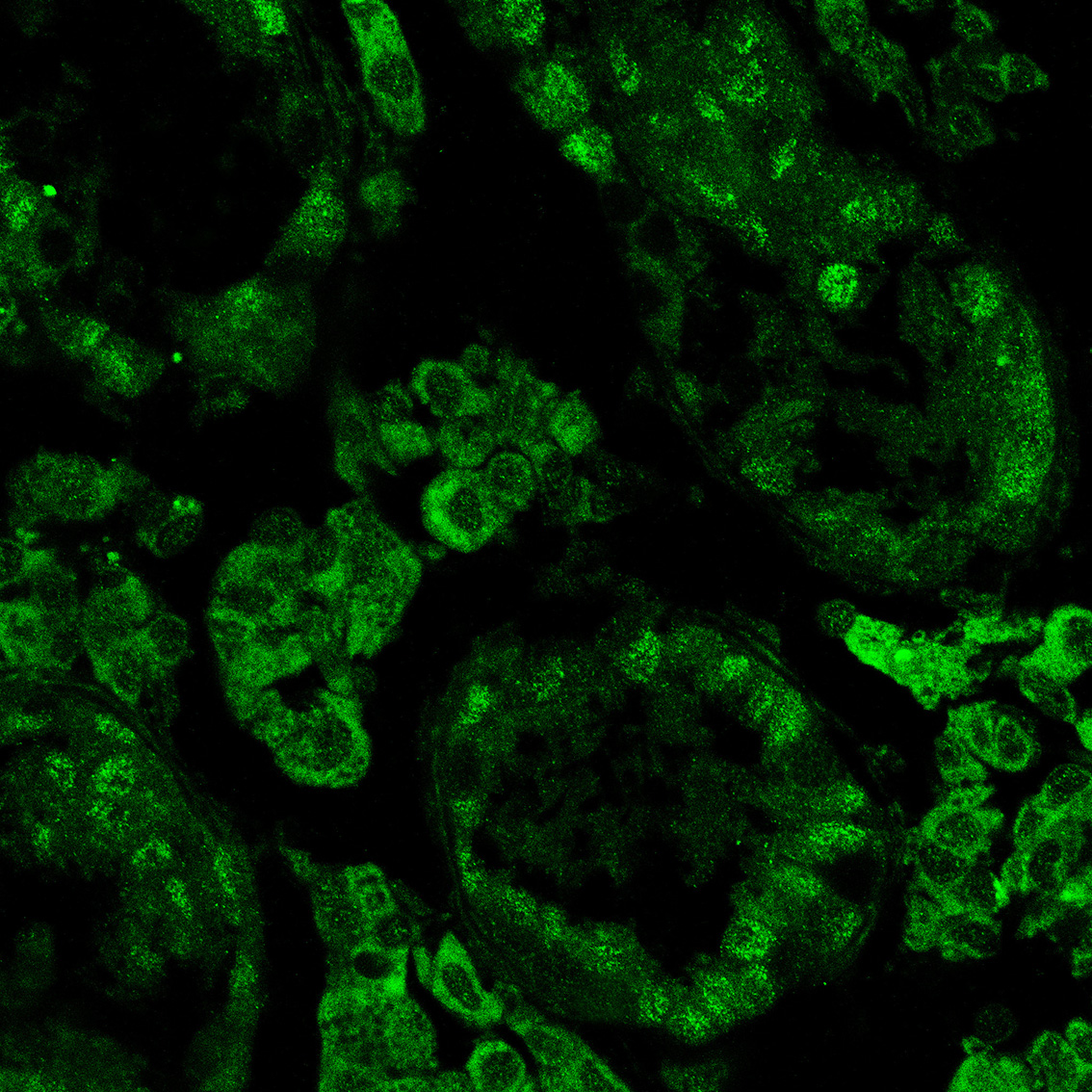

Supplement: Supplementary file 9 — Source data Fig. 2 [file 44318_2024_203_MOESM9_ESM.zip › Figure 2/Figure 2B/cKO-KDM2A.jpg]

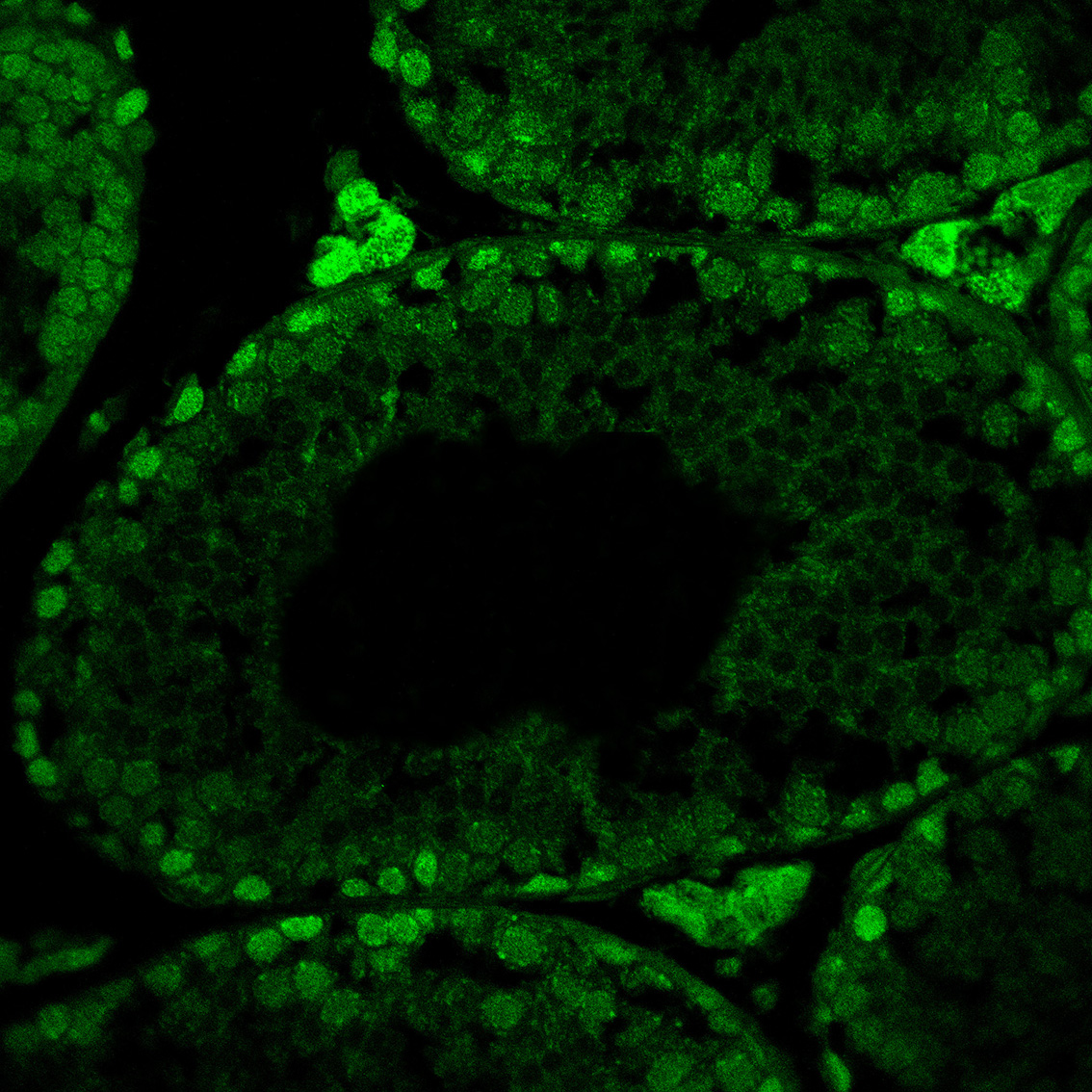

Supplement: Supplementary file 9 — Source data Fig. 2 [file 44318_2024_203_MOESM9_ESM.zip › Figure 2/Figure 2B/Ctrl-kdm2a.jpg]
