# Supplementary figures and images for: Histone demethylase KDM2A recruits HCFC1 and E2F1 to orchestrate male germ cell meiotic entry and progression (part 2 of 4)
Source: EMBO J. 2024 Aug 19;43(19):4197–227. doi: 10.1038/s44318-024-00203-4 (PMC11448500; doi:10.1038/s44318-024-00203-4)

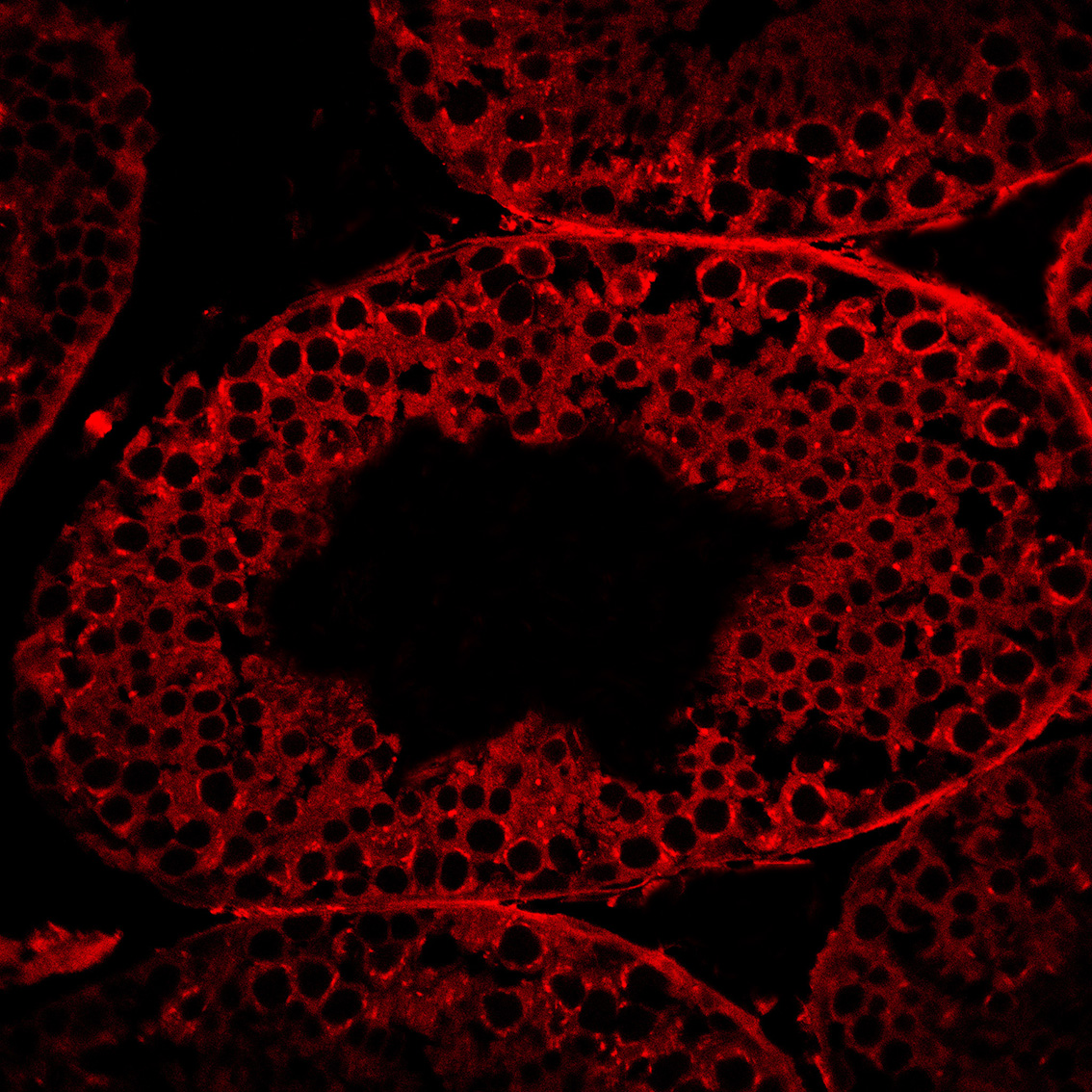

Supplement: Supplementary file 9 — Source data Fig. 2 [file 44318_2024_203_MOESM9_ESM.zip › Figure 2/Figure 2B/Ctrl-DDX4.jpg]

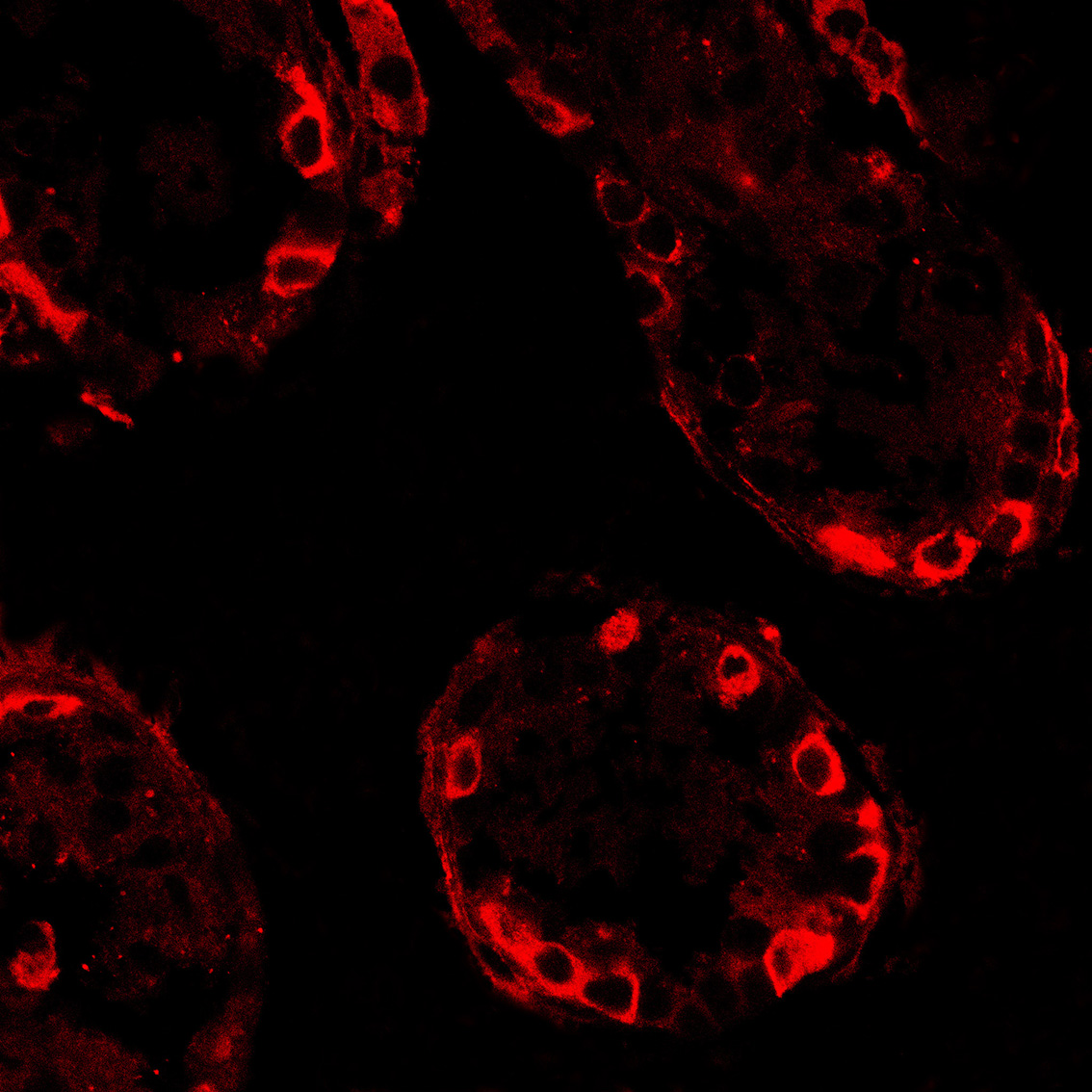

Supplement: Supplementary file 9 — Source data Fig. 2 [file 44318_2024_203_MOESM9_ESM.zip › Figure 2/Figure 2B/cKO-DDX4.jpg]

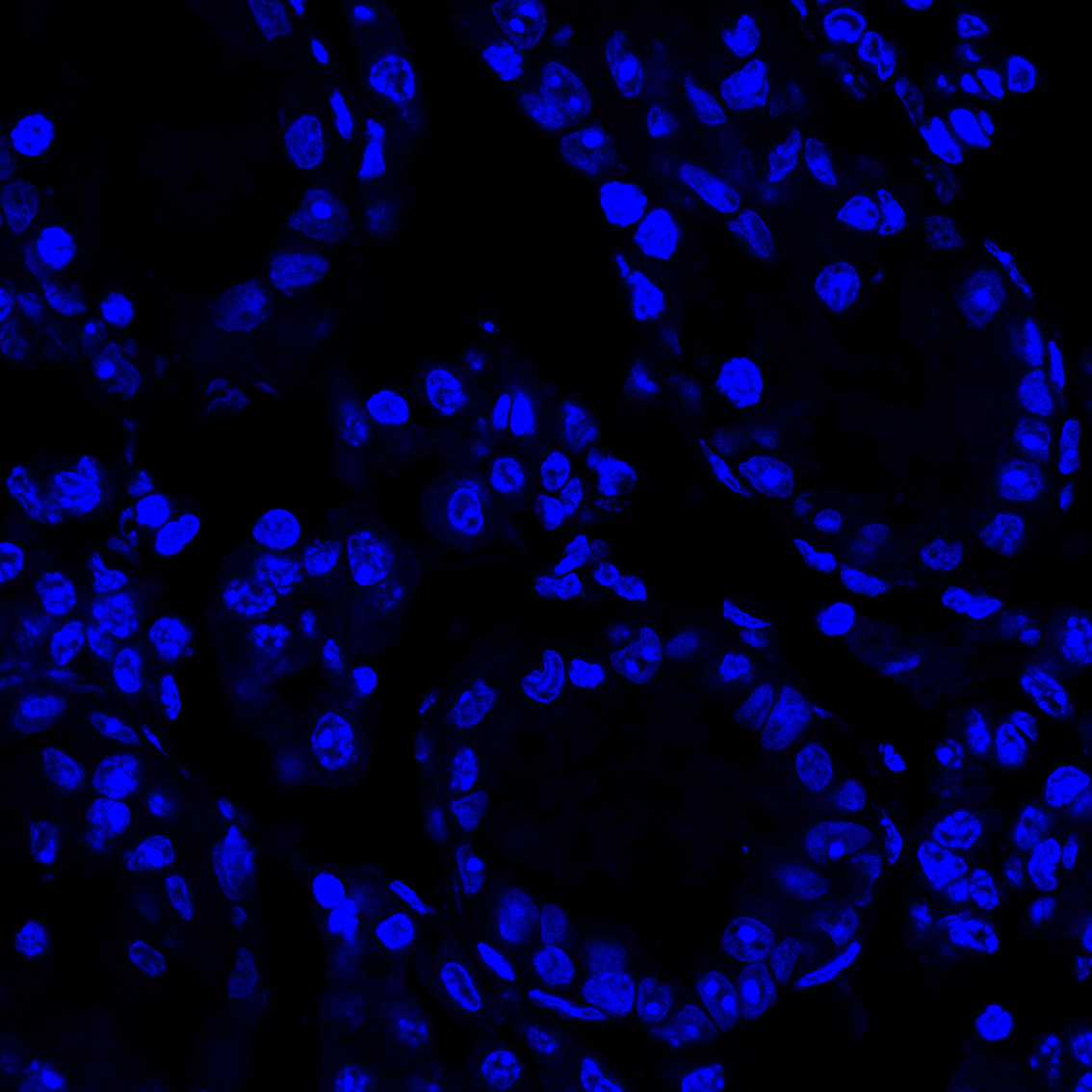

Supplement: Supplementary file 9 — Source data Fig. 2 [file 44318_2024_203_MOESM9_ESM.zip › Figure 2/Figure 2B/cKO-DAPI.jpg]

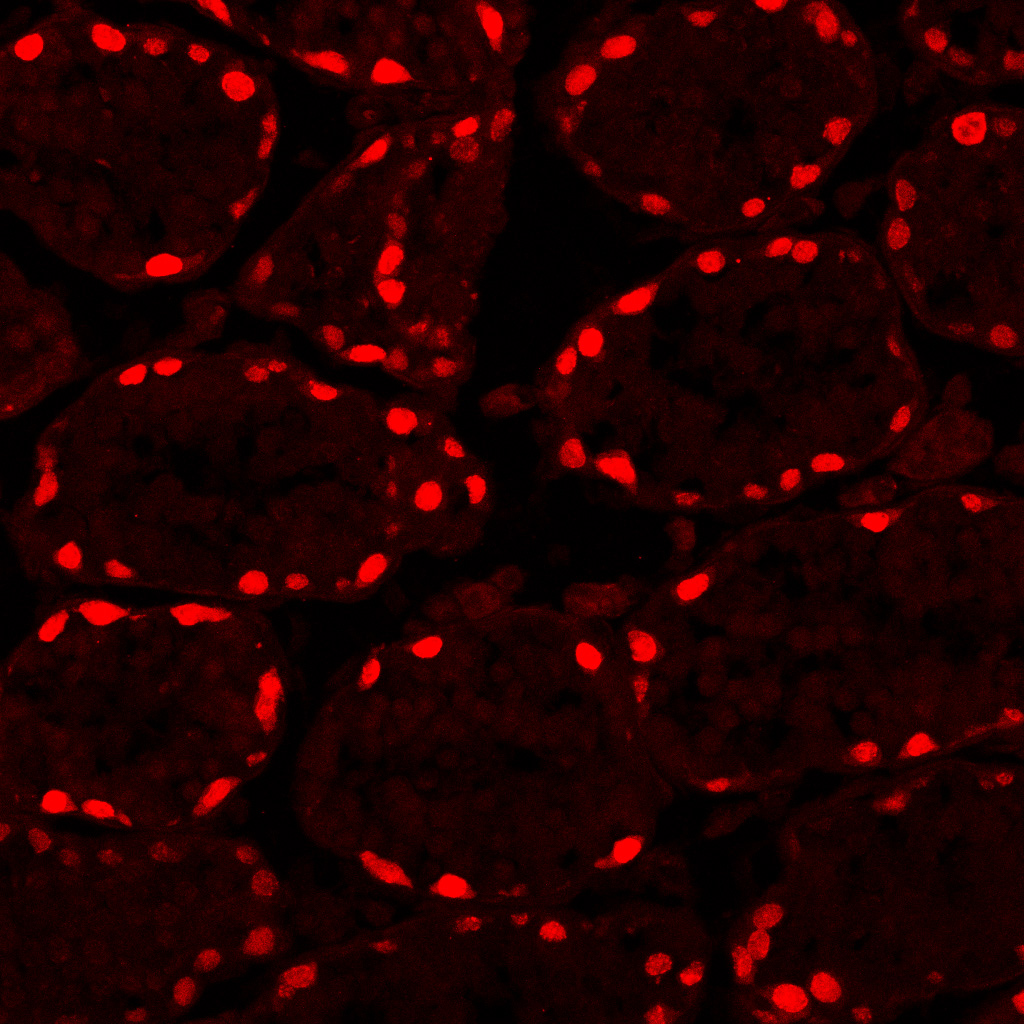

Supplement: Supplementary file 10 — Source data Fig. 3 [file 44318_2024_203_MOESM10_ESM.zip › Figure 3/Figure 3C/Ctrl-PLZF.jpg]

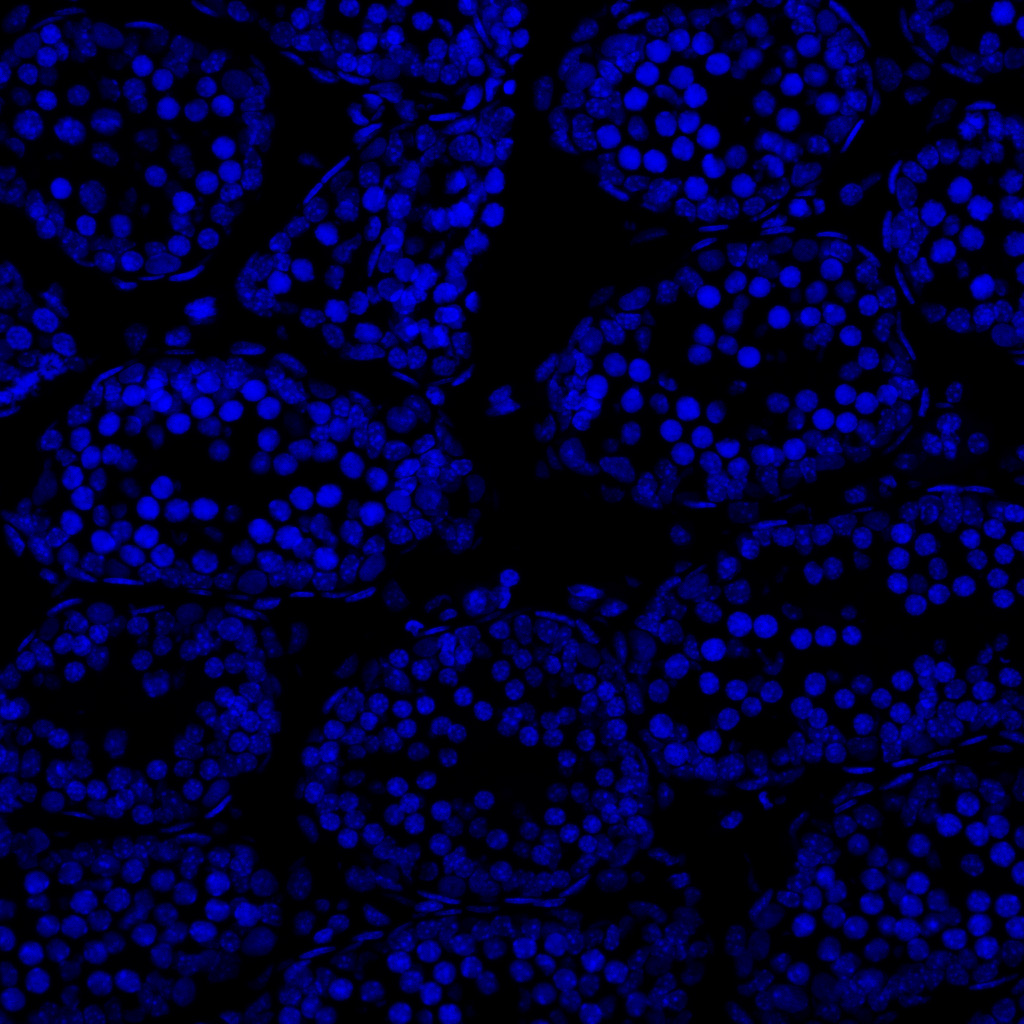

Supplement: Supplementary file 10 — Source data Fig. 3 [file 44318_2024_203_MOESM10_ESM.zip › Figure 3/Figure 3C/Ctrl-DAPI.jpg]

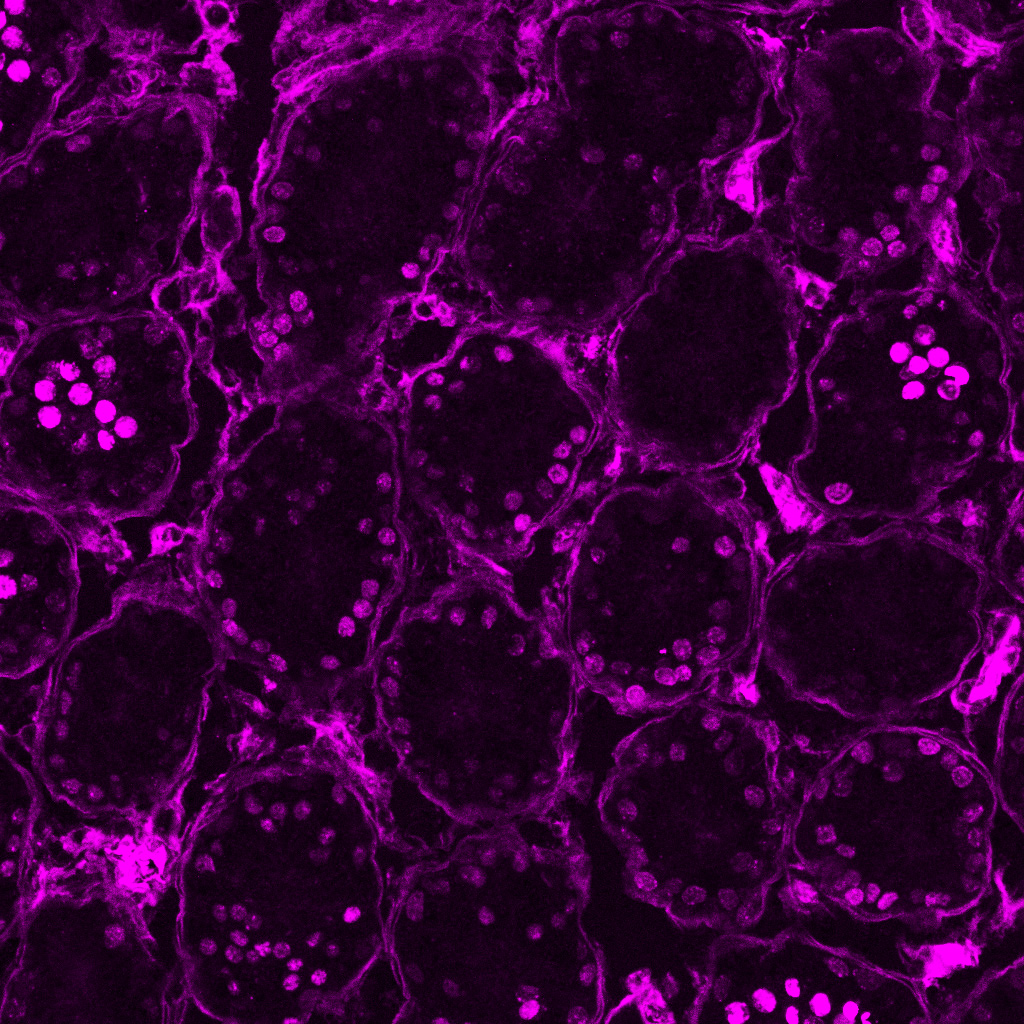

Supplement: Supplementary file 10 — Source data Fig. 3 [file 44318_2024_203_MOESM10_ESM.zip › Figure 3/Figure 3C/cKO-╬│H2AX.jpg]

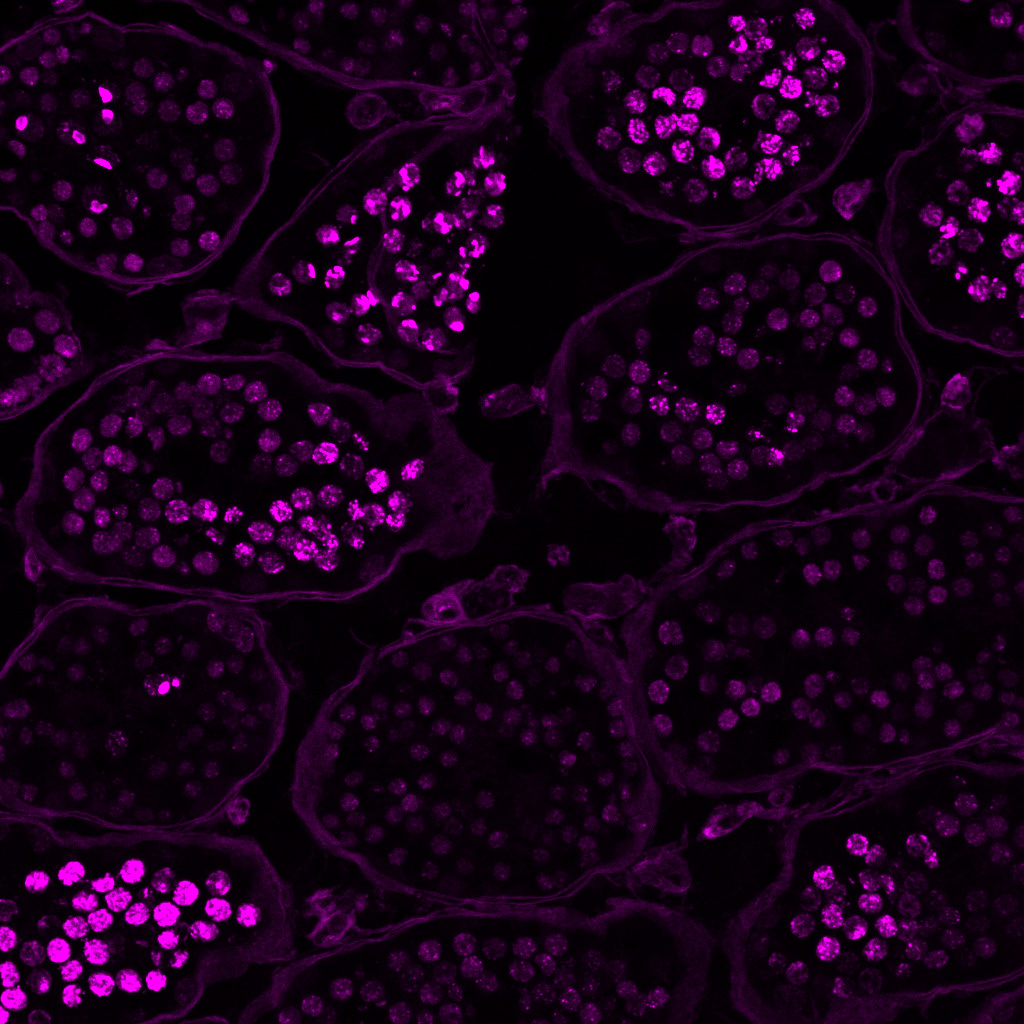

Supplement: Supplementary file 10 — Source data Fig. 3 [file 44318_2024_203_MOESM10_ESM.zip › Figure 3/Figure 3C/Ctrl-╬│H2AX.jpg]

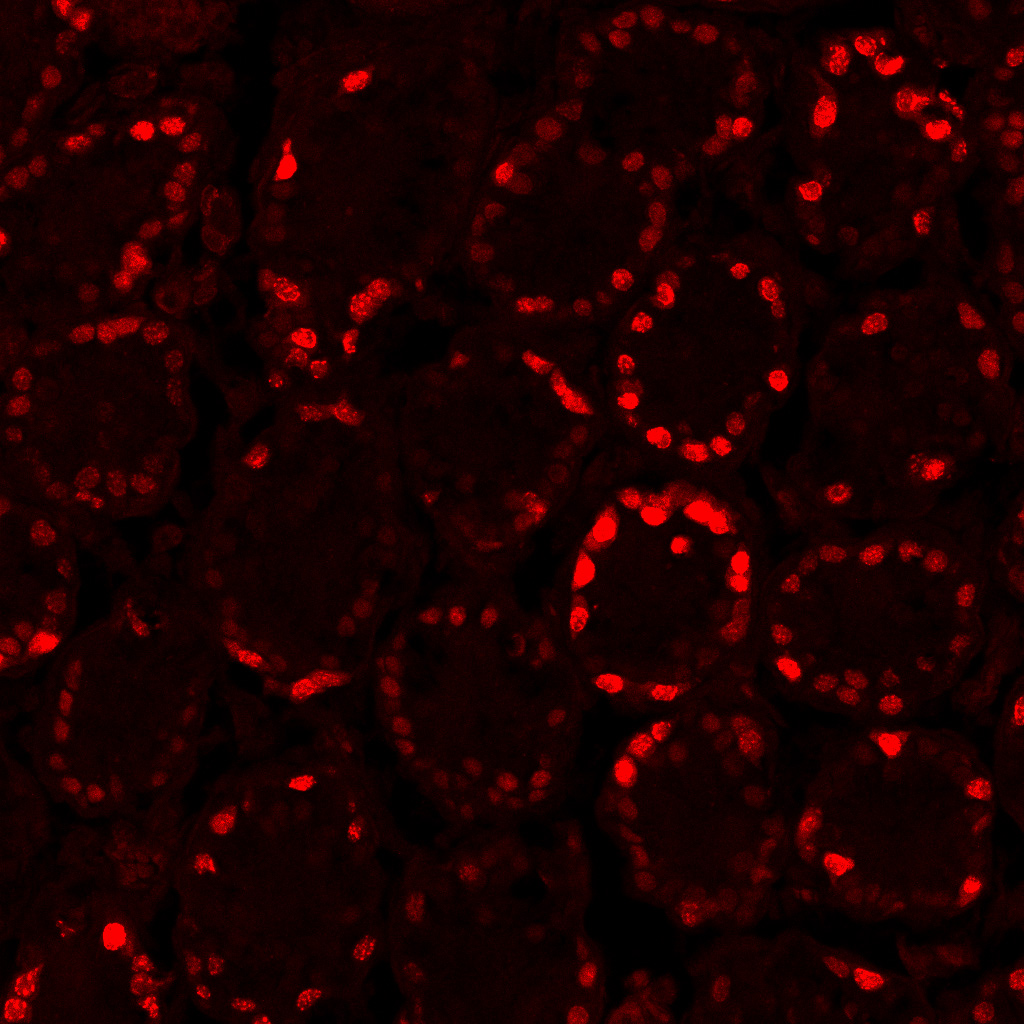

Supplement: Supplementary file 10 — Source data Fig. 3 [file 44318_2024_203_MOESM10_ESM.zip › Figure 3/Figure 3C/cKO-PLZF.jpg]

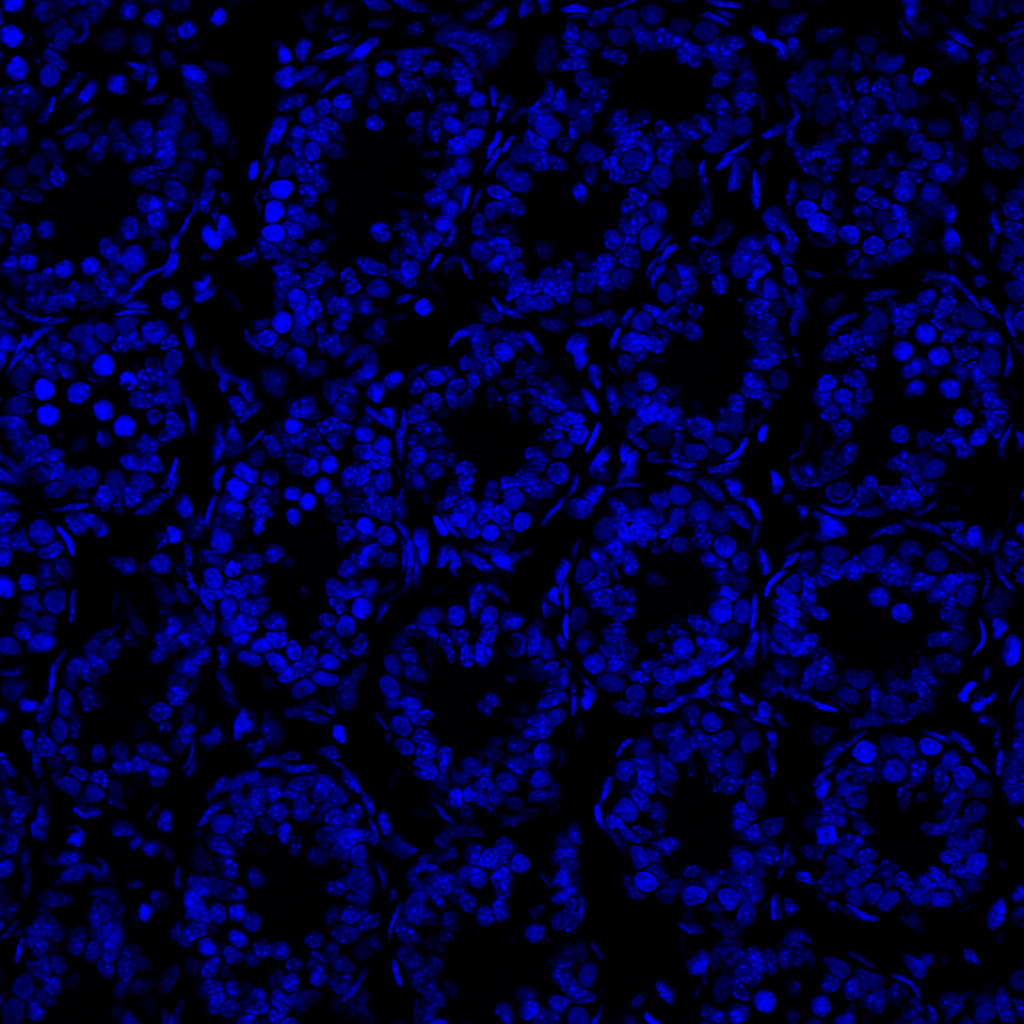

Supplement: Supplementary file 10 — Source data Fig. 3 [file 44318_2024_203_MOESM10_ESM.zip › Figure 3/Figure 3C/cKO-DAPI.jpg]

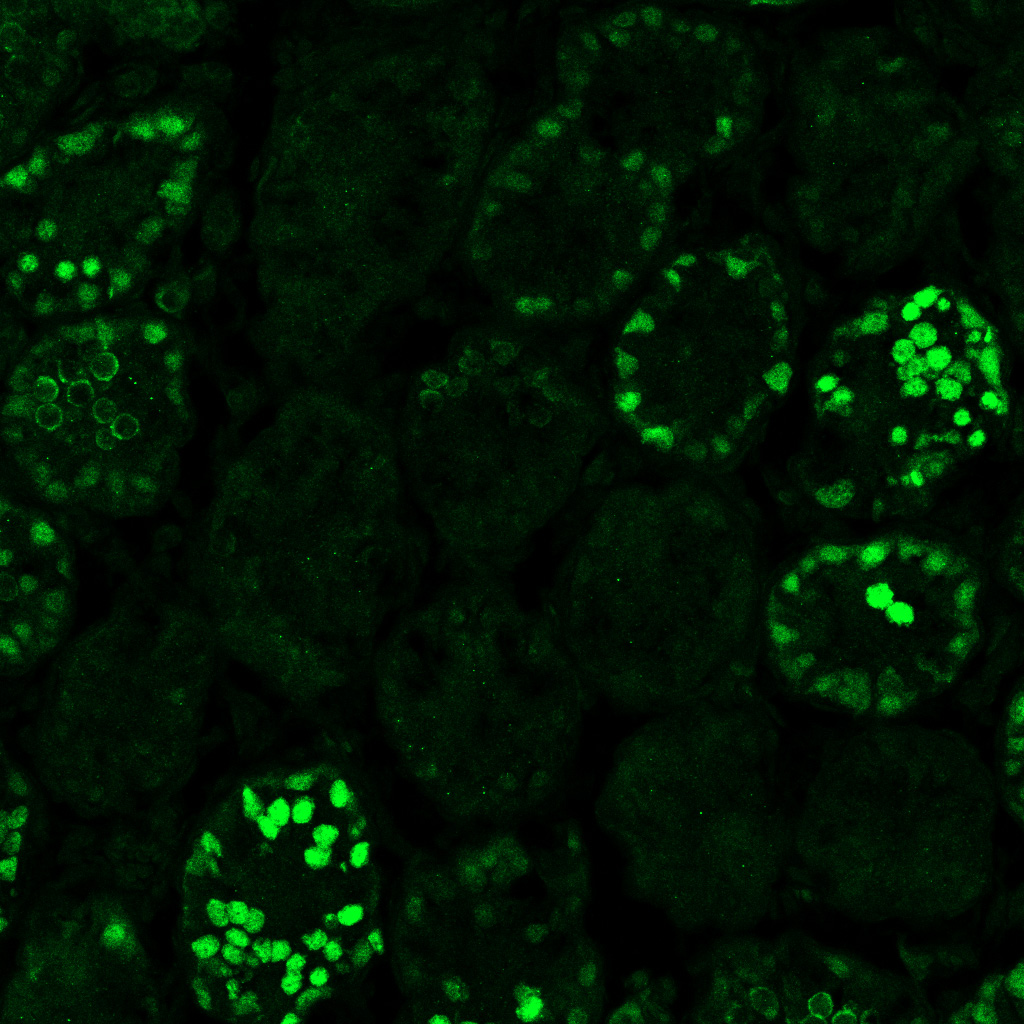

Supplement: Supplementary file 10 — Source data Fig. 3 [file 44318_2024_203_MOESM10_ESM.zip › Figure 3/Figure 3C/cKO-STRA8.jpg]

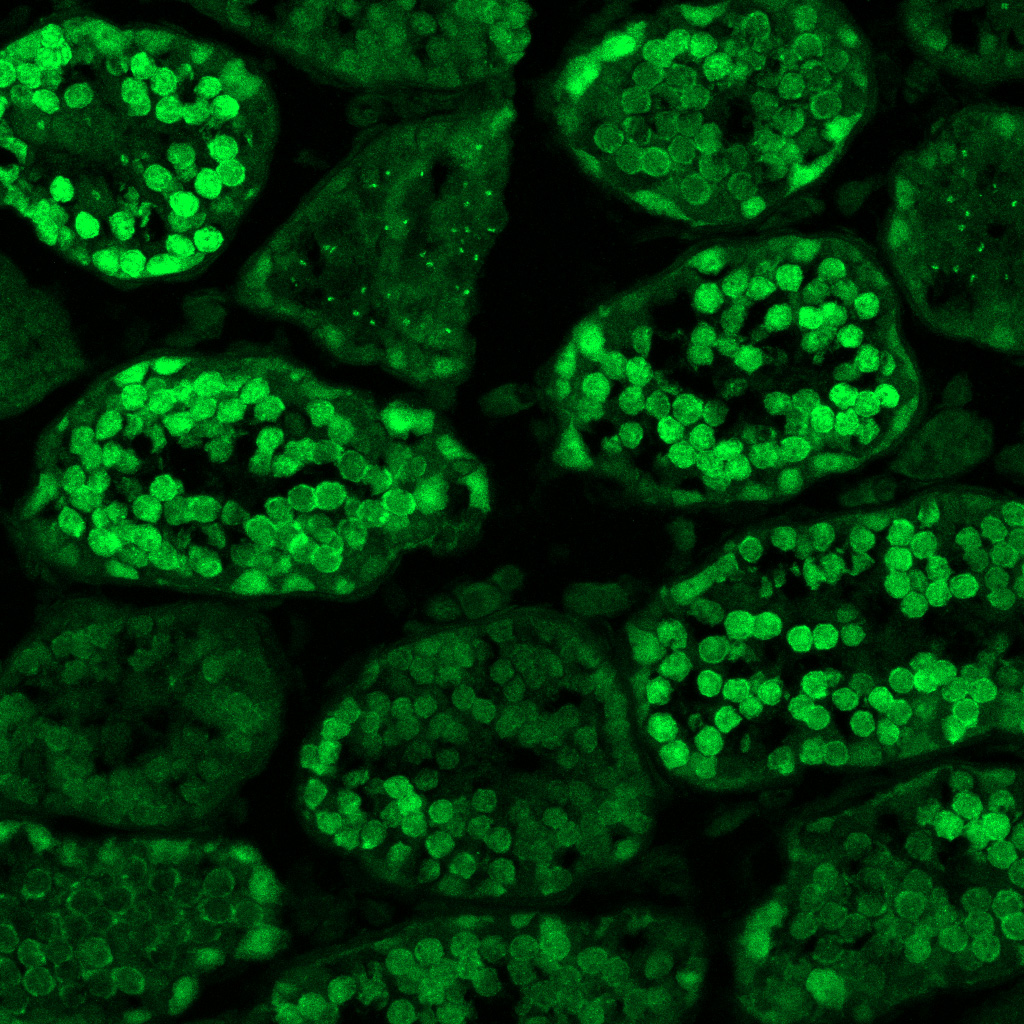

Supplement: Supplementary file 10 — Source data Fig. 3 [file 44318_2024_203_MOESM10_ESM.zip › Figure 3/Figure 3C/Ctrl-STRA8.jpg]

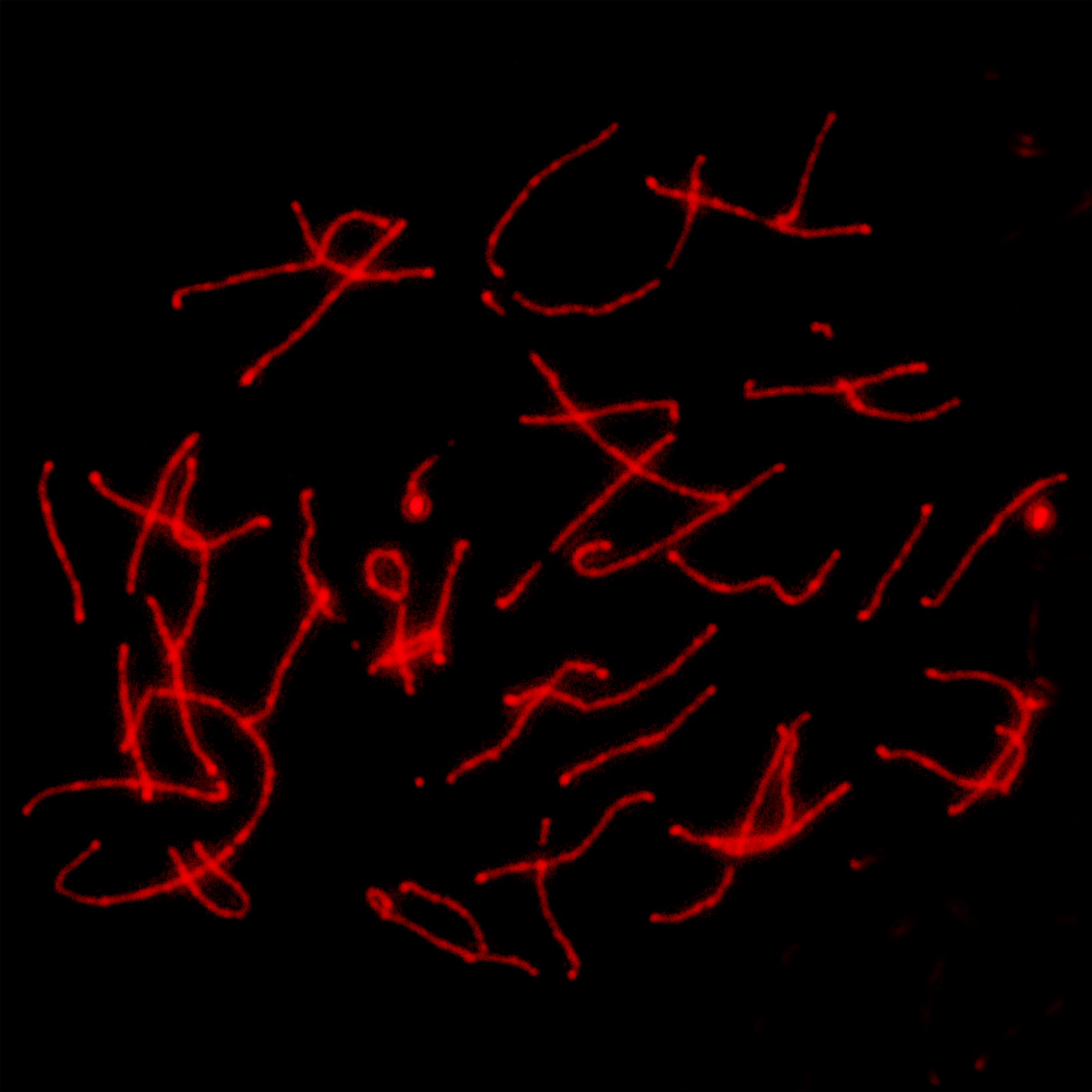

Supplement: Supplementary file 10 — Source data Fig. 3 [file 44318_2024_203_MOESM10_ESM.zip › Figure 3/Figure 3H/cKO-Zyg-ii SYCP3.jpg]

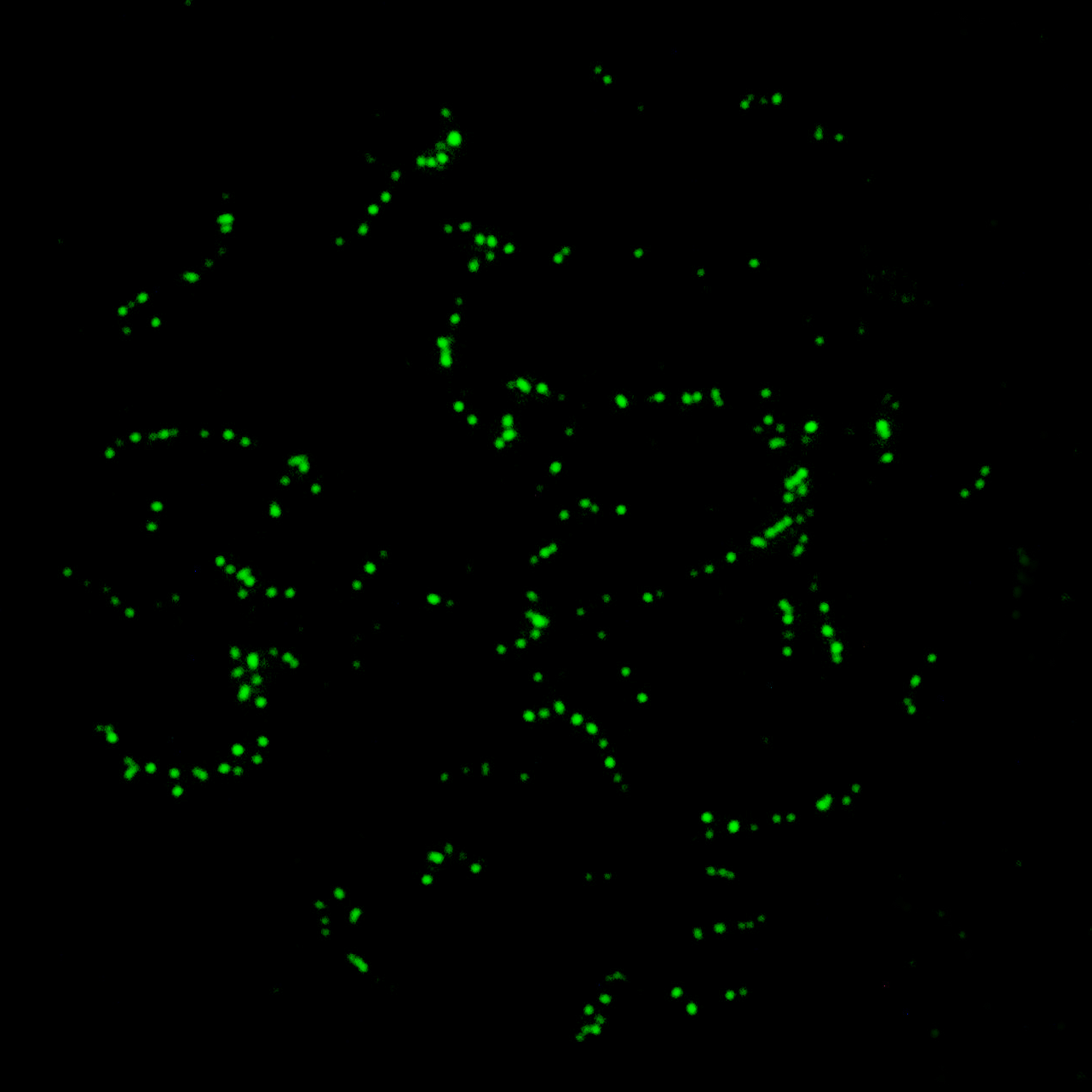

Supplement: Supplementary file 10 — Source data Fig. 3 [file 44318_2024_203_MOESM10_ESM.zip › Figure 3/Figure 3H/Ctrl-Zyg-RAD51.jpg]

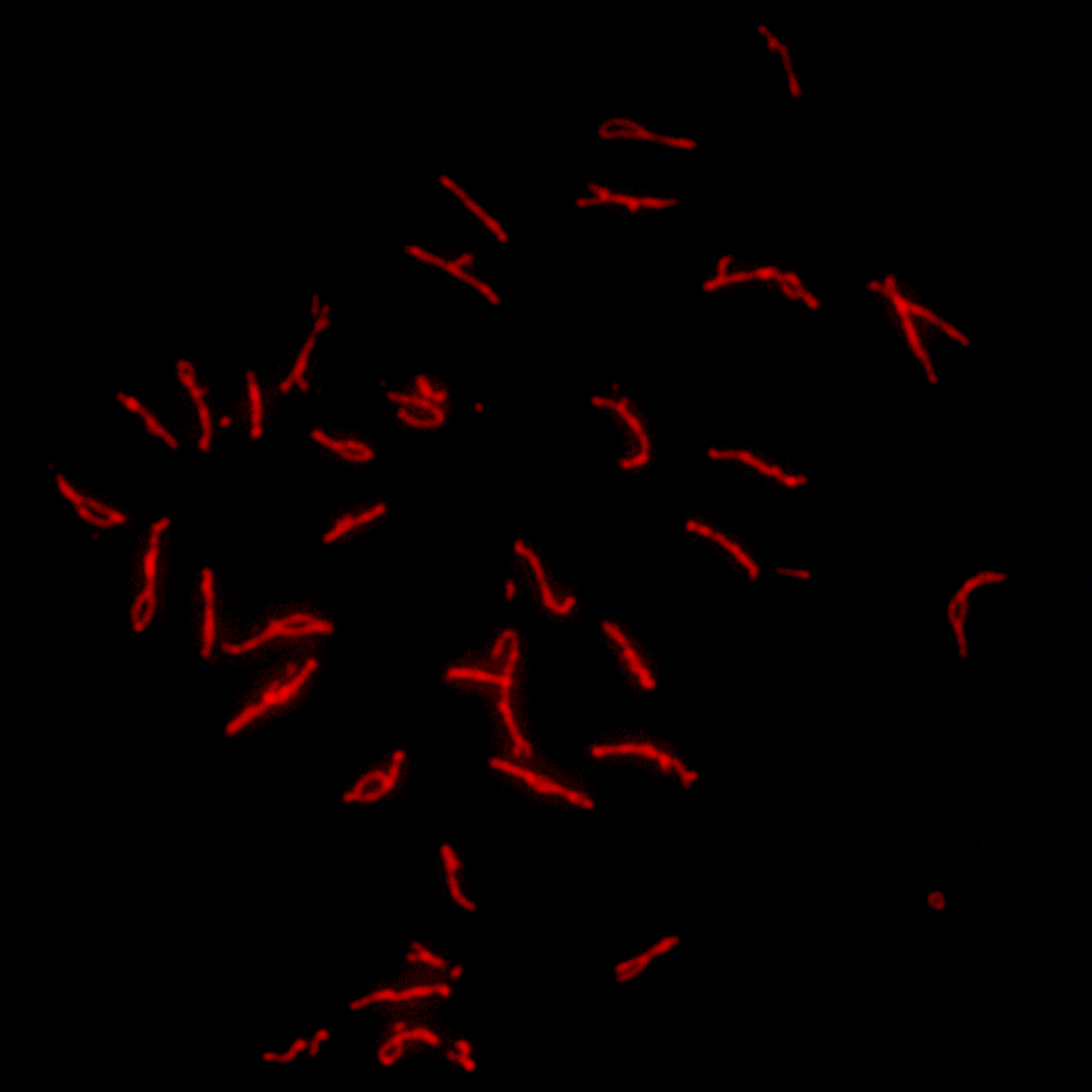

Supplement: Supplementary file 10 — Source data Fig. 3 [file 44318_2024_203_MOESM10_ESM.zip › Figure 3/Figure 3H/cKO-Zyg-i SYCP3.jpg]

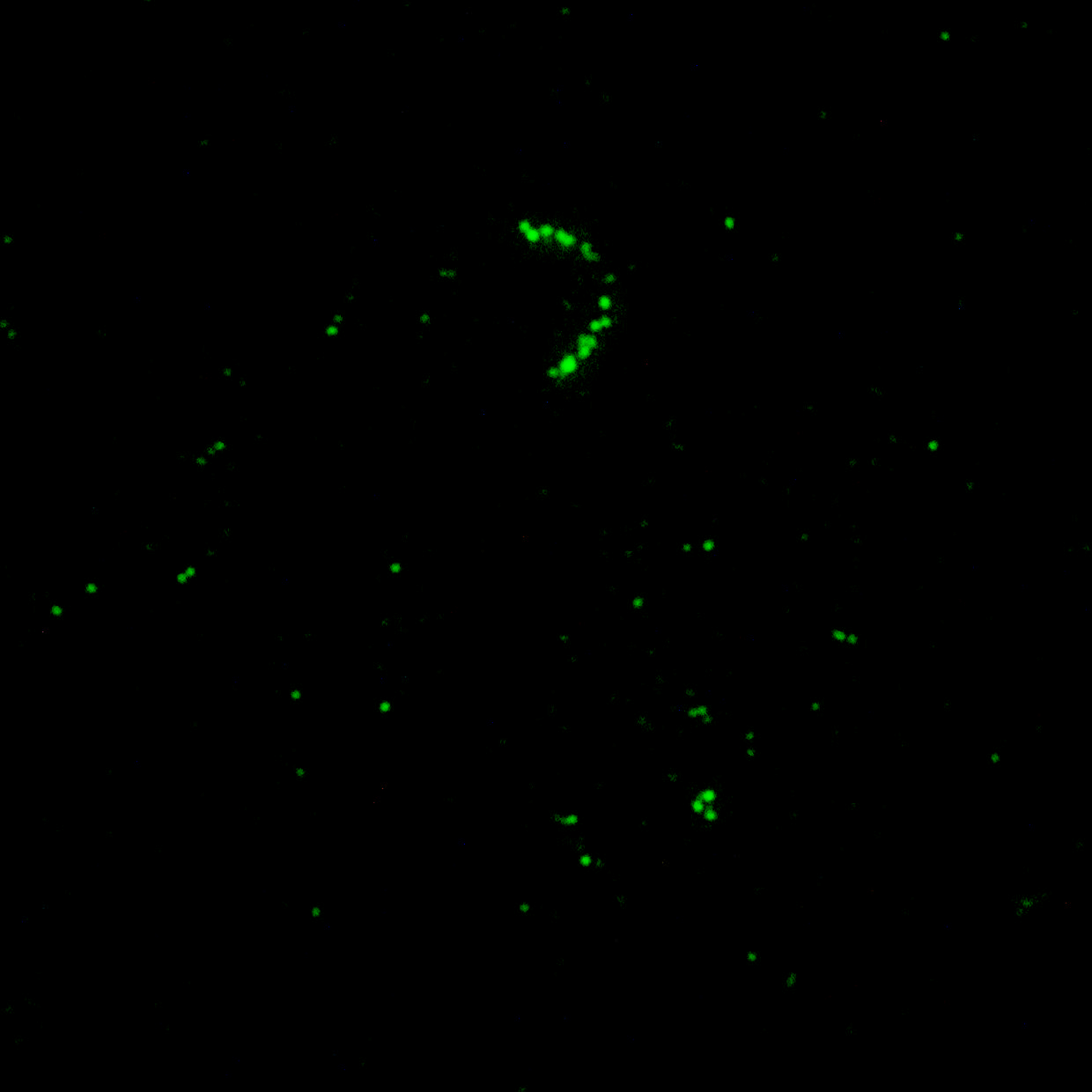

Supplement: Supplementary file 10 — Source data Fig. 3 [file 44318_2024_203_MOESM10_ESM.zip › Figure 3/Figure 3H/Ctrl-Pac-RAD51.jpg]

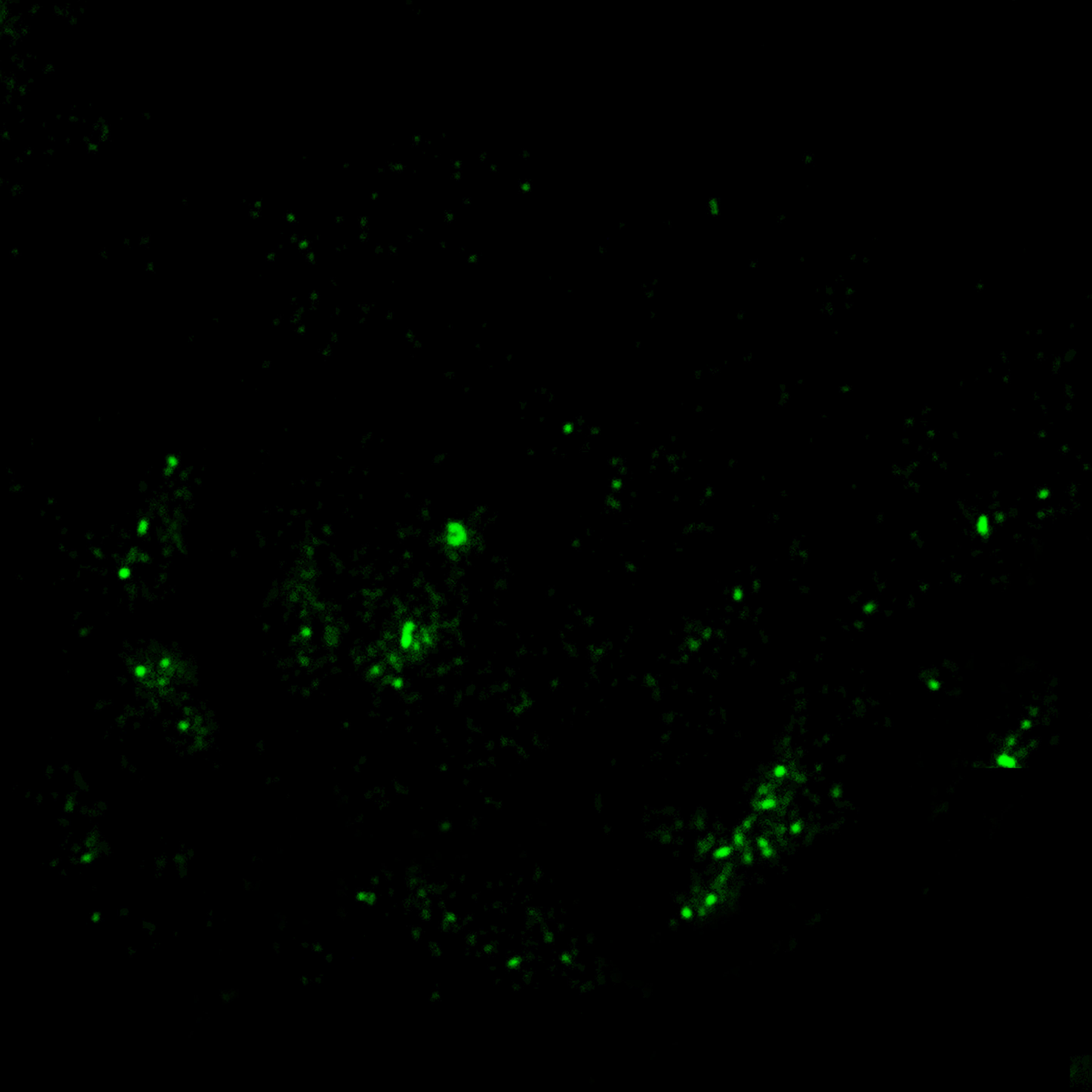

Supplement: Supplementary file 10 — Source data Fig. 3 [file 44318_2024_203_MOESM10_ESM.zip › Figure 3/Figure 3H/cKO-Zyg-ii RAD51.jpg]

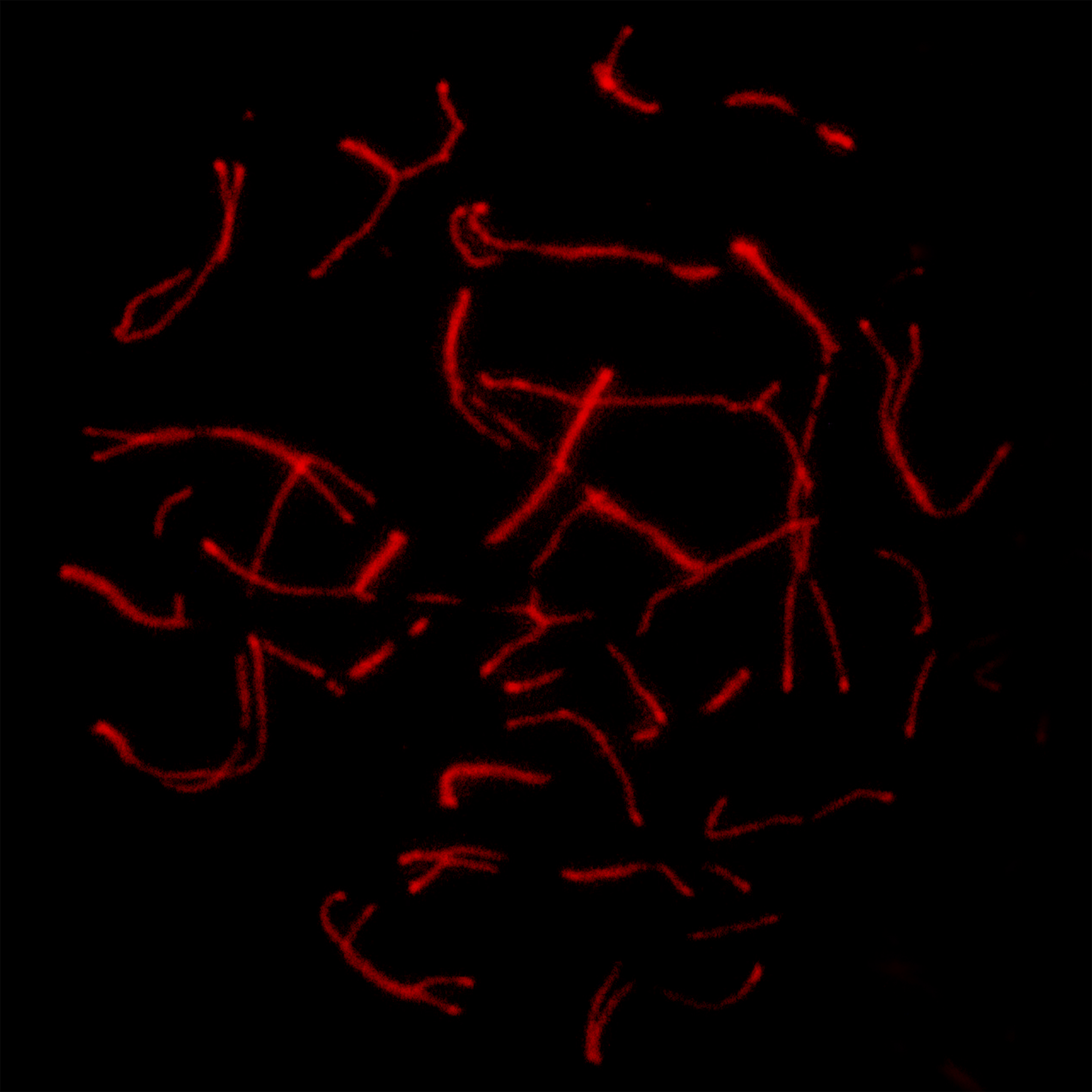

Supplement: Supplementary file 10 — Source data Fig. 3 [file 44318_2024_203_MOESM10_ESM.zip › Figure 3/Figure 3H/Ctrl-Zyg-SYCP3.jpg]

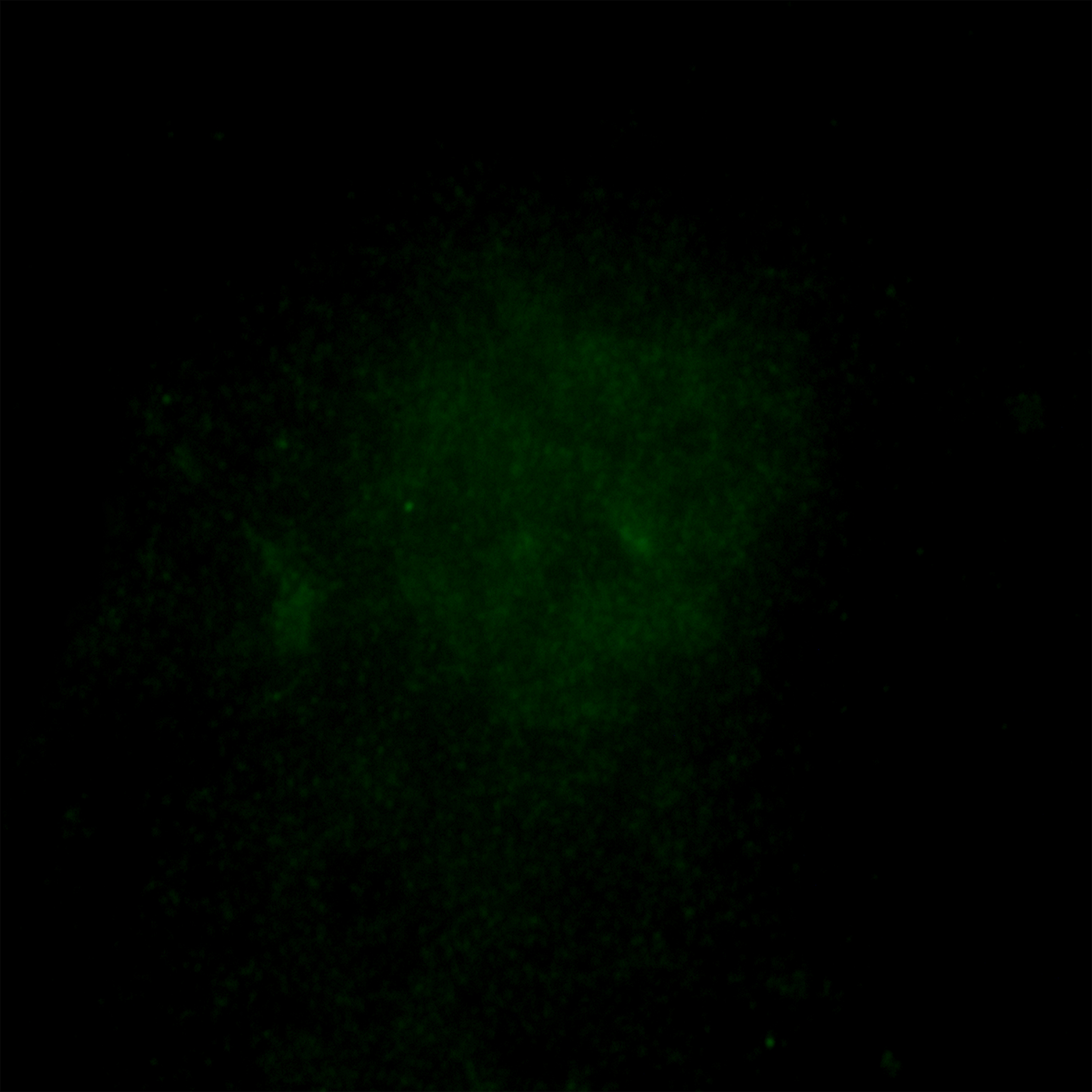

Supplement: Supplementary file 10 — Source data Fig. 3 [file 44318_2024_203_MOESM10_ESM.zip › Figure 3/Figure 3H/cKO-Zyg-i RAD51.jpg]

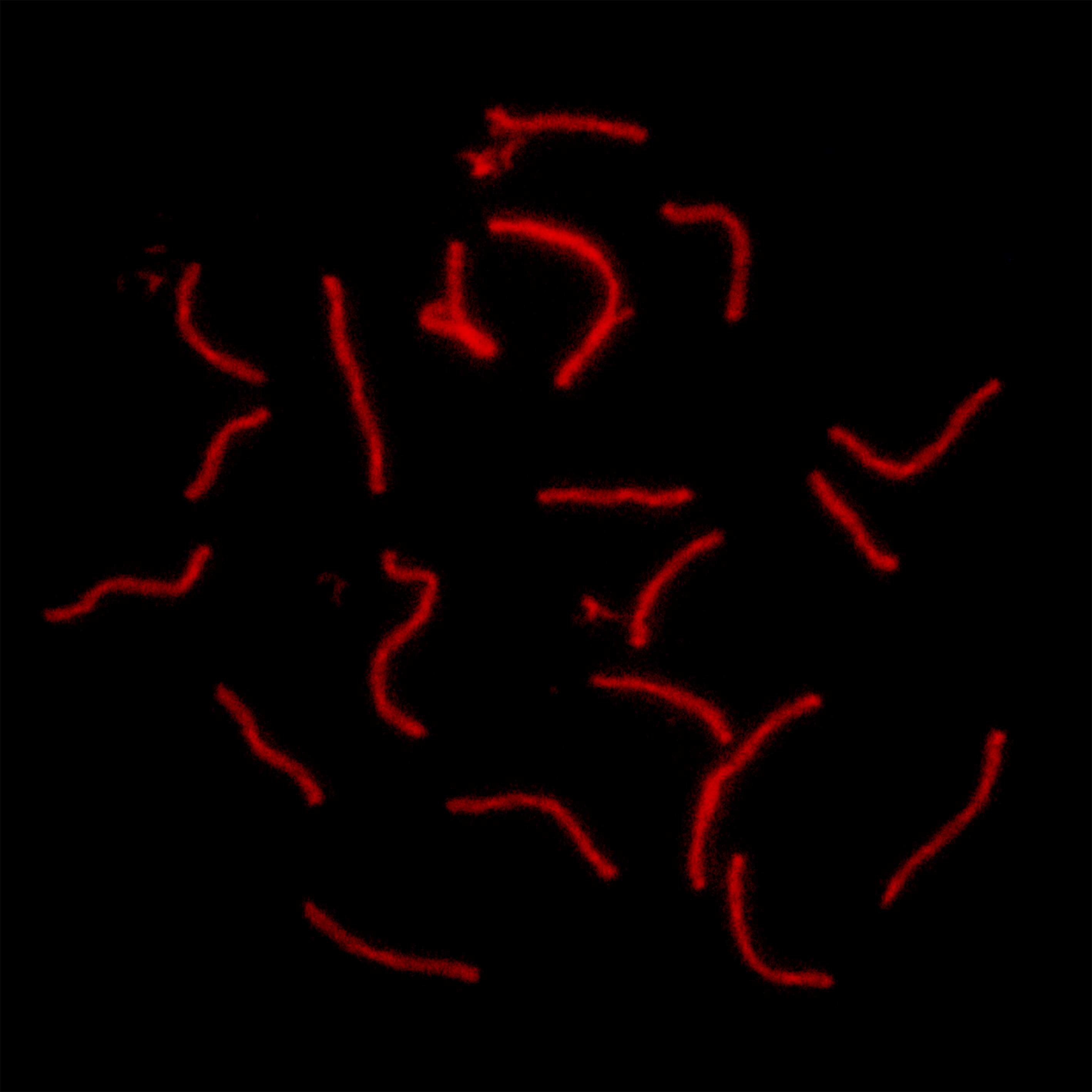

Supplement: Supplementary file 10 — Source data Fig. 3 [file 44318_2024_203_MOESM10_ESM.zip › Figure 3/Figure 3H/Ctrl-Pac-SYCP3.jpg]

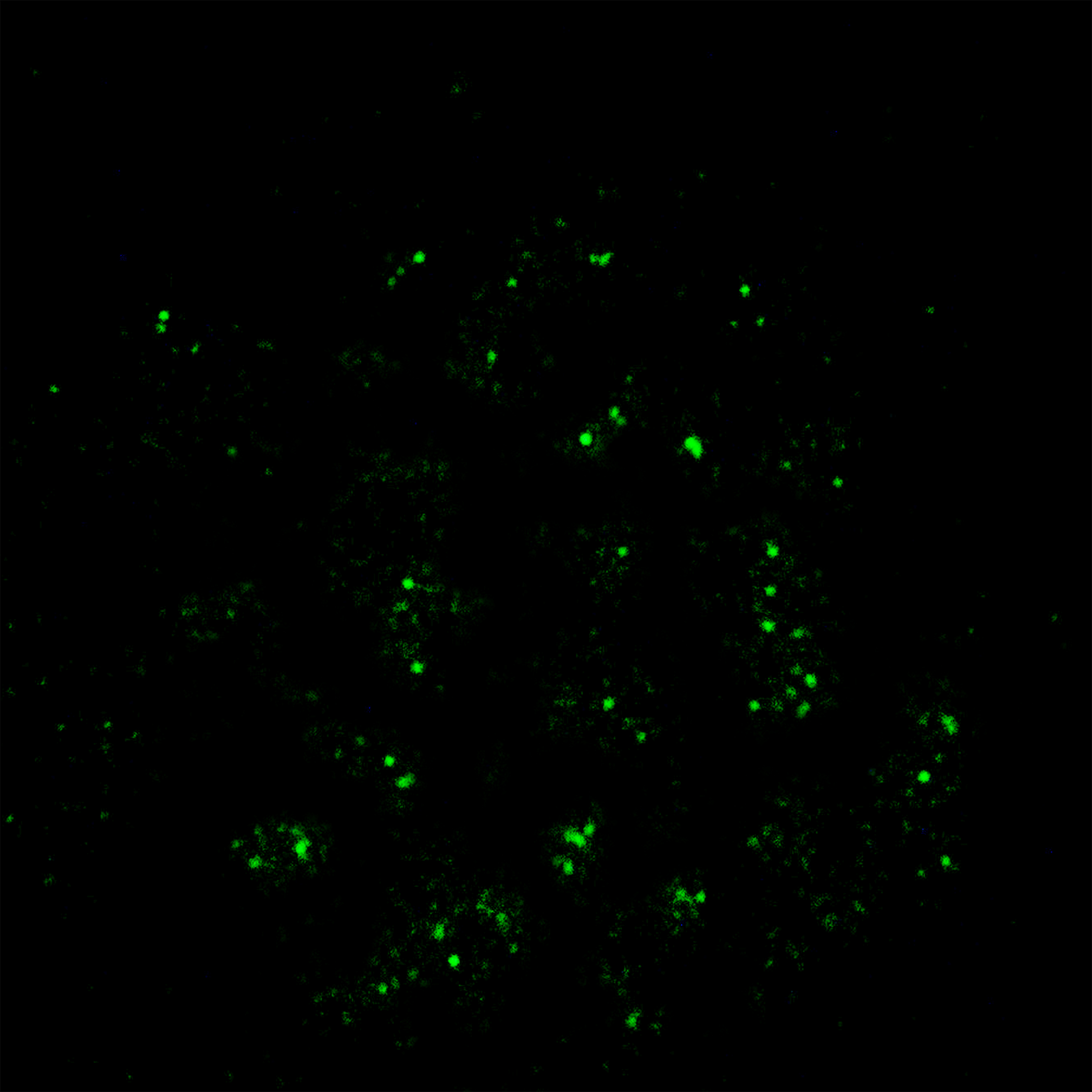

Supplement: Supplementary file 10 — Source data Fig. 3 [file 44318_2024_203_MOESM10_ESM.zip › Figure 3/Figure 3H/cKO-Lep-RAD51.jpg]

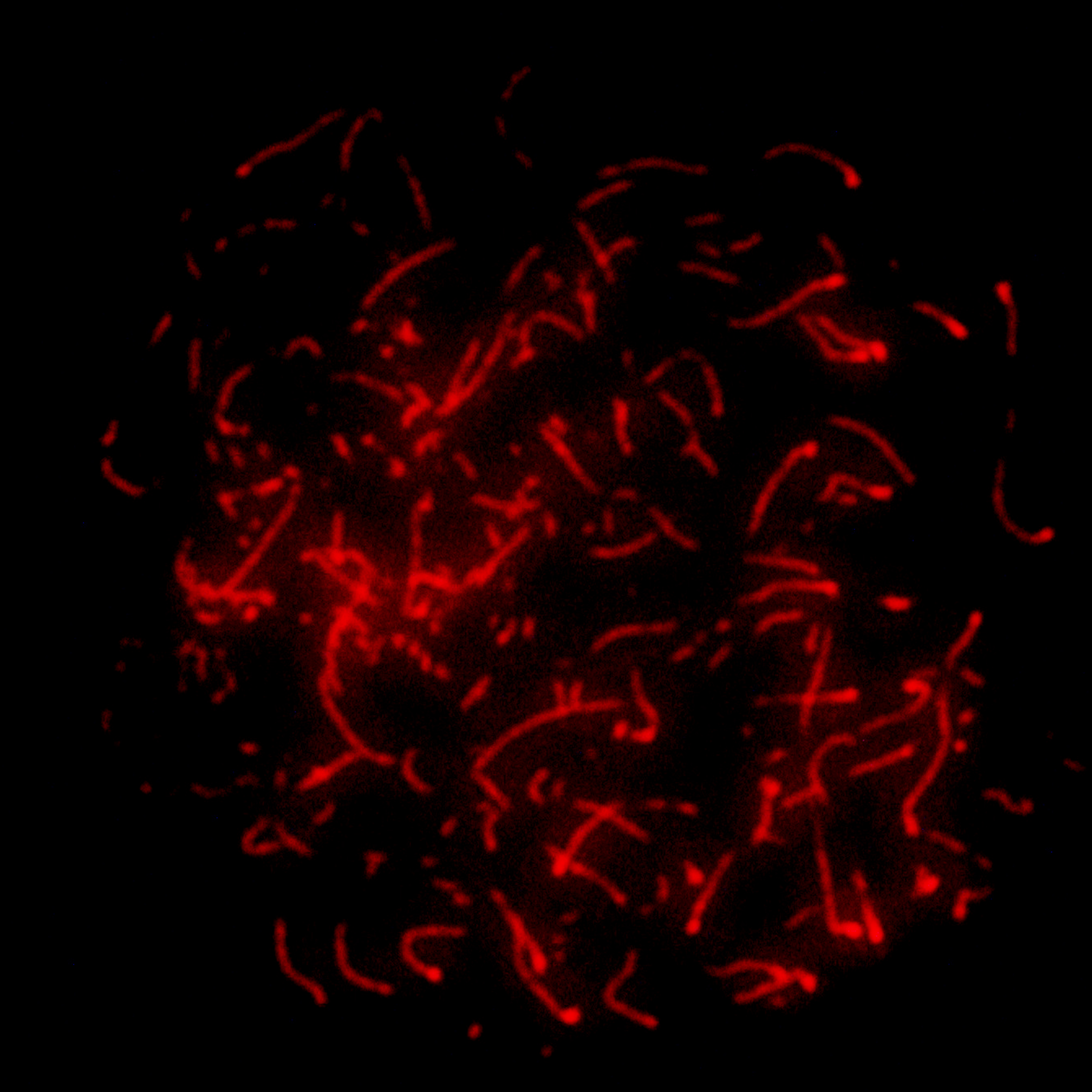

Supplement: Supplementary file 10 — Source data Fig. 3 [file 44318_2024_203_MOESM10_ESM.zip › Figure 3/Figure 3H/cKO-Lep-SYCP3.jpg]

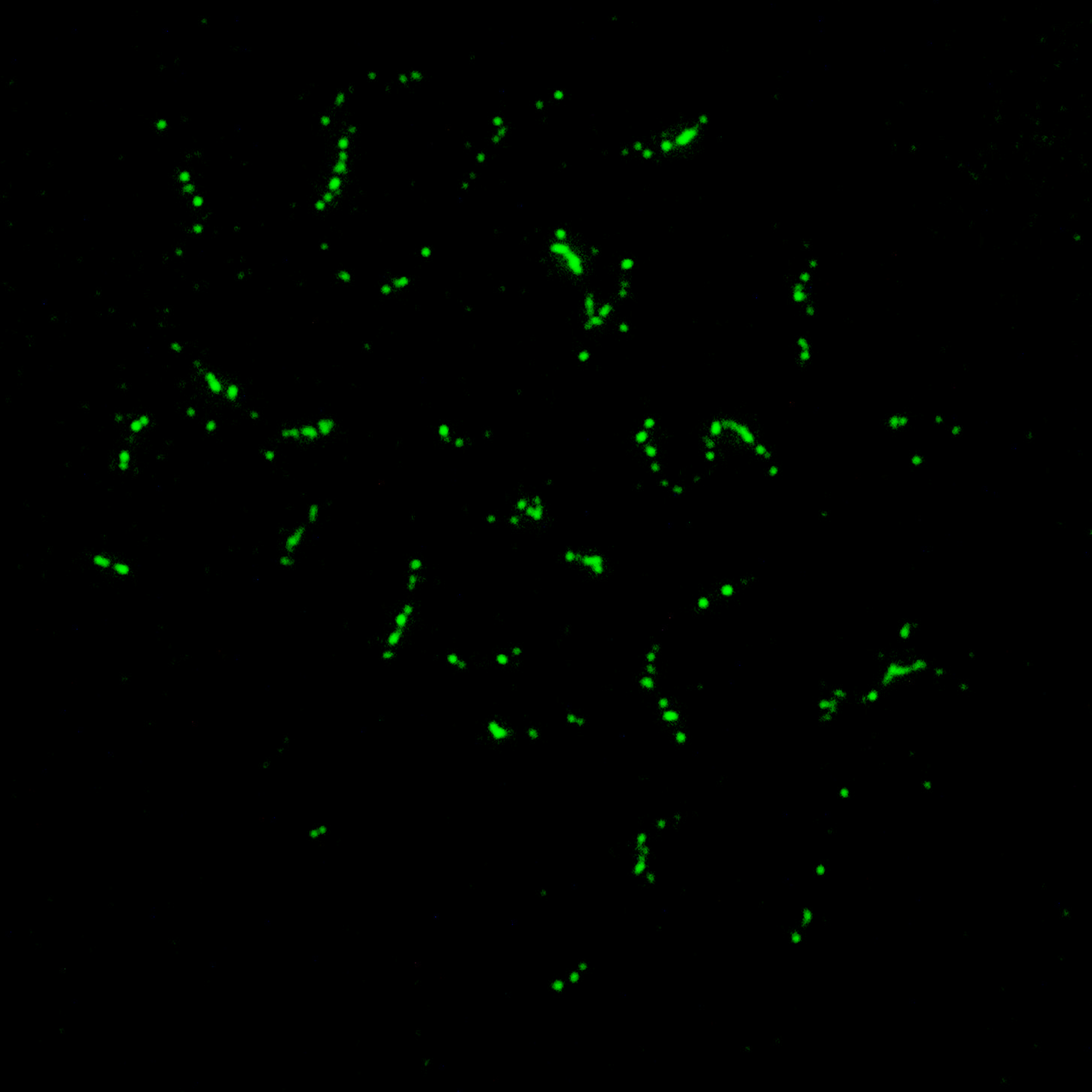

Supplement: Supplementary file 10 — Source data Fig. 3 [file 44318_2024_203_MOESM10_ESM.zip › Figure 3/Figure 3H/Ctrl-Lep-RAD51.jpg]

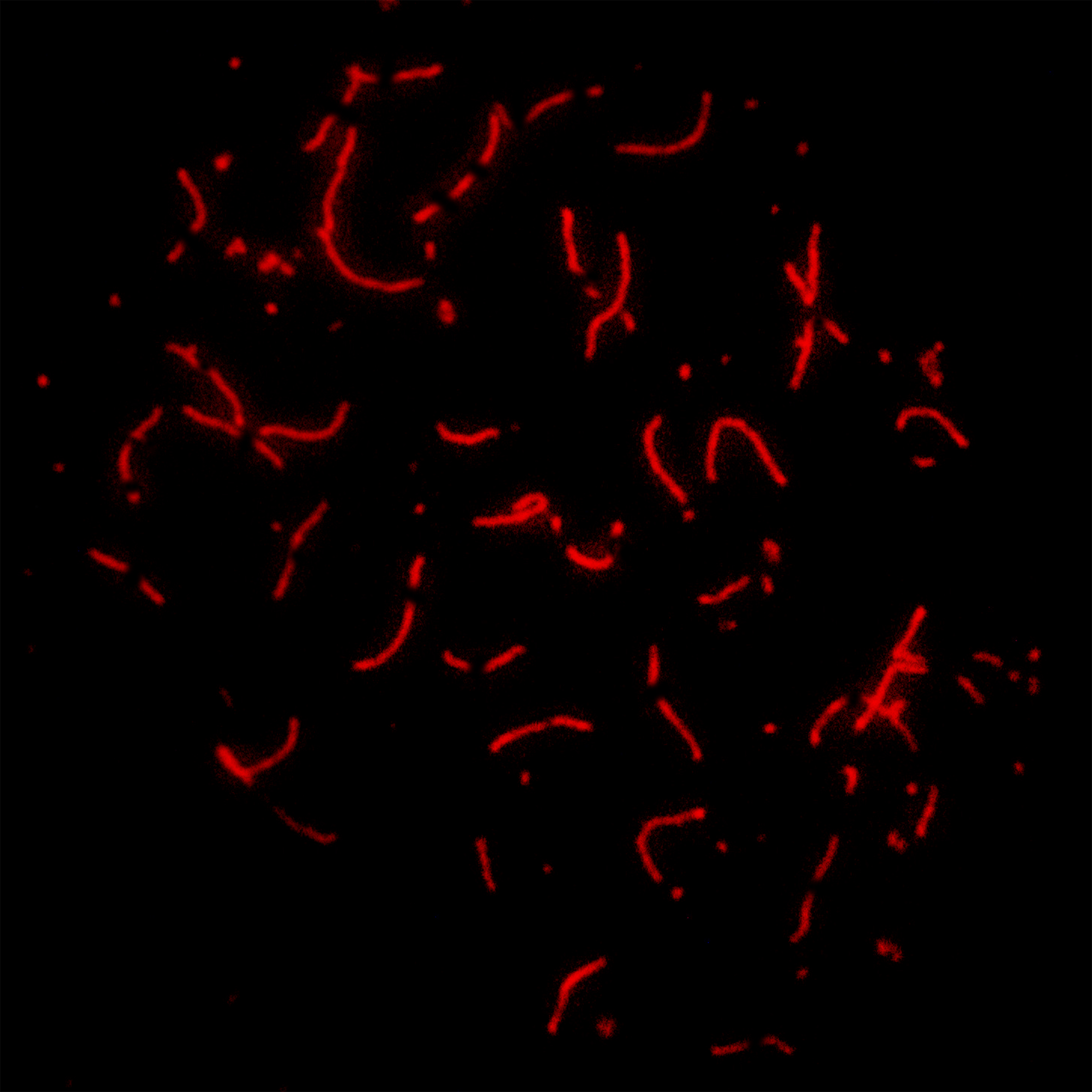

Supplement: Supplementary file 10 — Source data Fig. 3 [file 44318_2024_203_MOESM10_ESM.zip › Figure 3/Figure 3H/Ctrl-Lep-SYCP3.jpg]

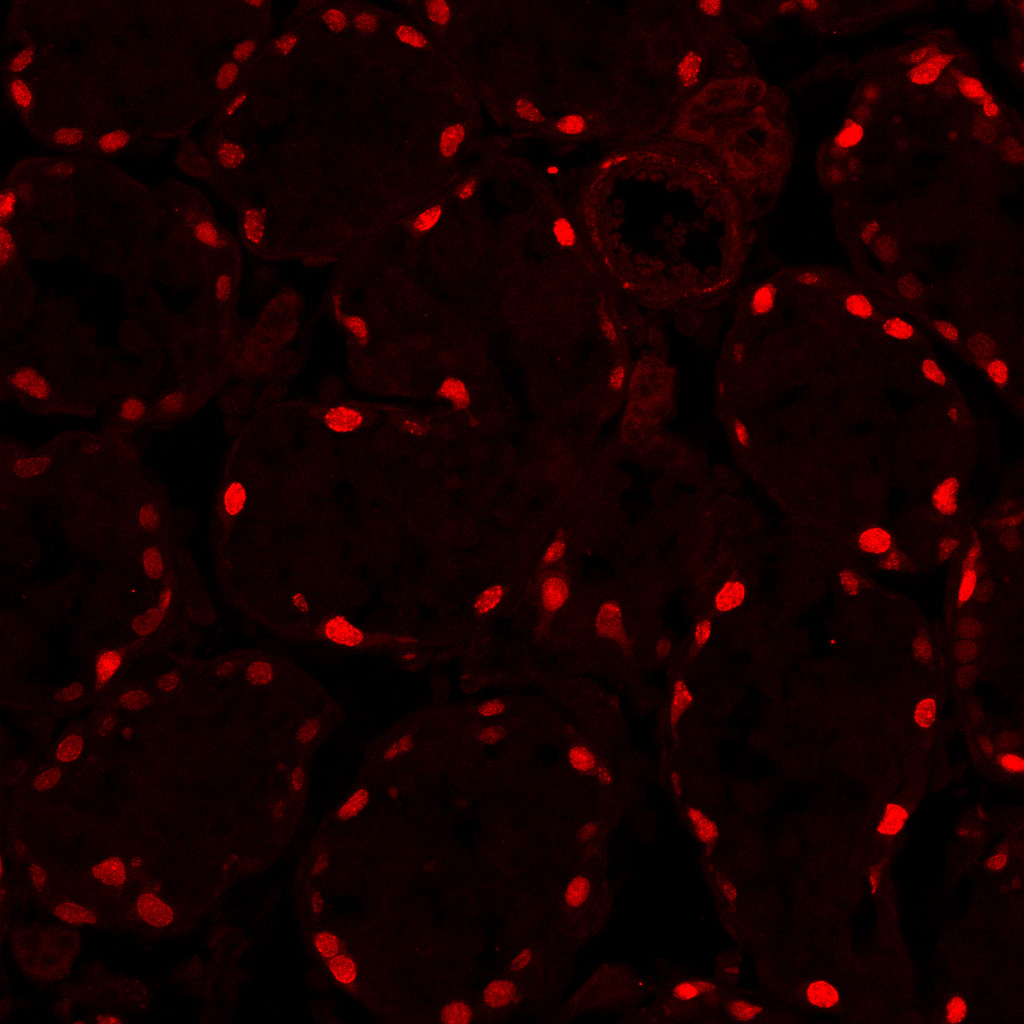

Supplement: Supplementary file 10 — Source data Fig. 3 [file 44318_2024_203_MOESM10_ESM.zip › Figure 3/Figure 3A/Ctrl-PLZF.jpg]

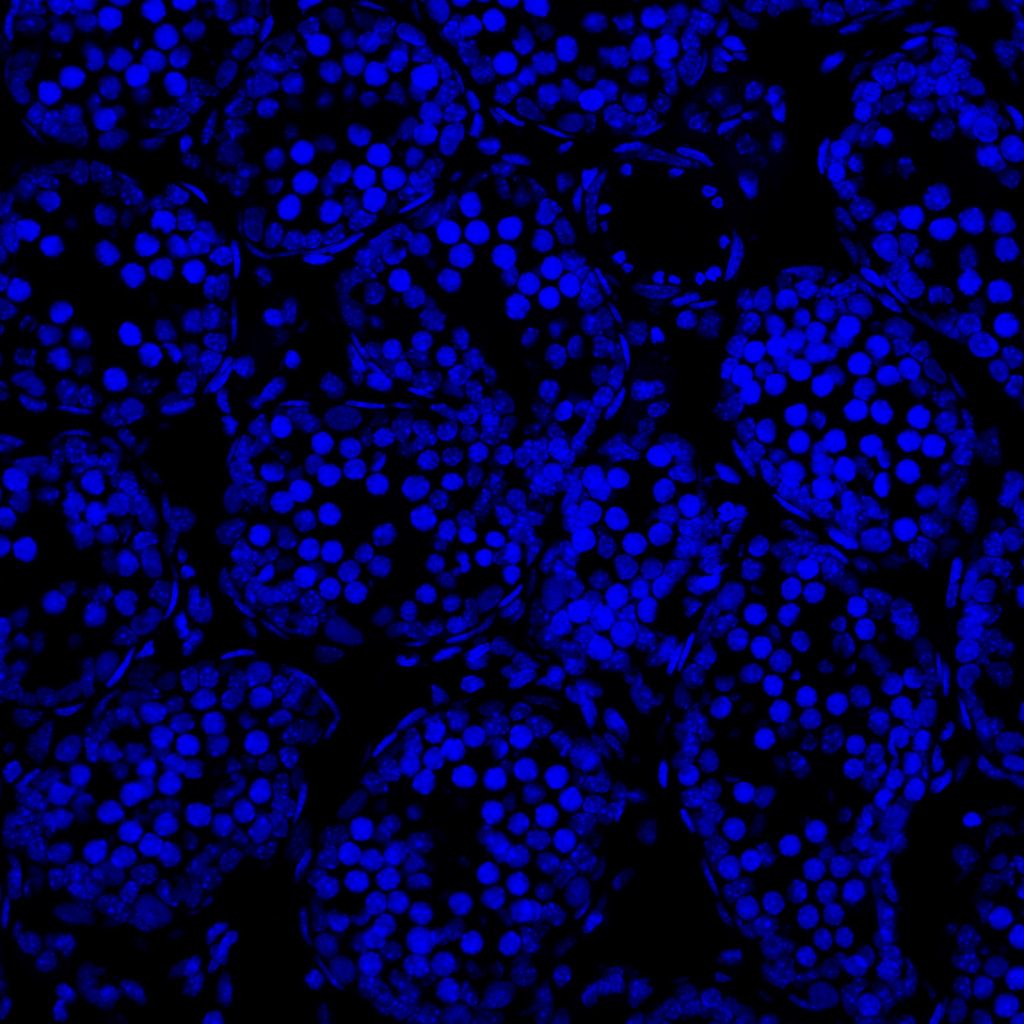

Supplement: Supplementary file 10 — Source data Fig. 3 [file 44318_2024_203_MOESM10_ESM.zip › Figure 3/Figure 3A/Ctrl-DAPI.jpg]

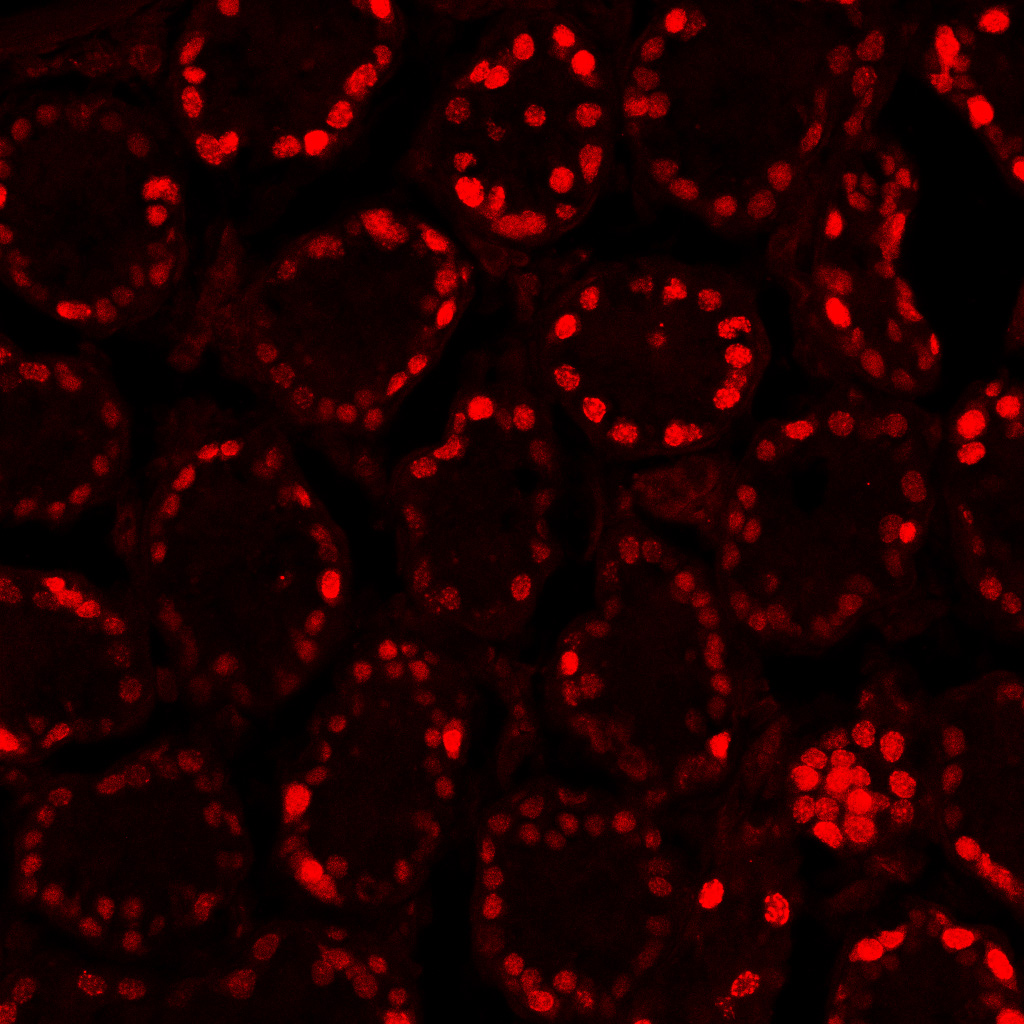

Supplement: Supplementary file 10 — Source data Fig. 3 [file 44318_2024_203_MOESM10_ESM.zip › Figure 3/Figure 3A/cKO-PLZF.jpg]

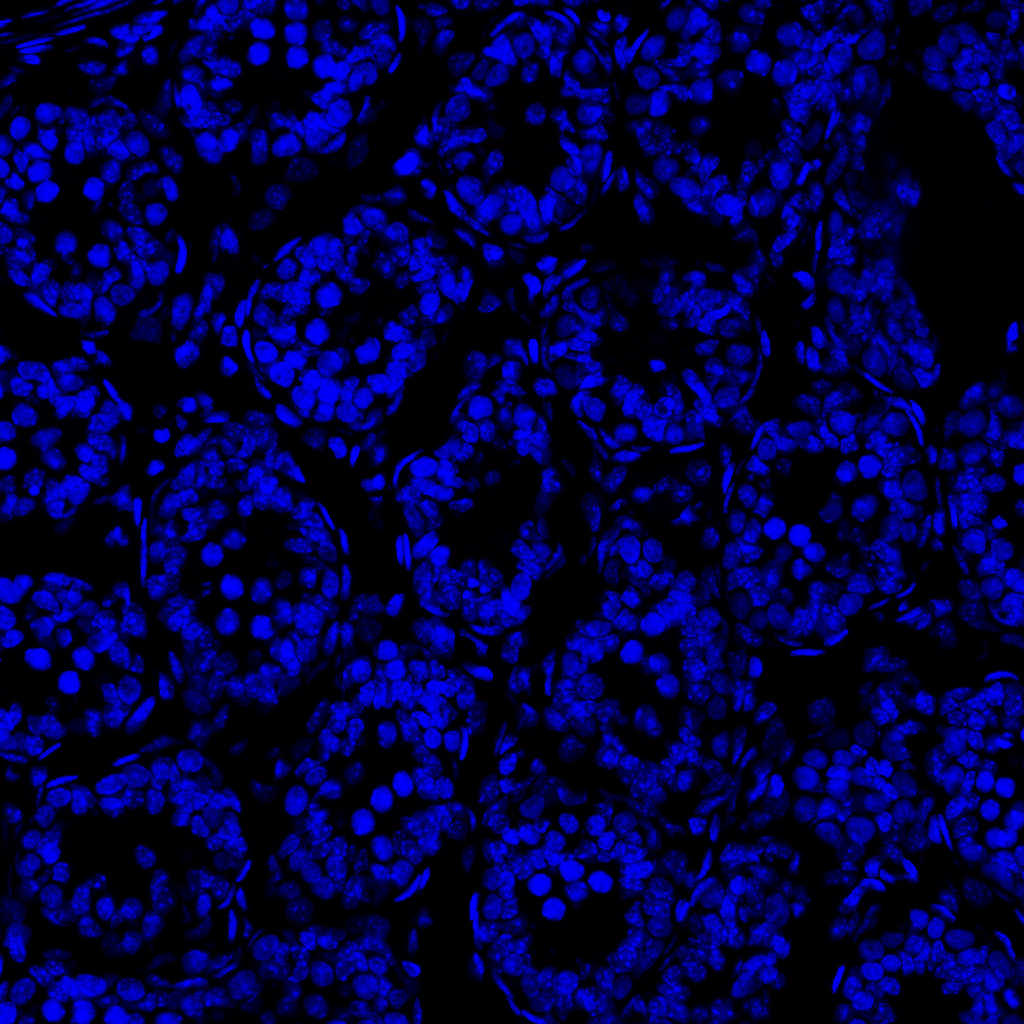

Supplement: Supplementary file 10 — Source data Fig. 3 [file 44318_2024_203_MOESM10_ESM.zip › Figure 3/Figure 3A/cKO-DAPI.jpg]

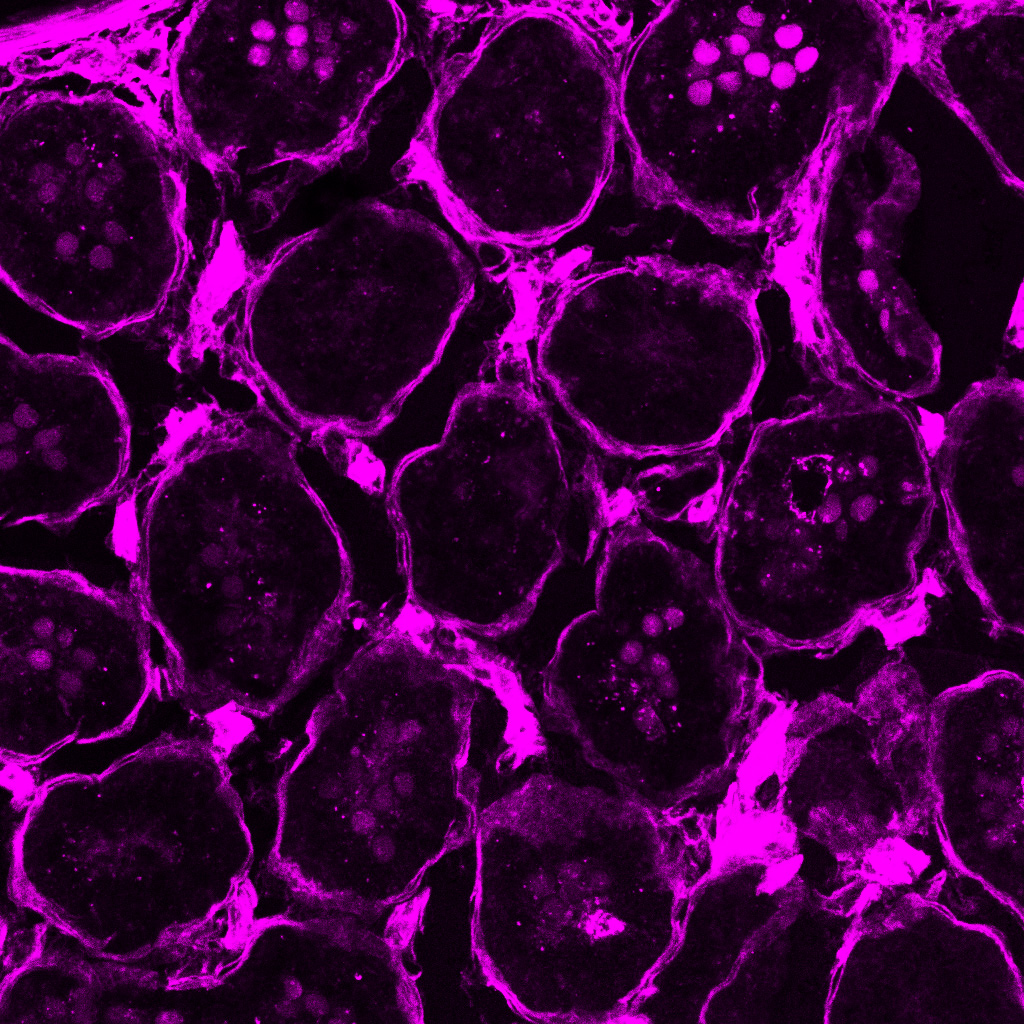

Supplement: Supplementary file 10 — Source data Fig. 3 [file 44318_2024_203_MOESM10_ESM.zip › Figure 3/Figure 3A/cKO-SYCP3.jpg]

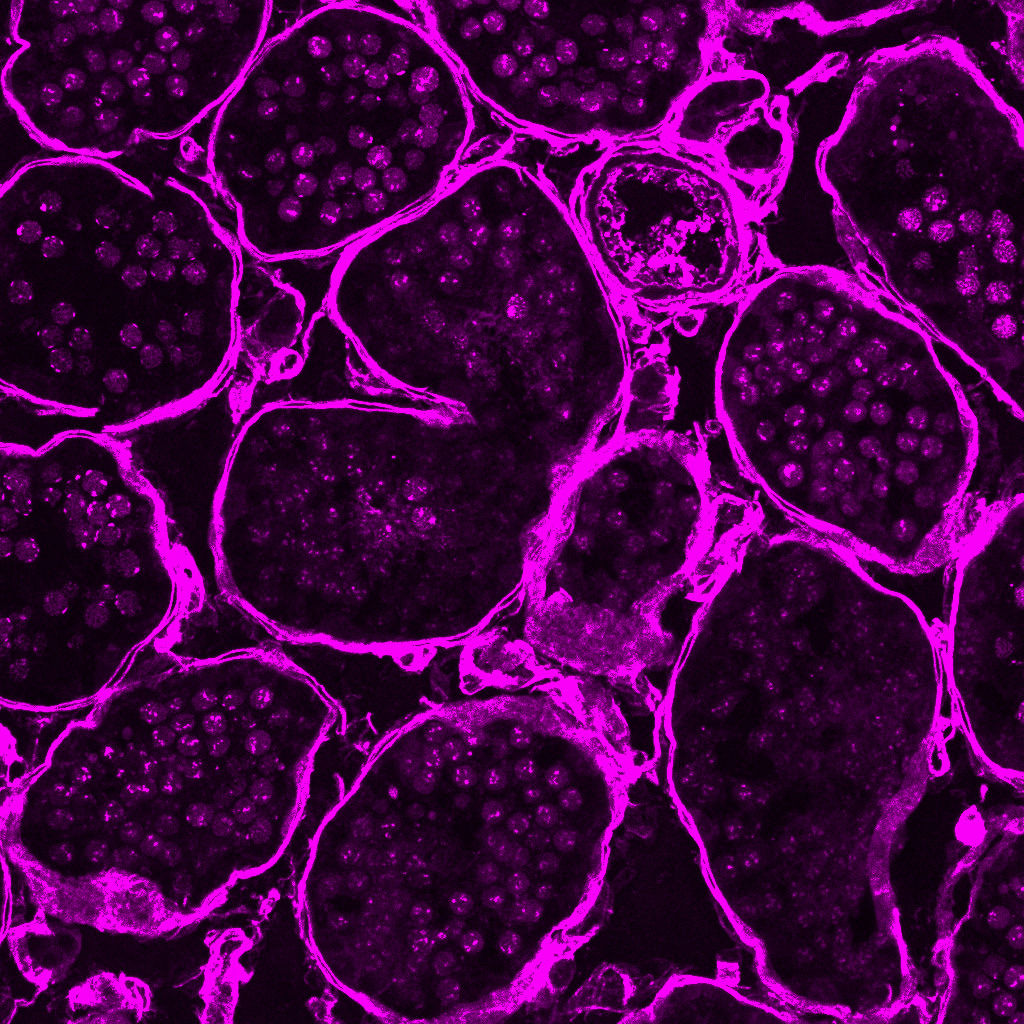

Supplement: Supplementary file 10 — Source data Fig. 3 [file 44318_2024_203_MOESM10_ESM.zip › Figure 3/Figure 3A/Ctrl-SYCP3.jpg]

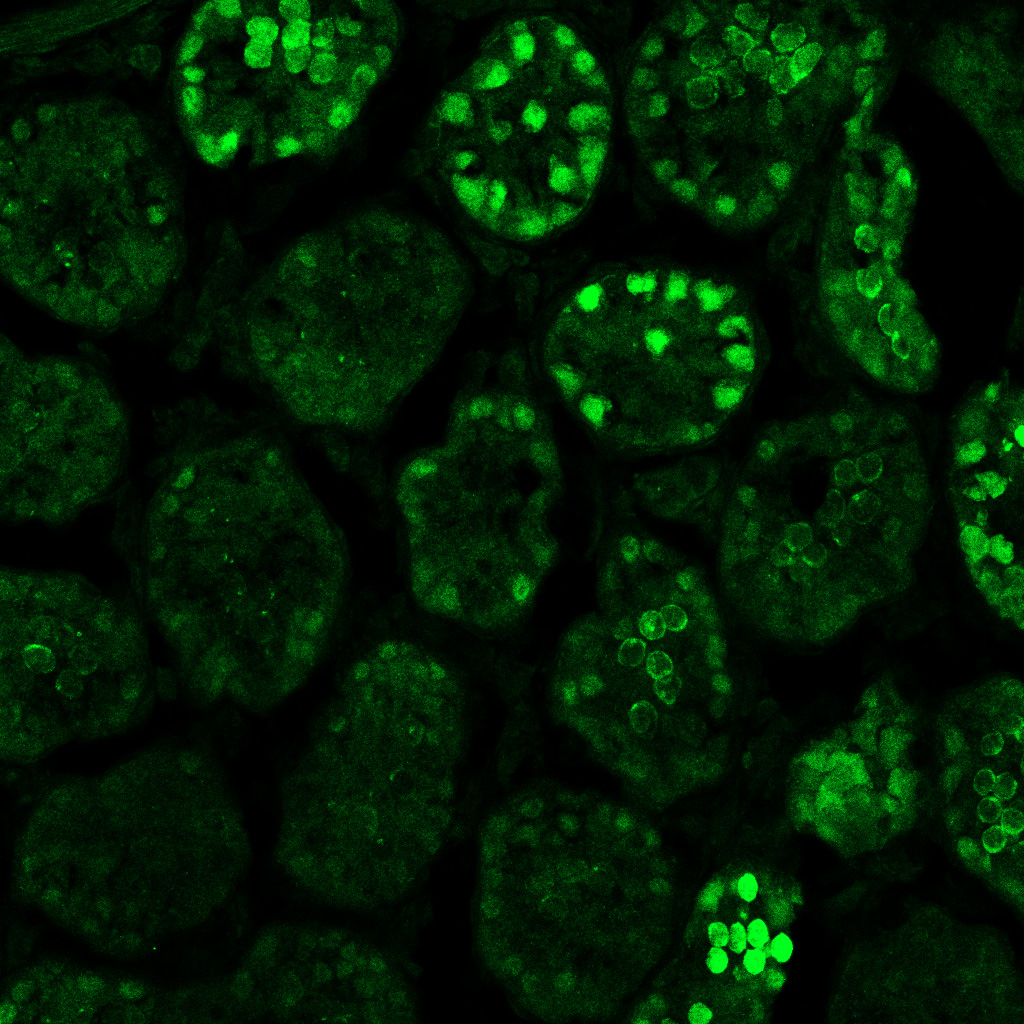

Supplement: Supplementary file 10 — Source data Fig. 3 [file 44318_2024_203_MOESM10_ESM.zip › Figure 3/Figure 3A/cKO-STRA8.jpg]

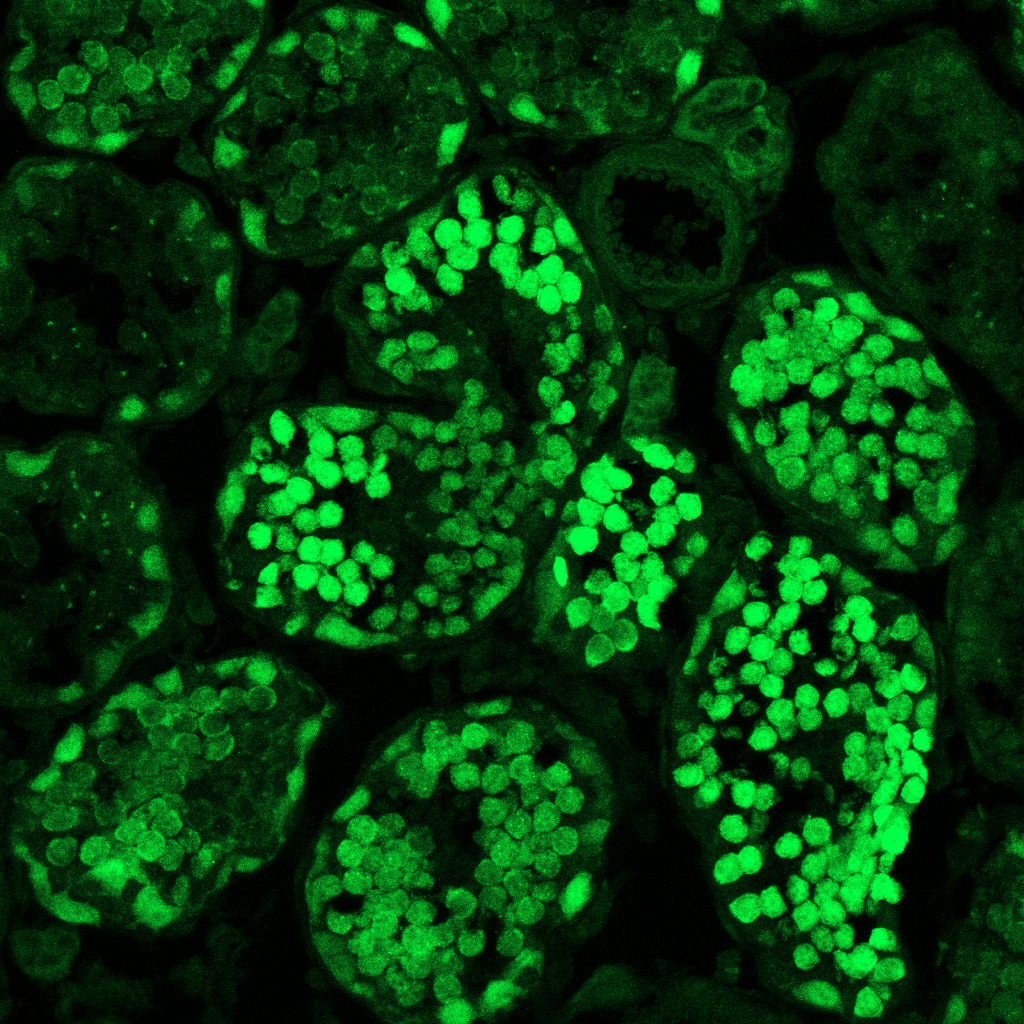

Supplement: Supplementary file 10 — Source data Fig. 3 [file 44318_2024_203_MOESM10_ESM.zip › Figure 3/Figure 3A/Ctrl-STRA8.jpg]

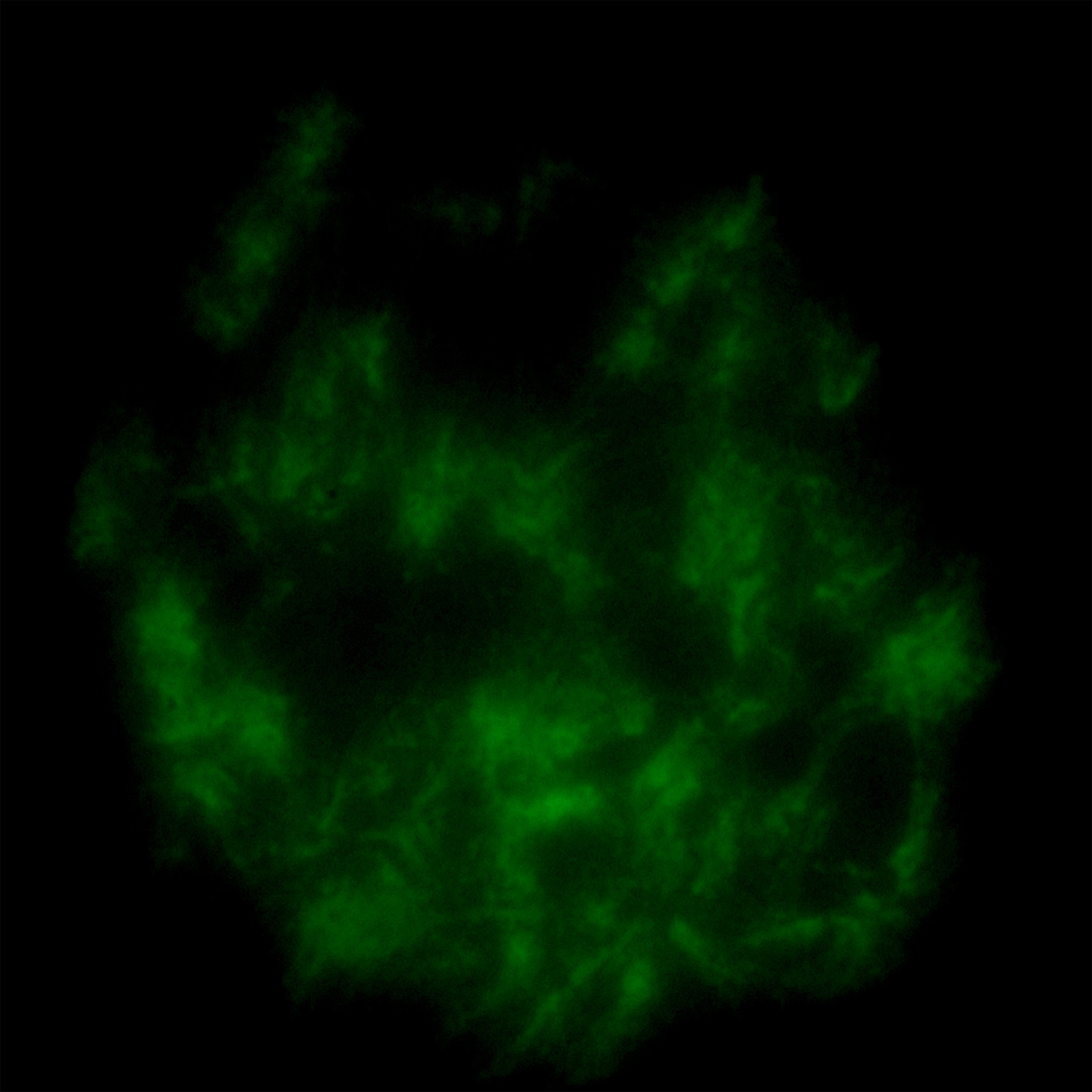

Supplement: Supplementary file 10 — Source data Fig. 3 [file 44318_2024_203_MOESM10_ESM.zip › Figure 3/Figure 3F/cKO Zyg-ii ╬│H2AX.jpg]

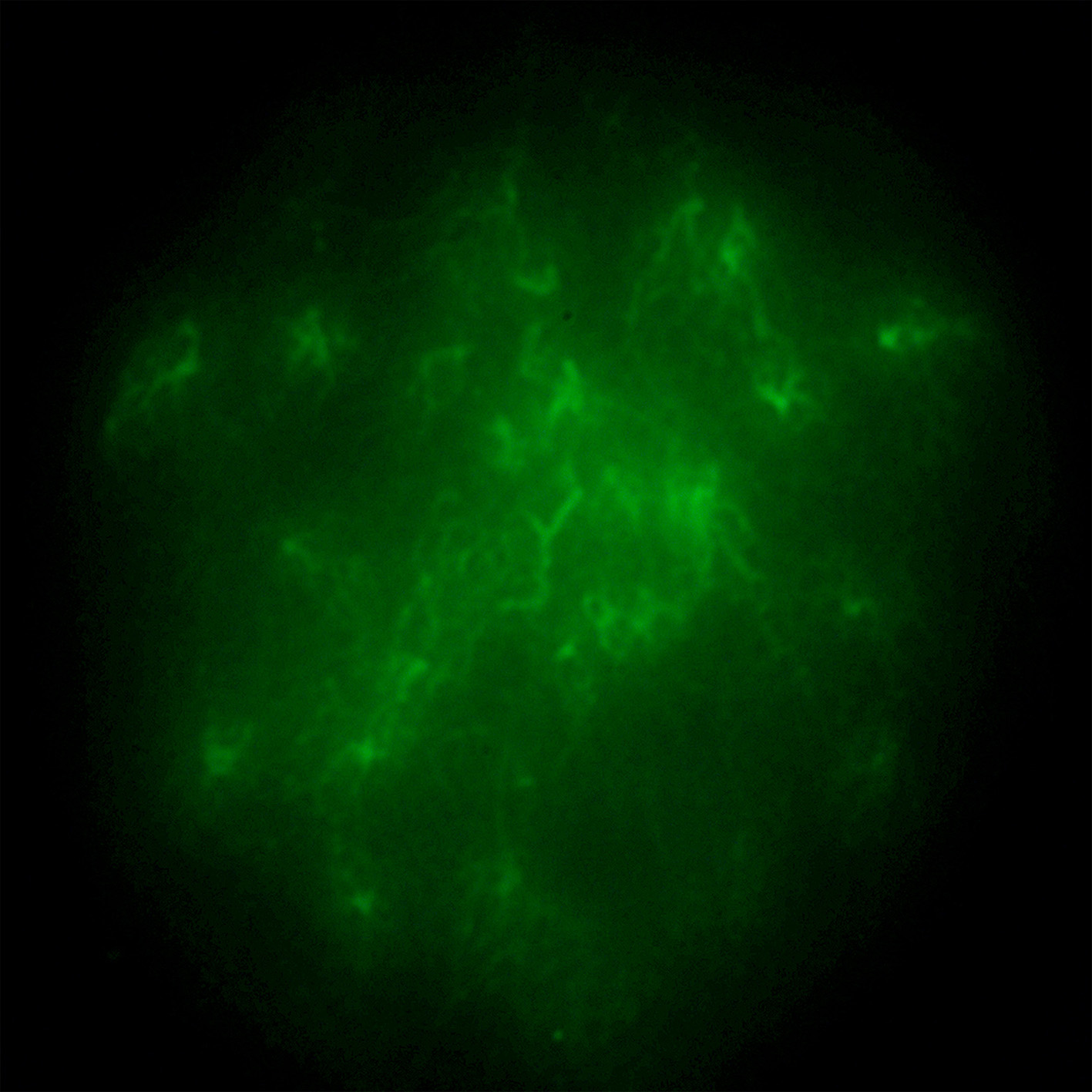

Supplement: Supplementary file 10 — Source data Fig. 3 [file 44318_2024_203_MOESM10_ESM.zip › Figure 3/Figure 3F/cKO Lep-╬│H2AX.jpg]

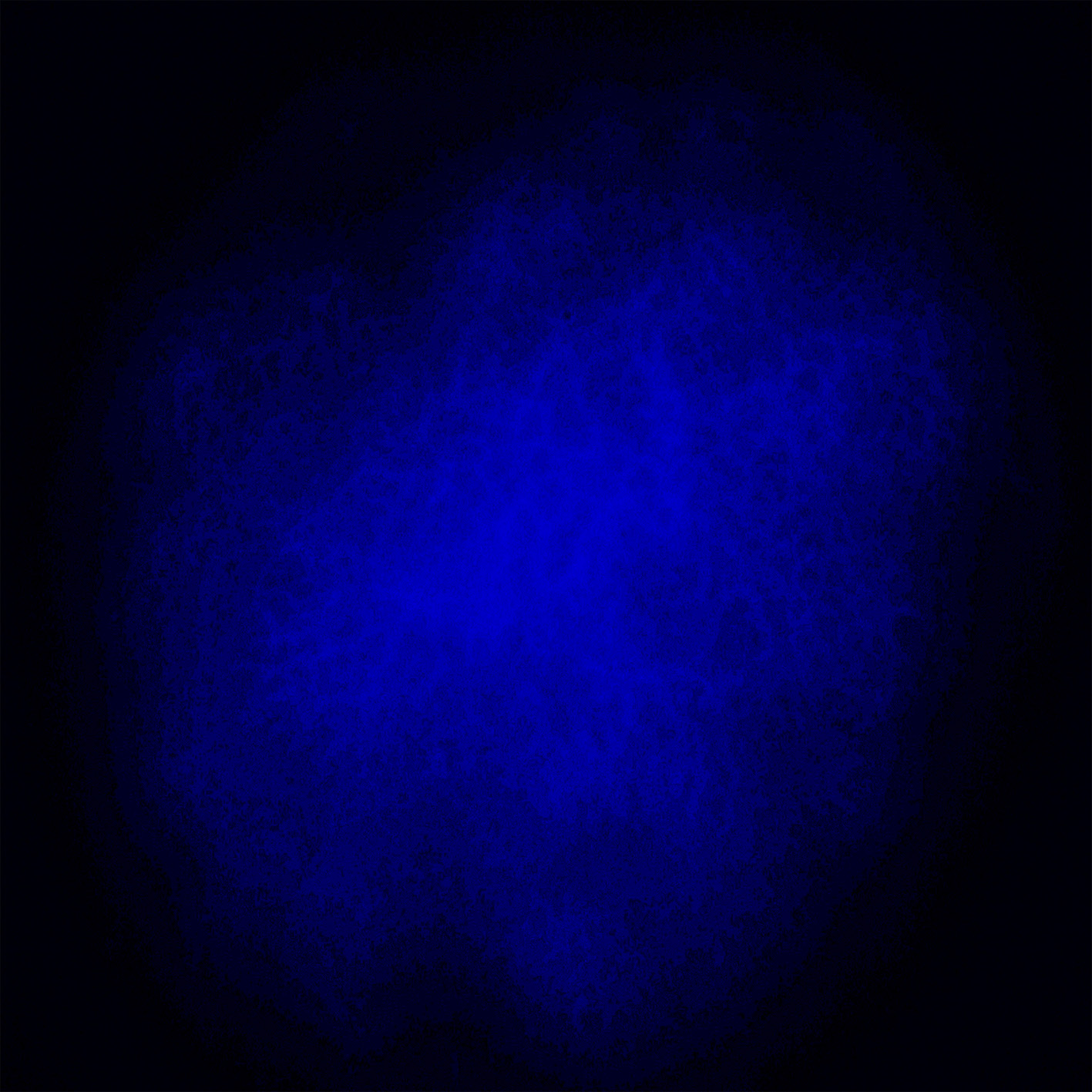

Supplement: Supplementary file 10 — Source data Fig. 3 [file 44318_2024_203_MOESM10_ESM.zip › Figure 3/Figure 3F/cKO Lep-DAPI.jpg]

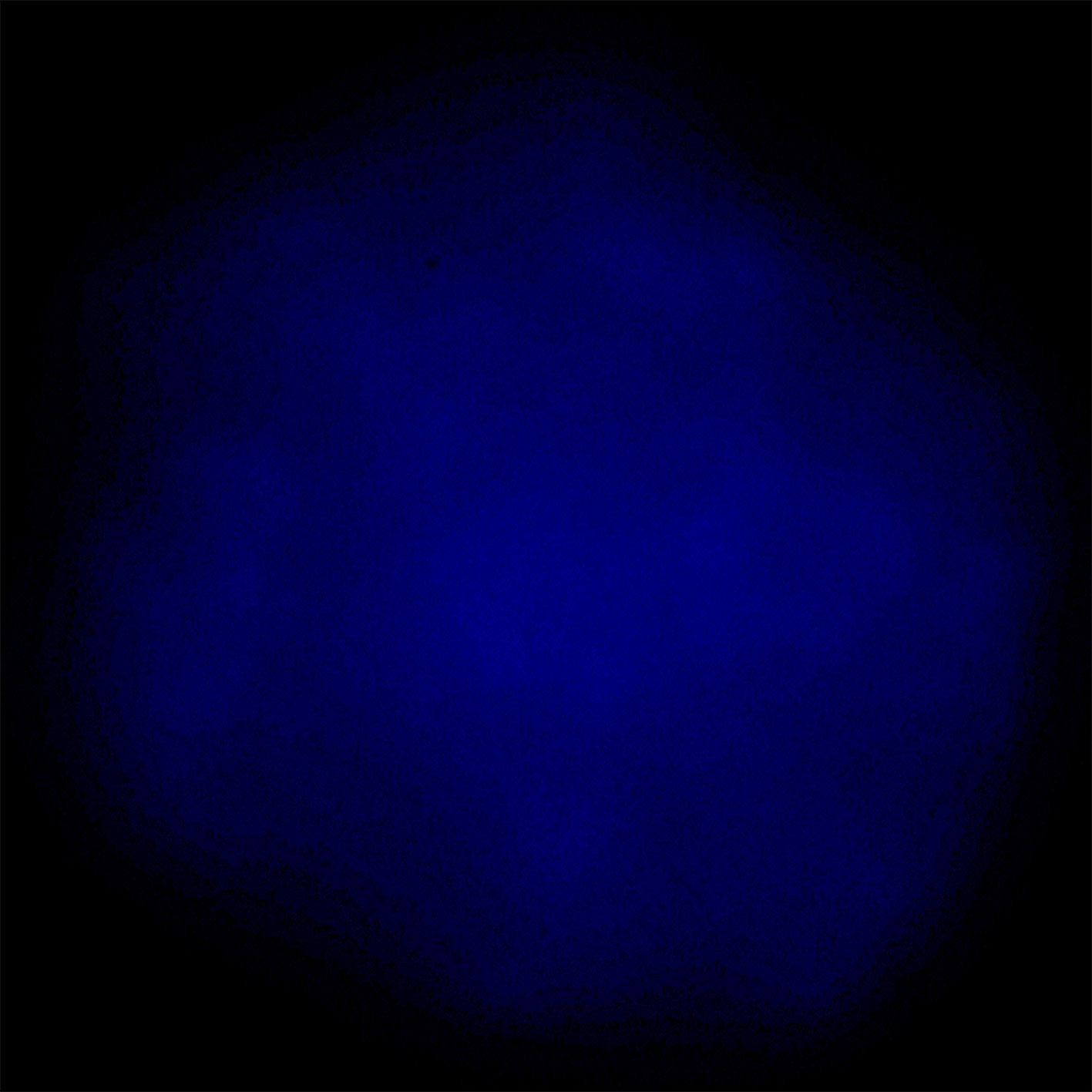

Supplement: Supplementary file 10 — Source data Fig. 3 [file 44318_2024_203_MOESM10_ESM.zip › Figure 3/Figure 3F/Ctrl Zyg-DAPI.jpg]

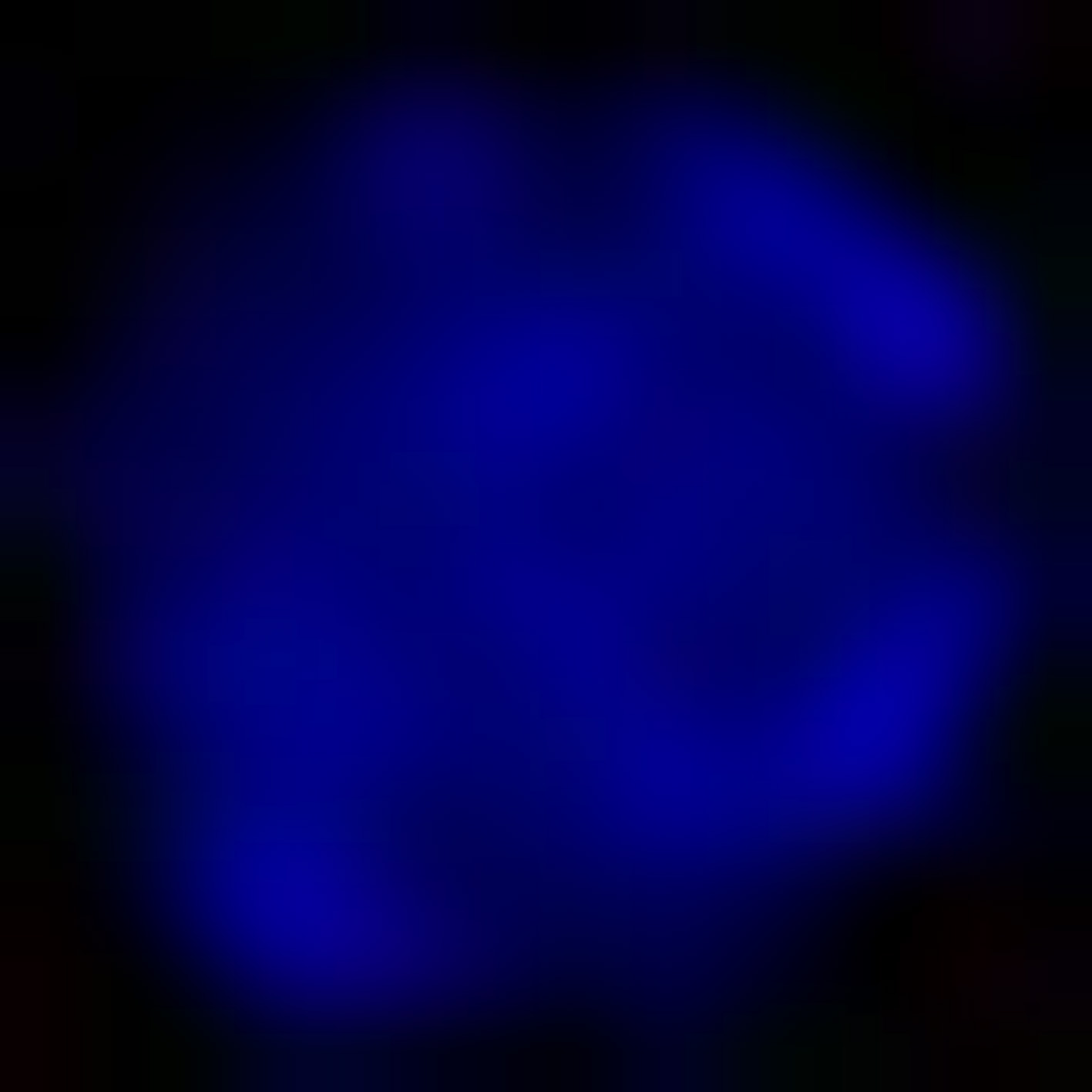

Supplement: Supplementary file 10 — Source data Fig. 3 [file 44318_2024_203_MOESM10_ESM.zip › Figure 3/Figure 3F/Ctrl Lep-DAPI.jpg]

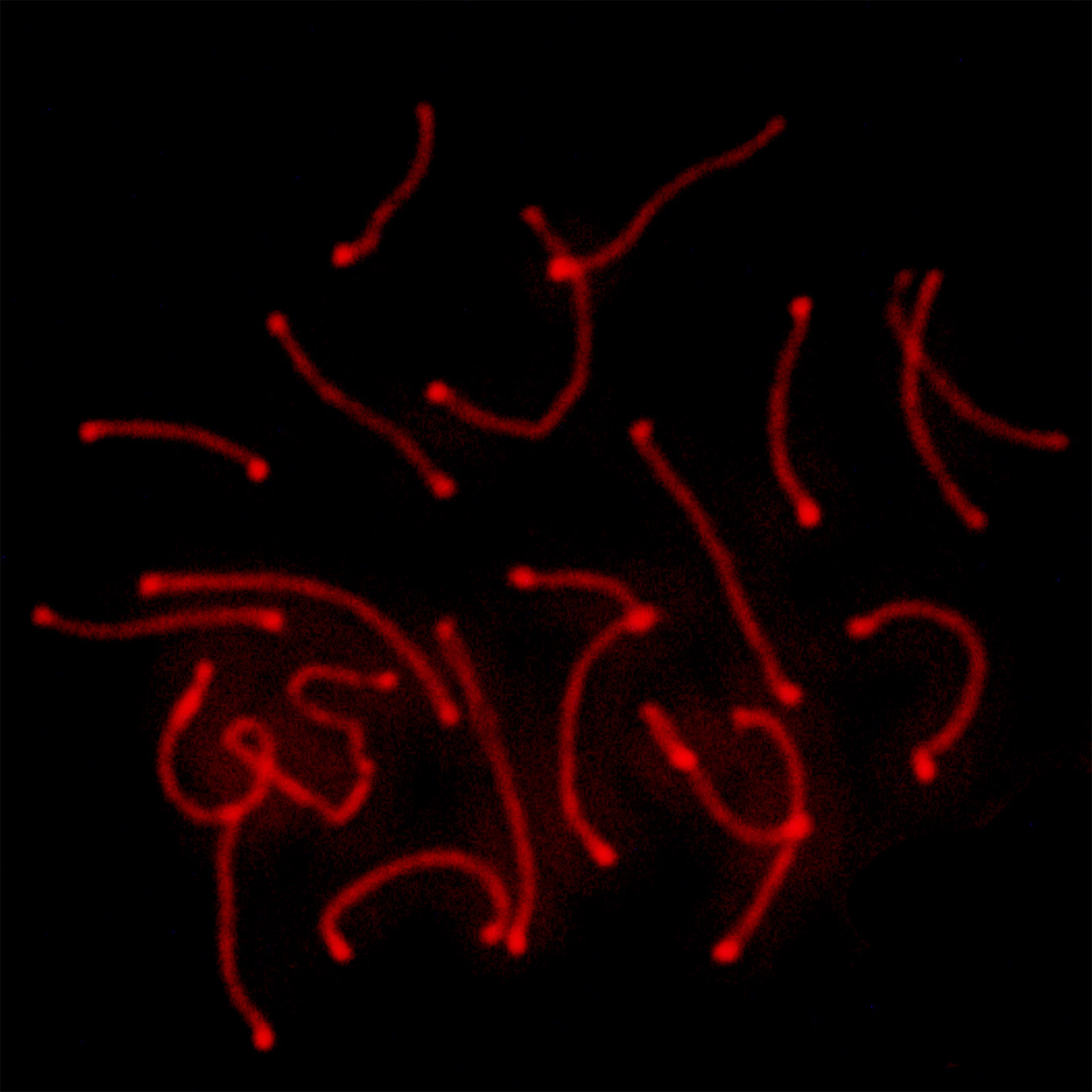

Supplement: Supplementary file 10 — Source data Fig. 3 [file 44318_2024_203_MOESM10_ESM.zip › Figure 3/Figure 3F/Ctrl Pac-SYCP3.jpg]

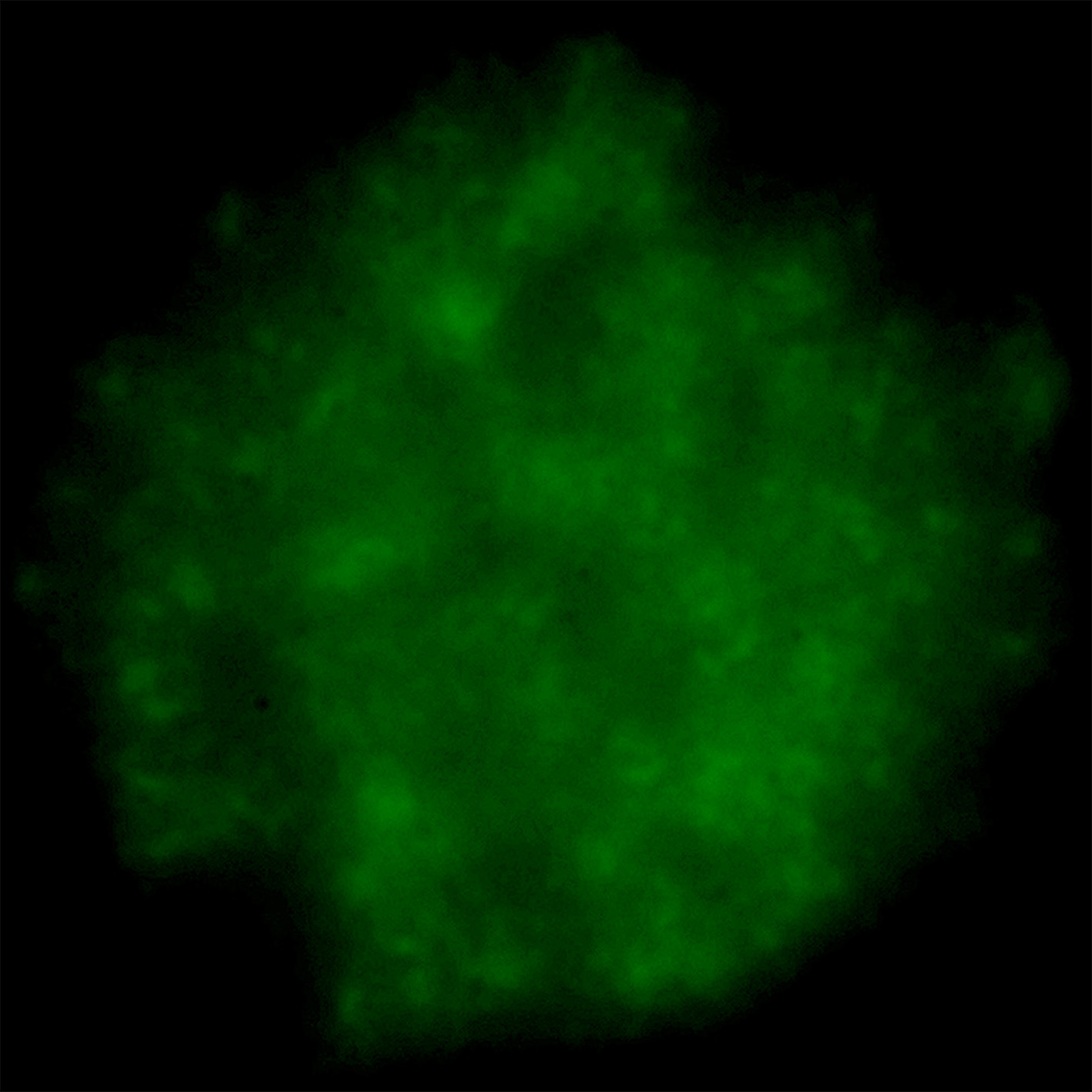

Supplement: Supplementary file 10 — Source data Fig. 3 [file 44318_2024_203_MOESM10_ESM.zip › Figure 3/Figure 3F/Ctrl Lep-╬│H2AX.jpg]

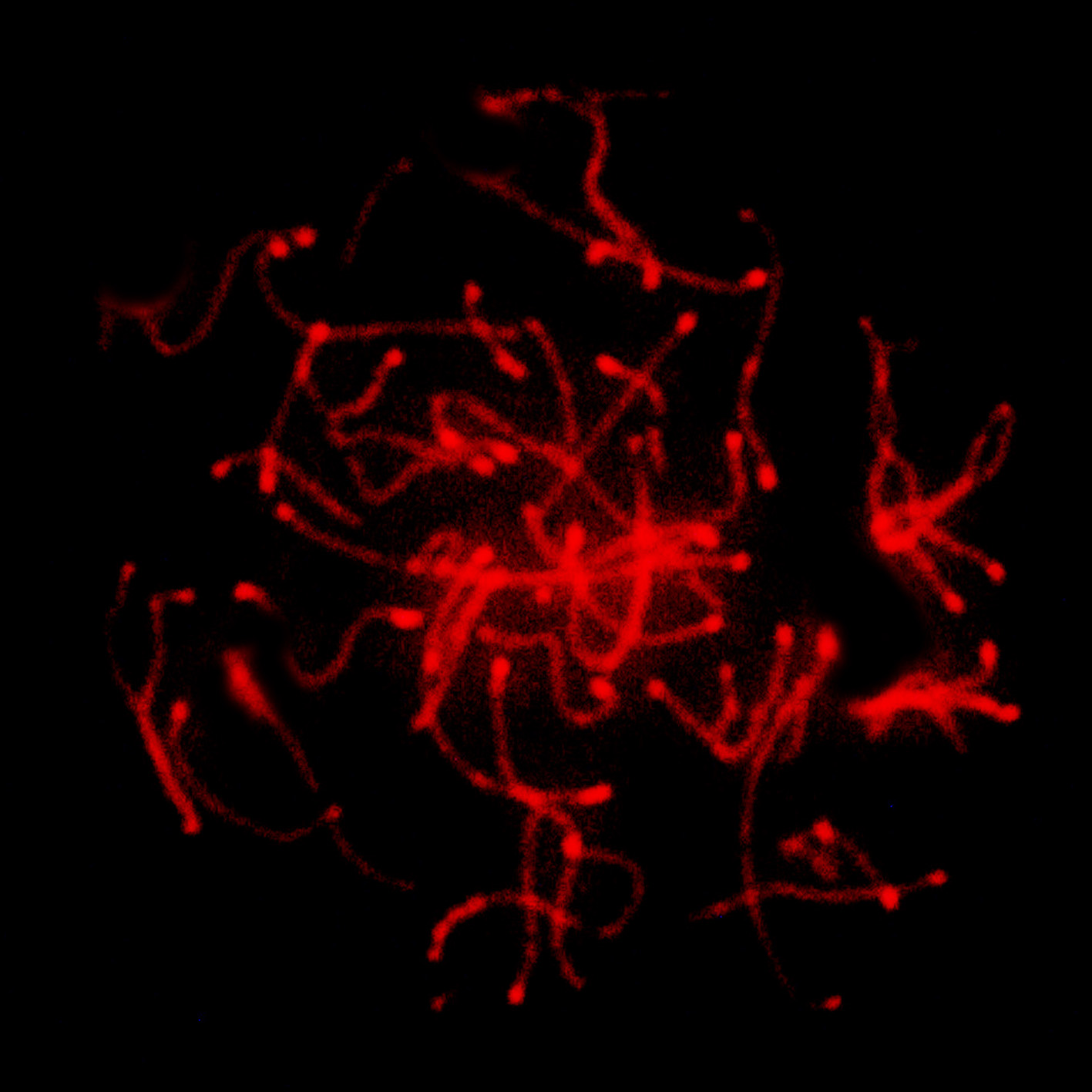

Supplement: Supplementary file 10 — Source data Fig. 3 [file 44318_2024_203_MOESM10_ESM.zip › Figure 3/Figure 3F/Ctrl Zyg-SYCP3.jpg]

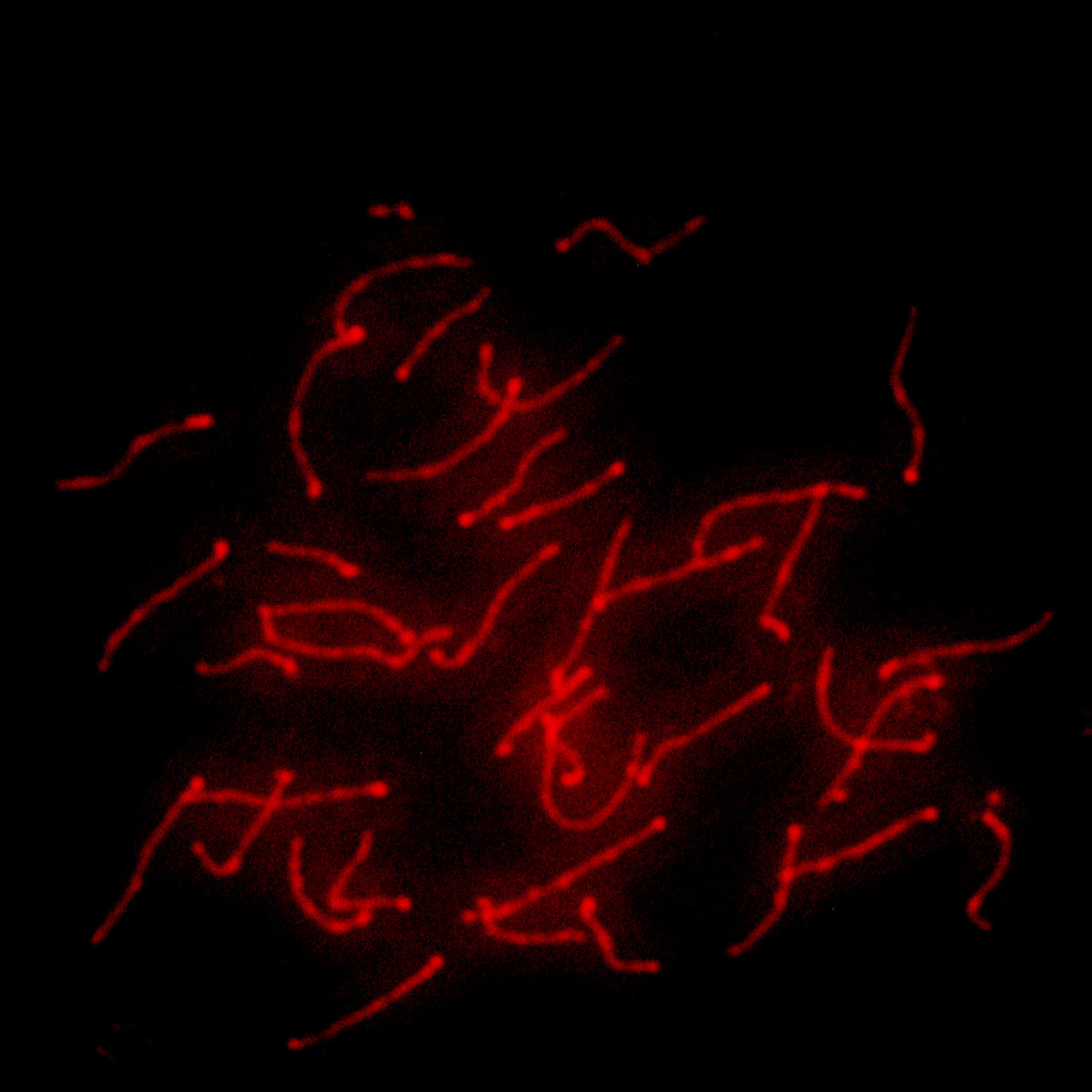

Supplement: Supplementary file 10 — Source data Fig. 3 [file 44318_2024_203_MOESM10_ESM.zip › Figure 3/Figure 3F/cKO Zyg-i SYCP3.jpg]

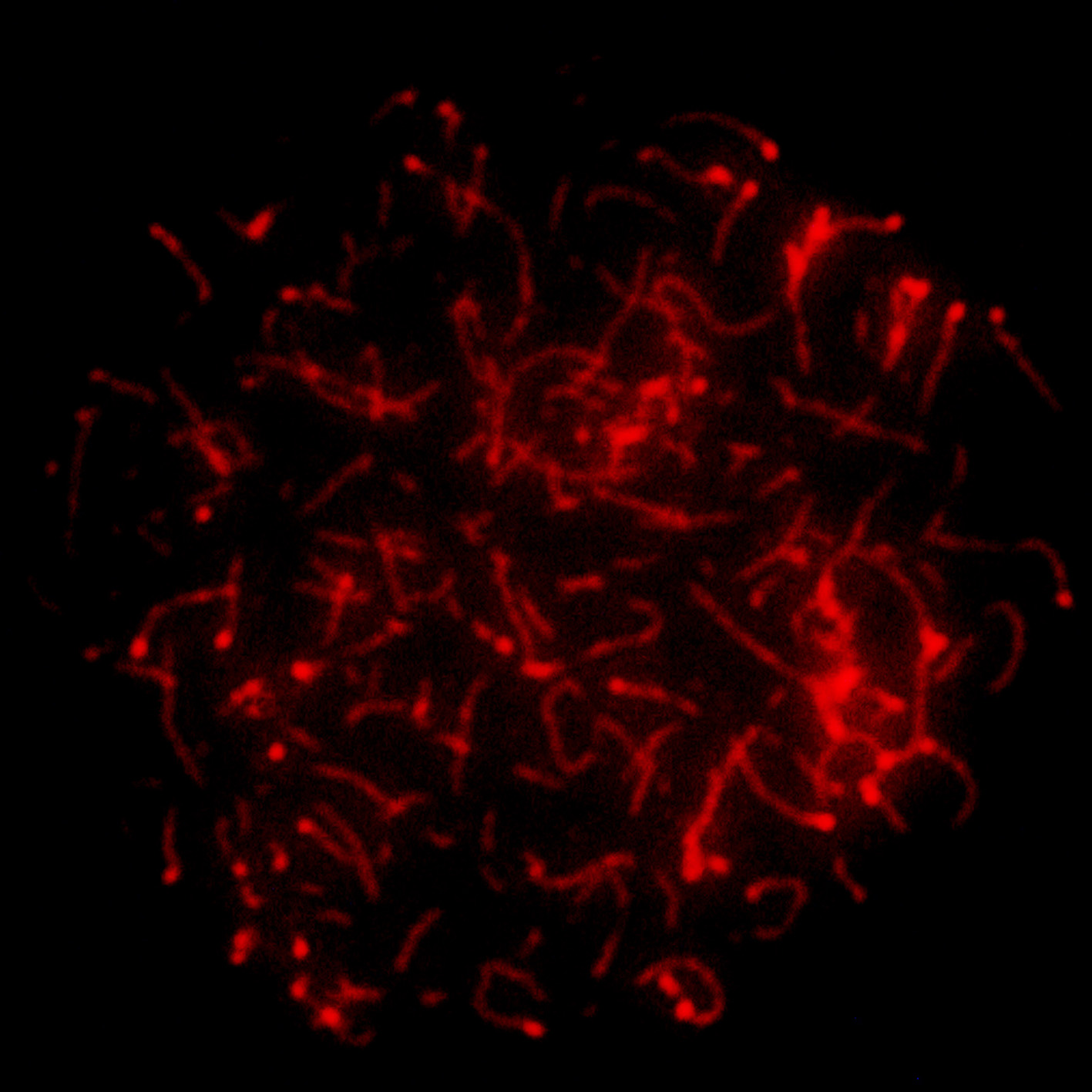

Supplement: Supplementary file 10 — Source data Fig. 3 [file 44318_2024_203_MOESM10_ESM.zip › Figure 3/Figure 3F/Ctrl Lep-SYCP3.jpg]

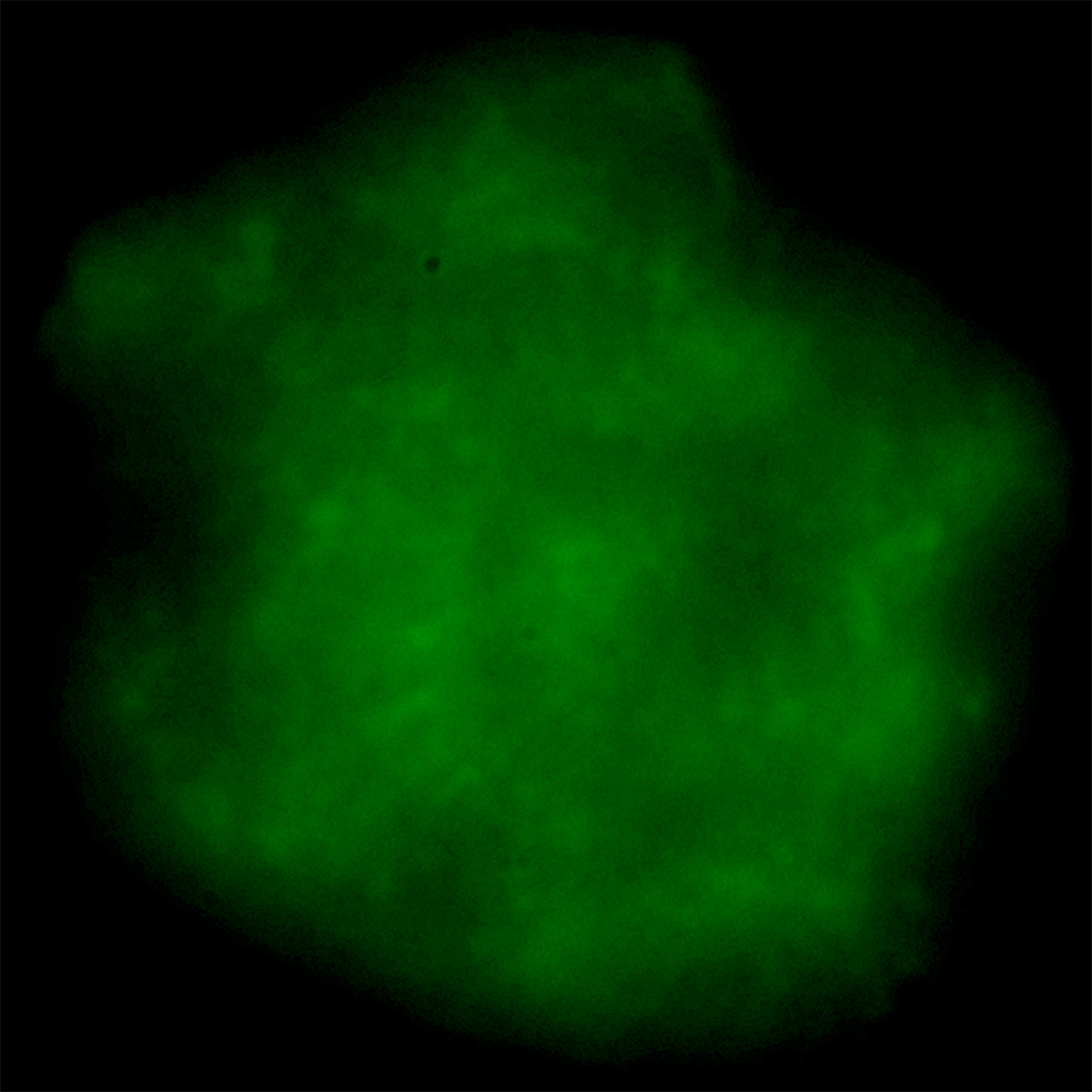

Supplement: Supplementary file 10 — Source data Fig. 3 [file 44318_2024_203_MOESM10_ESM.zip › Figure 3/Figure 3F/Ctrl Zyg-╬│H2AX.jpg]

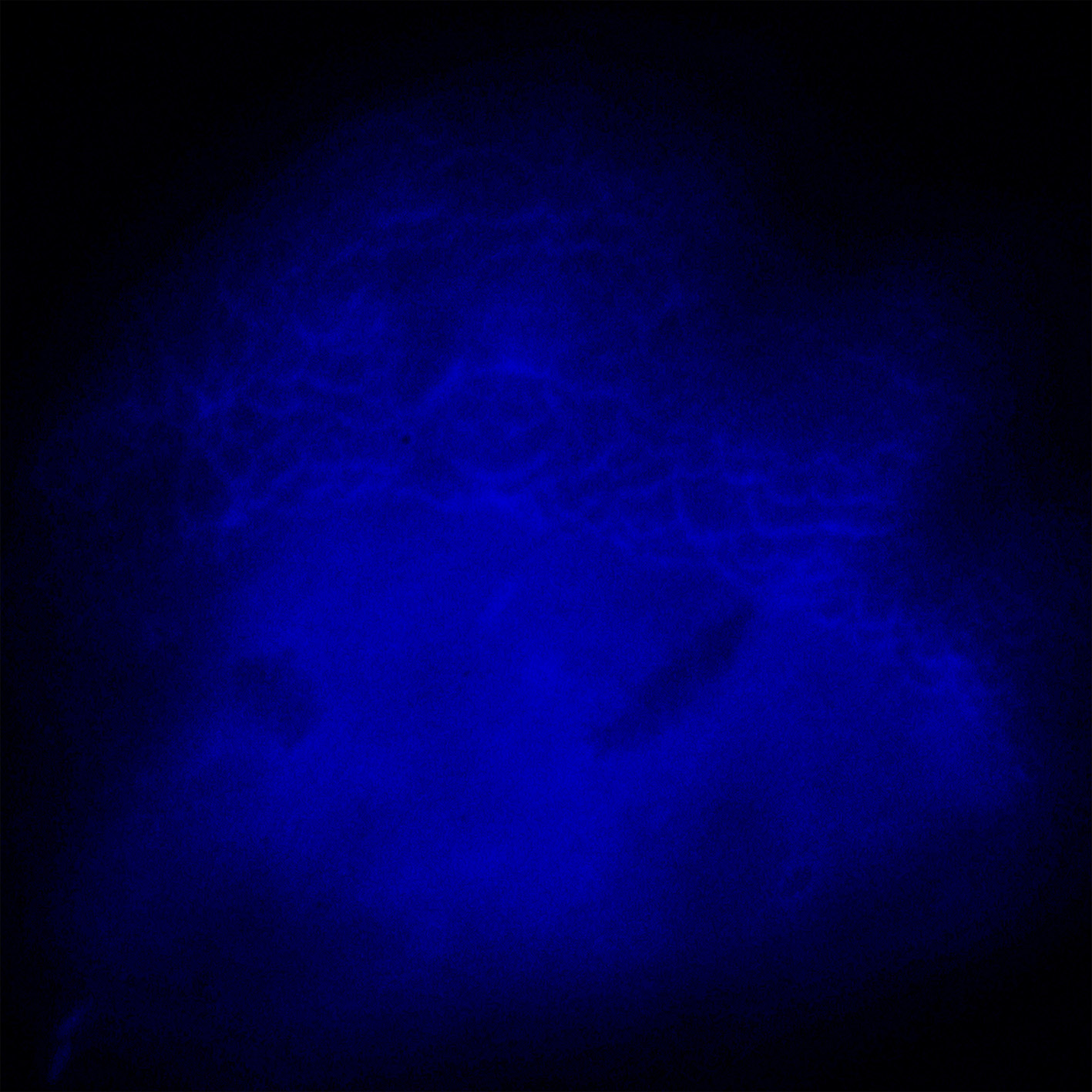

Supplement: Supplementary file 10 — Source data Fig. 3 [file 44318_2024_203_MOESM10_ESM.zip › Figure 3/Figure 3F/cKO Zyg-i DAPI.jpg]

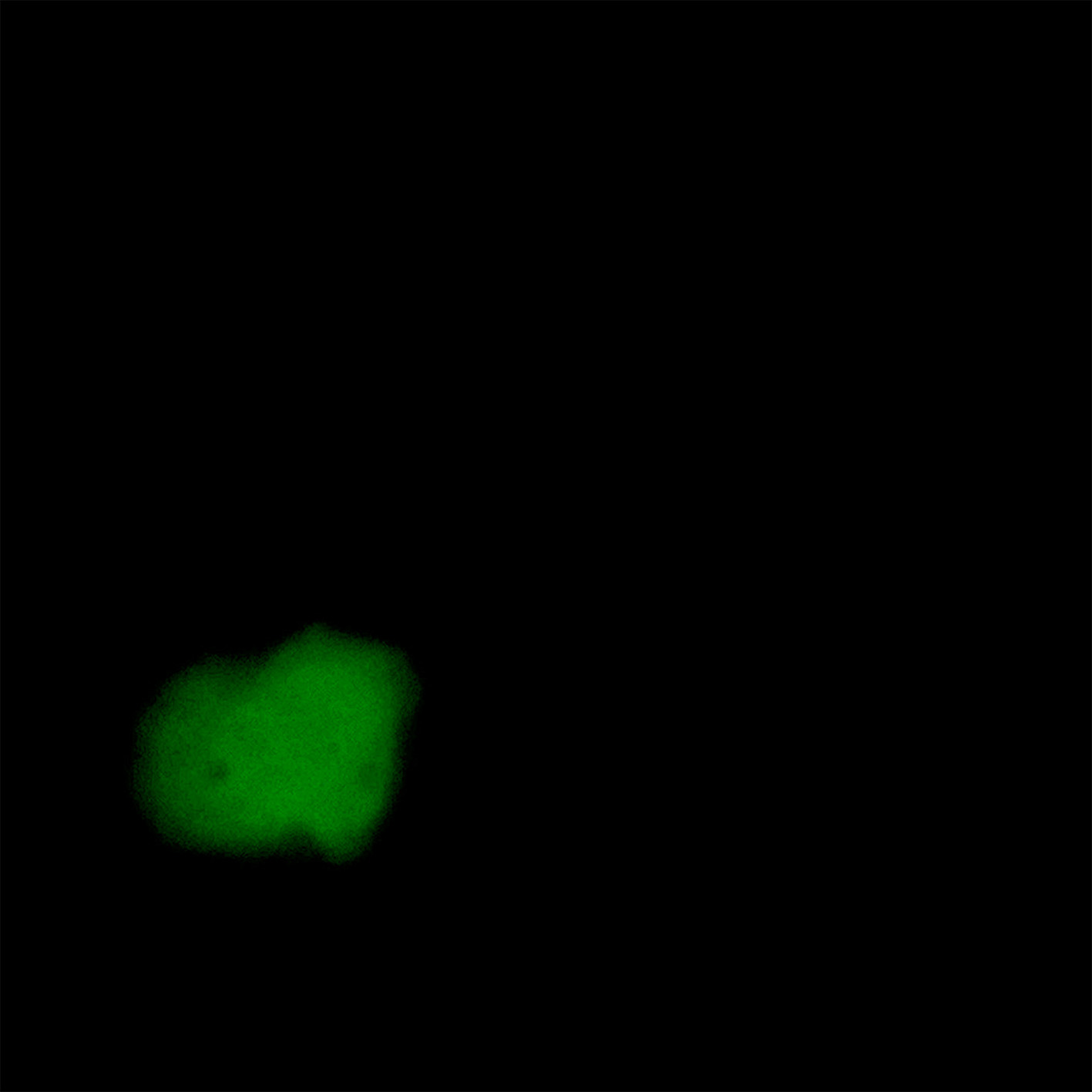

Supplement: Supplementary file 10 — Source data Fig. 3 [file 44318_2024_203_MOESM10_ESM.zip › Figure 3/Figure 3F/Ctrl Pac-╬│H2AX.jpg]

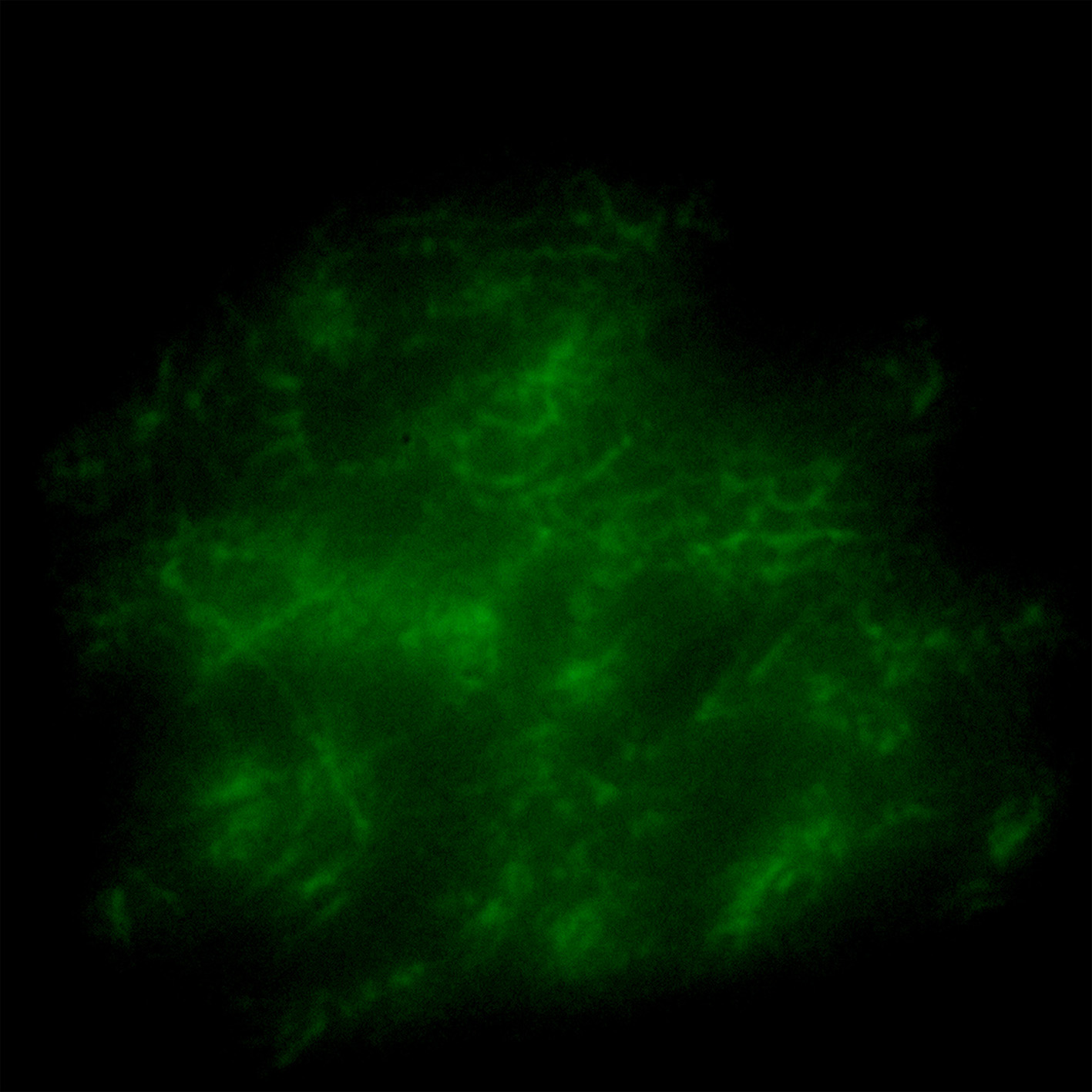

Supplement: Supplementary file 10 — Source data Fig. 3 [file 44318_2024_203_MOESM10_ESM.zip › Figure 3/Figure 3F/cKO Zyg-i ╬│H2AX.jpg]

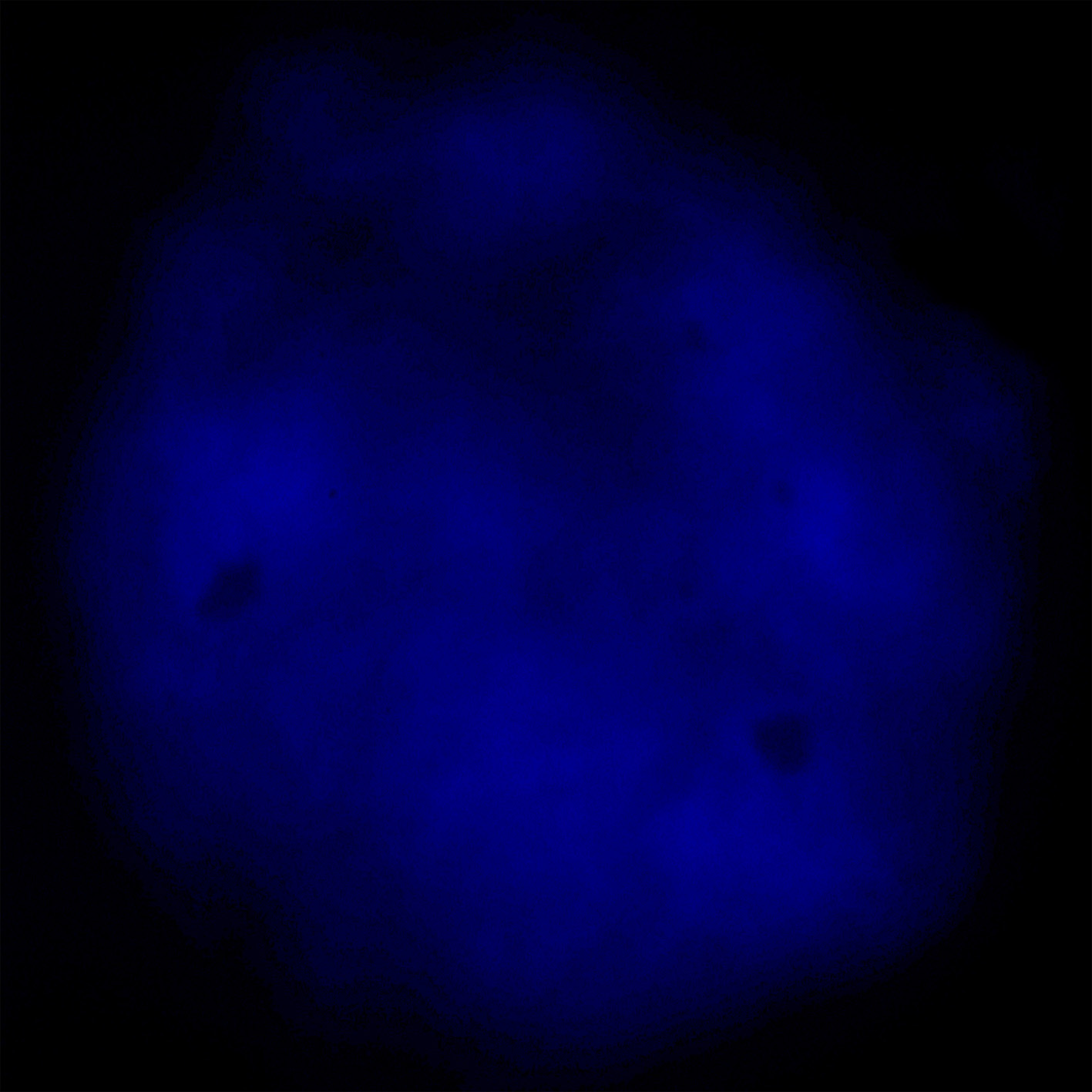

Supplement: Supplementary file 10 — Source data Fig. 3 [file 44318_2024_203_MOESM10_ESM.zip › Figure 3/Figure 3F/cKO Zyg-ii DAPI.jpg]

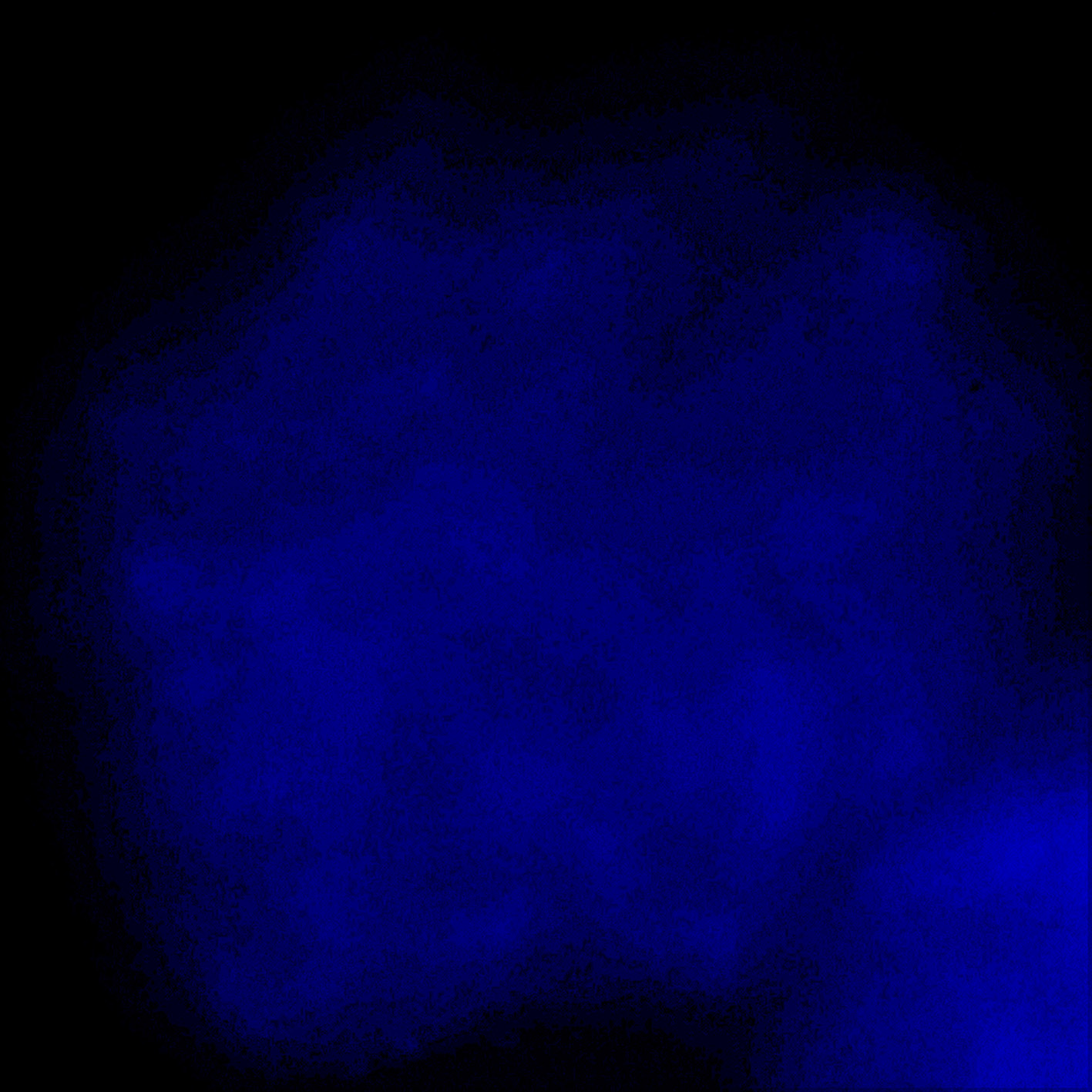

Supplement: Supplementary file 10 — Source data Fig. 3 [file 44318_2024_203_MOESM10_ESM.zip › Figure 3/Figure 3F/Ctrl Pac-DAPI.jpg]

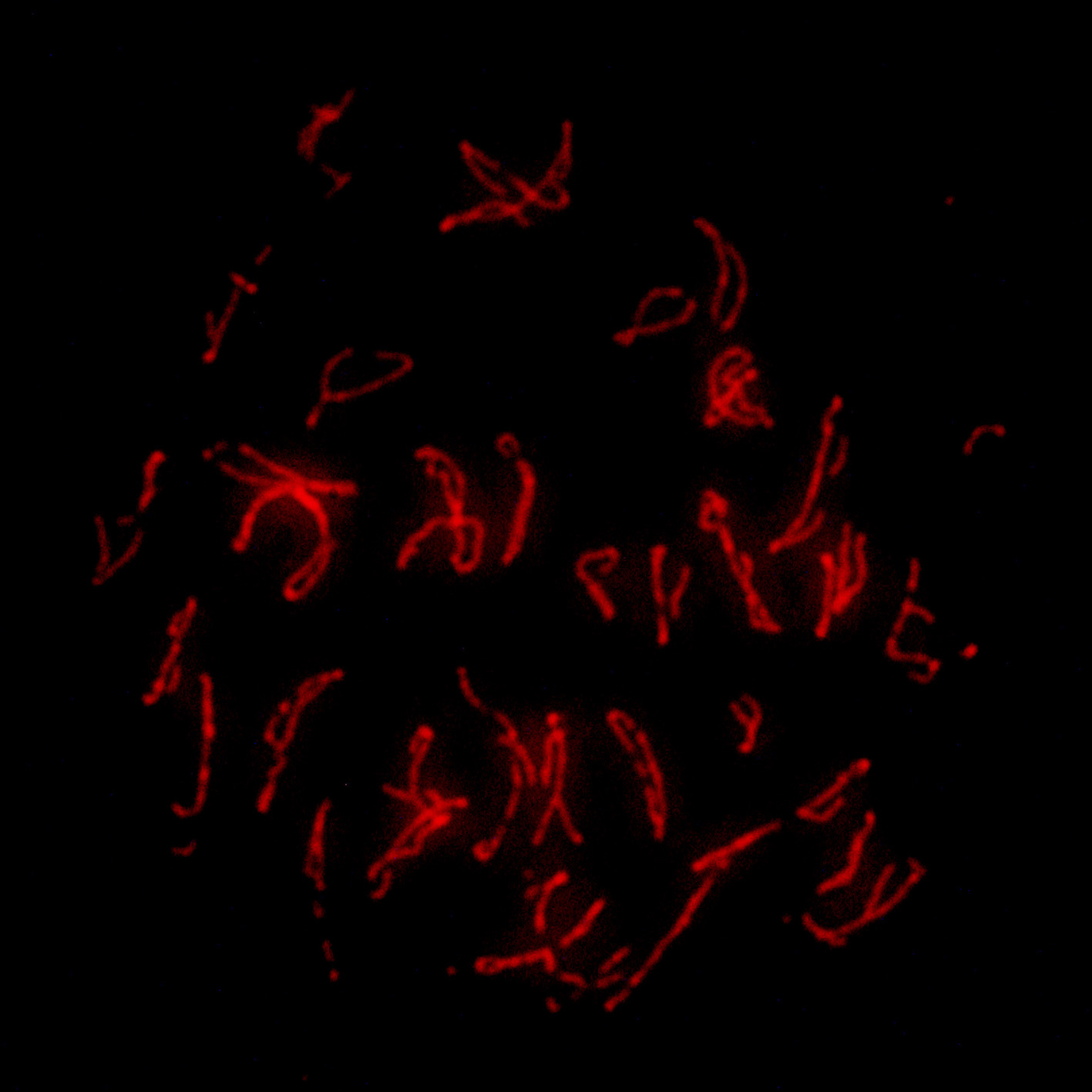

Supplement: Supplementary file 10 — Source data Fig. 3 [file 44318_2024_203_MOESM10_ESM.zip › Figure 3/Figure 3F/cKO Zyg-ii SYCP3.jpg]

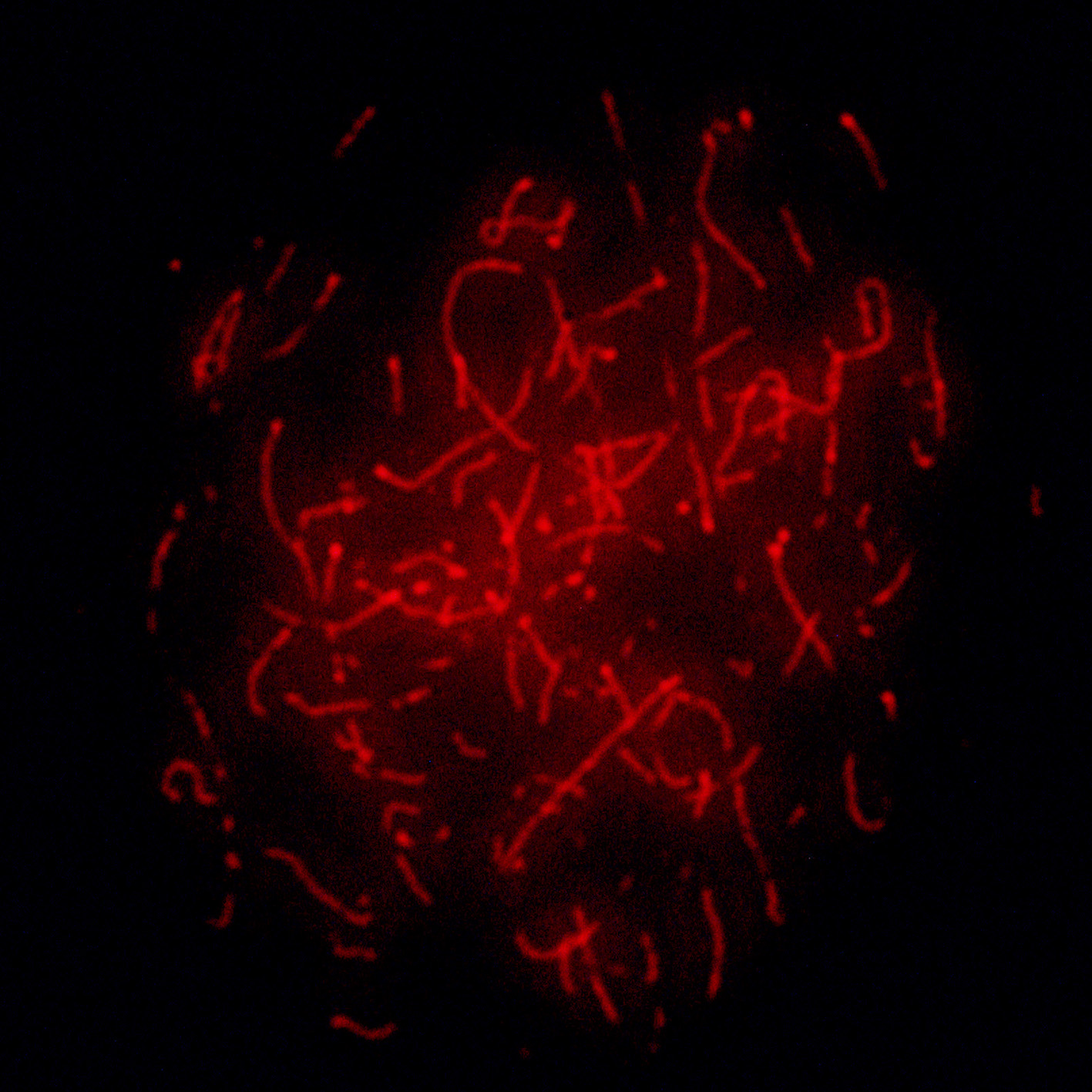

Supplement: Supplementary file 10 — Source data Fig. 3 [file 44318_2024_203_MOESM10_ESM.zip › Figure 3/Figure 3F/cKO Lep-SYCP3.jpg]

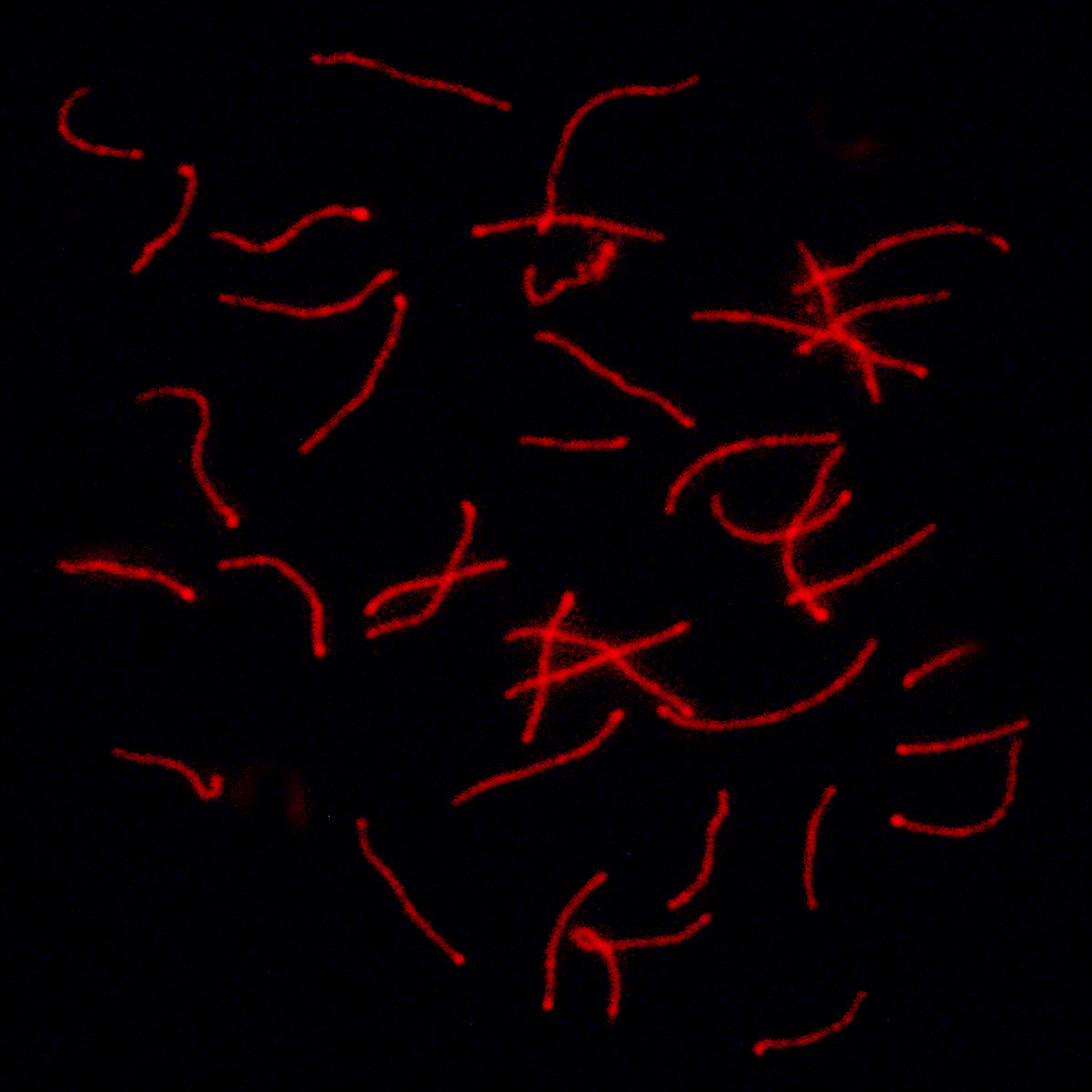

Supplement: Supplementary file 10 — Source data Fig. 3 [file 44318_2024_203_MOESM10_ESM.zip › Figure 3/Figure 3G/cKO-i-SYCP3.jpg]

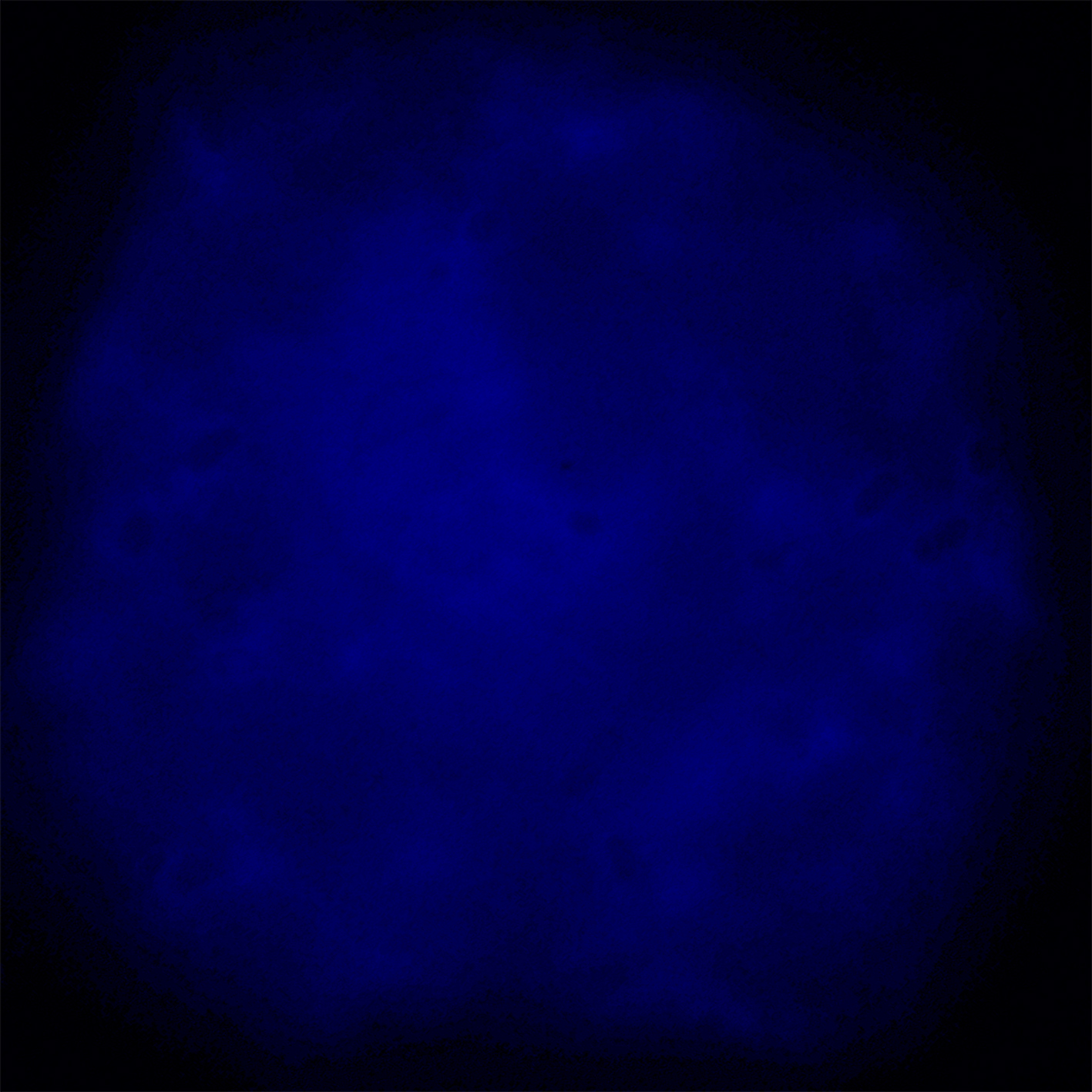

Supplement: Supplementary file 10 — Source data Fig. 3 [file 44318_2024_203_MOESM10_ESM.zip › Figure 3/Figure 3G/Ctrl-DAPI.jpg]

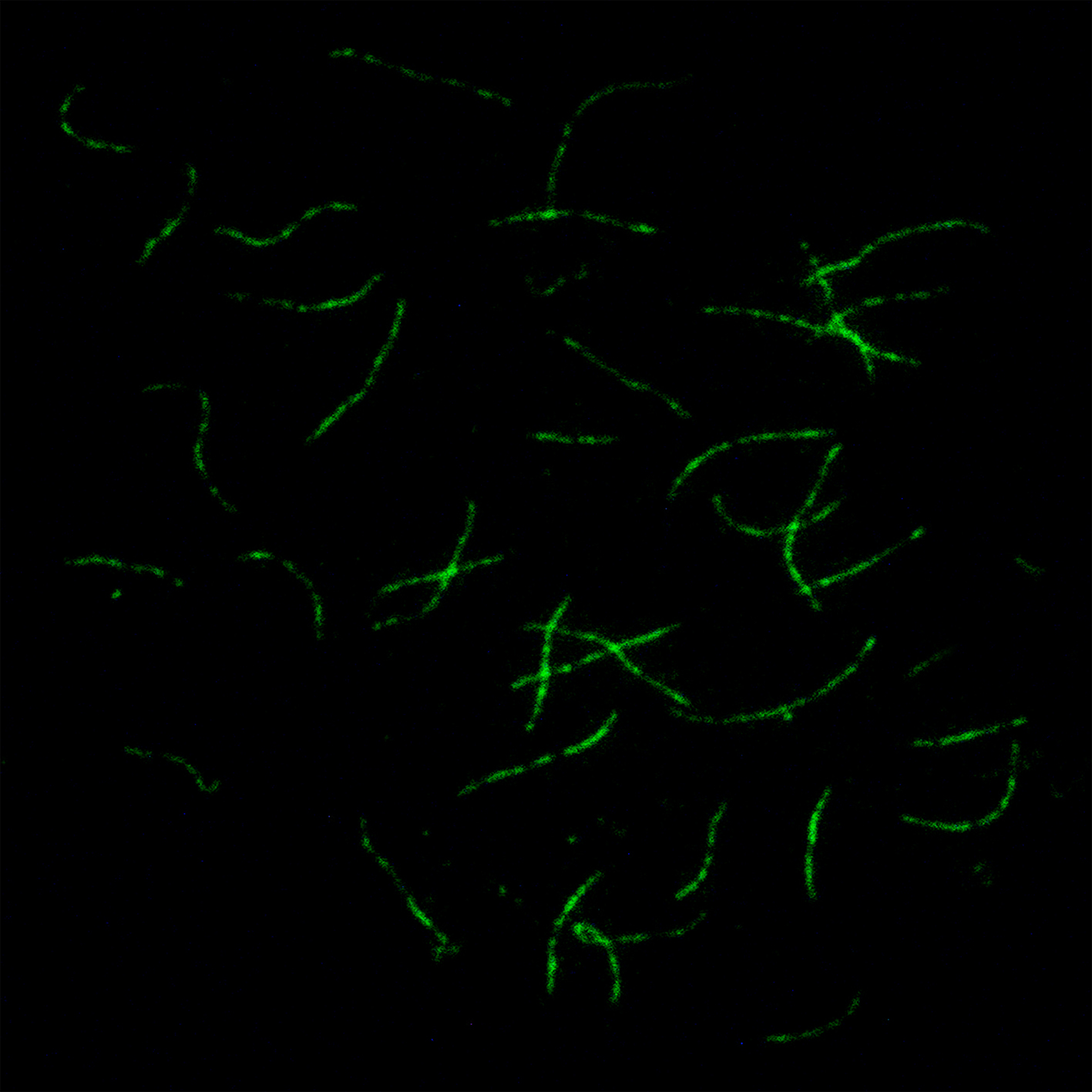

Supplement: Supplementary file 10 — Source data Fig. 3 [file 44318_2024_203_MOESM10_ESM.zip › Figure 3/Figure 3G/cKO-i-SYCP1.jpg]

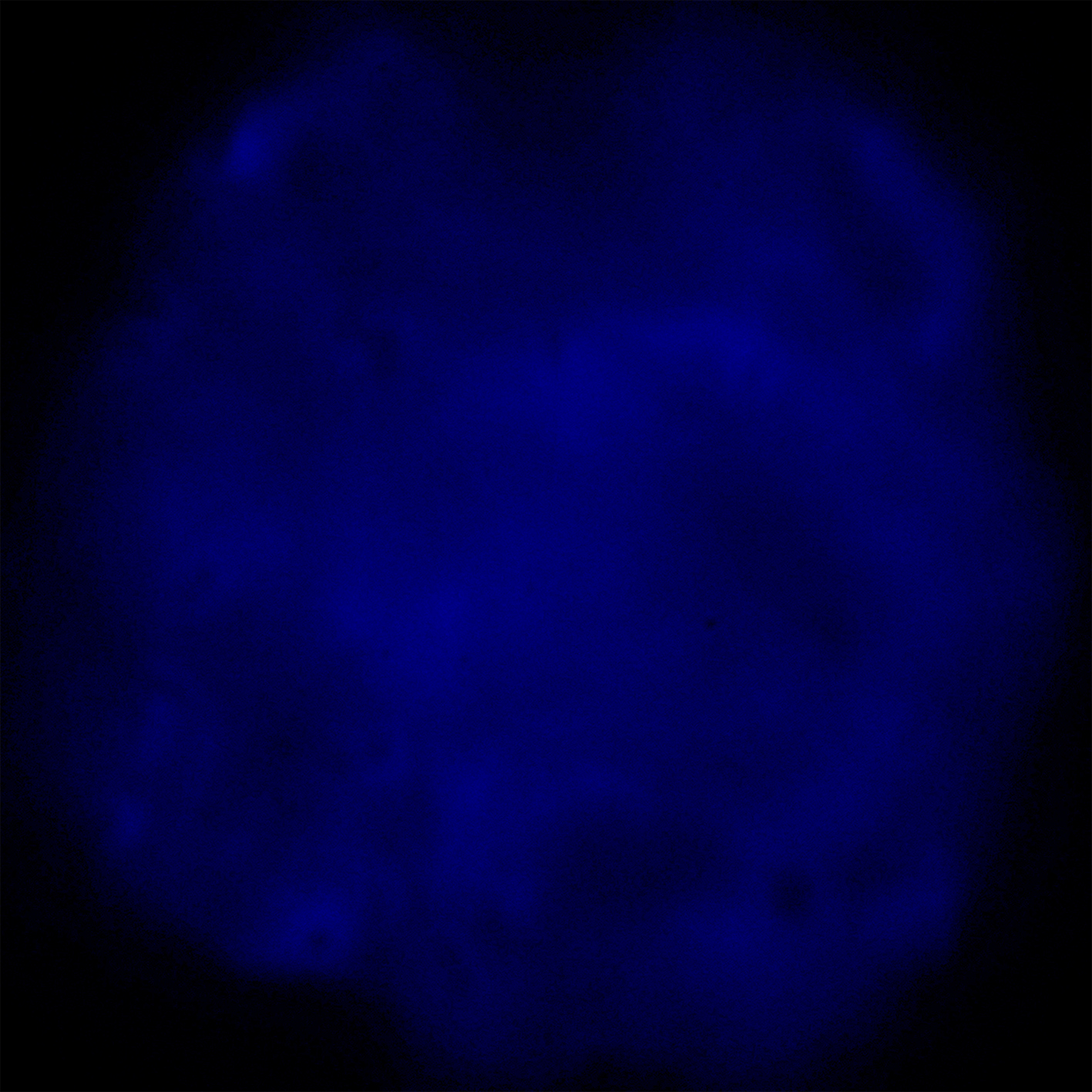

Supplement: Supplementary file 10 — Source data Fig. 3 [file 44318_2024_203_MOESM10_ESM.zip › Figure 3/Figure 3G/cKO-ii-DAPI.jpg]

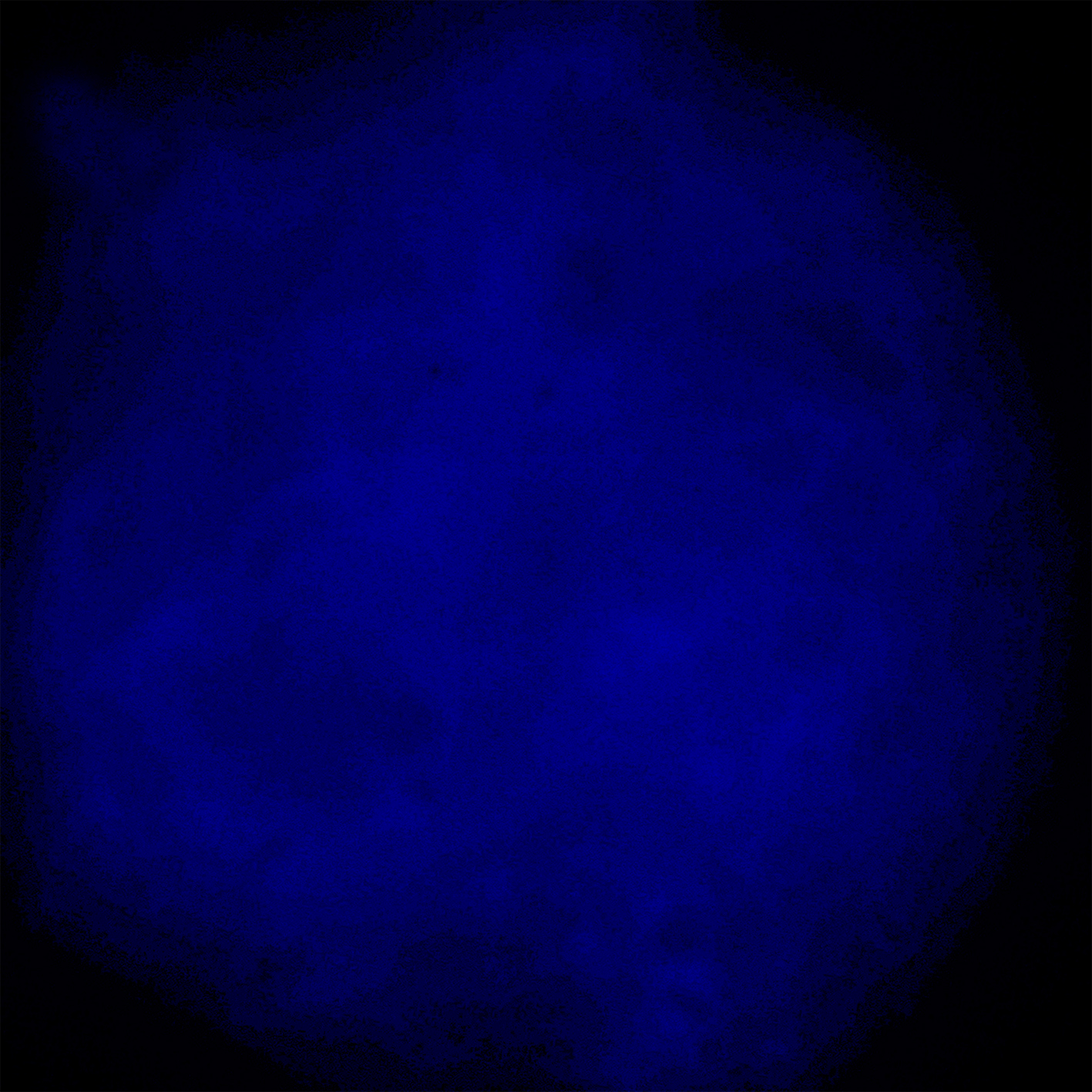

Supplement: Supplementary file 10 — Source data Fig. 3 [file 44318_2024_203_MOESM10_ESM.zip › Figure 3/Figure 3G/cKO-i-DAPI.jpg]

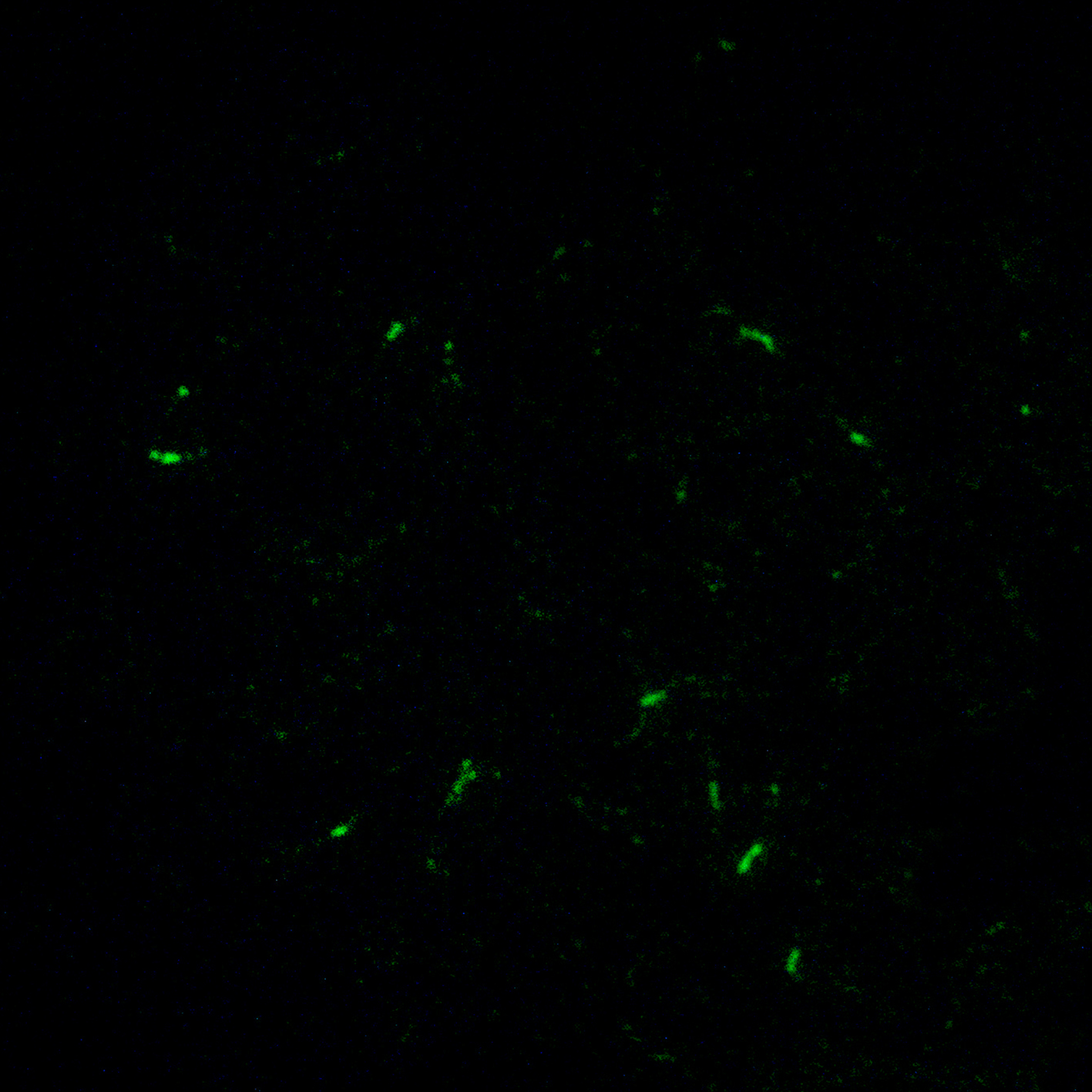

Supplement: Supplementary file 10 — Source data Fig. 3 [file 44318_2024_203_MOESM10_ESM.zip › Figure 3/Figure 3G/cKO-ii-SYCP1.jpg]

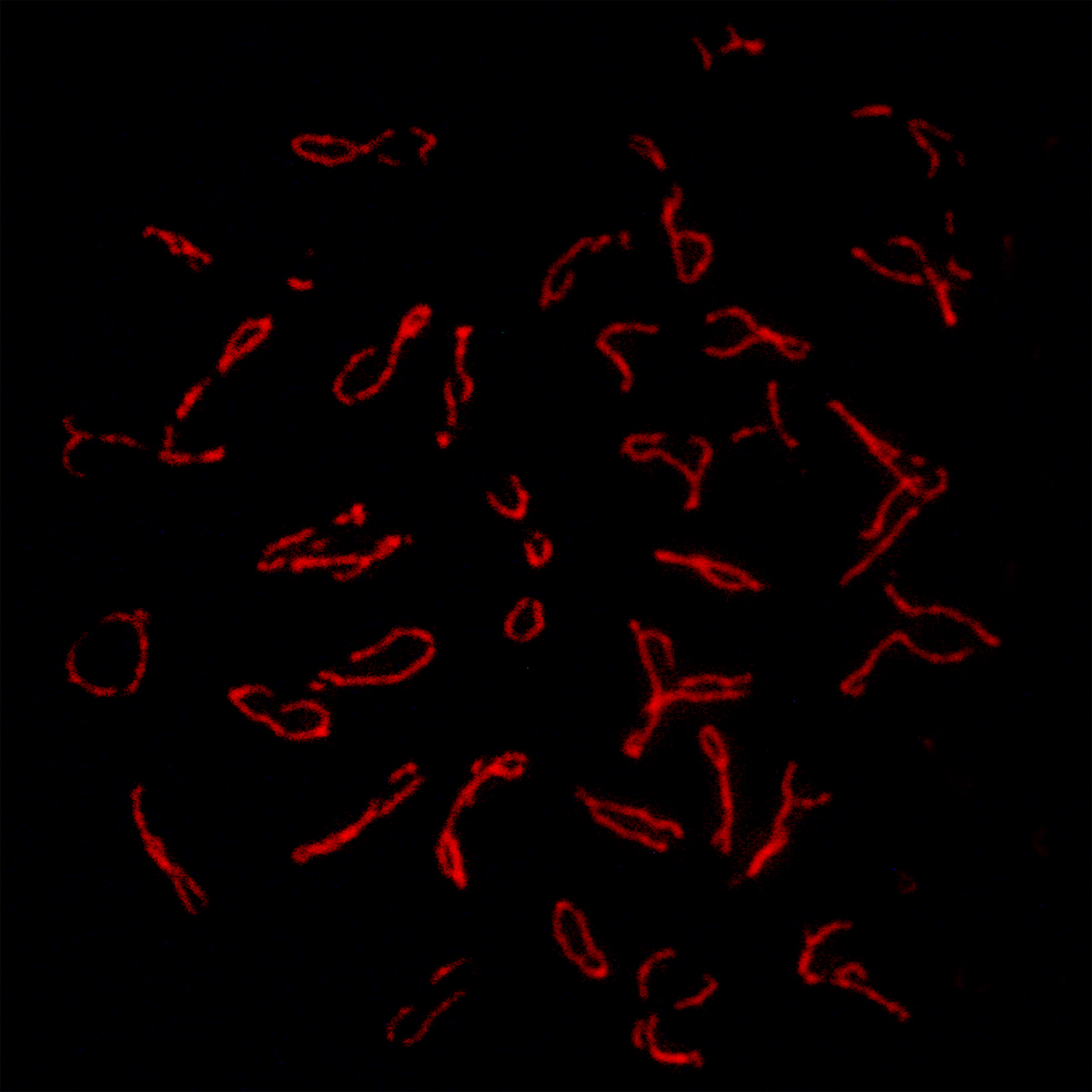

Supplement: Supplementary file 10 — Source data Fig. 3 [file 44318_2024_203_MOESM10_ESM.zip › Figure 3/Figure 3G/cKO-ii-SYCP3.jpg]

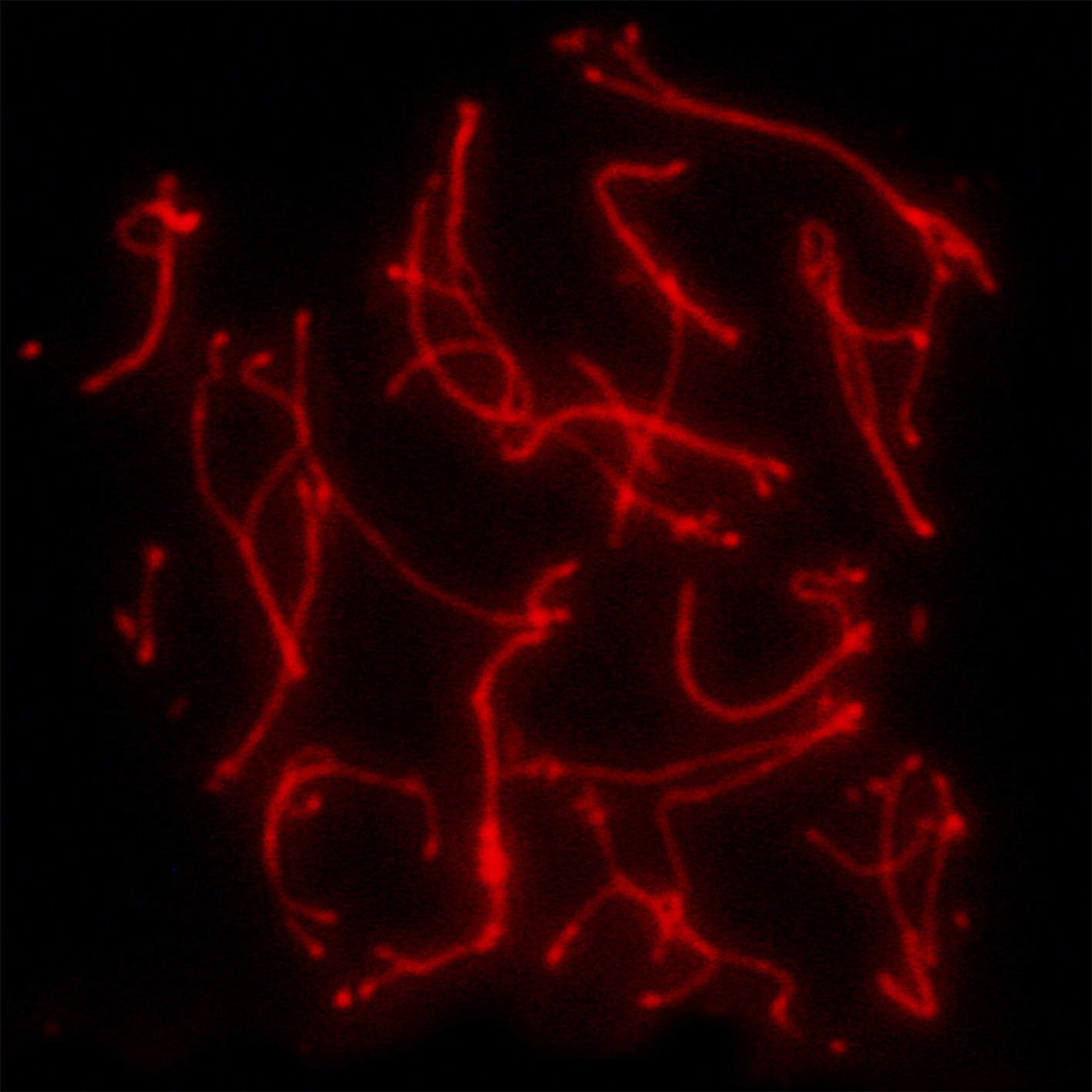

Supplement: Supplementary file 10 — Source data Fig. 3 [file 44318_2024_203_MOESM10_ESM.zip › Figure 3/Figure 3G/Ctrl-SYCP3.jpg]

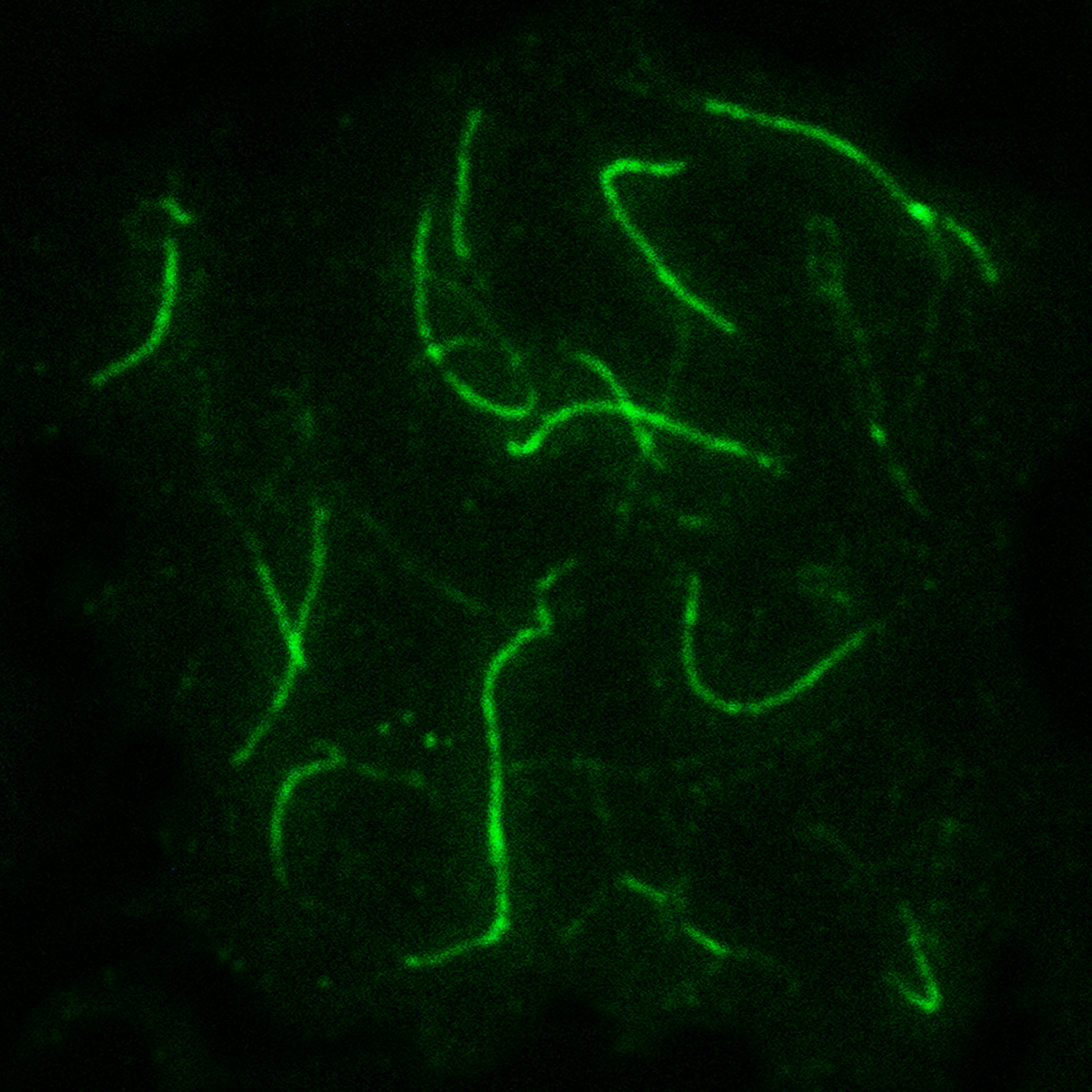

Supplement: Supplementary file 10 — Source data Fig. 3 [file 44318_2024_203_MOESM10_ESM.zip › Figure 3/Figure 3G/Ctrl-SYCP1.jpg]

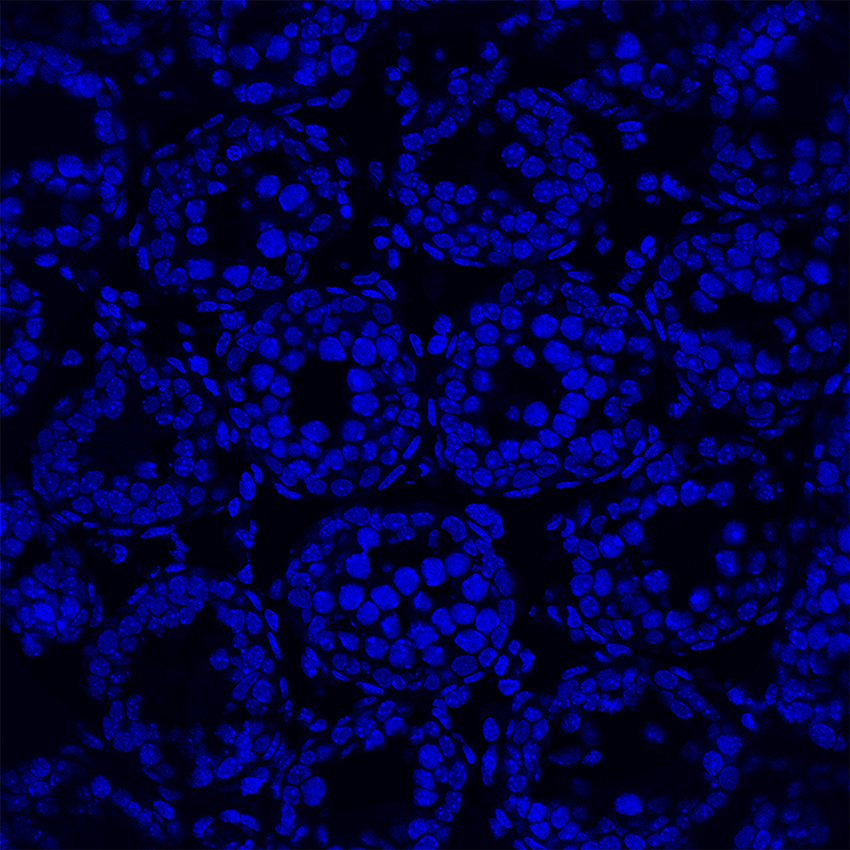

Supplement: Supplementary file 11 — Source data Fig. 5 [file 44318_2024_203_MOESM11_ESM.zip › Figure 5/Figure 5G/Ctrl-DAPI.jpg]

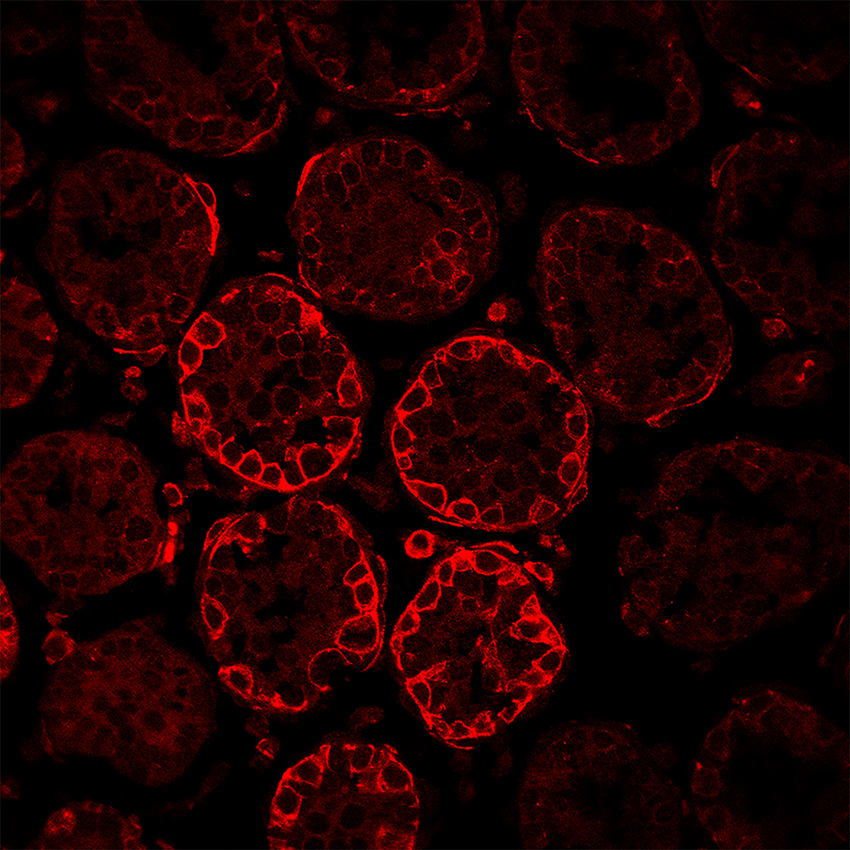

Supplement: Supplementary file 11 — Source data Fig. 5 [file 44318_2024_203_MOESM11_ESM.zip › Figure 5/Figure 5G/cKO-cKIT.jpg]

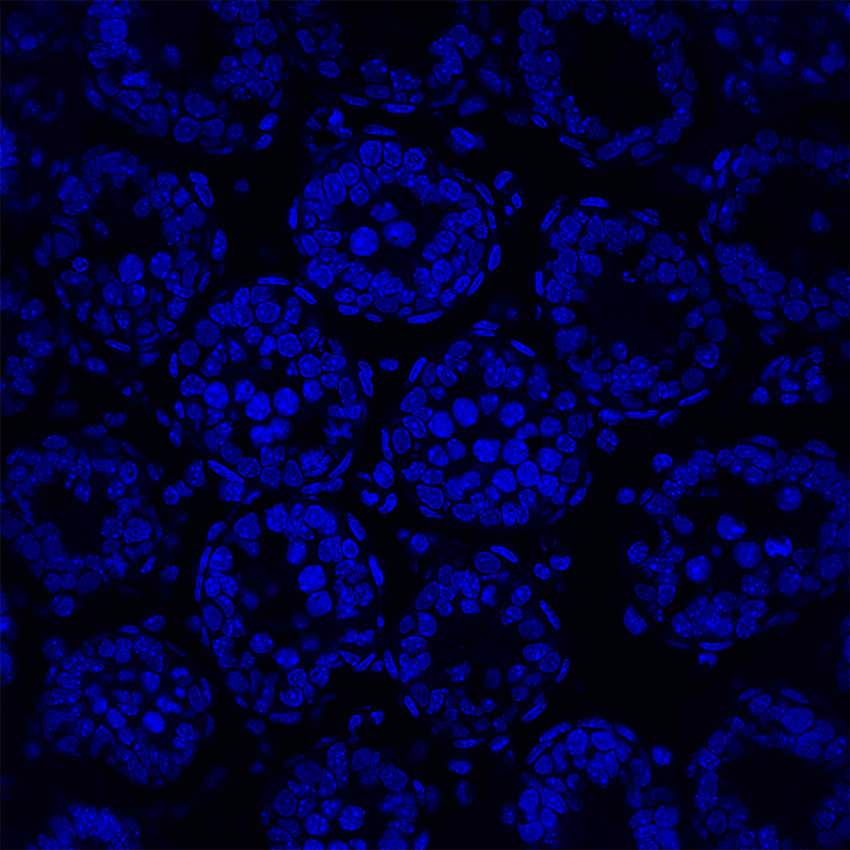

Supplement: Supplementary file 11 — Source data Fig. 5 [file 44318_2024_203_MOESM11_ESM.zip › Figure 5/Figure 5G/cKO-DAPI.jpg]

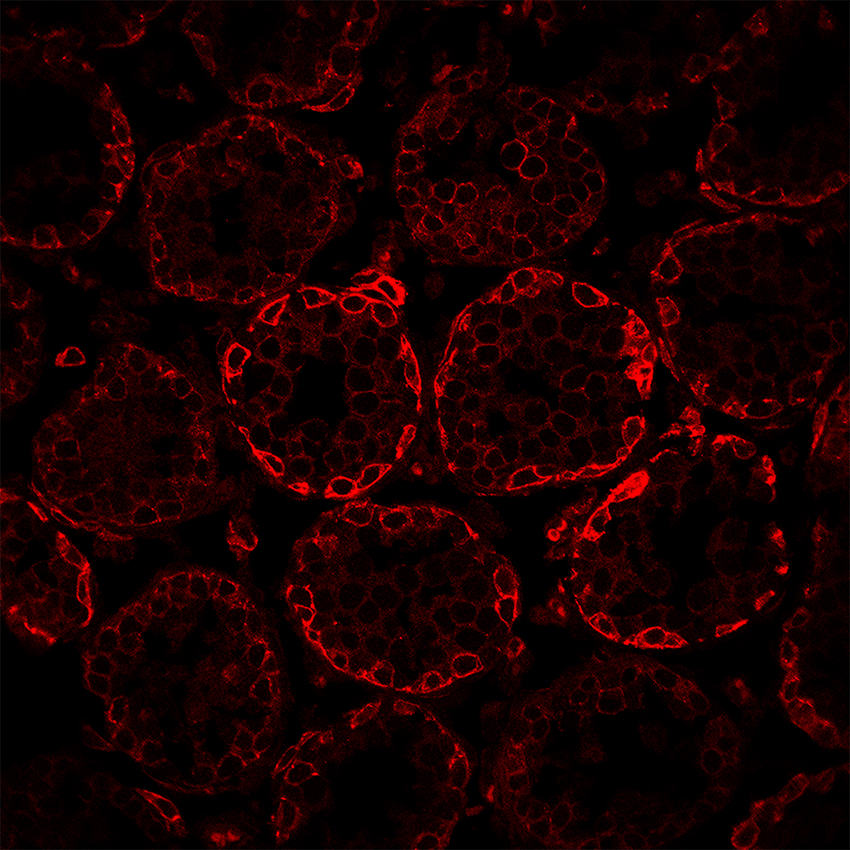

Supplement: Supplementary file 11 — Source data Fig. 5 [file 44318_2024_203_MOESM11_ESM.zip › Figure 5/Figure 5G/Ctrl-SYCP3.jpg]

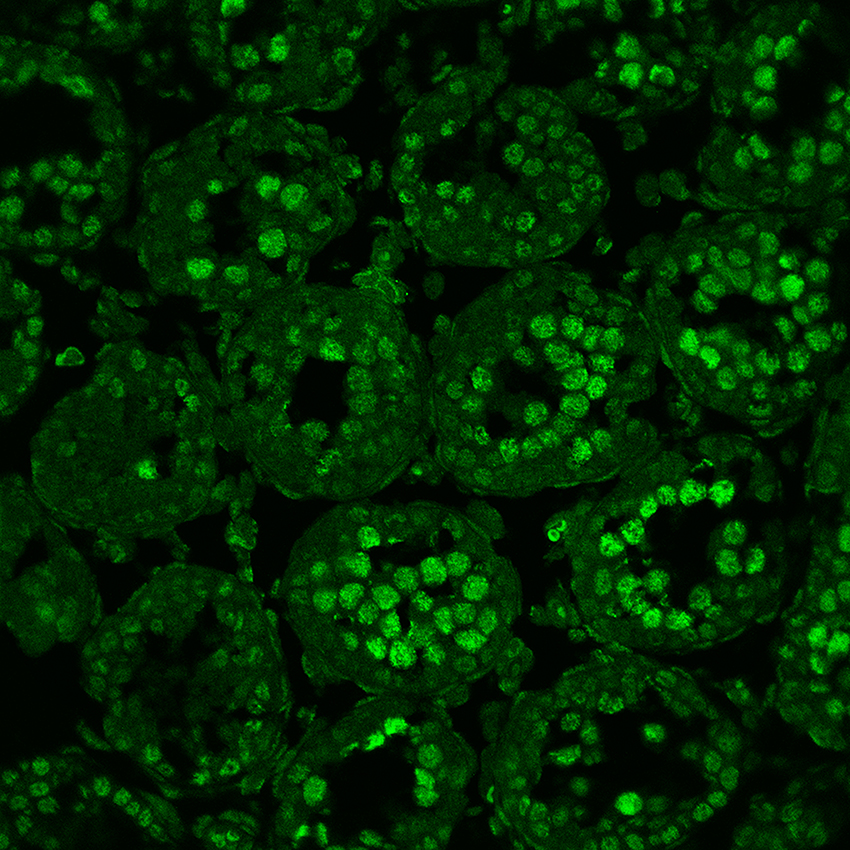

Supplement: Supplementary file 11 — Source data Fig. 5 [file 44318_2024_203_MOESM11_ESM.zip › Figure 5/Figure 5G/Ctrl-H3K36me3.jpg]

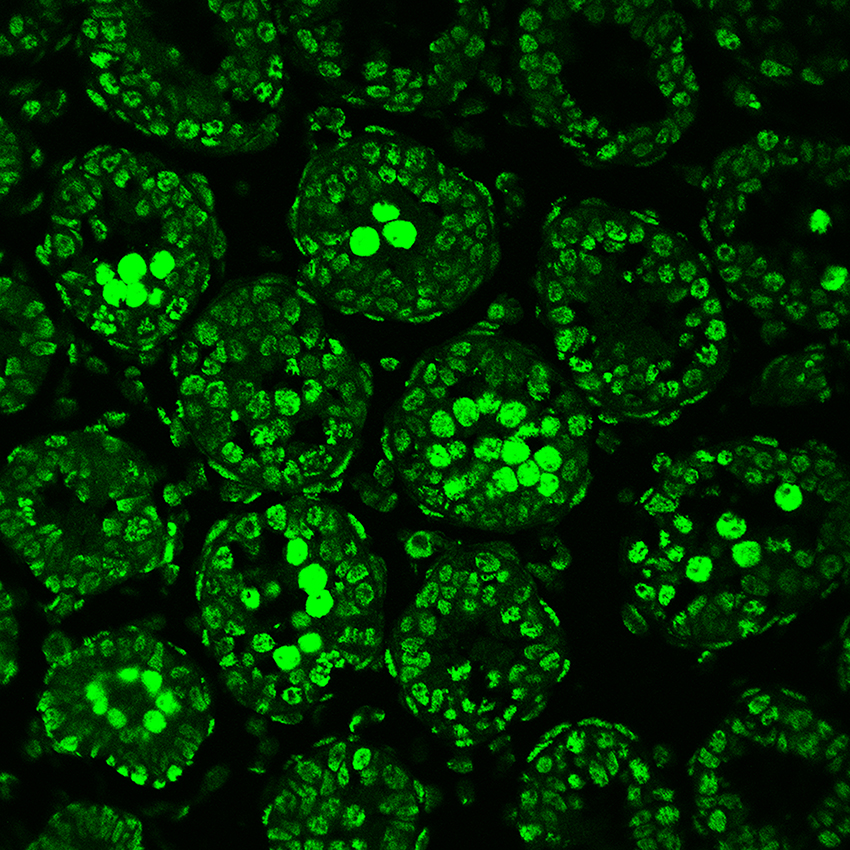

Supplement: Supplementary file 11 — Source data Fig. 5 [file 44318_2024_203_MOESM11_ESM.zip › Figure 5/Figure 5G/cKO-H3K36me3.jpg]

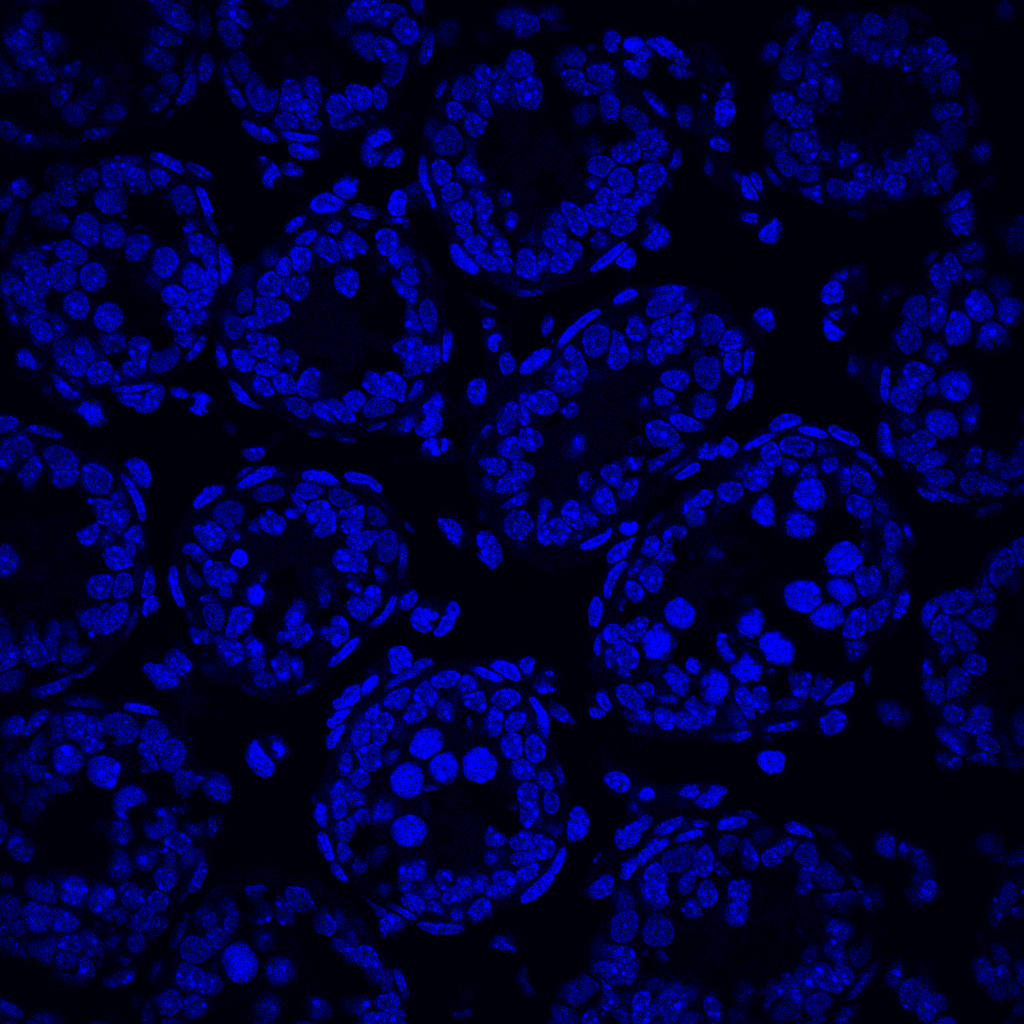

Supplement: Supplementary file 11 — Source data Fig. 5 [file 44318_2024_203_MOESM11_ESM.zip › Figure 5/Figure 5F/Ctrl-DAPI.jpg]

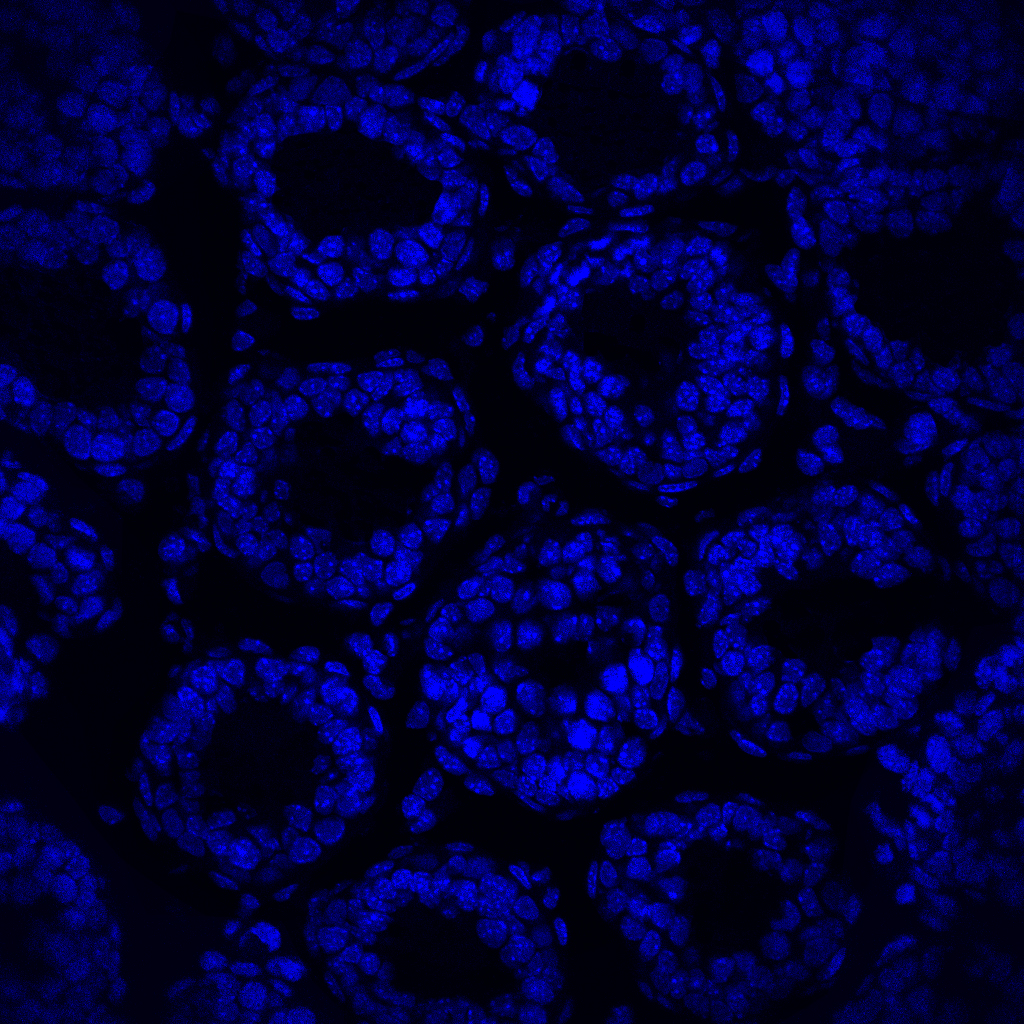

Supplement: Supplementary file 11 — Source data Fig. 5 [file 44318_2024_203_MOESM11_ESM.zip › Figure 5/Figure 5F/cKO-DAPI.jpg]

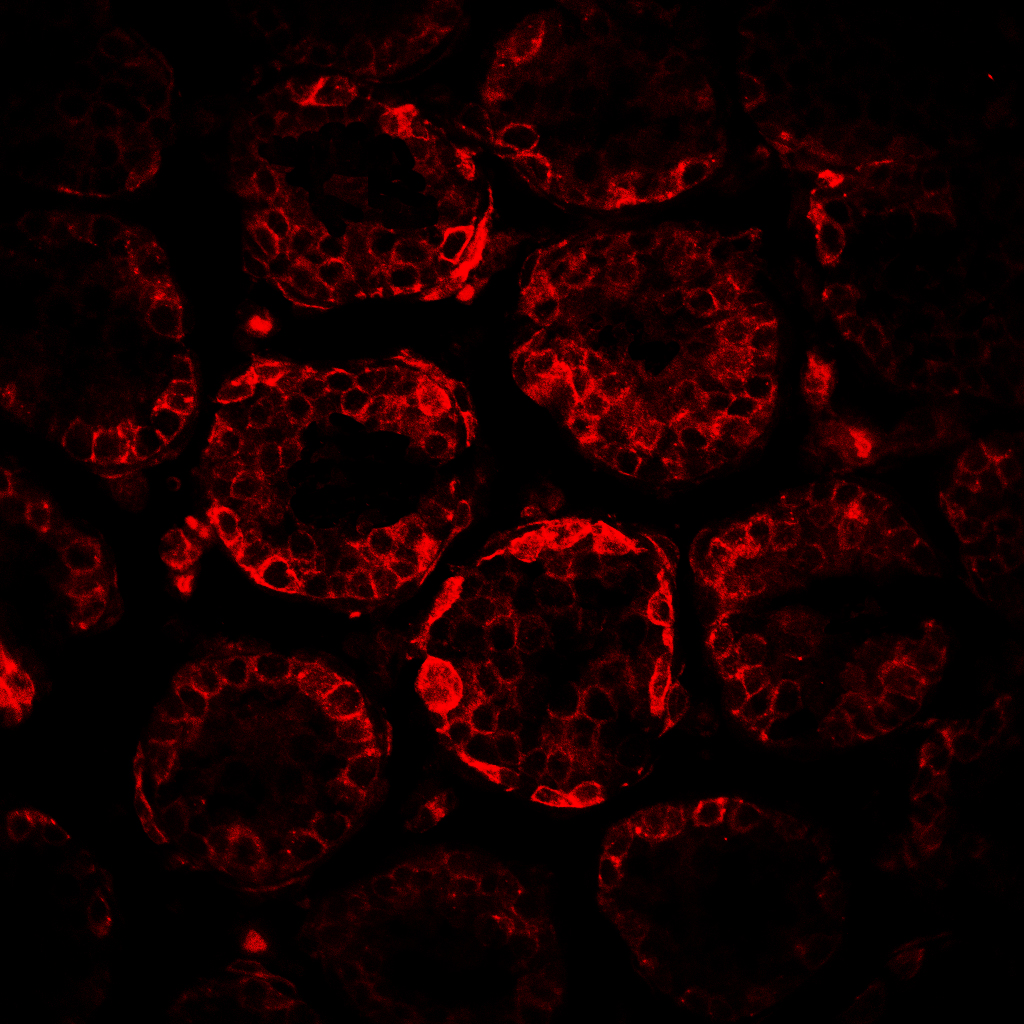

Supplement: Supplementary file 11 — Source data Fig. 5 [file 44318_2024_203_MOESM11_ESM.zip › Figure 5/Figure 5F/cKO-SYCP3.jpg]

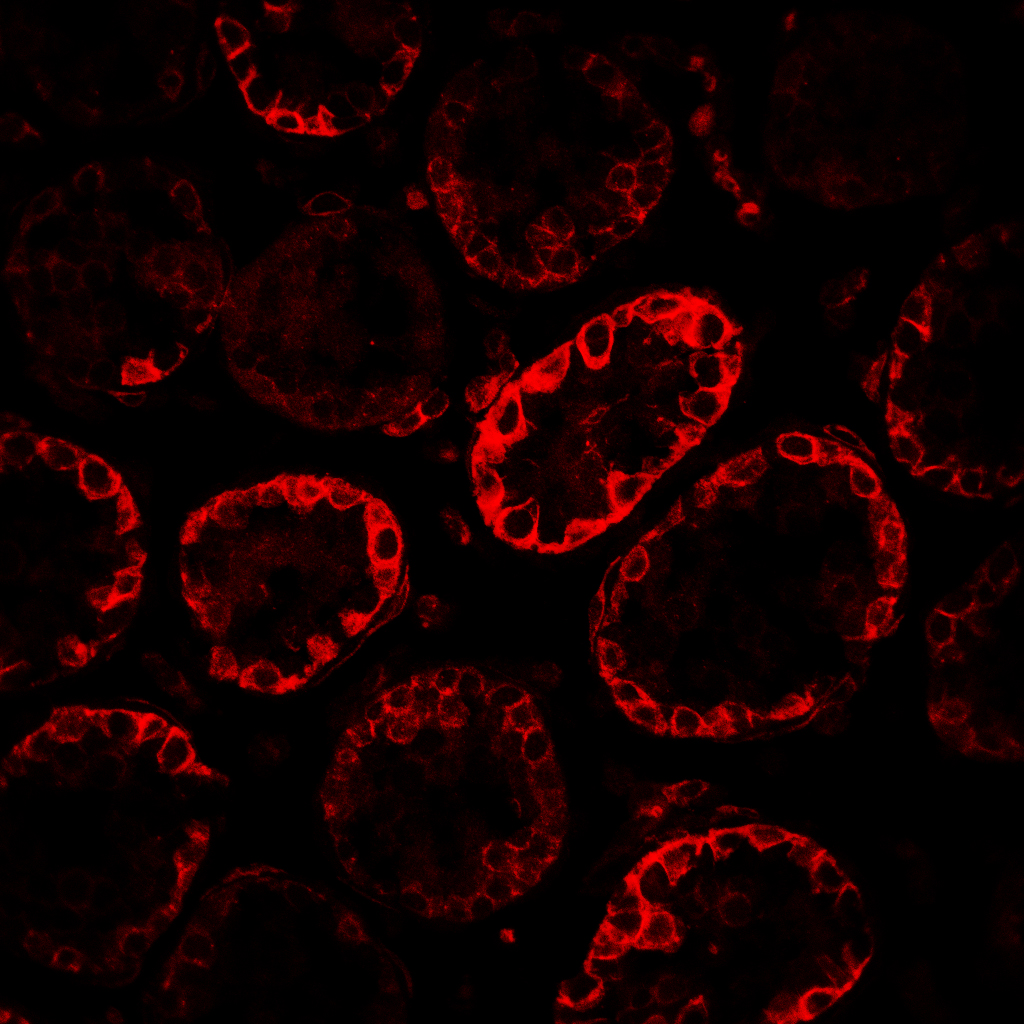

Supplement: Supplementary file 11 — Source data Fig. 5 [file 44318_2024_203_MOESM11_ESM.zip › Figure 5/Figure 5F/Ctrl-SYCP3.jpg]

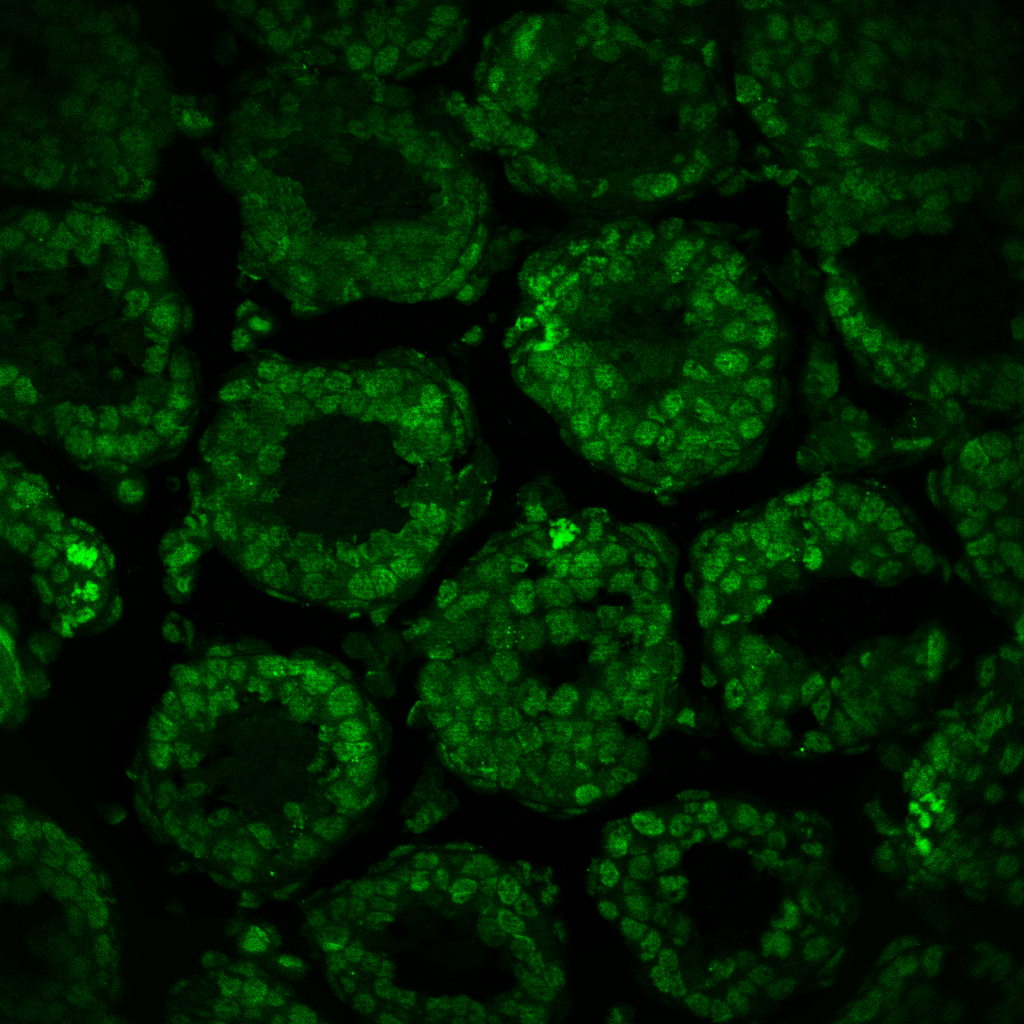

Supplement: Supplementary file 11 — Source data Fig. 5 [file 44318_2024_203_MOESM11_ESM.zip › Figure 5/Figure 5F/cKO-H3K36me2.jpg]

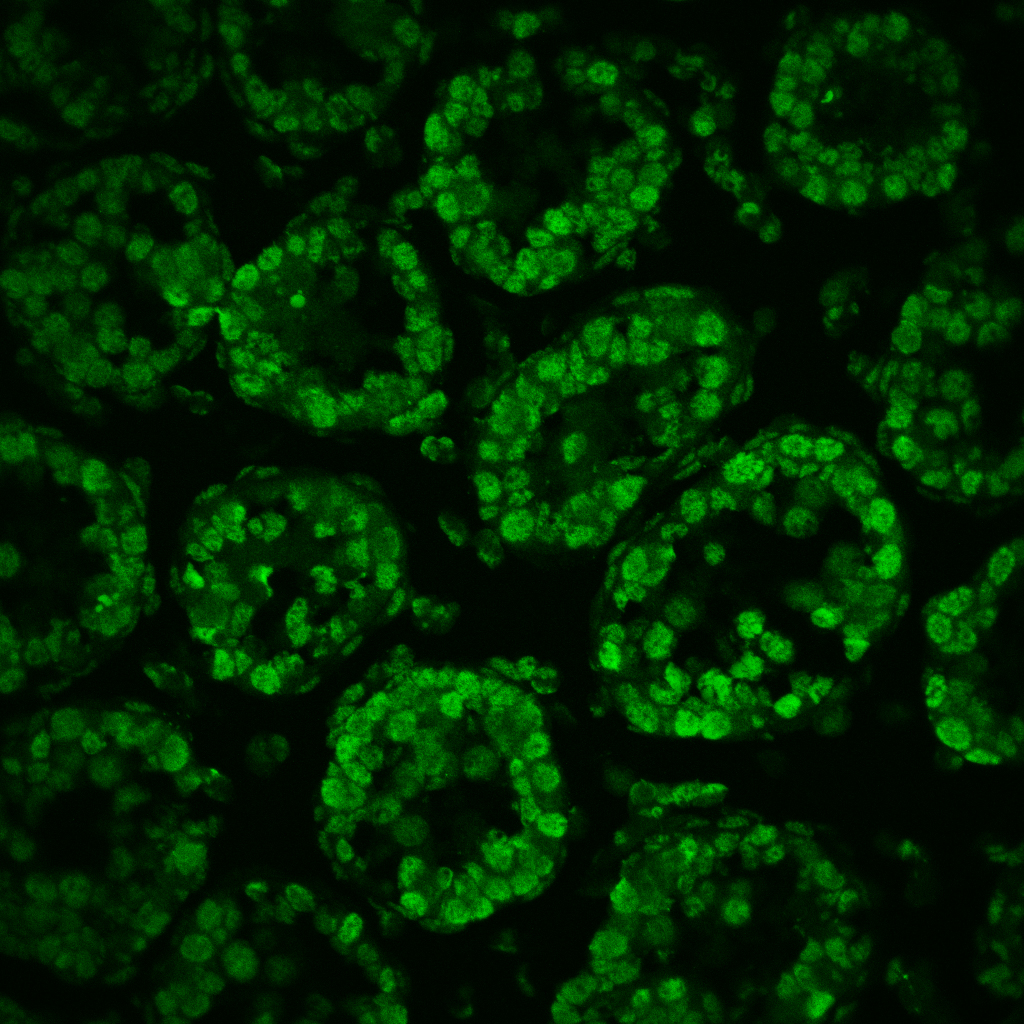

Supplement: Supplementary file 11 — Source data Fig. 5 [file 44318_2024_203_MOESM11_ESM.zip › Figure 5/Figure 5F/Ctrl-H3K36me2.jpg]

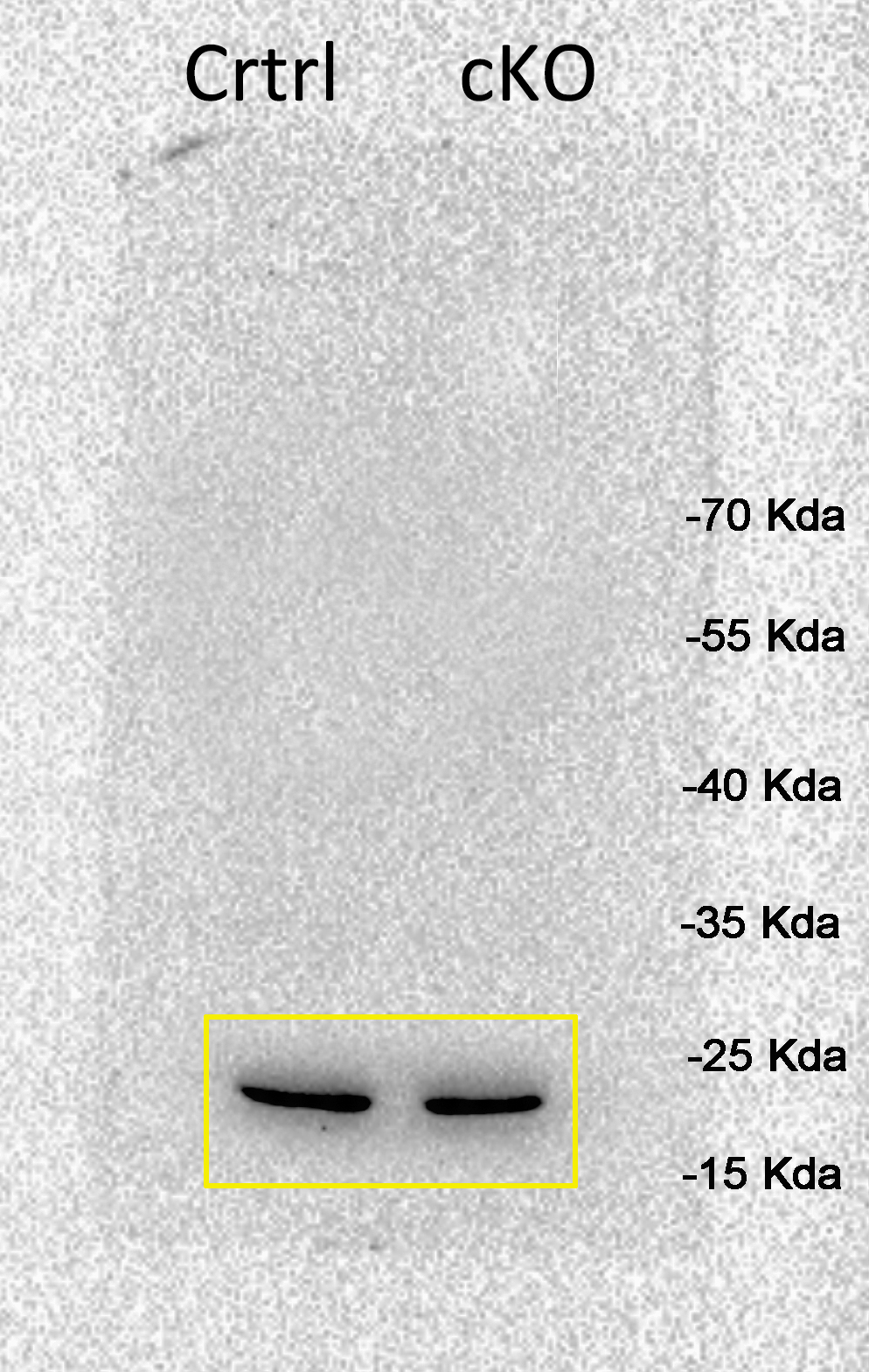

Supplement: Supplementary file 11 — Source data Fig. 5 [file 44318_2024_203_MOESM11_ESM.zip › Figure 5/Figure 5H/WB-H3.jpg]

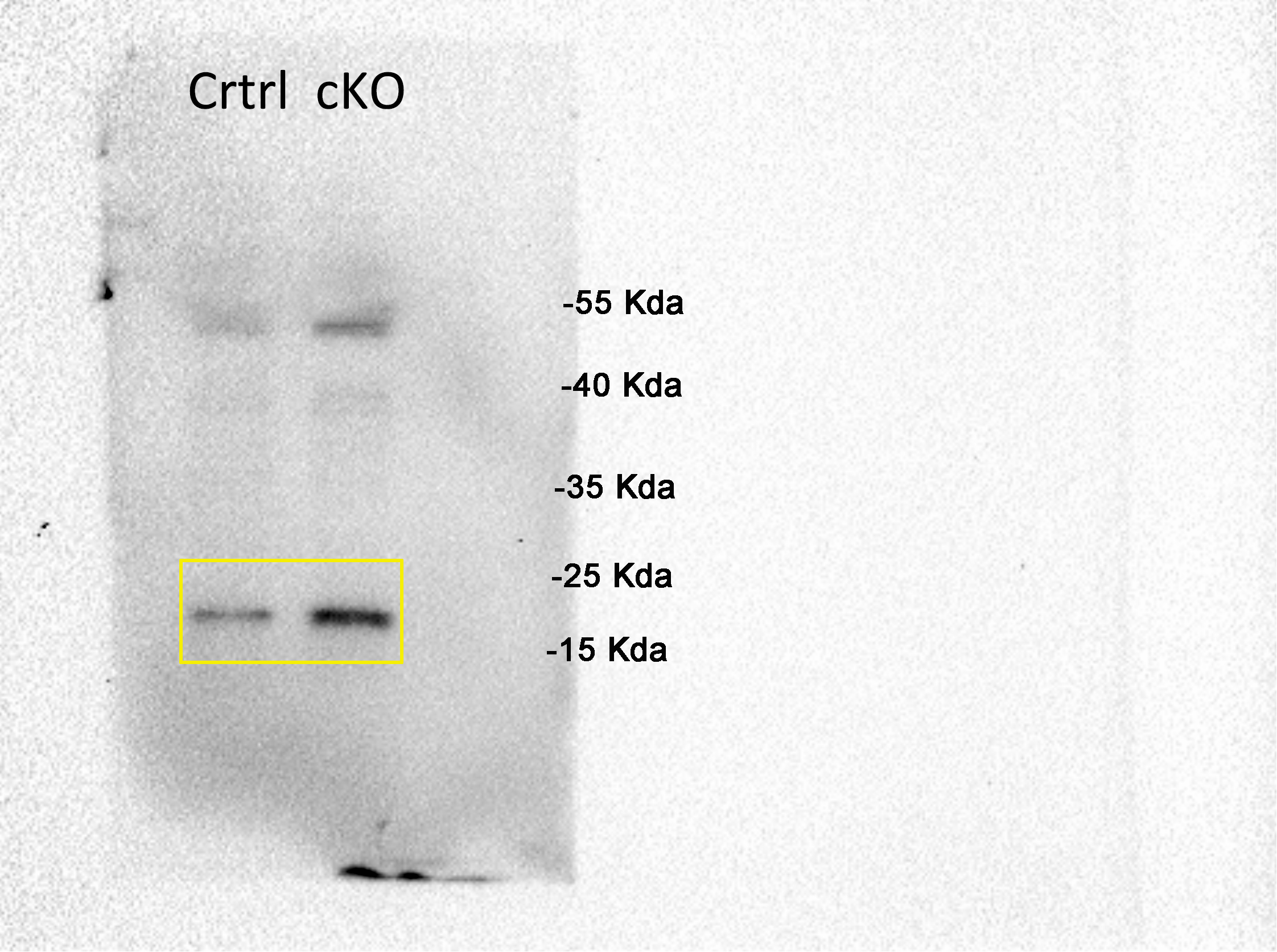

Supplement: Supplementary file 11 — Source data Fig. 5 [file 44318_2024_203_MOESM11_ESM.zip › Figure 5/Figure 5H/WB-H3KK36me3.jpg]

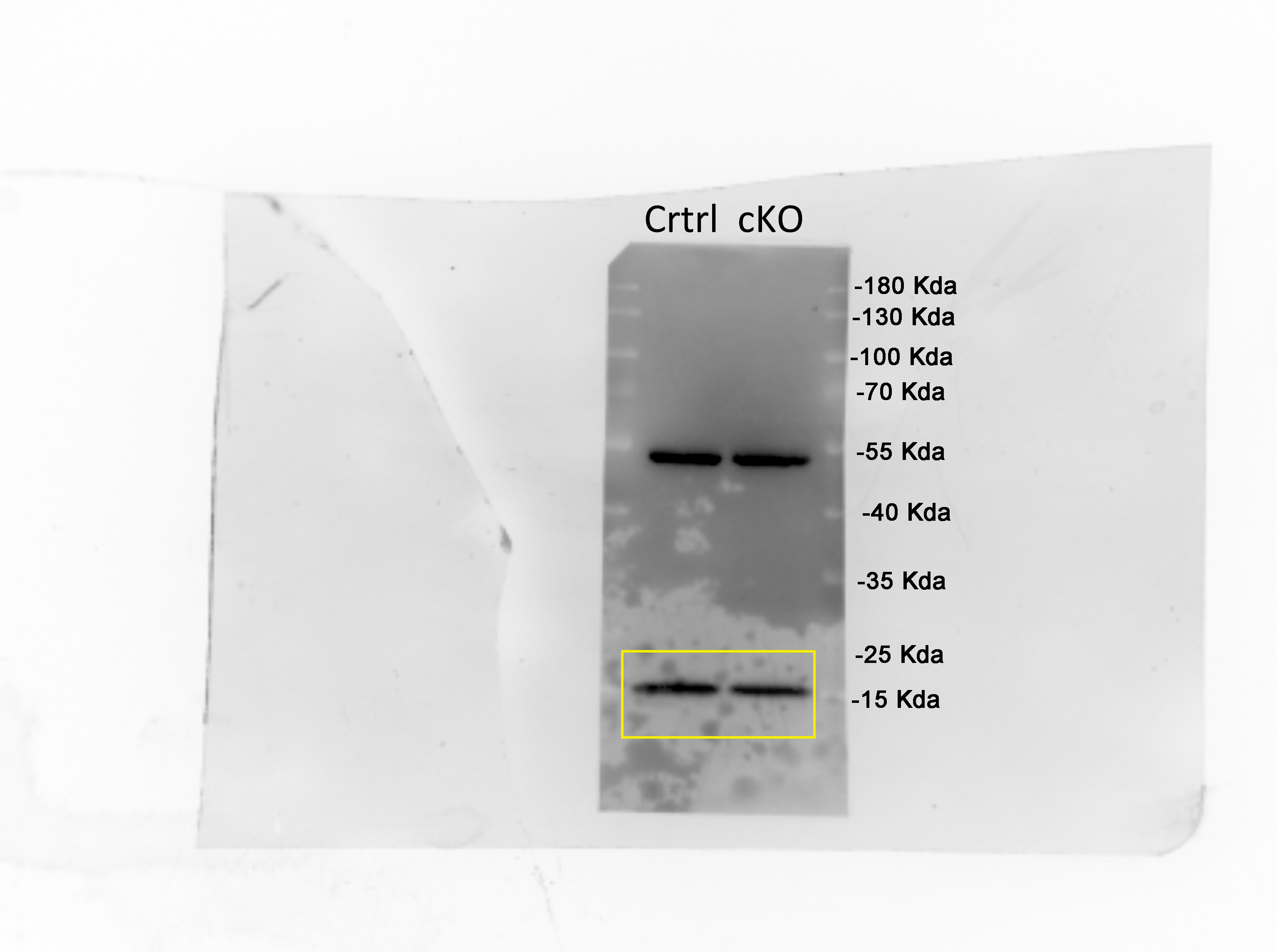

Supplement: Supplementary file 11 — Source data Fig. 5 [file 44318_2024_203_MOESM11_ESM.zip › Figure 5/Figure 5H/WB-H3K36me1.jpg]

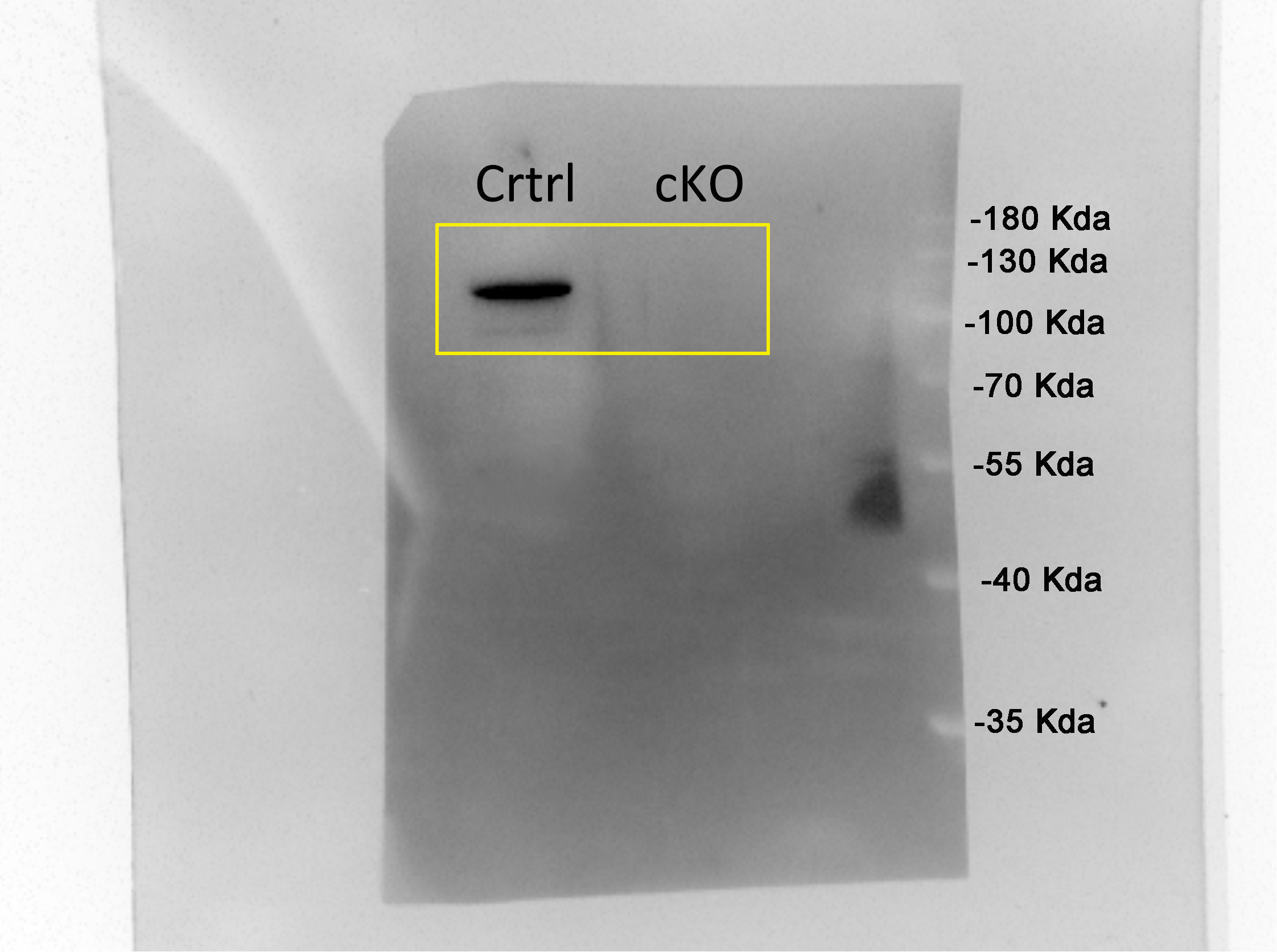

Supplement: Supplementary file 11 — Source data Fig. 5 [file 44318_2024_203_MOESM11_ESM.zip › Figure 5/Figure 5H/WB-KDM2A.jpg]

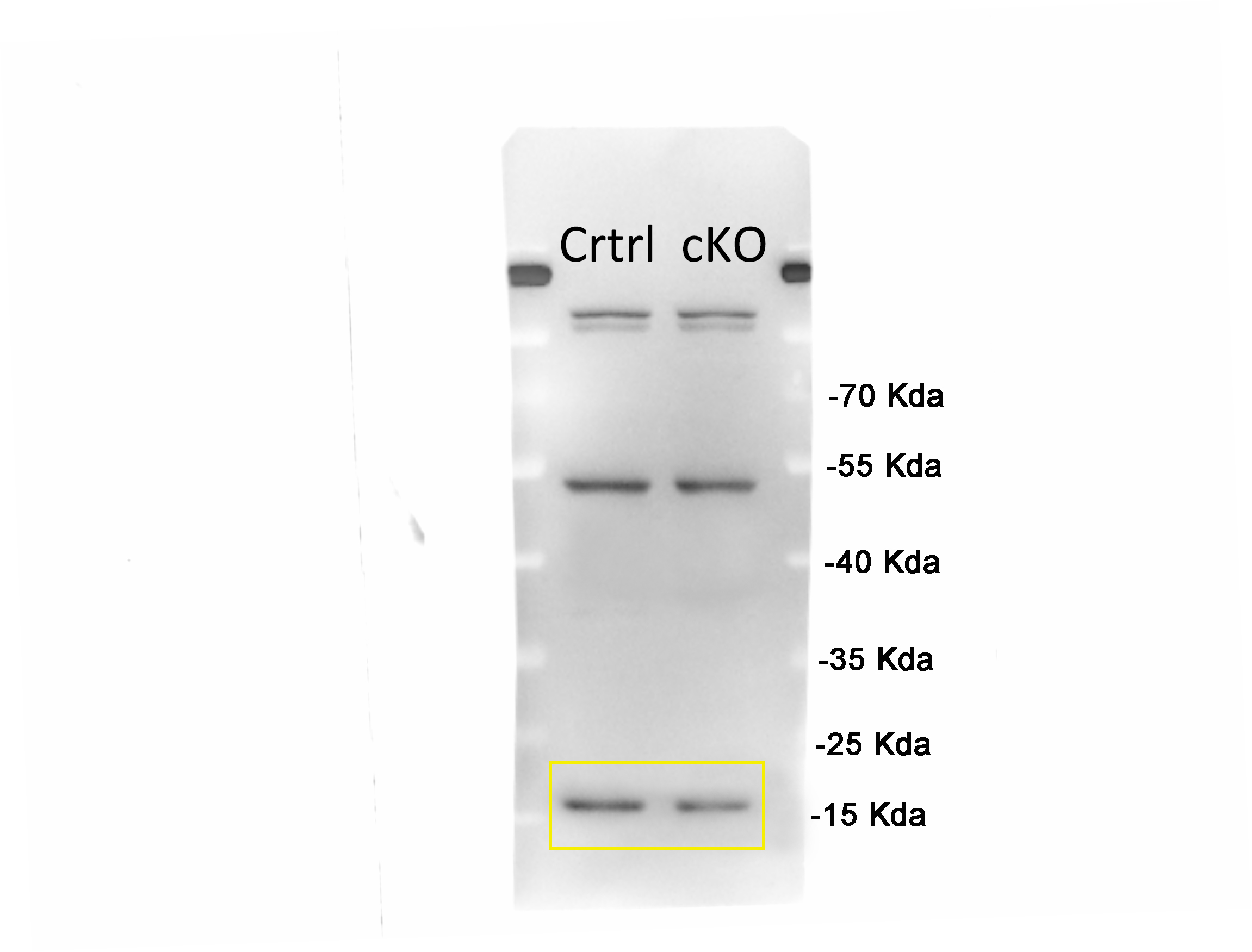

Supplement: Supplementary file 11 — Source data Fig. 5 [file 44318_2024_203_MOESM11_ESM.zip › Figure 5/Figure 5H/WB-H3K36me2.jpg]

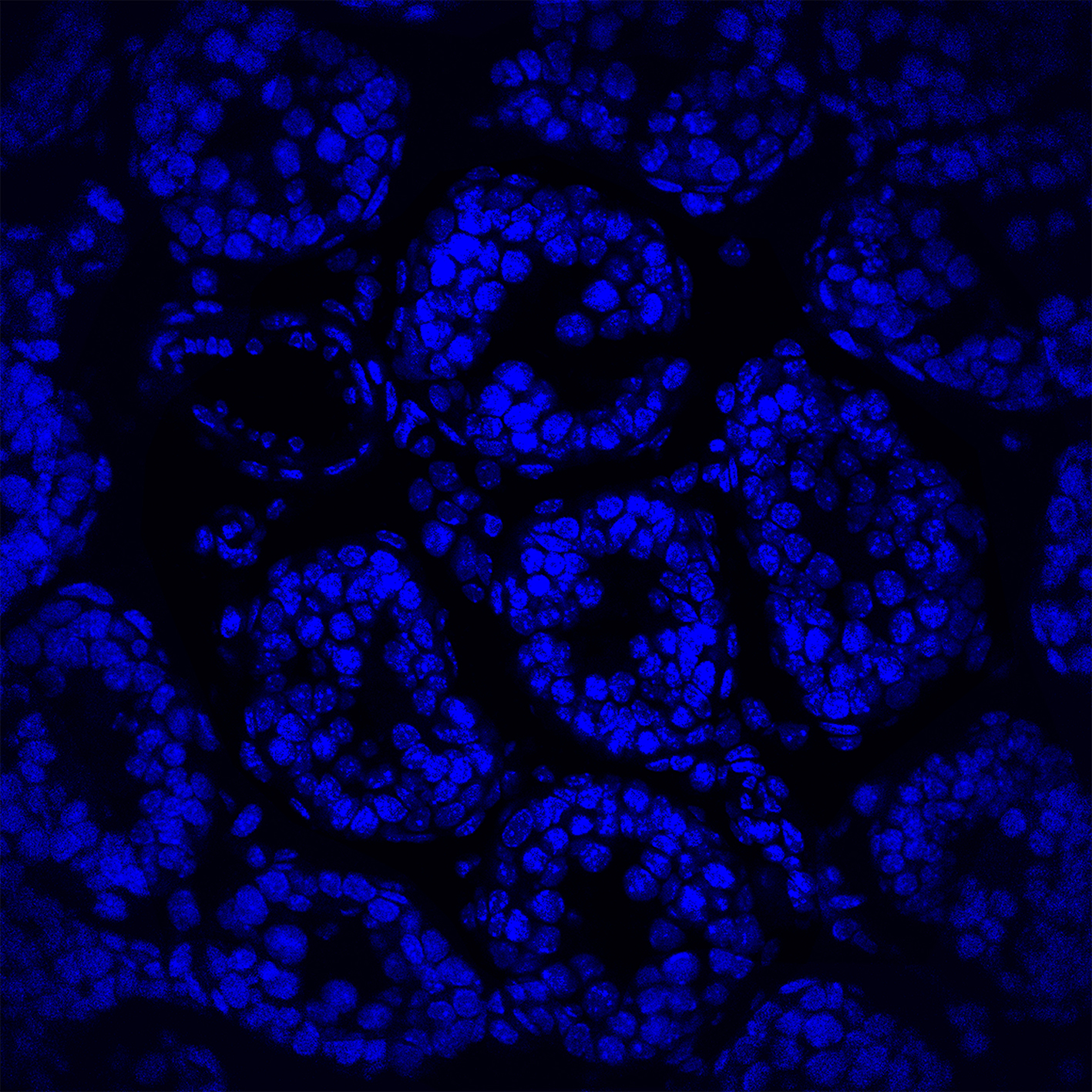

Supplement: Supplementary file 11 — Source data Fig. 5 [file 44318_2024_203_MOESM11_ESM.zip › Figure 5/Figure 5E/Ctrl-DAPI.jpg]

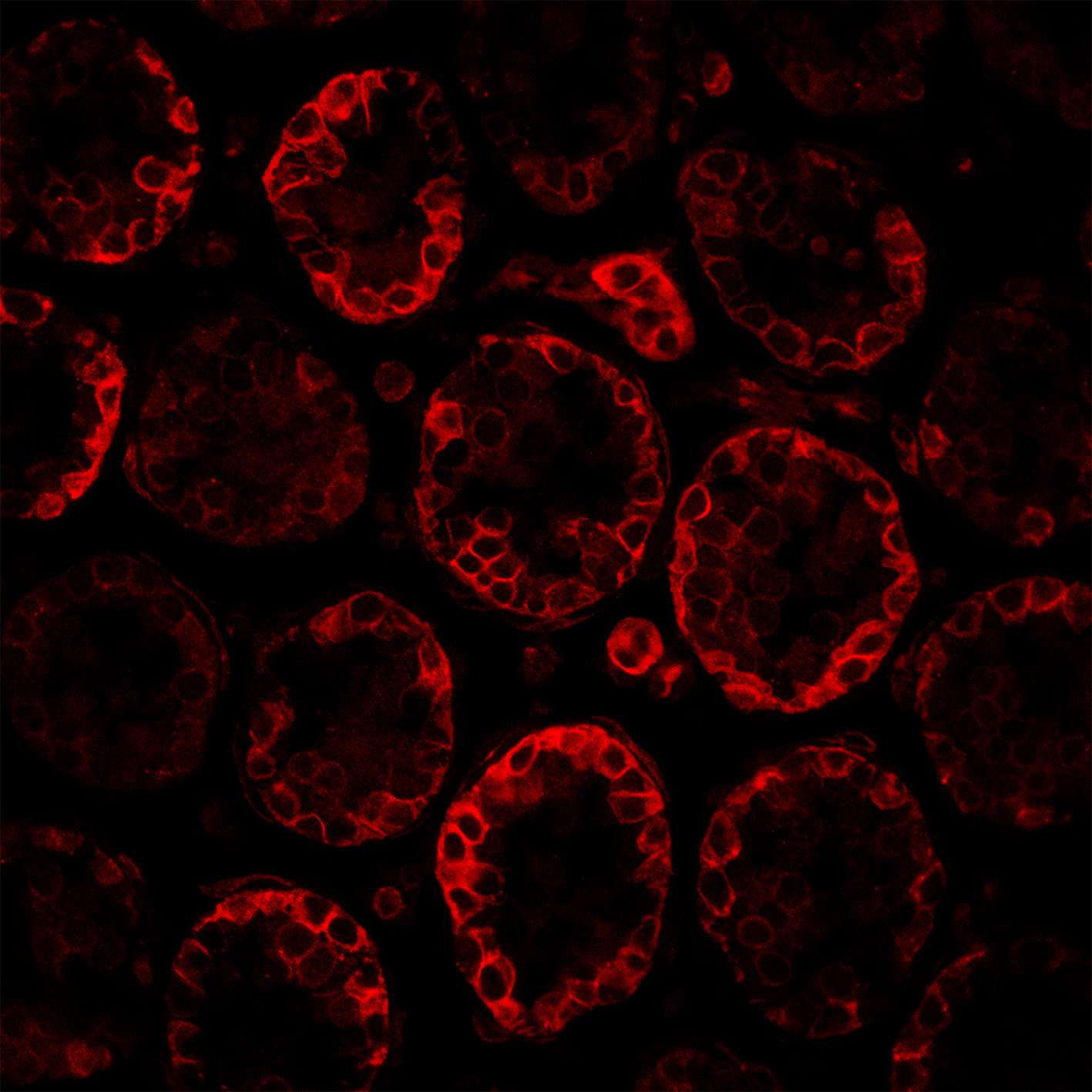

Supplement: Supplementary file 11 — Source data Fig. 5 [file 44318_2024_203_MOESM11_ESM.zip › Figure 5/Figure 5E/cKO-cKIT.jpg]

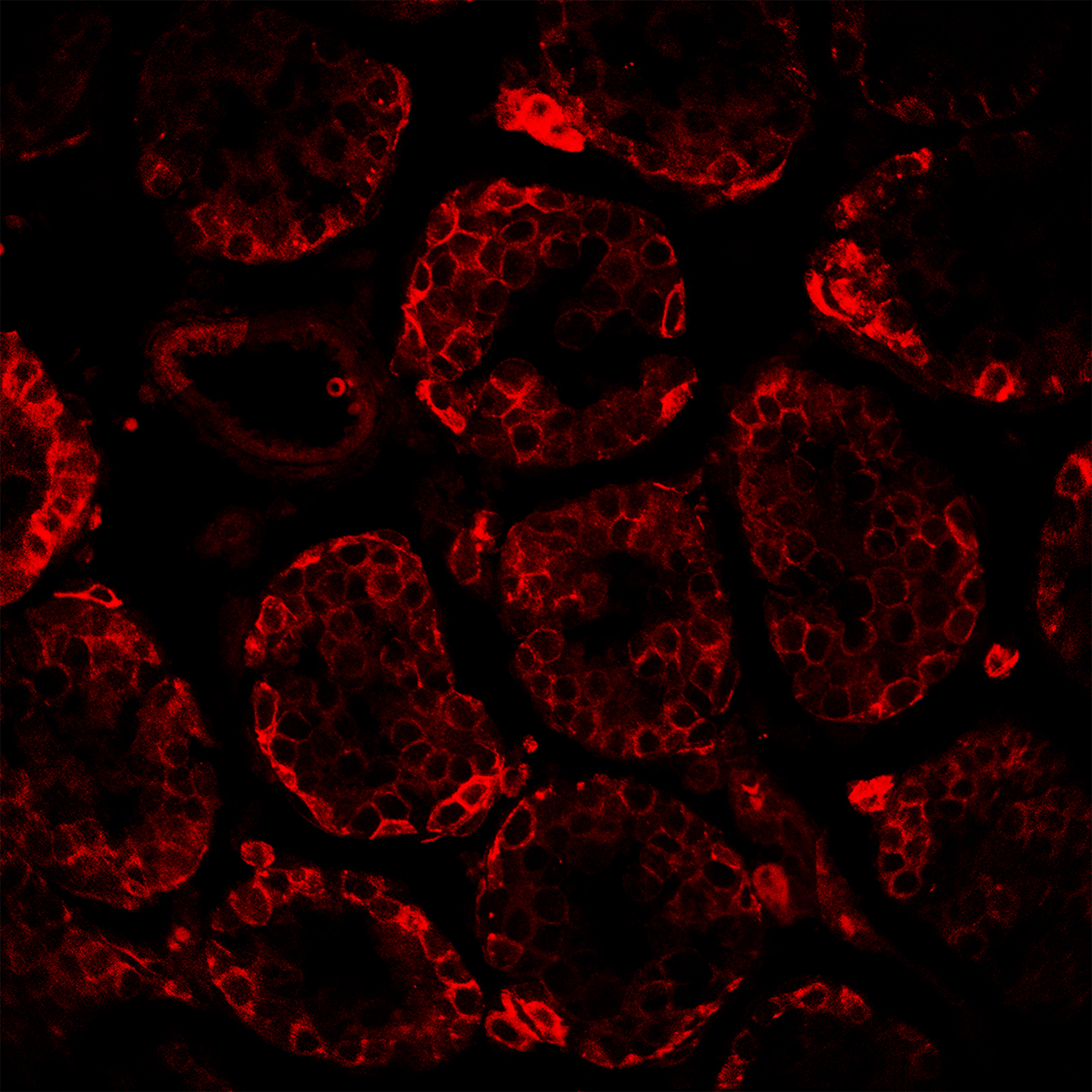

Supplement: Supplementary file 11 — Source data Fig. 5 [file 44318_2024_203_MOESM11_ESM.zip › Figure 5/Figure 5E/Ctrl-cKIT.jpg]

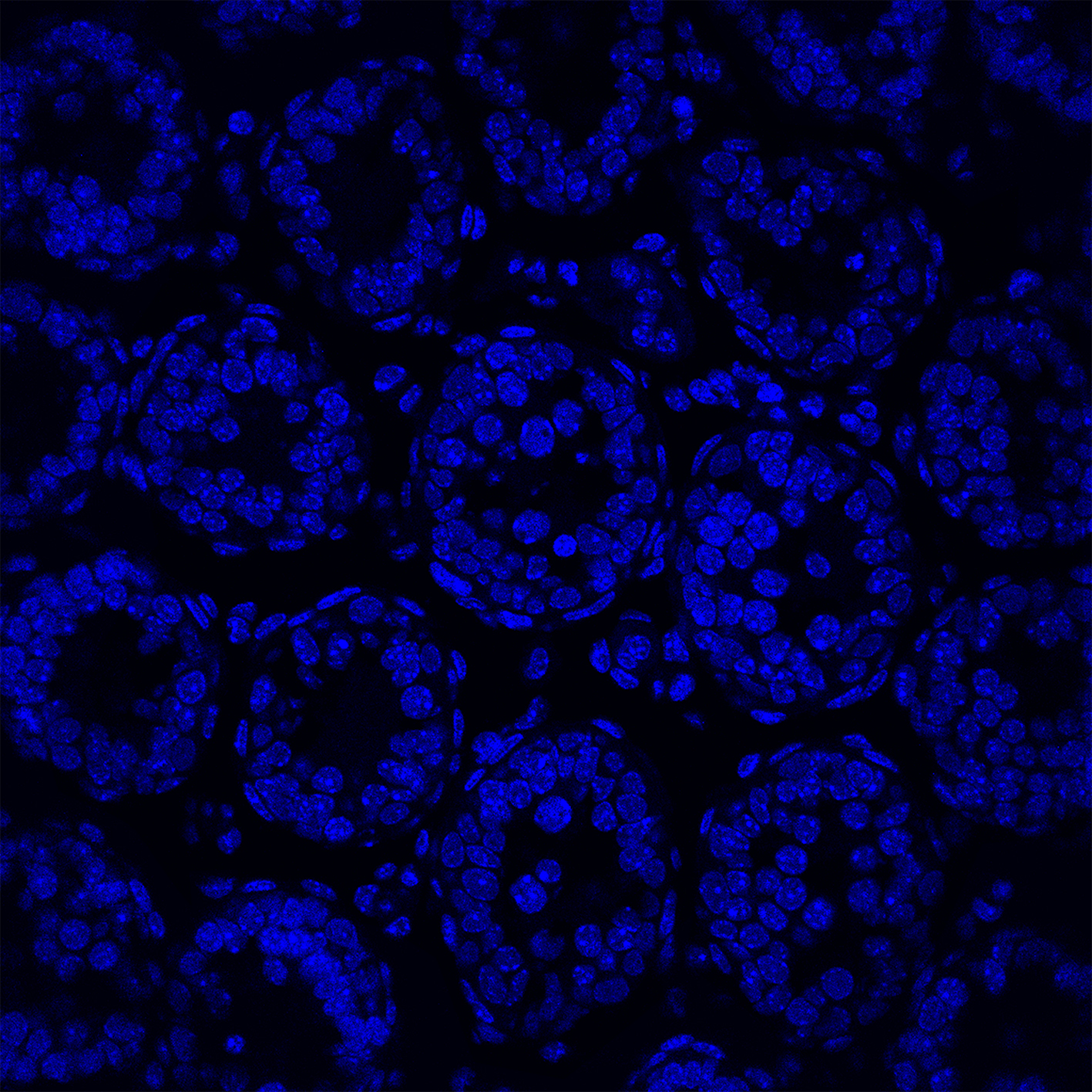

Supplement: Supplementary file 11 — Source data Fig. 5 [file 44318_2024_203_MOESM11_ESM.zip › Figure 5/Figure 5E/cKO-DAPI.jpg]

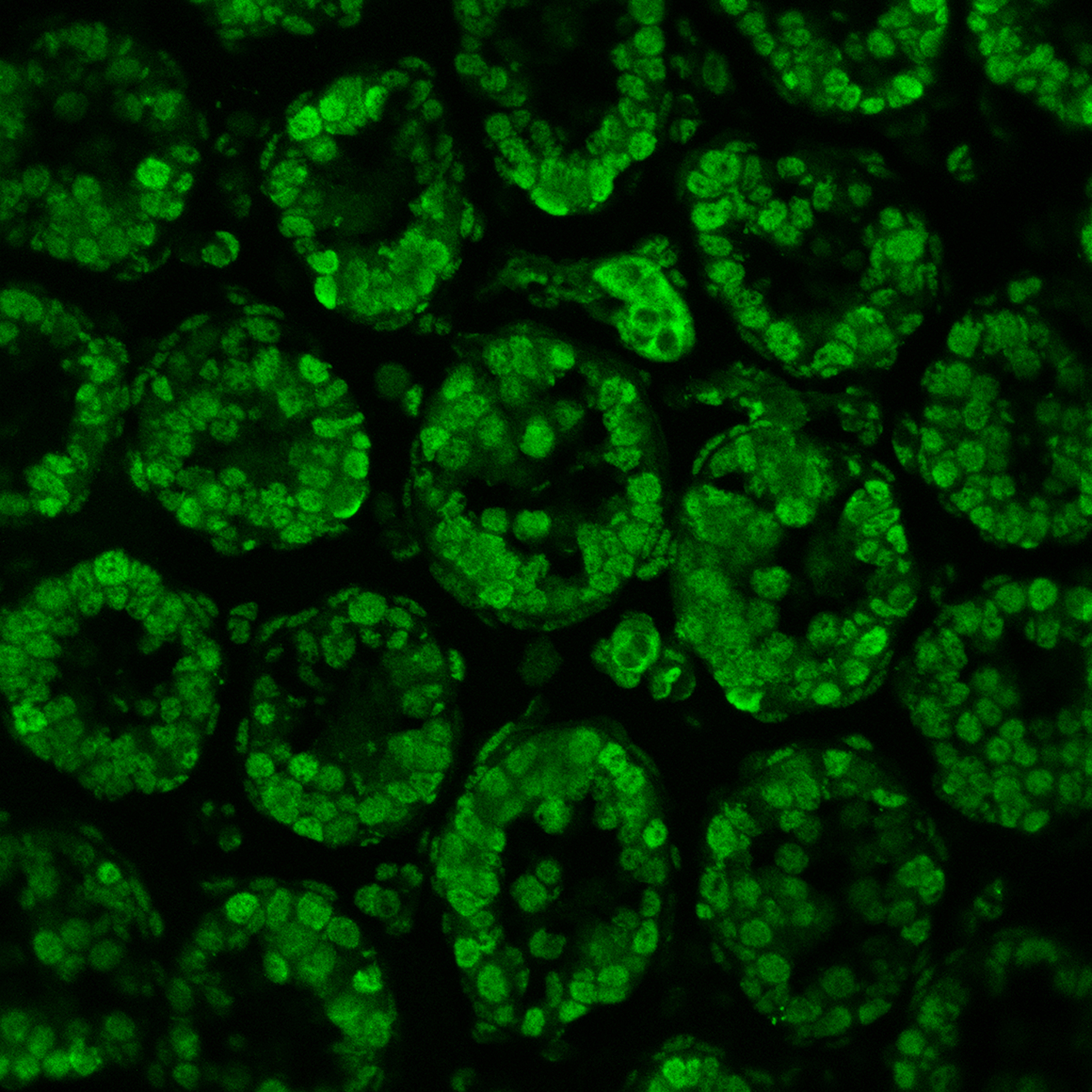

Supplement: Supplementary file 11 — Source data Fig. 5 [file 44318_2024_203_MOESM11_ESM.zip › Figure 5/Figure 5E/cKO-H3K36me1.jpg]

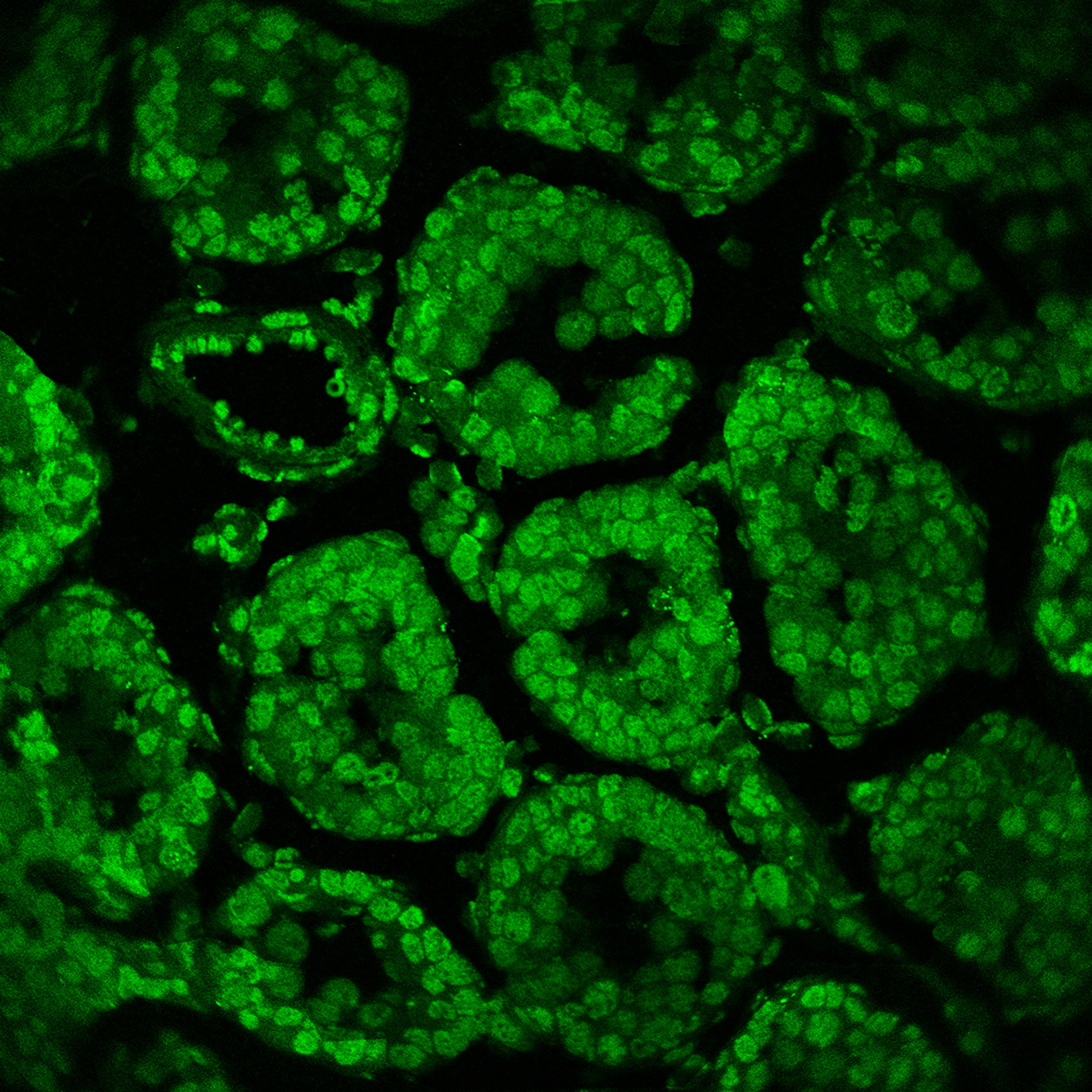

Supplement: Supplementary file 11 — Source data Fig. 5 [file 44318_2024_203_MOESM11_ESM.zip › Figure 5/Figure 5E/Ctrl-H3K36me1.jpg]

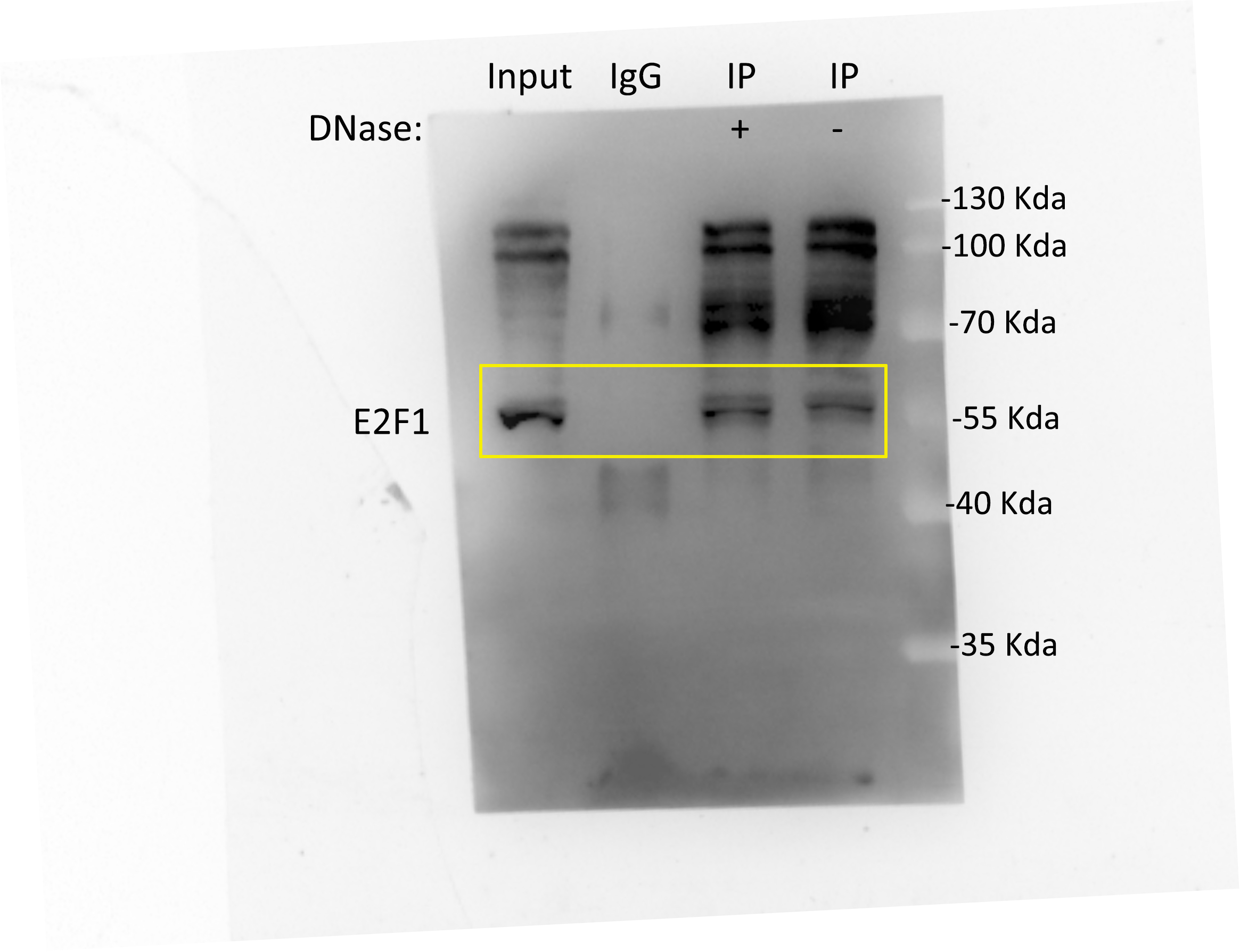

Supplement: Supplementary file 12 — Source data Fig. 6 [file 44318_2024_203_MOESM12_ESM.zip › Figure 6/Figure 6C/WB-E2F1.jpg]

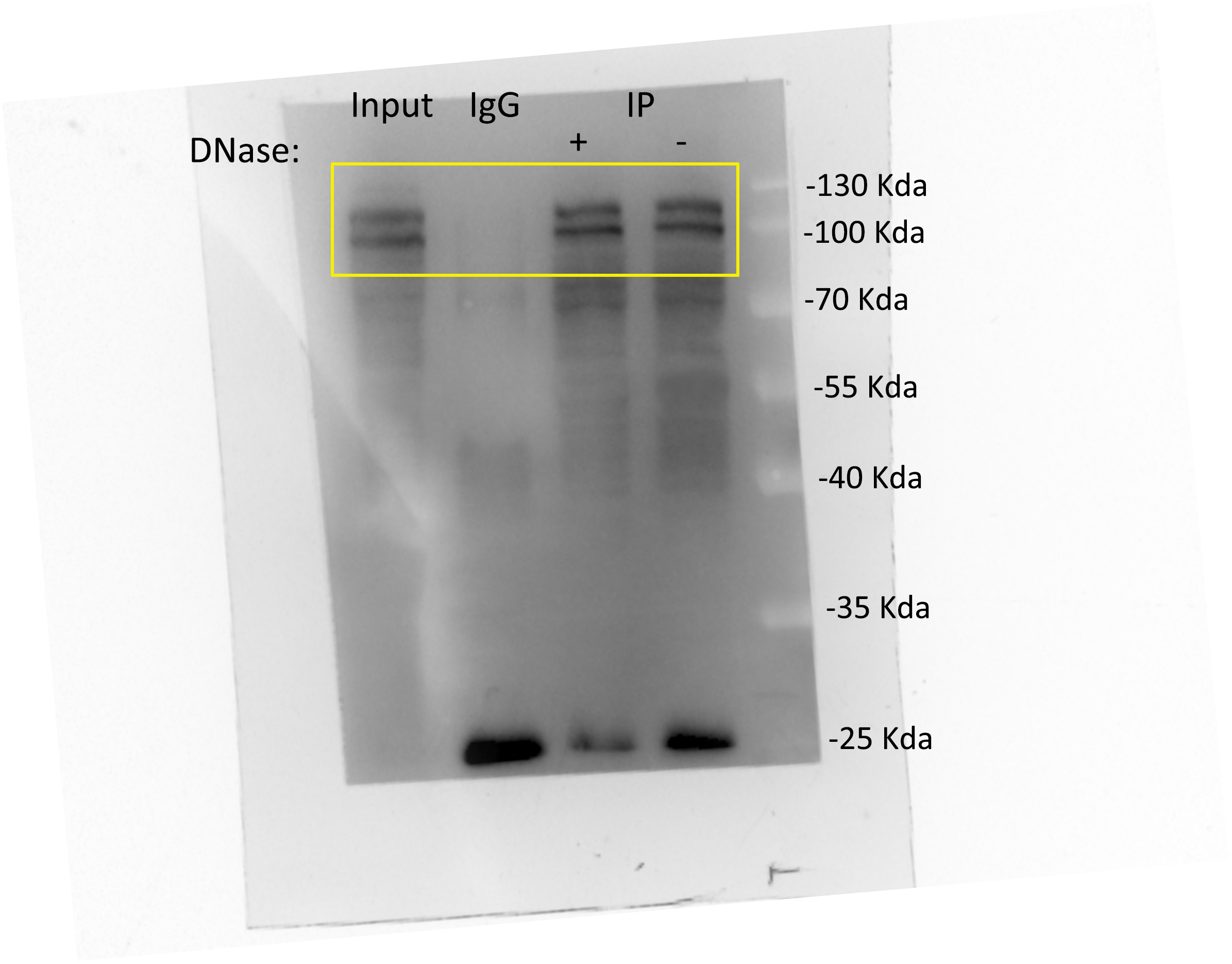

Supplement: Supplementary file 12 — Source data Fig. 6 [file 44318_2024_203_MOESM12_ESM.zip › Figure 6/Figure 6C/WB-HCFC1.jpg]

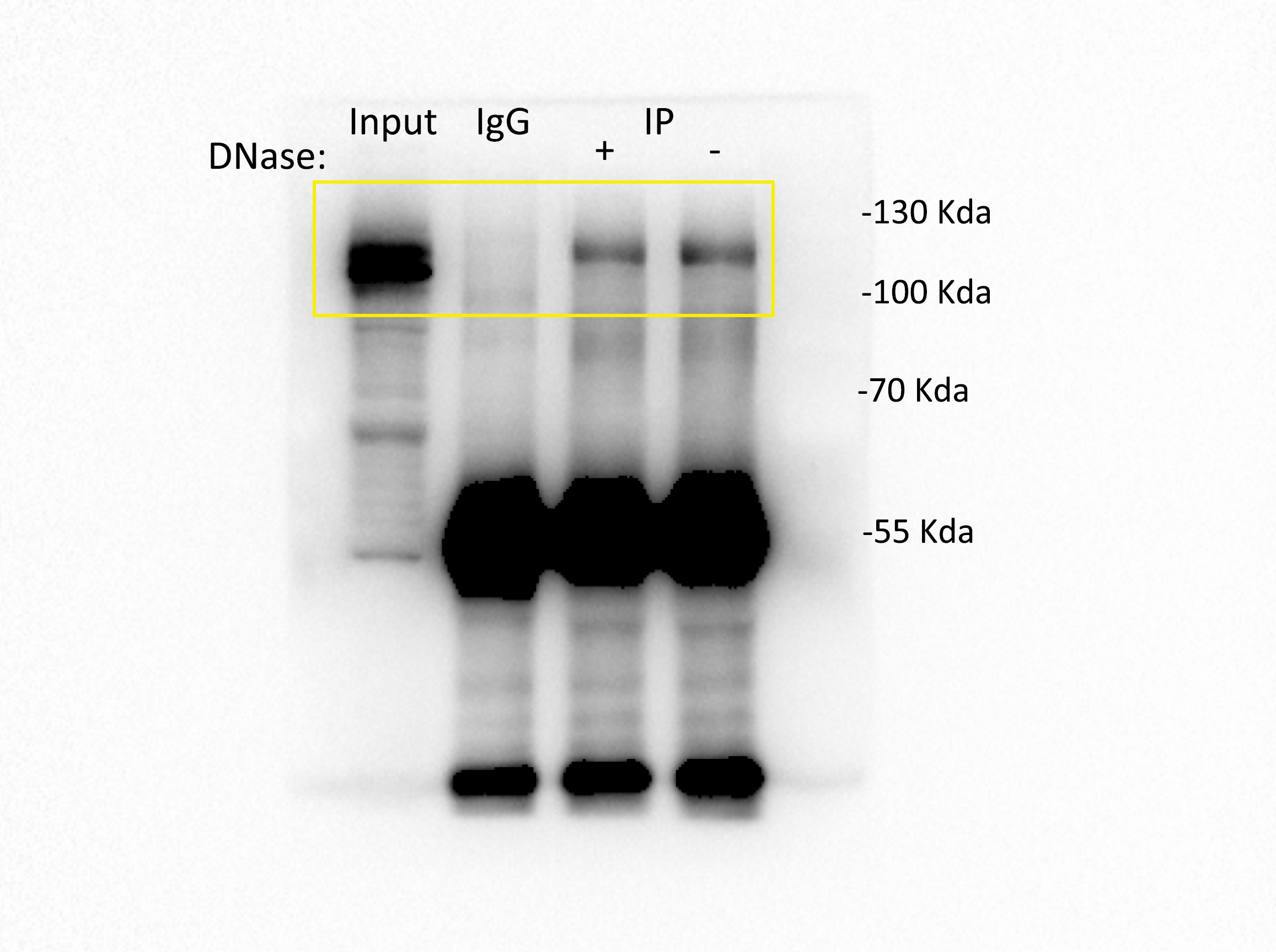

Supplement: Supplementary file 12 — Source data Fig. 6 [file 44318_2024_203_MOESM12_ESM.zip › Figure 6/Figure 6C/WB-KDM2A.jpg]

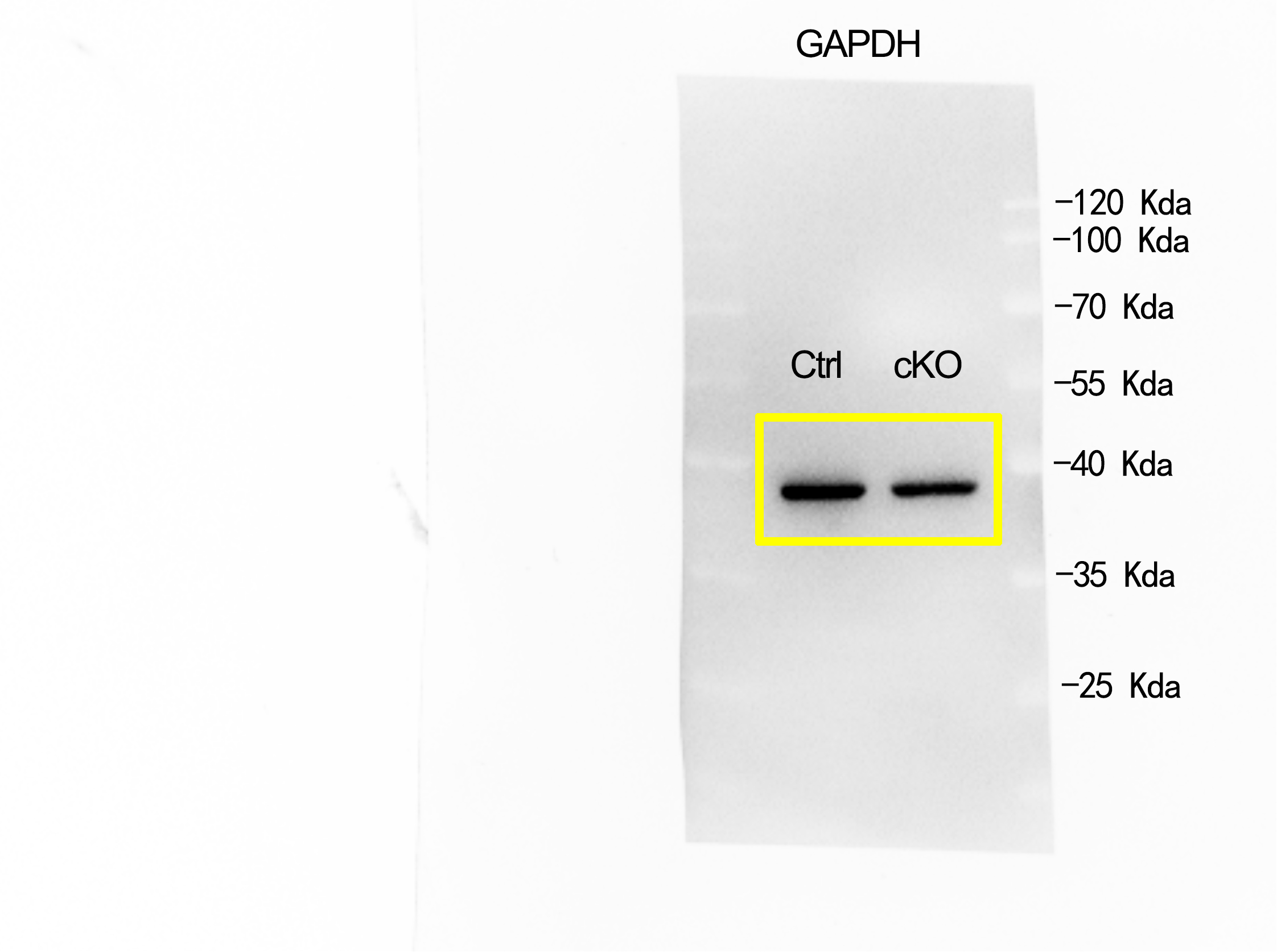

Supplement: Supplementary file 14 — EV and Appendix Figure Source Data [file 44318_2024_203_MOESM14_ESM.zip › Source Data for Expanded View and Appendix/Appendix Figure S1/S1C/WB-GAPDH.jpg]

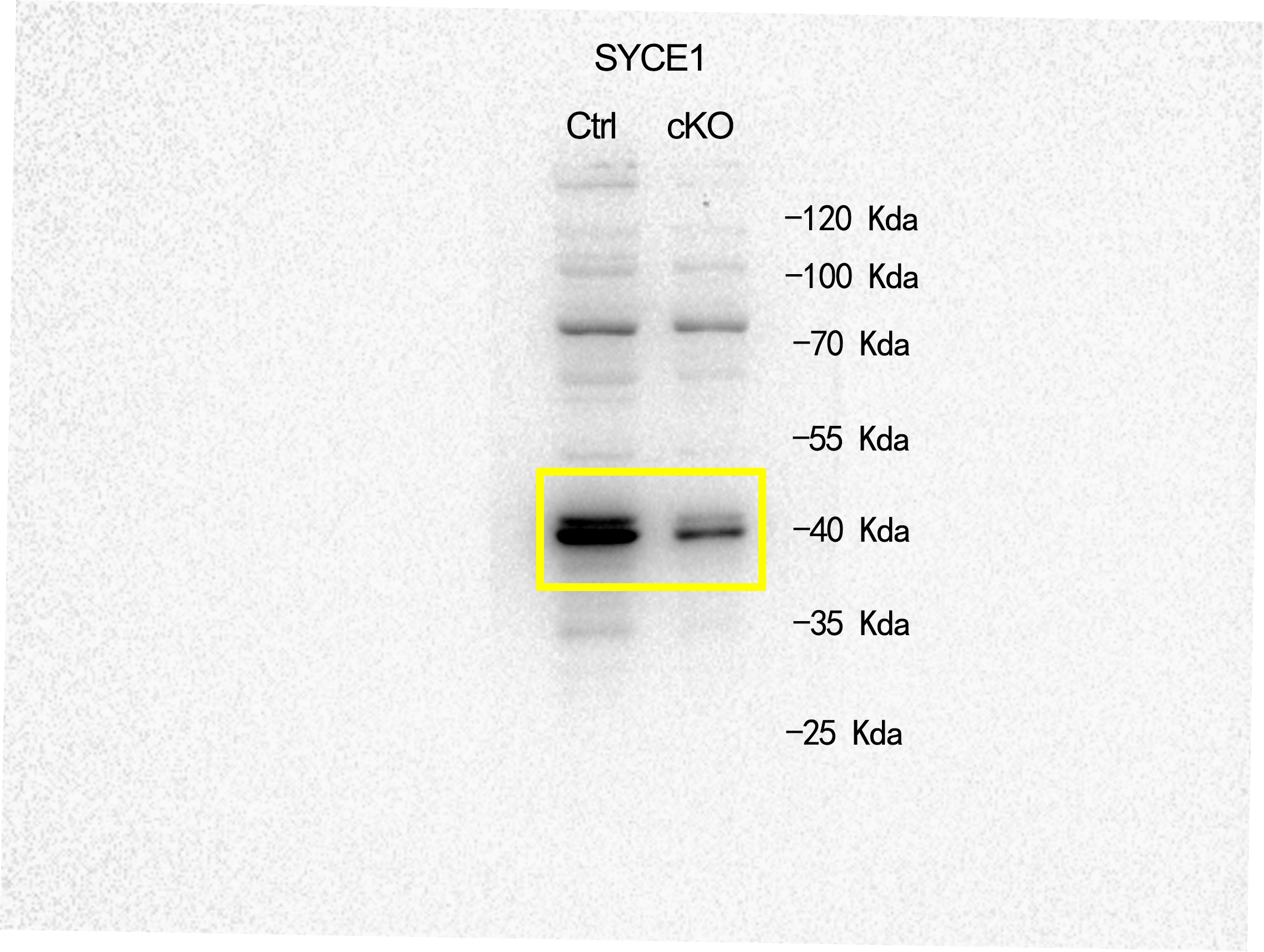

Supplement: Supplementary file 14 — EV and Appendix Figure Source Data [file 44318_2024_203_MOESM14_ESM.zip › Source Data for Expanded View and Appendix/Appendix Figure S1/S1C/WB-SYCE1.jpg]

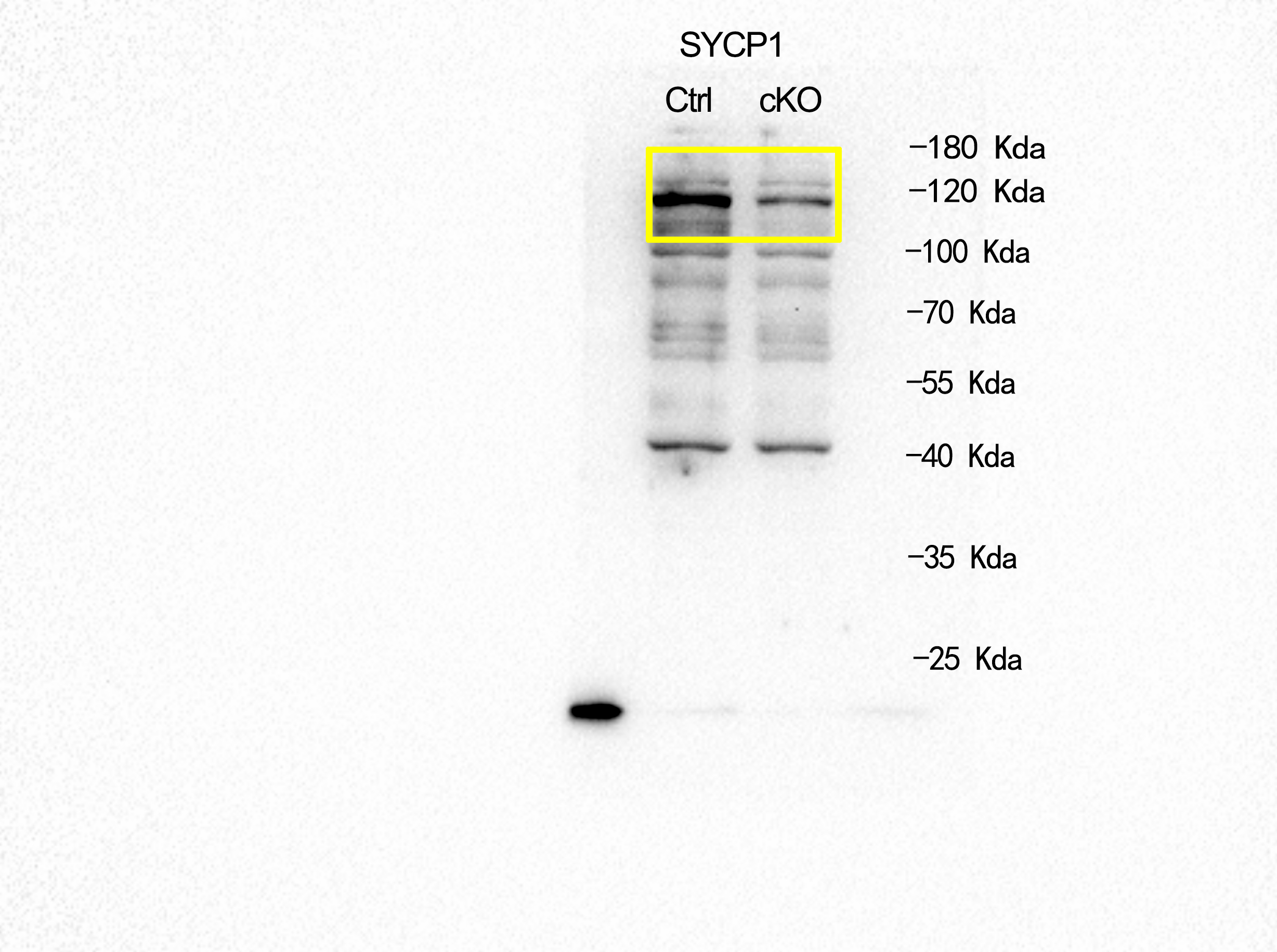

Supplement: Supplementary file 14 — EV and Appendix Figure Source Data [file 44318_2024_203_MOESM14_ESM.zip › Source Data for Expanded View and Appendix/Appendix Figure S1/S1C/WB-SYCP1.jpg]

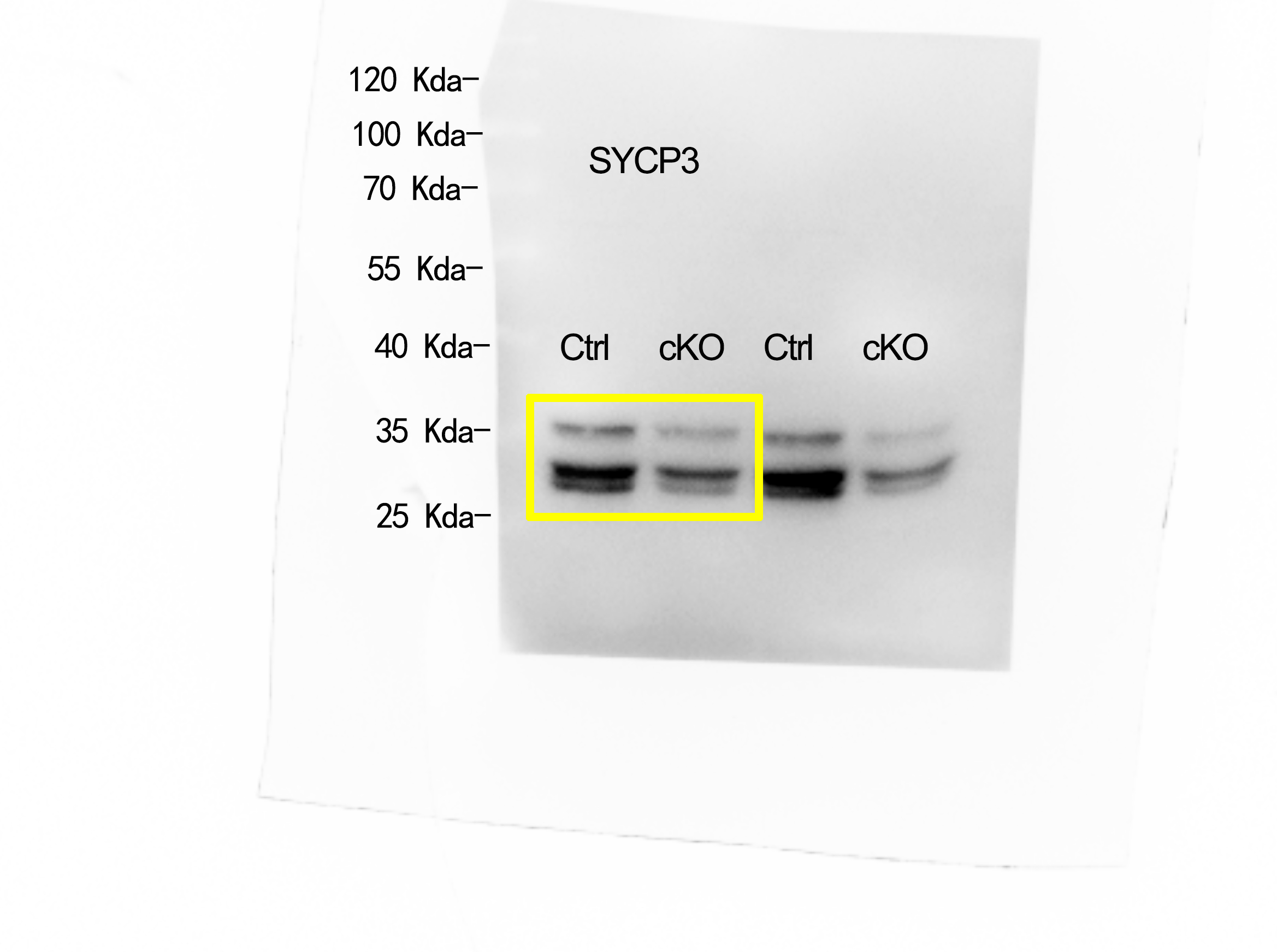

Supplement: Supplementary file 14 — EV and Appendix Figure Source Data [file 44318_2024_203_MOESM14_ESM.zip › Source Data for Expanded View and Appendix/Appendix Figure S1/S1C/WB-SYCP3.jpg]

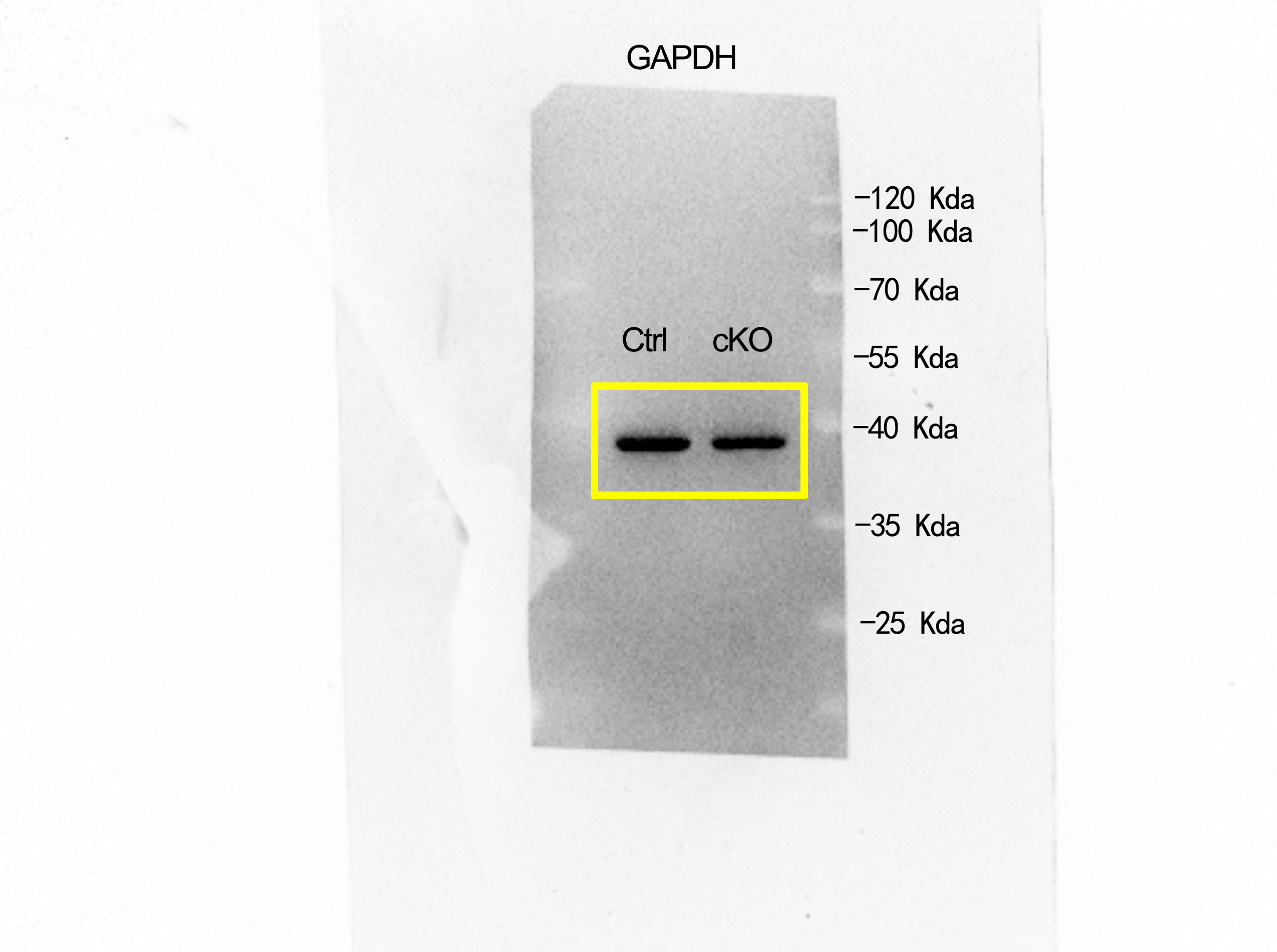

Supplement: Supplementary file 14 — EV and Appendix Figure Source Data [file 44318_2024_203_MOESM14_ESM.zip › Source Data for Expanded View and Appendix/Appendix Figure S1/S1C/WB-Stra8.jpg]

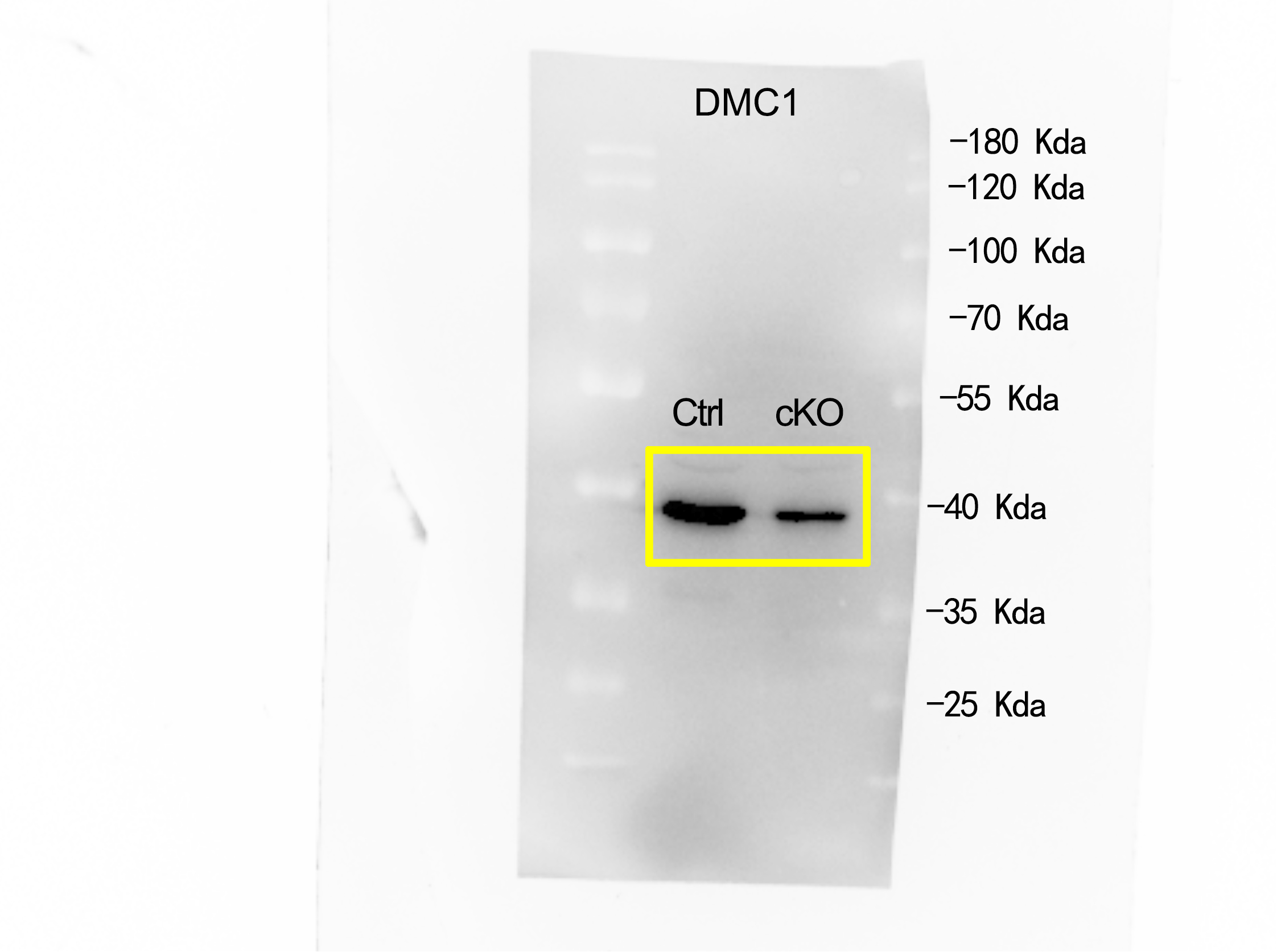

Supplement: Supplementary file 14 — EV and Appendix Figure Source Data [file 44318_2024_203_MOESM14_ESM.zip › Source Data for Expanded View and Appendix/Appendix Figure S1/S1C/WB-DMC1.jpg]

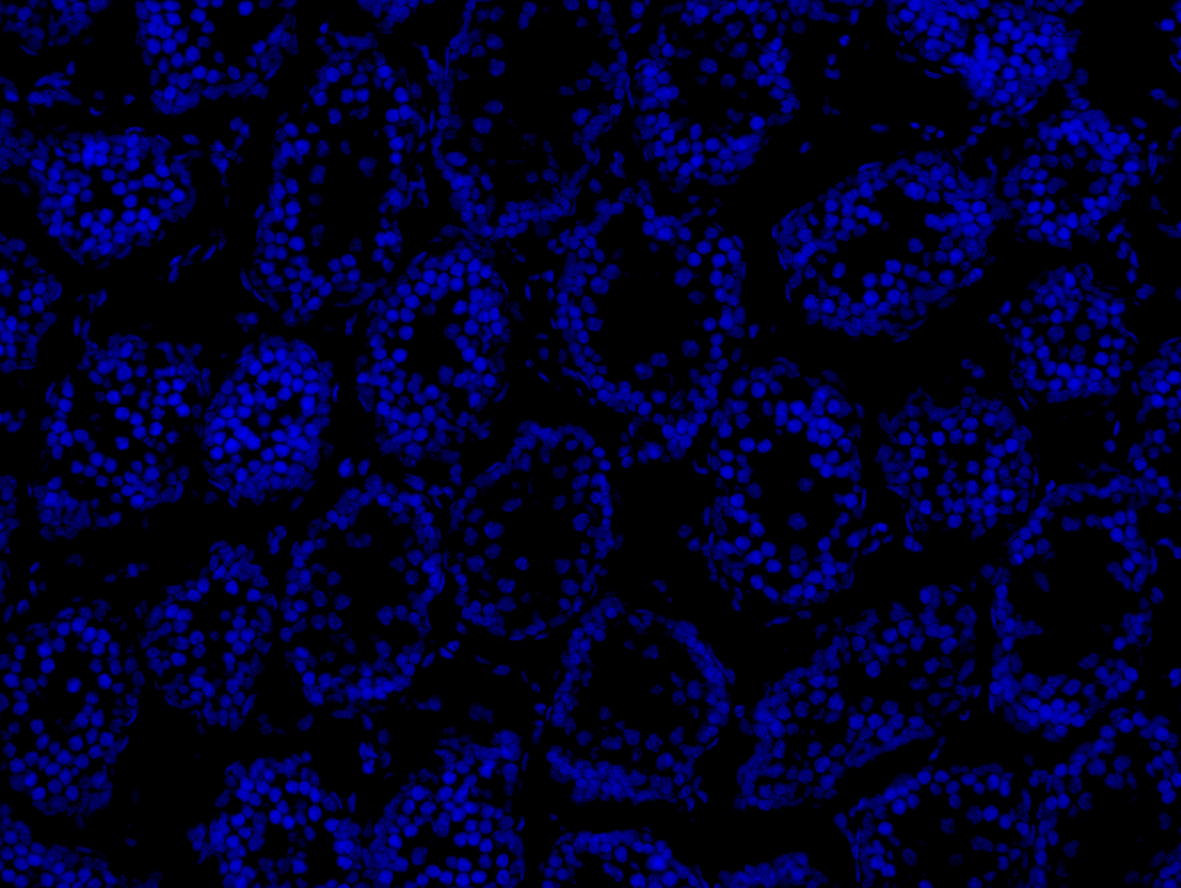

Supplement: Supplementary file 14 — EV and Appendix Figure Source Data [file 44318_2024_203_MOESM14_ESM.zip › Source Data for Expanded View and Appendix/Figure EV4/EV4G/Low magnification of P14 control - DAPI.jpg]

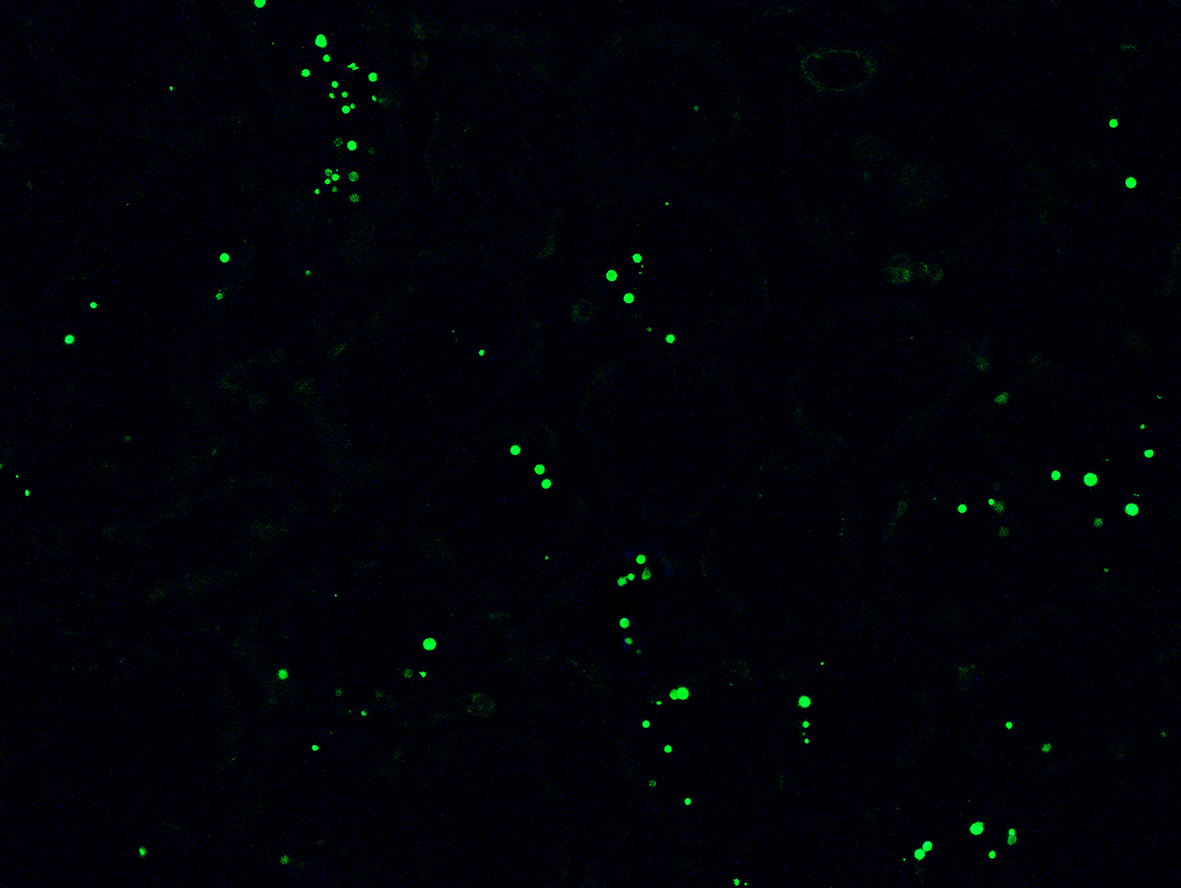

Supplement: Supplementary file 14 — EV and Appendix Figure Source Data [file 44318_2024_203_MOESM14_ESM.zip › Source Data for Expanded View and Appendix/Figure EV4/EV4G/Low magnification of P14 cKO - Tunnel signal.jpg]

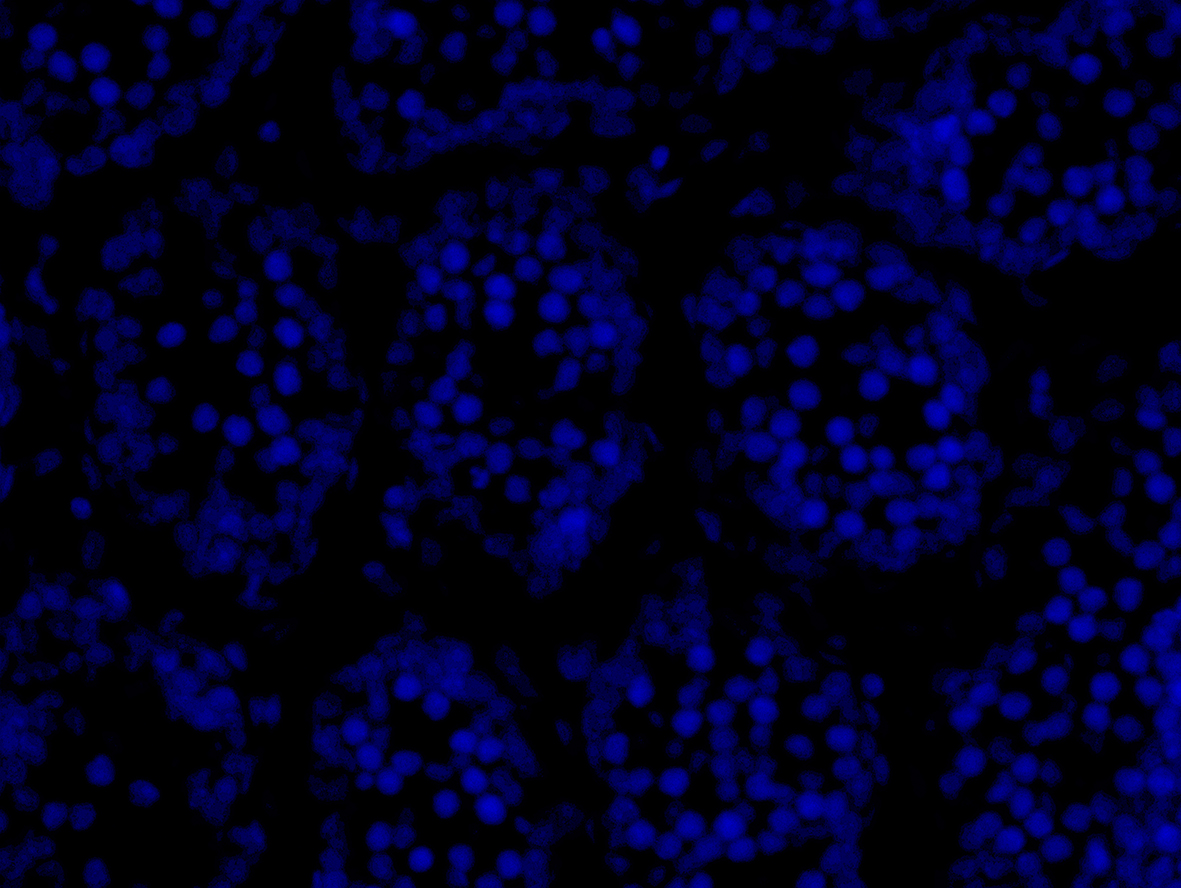

Supplement: Supplementary file 14 — EV and Appendix Figure Source Data [file 44318_2024_203_MOESM14_ESM.zip › Source Data for Expanded View and Appendix/Figure EV4/EV4G/High magnification of P10 control - DAPI.jpg]

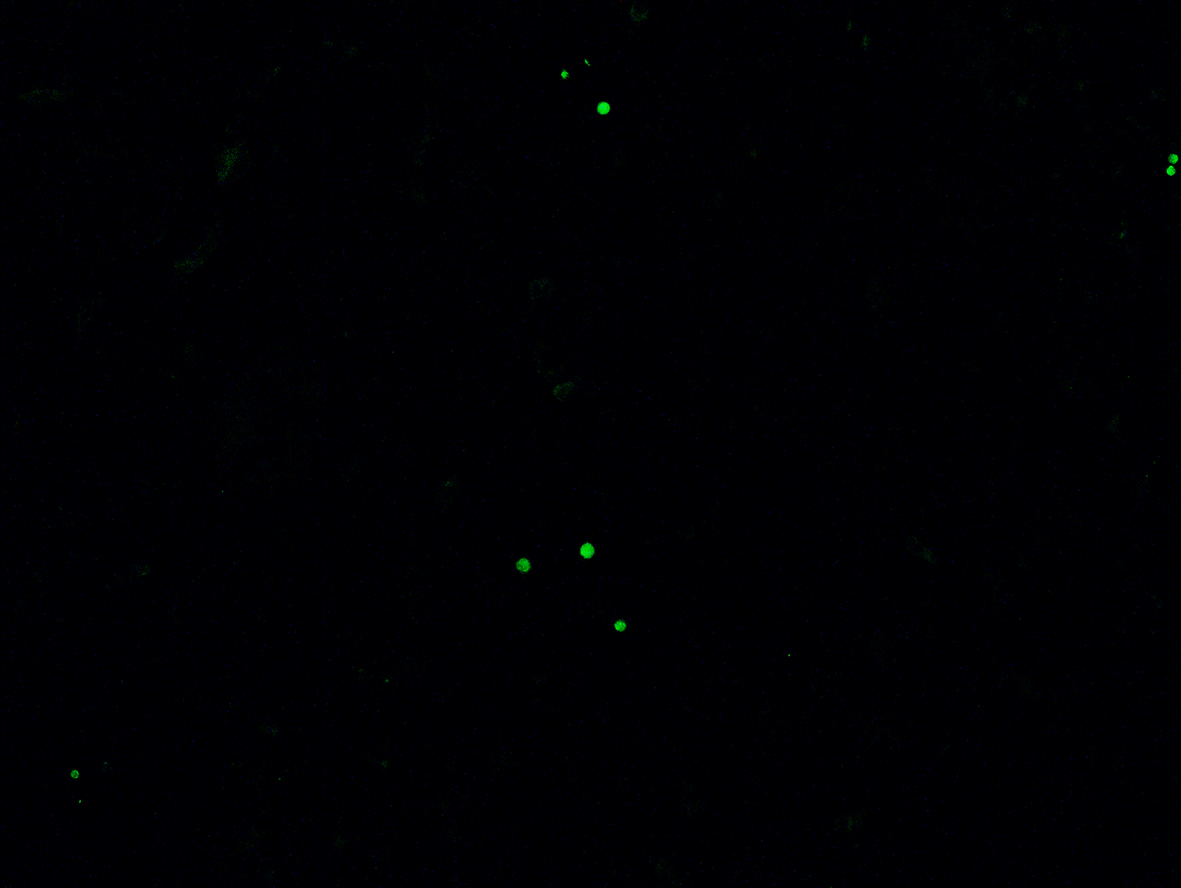

Supplement: Supplementary file 14 — EV and Appendix Figure Source Data [file 44318_2024_203_MOESM14_ESM.zip › Source Data for Expanded View and Appendix/Figure EV4/EV4G/Low magnification of P14 control - Tunnel signal.jpg]

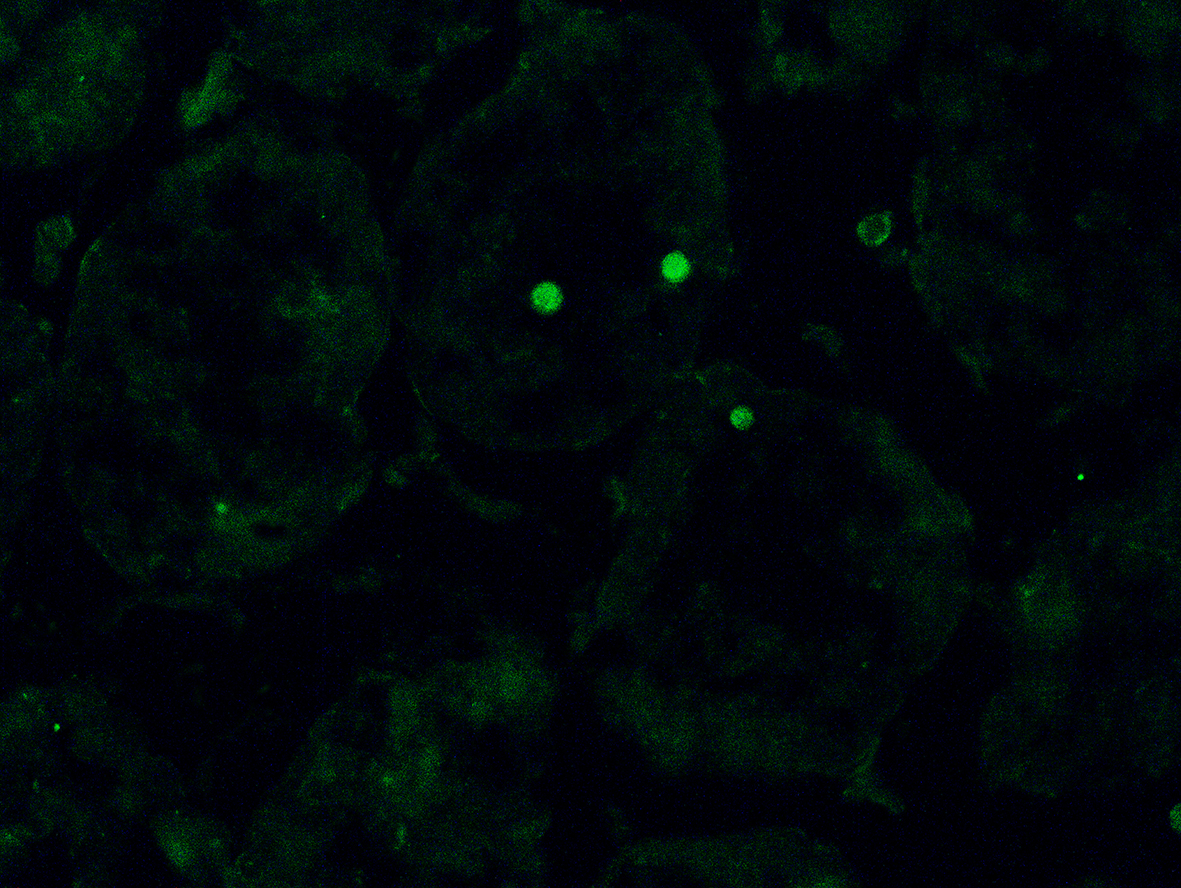

Supplement: Supplementary file 14 — EV and Appendix Figure Source Data [file 44318_2024_203_MOESM14_ESM.zip › Source Data for Expanded View and Appendix/Figure EV4/EV4G/High magnification of P14 control - Tunnel signal.jpg]

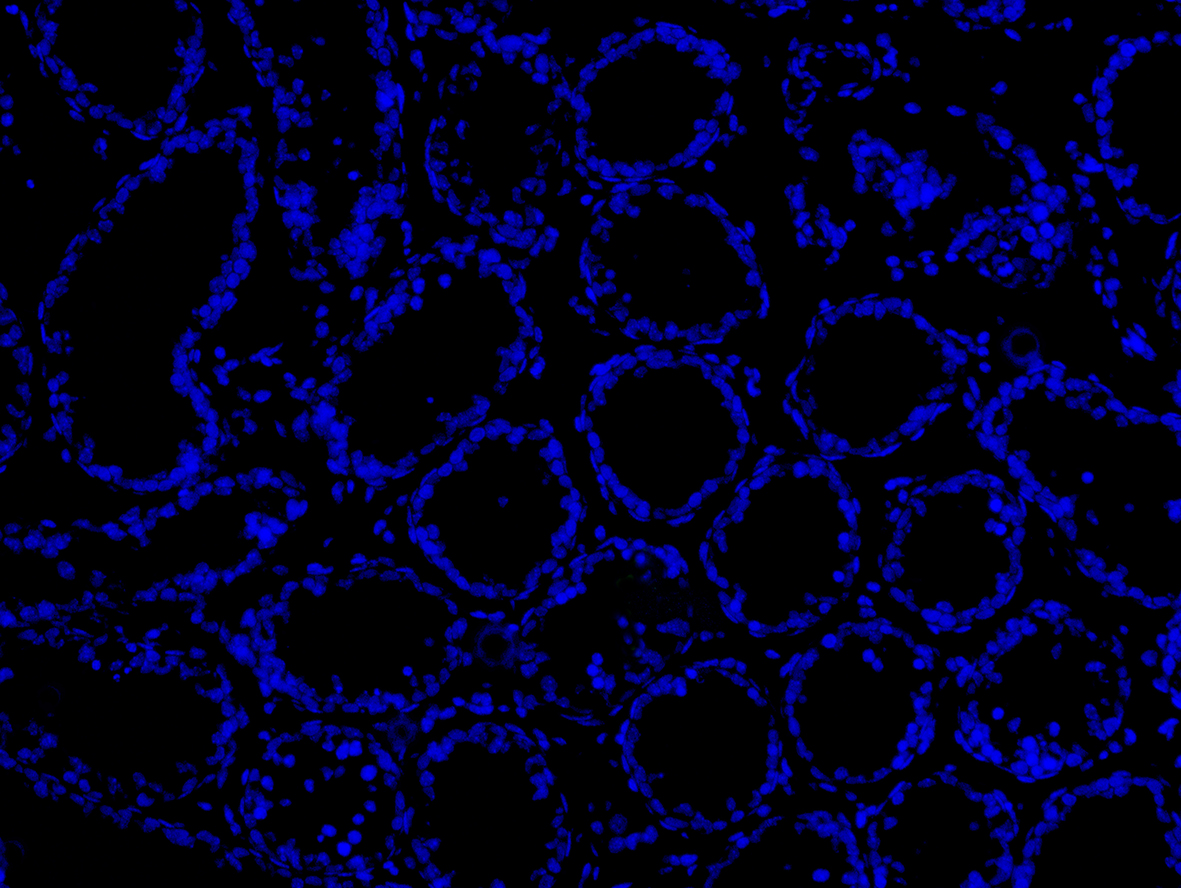

Supplement: Supplementary file 14 — EV and Appendix Figure Source Data [file 44318_2024_203_MOESM14_ESM.zip › Source Data for Expanded View and Appendix/Figure EV4/EV4G/Low magnification of P14 cKO - DAPI.jpg]

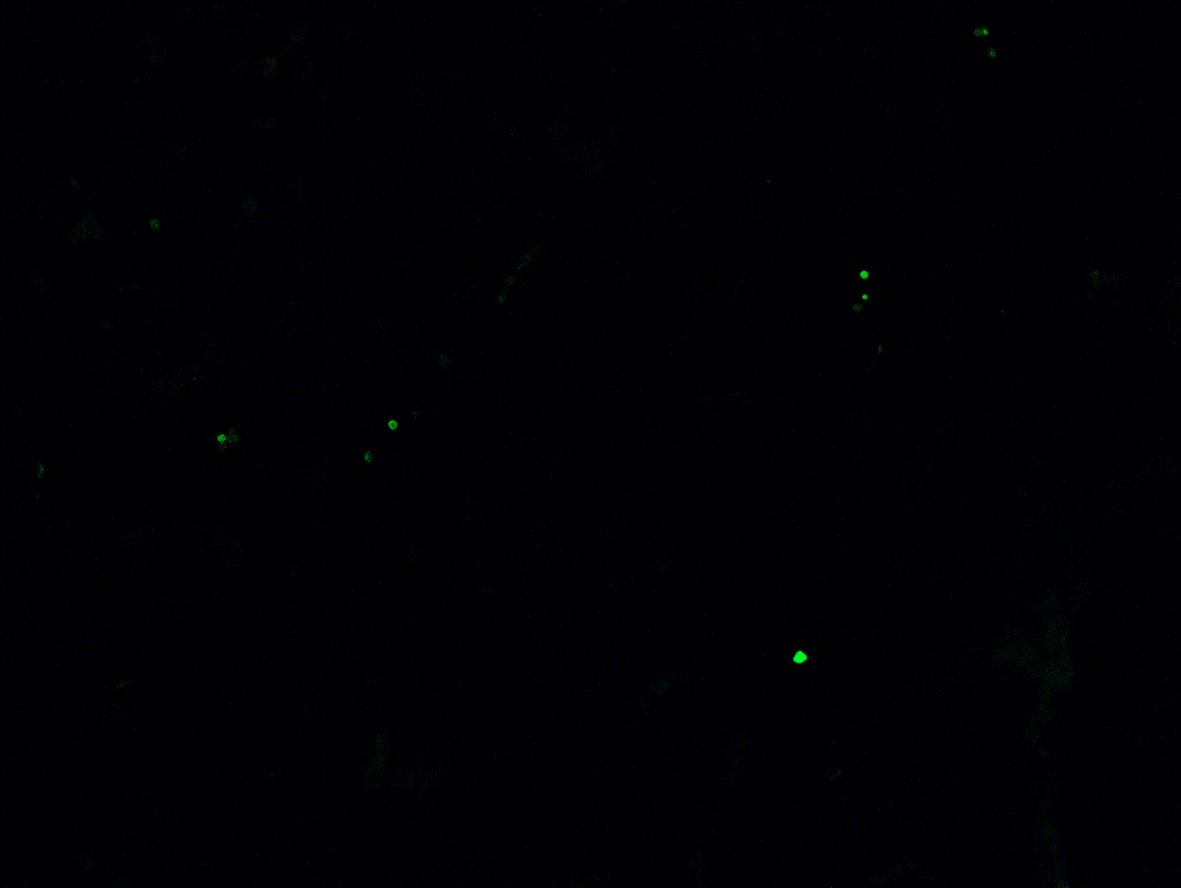

Supplement: Supplementary file 14 — EV and Appendix Figure Source Data [file 44318_2024_203_MOESM14_ESM.zip › Source Data for Expanded View and Appendix/Figure EV4/EV4G/Low magnification of P10 control - Tunnel signal.jpg]

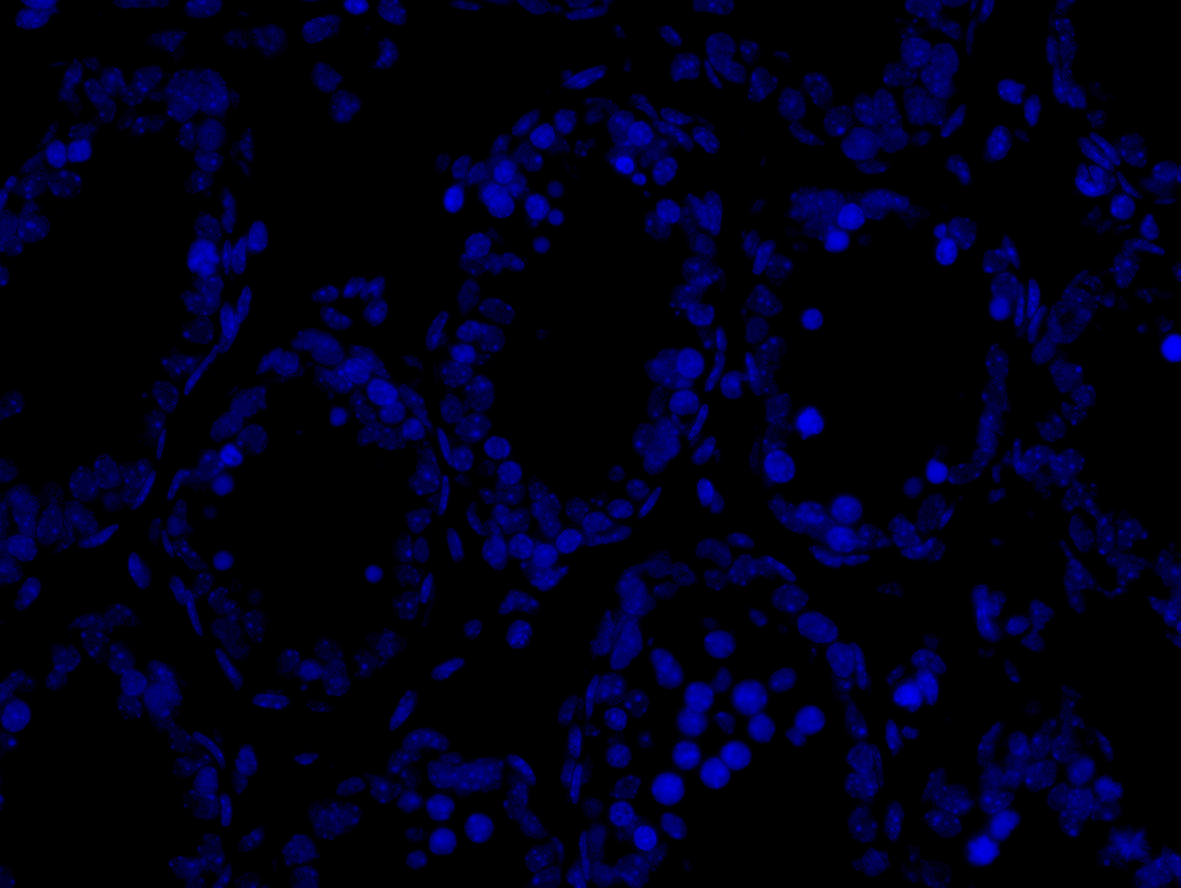

Supplement: Supplementary file 14 — EV and Appendix Figure Source Data [file 44318_2024_203_MOESM14_ESM.zip › Source Data for Expanded View and Appendix/Figure EV4/EV4G/High magnification of P12 Kdm2a cKO - DAPI.jpg]

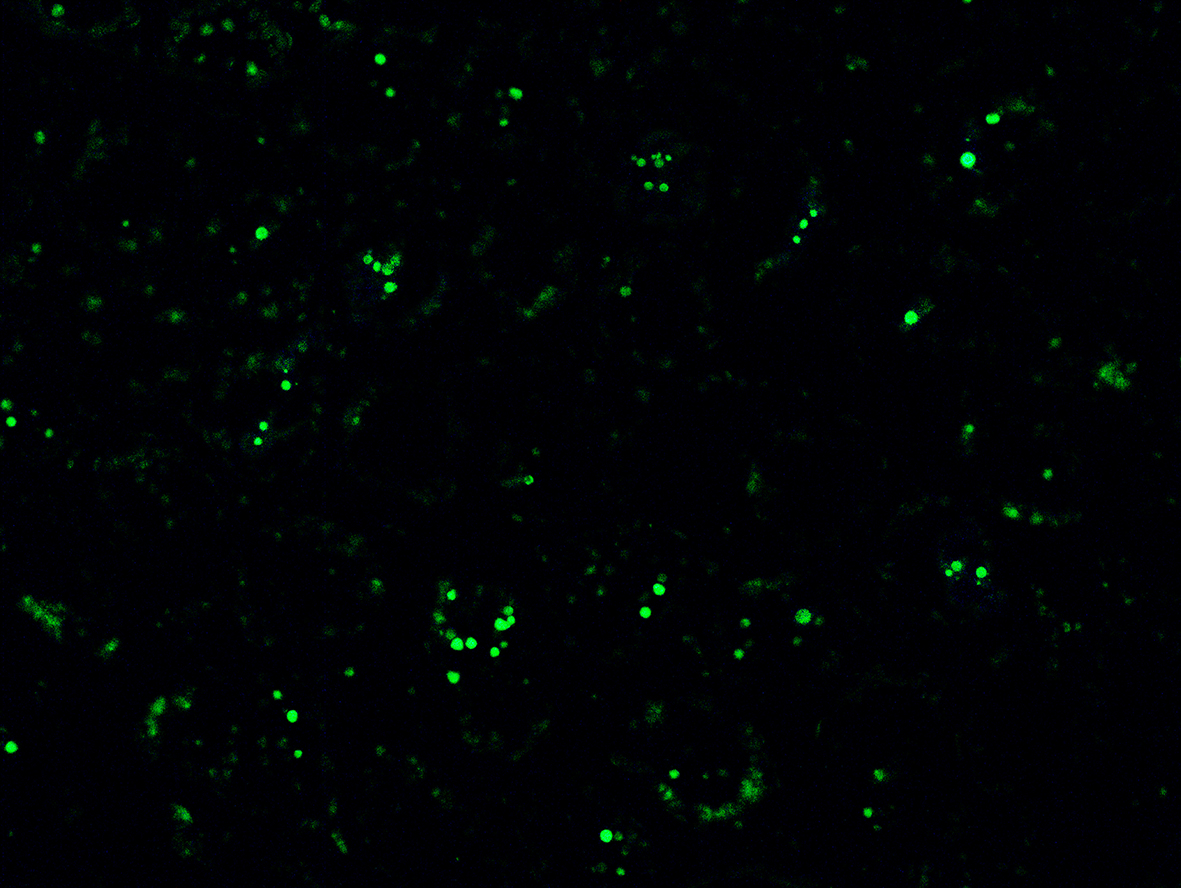

Supplement: Supplementary file 14 — EV and Appendix Figure Source Data [file 44318_2024_203_MOESM14_ESM.zip › Source Data for Expanded View and Appendix/Figure EV4/EV4G/Low magnification of P10 Kdm2a cKO - Tunnel signal.jpg]

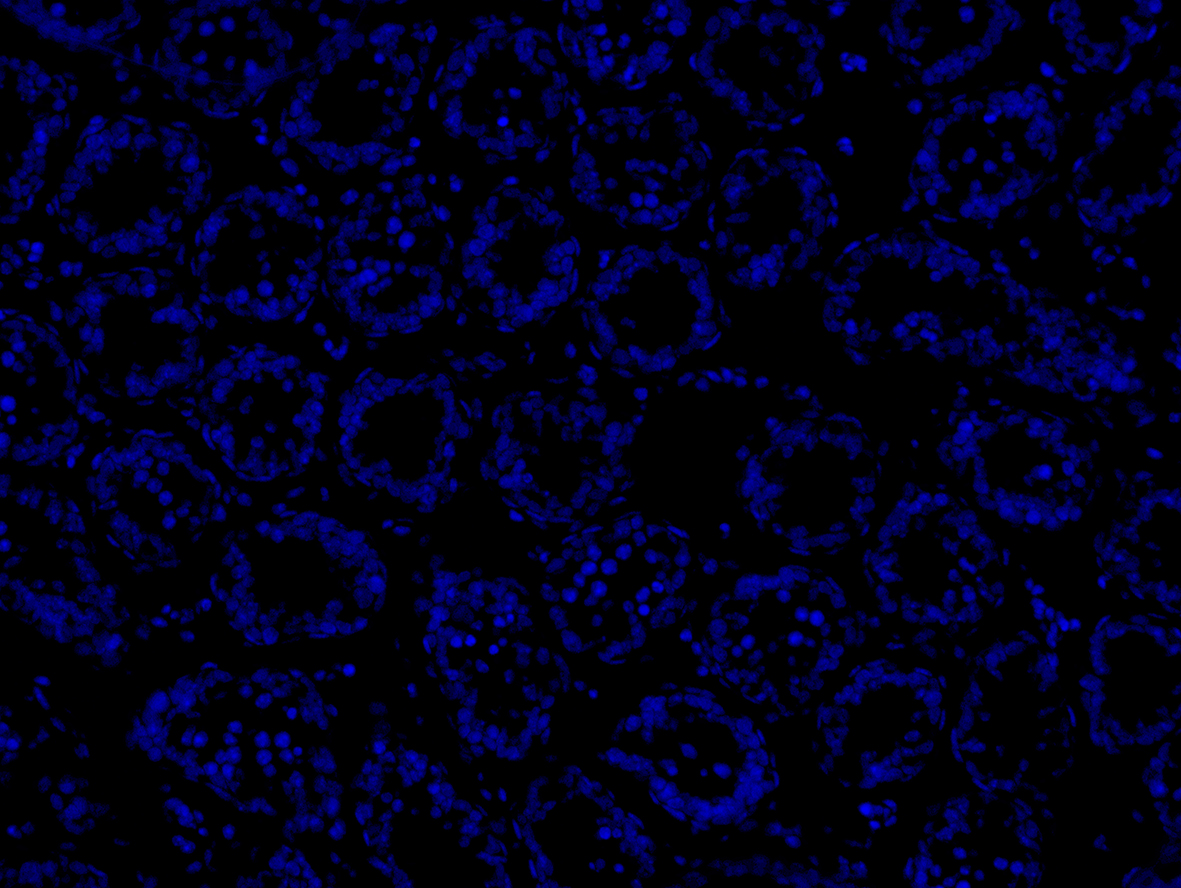

Supplement: Supplementary file 14 — EV and Appendix Figure Source Data [file 44318_2024_203_MOESM14_ESM.zip › Source Data for Expanded View and Appendix/Figure EV4/EV4G/Low magnification of P10 Kdm2a cKO - DAPI.jpg]
