# Supplementary figures and images for: Histone demethylase KDM2A recruits HCFC1 and E2F1 to orchestrate male germ cell meiotic entry and progression (part 4 of 4)
Source: EMBO J. 2024 Aug 19;43(19):4197–227. doi: 10.1038/s44318-024-00203-4 (PMC11448500; doi:10.1038/s44318-024-00203-4)

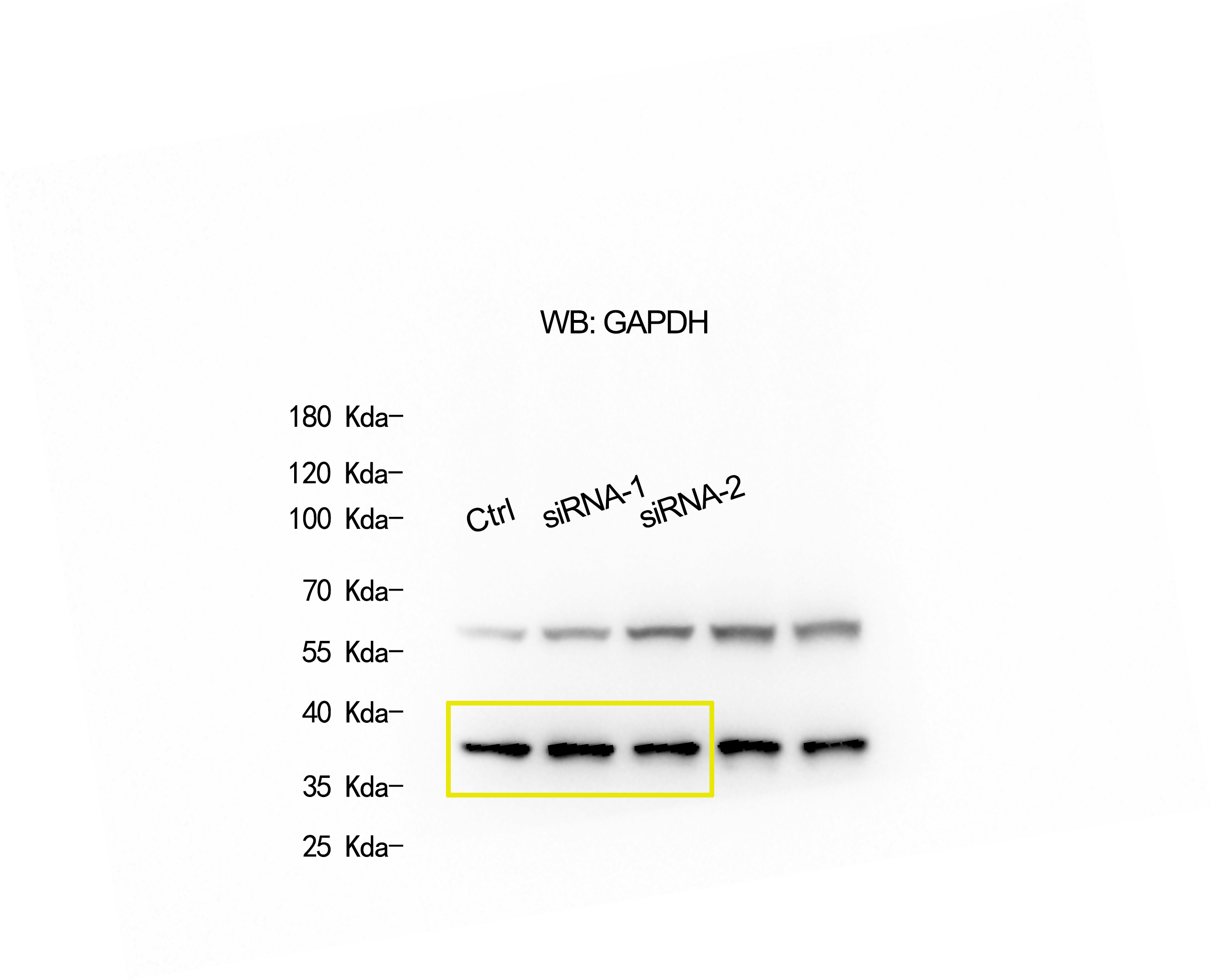

Supplement: Supplementary file 14 — EV and Appendix Figure Source Data [file 44318_2024_203_MOESM14_ESM.zip › Source Data for Expanded View and Appendix/Appendix Figure S4/S4I/WB-GAPDH.jpg]

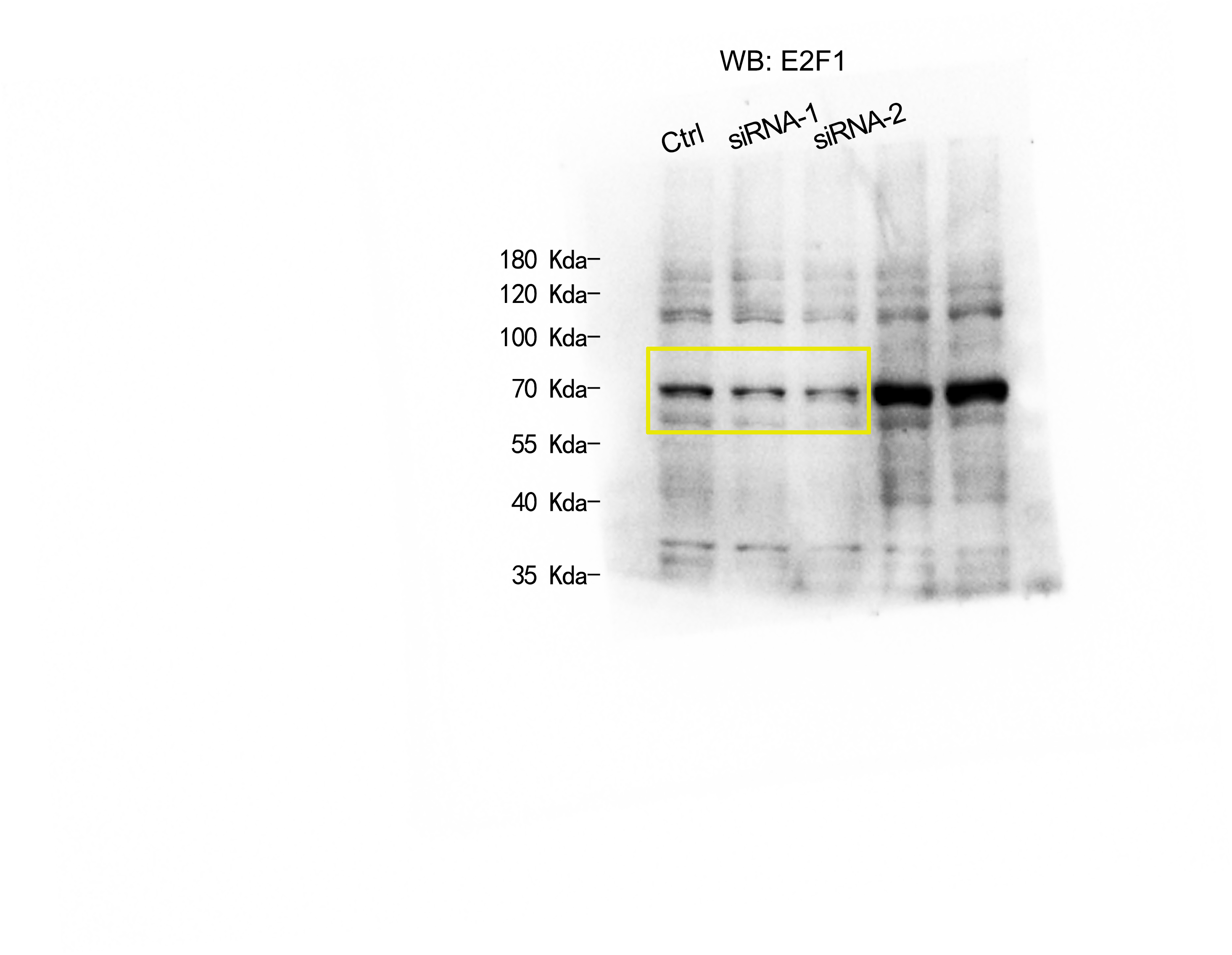

Supplement: Supplementary file 14 — EV and Appendix Figure Source Data [file 44318_2024_203_MOESM14_ESM.zip › Source Data for Expanded View and Appendix/Appendix Figure S4/S4I/WB-E2F1.jpg]

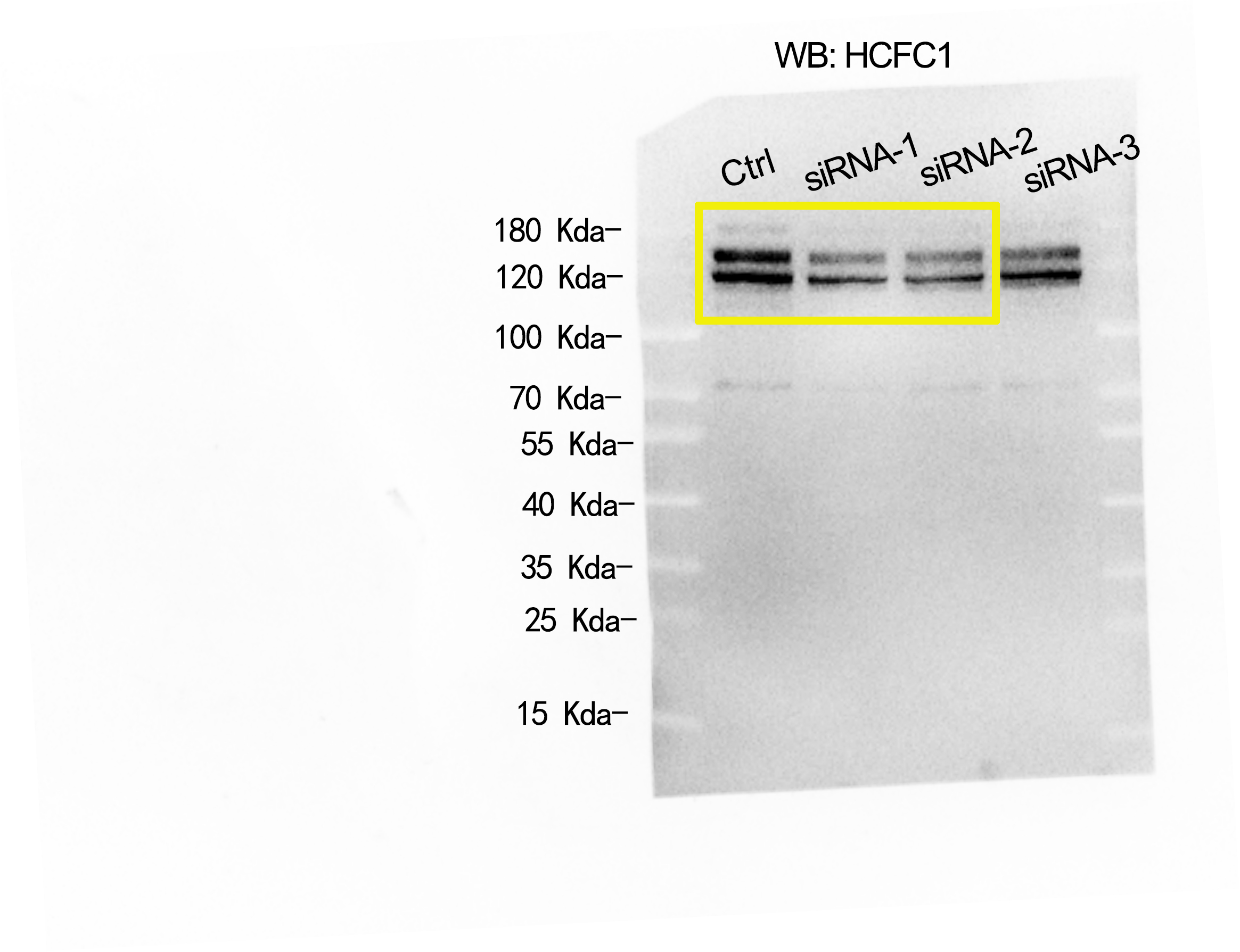

Supplement: Supplementary file 14 — EV and Appendix Figure Source Data [file 44318_2024_203_MOESM14_ESM.zip › Source Data for Expanded View and Appendix/Appendix Figure S4/S4H/WB-HCFC1.jpg]

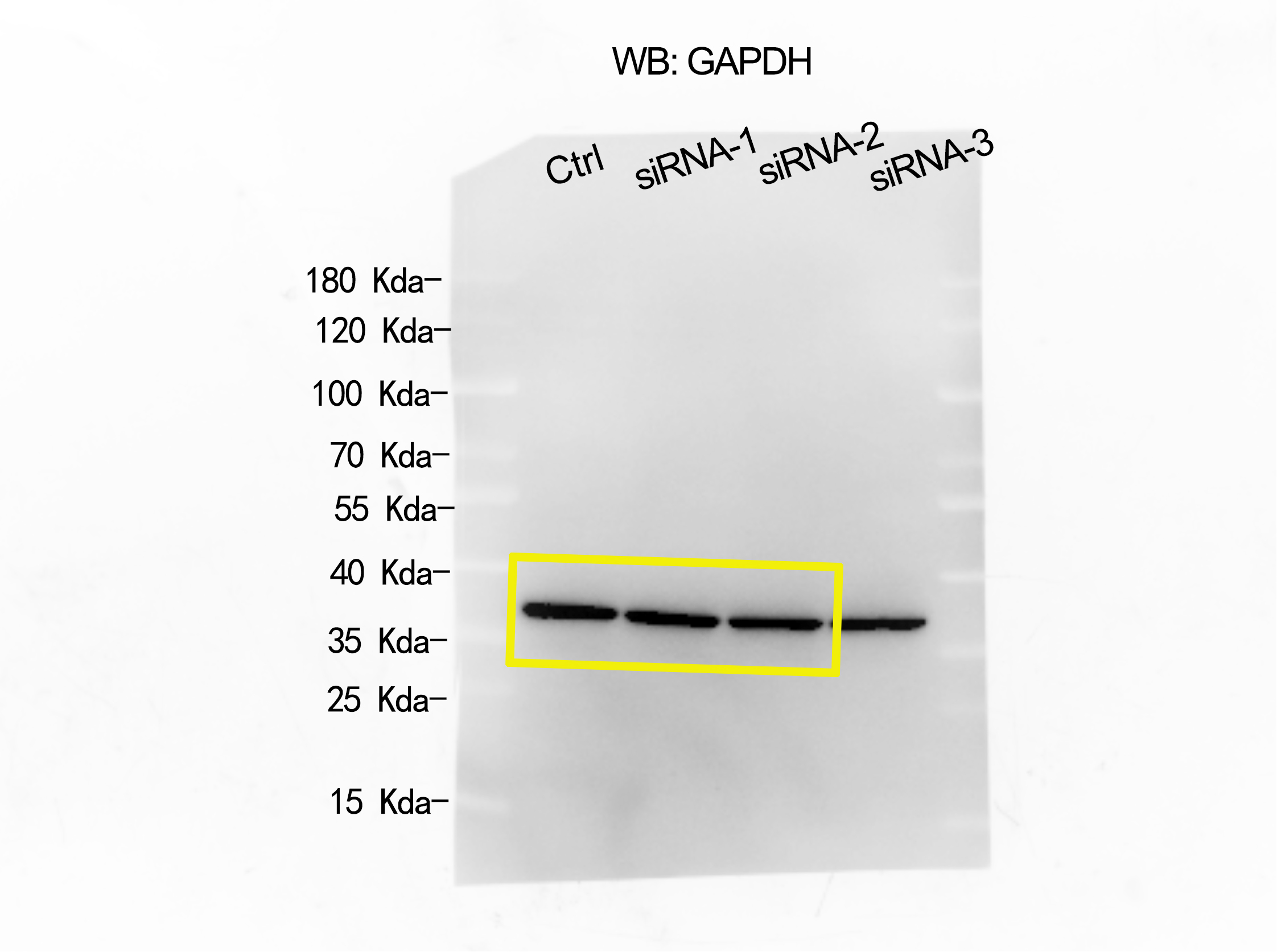

Supplement: Supplementary file 14 — EV and Appendix Figure Source Data [file 44318_2024_203_MOESM14_ESM.zip › Source Data for Expanded View and Appendix/Appendix Figure S4/S4H/source data-GAPDH.jpg]

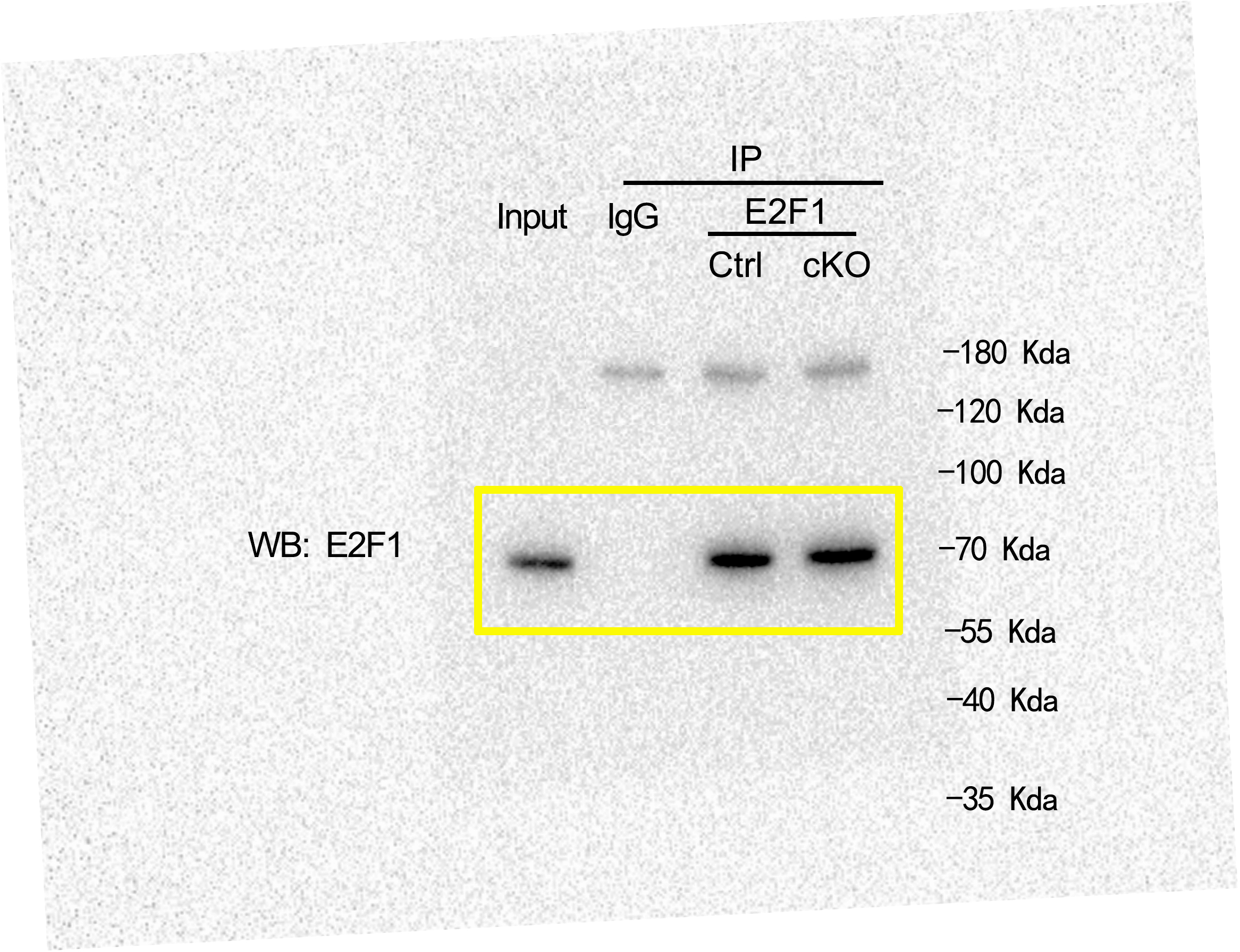

Supplement: Supplementary file 14 — EV and Appendix Figure Source Data [file 44318_2024_203_MOESM14_ESM.zip › Source Data for Expanded View and Appendix/Appendix Figure S4/S4A/WB-E2F1.jpg]

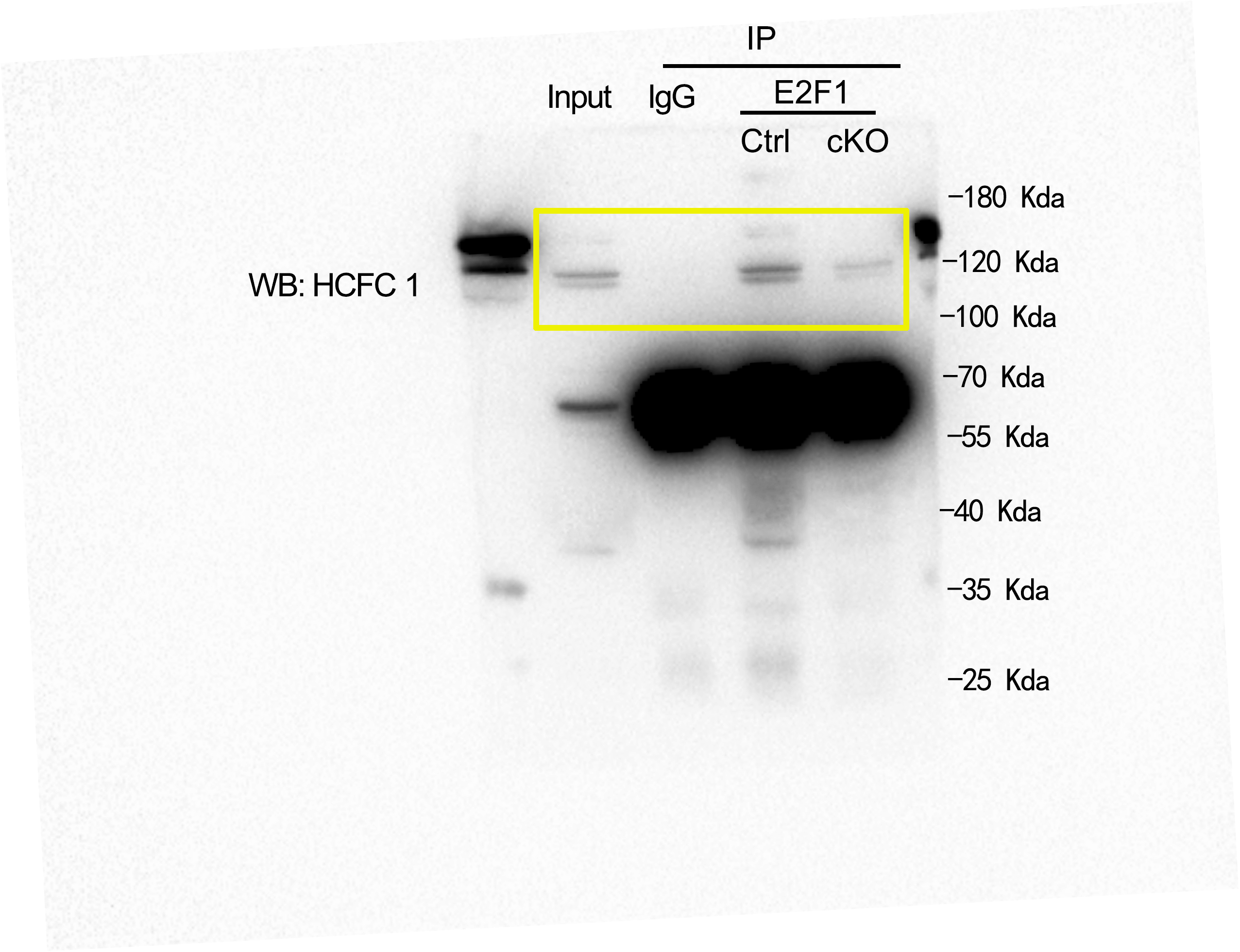

Supplement: Supplementary file 14 — EV and Appendix Figure Source Data [file 44318_2024_203_MOESM14_ESM.zip › Source Data for Expanded View and Appendix/Appendix Figure S4/S4A/WB-HCFC1.jpg]

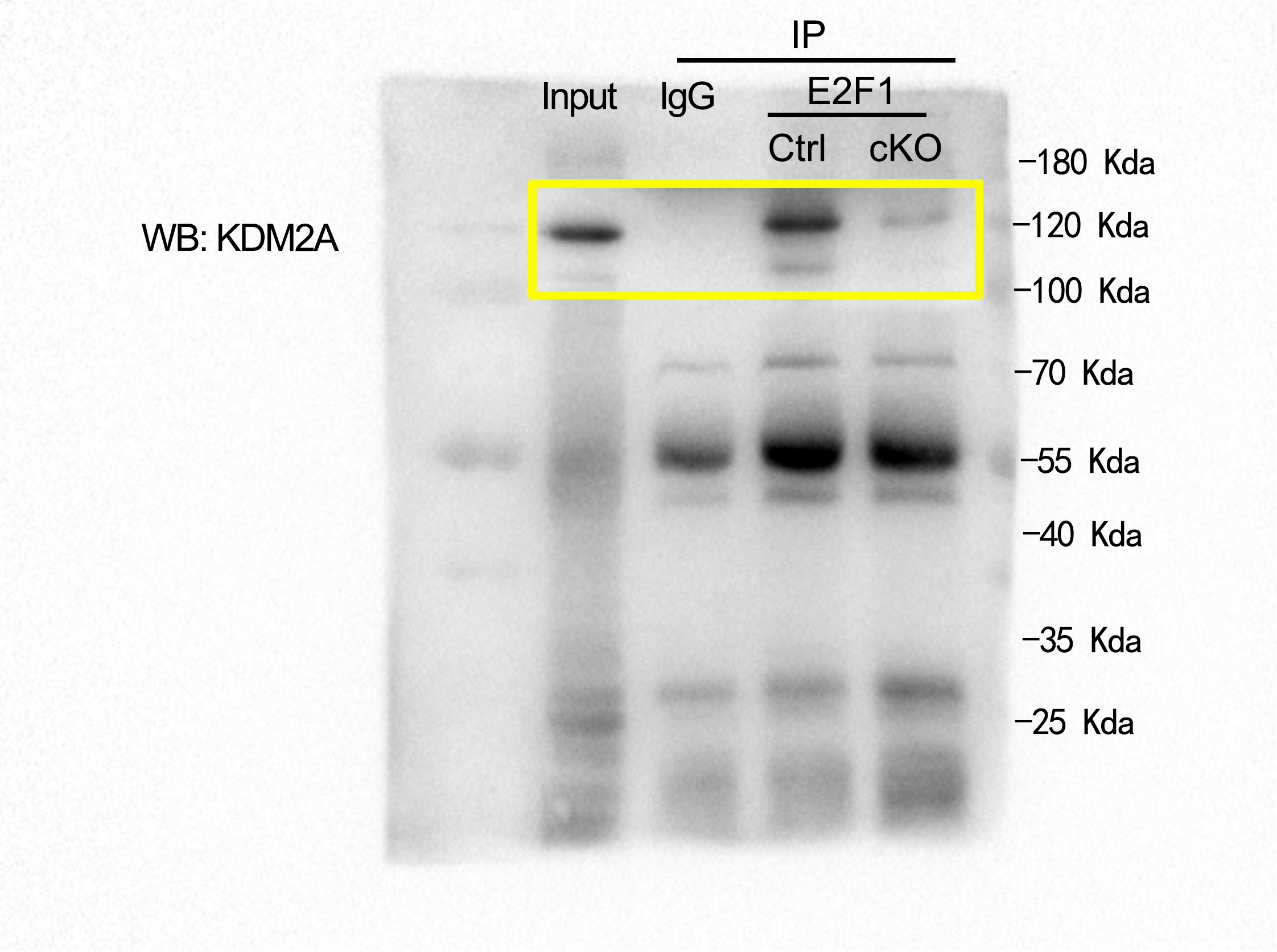

Supplement: Supplementary file 14 — EV and Appendix Figure Source Data [file 44318_2024_203_MOESM14_ESM.zip › Source Data for Expanded View and Appendix/Appendix Figure S4/S4A/WB-KDM2A.jpg]

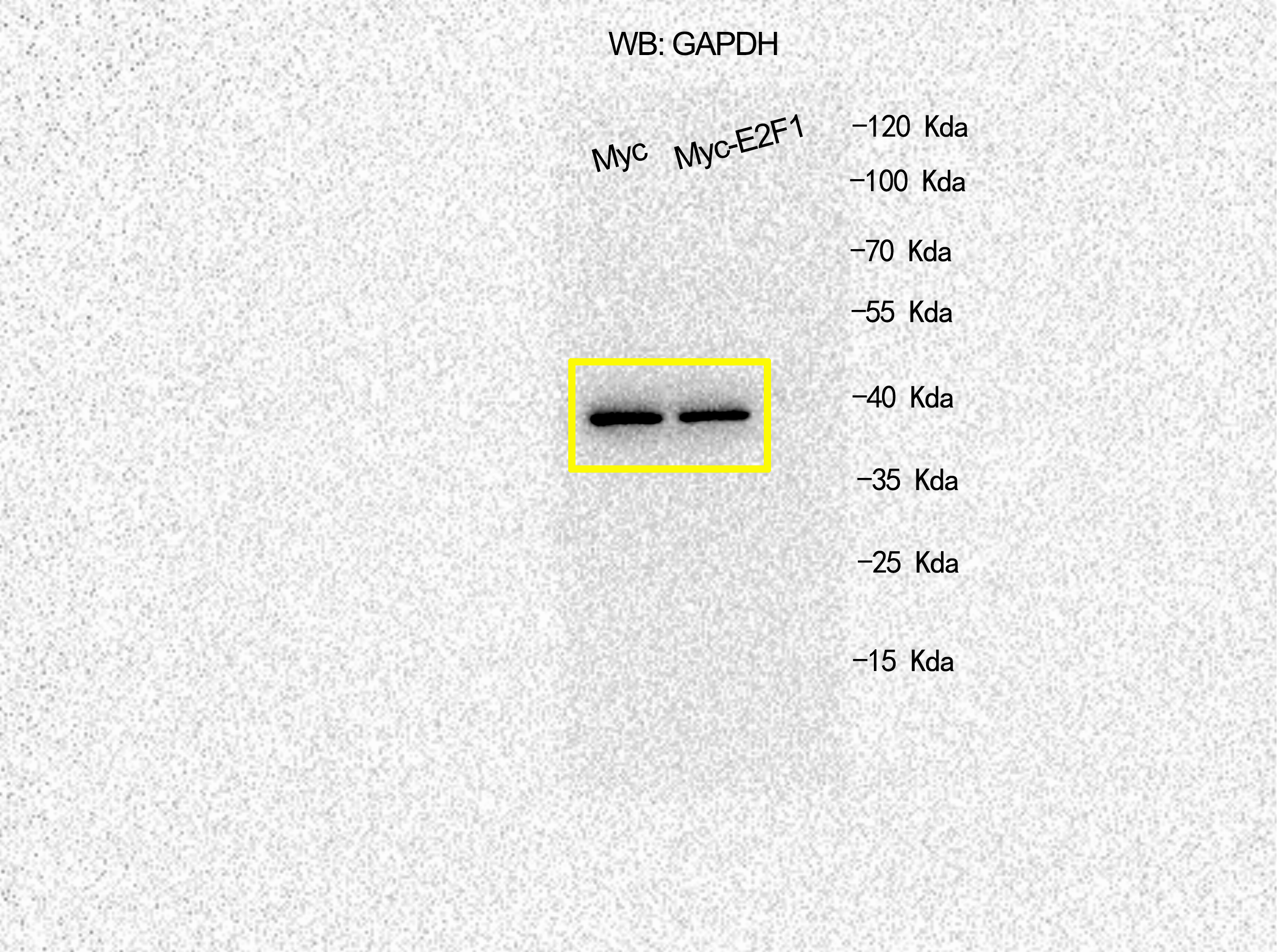

Supplement: Supplementary file 14 — EV and Appendix Figure Source Data [file 44318_2024_203_MOESM14_ESM.zip › Source Data for Expanded View and Appendix/Appendix Figure S4/S4F/WB-GAPDH.jpg]

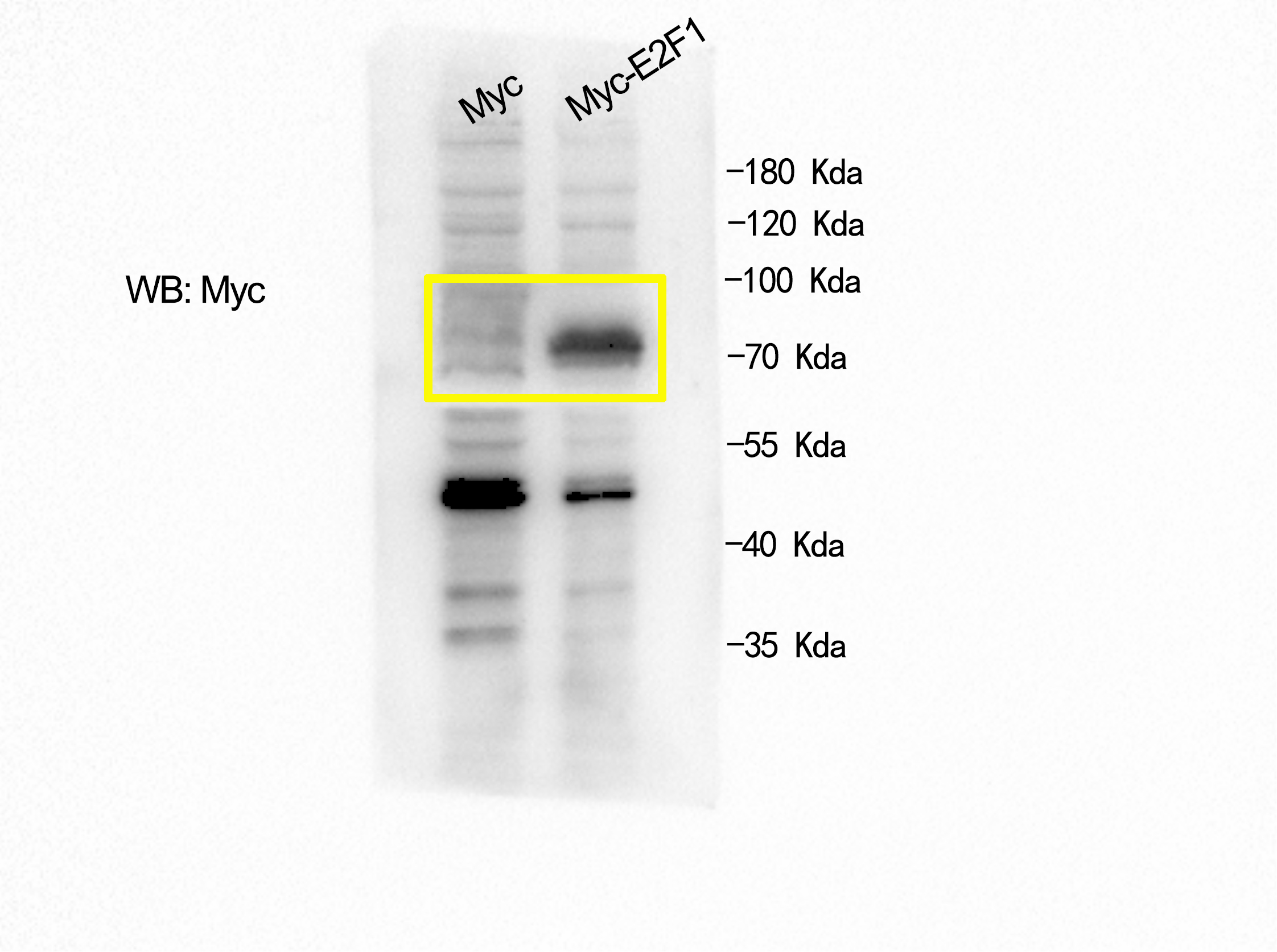

Supplement: Supplementary file 14 — EV and Appendix Figure Source Data [file 44318_2024_203_MOESM14_ESM.zip › Source Data for Expanded View and Appendix/Appendix Figure S4/S4F/WB-Myc.jpg]

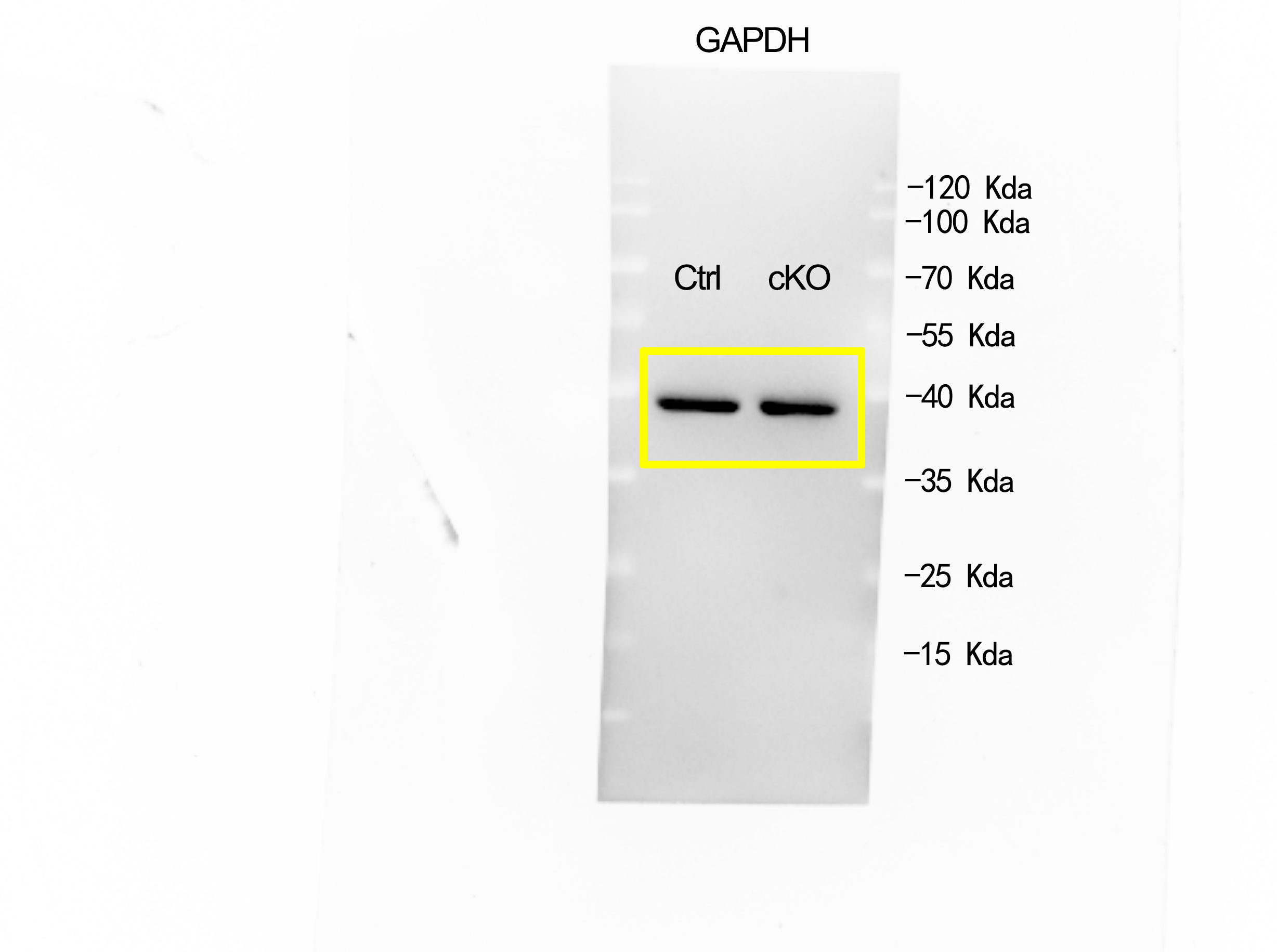

Supplement: Supplementary file 14 — EV and Appendix Figure Source Data [file 44318_2024_203_MOESM14_ESM.zip › Source Data for Expanded View and Appendix/Appendix Figure S3/S3B/WB-GAPDH.jpg]

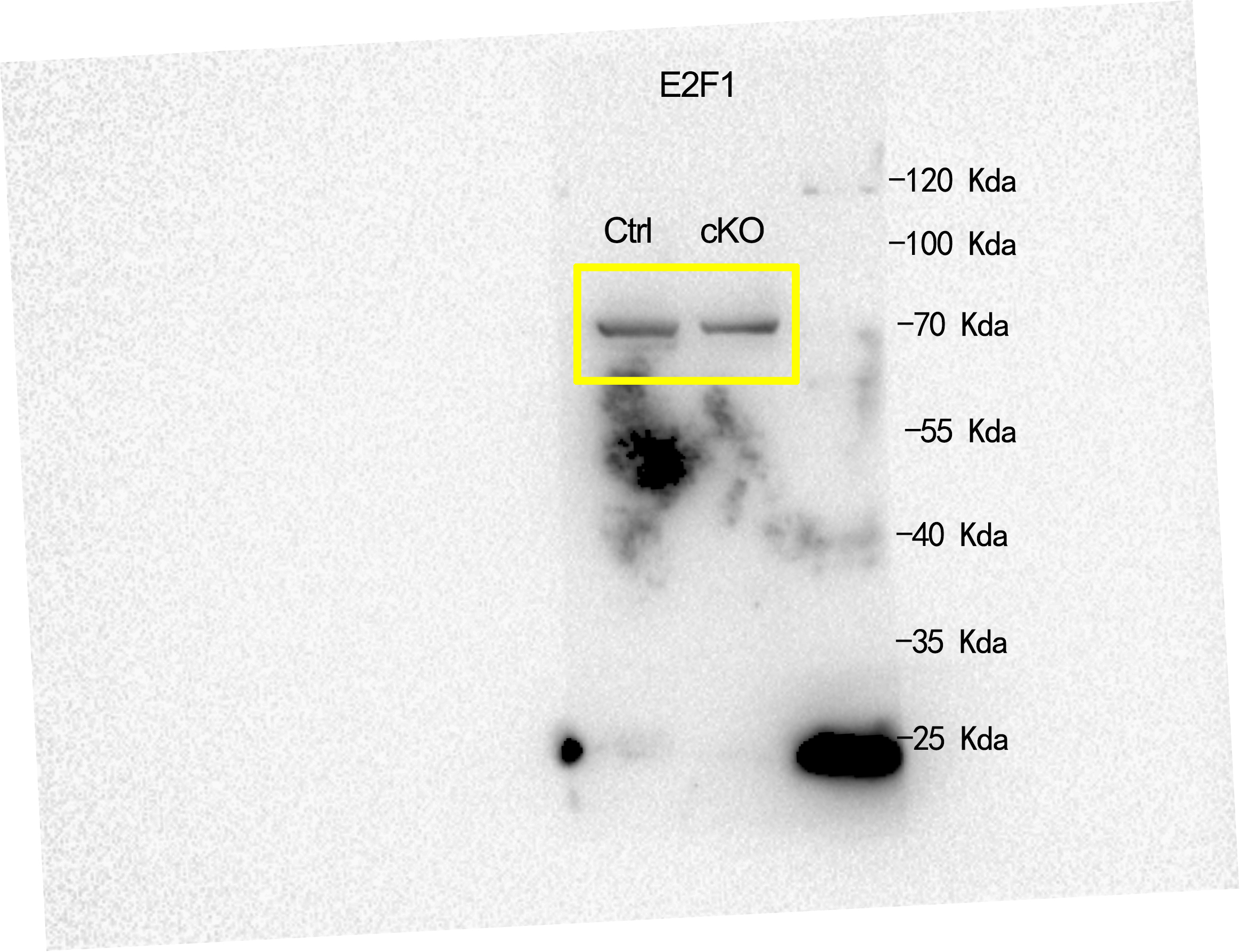

Supplement: Supplementary file 14 — EV and Appendix Figure Source Data [file 44318_2024_203_MOESM14_ESM.zip › Source Data for Expanded View and Appendix/Appendix Figure S3/S3B/WB-E2F1.jpg]

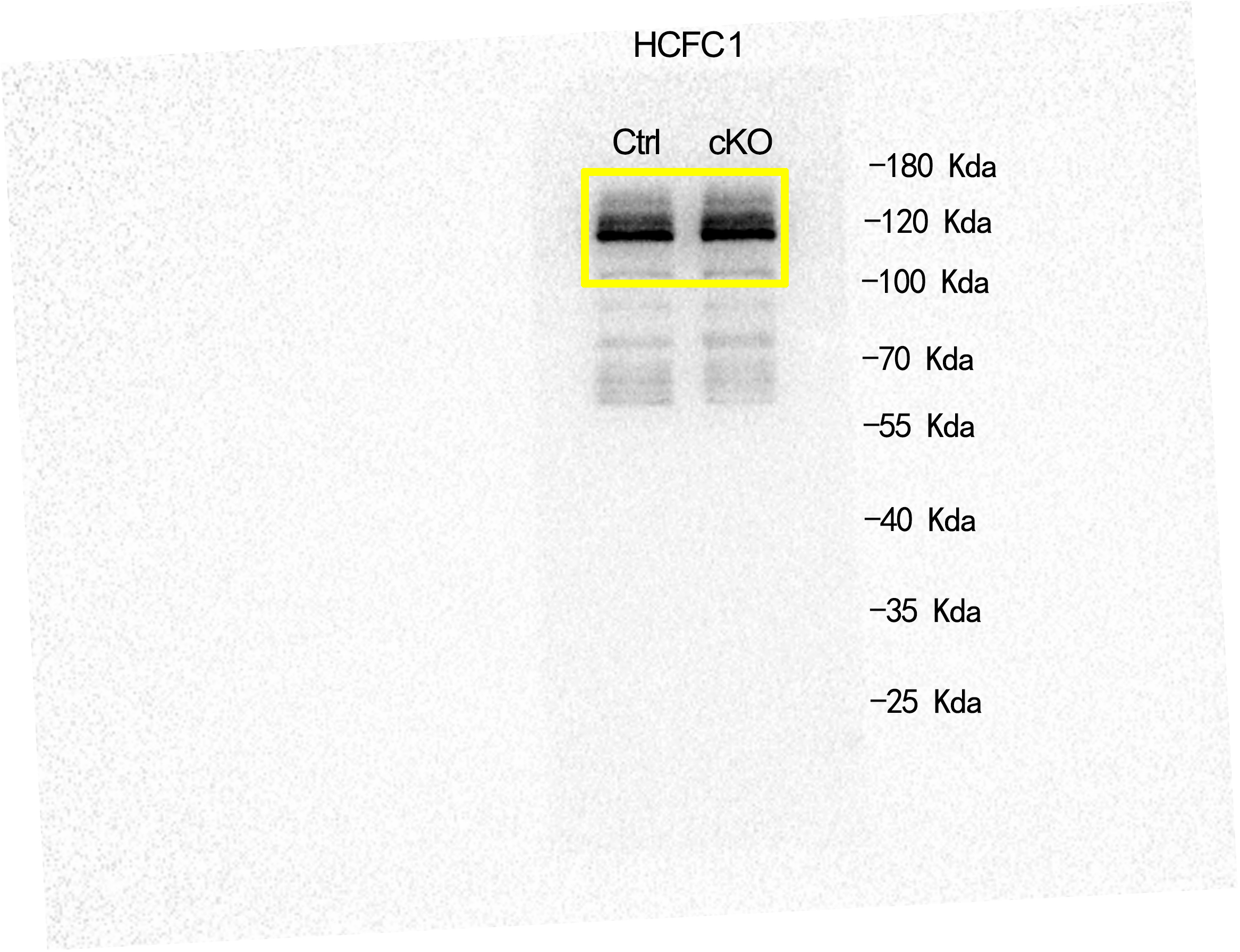

Supplement: Supplementary file 14 — EV and Appendix Figure Source Data [file 44318_2024_203_MOESM14_ESM.zip › Source Data for Expanded View and Appendix/Appendix Figure S3/S3B/WB-HCFC1.jpg]

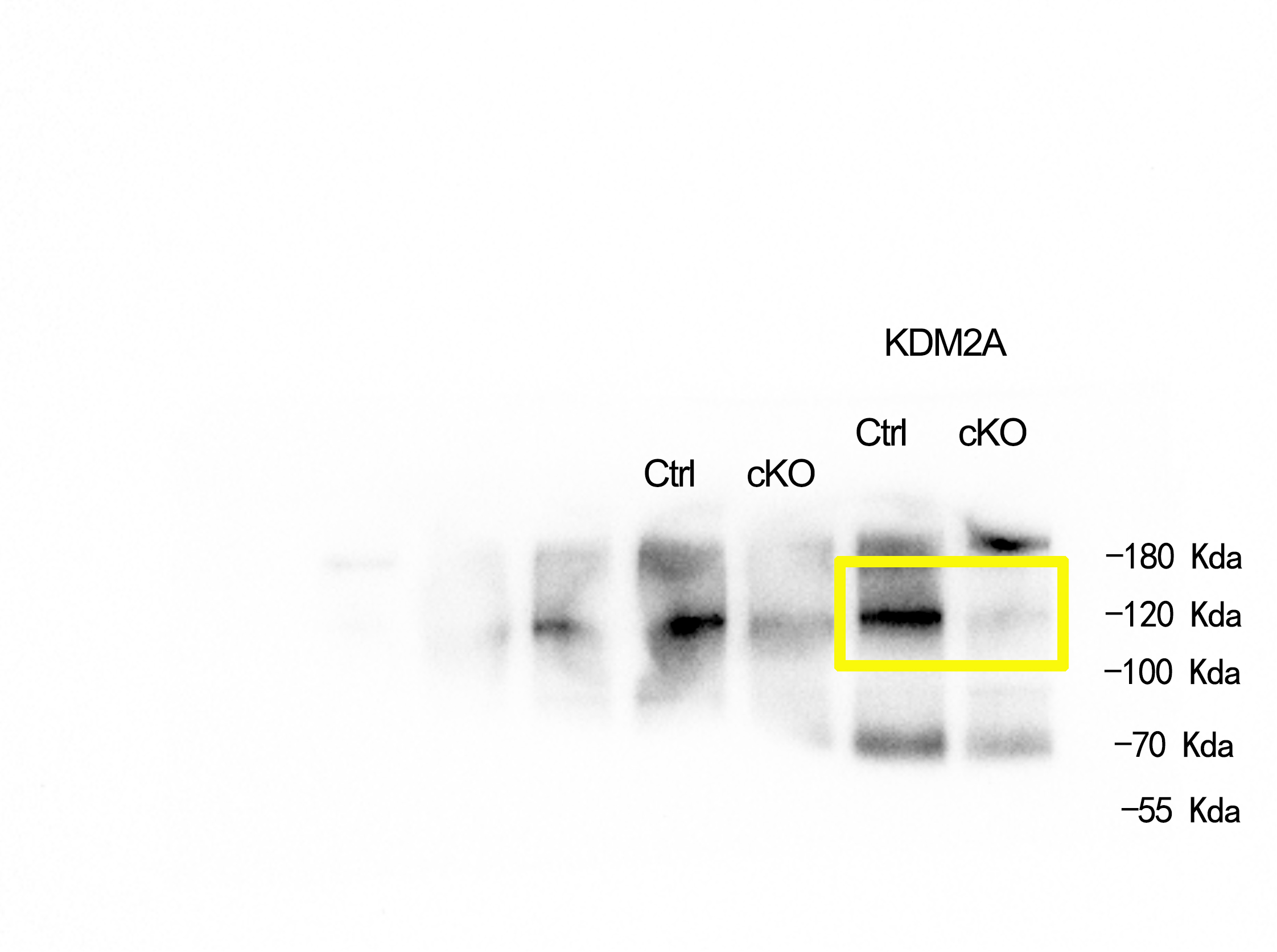

Supplement: Supplementary file 14 — EV and Appendix Figure Source Data [file 44318_2024_203_MOESM14_ESM.zip › Source Data for Expanded View and Appendix/Appendix Figure S3/S3B/WB-KDM2A.jpg]

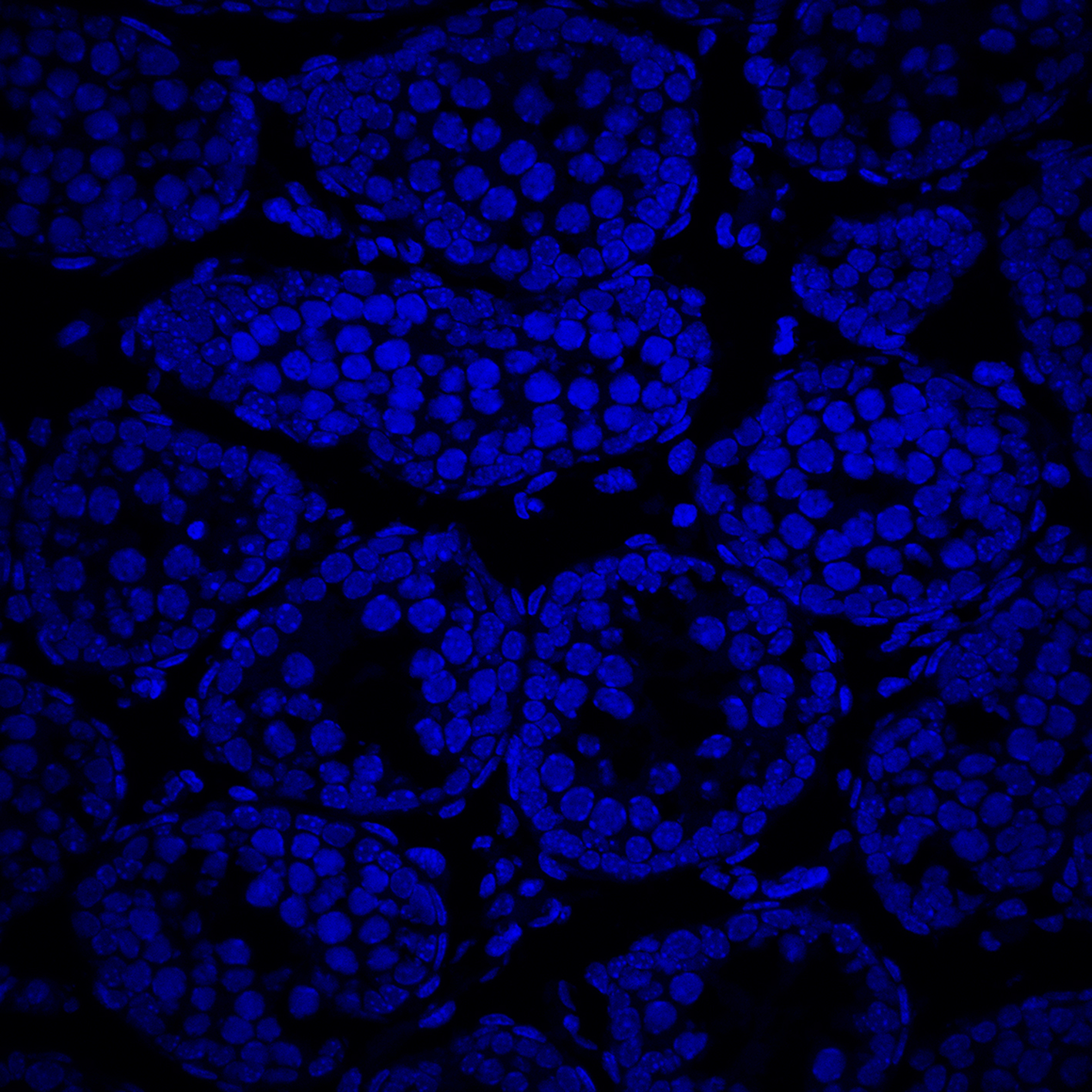

Supplement: Supplementary file 14 — EV and Appendix Figure Source Data [file 44318_2024_203_MOESM14_ESM.zip › Source Data for Expanded View and Appendix/Appendix Figure S2/S2A/Ctrl-DAPI.jpg]

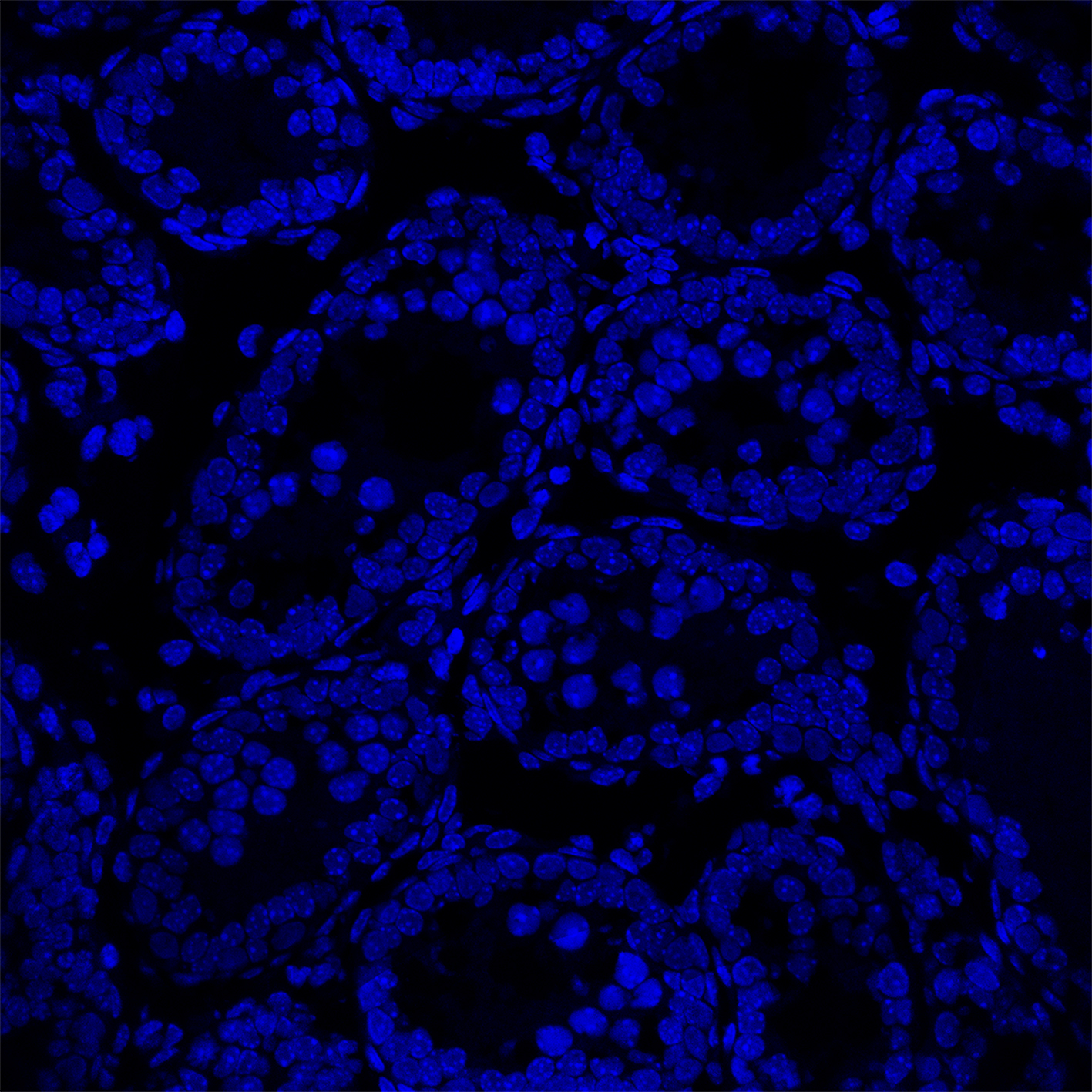

Supplement: Supplementary file 14 — EV and Appendix Figure Source Data [file 44318_2024_203_MOESM14_ESM.zip › Source Data for Expanded View and Appendix/Appendix Figure S2/S2A/cKO-DAPI.jpg]

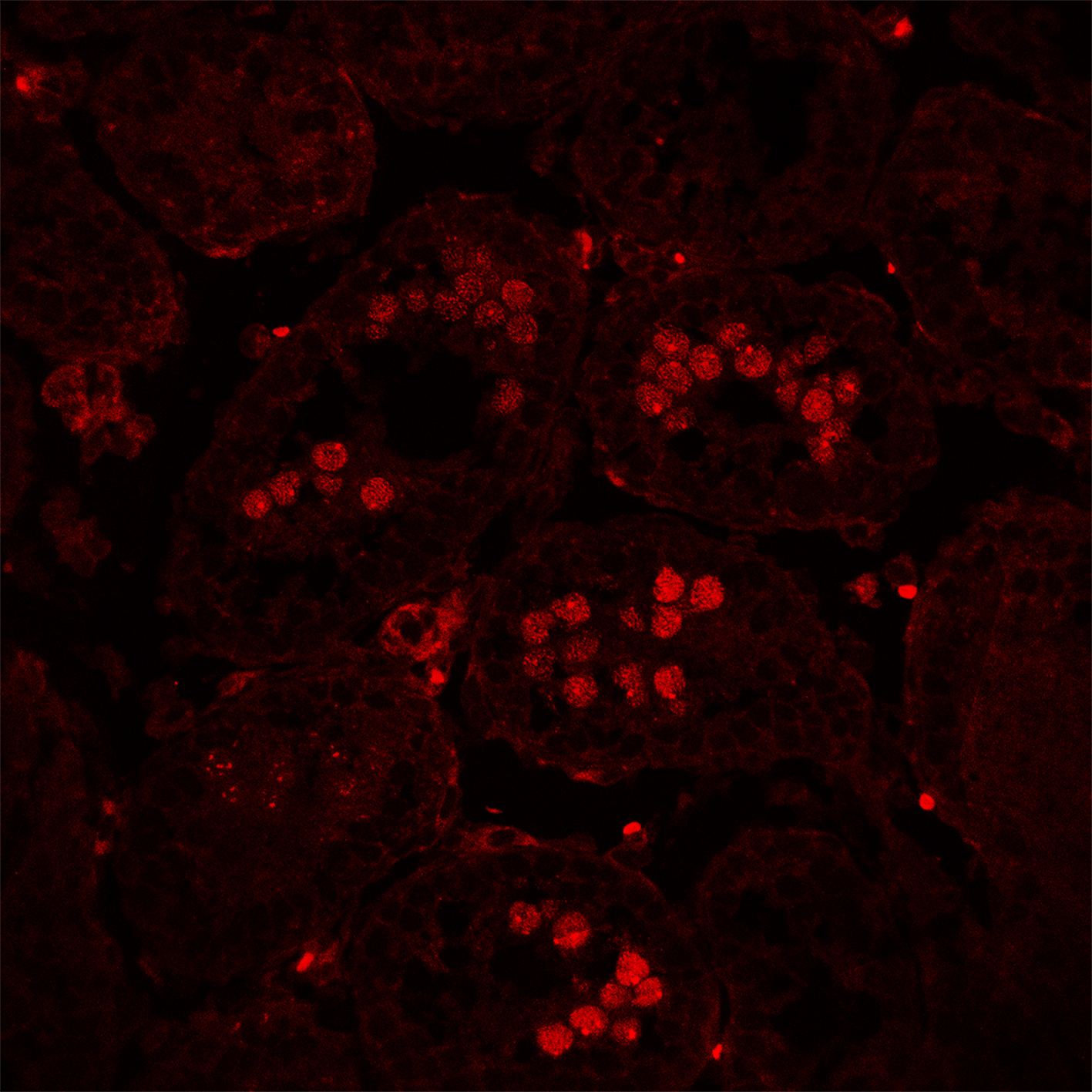

Supplement: Supplementary file 14 — EV and Appendix Figure Source Data [file 44318_2024_203_MOESM14_ESM.zip › Source Data for Expanded View and Appendix/Appendix Figure S2/S2A/cKO-SYCP3.jpg]

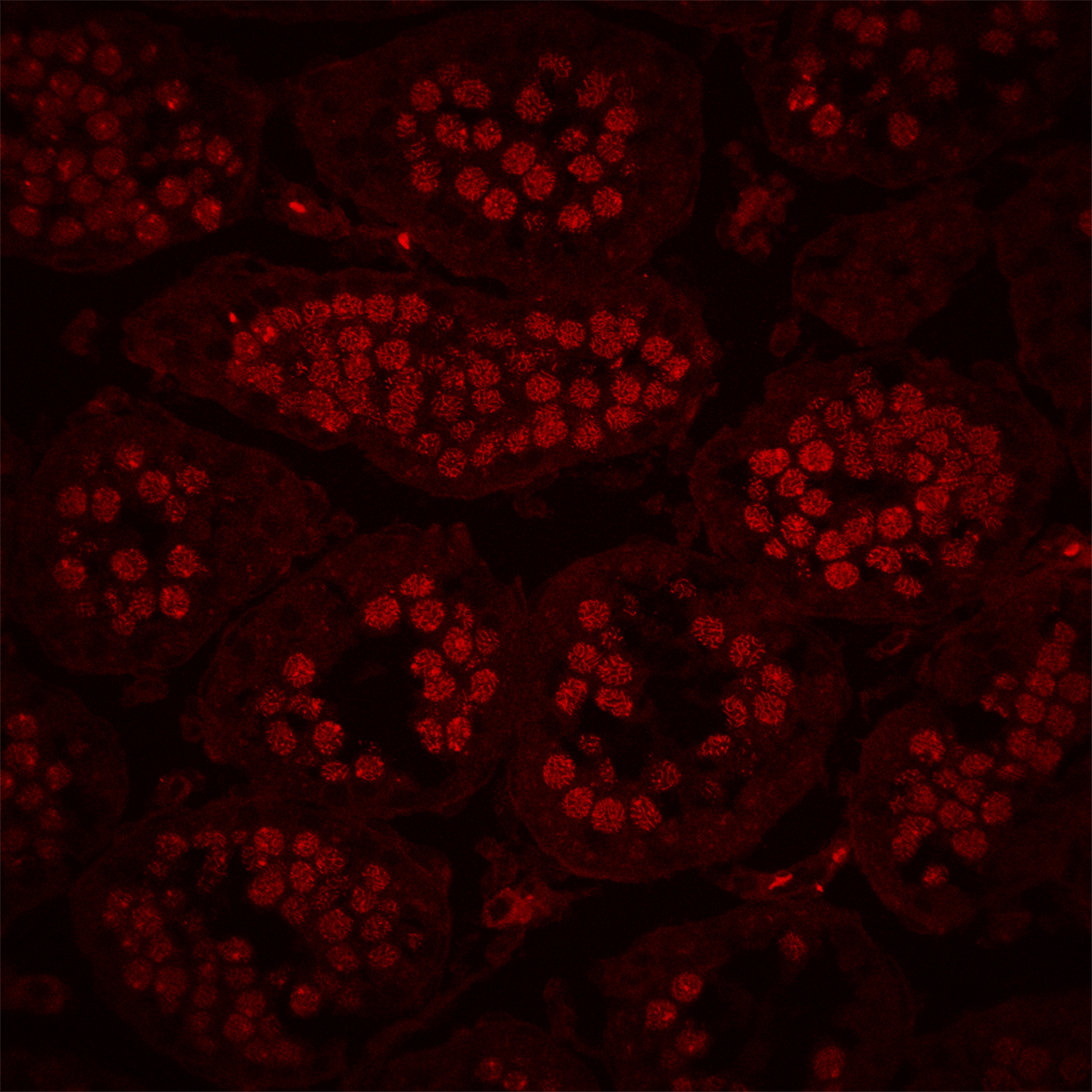

Supplement: Supplementary file 14 — EV and Appendix Figure Source Data [file 44318_2024_203_MOESM14_ESM.zip › Source Data for Expanded View and Appendix/Appendix Figure S2/S2A/Ctrl-SYCP3.jpg]

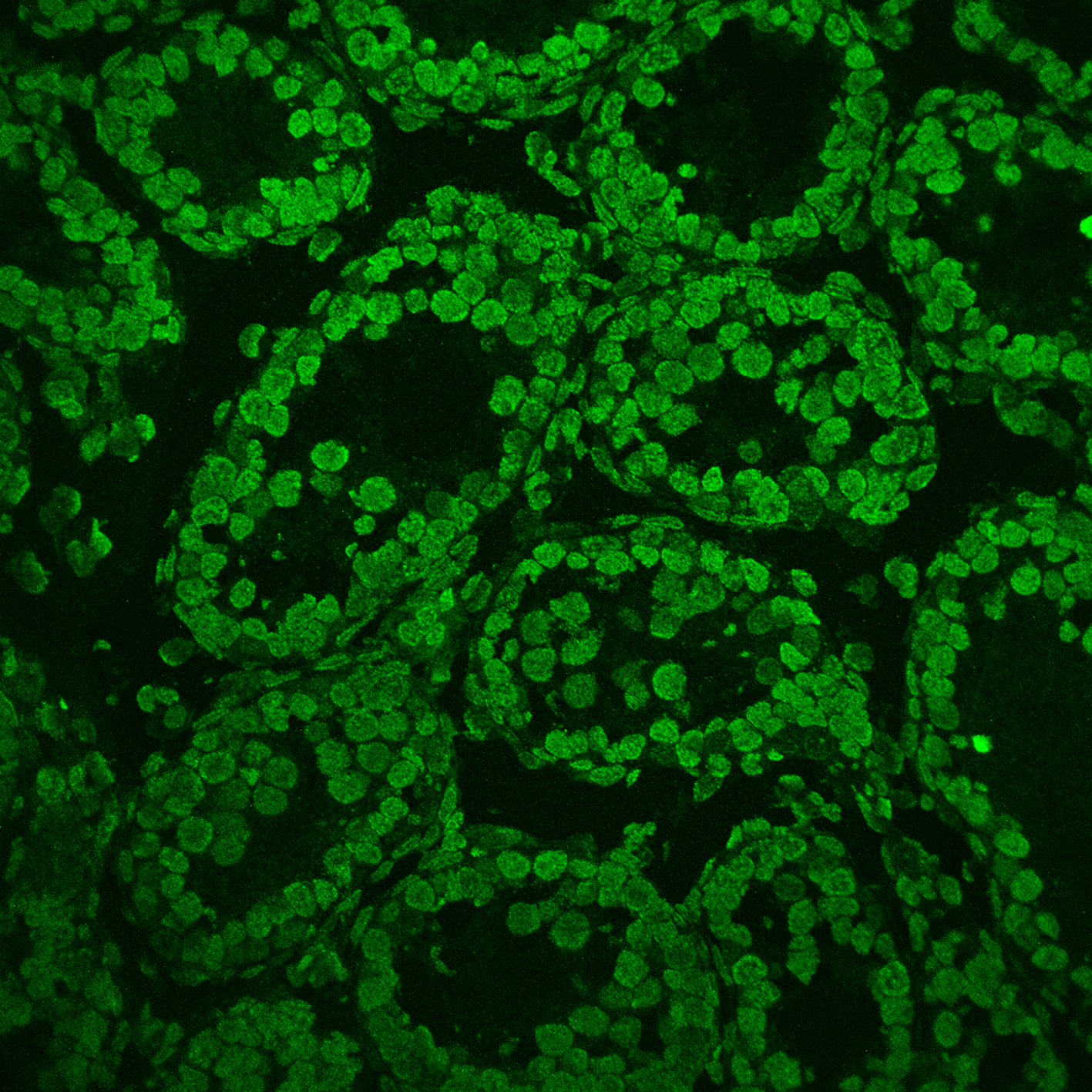

Supplement: Supplementary file 14 — EV and Appendix Figure Source Data [file 44318_2024_203_MOESM14_ESM.zip › Source Data for Expanded View and Appendix/Appendix Figure S2/S2A/cKO-H3K36me1.jpg]

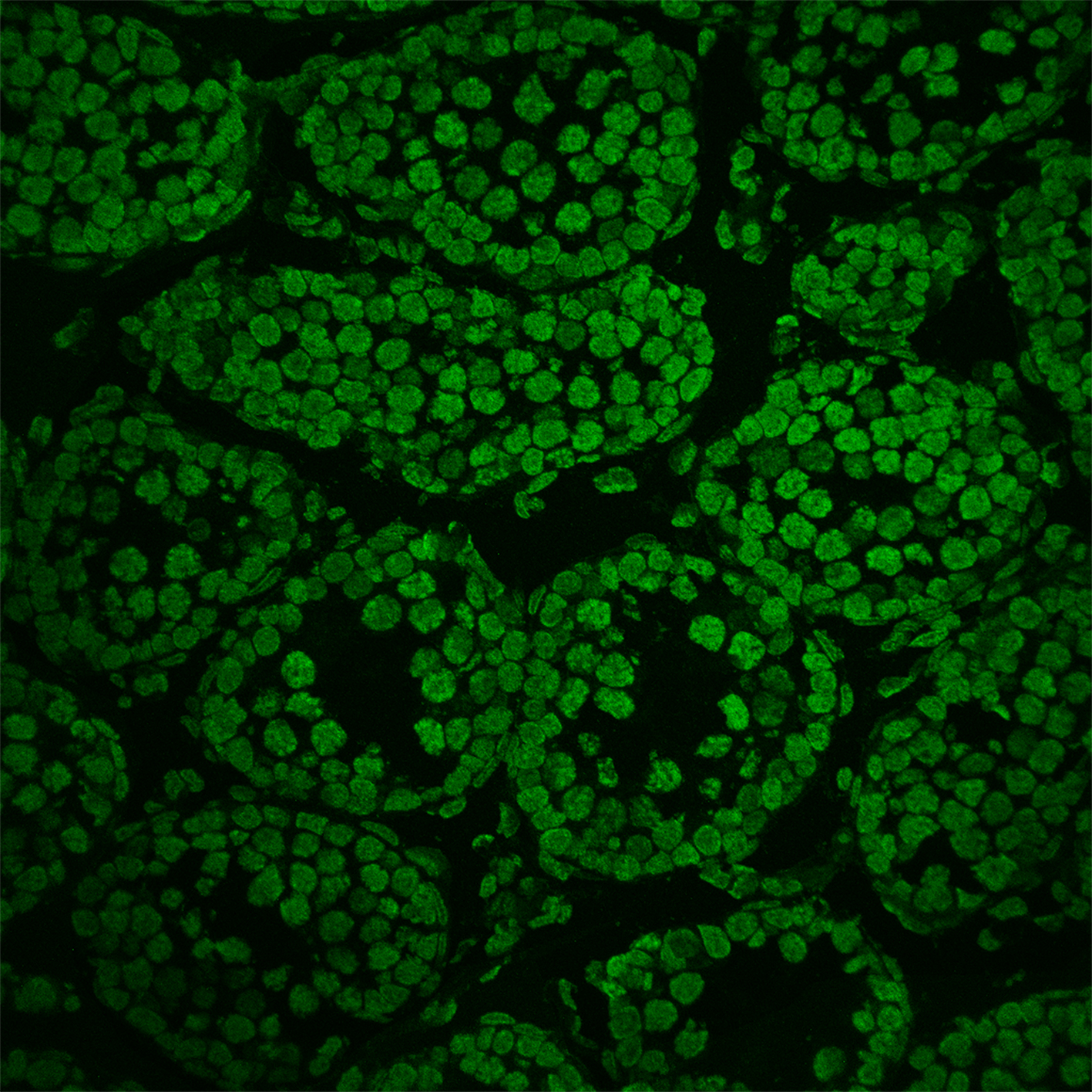

Supplement: Supplementary file 14 — EV and Appendix Figure Source Data [file 44318_2024_203_MOESM14_ESM.zip › Source Data for Expanded View and Appendix/Appendix Figure S2/S2A/Ctrl-H3K36me1.jpg]

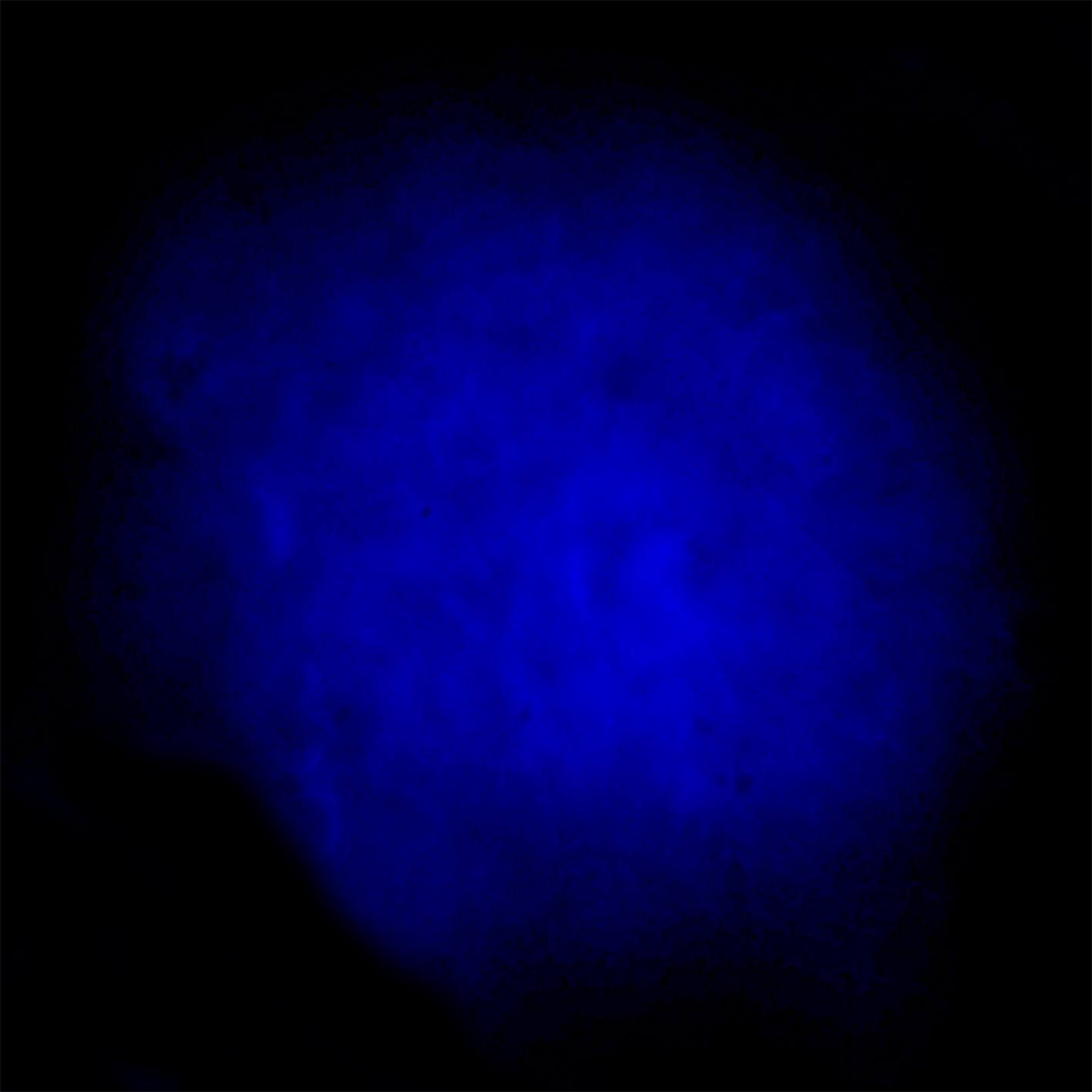

Supplement: Supplementary file 14 — EV and Appendix Figure Source Data [file 44318_2024_203_MOESM14_ESM.zip › Source Data for Expanded View and Appendix/Appendix Figure S2/S2E/cKO-Zyg-ii-DAPI.jpg]

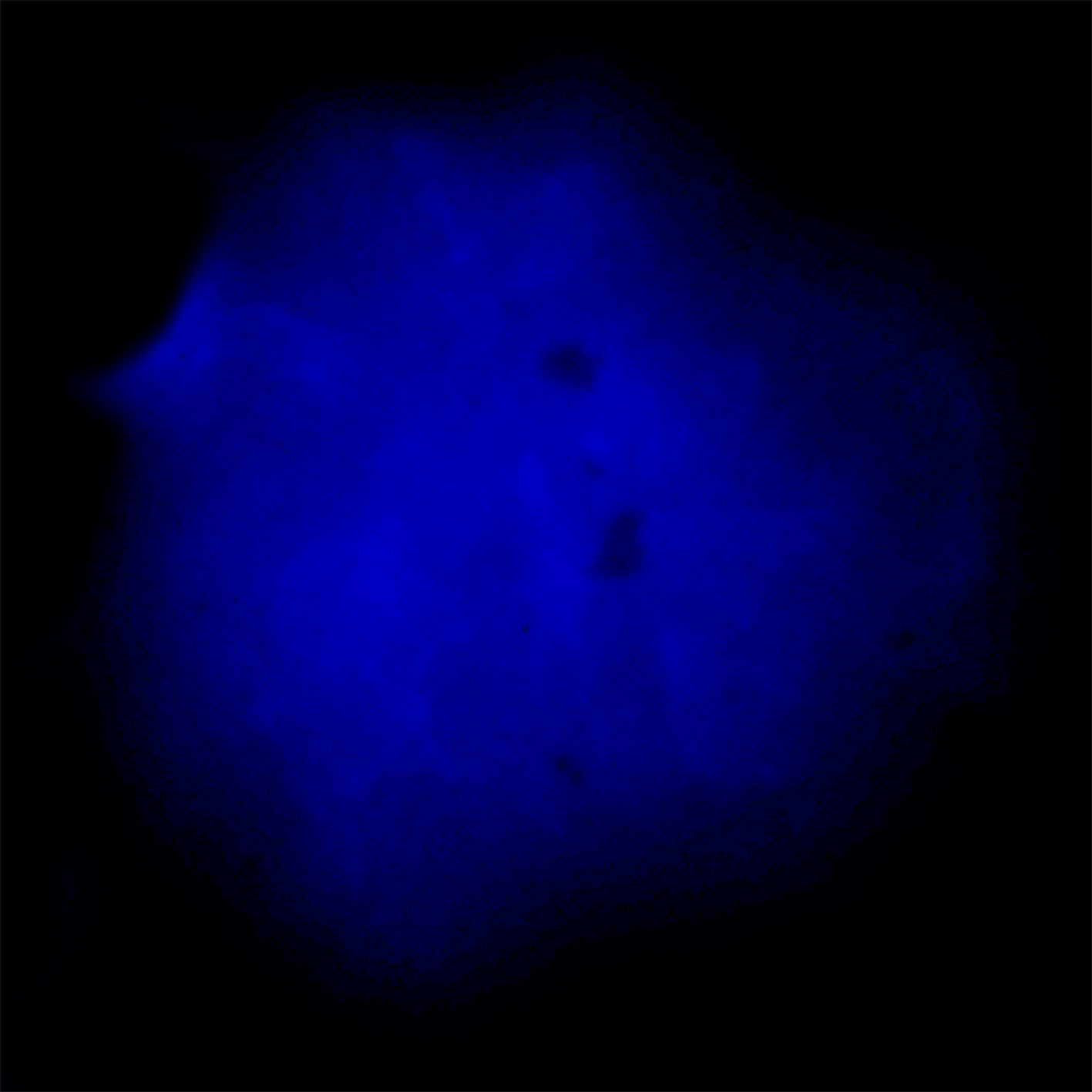

Supplement: Supplementary file 14 — EV and Appendix Figure Source Data [file 44318_2024_203_MOESM14_ESM.zip › Source Data for Expanded View and Appendix/Appendix Figure S2/S2E/cKO-Zyg-i-DAPI.jpg]

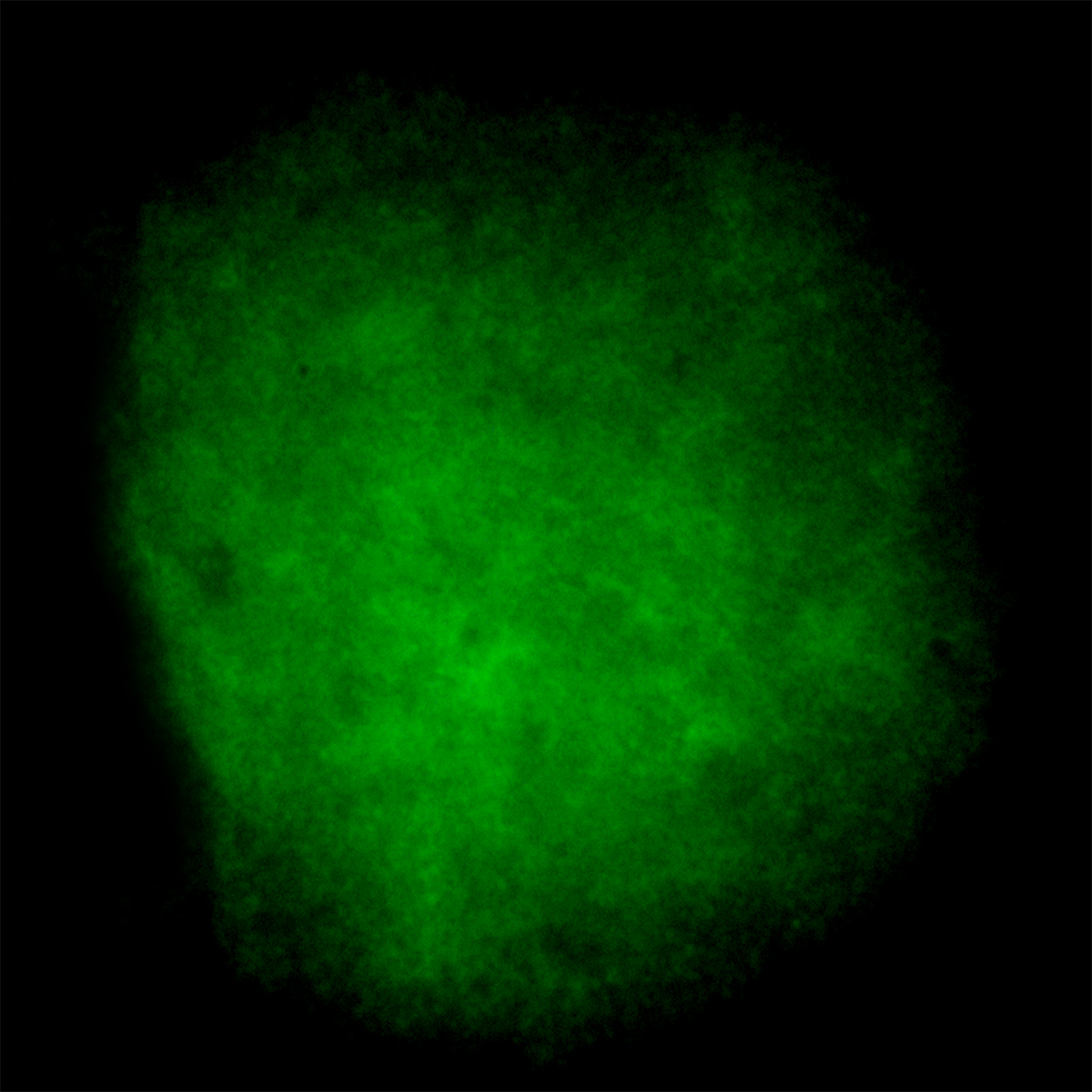

Supplement: Supplementary file 14 — EV and Appendix Figure Source Data [file 44318_2024_203_MOESM14_ESM.zip › Source Data for Expanded View and Appendix/Appendix Figure S2/S2E/cKO-Lep-H3K36me3.jpg]

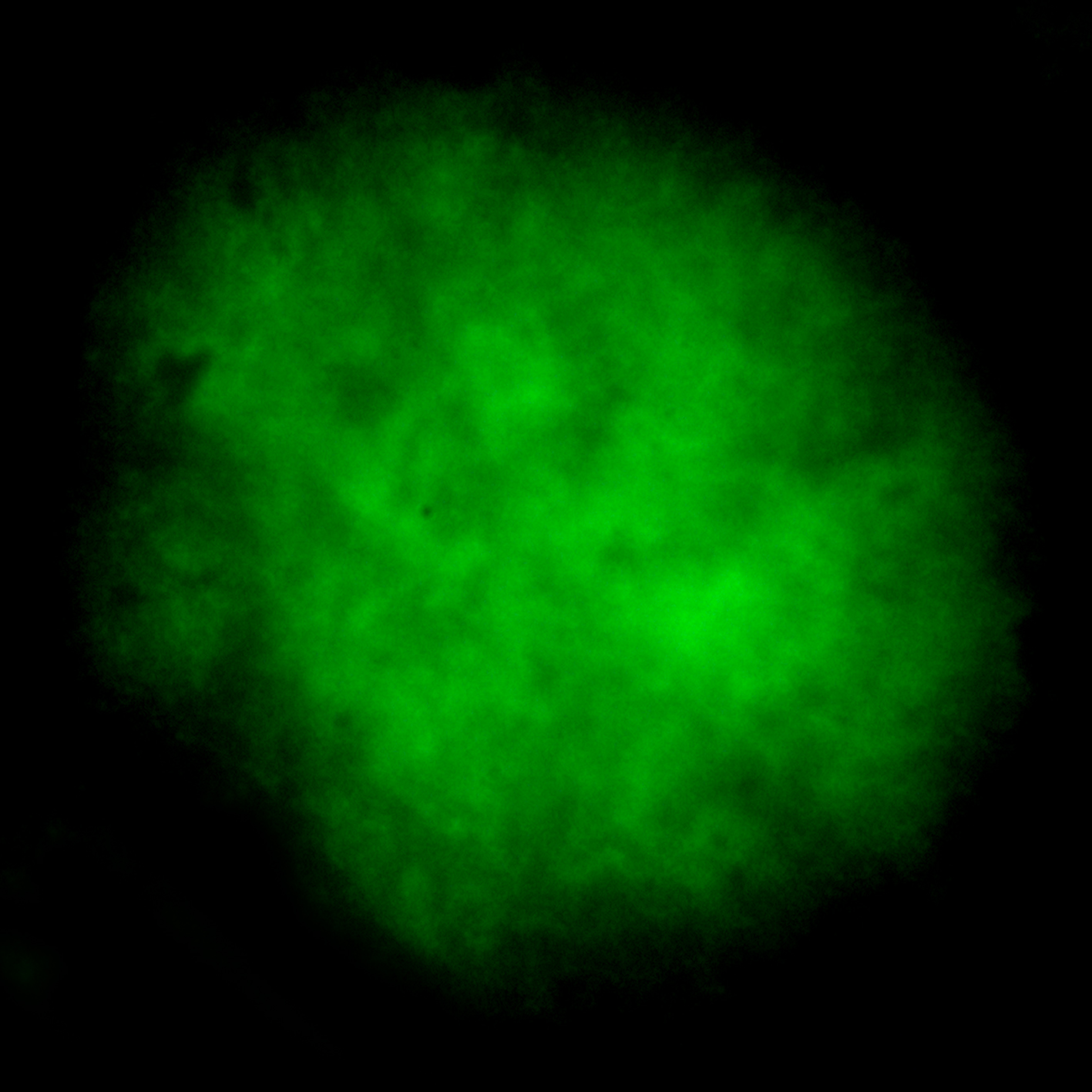

Supplement: Supplementary file 14 — EV and Appendix Figure Source Data [file 44318_2024_203_MOESM14_ESM.zip › Source Data for Expanded View and Appendix/Appendix Figure S2/S2E/cKO-Zyg-ii-H3K36me3.jpg]

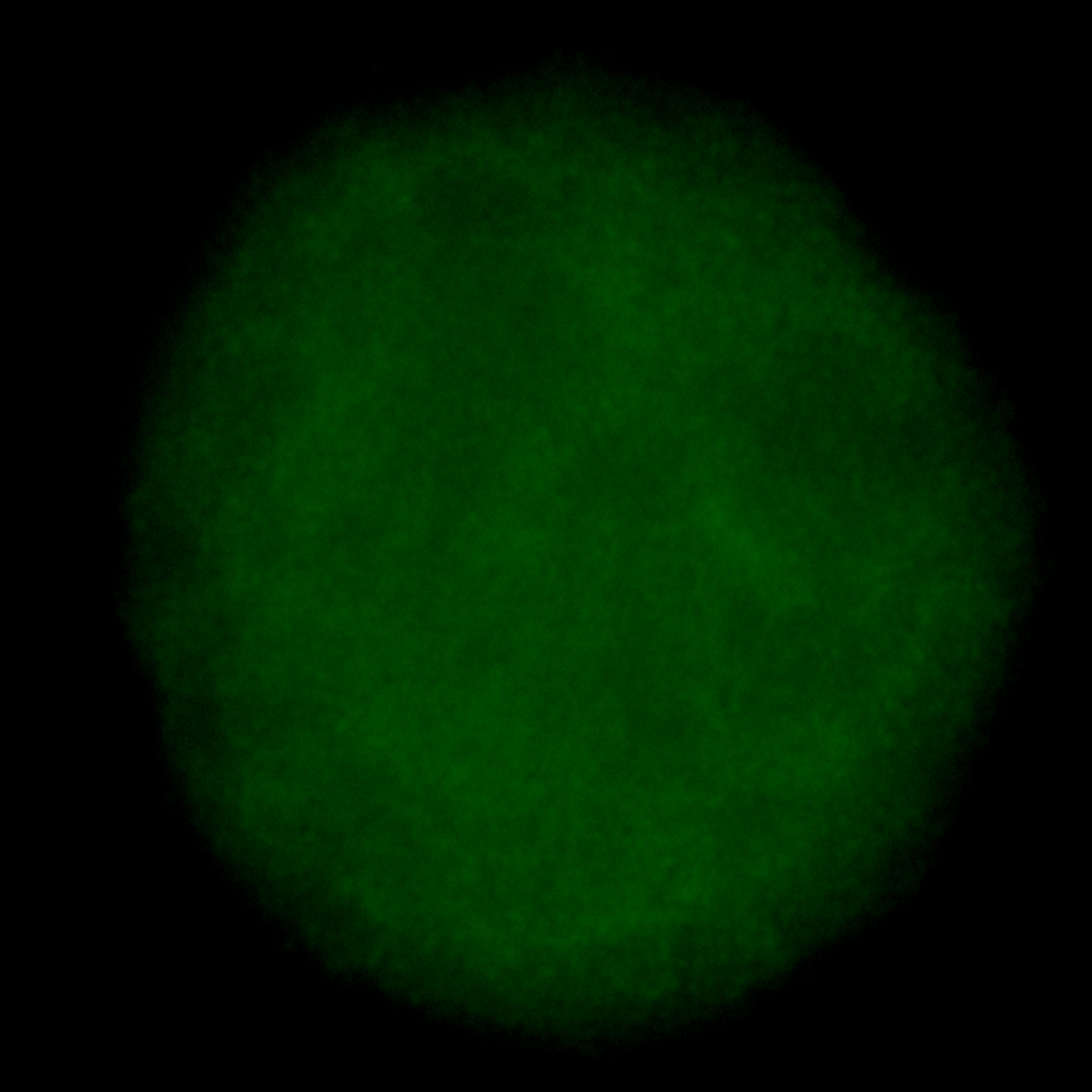

Supplement: Supplementary file 14 — EV and Appendix Figure Source Data [file 44318_2024_203_MOESM14_ESM.zip › Source Data for Expanded View and Appendix/Appendix Figure S2/S2E/Ctrl-Zyg-H3K36me3.jpg]

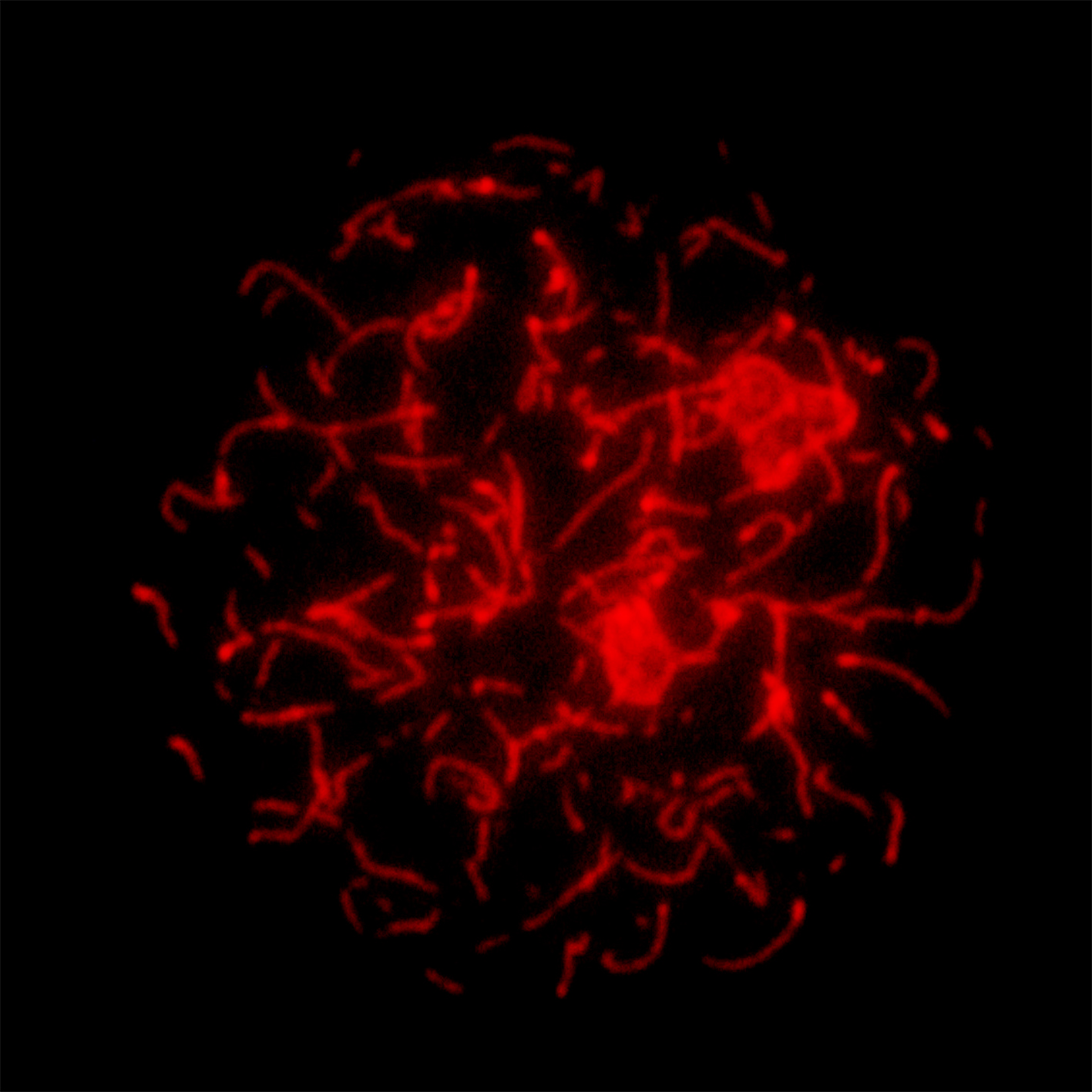

Supplement: Supplementary file 14 — EV and Appendix Figure Source Data [file 44318_2024_203_MOESM14_ESM.zip › Source Data for Expanded View and Appendix/Appendix Figure S2/S2E/Ctrl-Zyg-SYCP3.jpg]

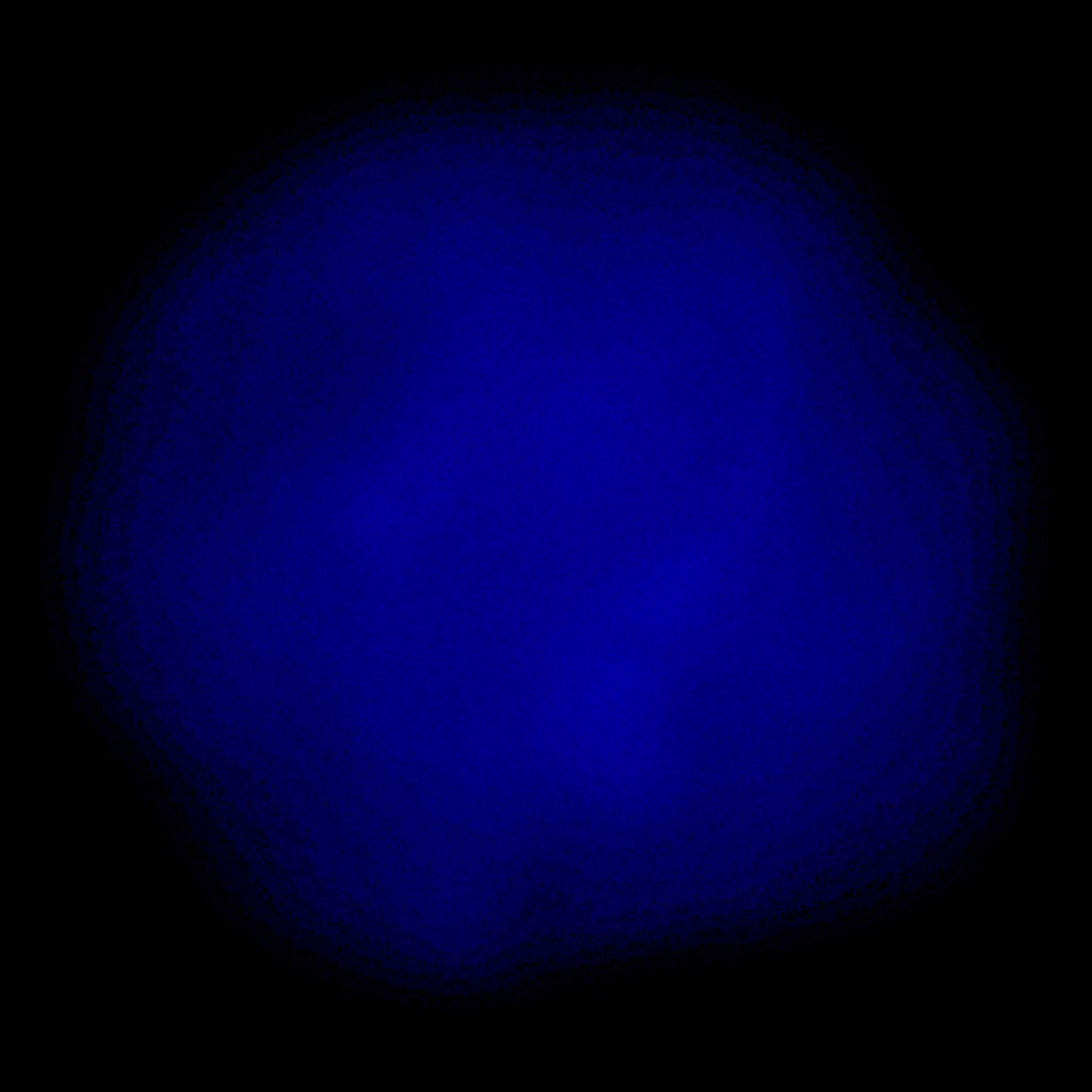

Supplement: Supplementary file 14 — EV and Appendix Figure Source Data [file 44318_2024_203_MOESM14_ESM.zip › Source Data for Expanded View and Appendix/Appendix Figure S2/S2E/Ctrl-Pac-DAPI.jpg]

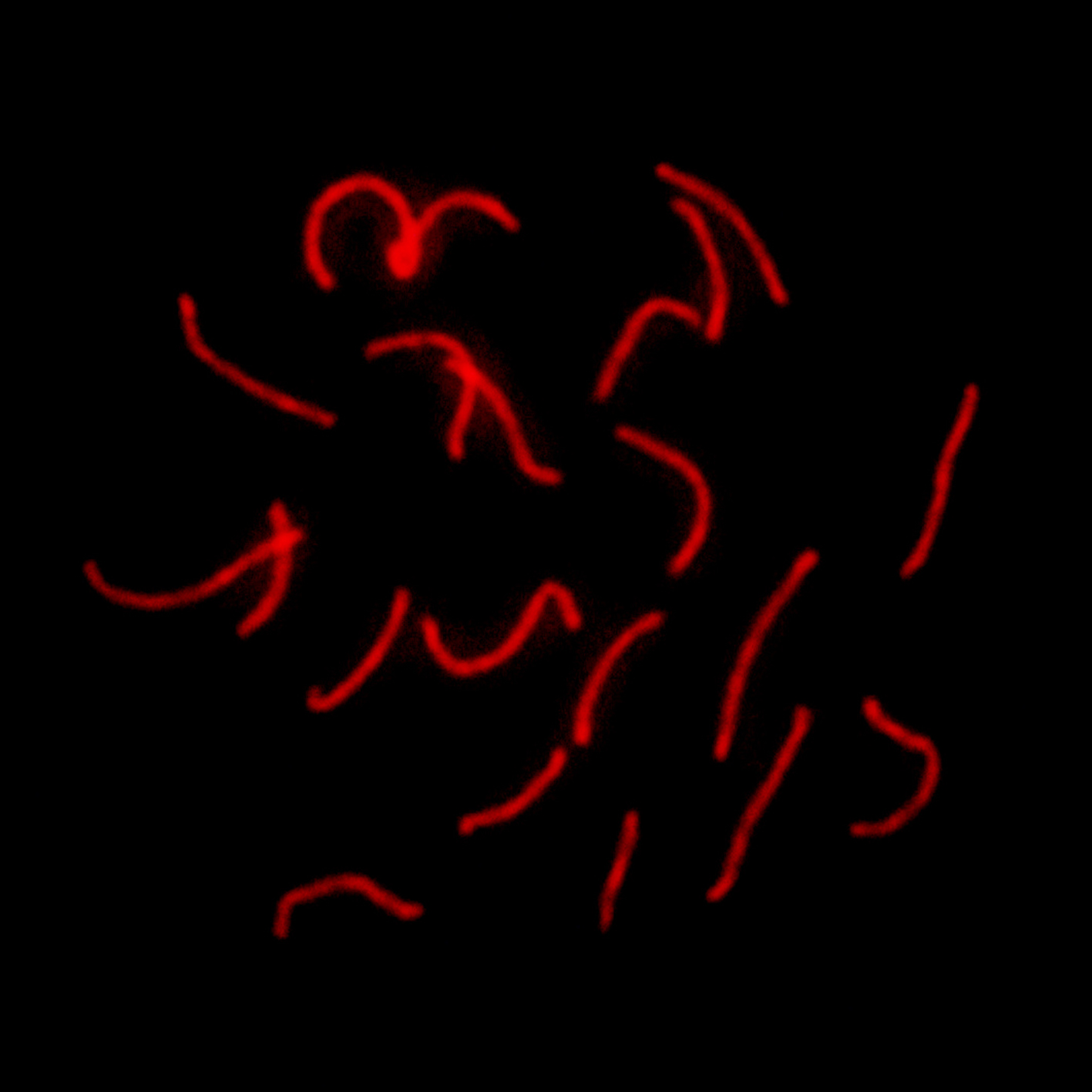

Supplement: Supplementary file 14 — EV and Appendix Figure Source Data [file 44318_2024_203_MOESM14_ESM.zip › Source Data for Expanded View and Appendix/Appendix Figure S2/S2E/Ctrl-Pac-SYCP3.jpg]

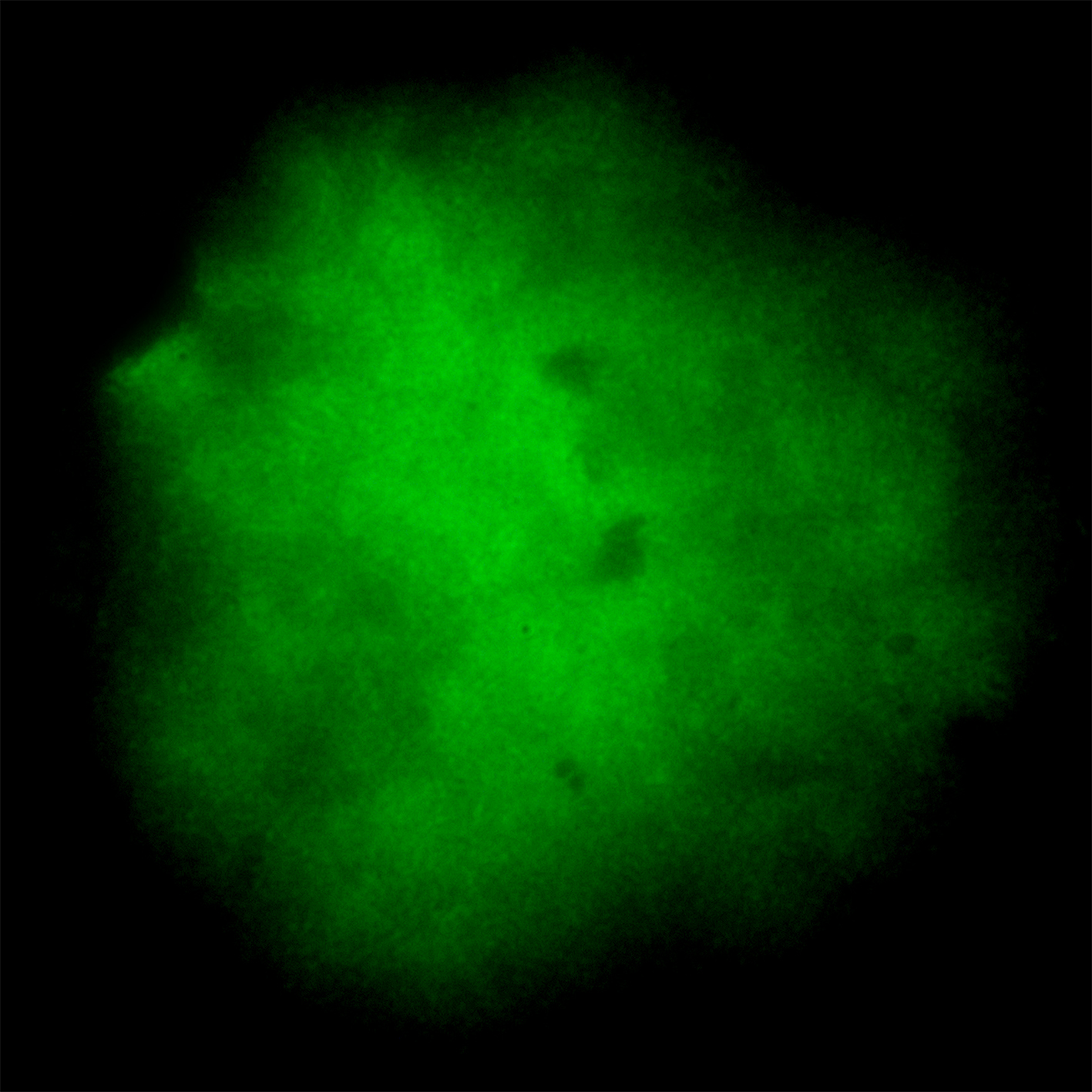

Supplement: Supplementary file 14 — EV and Appendix Figure Source Data [file 44318_2024_203_MOESM14_ESM.zip › Source Data for Expanded View and Appendix/Appendix Figure S2/S2E/cKO-Zyg-i-H3K36me3.jpg]

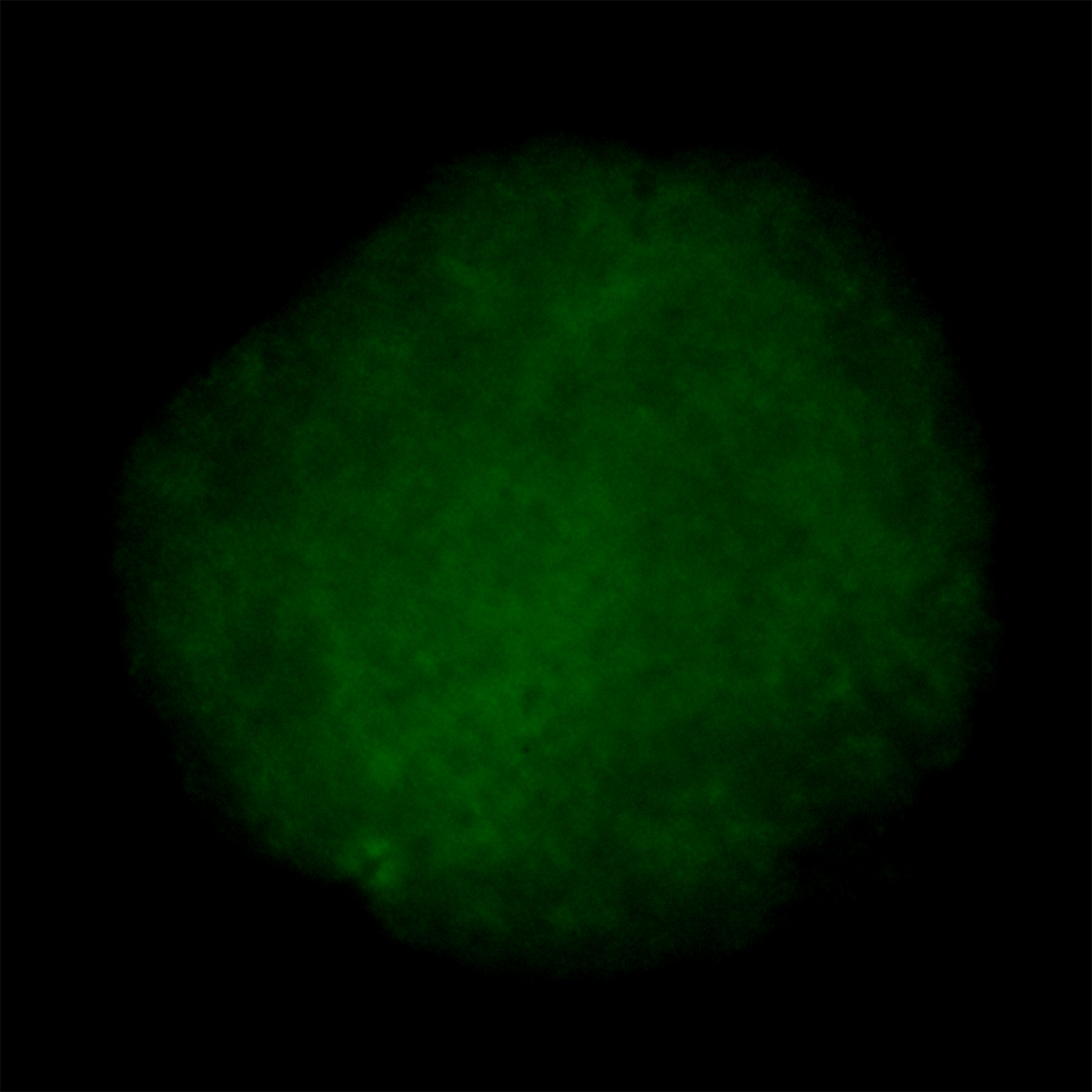

Supplement: Supplementary file 14 — EV and Appendix Figure Source Data [file 44318_2024_203_MOESM14_ESM.zip › Source Data for Expanded View and Appendix/Appendix Figure S2/S2E/Ctrl-Lep-H3K36me3.jpg]

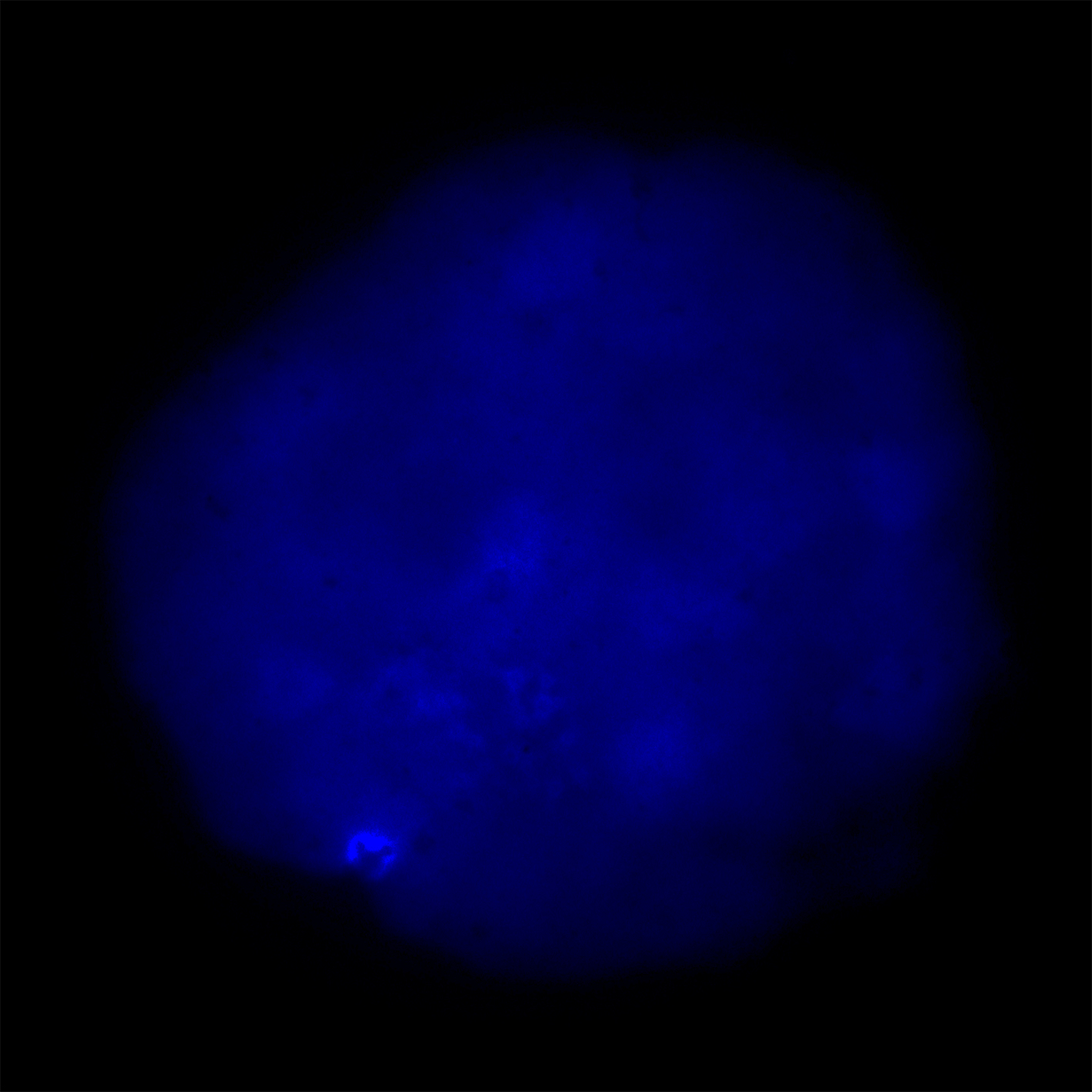

Supplement: Supplementary file 14 — EV and Appendix Figure Source Data [file 44318_2024_203_MOESM14_ESM.zip › Source Data for Expanded View and Appendix/Appendix Figure S2/S2E/Ctrl-Lep-DAPI.jpg]

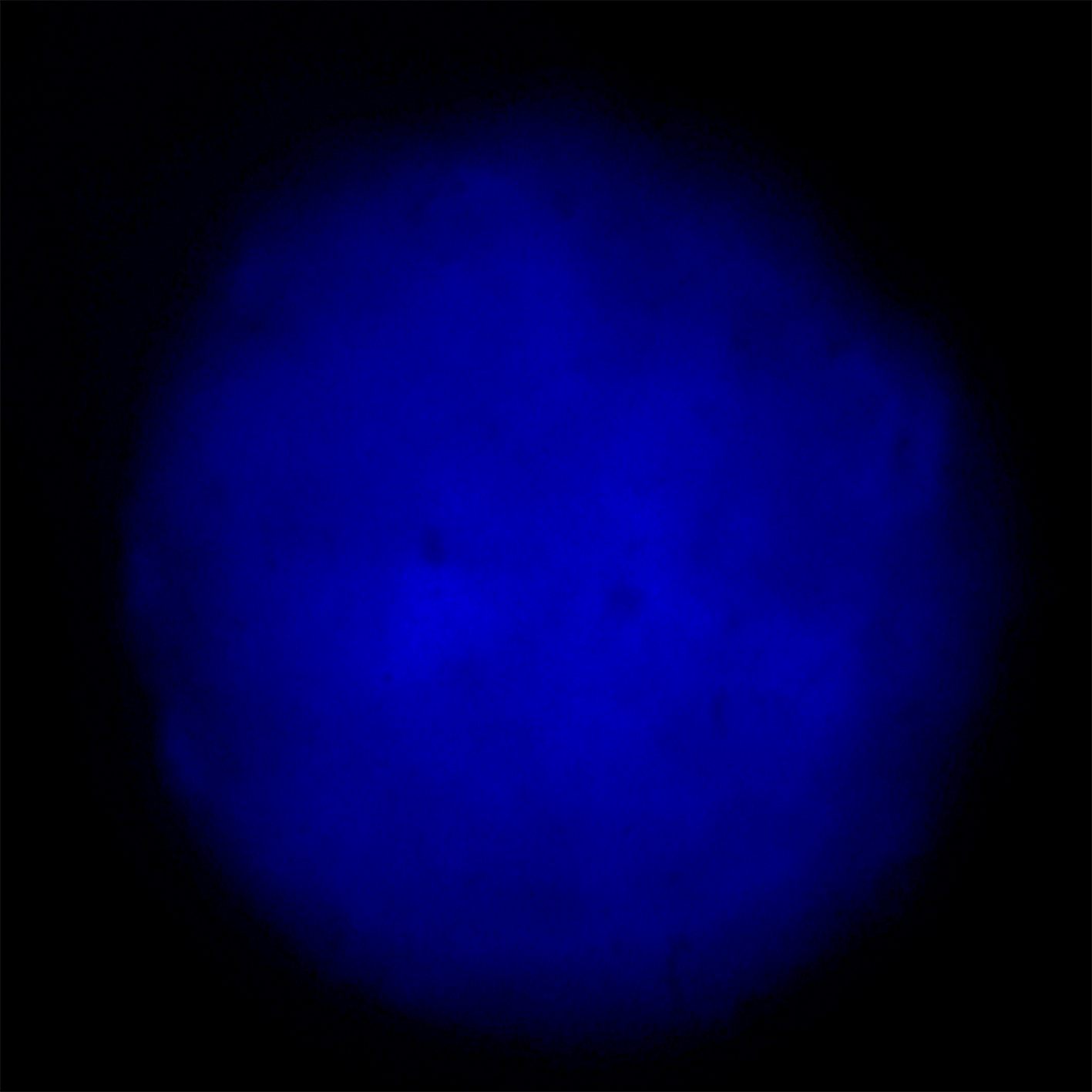

Supplement: Supplementary file 14 — EV and Appendix Figure Source Data [file 44318_2024_203_MOESM14_ESM.zip › Source Data for Expanded View and Appendix/Appendix Figure S2/S2E/Ctrl-Zyg-DAPI.jpg]

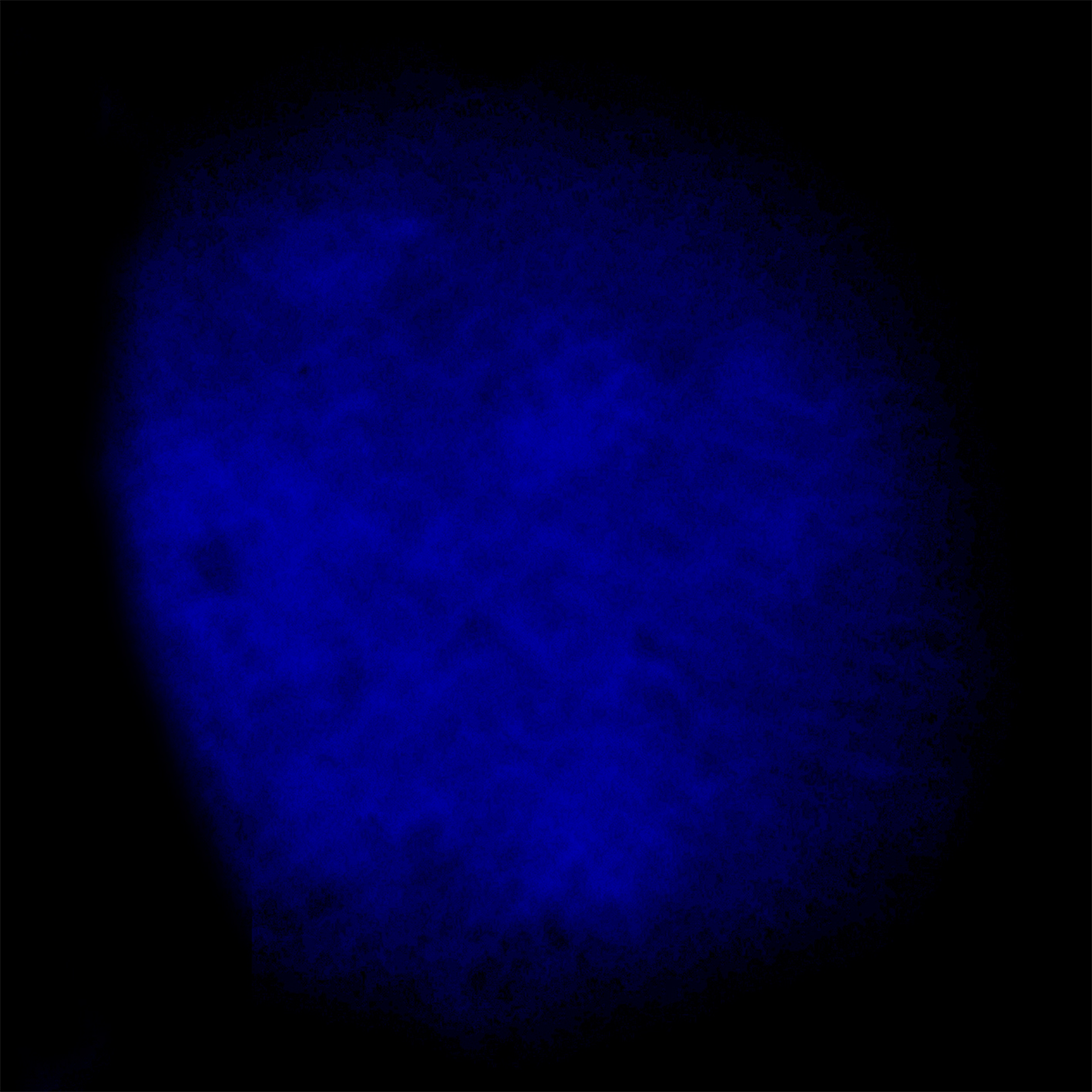

Supplement: Supplementary file 14 — EV and Appendix Figure Source Data [file 44318_2024_203_MOESM14_ESM.zip › Source Data for Expanded View and Appendix/Appendix Figure S2/S2E/cKO-Lep-DAPI.jpg]

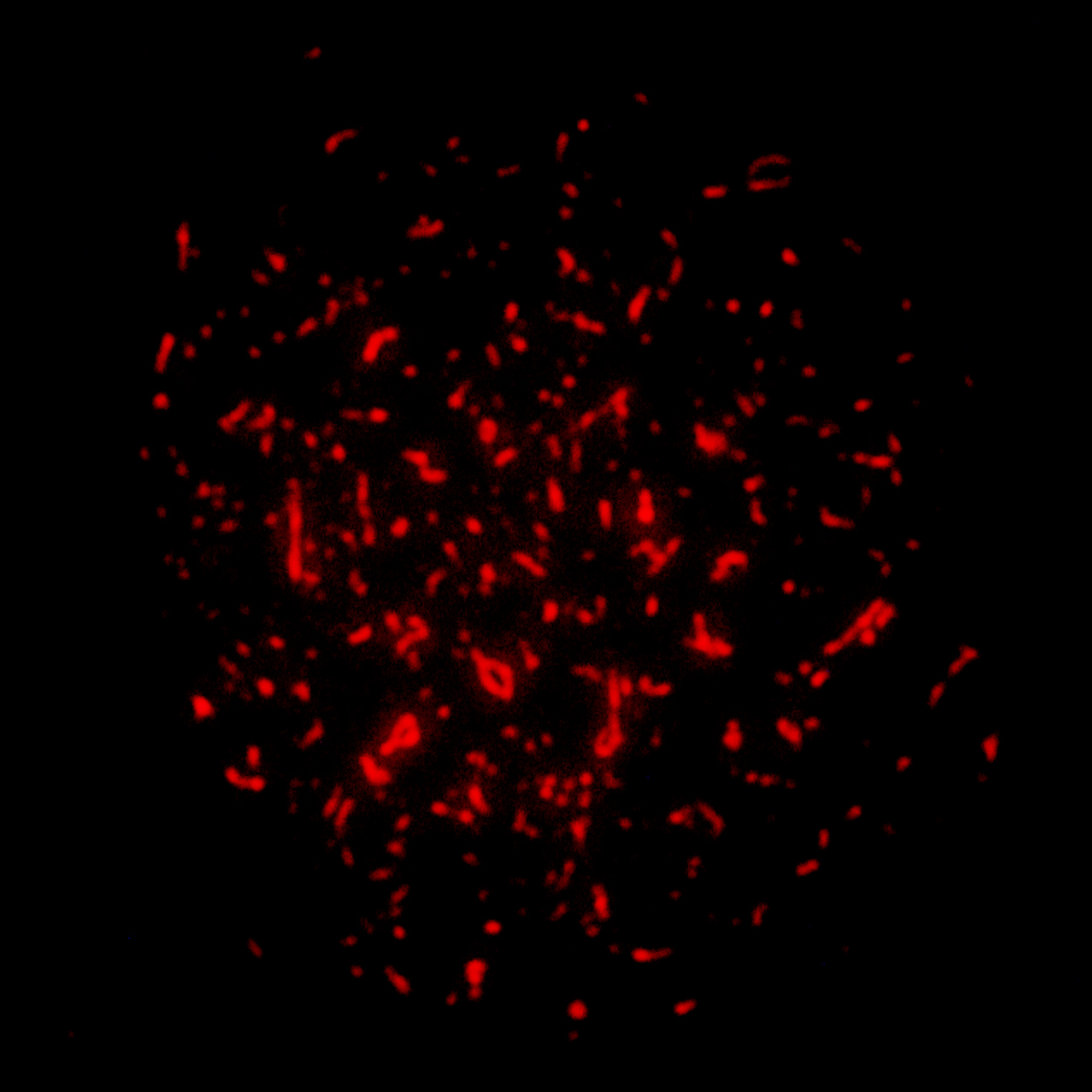

Supplement: Supplementary file 14 — EV and Appendix Figure Source Data [file 44318_2024_203_MOESM14_ESM.zip › Source Data for Expanded View and Appendix/Appendix Figure S2/S2E/cKO-Lep-SYCP3.jpg]

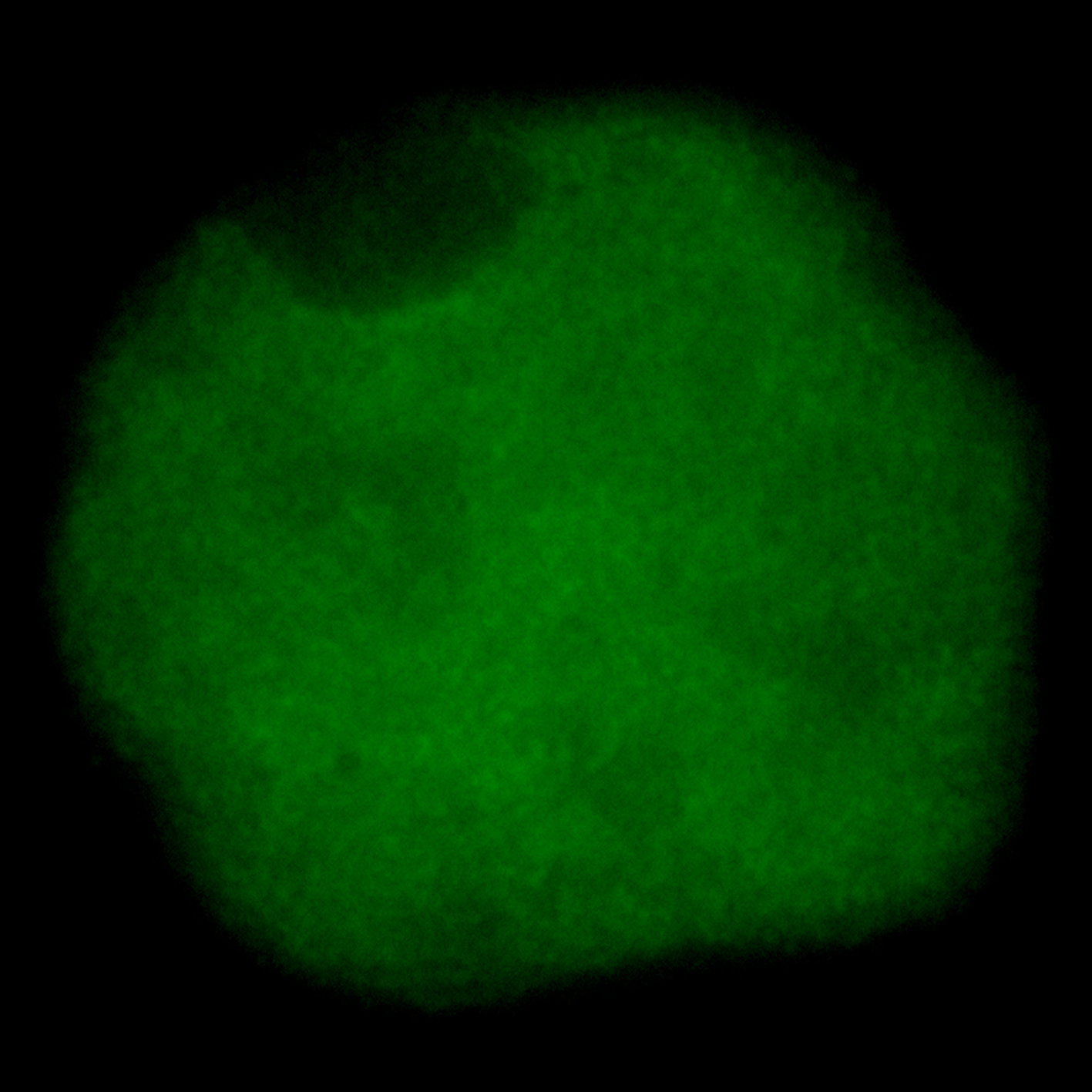

Supplement: Supplementary file 14 — EV and Appendix Figure Source Data [file 44318_2024_203_MOESM14_ESM.zip › Source Data for Expanded View and Appendix/Appendix Figure S2/S2E/Ctrl-Pac-H3K36me3.jpg]

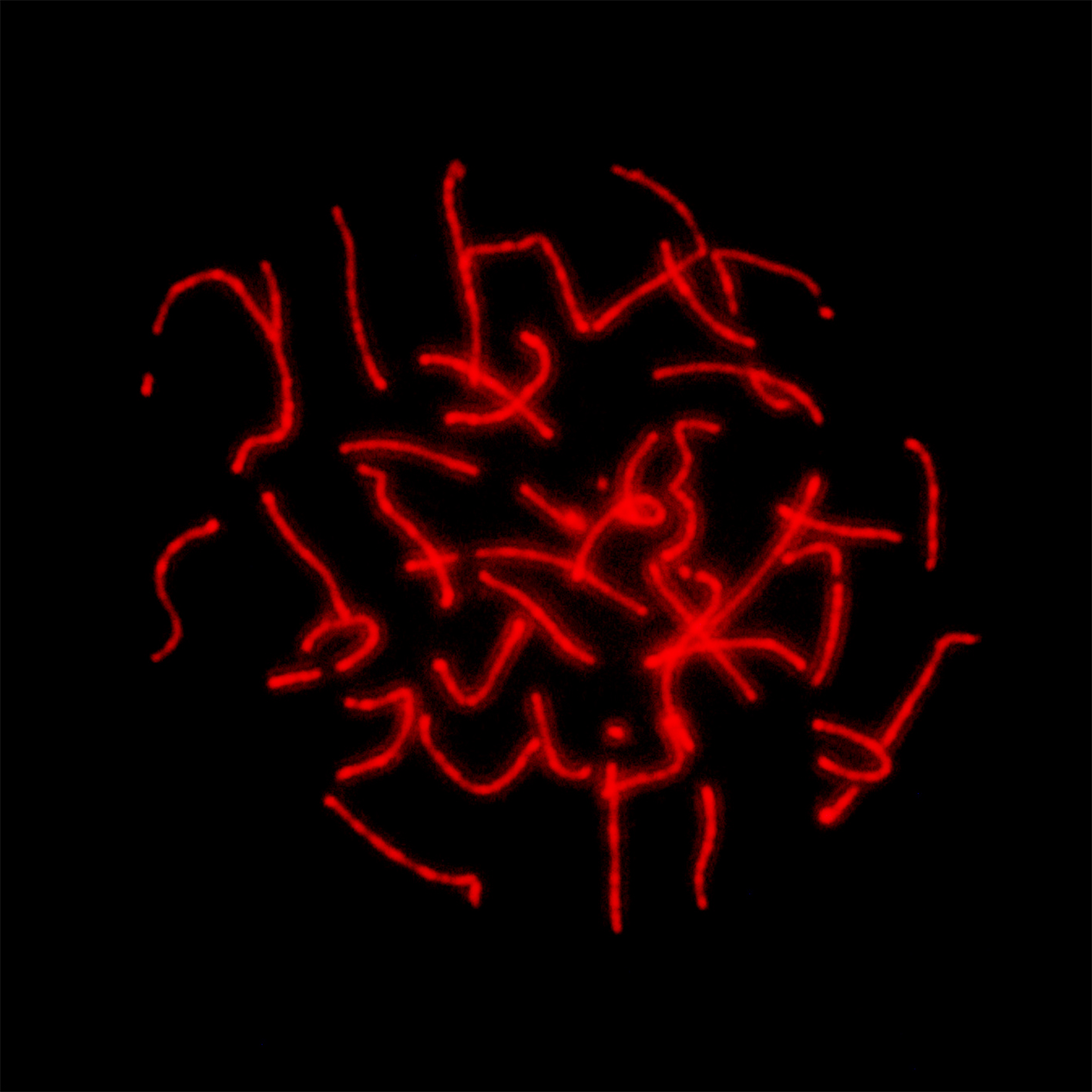

Supplement: Supplementary file 14 — EV and Appendix Figure Source Data [file 44318_2024_203_MOESM14_ESM.zip › Source Data for Expanded View and Appendix/Appendix Figure S2/S2E/cKO-Zyg-ii-SYPC3.jpg]

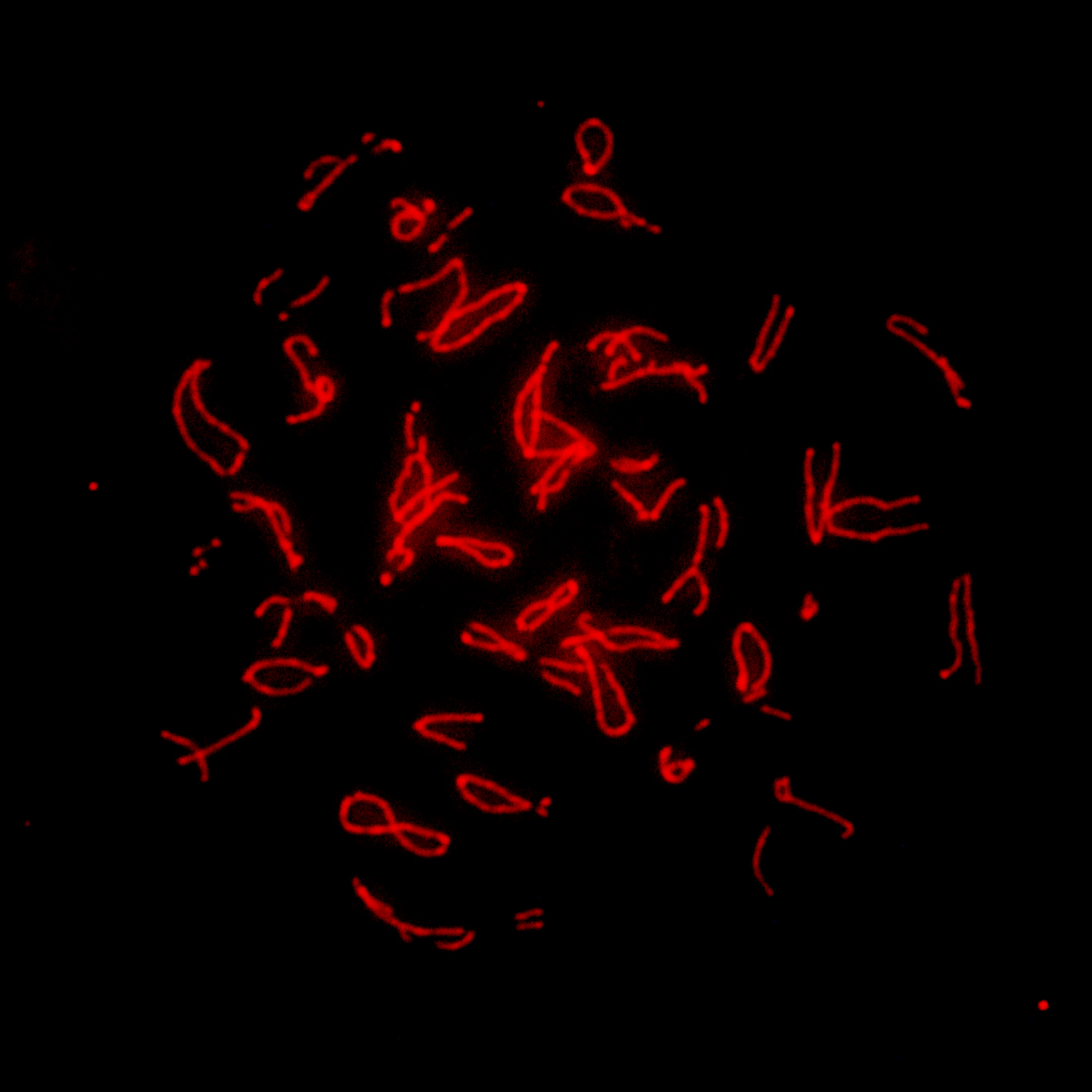

Supplement: Supplementary file 14 — EV and Appendix Figure Source Data [file 44318_2024_203_MOESM14_ESM.zip › Source Data for Expanded View and Appendix/Appendix Figure S2/S2E/cKO-Zyg-i-SYPC3.jpg]

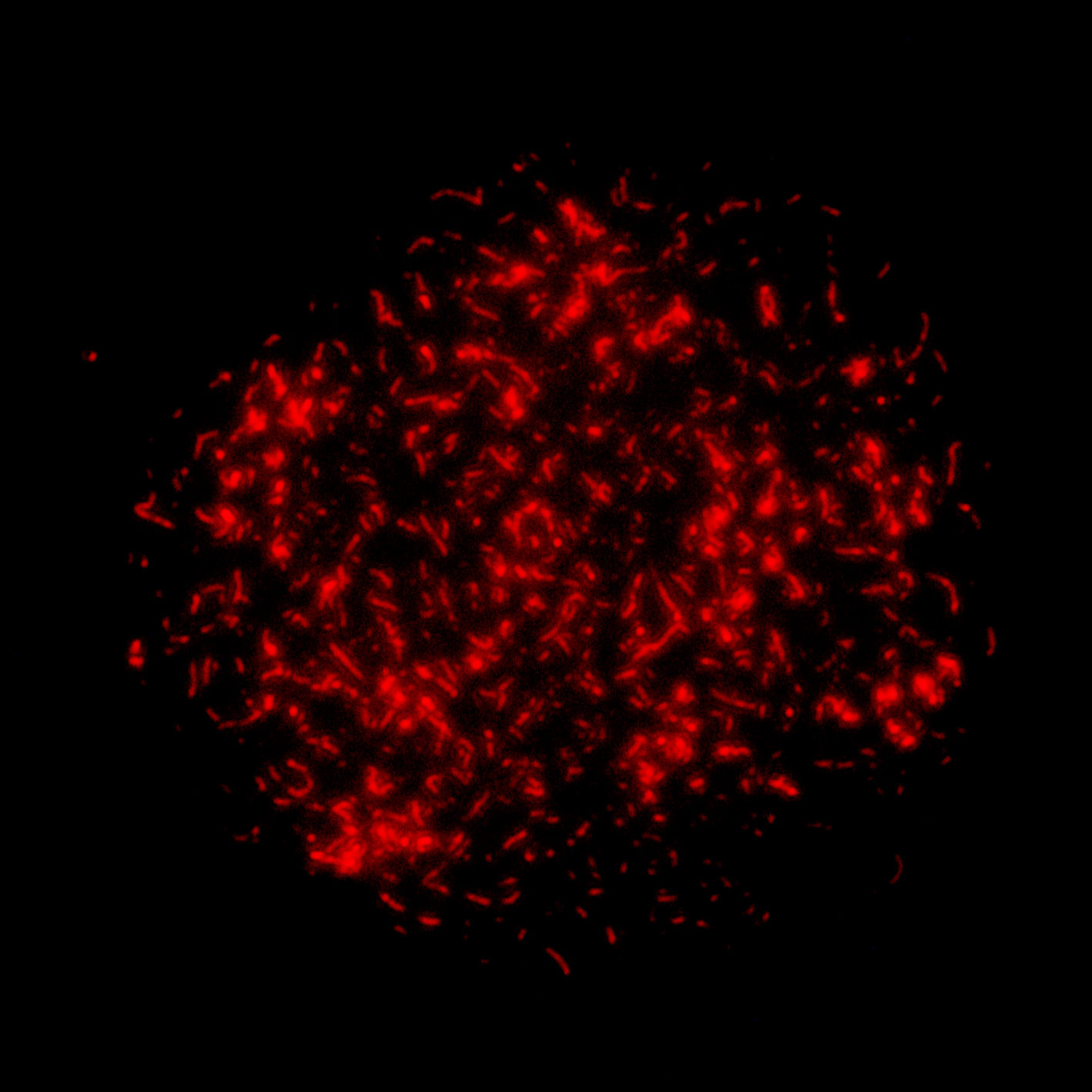

Supplement: Supplementary file 14 — EV and Appendix Figure Source Data [file 44318_2024_203_MOESM14_ESM.zip › Source Data for Expanded View and Appendix/Appendix Figure S2/S2E/Ctrl-Lep-SYCP3.jpg]

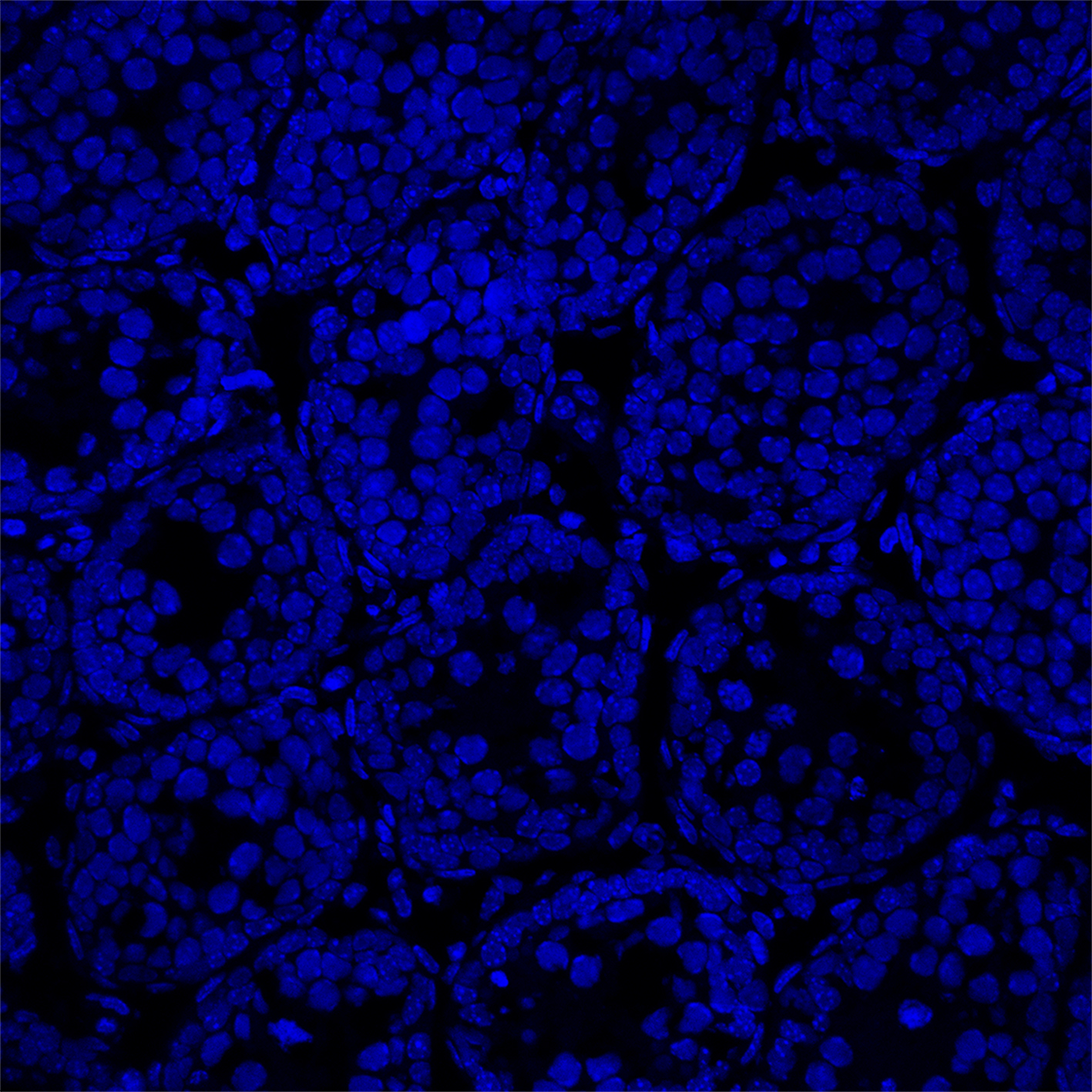

Supplement: Supplementary file 14 — EV and Appendix Figure Source Data [file 44318_2024_203_MOESM14_ESM.zip › Source Data for Expanded View and Appendix/Appendix Figure S2/S2B/Ctrl-DAPI.jpg]

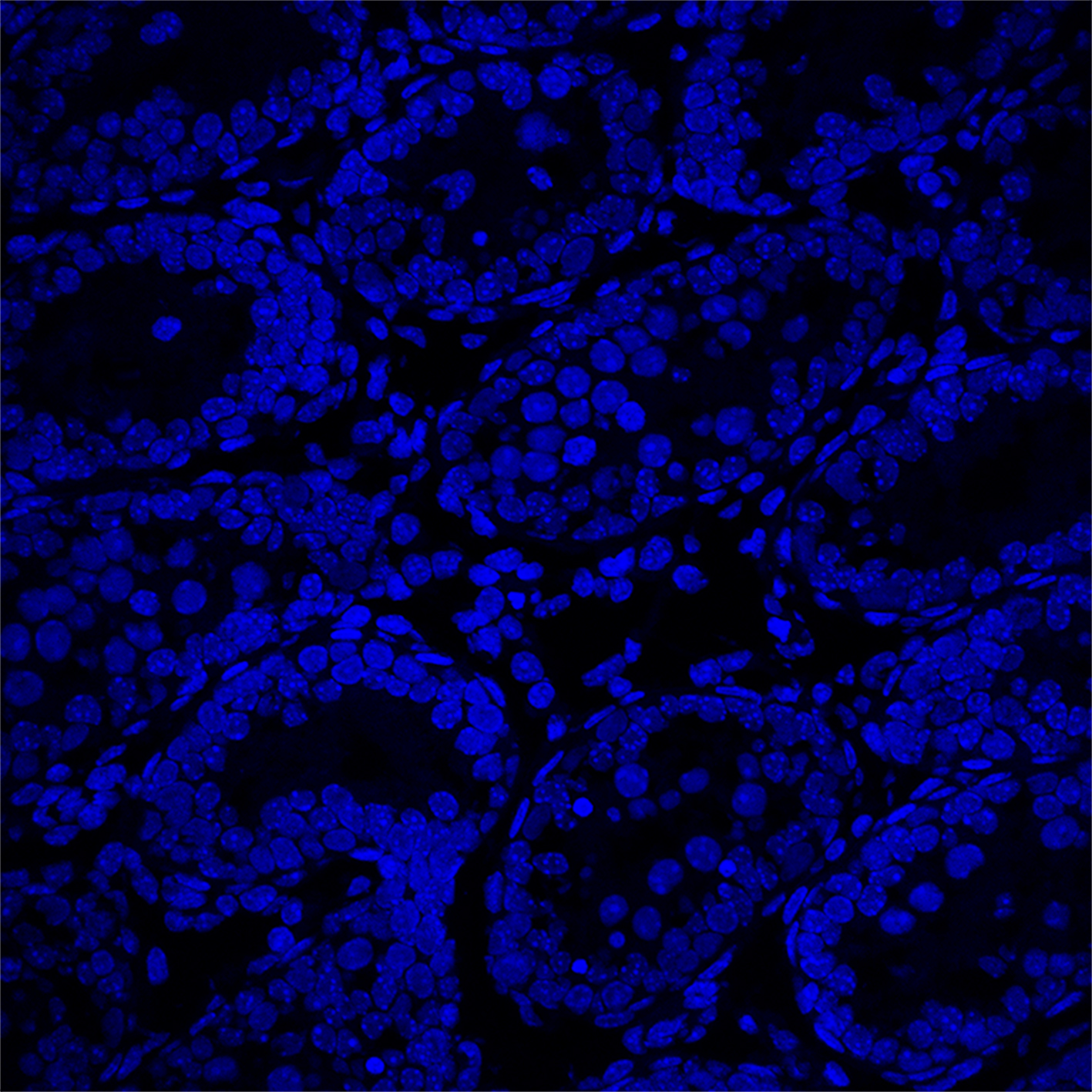

Supplement: Supplementary file 14 — EV and Appendix Figure Source Data [file 44318_2024_203_MOESM14_ESM.zip › Source Data for Expanded View and Appendix/Appendix Figure S2/S2B/cKO-DAPI.jpg]

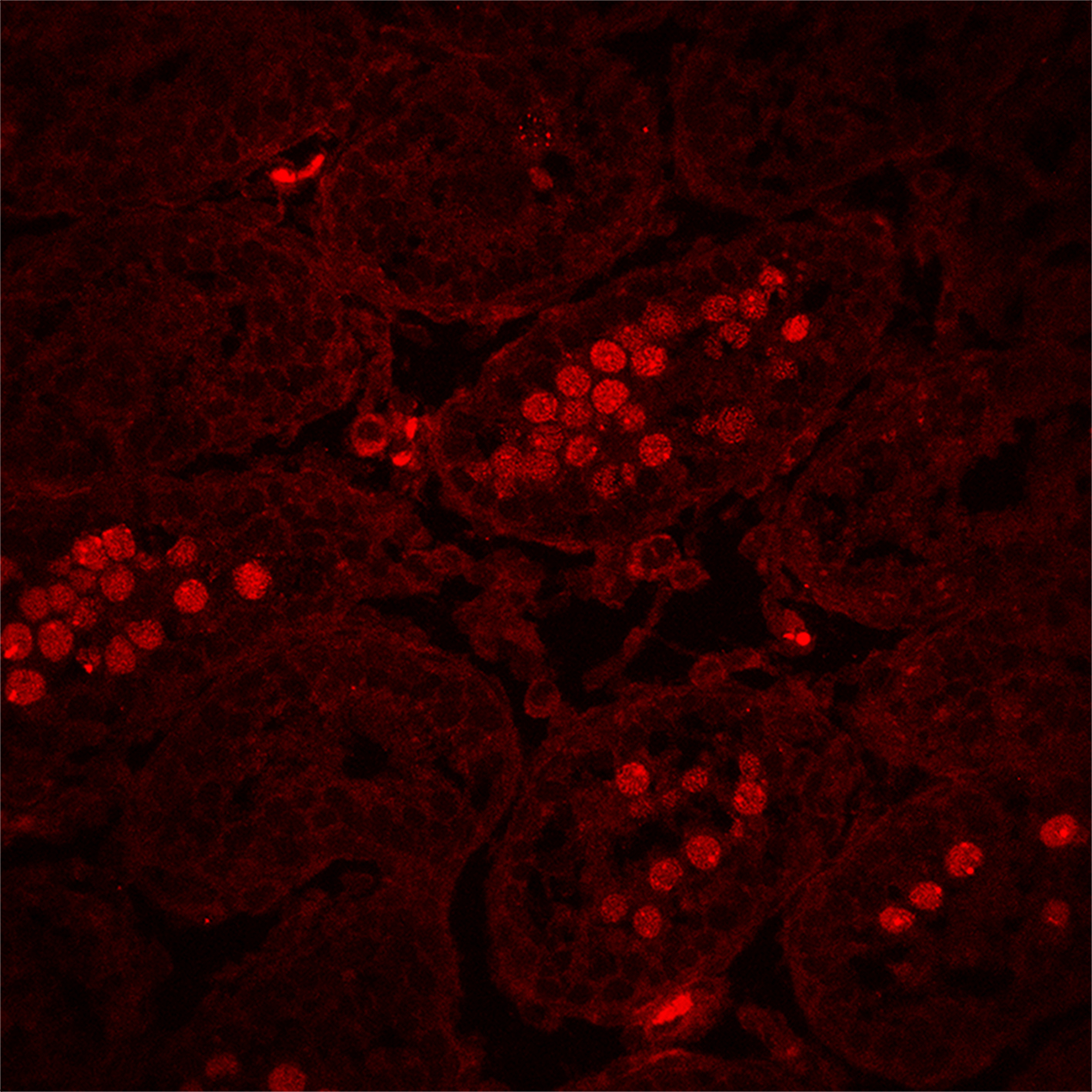

Supplement: Supplementary file 14 — EV and Appendix Figure Source Data [file 44318_2024_203_MOESM14_ESM.zip › Source Data for Expanded View and Appendix/Appendix Figure S2/S2B/cKO-SYCP3.jpg]

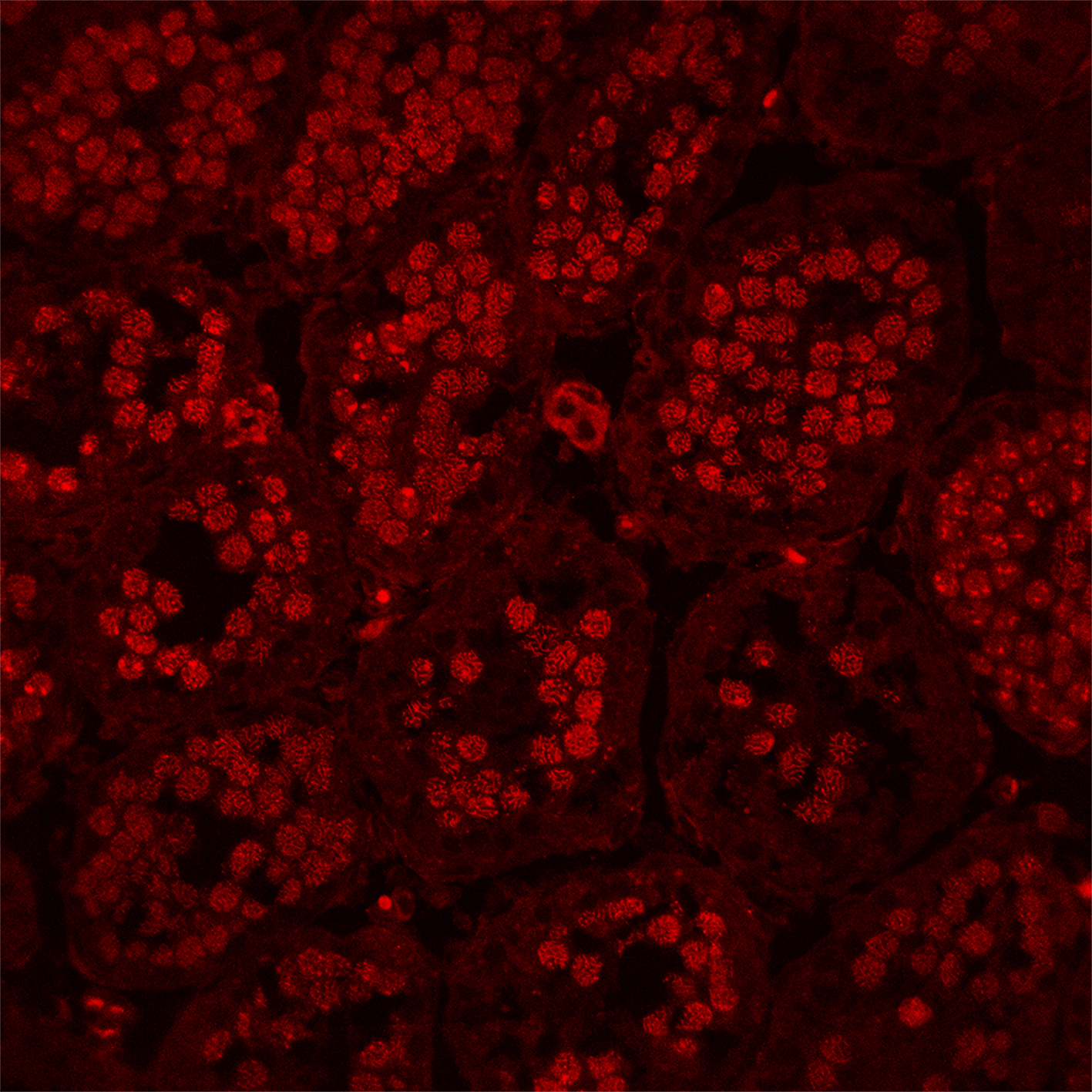

Supplement: Supplementary file 14 — EV and Appendix Figure Source Data [file 44318_2024_203_MOESM14_ESM.zip › Source Data for Expanded View and Appendix/Appendix Figure S2/S2B/Ctrl-SYCP3.jpg]

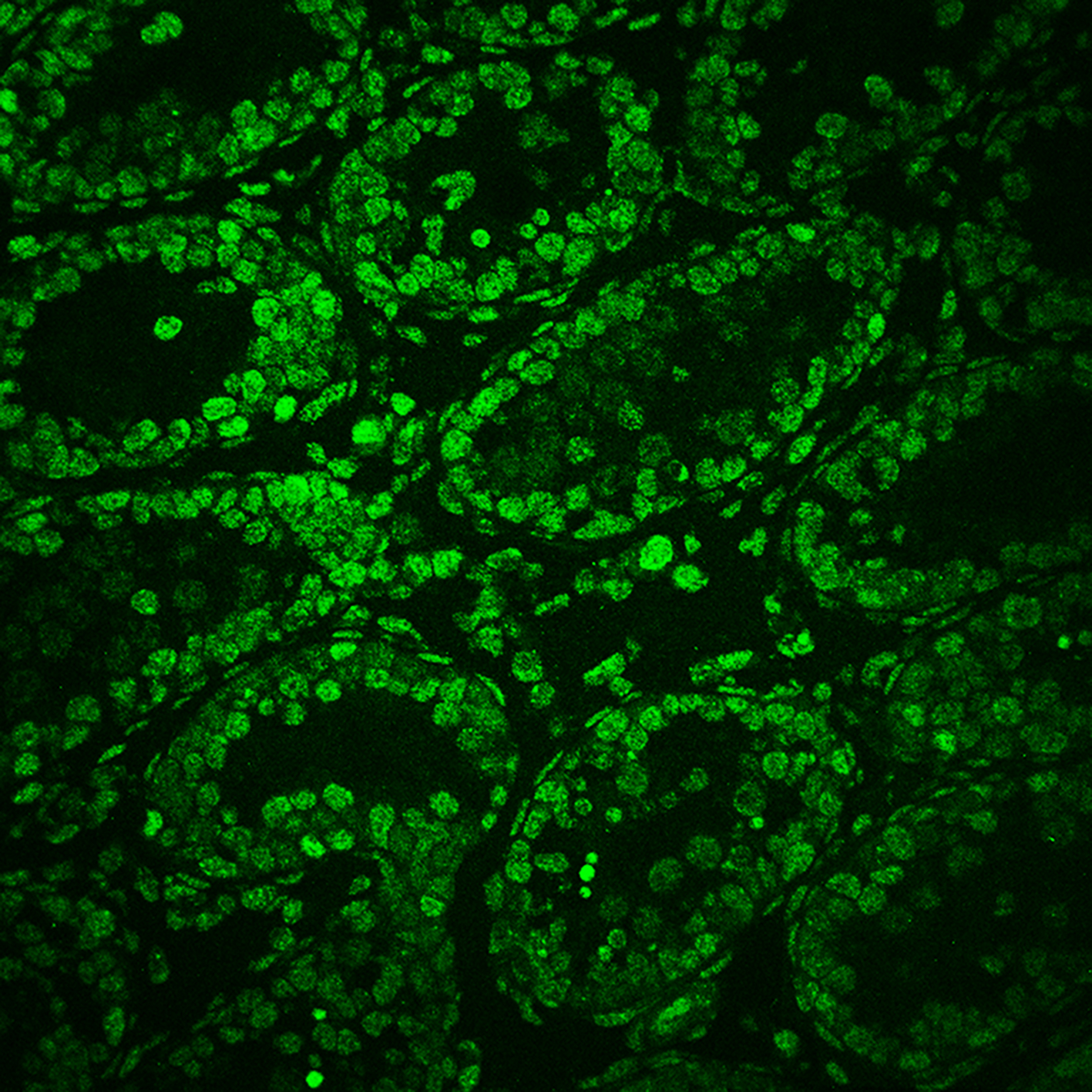

Supplement: Supplementary file 14 — EV and Appendix Figure Source Data [file 44318_2024_203_MOESM14_ESM.zip › Source Data for Expanded View and Appendix/Appendix Figure S2/S2B/cKO-H3K36me2.jpg]

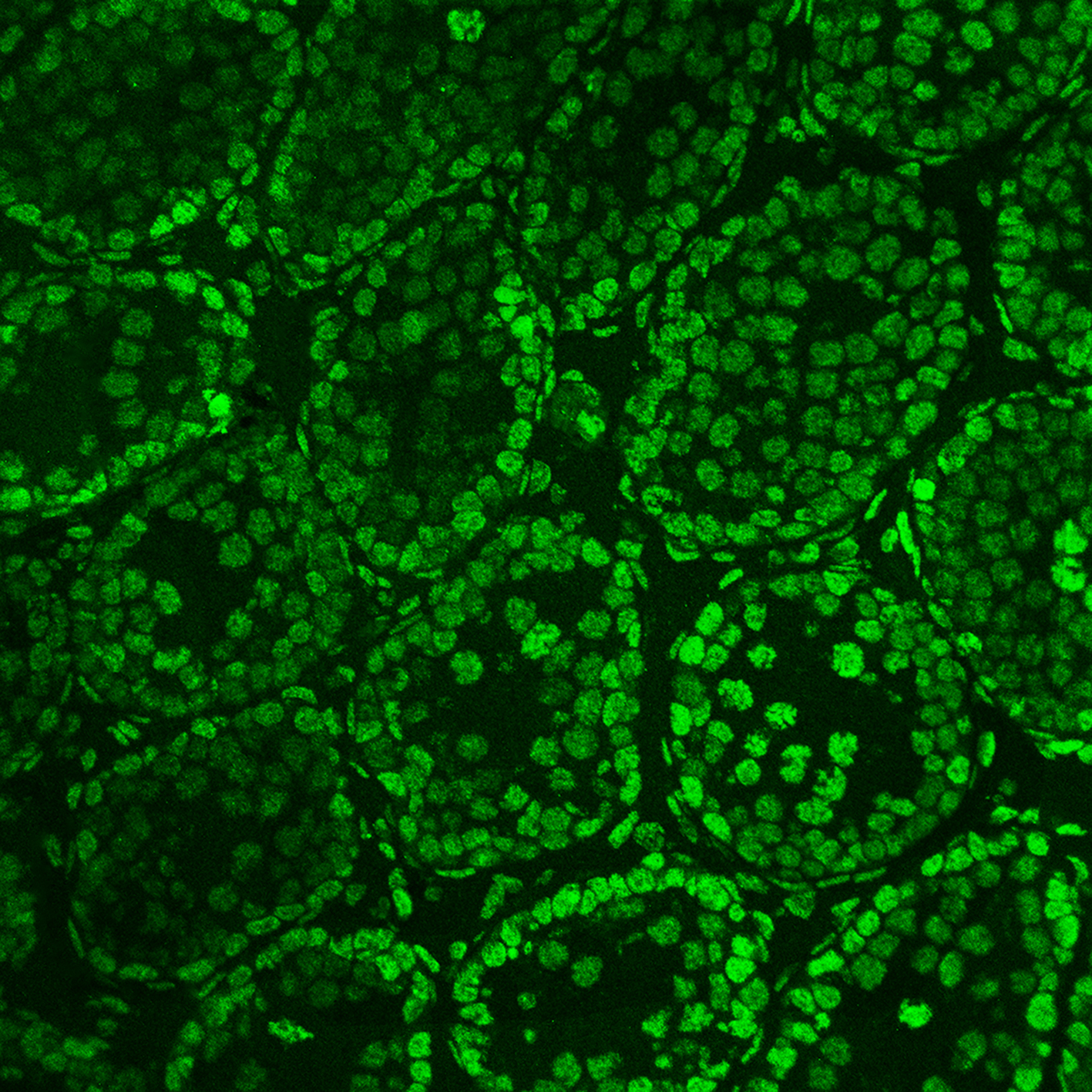

Supplement: Supplementary file 14 — EV and Appendix Figure Source Data [file 44318_2024_203_MOESM14_ESM.zip › Source Data for Expanded View and Appendix/Appendix Figure S2/S2B/Ctrl-H3K36me2.jpg]

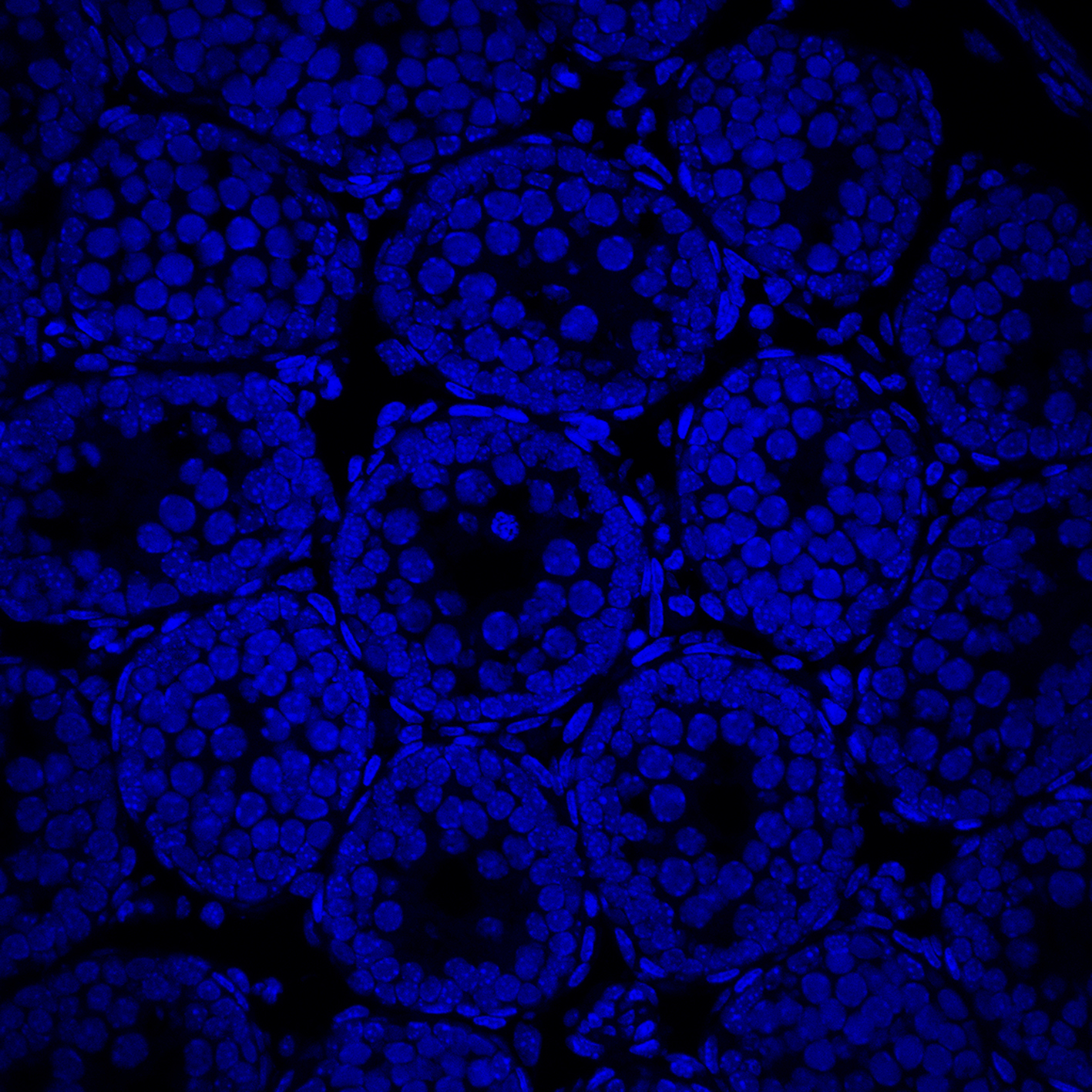

Supplement: Supplementary file 14 — EV and Appendix Figure Source Data [file 44318_2024_203_MOESM14_ESM.zip › Source Data for Expanded View and Appendix/Appendix Figure S2/S2C/Ctrl-DAPI.jpg]

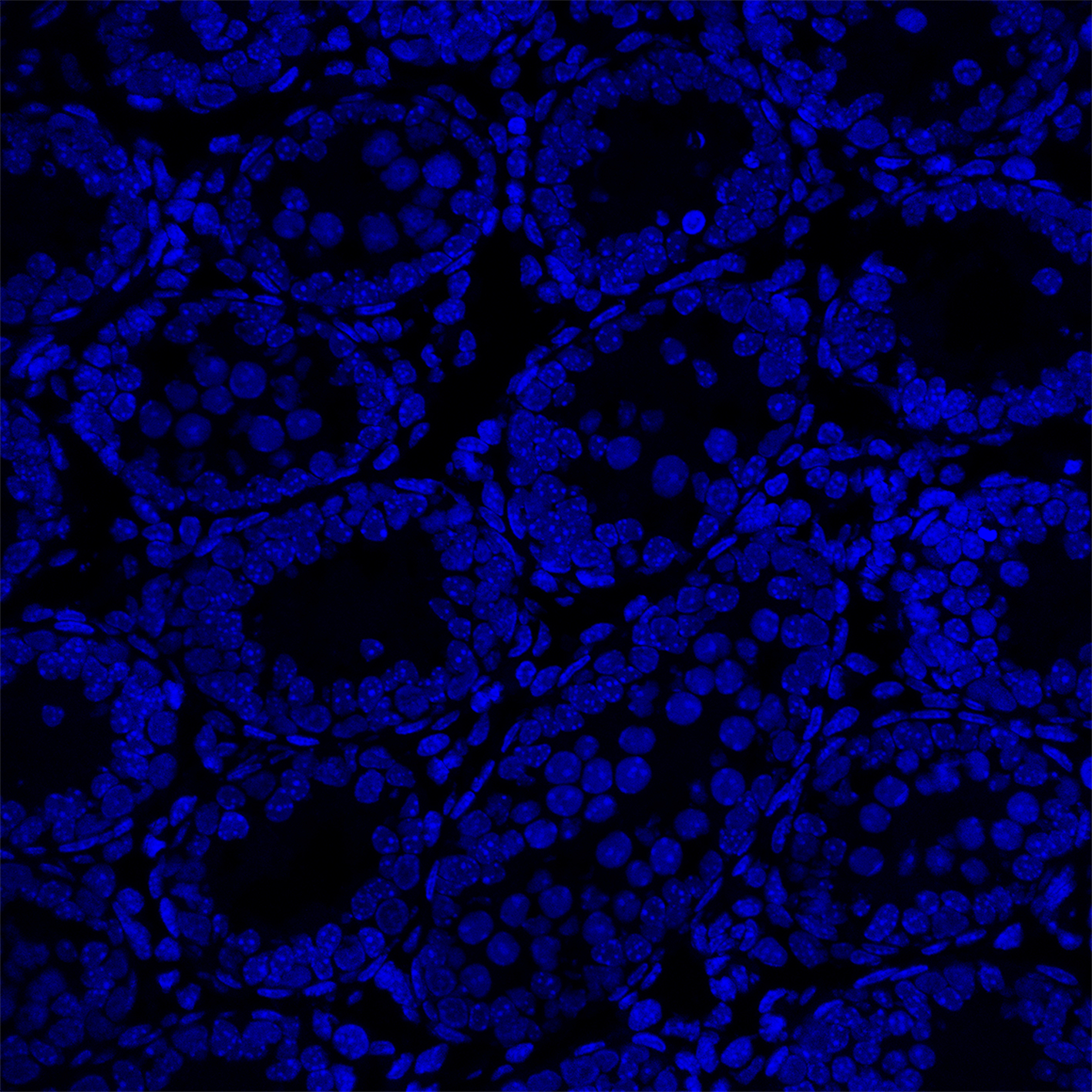

Supplement: Supplementary file 14 — EV and Appendix Figure Source Data [file 44318_2024_203_MOESM14_ESM.zip › Source Data for Expanded View and Appendix/Appendix Figure S2/S2C/cKO-DAPI.jpg]

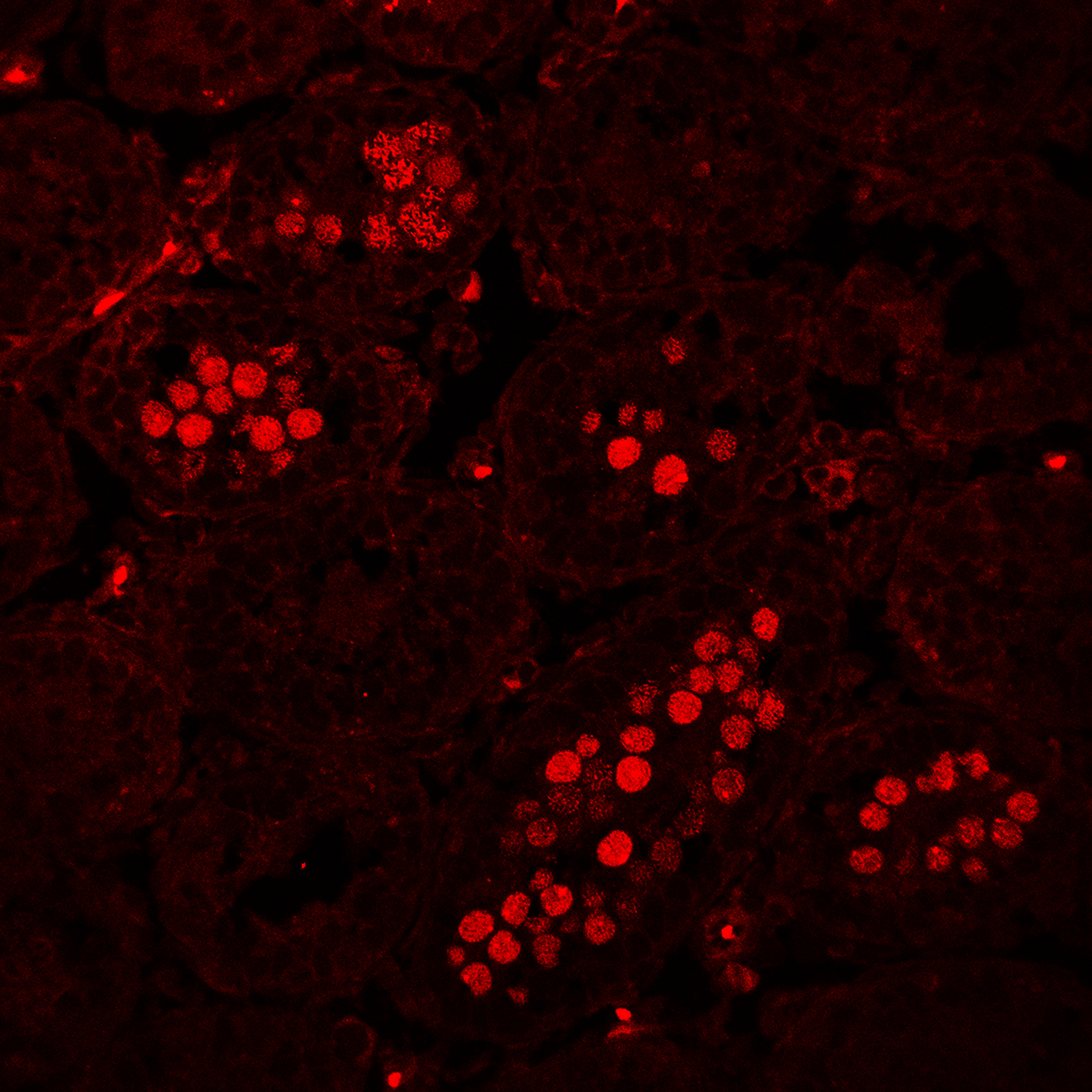

Supplement: Supplementary file 14 — EV and Appendix Figure Source Data [file 44318_2024_203_MOESM14_ESM.zip › Source Data for Expanded View and Appendix/Appendix Figure S2/S2C/cKO-SYCP3.jpg]

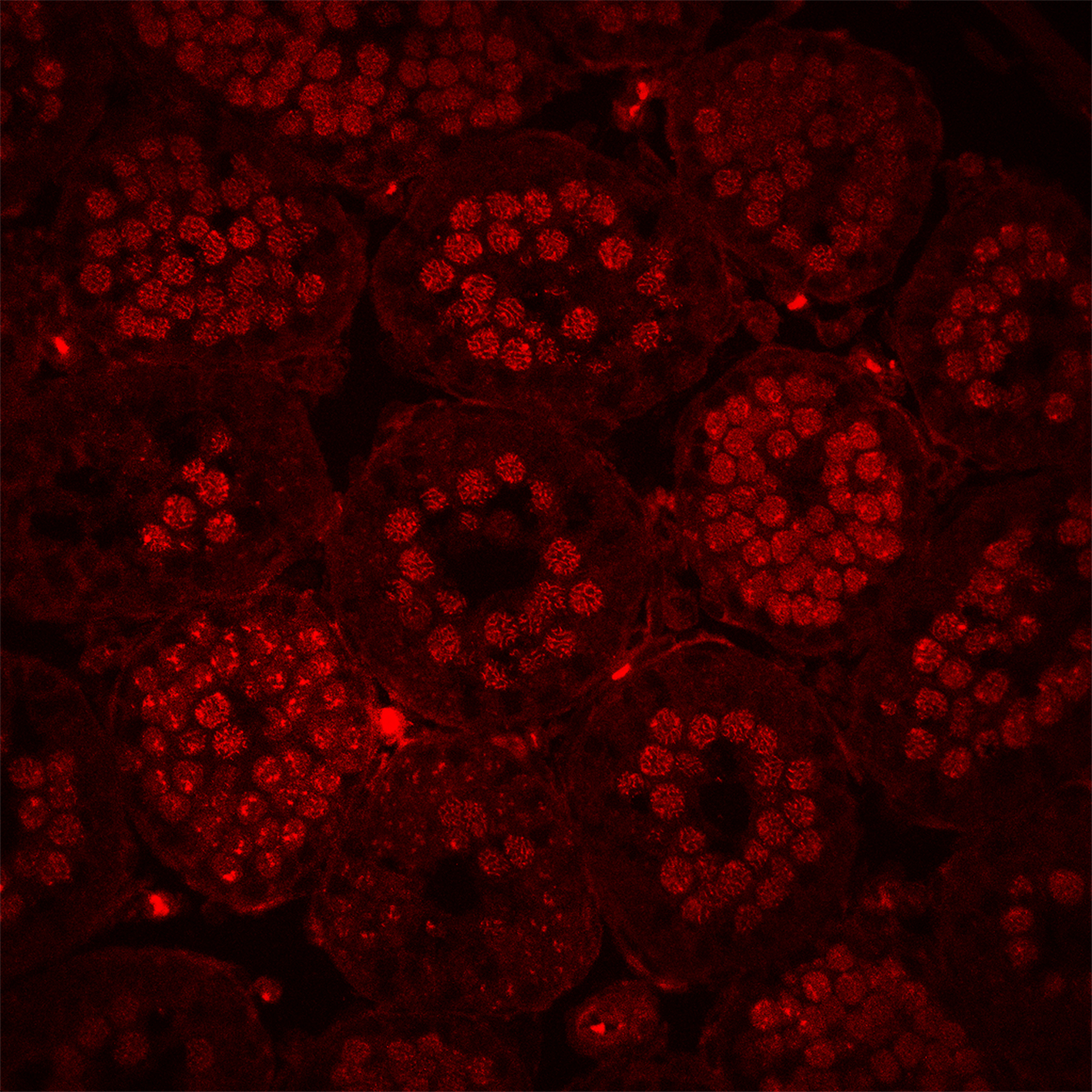

Supplement: Supplementary file 14 — EV and Appendix Figure Source Data [file 44318_2024_203_MOESM14_ESM.zip › Source Data for Expanded View and Appendix/Appendix Figure S2/S2C/Ctrl-SYCP3.jpg]

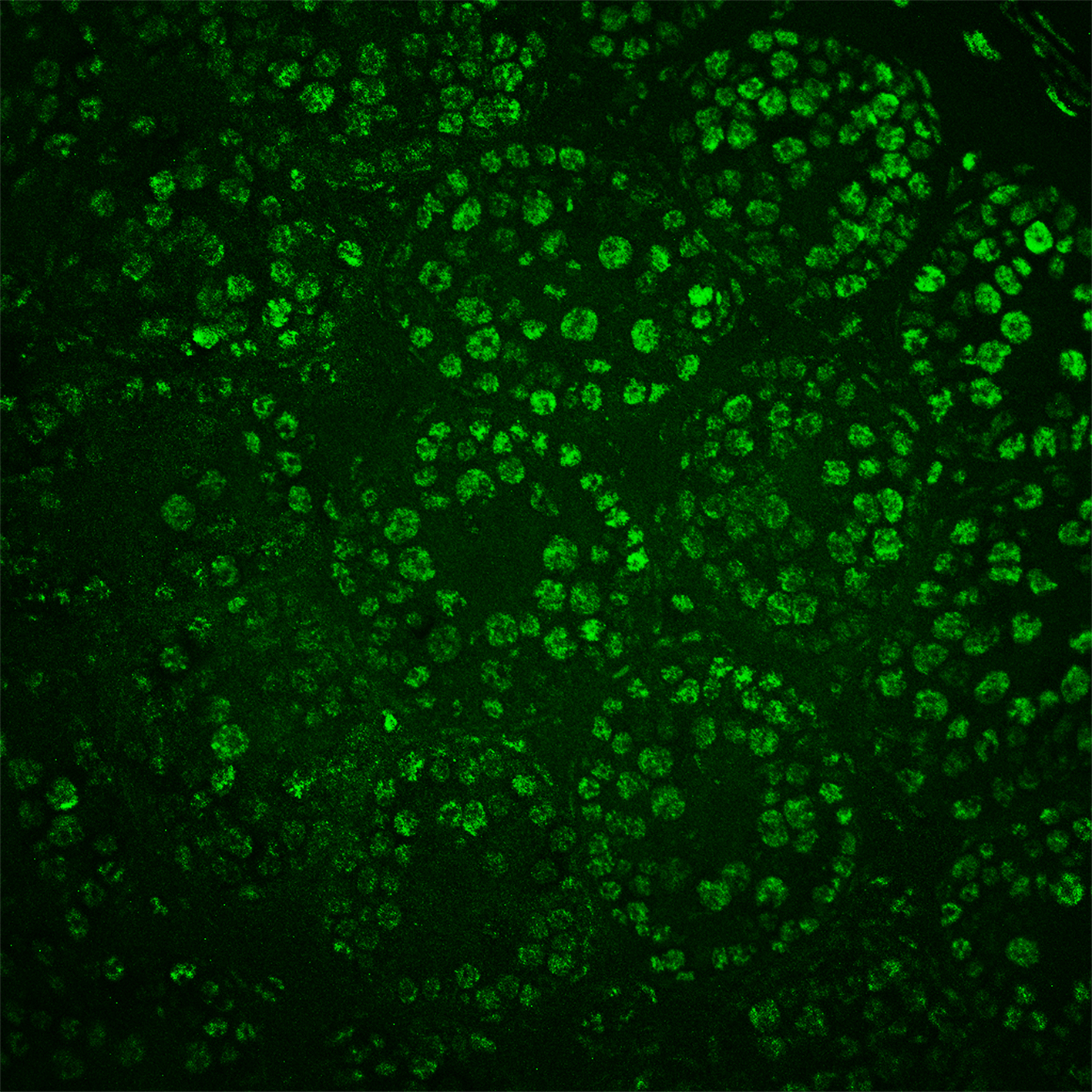

Supplement: Supplementary file 14 — EV and Appendix Figure Source Data [file 44318_2024_203_MOESM14_ESM.zip › Source Data for Expanded View and Appendix/Appendix Figure S2/S2C/Ctrl-H3K36me3.jpg]

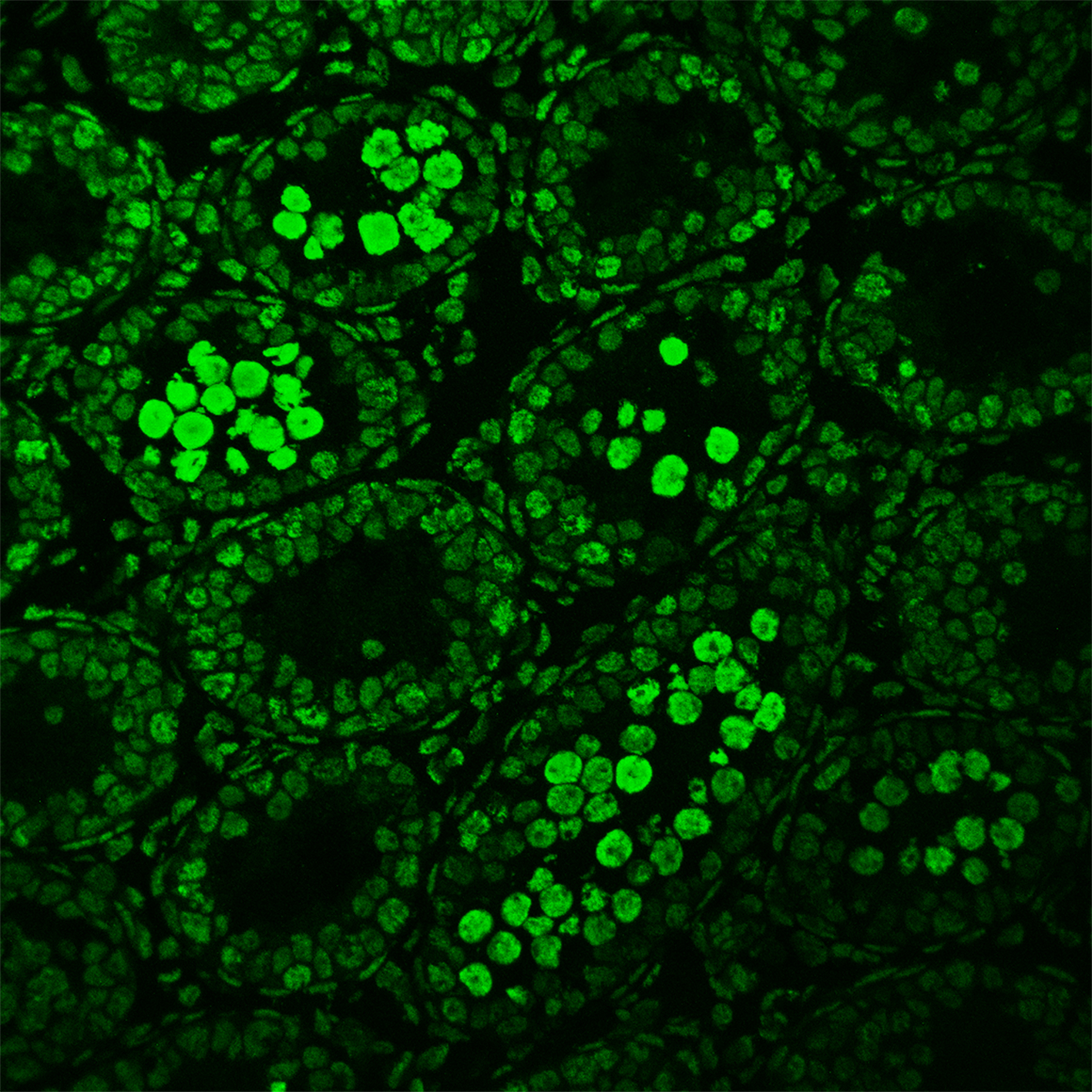

Supplement: Supplementary file 14 — EV and Appendix Figure Source Data [file 44318_2024_203_MOESM14_ESM.zip › Source Data for Expanded View and Appendix/Appendix Figure S2/S2C/cKO-H3K36me3.jpg]

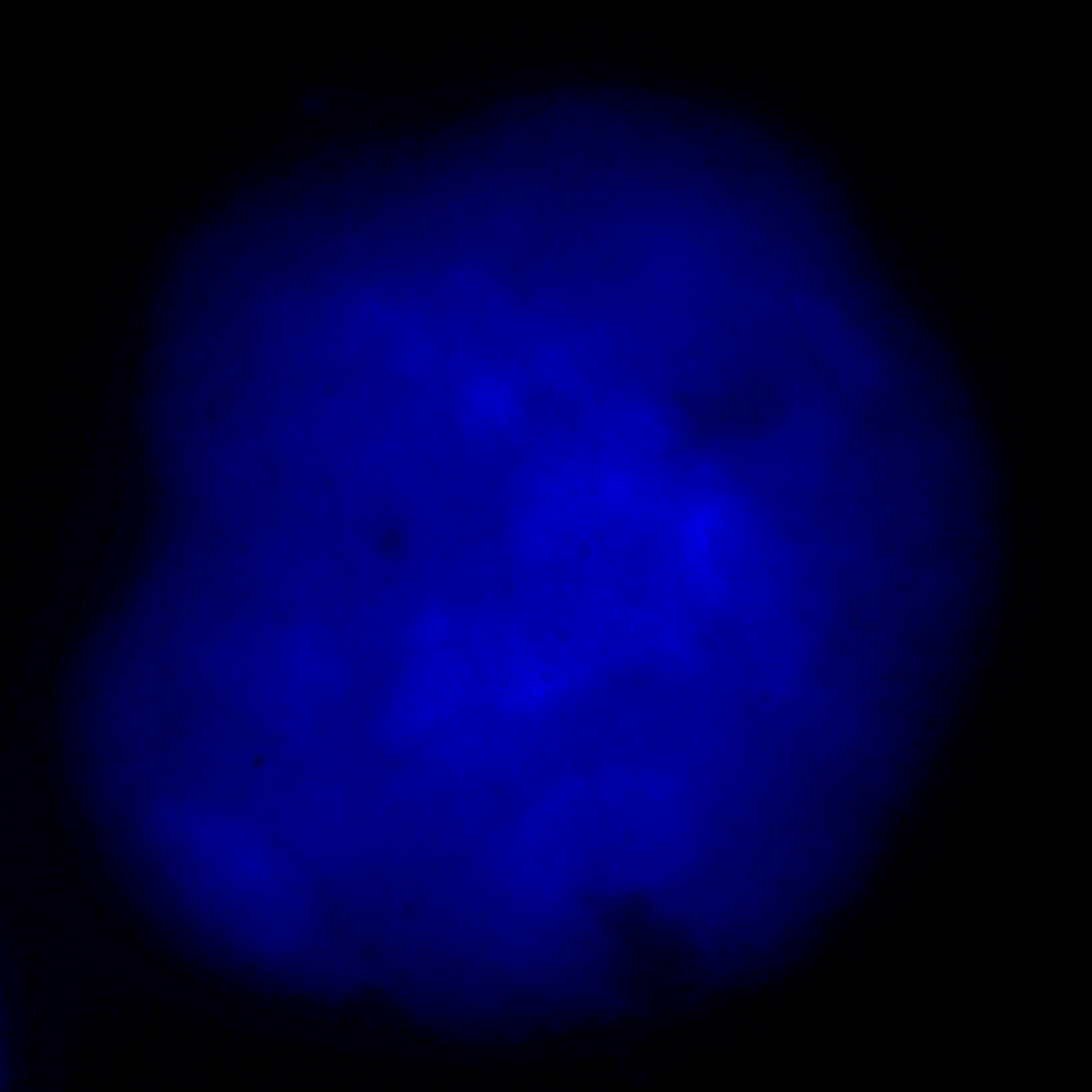

Supplement: Supplementary file 14 — EV and Appendix Figure Source Data [file 44318_2024_203_MOESM14_ESM.zip › Source Data for Expanded View and Appendix/Appendix Figure S2/S2D/cKO-Zyg-ii-DAPI.jpg]

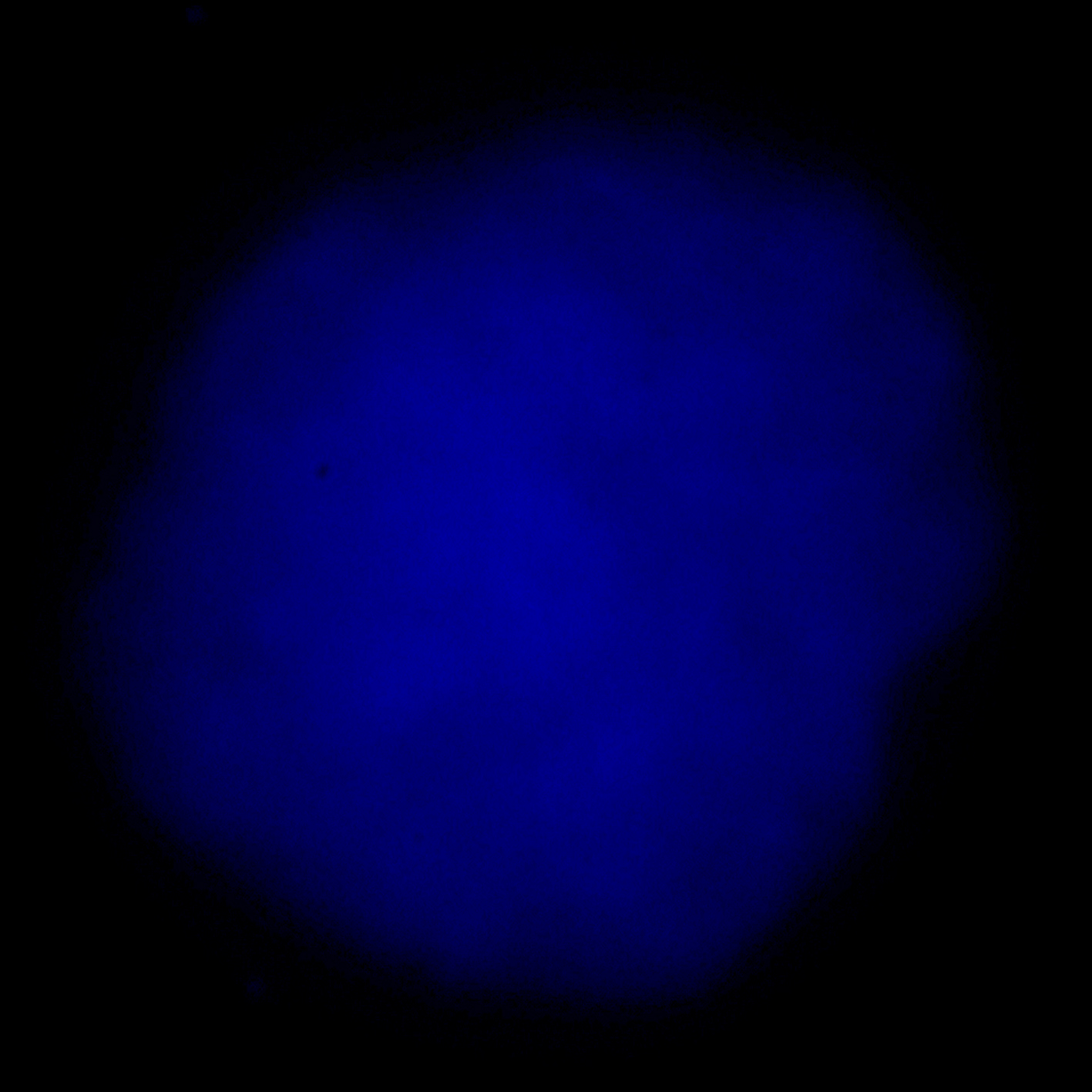

Supplement: Supplementary file 14 — EV and Appendix Figure Source Data [file 44318_2024_203_MOESM14_ESM.zip › Source Data for Expanded View and Appendix/Appendix Figure S2/S2D/cKO-Zyg-i-DAPI.jpg]

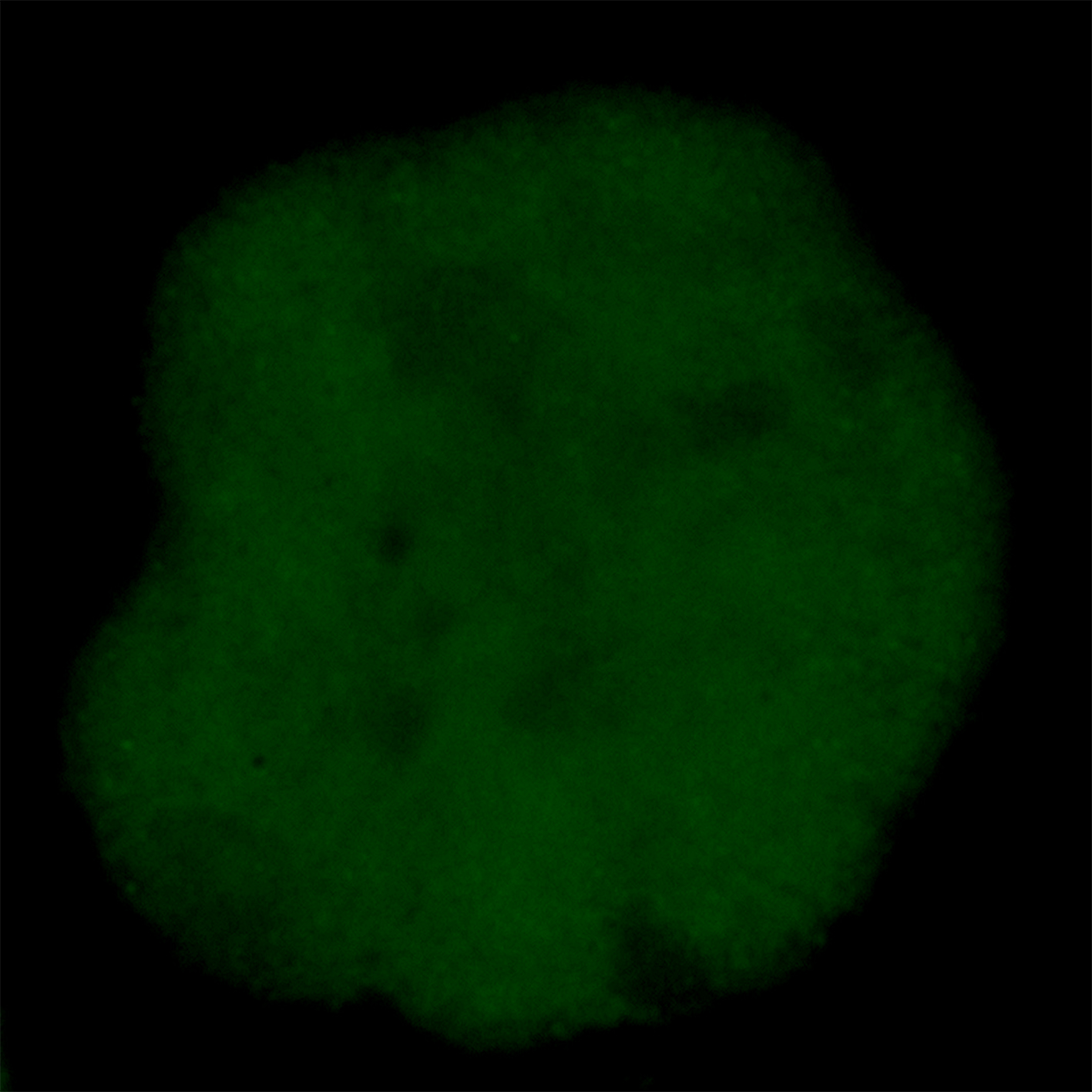

Supplement: Supplementary file 14 — EV and Appendix Figure Source Data [file 44318_2024_203_MOESM14_ESM.zip › Source Data for Expanded View and Appendix/Appendix Figure S2/S2D/cKO-Zyg-ii-H3K36me2.jpg]

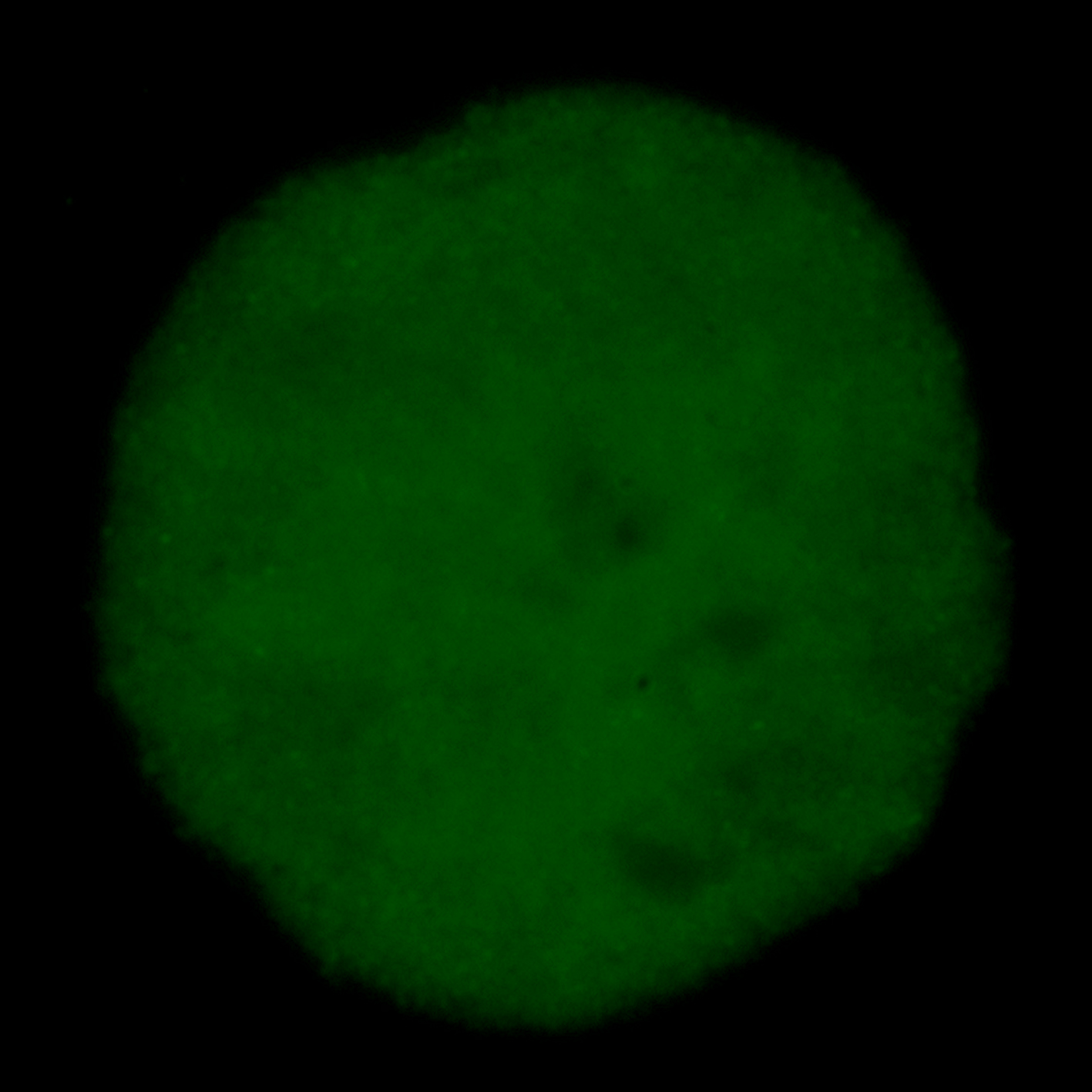

Supplement: Supplementary file 14 — EV and Appendix Figure Source Data [file 44318_2024_203_MOESM14_ESM.zip › Source Data for Expanded View and Appendix/Appendix Figure S2/S2D/cKO-lep-H3K36me2.jpg]

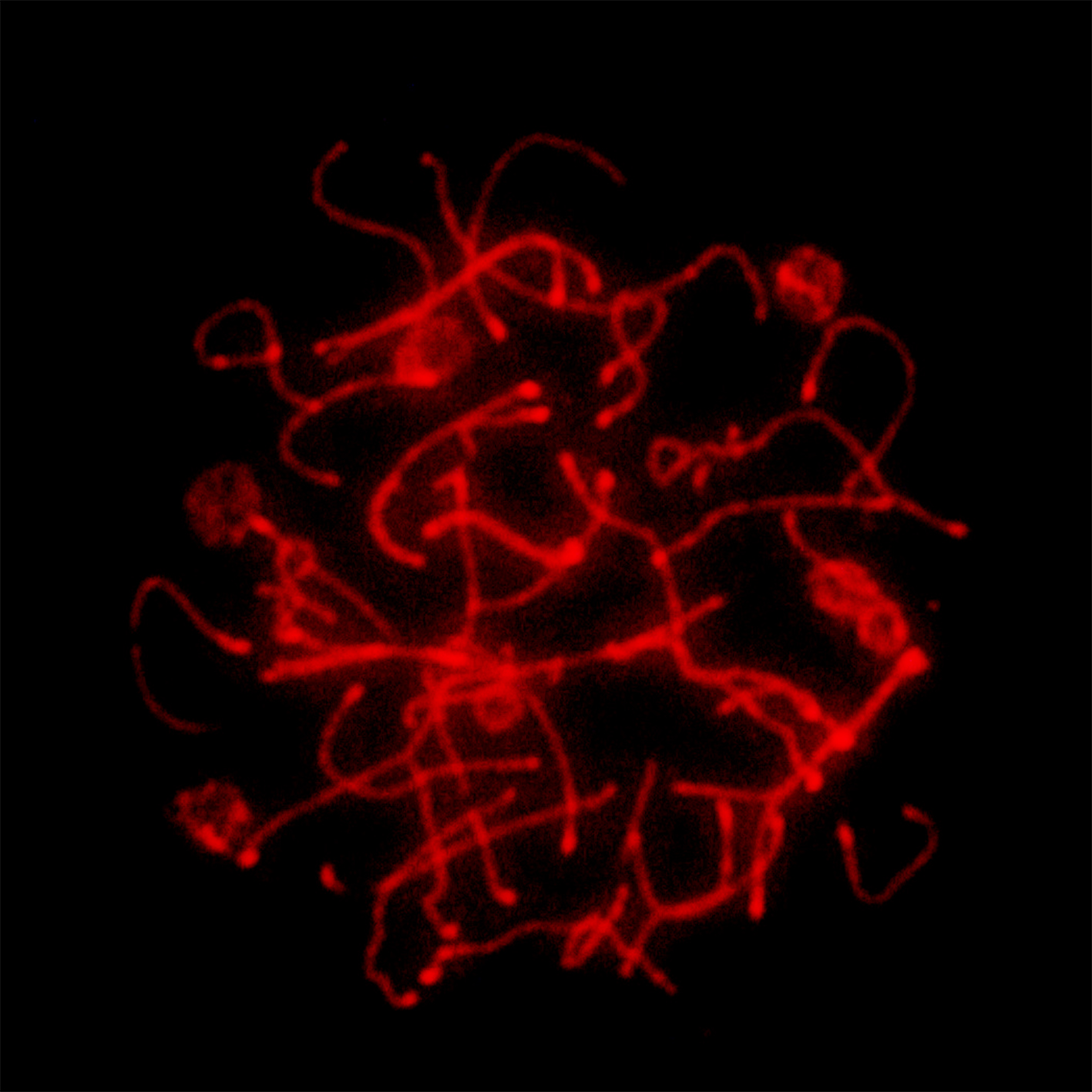

Supplement: Supplementary file 14 — EV and Appendix Figure Source Data [file 44318_2024_203_MOESM14_ESM.zip › Source Data for Expanded View and Appendix/Appendix Figure S2/S2D/Ctrl-Zyg-SYCP3.jpg]

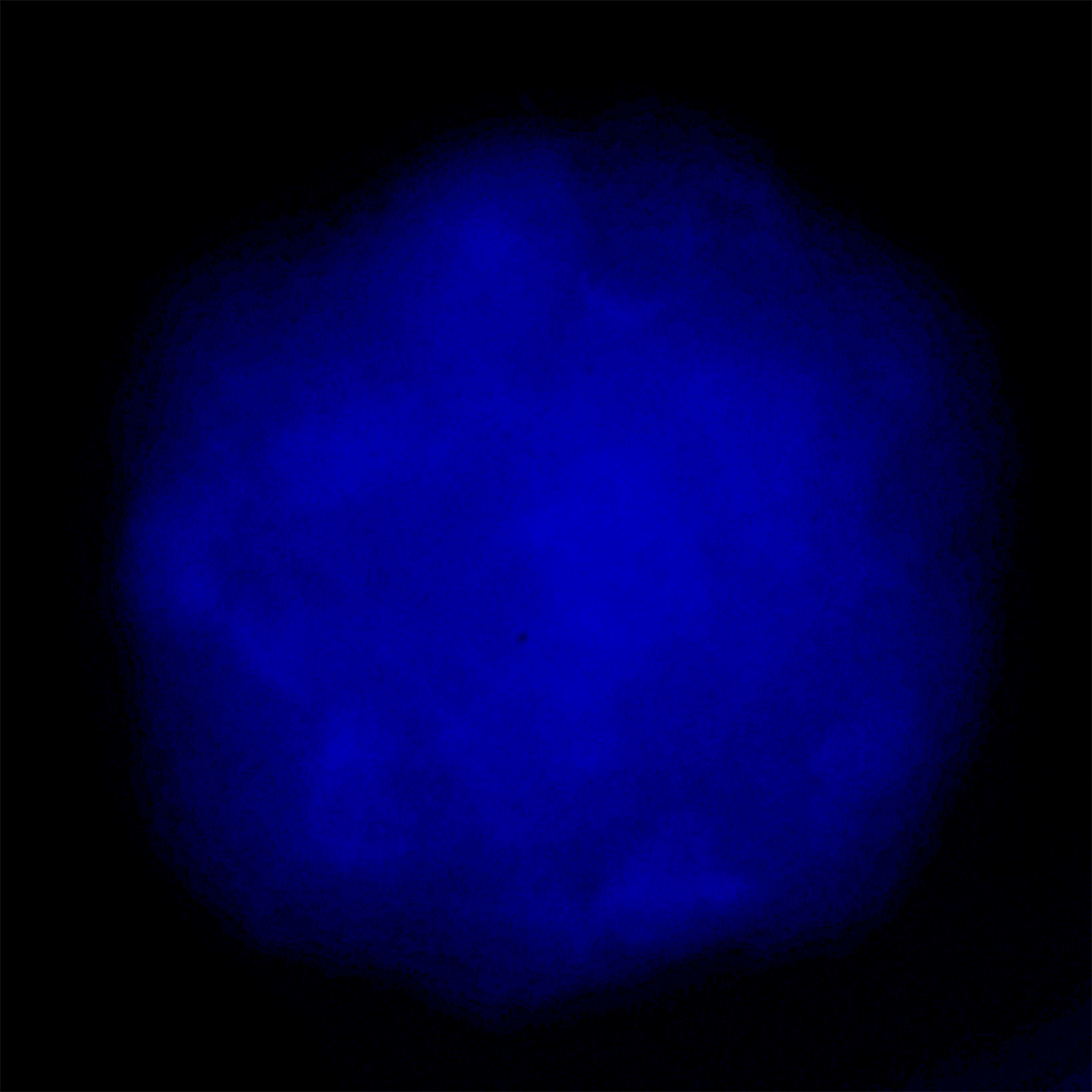

Supplement: Supplementary file 14 — EV and Appendix Figure Source Data [file 44318_2024_203_MOESM14_ESM.zip › Source Data for Expanded View and Appendix/Appendix Figure S2/S2D/Ctrl-Pac-DAPI.jpg]

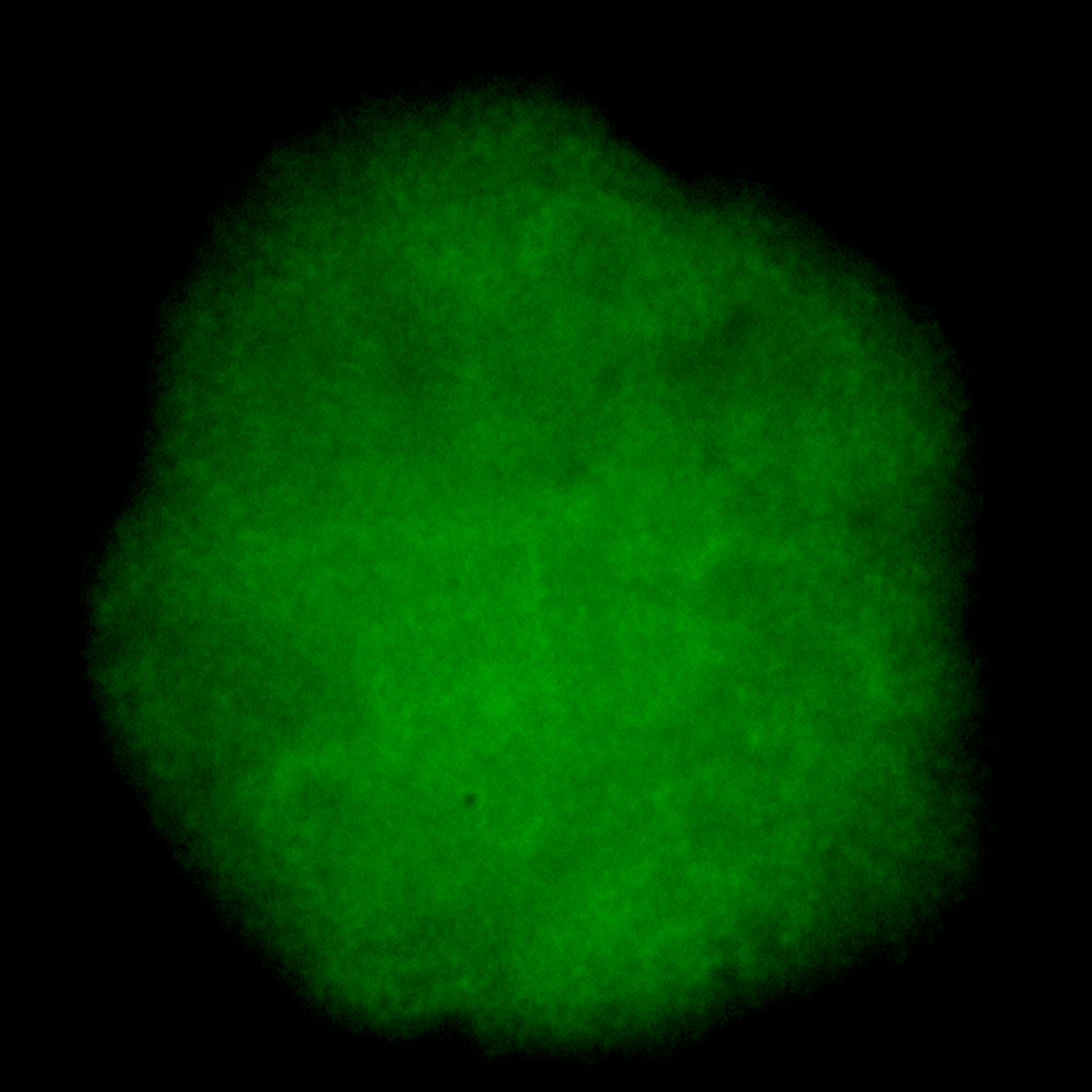

Supplement: Supplementary file 14 — EV and Appendix Figure Source Data [file 44318_2024_203_MOESM14_ESM.zip › Source Data for Expanded View and Appendix/Appendix Figure S2/S2D/Ctrl-Zyg-H3K36me2.jpg]

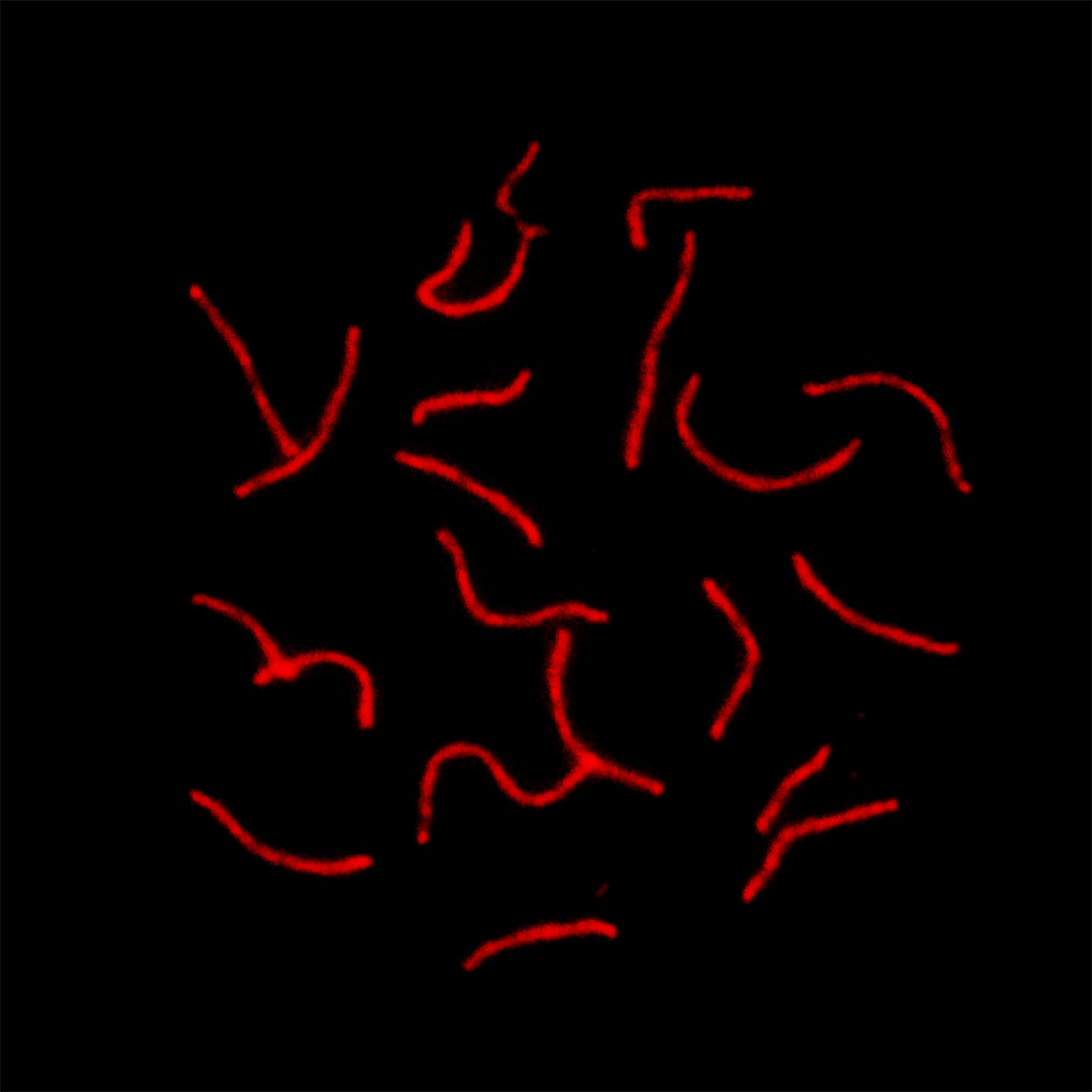

Supplement: Supplementary file 14 — EV and Appendix Figure Source Data [file 44318_2024_203_MOESM14_ESM.zip › Source Data for Expanded View and Appendix/Appendix Figure S2/S2D/Ctrl-Pac-SYCP3.jpg]
