# Supplementary figures and images for: Histone demethylase KDM2A recruits HCFC1 and E2F1 to orchestrate male germ cell meiotic entry and progression (part 3 of 4)
Source: EMBO J. 2024 Aug 19;43(19):4197–227. doi: 10.1038/s44318-024-00203-4 (PMC11448500; doi:10.1038/s44318-024-00203-4)

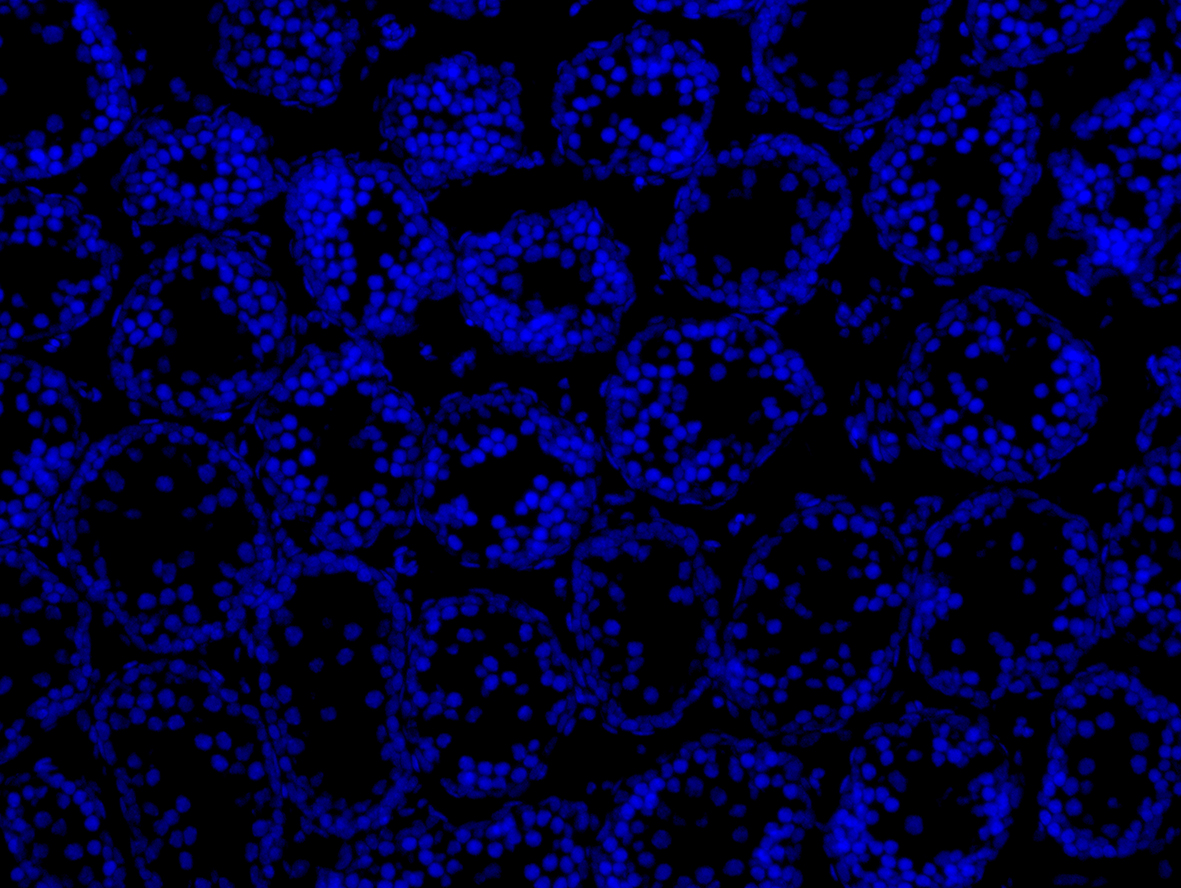

Supplement: Supplementary file 14 — EV and Appendix Figure Source Data [file 44318_2024_203_MOESM14_ESM.zip › Source Data for Expanded View and Appendix/Figure EV4/EV4G/Low magnification of P12 control - DAPI.jpg]

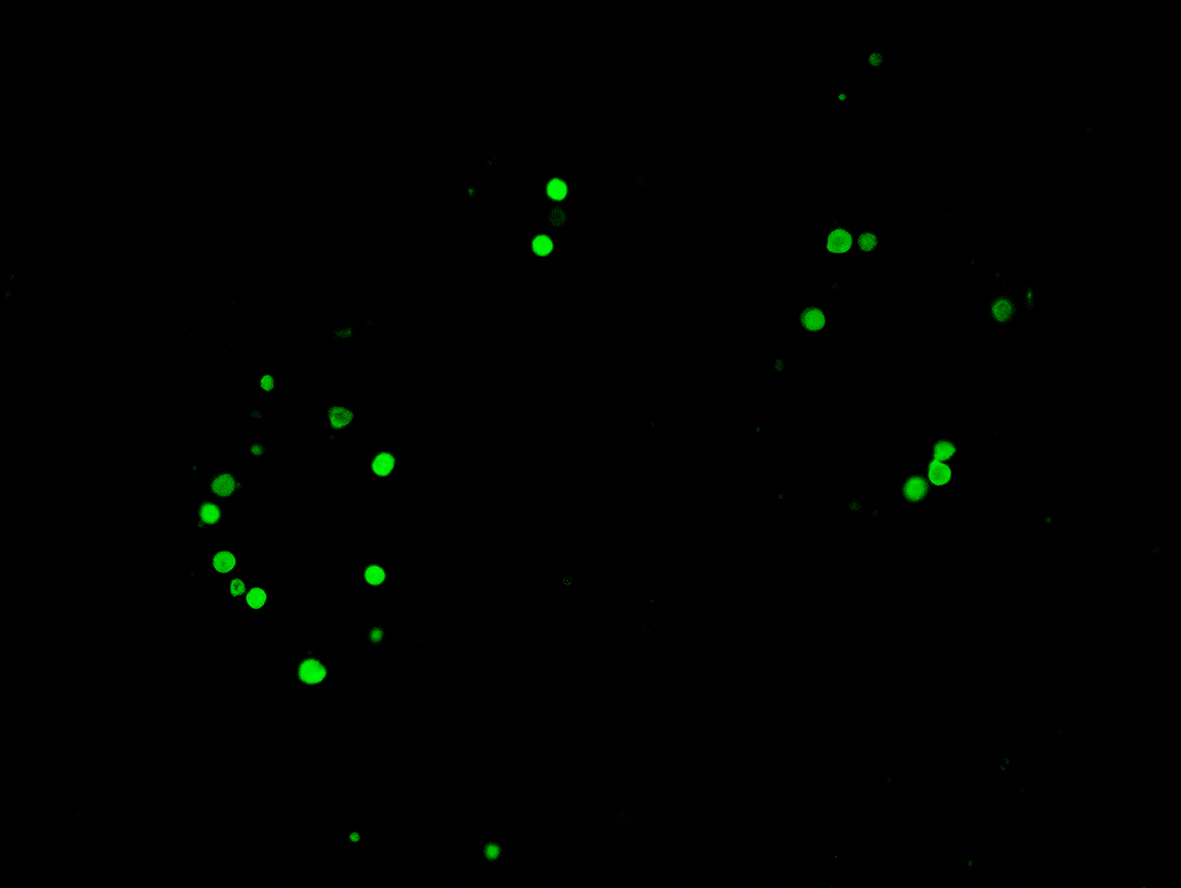

Supplement: Supplementary file 14 — EV and Appendix Figure Source Data [file 44318_2024_203_MOESM14_ESM.zip › Source Data for Expanded View and Appendix/Figure EV4/EV4G/High magnification of P12 Kdm2a cKO - Tunnel signal.jpg]

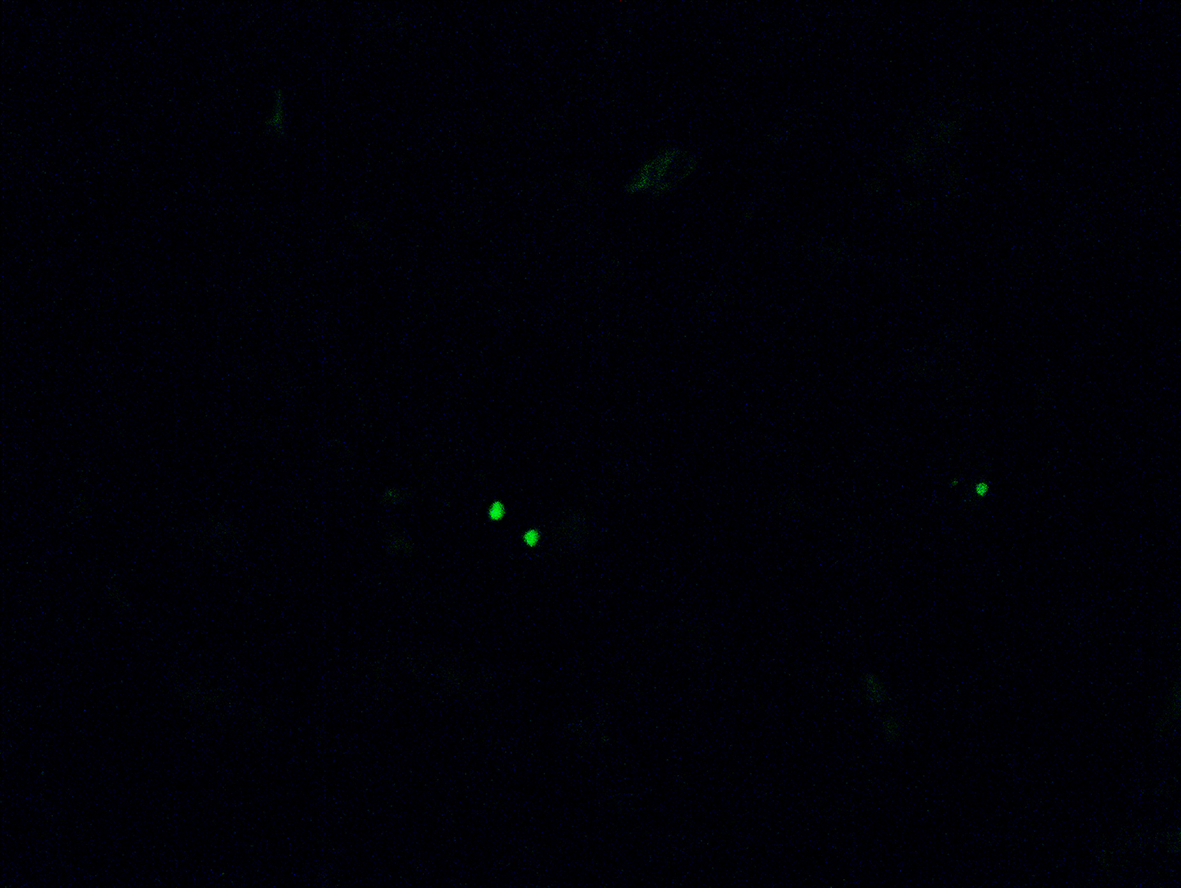

Supplement: Supplementary file 14 — EV and Appendix Figure Source Data [file 44318_2024_203_MOESM14_ESM.zip › Source Data for Expanded View and Appendix/Figure EV4/EV4G/High magnification of P10 control - Tunnel signal.jpg]

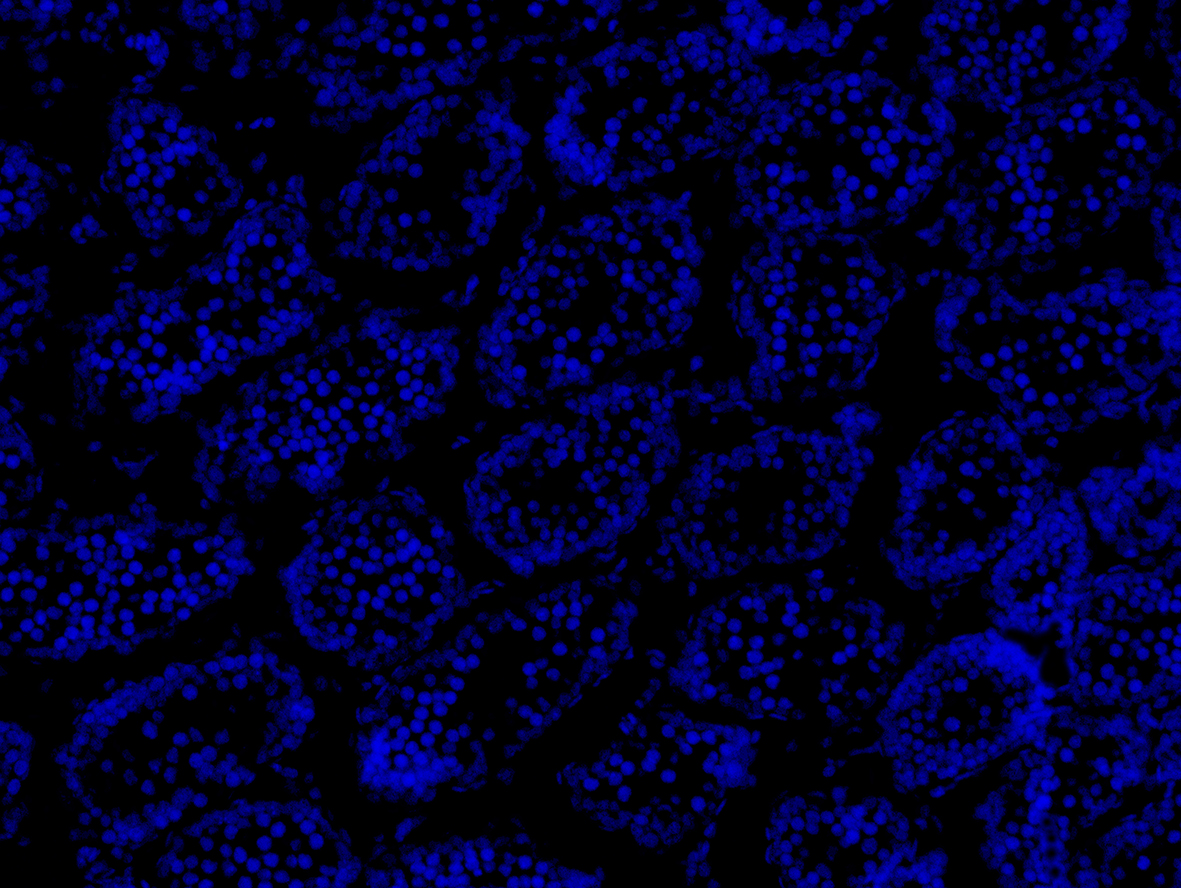

Supplement: Supplementary file 14 — EV and Appendix Figure Source Data [file 44318_2024_203_MOESM14_ESM.zip › Source Data for Expanded View and Appendix/Figure EV4/EV4G/Low magnification of P10 control - DAPI.jpg]

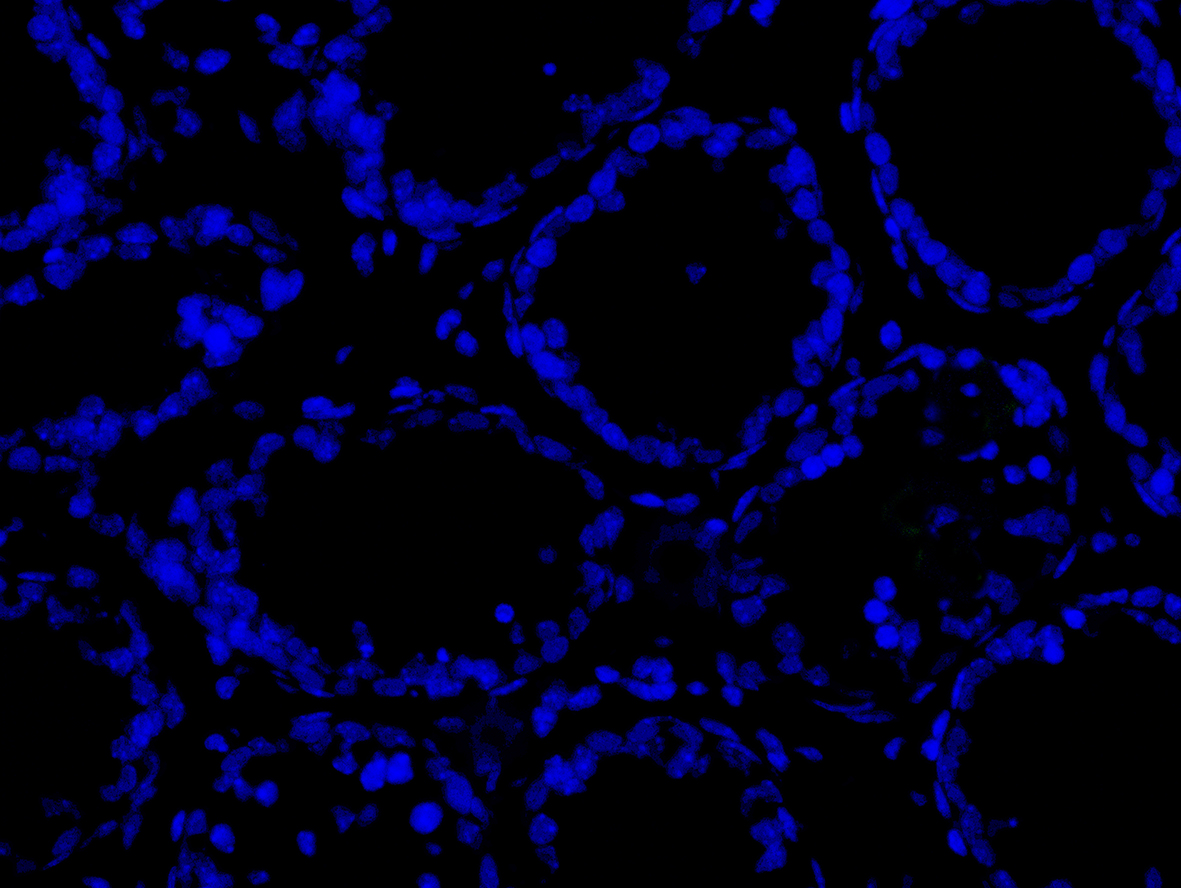

Supplement: Supplementary file 14 — EV and Appendix Figure Source Data [file 44318_2024_203_MOESM14_ESM.zip › Source Data for Expanded View and Appendix/Figure EV4/EV4G/High magnification of P14 cKO - DAPI.jpg]

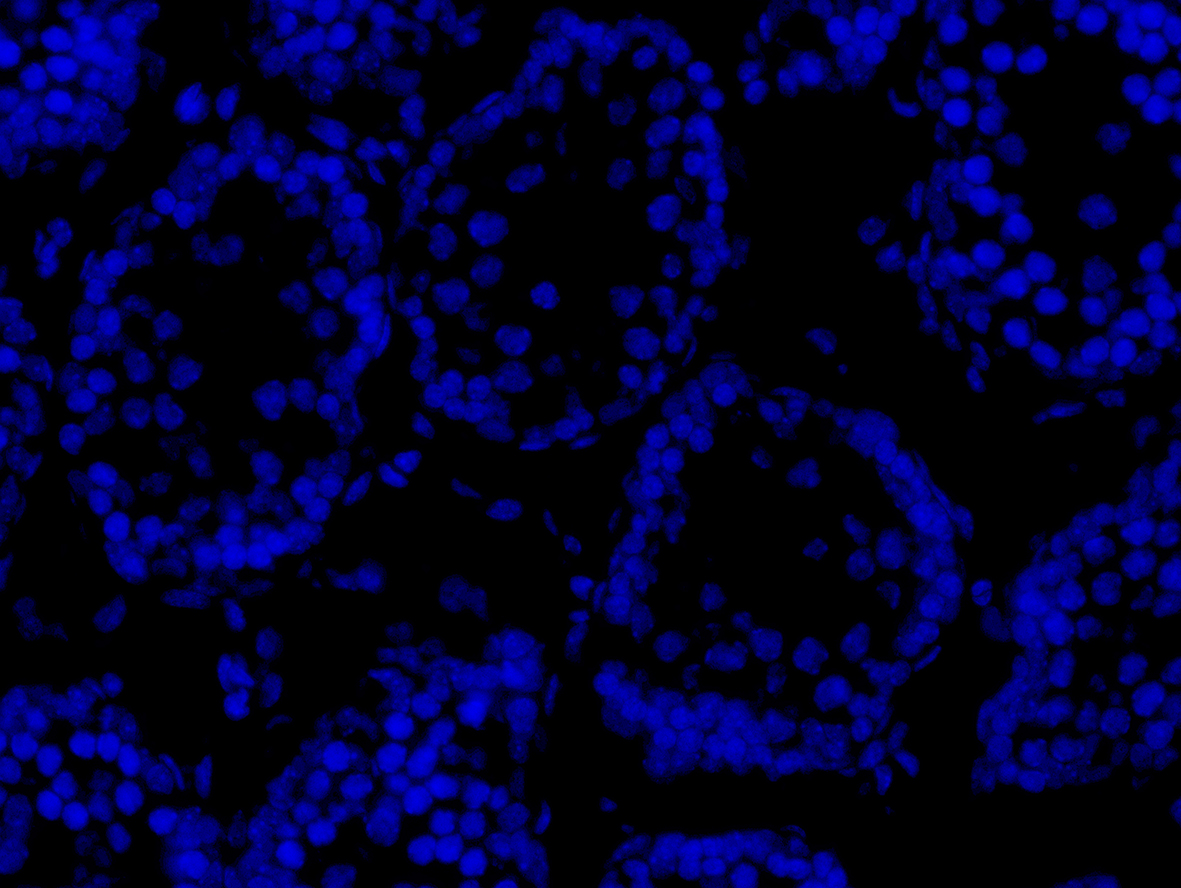

Supplement: Supplementary file 14 — EV and Appendix Figure Source Data [file 44318_2024_203_MOESM14_ESM.zip › Source Data for Expanded View and Appendix/Figure EV4/EV4G/High magnification of P14 control - DAPI.jpg]

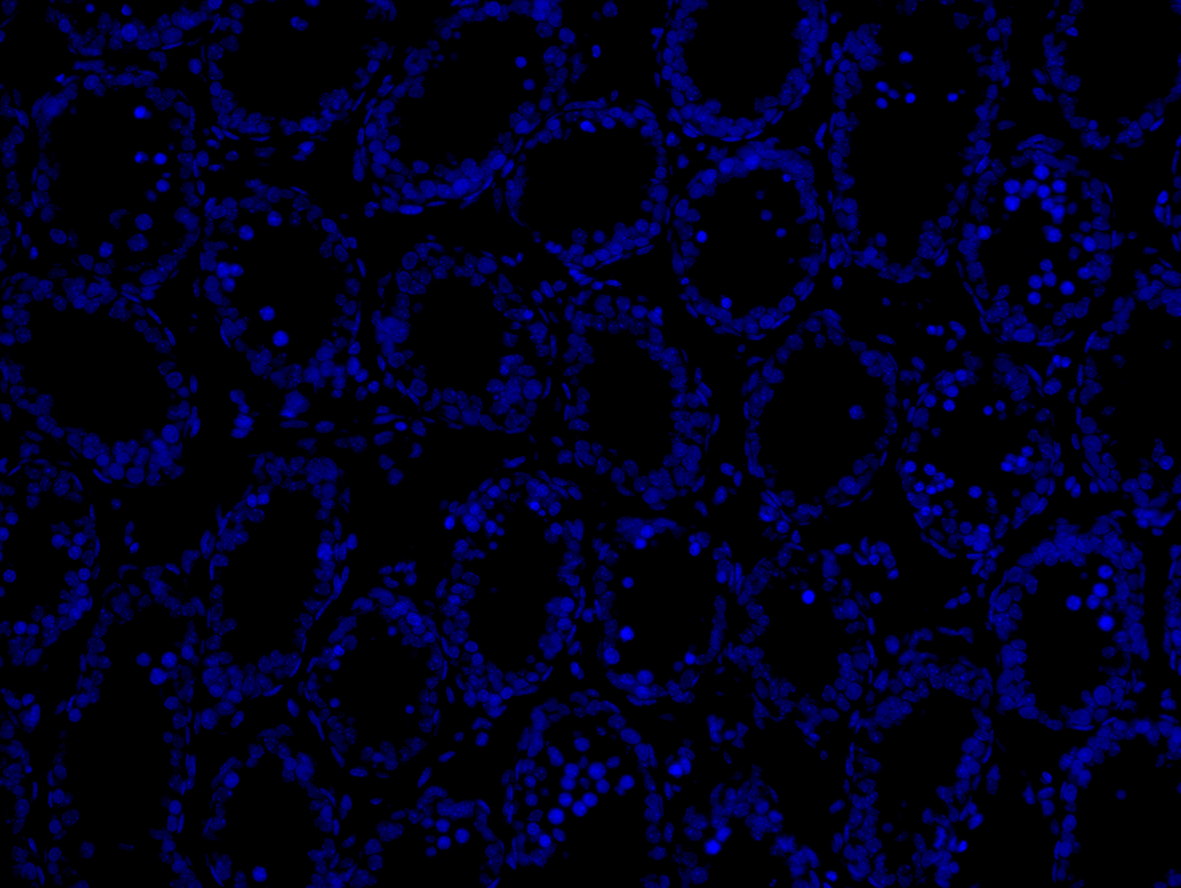

Supplement: Supplementary file 14 — EV and Appendix Figure Source Data [file 44318_2024_203_MOESM14_ESM.zip › Source Data for Expanded View and Appendix/Figure EV4/EV4G/Low magnification of P12 Kdm2a cKO - DAPI.jpg]

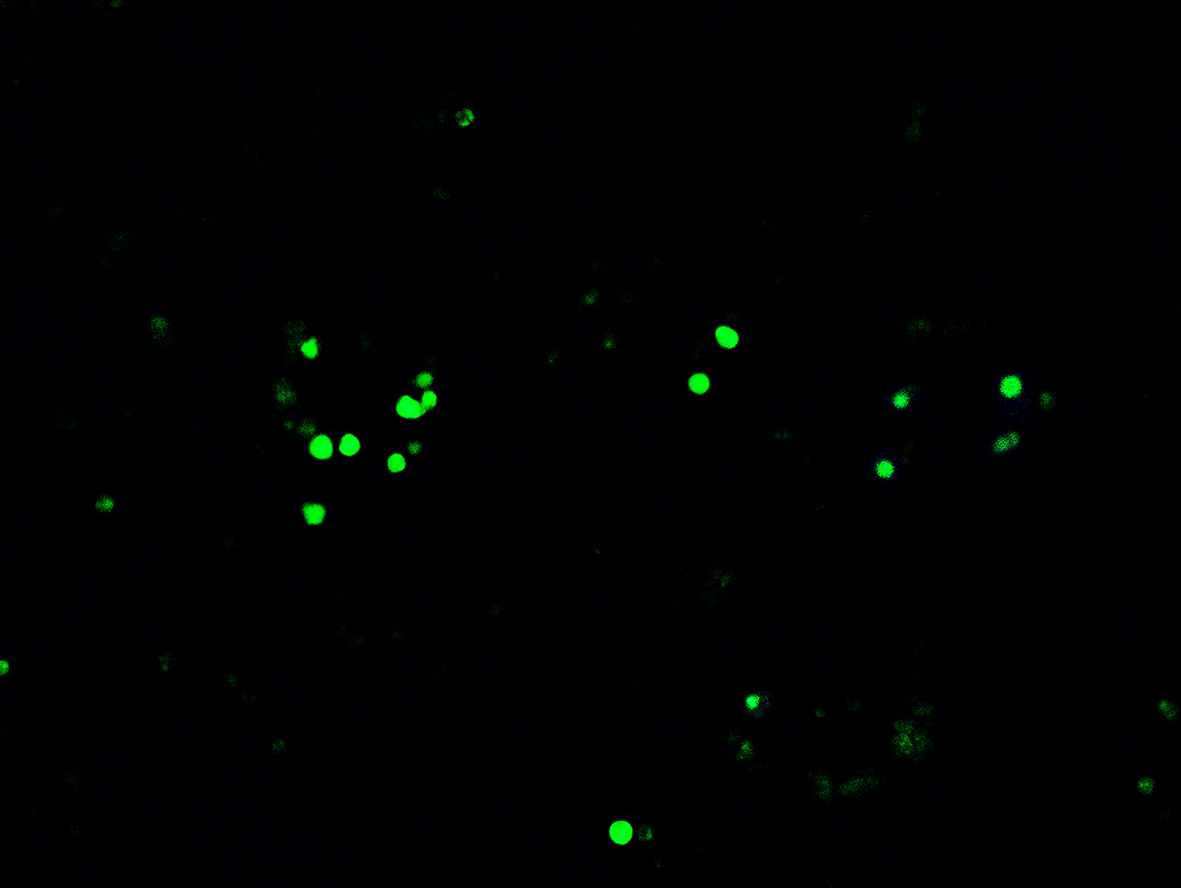

Supplement: Supplementary file 14 — EV and Appendix Figure Source Data [file 44318_2024_203_MOESM14_ESM.zip › Source Data for Expanded View and Appendix/Figure EV4/EV4G/High magnification of P10 Kdm2a cKO - Tunnel signal.jpg]

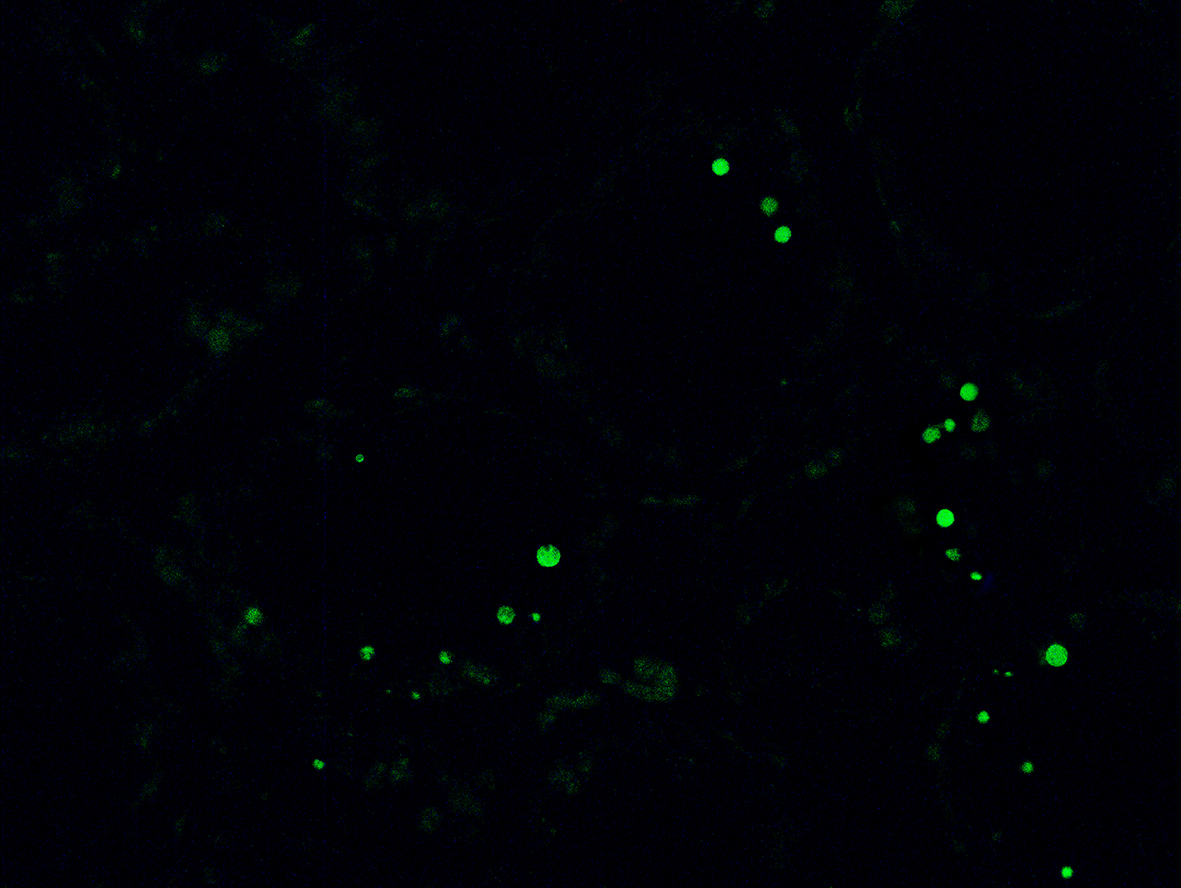

Supplement: Supplementary file 14 — EV and Appendix Figure Source Data [file 44318_2024_203_MOESM14_ESM.zip › Source Data for Expanded View and Appendix/Figure EV4/EV4G/High magnification of P14 cKO - Tunnel signal.jpg]

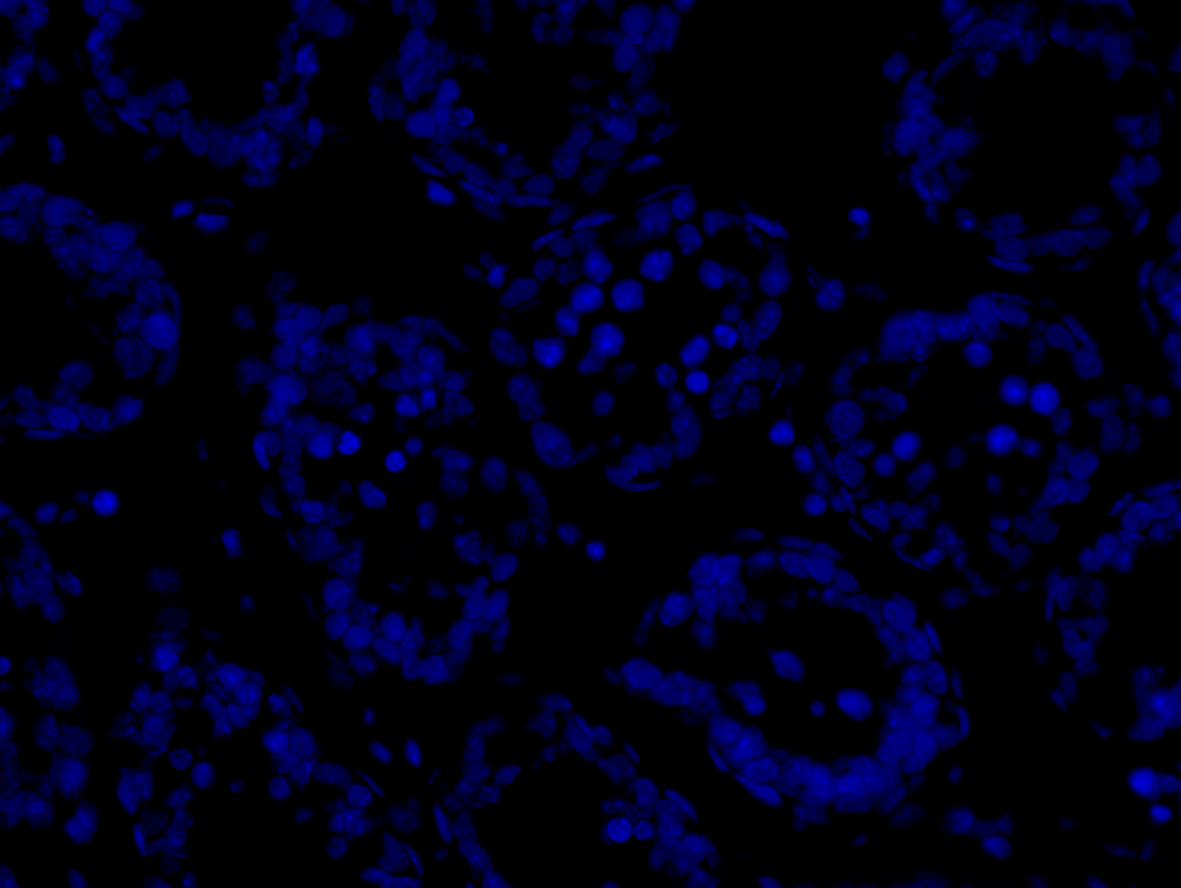

Supplement: Supplementary file 14 — EV and Appendix Figure Source Data [file 44318_2024_203_MOESM14_ESM.zip › Source Data for Expanded View and Appendix/Figure EV4/EV4G/High magnification of P10 Kdm2a cKO - DAPI.jpg]

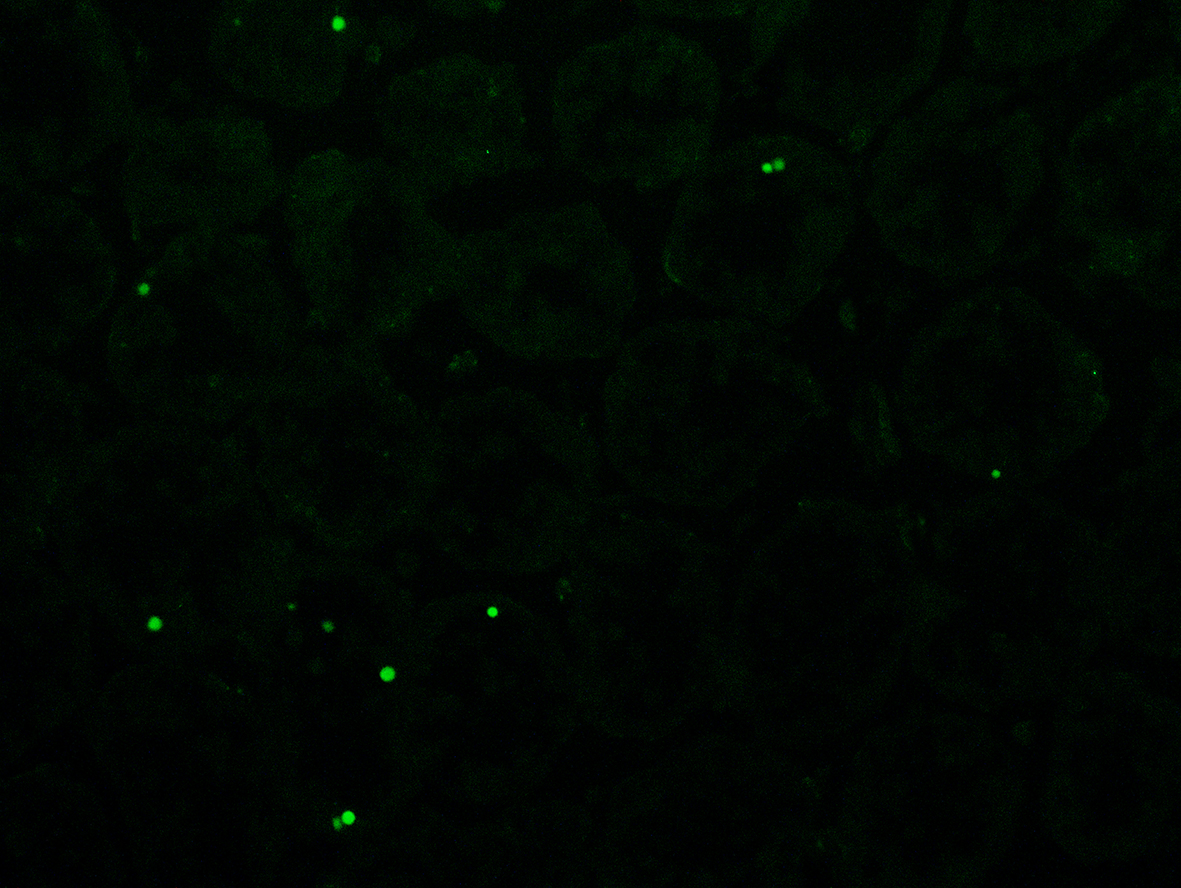

Supplement: Supplementary file 14 — EV and Appendix Figure Source Data [file 44318_2024_203_MOESM14_ESM.zip › Source Data for Expanded View and Appendix/Figure EV4/EV4G/Low magnification of P12 control - Tunnel signal.jpg]

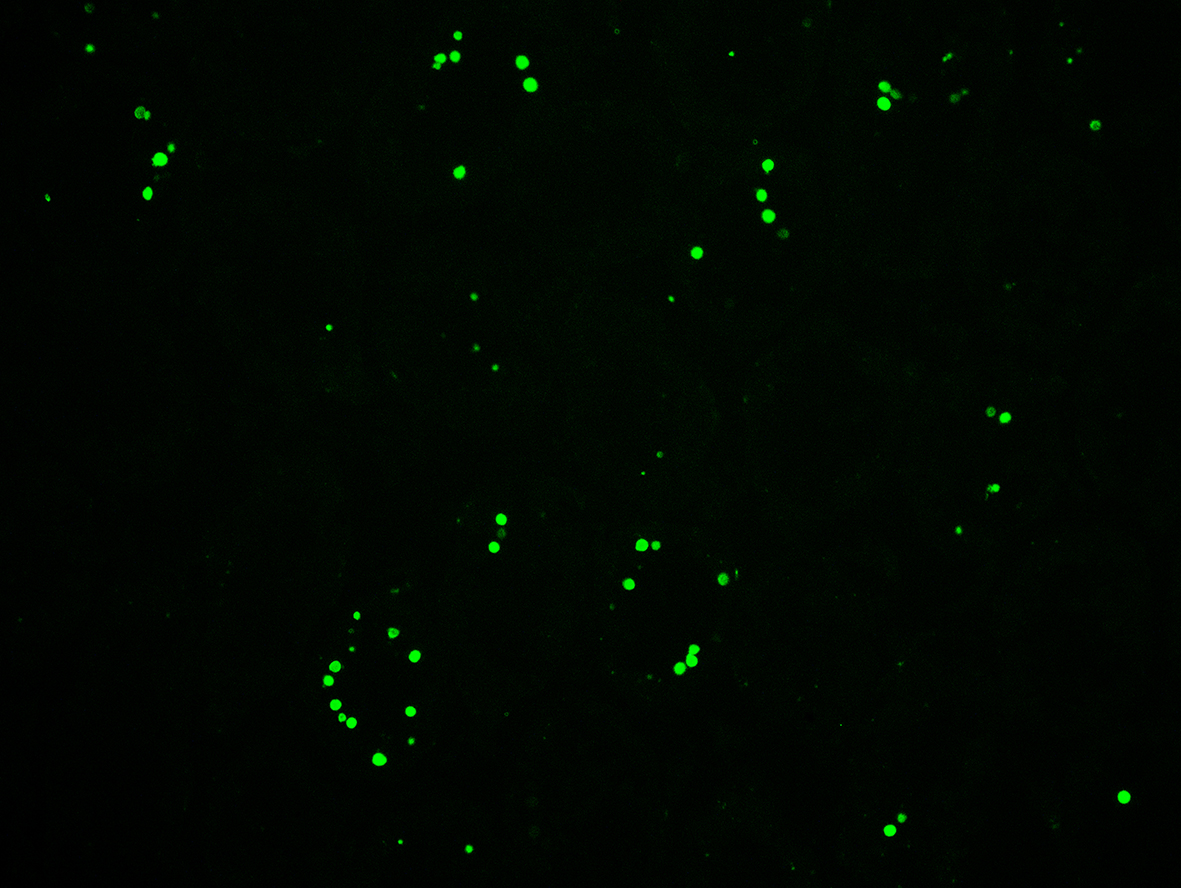

Supplement: Supplementary file 14 — EV and Appendix Figure Source Data [file 44318_2024_203_MOESM14_ESM.zip › Source Data for Expanded View and Appendix/Figure EV4/EV4G/Low magnification of P12 Kdm2a cKO - Tunnel signal.jpg]

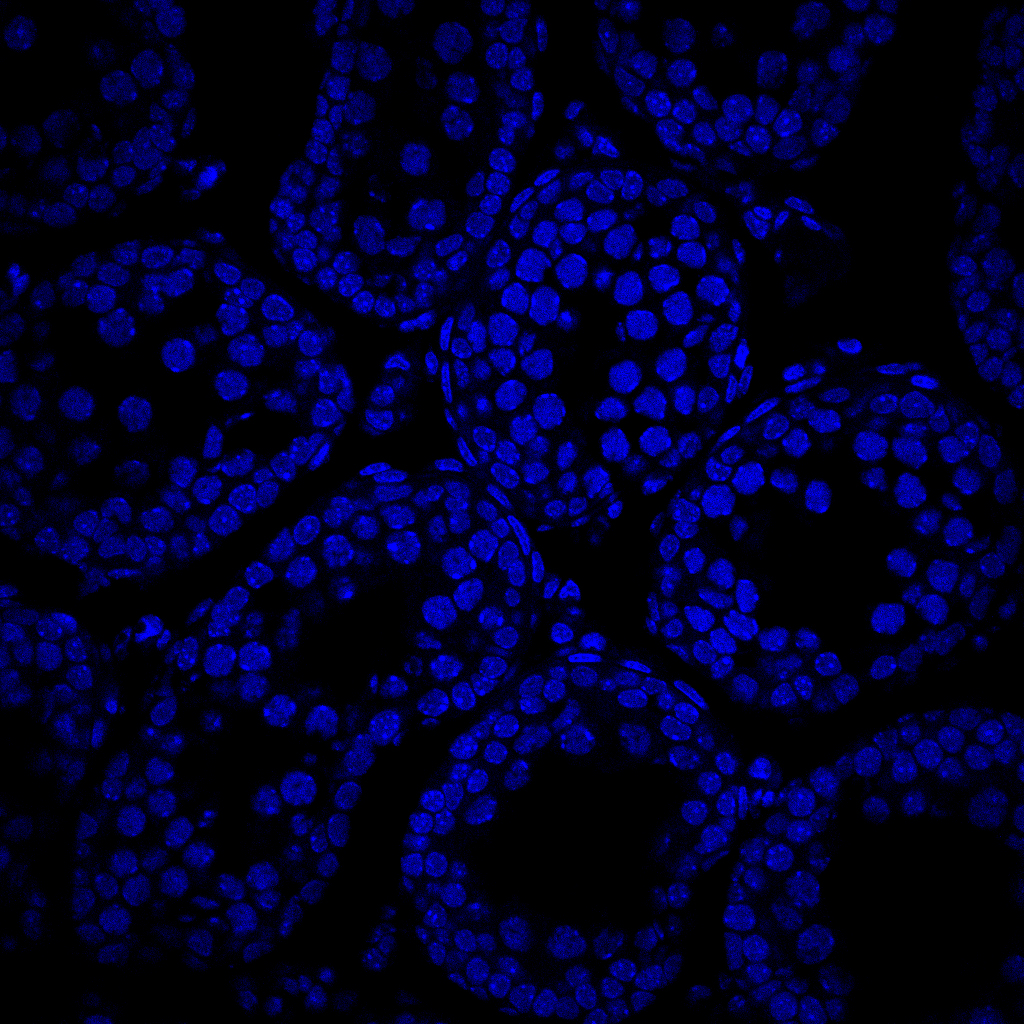

Supplement: Supplementary file 14 — EV and Appendix Figure Source Data [file 44318_2024_203_MOESM14_ESM.zip › Source Data for Expanded View and Appendix/Figure EV4/EV4A/Ctrl-DAPI.jpg]

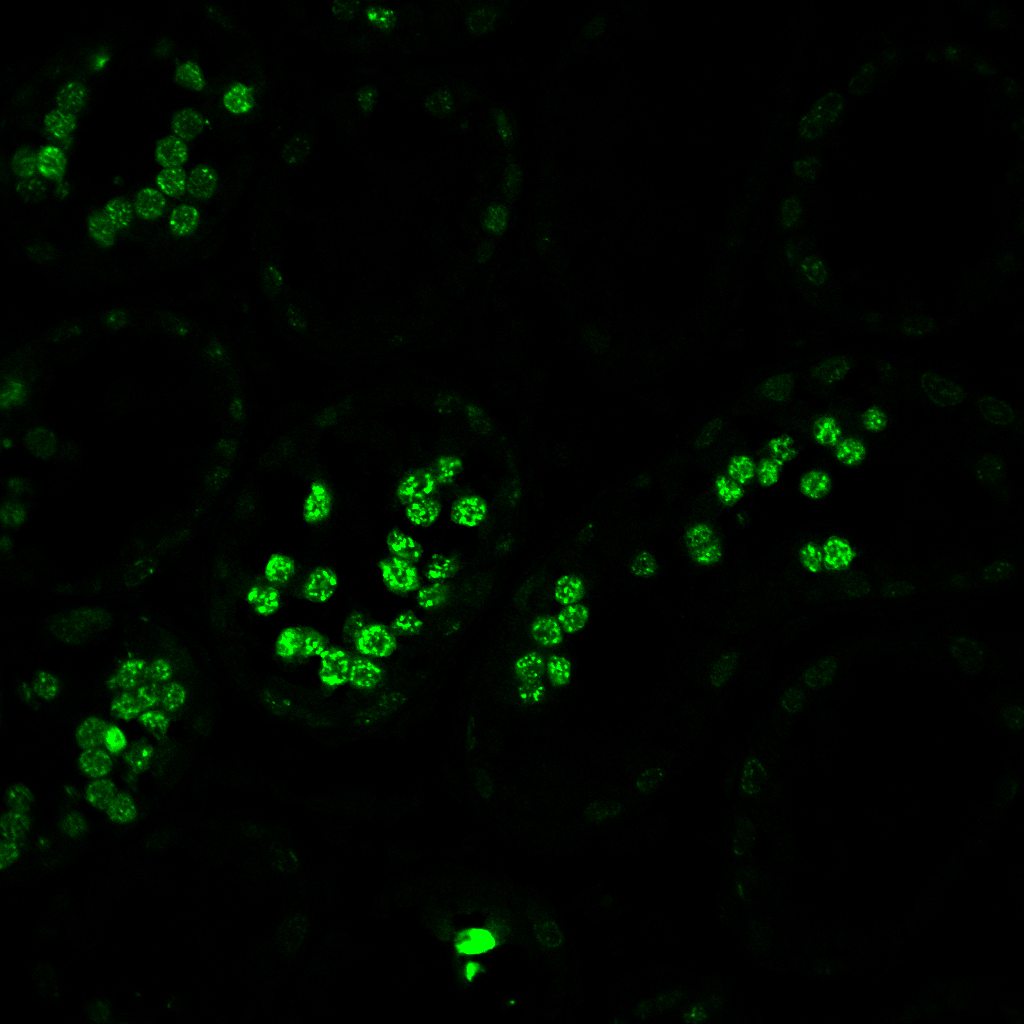

Supplement: Supplementary file 14 — EV and Appendix Figure Source Data [file 44318_2024_203_MOESM14_ESM.zip › Source Data for Expanded View and Appendix/Figure EV4/EV4A/cKO-╬│H2AX.jpg]

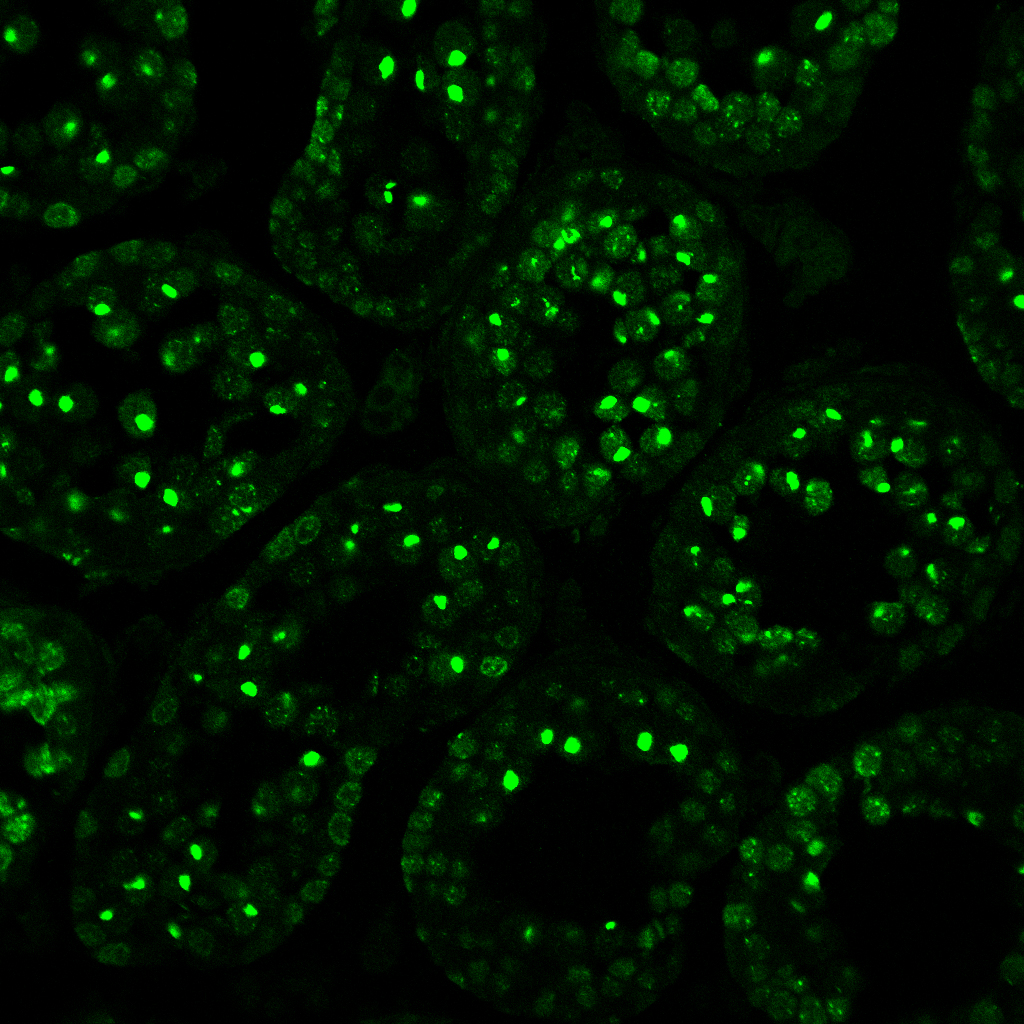

Supplement: Supplementary file 14 — EV and Appendix Figure Source Data [file 44318_2024_203_MOESM14_ESM.zip › Source Data for Expanded View and Appendix/Figure EV4/EV4A/Ctrl-╬│H2AX.jpg]

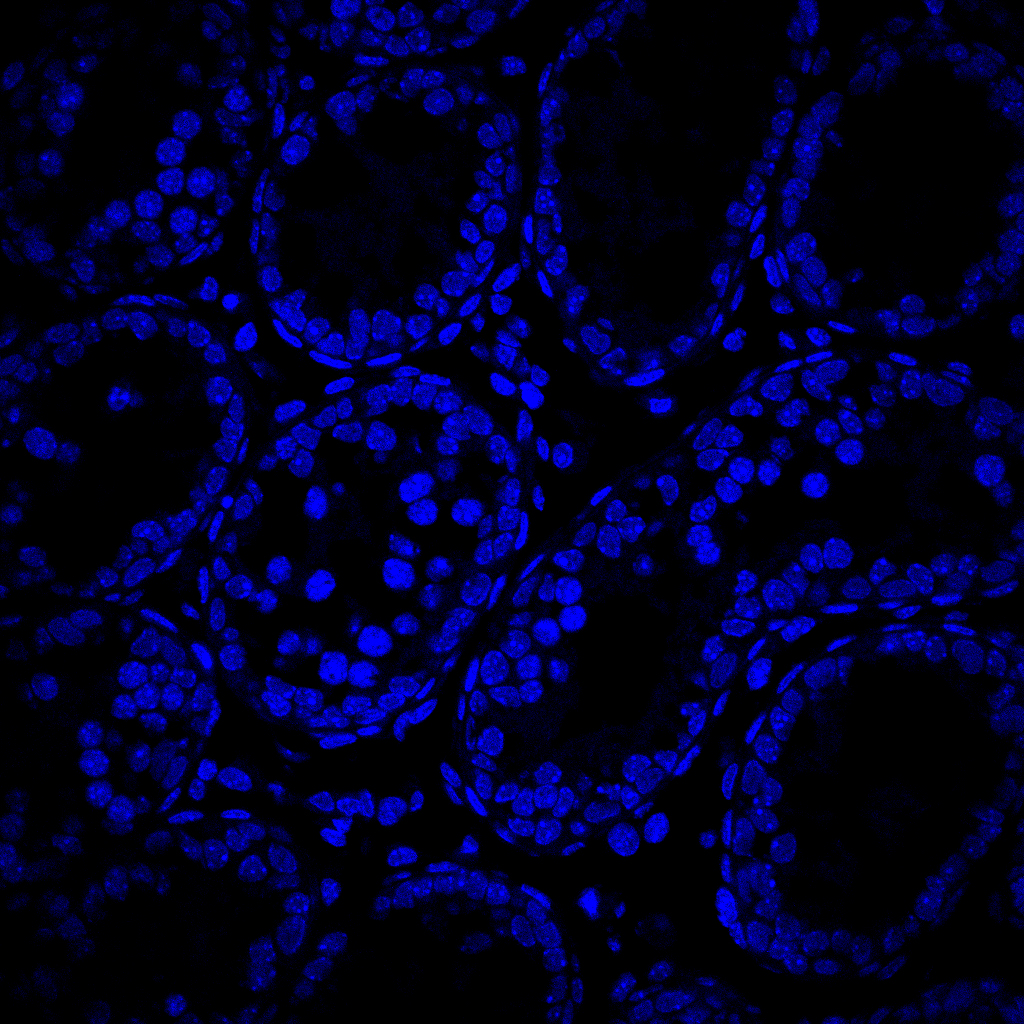

Supplement: Supplementary file 14 — EV and Appendix Figure Source Data [file 44318_2024_203_MOESM14_ESM.zip › Source Data for Expanded View and Appendix/Figure EV4/EV4A/cKO-DAPI.jpg]

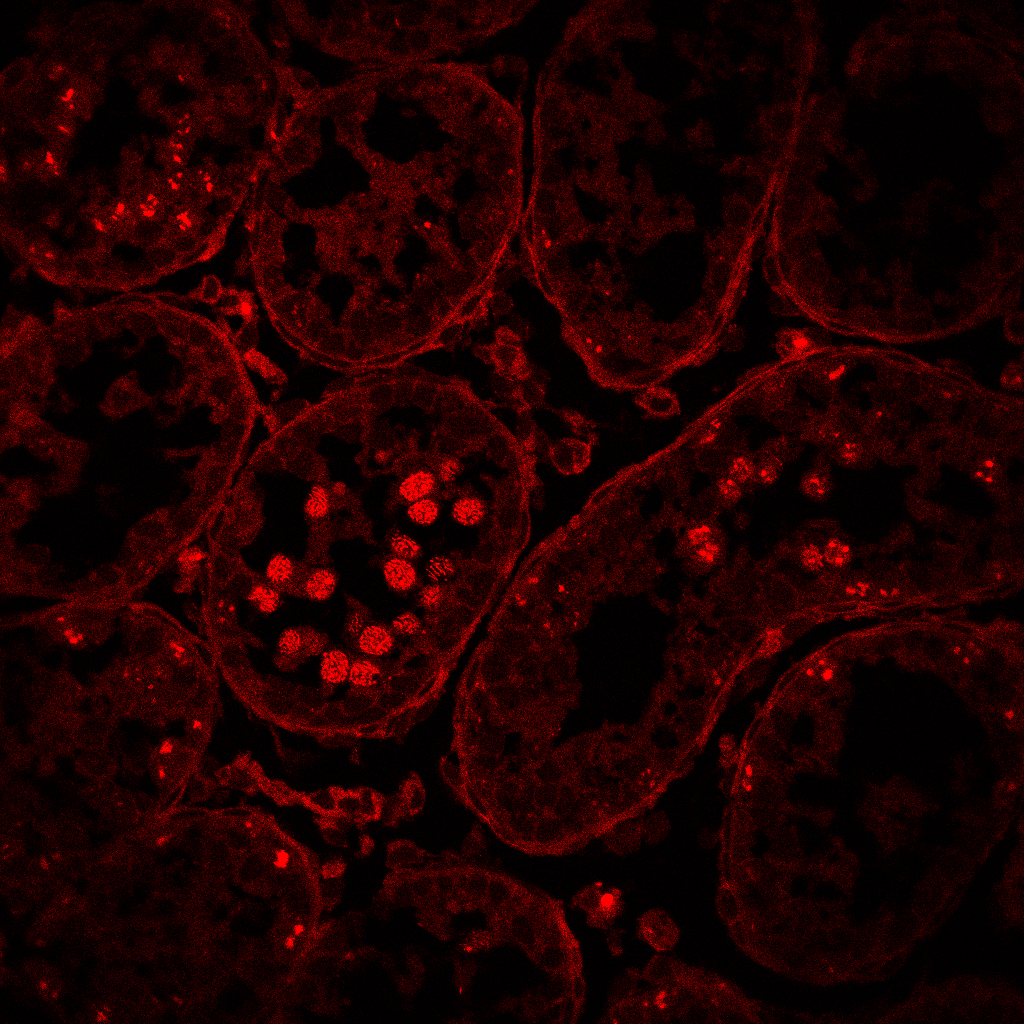

Supplement: Supplementary file 14 — EV and Appendix Figure Source Data [file 44318_2024_203_MOESM14_ESM.zip › Source Data for Expanded View and Appendix/Figure EV4/EV4A/cKO-SYCP3.jpg]

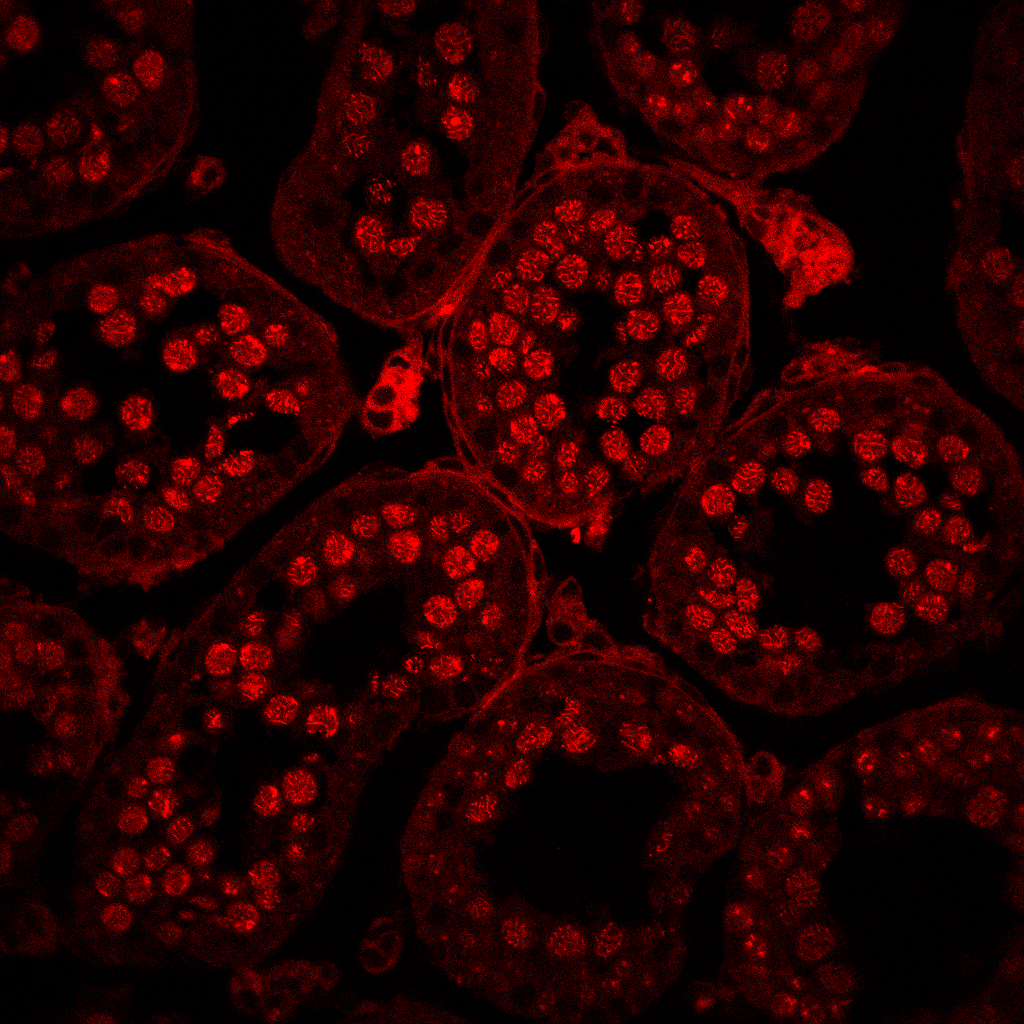

Supplement: Supplementary file 14 — EV and Appendix Figure Source Data [file 44318_2024_203_MOESM14_ESM.zip › Source Data for Expanded View and Appendix/Figure EV4/EV4A/Ctrl-SYCP3.jpg]

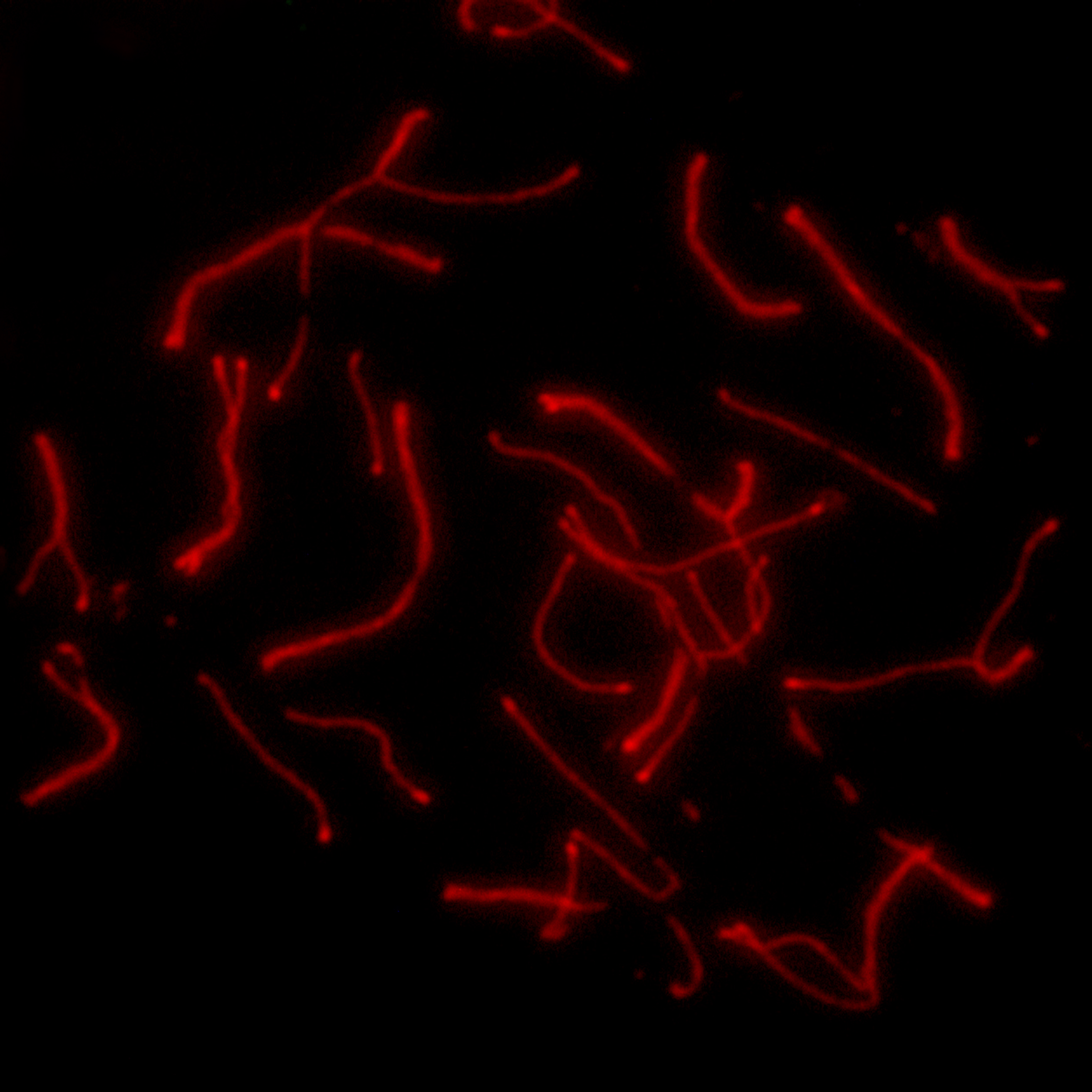

Supplement: Supplementary file 14 — EV and Appendix Figure Source Data [file 44318_2024_203_MOESM14_ESM.zip › Source Data for Expanded View and Appendix/Figure EV4/EV4E/Ctrl-Zyg-SYCP3.jpg]

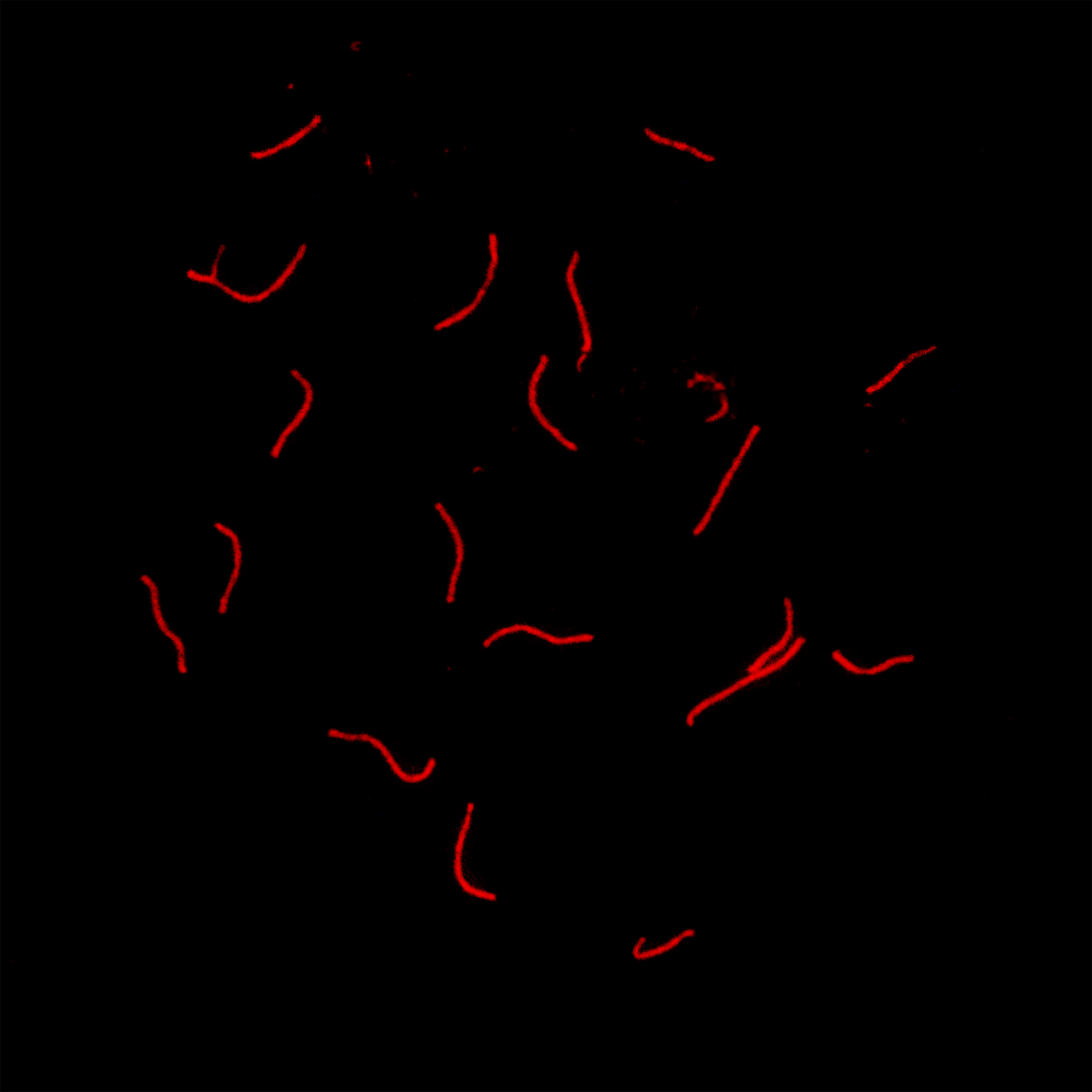

Supplement: Supplementary file 14 — EV and Appendix Figure Source Data [file 44318_2024_203_MOESM14_ESM.zip › Source Data for Expanded View and Appendix/Figure EV4/EV4E/Ctrl-Pac-SYCP3.jpg]

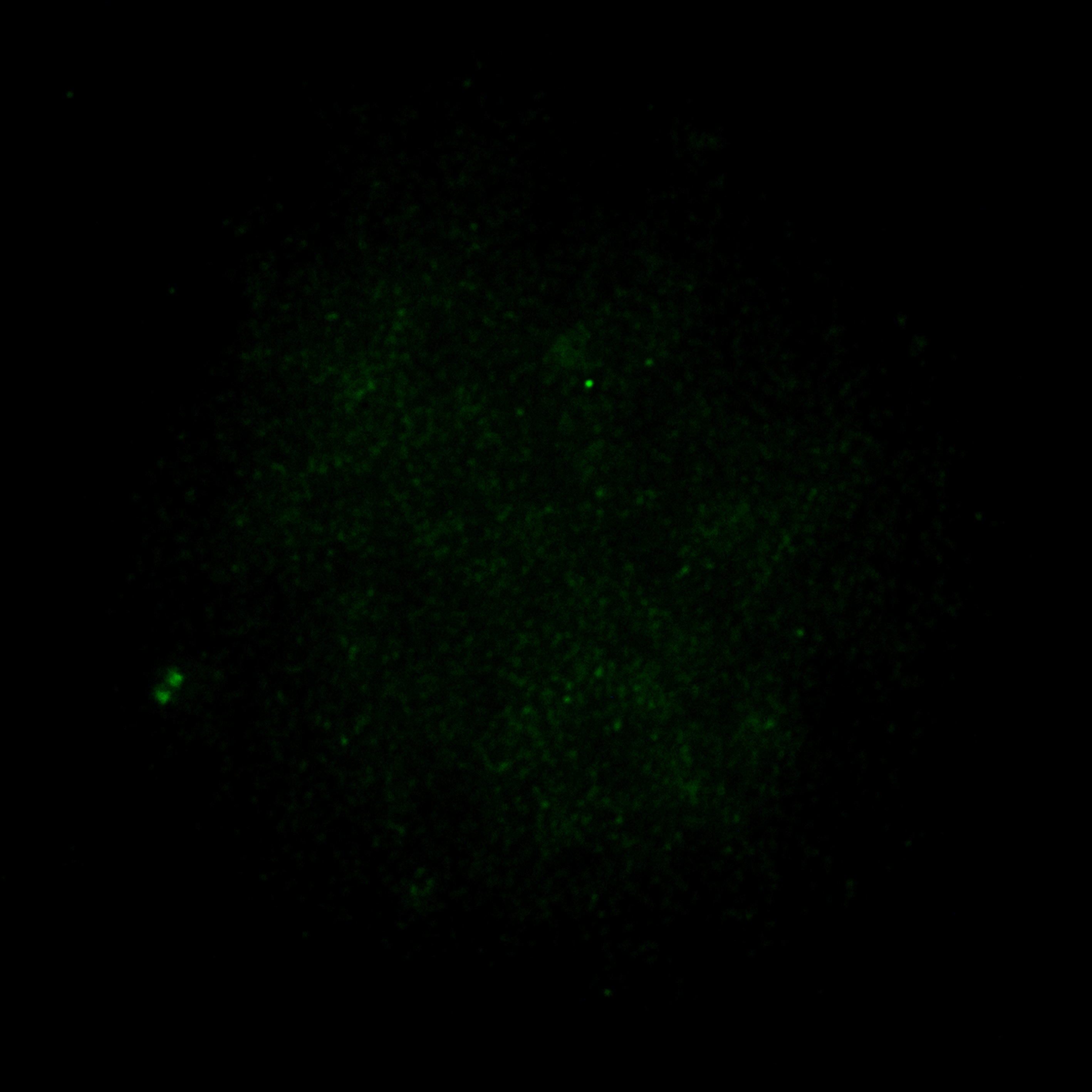

Supplement: Supplementary file 14 — EV and Appendix Figure Source Data [file 44318_2024_203_MOESM14_ESM.zip › Source Data for Expanded View and Appendix/Figure EV4/EV4E/cKO-Zyg-i-DMC1.jpg]

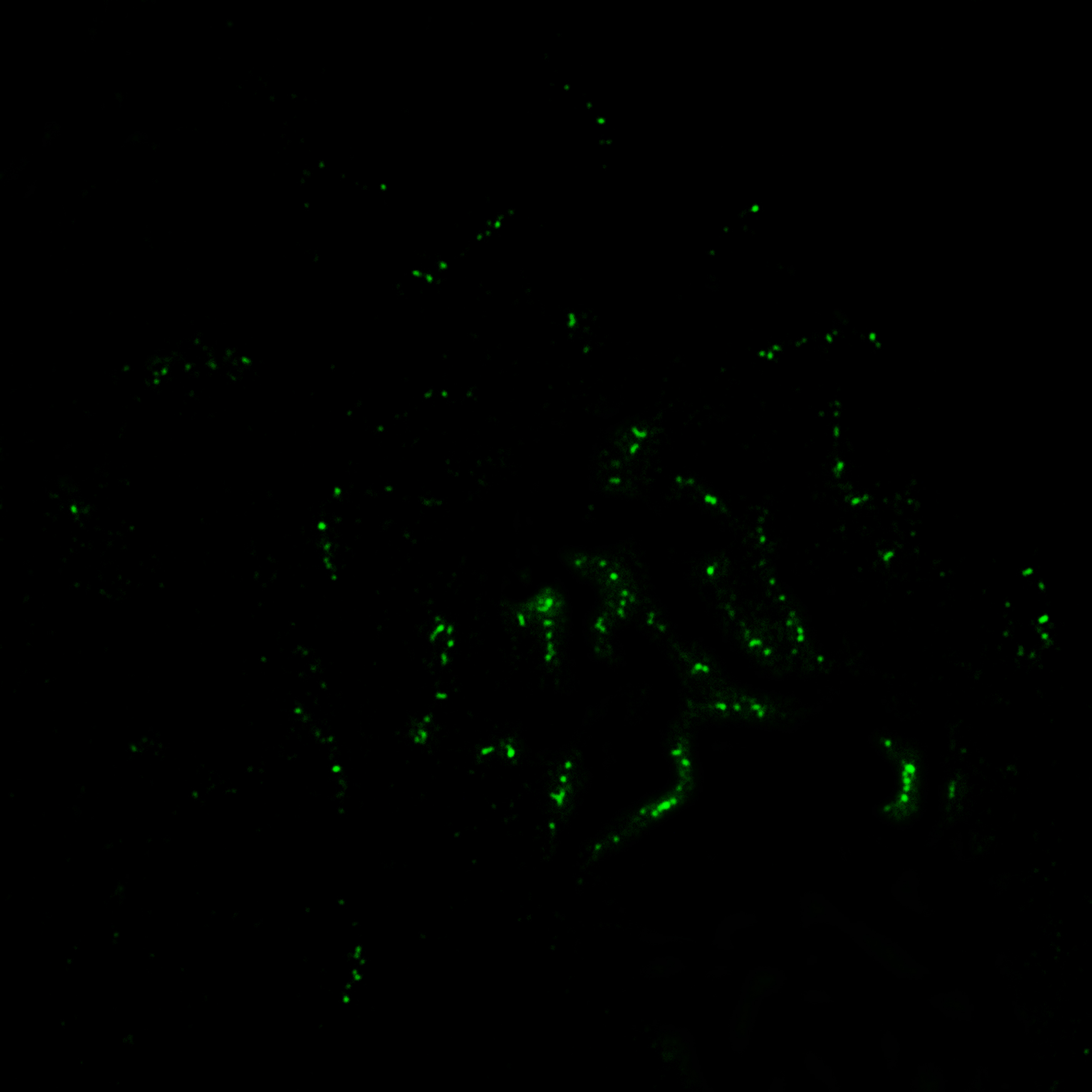

Supplement: Supplementary file 14 — EV and Appendix Figure Source Data [file 44318_2024_203_MOESM14_ESM.zip › Source Data for Expanded View and Appendix/Figure EV4/EV4E/cKO-Zyg-ii-DMC1.jpg]

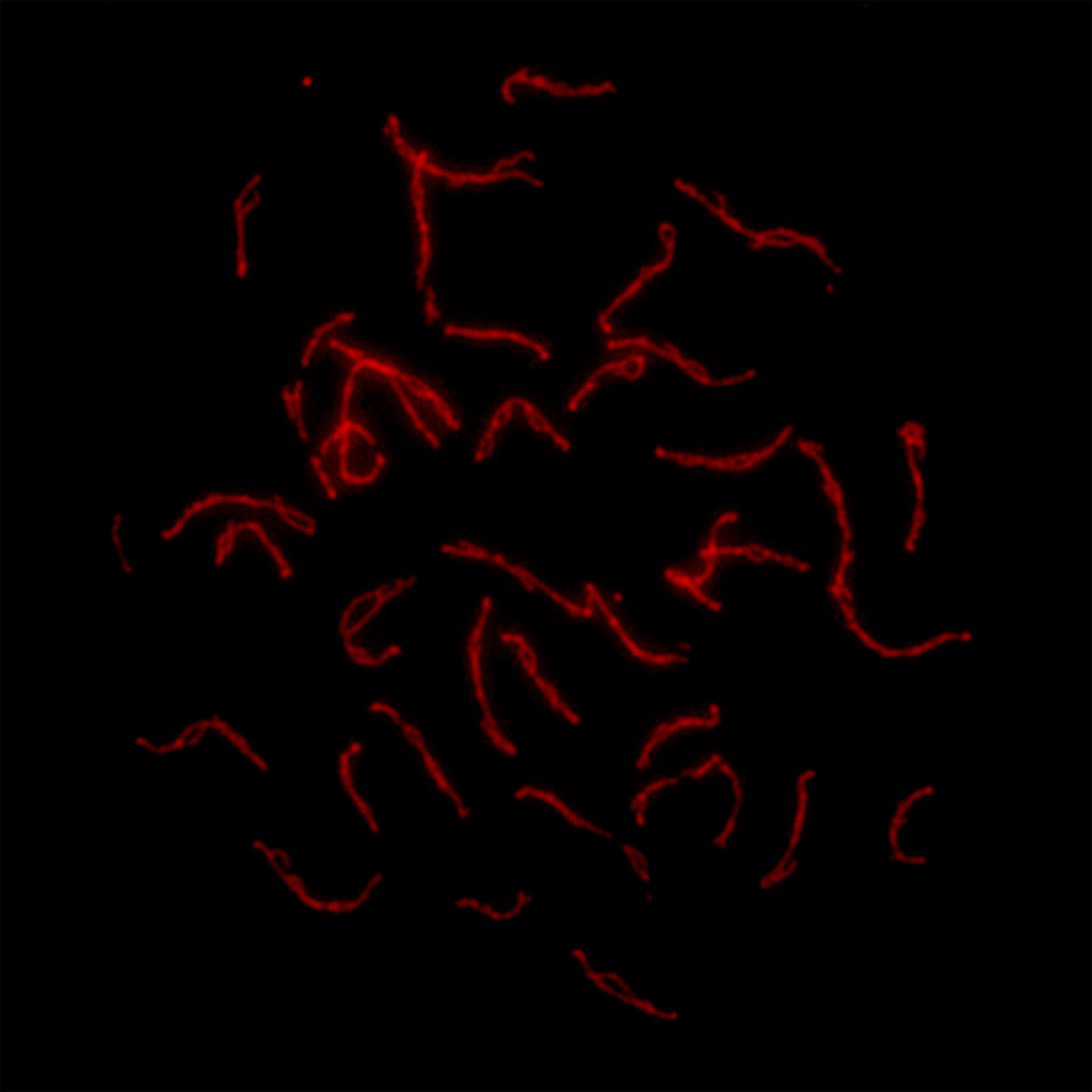

Supplement: Supplementary file 14 — EV and Appendix Figure Source Data [file 44318_2024_203_MOESM14_ESM.zip › Source Data for Expanded View and Appendix/Figure EV4/EV4E/cKO-Zyg-i-SYCP3.jpg]

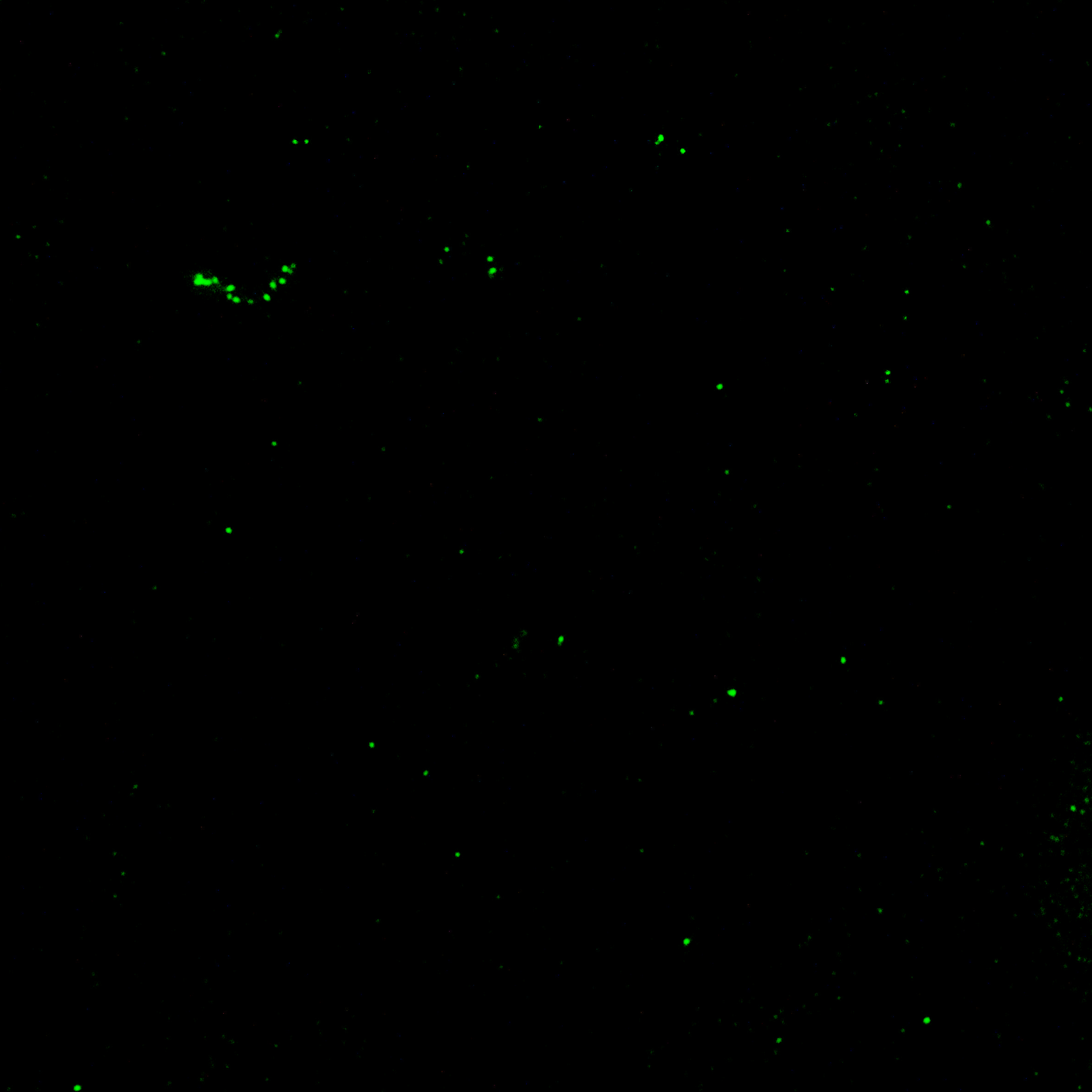

Supplement: Supplementary file 14 — EV and Appendix Figure Source Data [file 44318_2024_203_MOESM14_ESM.zip › Source Data for Expanded View and Appendix/Figure EV4/EV4E/Ctrl-Pac-DMC1.jpg]

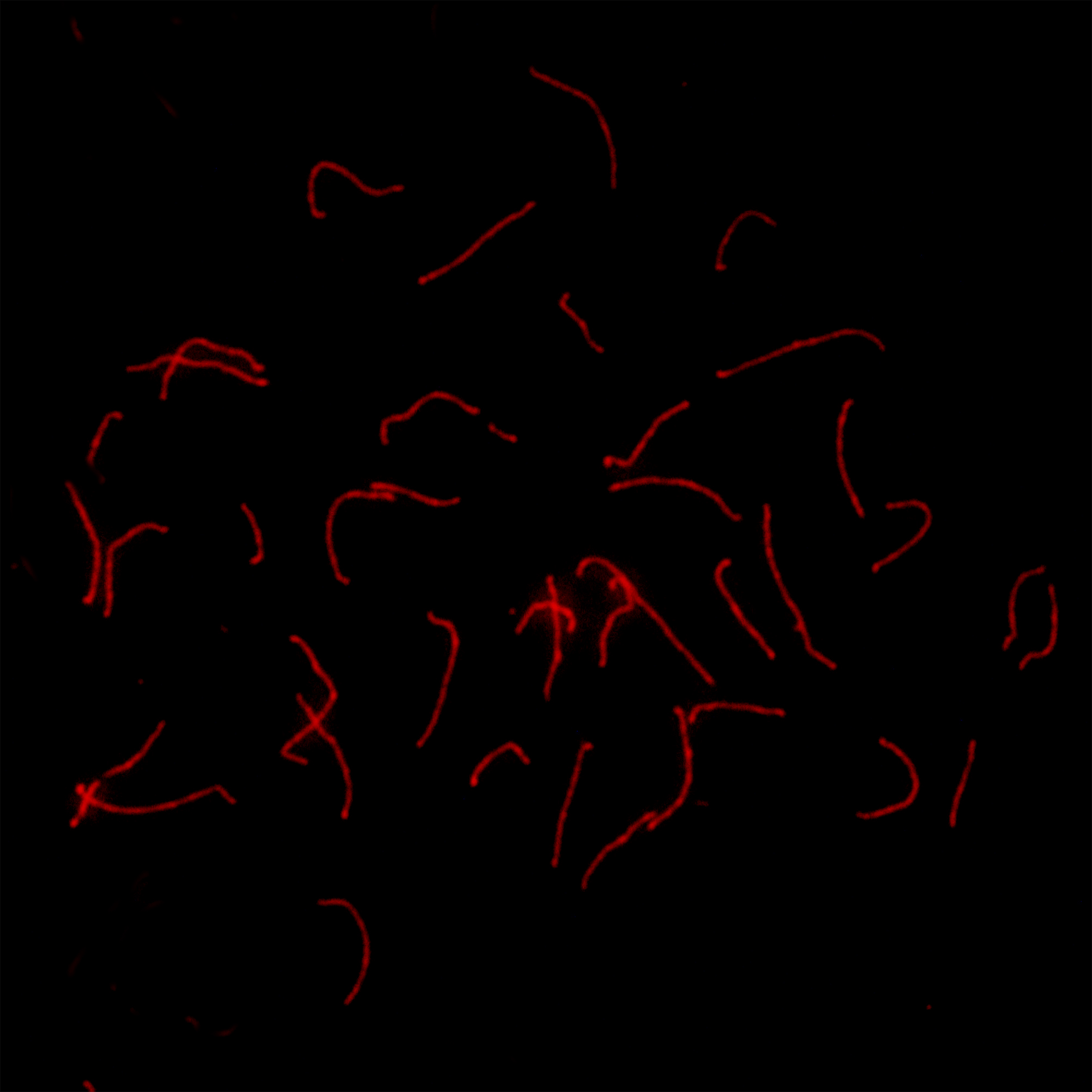

Supplement: Supplementary file 14 — EV and Appendix Figure Source Data [file 44318_2024_203_MOESM14_ESM.zip › Source Data for Expanded View and Appendix/Figure EV4/EV4E/cKO-Zyg-ii-SYCP3.jpg]

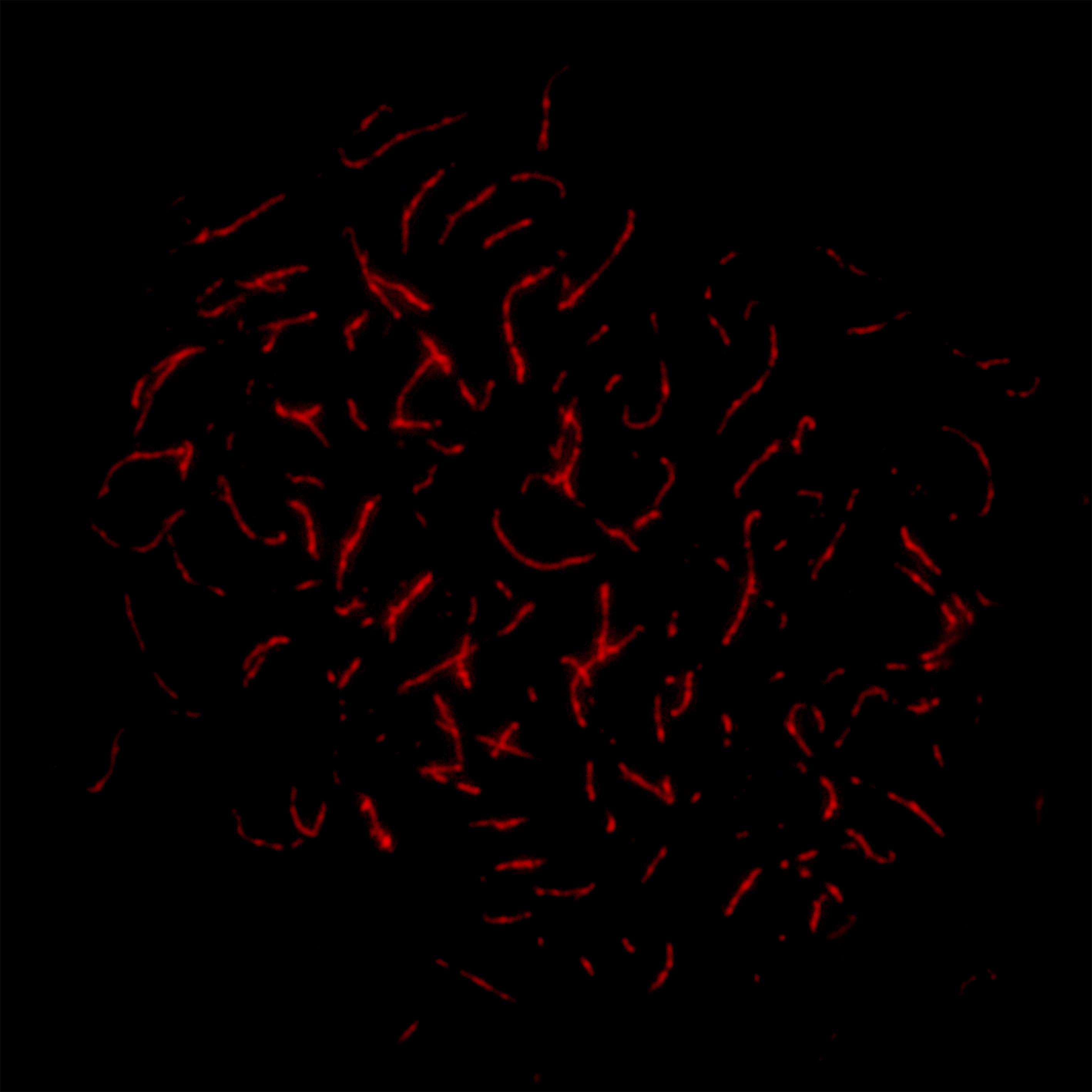

Supplement: Supplementary file 14 — EV and Appendix Figure Source Data [file 44318_2024_203_MOESM14_ESM.zip › Source Data for Expanded View and Appendix/Figure EV4/EV4E/cKO-Lep-sycp3.jpg]

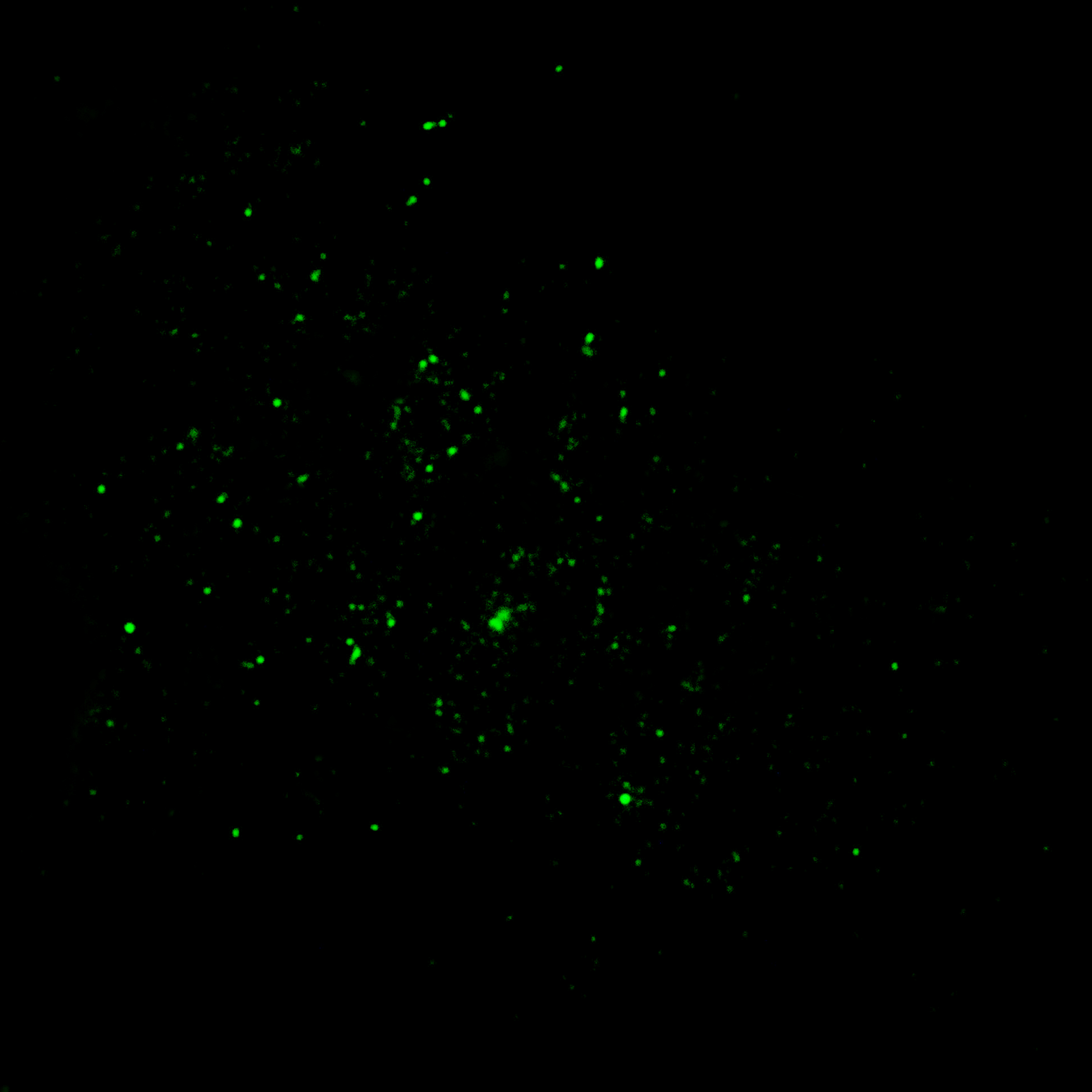

Supplement: Supplementary file 14 — EV and Appendix Figure Source Data [file 44318_2024_203_MOESM14_ESM.zip › Source Data for Expanded View and Appendix/Figure EV4/EV4E/cKO-Lep-DMC1.jpg]

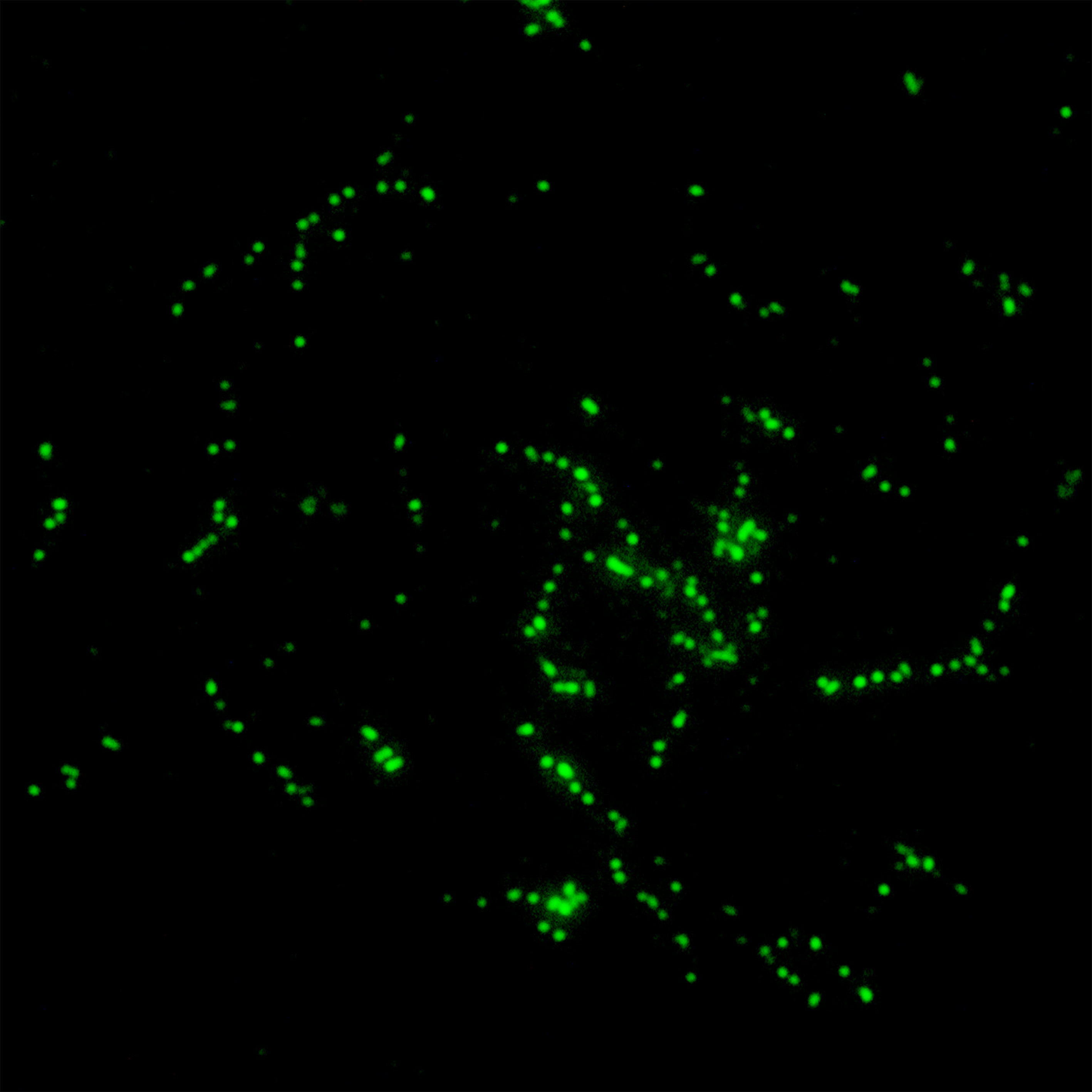

Supplement: Supplementary file 14 — EV and Appendix Figure Source Data [file 44318_2024_203_MOESM14_ESM.zip › Source Data for Expanded View and Appendix/Figure EV4/EV4E/Ctrl-Zyg-DMC1.jpg]

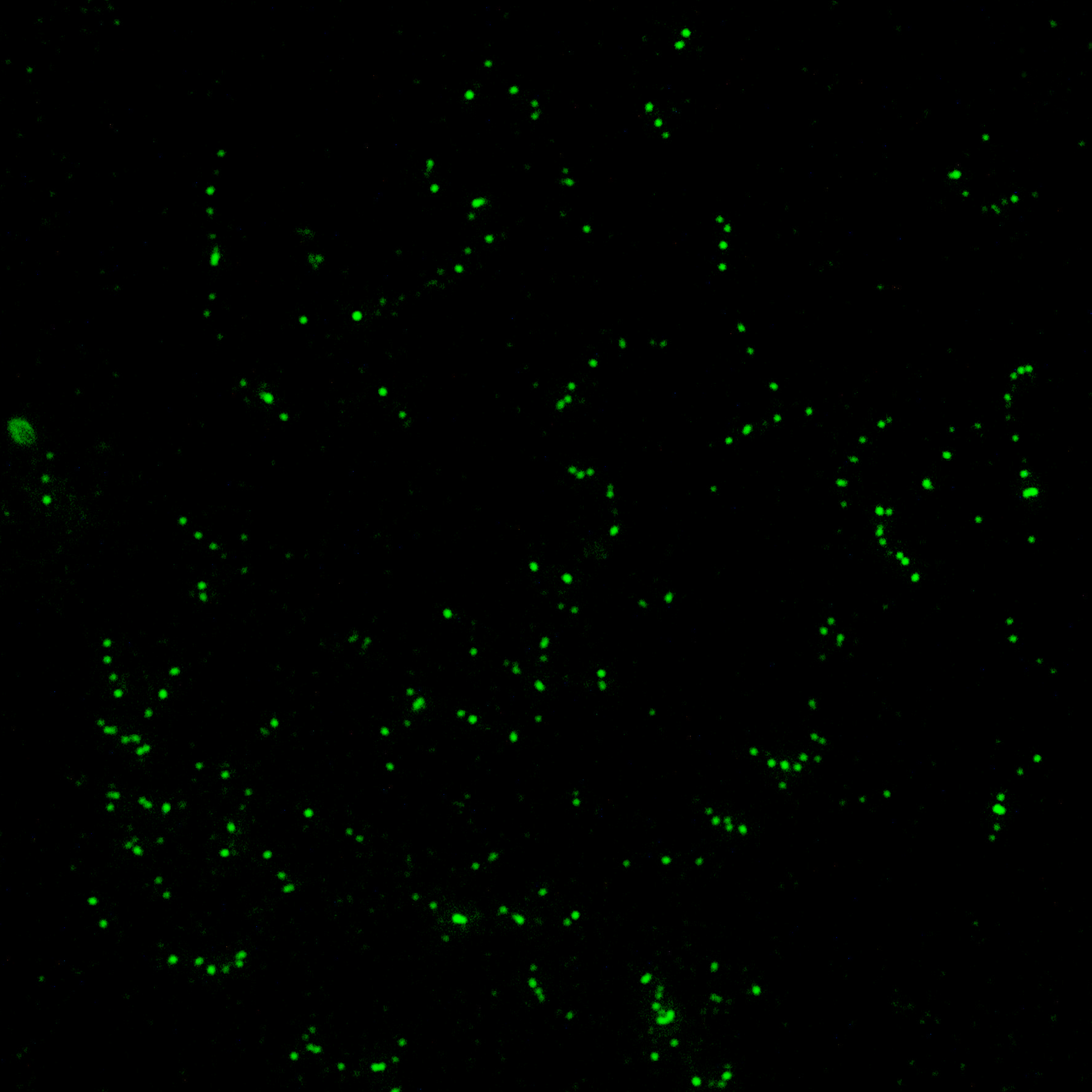

Supplement: Supplementary file 14 — EV and Appendix Figure Source Data [file 44318_2024_203_MOESM14_ESM.zip › Source Data for Expanded View and Appendix/Figure EV4/EV4E/Ctrl-Lep-DMC1.jpg]

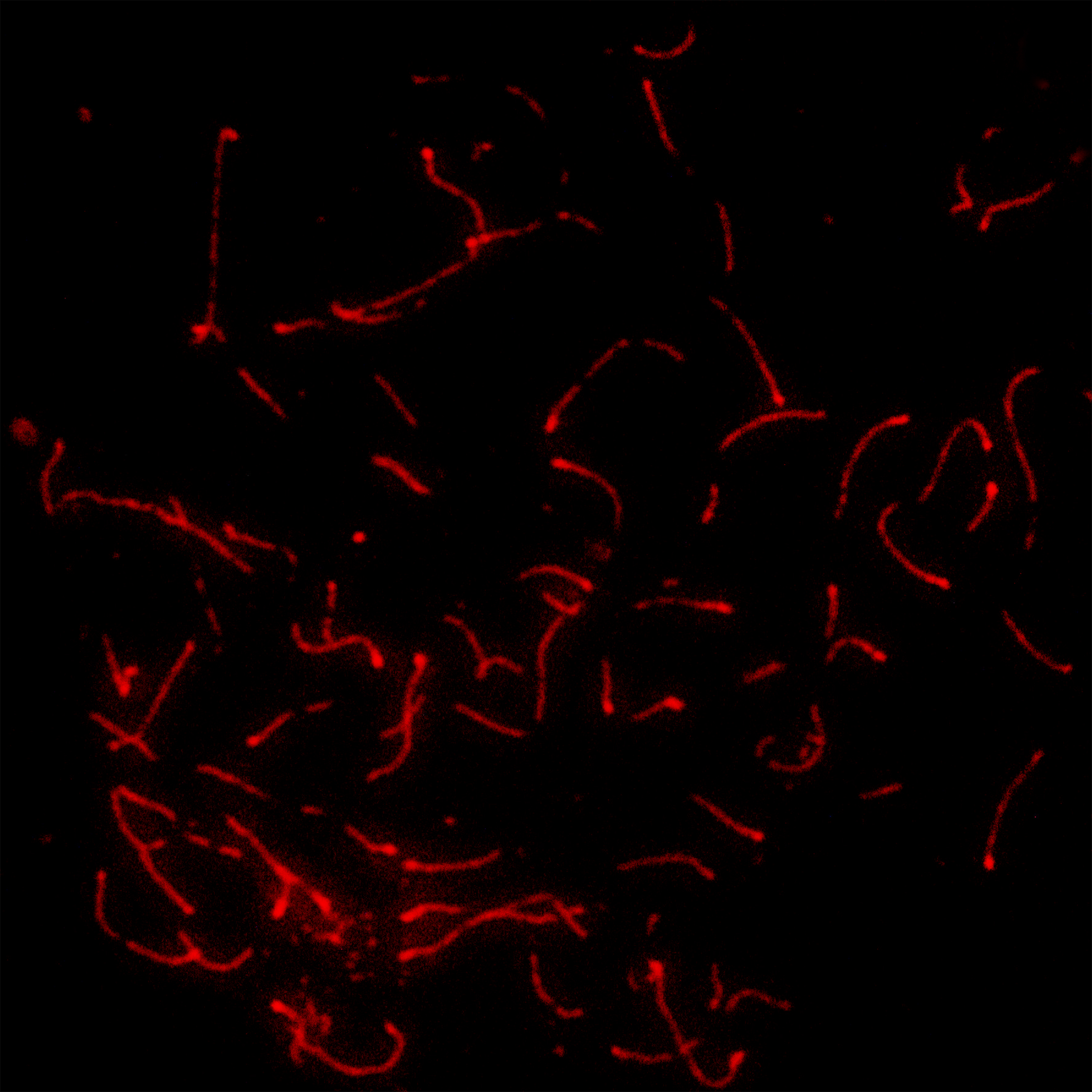

Supplement: Supplementary file 14 — EV and Appendix Figure Source Data [file 44318_2024_203_MOESM14_ESM.zip › Source Data for Expanded View and Appendix/Figure EV4/EV4E/Ctrl-Lep-SYCP3.jpg]

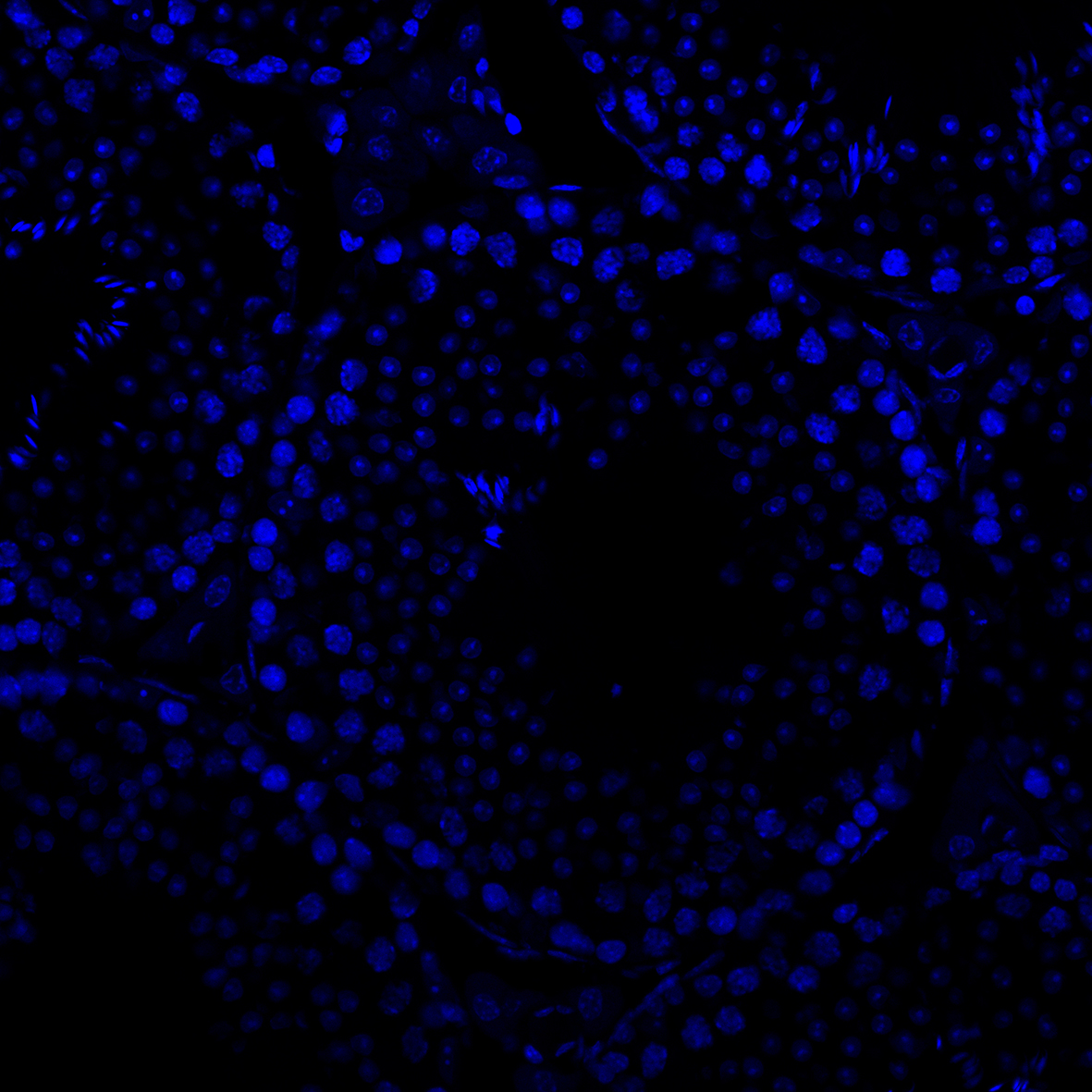

Supplement: Supplementary file 14 — EV and Appendix Figure Source Data [file 44318_2024_203_MOESM14_ESM.zip › Source Data for Expanded View and Appendix/Figure EV3/EV3H/Ctrl-DAPI.jpg]

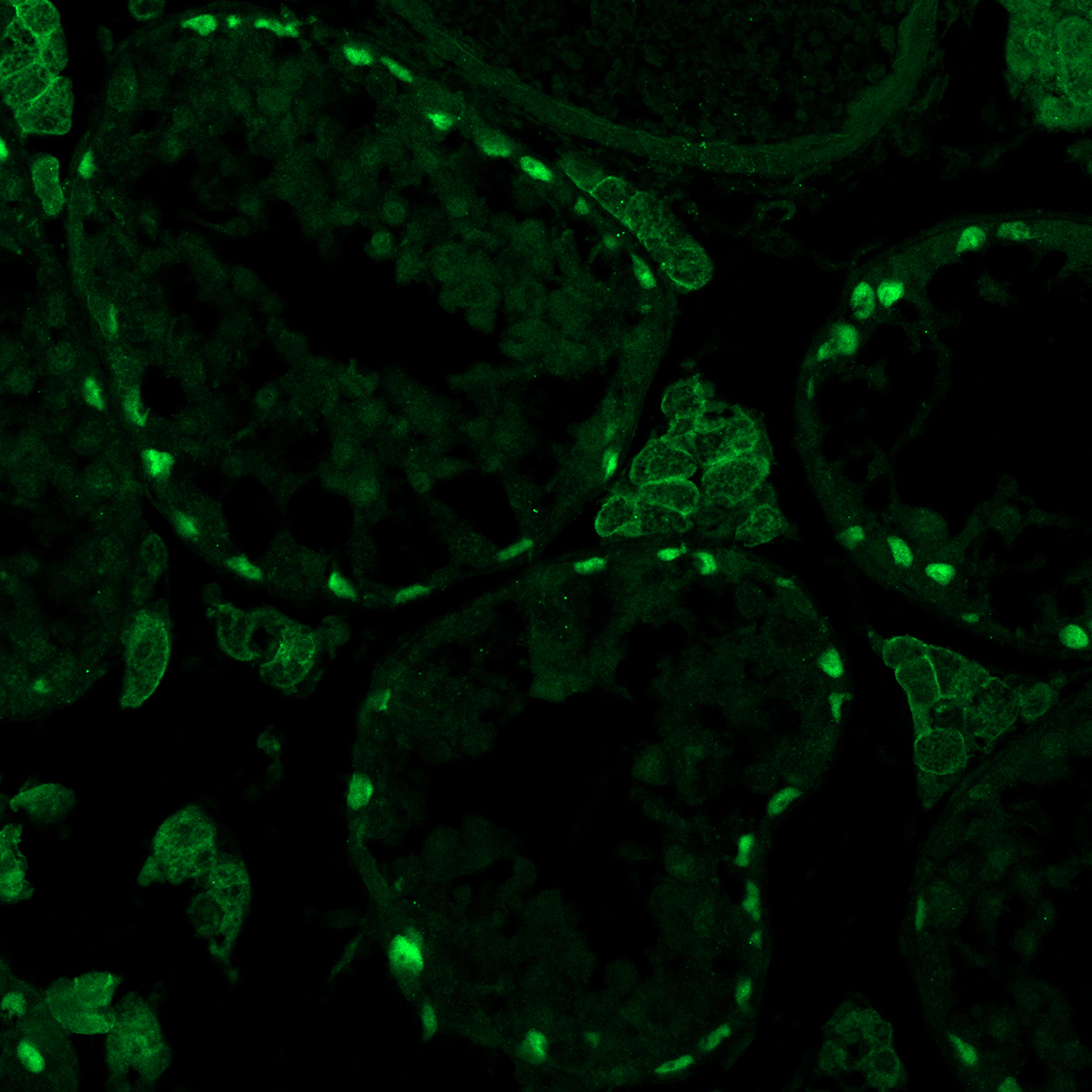

Supplement: Supplementary file 14 — EV and Appendix Figure Source Data [file 44318_2024_203_MOESM14_ESM.zip › Source Data for Expanded View and Appendix/Figure EV3/EV3H/cKO-WT1.jpg]

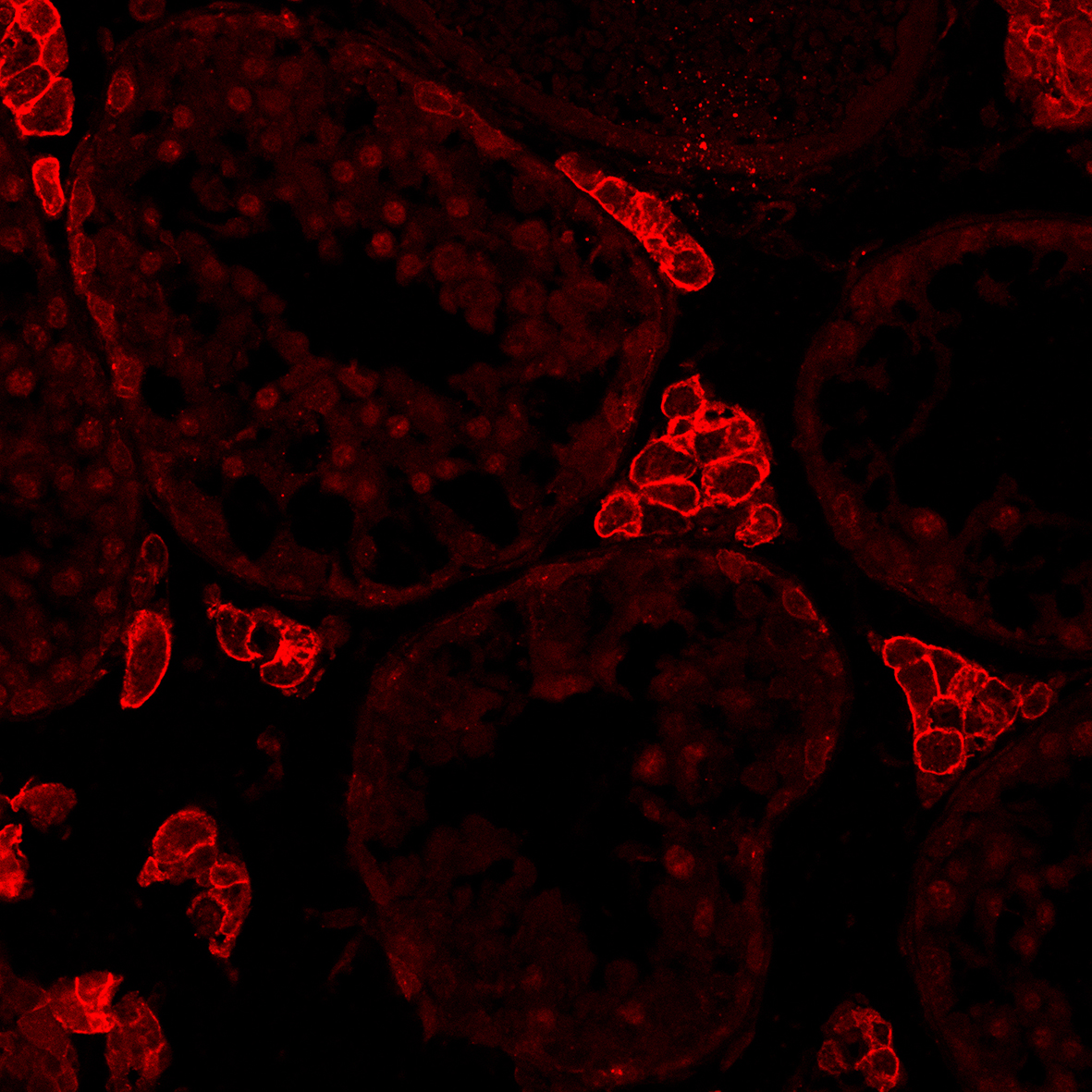

Supplement: Supplementary file 14 — EV and Appendix Figure Source Data [file 44318_2024_203_MOESM14_ESM.zip › Source Data for Expanded View and Appendix/Figure EV3/EV3H/cKO-cKIT.jpg]

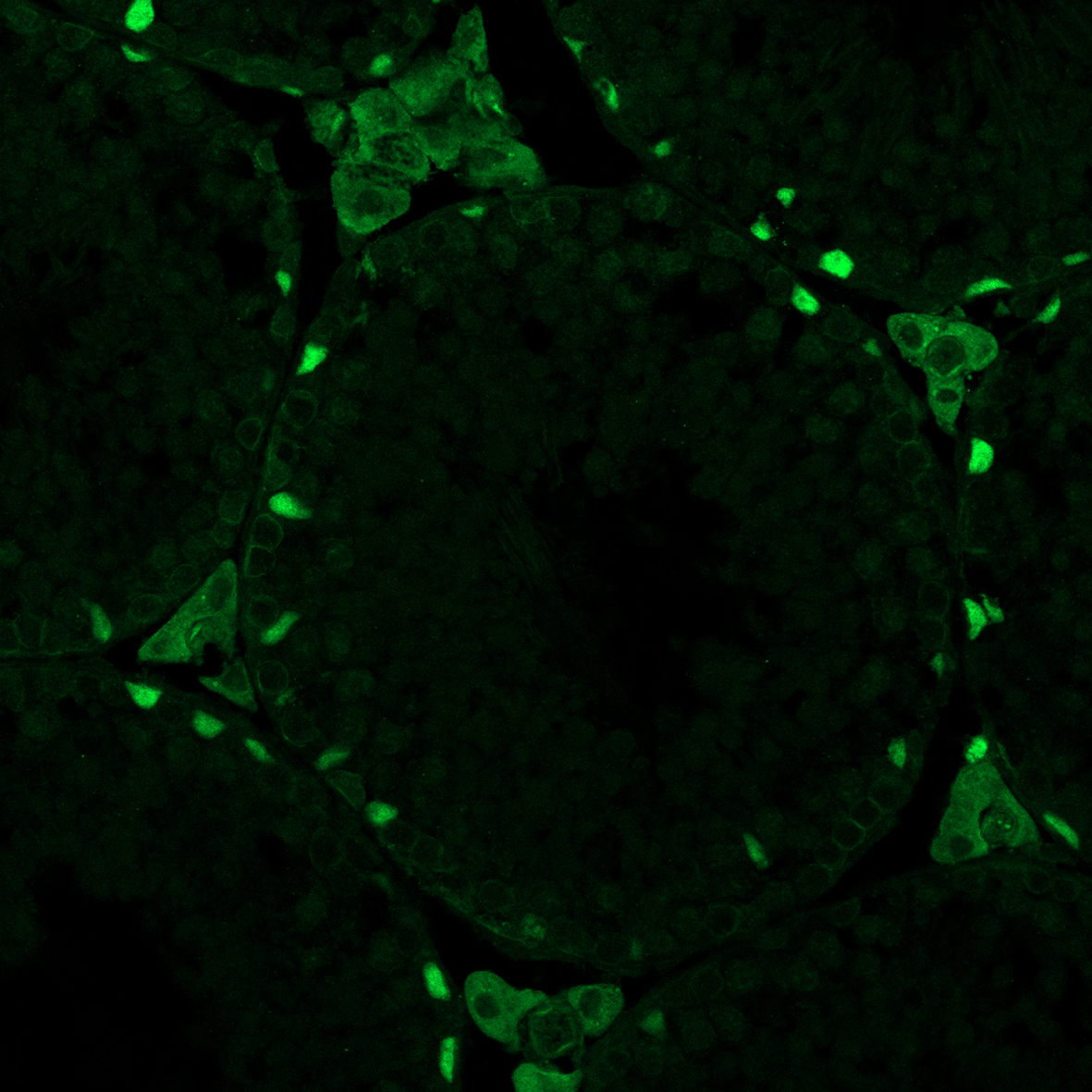

Supplement: Supplementary file 14 — EV and Appendix Figure Source Data [file 44318_2024_203_MOESM14_ESM.zip › Source Data for Expanded View and Appendix/Figure EV3/EV3H/Ctrl-WT1.jpg]

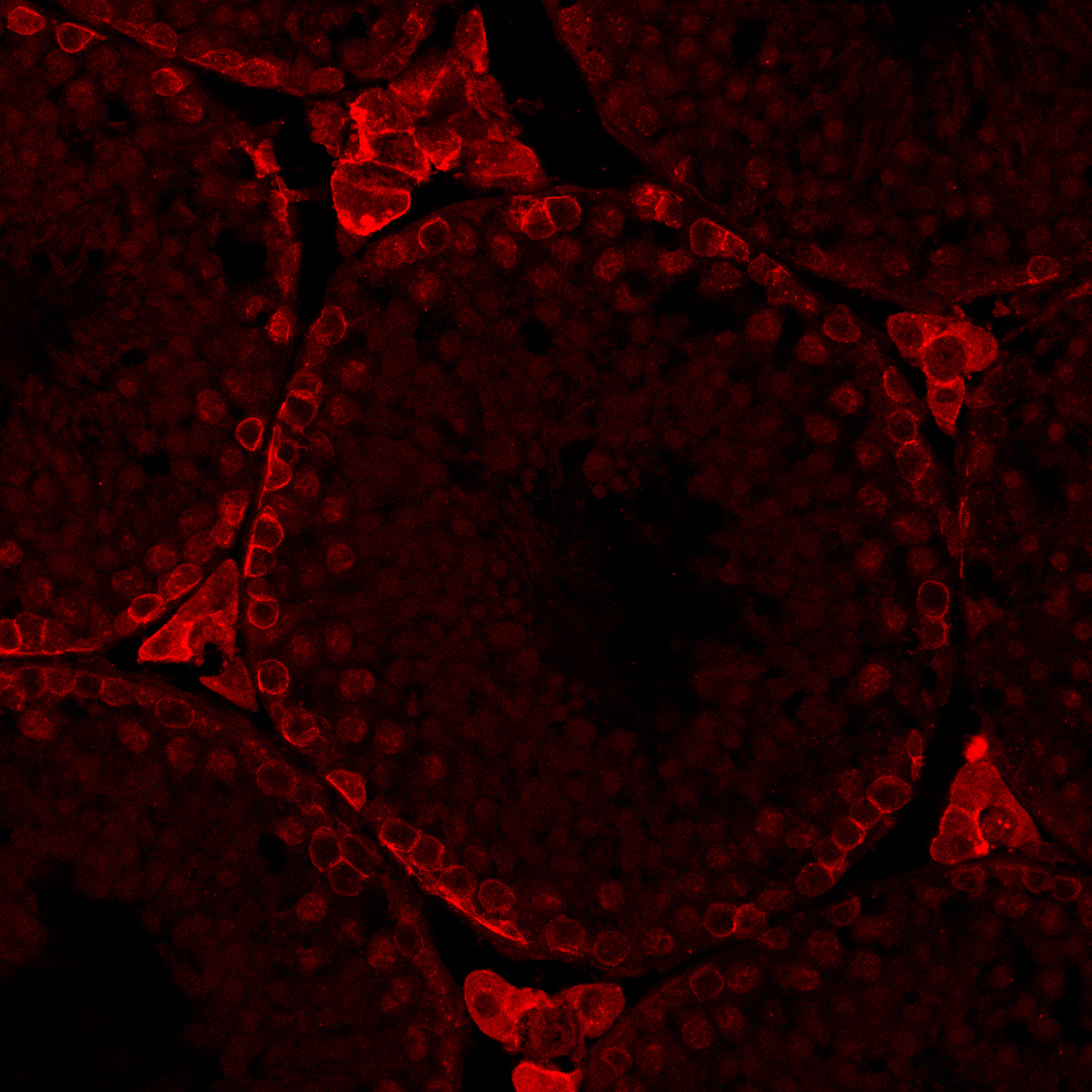

Supplement: Supplementary file 14 — EV and Appendix Figure Source Data [file 44318_2024_203_MOESM14_ESM.zip › Source Data for Expanded View and Appendix/Figure EV3/EV3H/Ctrl-cKIT.jpg]

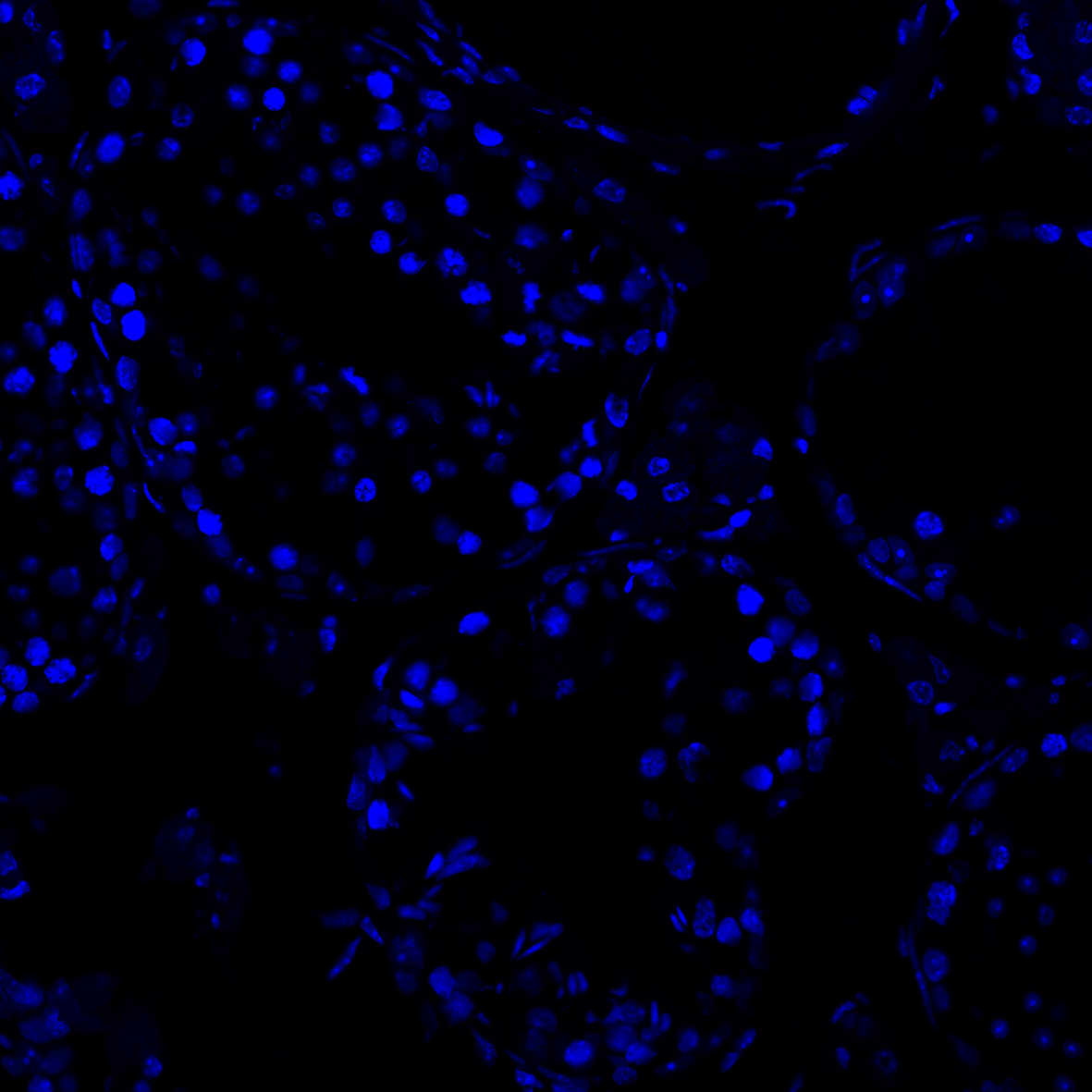

Supplement: Supplementary file 14 — EV and Appendix Figure Source Data [file 44318_2024_203_MOESM14_ESM.zip › Source Data for Expanded View and Appendix/Figure EV3/EV3H/cKO-DAPI.jpg]

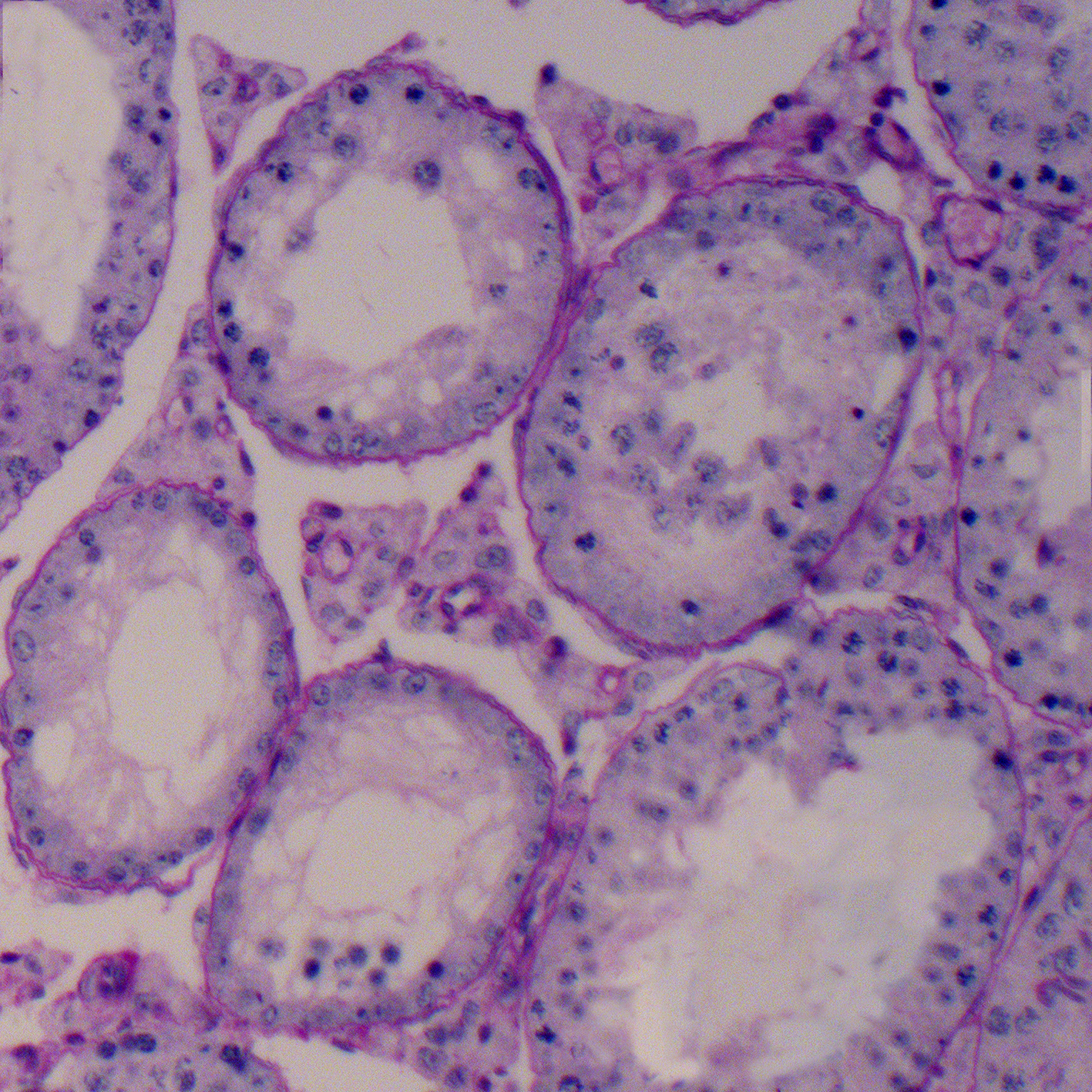

Supplement: Supplementary file 14 — EV and Appendix Figure Source Data [file 44318_2024_203_MOESM14_ESM.zip › Source Data for Expanded View and Appendix/Figure EV3/EV3D/cKO.jpg]

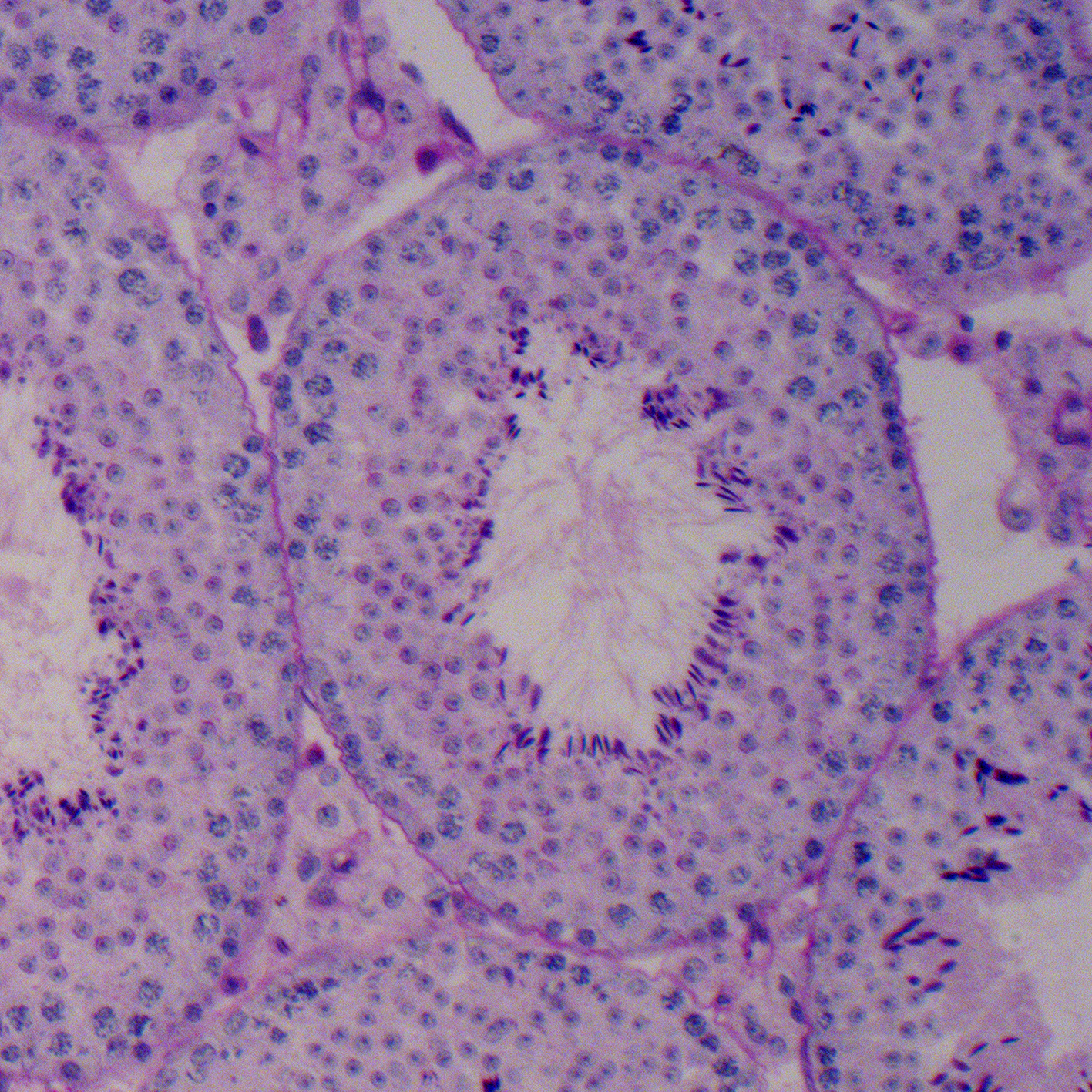

Supplement: Supplementary file 14 — EV and Appendix Figure Source Data [file 44318_2024_203_MOESM14_ESM.zip › Source Data for Expanded View and Appendix/Figure EV3/EV3D/Ctrl.jpg]

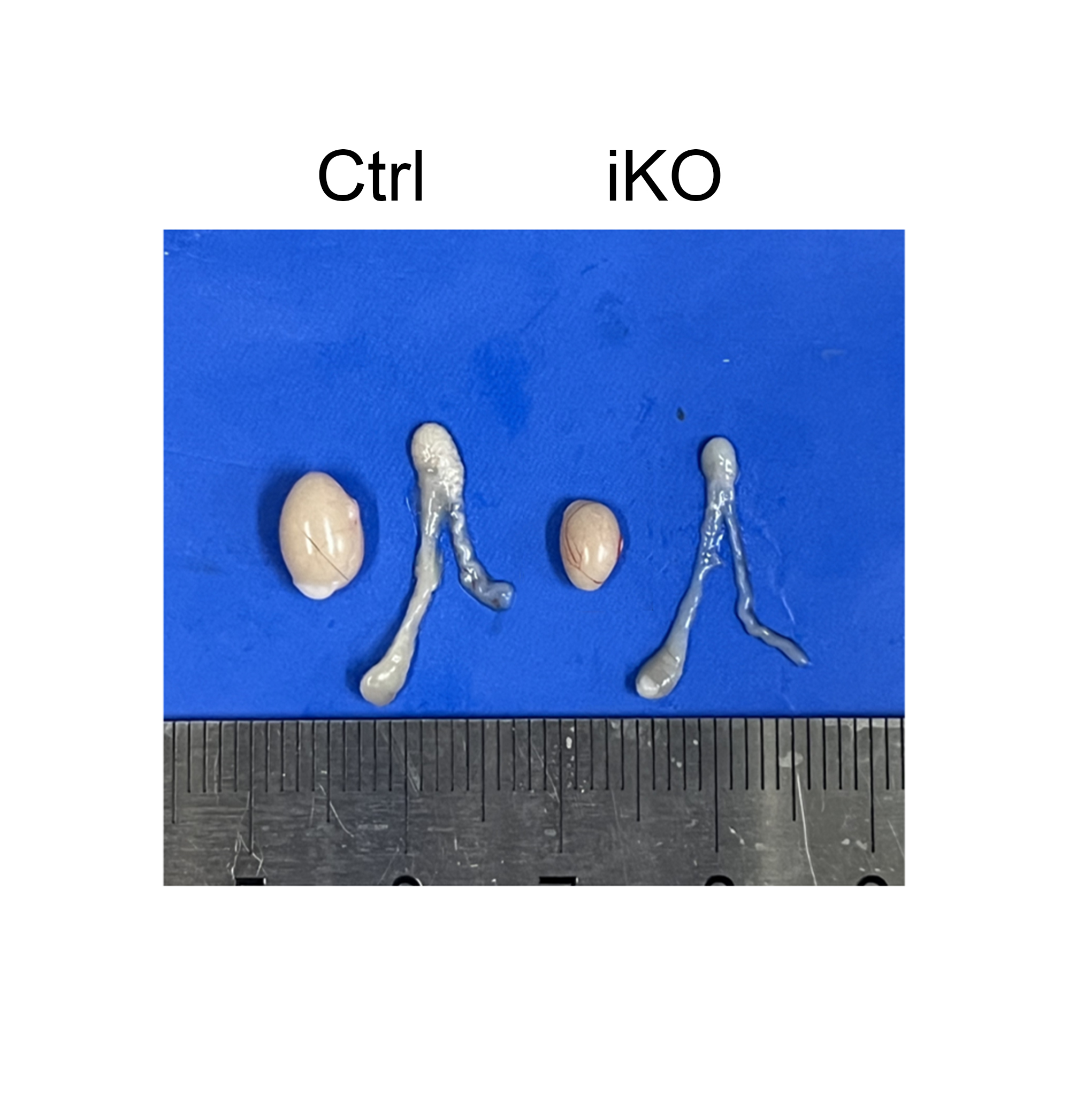

Supplement: Supplementary file 14 — EV and Appendix Figure Source Data [file 44318_2024_203_MOESM14_ESM.zip › Source Data for Expanded View and Appendix/Figure EV3/EV3C/EV3C-testis.jpg]

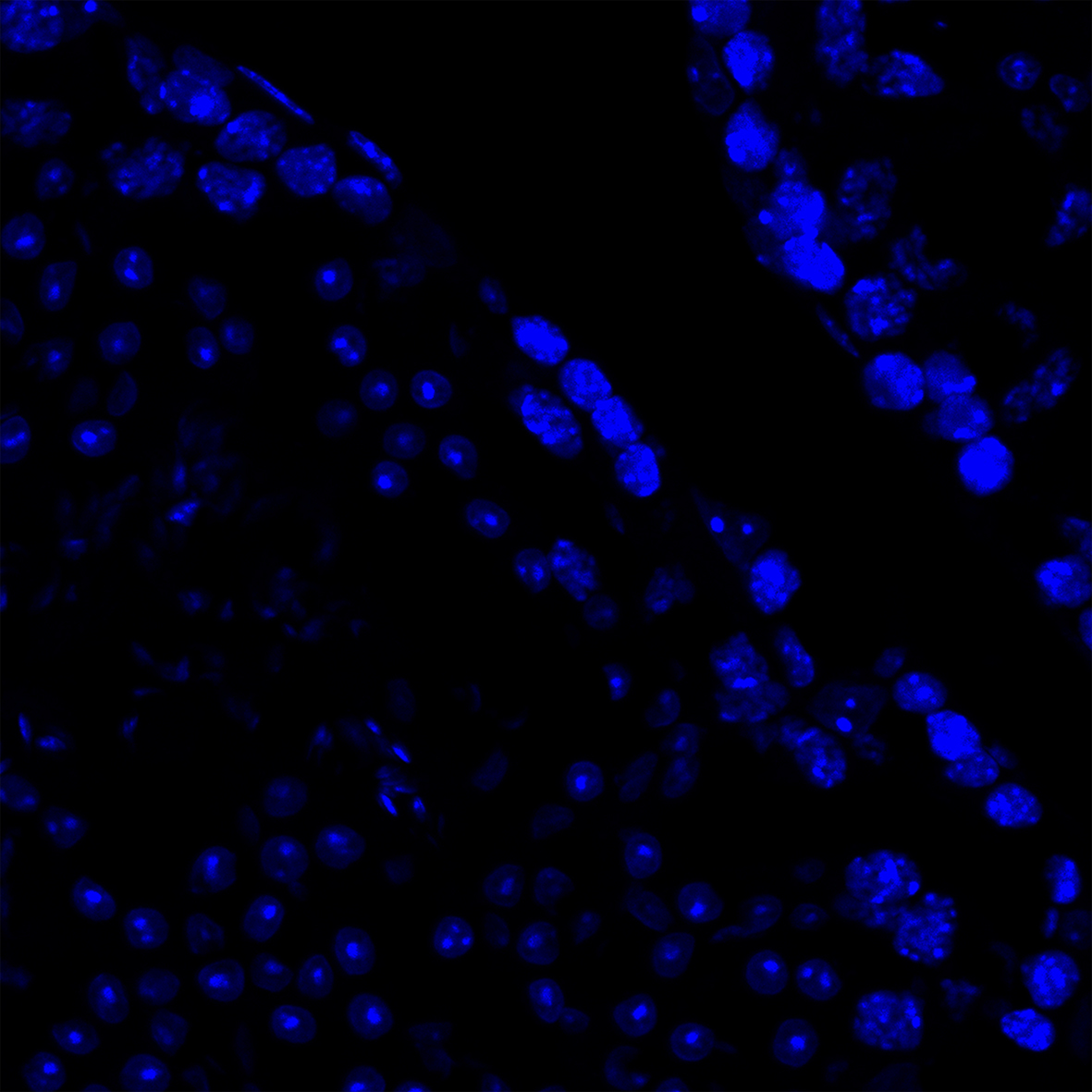

Supplement: Supplementary file 14 — EV and Appendix Figure Source Data [file 44318_2024_203_MOESM14_ESM.zip › Source Data for Expanded View and Appendix/Figure EV3/EV3B/Ctrl-DAPI.jpg]

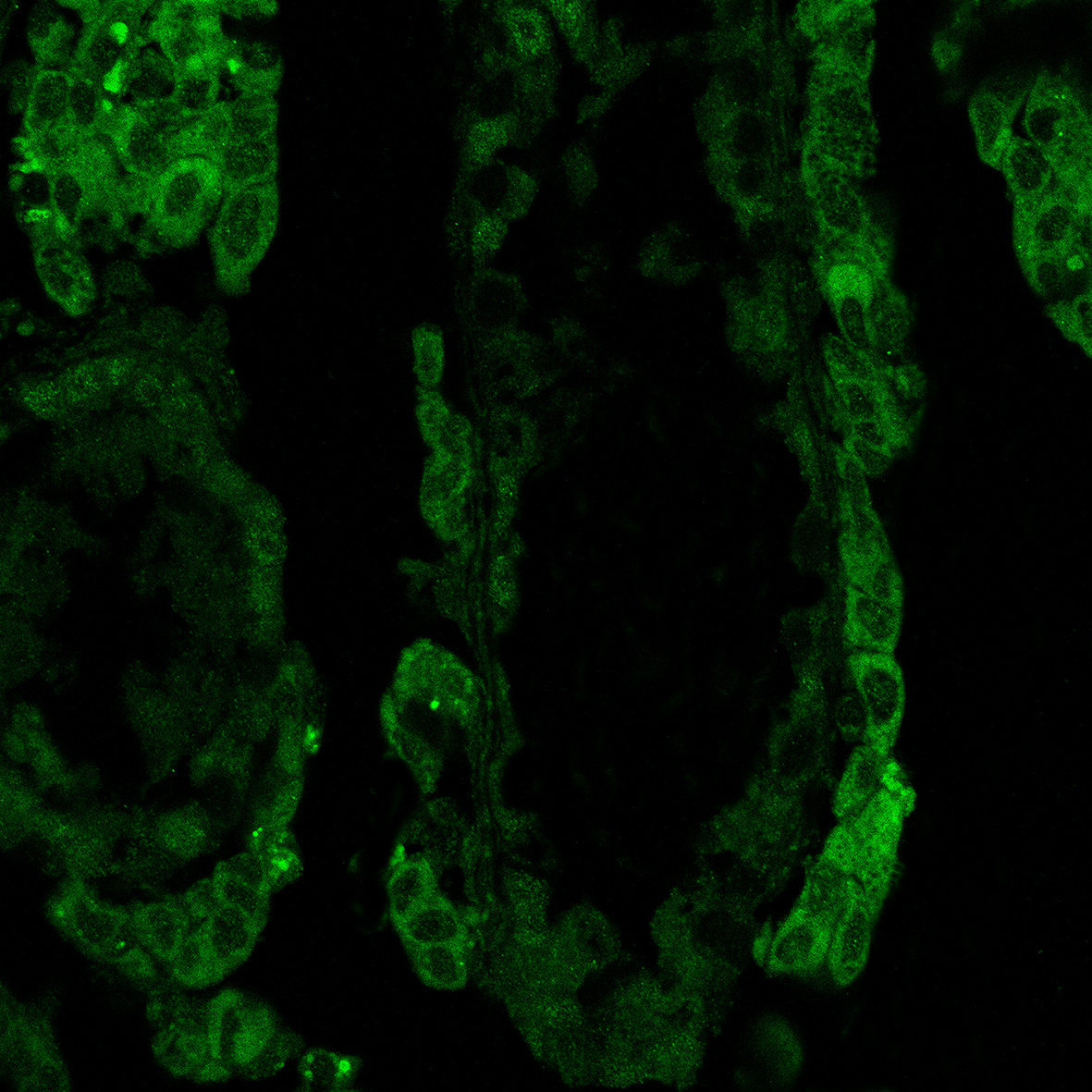

Supplement: Supplementary file 14 — EV and Appendix Figure Source Data [file 44318_2024_203_MOESM14_ESM.zip › Source Data for Expanded View and Appendix/Figure EV3/EV3B/cKO-KDM2A.jpg]

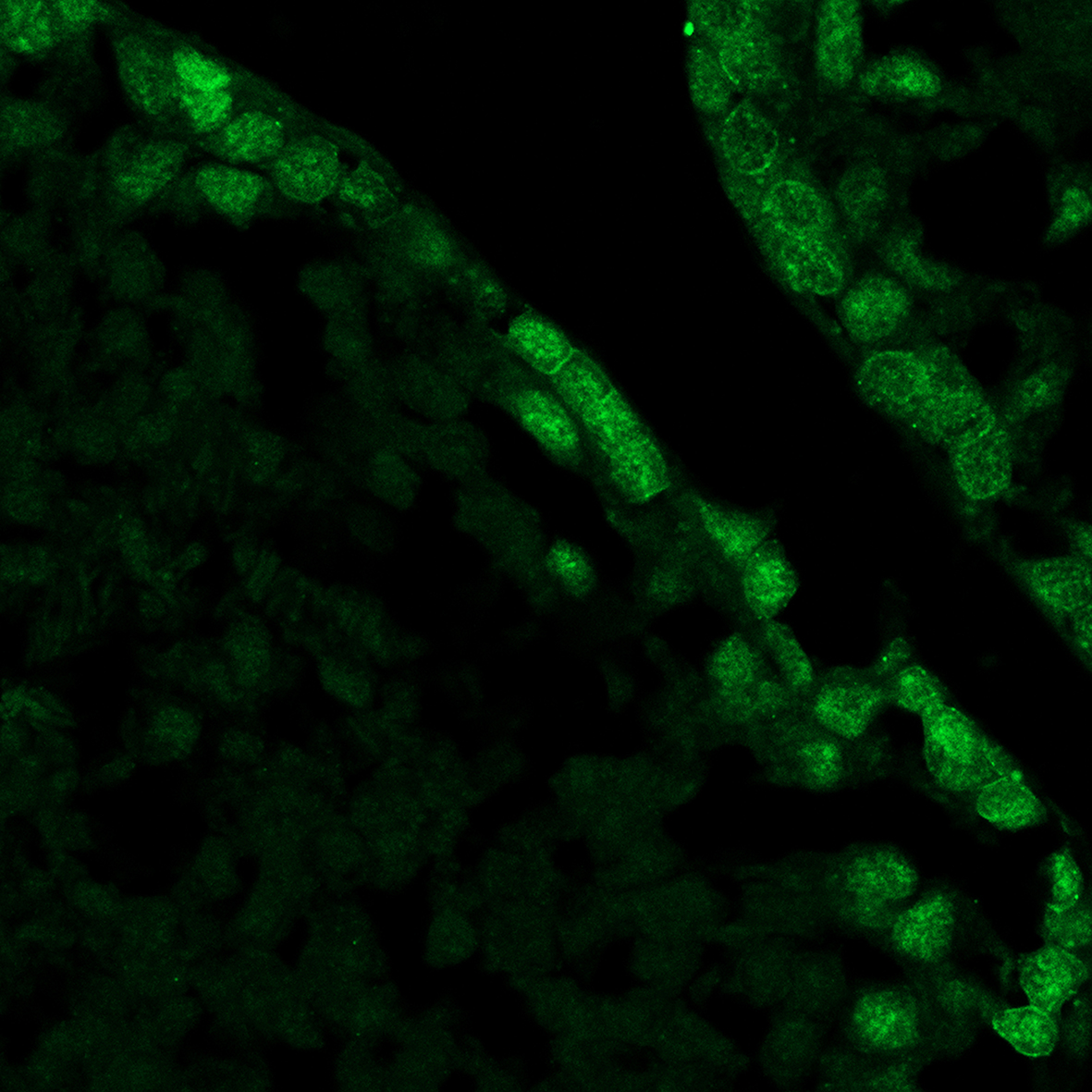

Supplement: Supplementary file 14 — EV and Appendix Figure Source Data [file 44318_2024_203_MOESM14_ESM.zip › Source Data for Expanded View and Appendix/Figure EV3/EV3B/Ctrl-KDM2A.jpg]

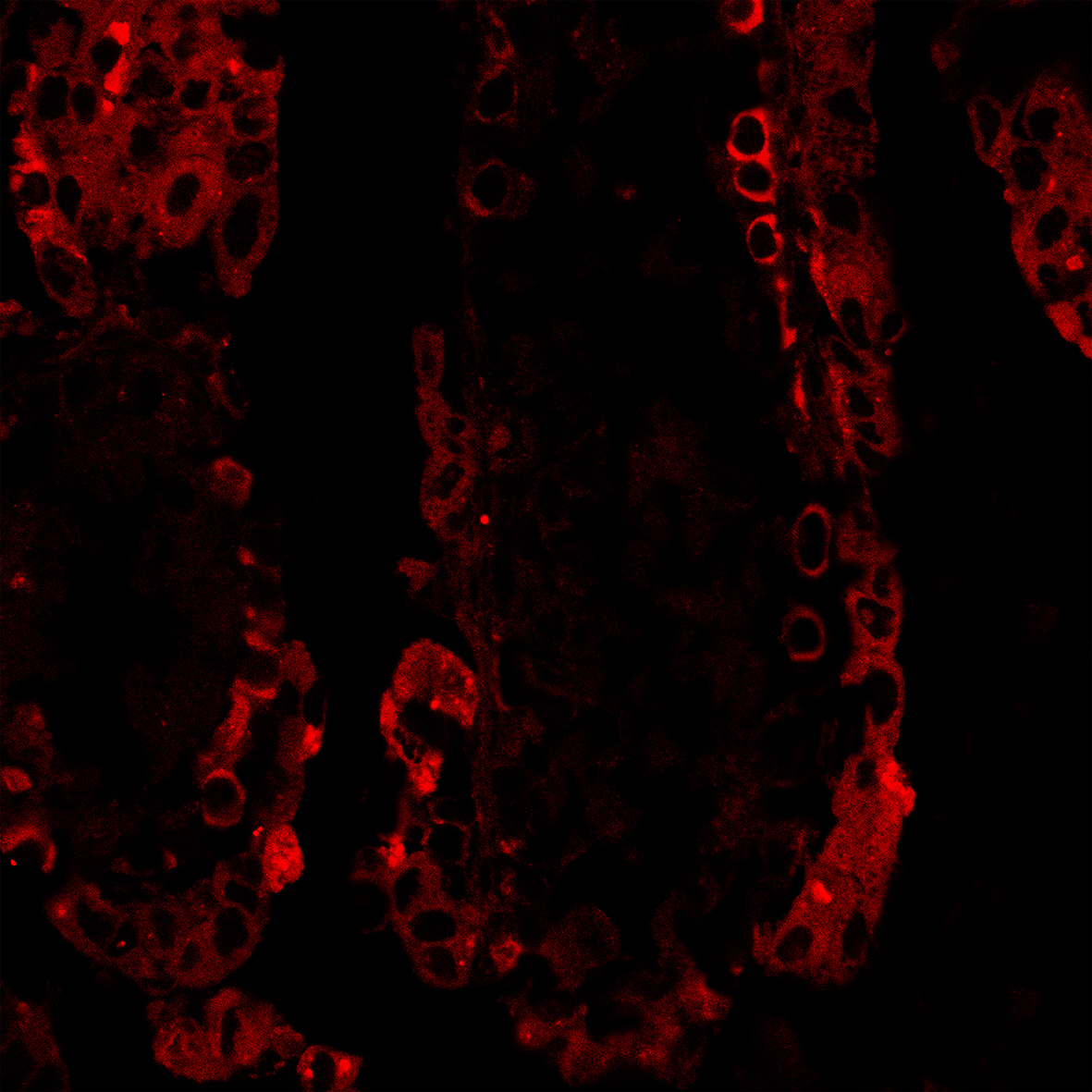

Supplement: Supplementary file 14 — EV and Appendix Figure Source Data [file 44318_2024_203_MOESM14_ESM.zip › Source Data for Expanded View and Appendix/Figure EV3/EV3B/cKO-cKIT.jpg]

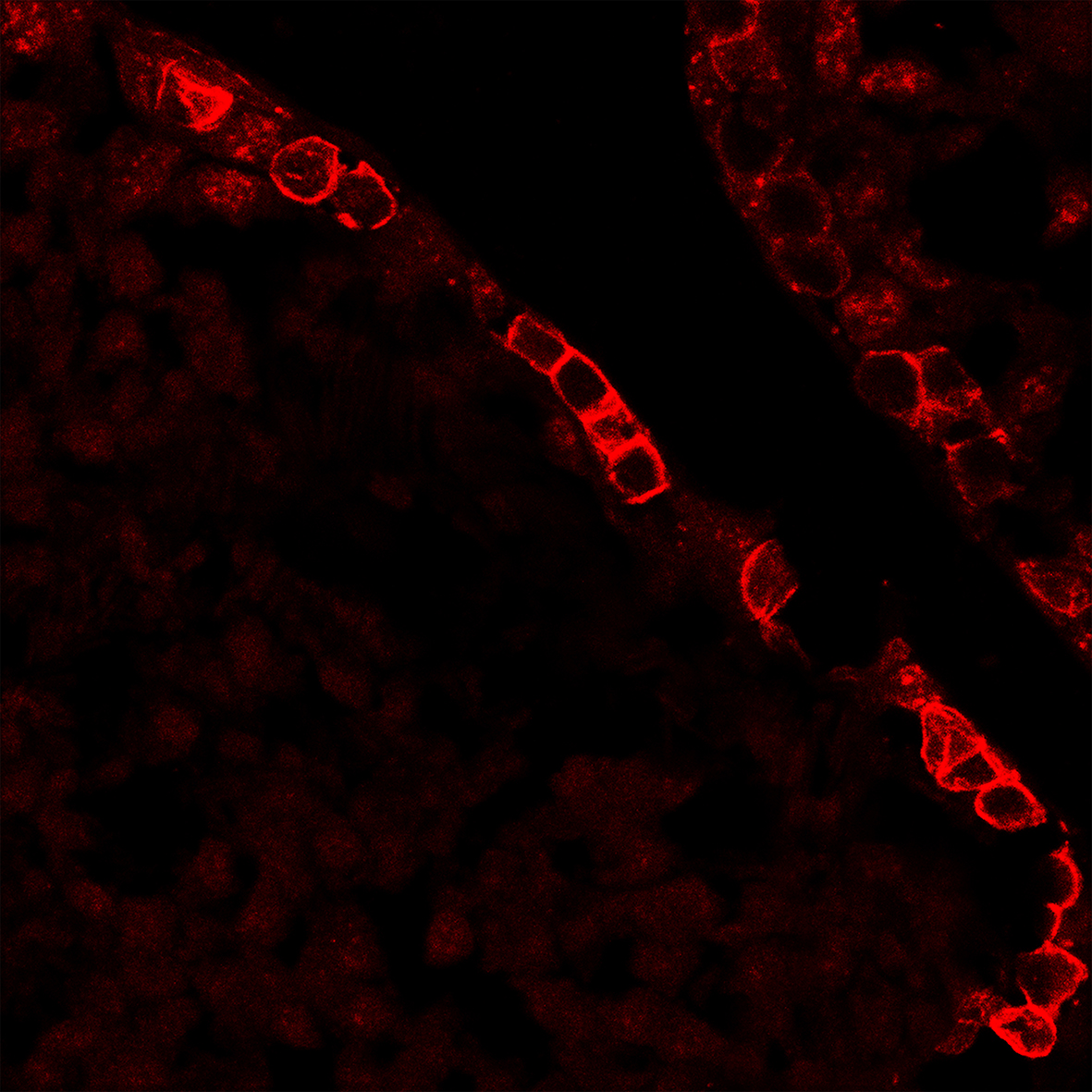

Supplement: Supplementary file 14 — EV and Appendix Figure Source Data [file 44318_2024_203_MOESM14_ESM.zip › Source Data for Expanded View and Appendix/Figure EV3/EV3B/Ctrl-cKIT.jpg]

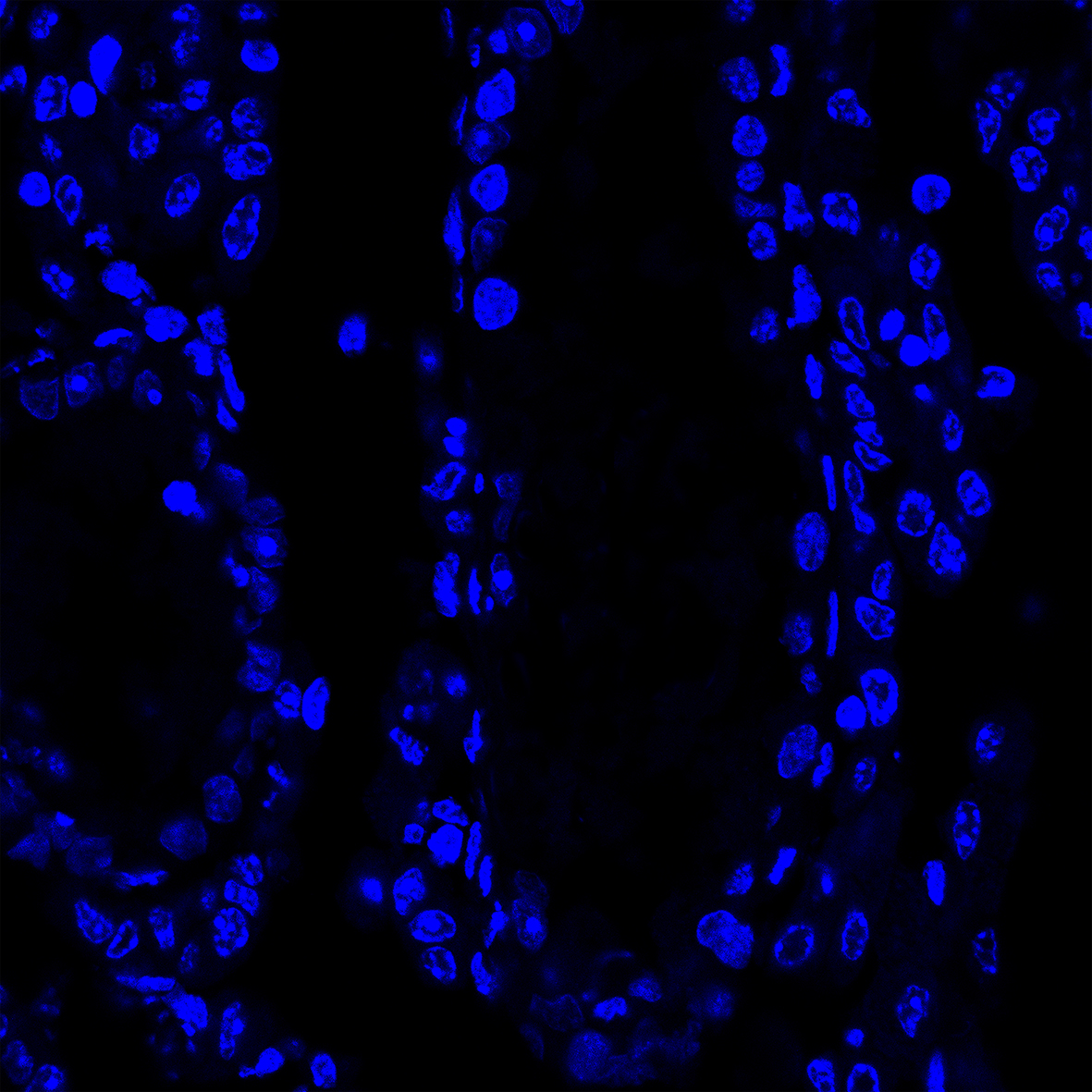

Supplement: Supplementary file 14 — EV and Appendix Figure Source Data [file 44318_2024_203_MOESM14_ESM.zip › Source Data for Expanded View and Appendix/Figure EV3/EV3B/cKO-DAPI.jpg]

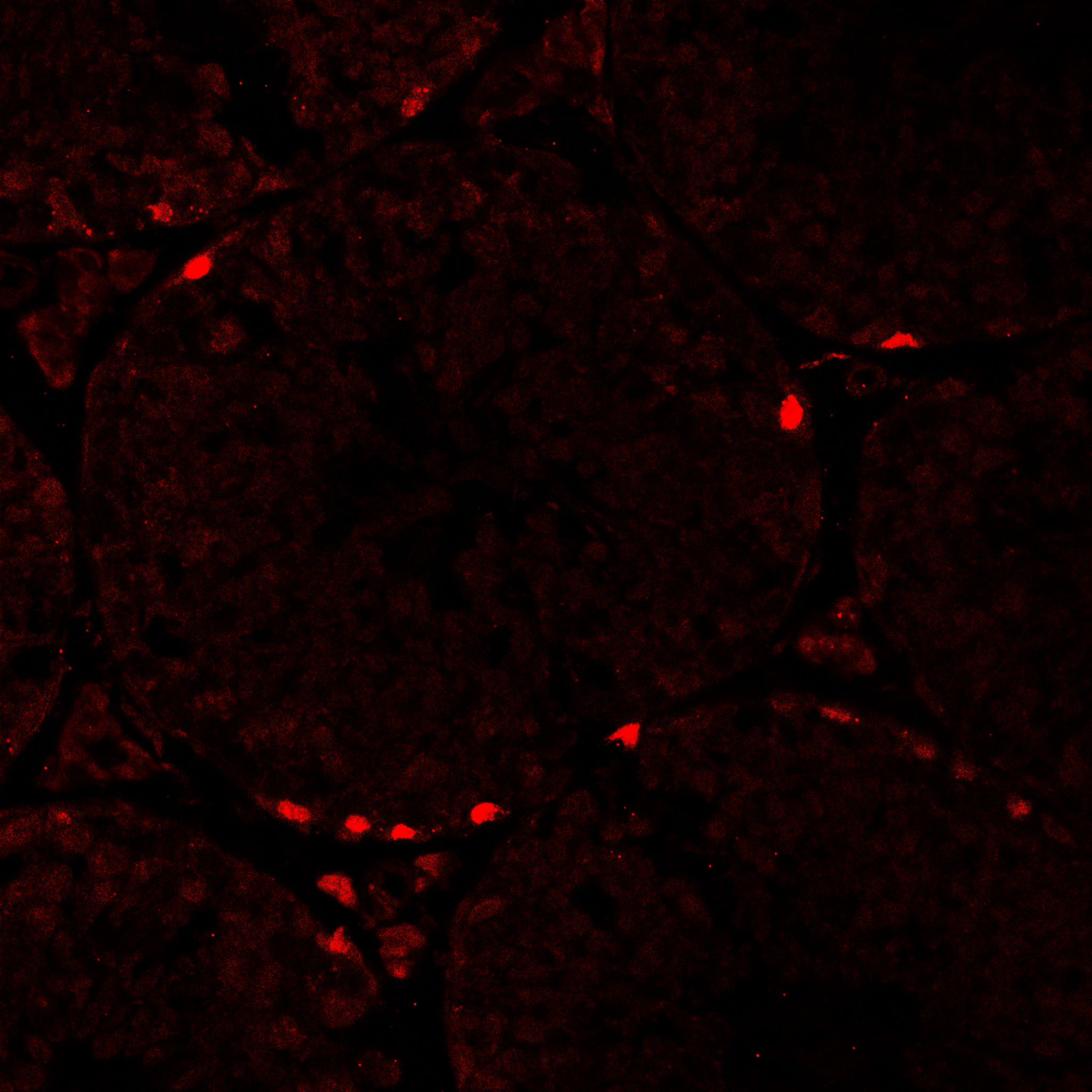

Supplement: Supplementary file 14 — EV and Appendix Figure Source Data [file 44318_2024_203_MOESM14_ESM.zip › Source Data for Expanded View and Appendix/Figure EV3/EV3E/Ctrl-PLZF.jpg]

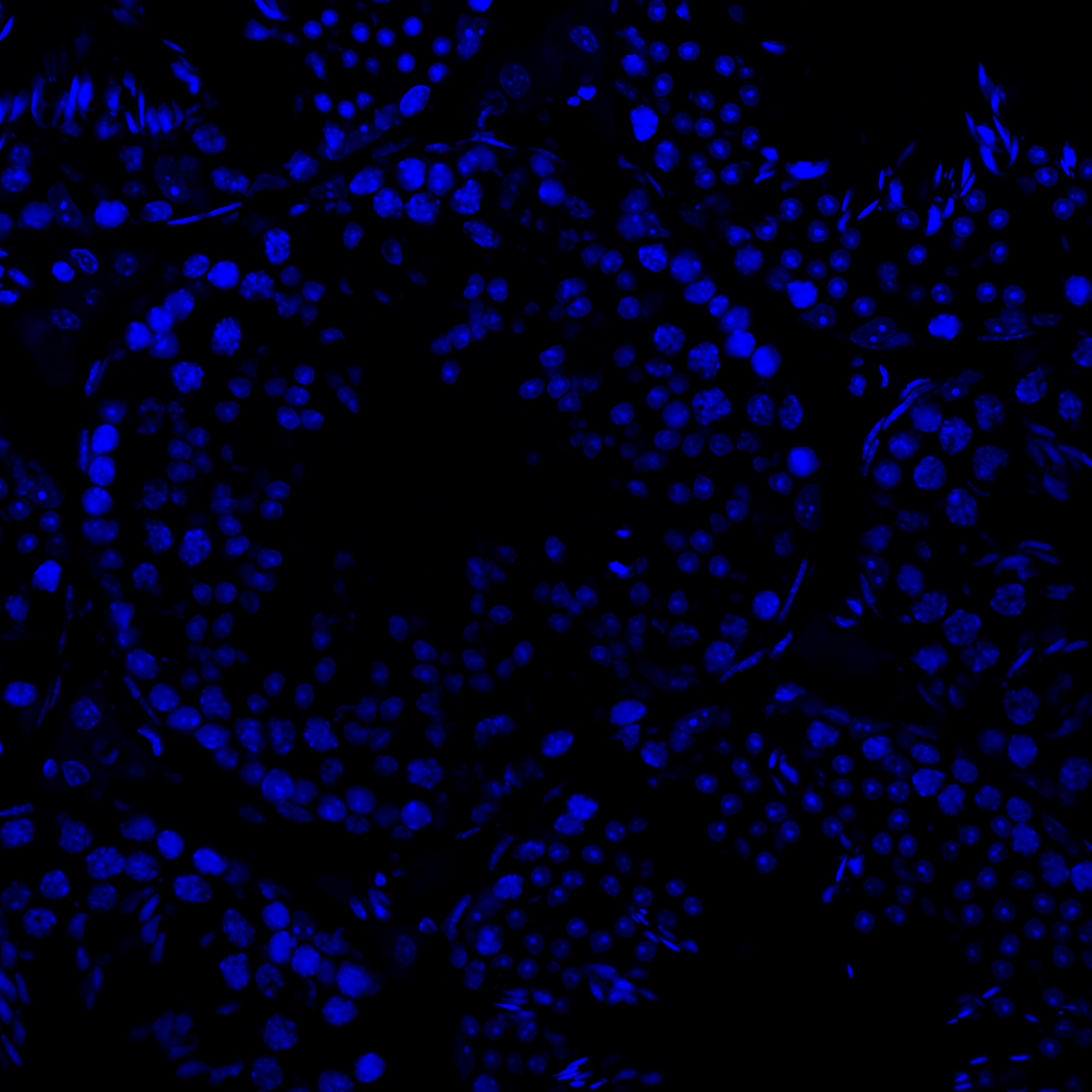

Supplement: Supplementary file 14 — EV and Appendix Figure Source Data [file 44318_2024_203_MOESM14_ESM.zip › Source Data for Expanded View and Appendix/Figure EV3/EV3E/Ctrl-DAPI.jpg]

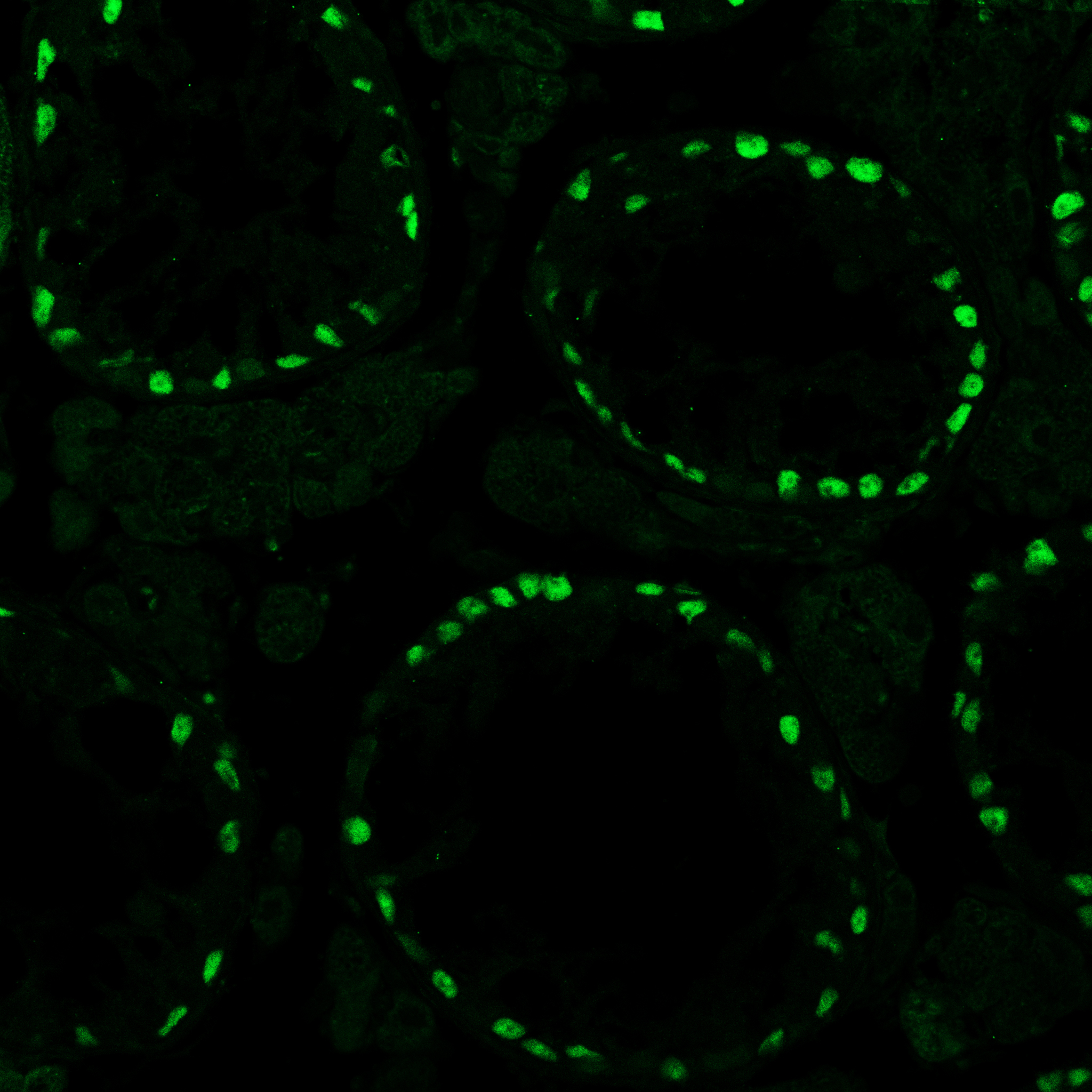

Supplement: Supplementary file 14 — EV and Appendix Figure Source Data [file 44318_2024_203_MOESM14_ESM.zip › Source Data for Expanded View and Appendix/Figure EV3/EV3E/cKO-WT1.jpg]

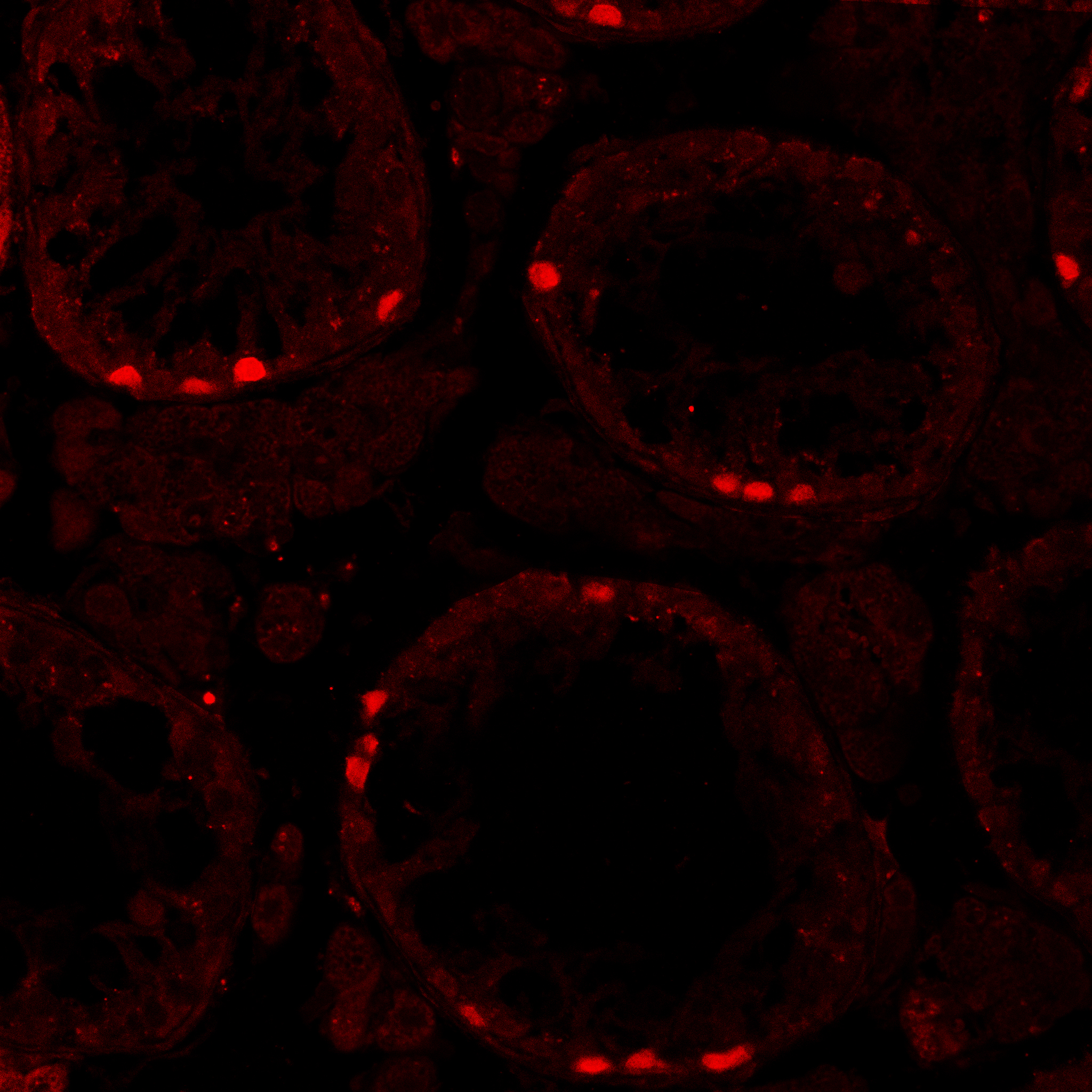

Supplement: Supplementary file 14 — EV and Appendix Figure Source Data [file 44318_2024_203_MOESM14_ESM.zip › Source Data for Expanded View and Appendix/Figure EV3/EV3E/cKO-cKIT.jpg]

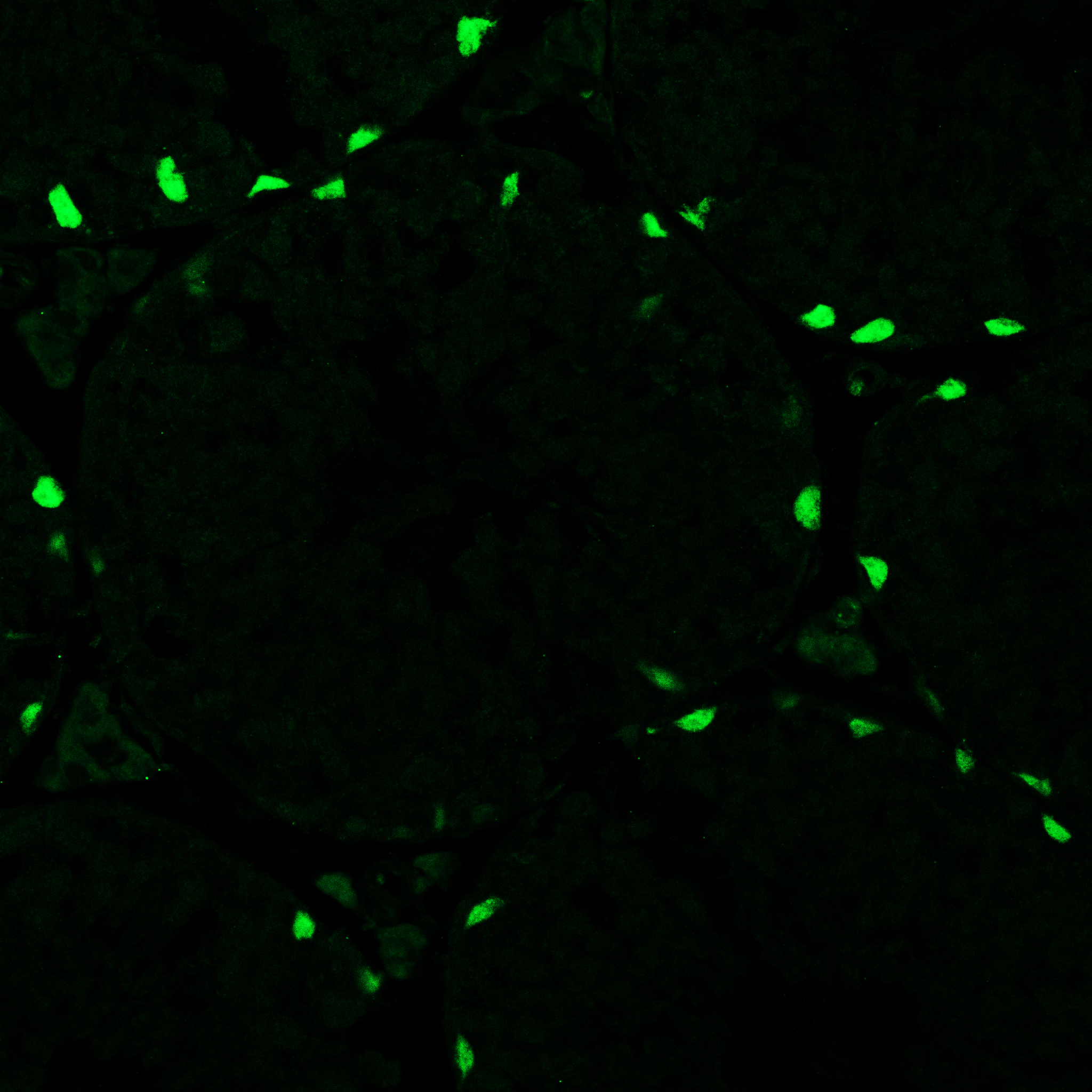

Supplement: Supplementary file 14 — EV and Appendix Figure Source Data [file 44318_2024_203_MOESM14_ESM.zip › Source Data for Expanded View and Appendix/Figure EV3/EV3E/Ctrl-WT1.jpg]

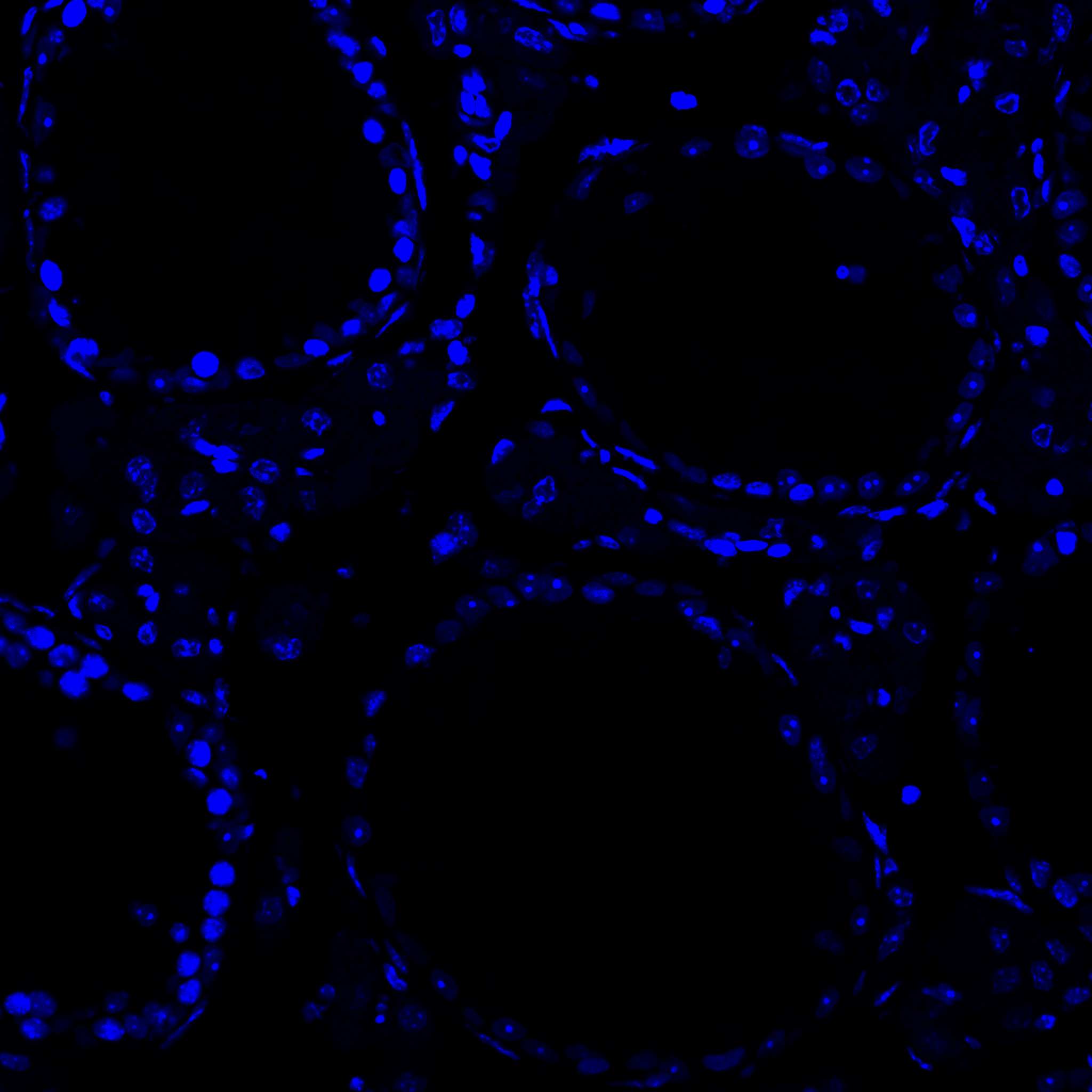

Supplement: Supplementary file 14 — EV and Appendix Figure Source Data [file 44318_2024_203_MOESM14_ESM.zip › Source Data for Expanded View and Appendix/Figure EV3/EV3E/cKO-DAPI.jpg]

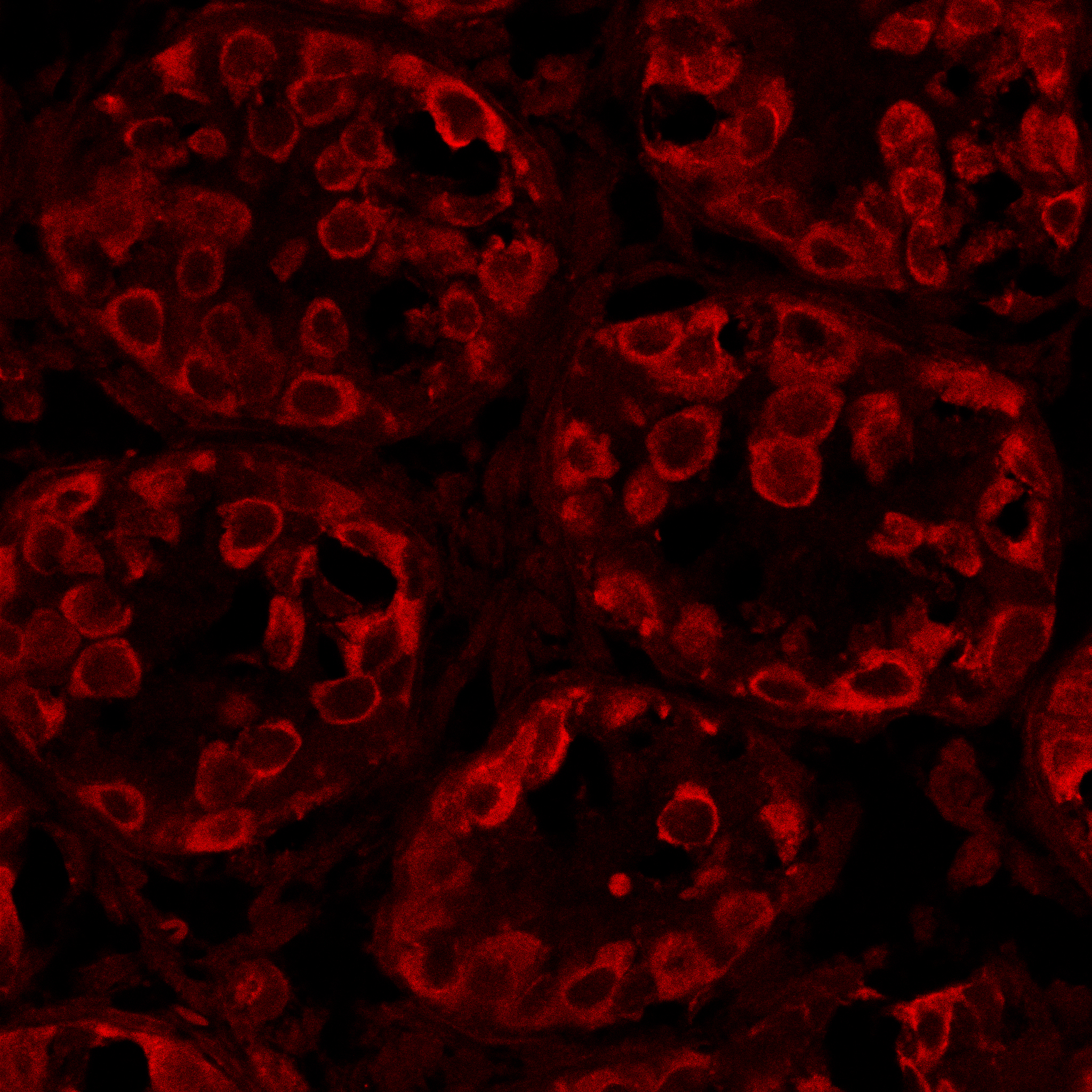

Supplement: Supplementary file 14 — EV and Appendix Figure Source Data [file 44318_2024_203_MOESM14_ESM.zip › Source Data for Expanded View and Appendix/Figure EV2/EV2B/P8-Ctrl-DDX4.jpg]

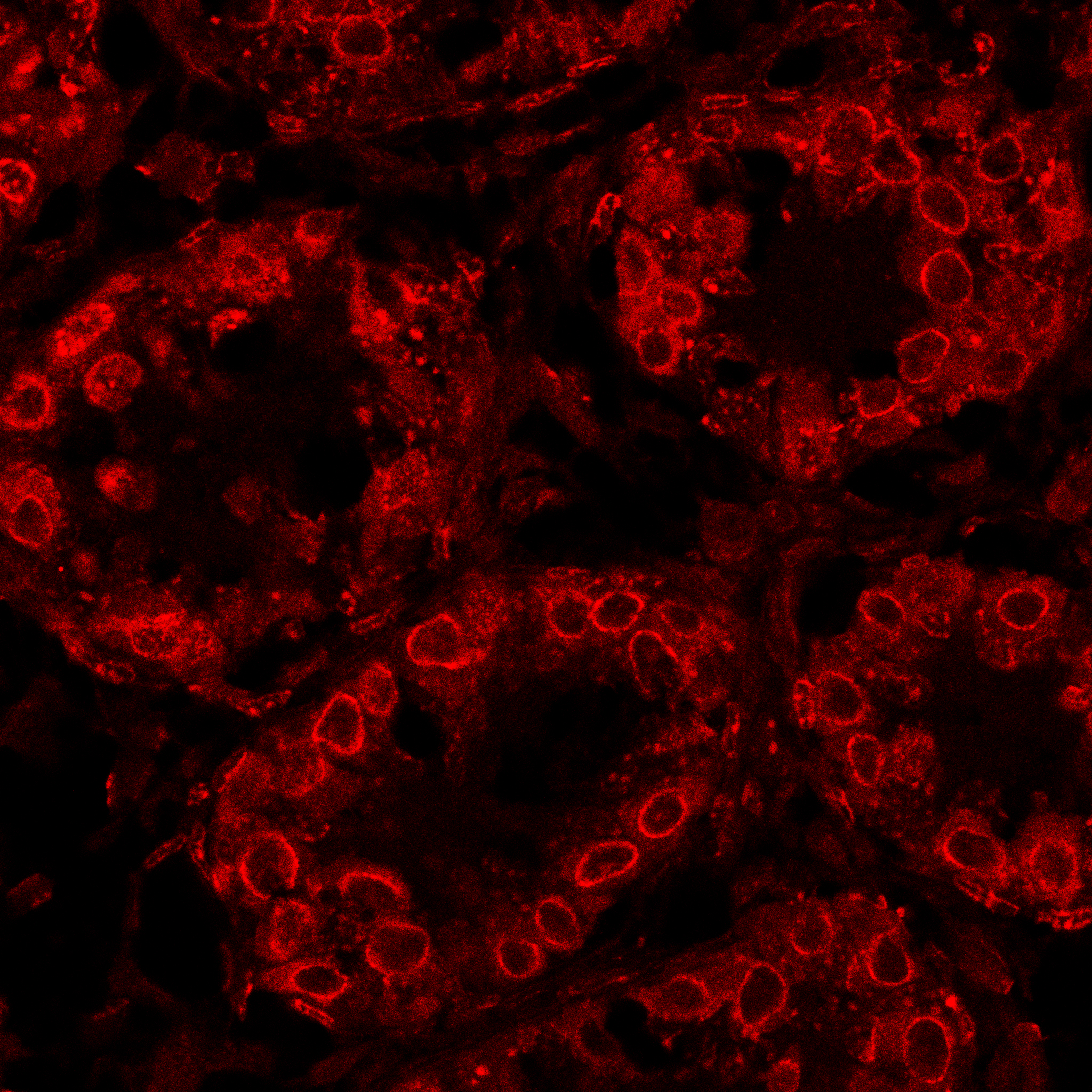

Supplement: Supplementary file 14 — EV and Appendix Figure Source Data [file 44318_2024_203_MOESM14_ESM.zip › Source Data for Expanded View and Appendix/Figure EV2/EV2B/P8-cKO-DDX4.jpg]

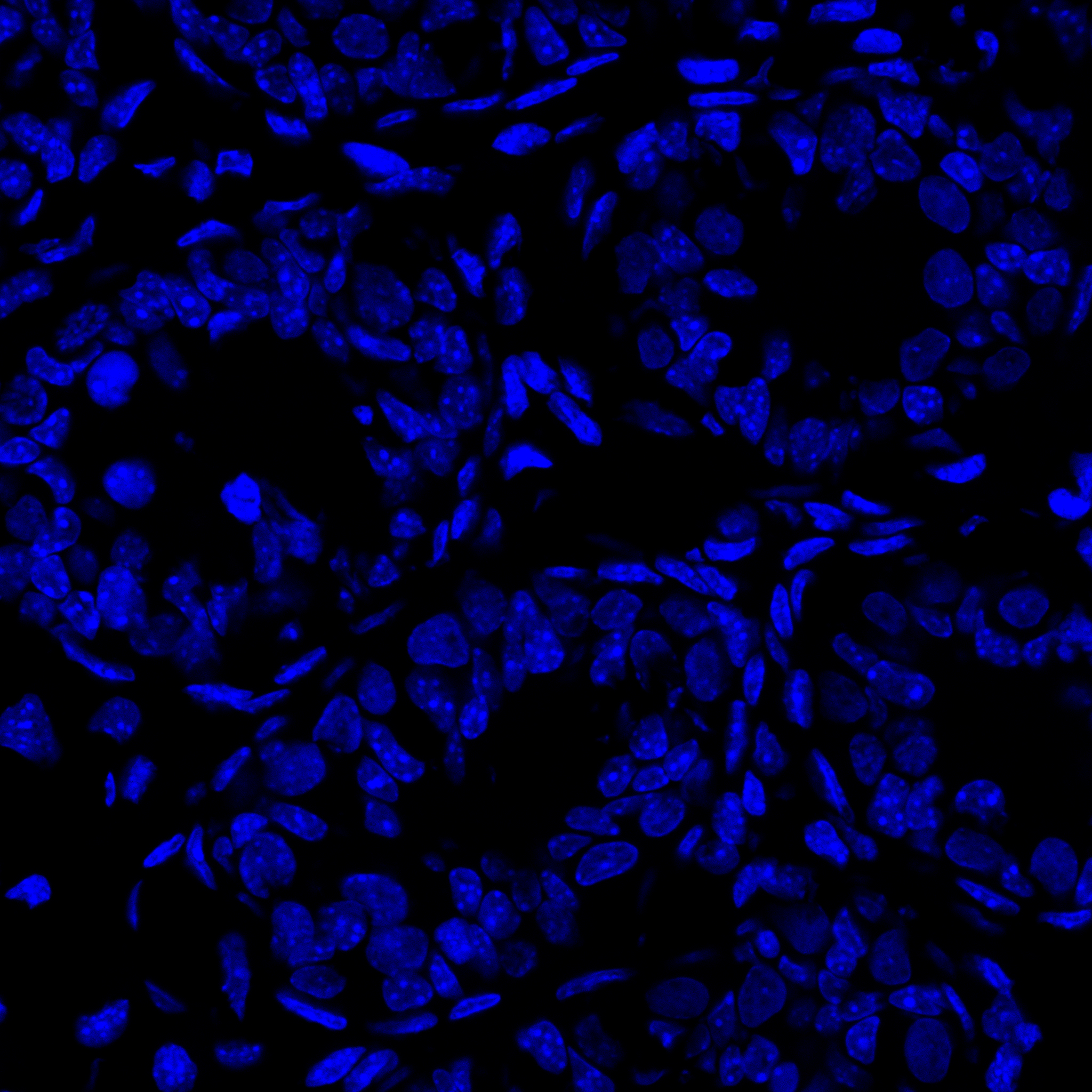

Supplement: Supplementary file 14 — EV and Appendix Figure Source Data [file 44318_2024_203_MOESM14_ESM.zip › Source Data for Expanded View and Appendix/Figure EV2/EV2B/P8-cKO-DAPI.jpg]

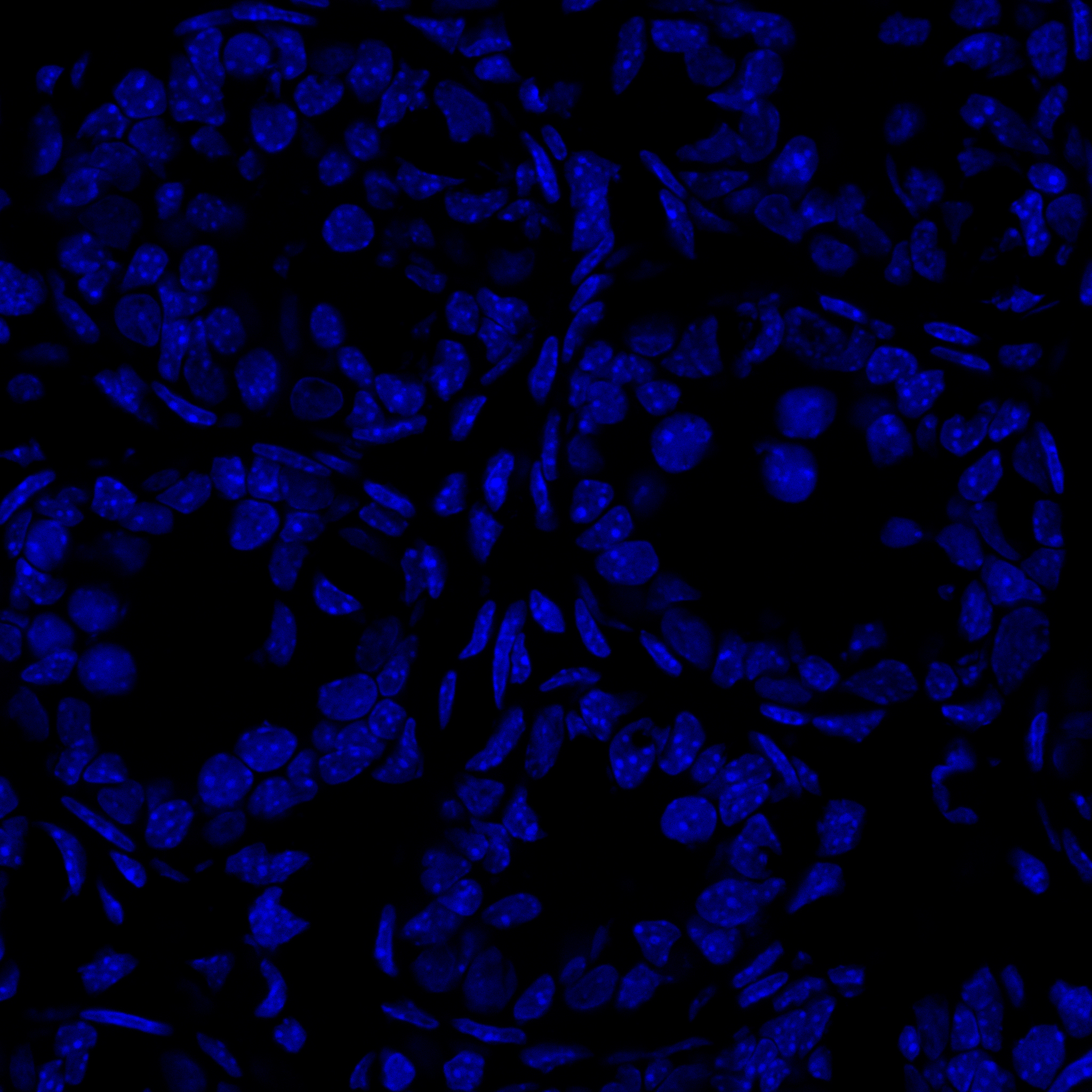

Supplement: Supplementary file 14 — EV and Appendix Figure Source Data [file 44318_2024_203_MOESM14_ESM.zip › Source Data for Expanded View and Appendix/Figure EV2/EV2B/P8-Ctrl-DAPI.jpg]

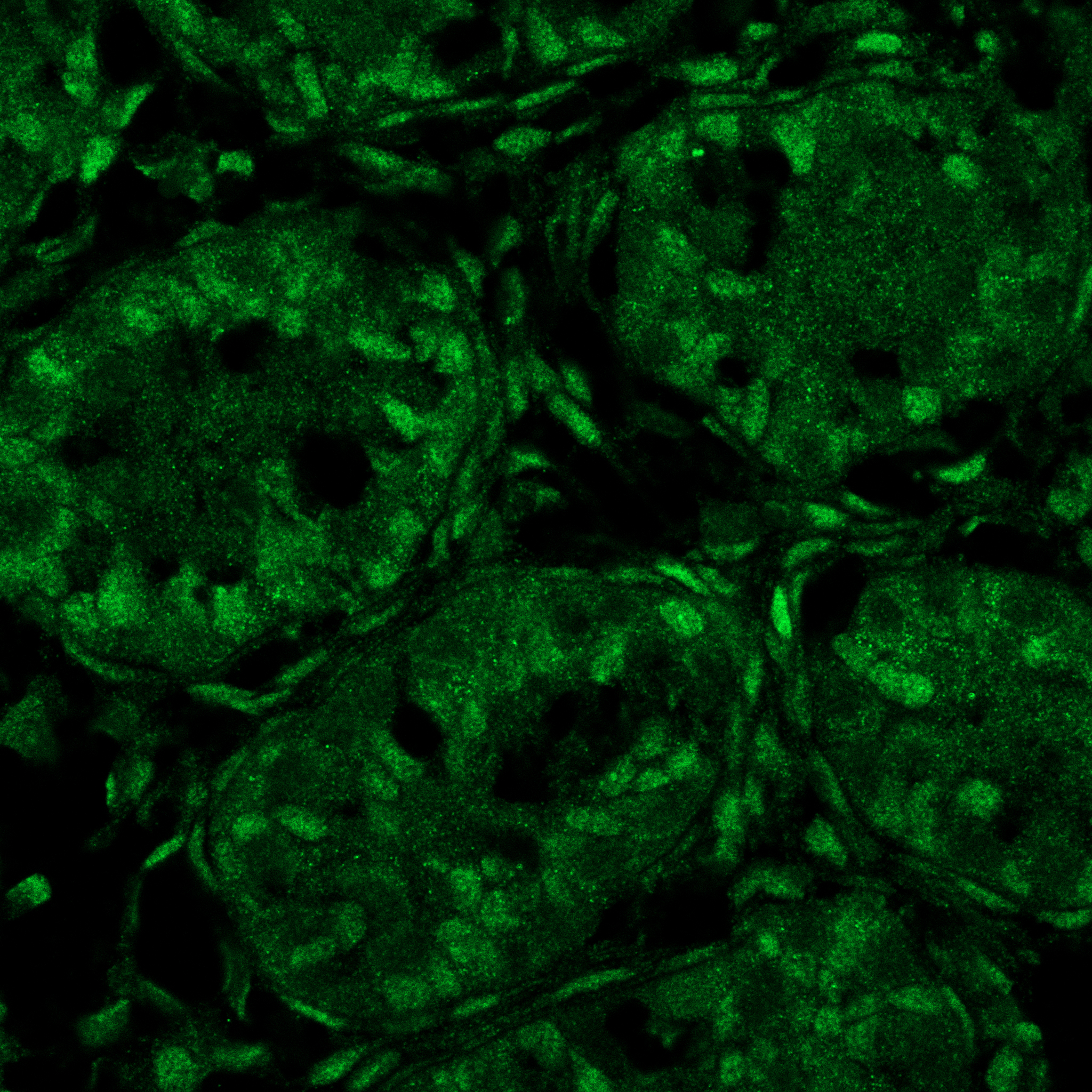

Supplement: Supplementary file 14 — EV and Appendix Figure Source Data [file 44318_2024_203_MOESM14_ESM.zip › Source Data for Expanded View and Appendix/Figure EV2/EV2B/P8-cKO-KDM2A.jpg]

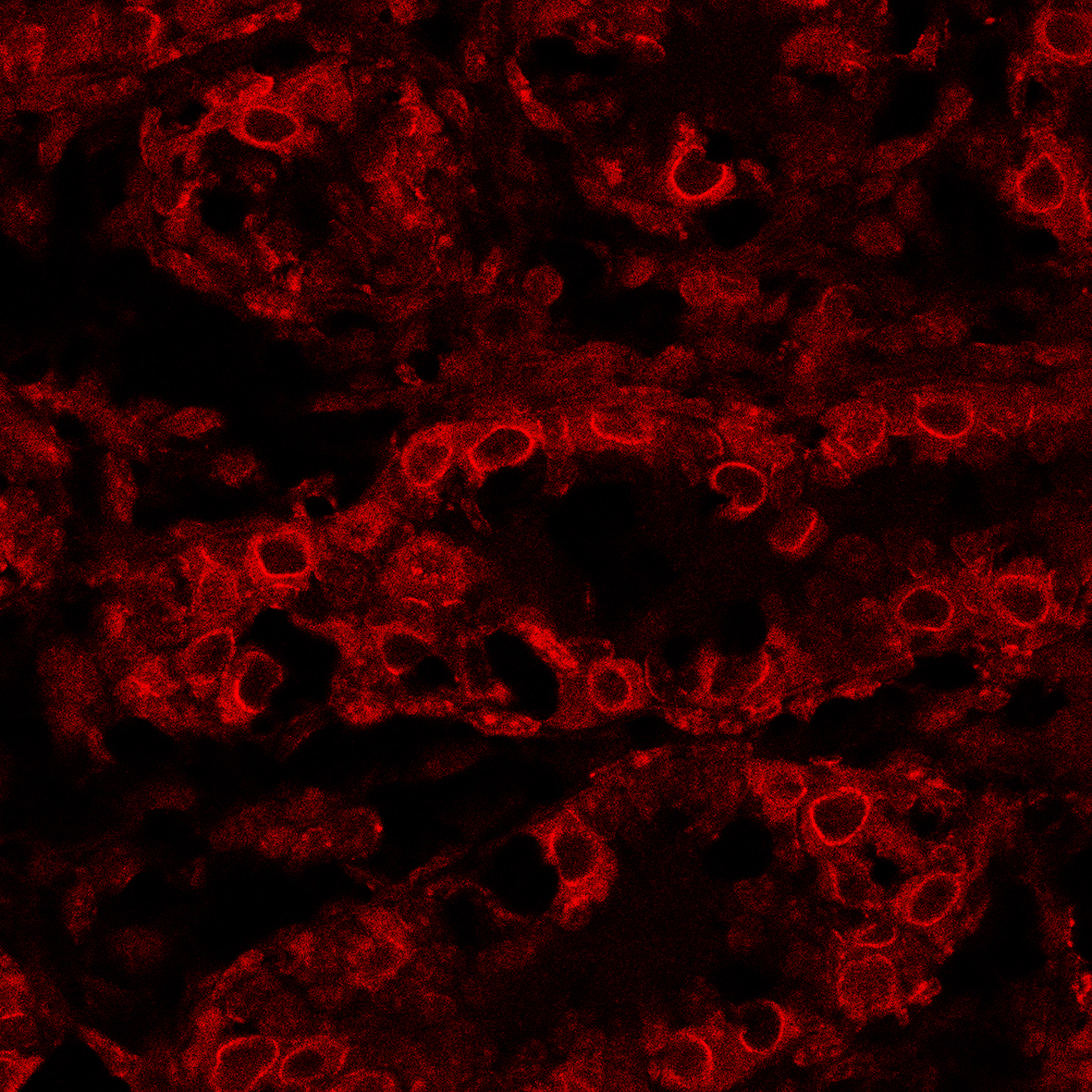

Supplement: Supplementary file 14 — EV and Appendix Figure Source Data [file 44318_2024_203_MOESM14_ESM.zip › Source Data for Expanded View and Appendix/Figure EV2/EV2B/P5-Ctrl-DDX4.jpg]

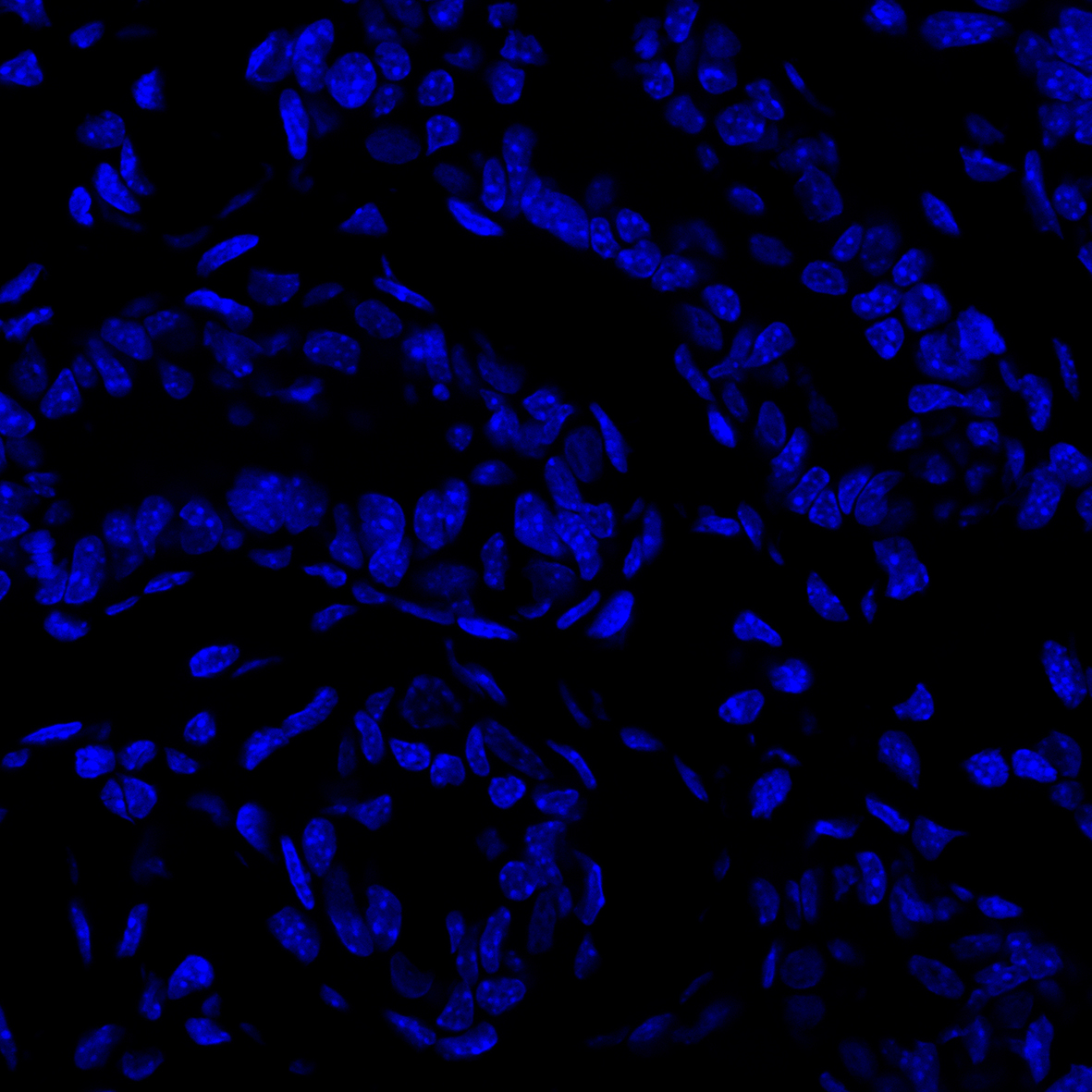

Supplement: Supplementary file 14 — EV and Appendix Figure Source Data [file 44318_2024_203_MOESM14_ESM.zip › Source Data for Expanded View and Appendix/Figure EV2/EV2B/P5-cKO-DAPI.jpg]

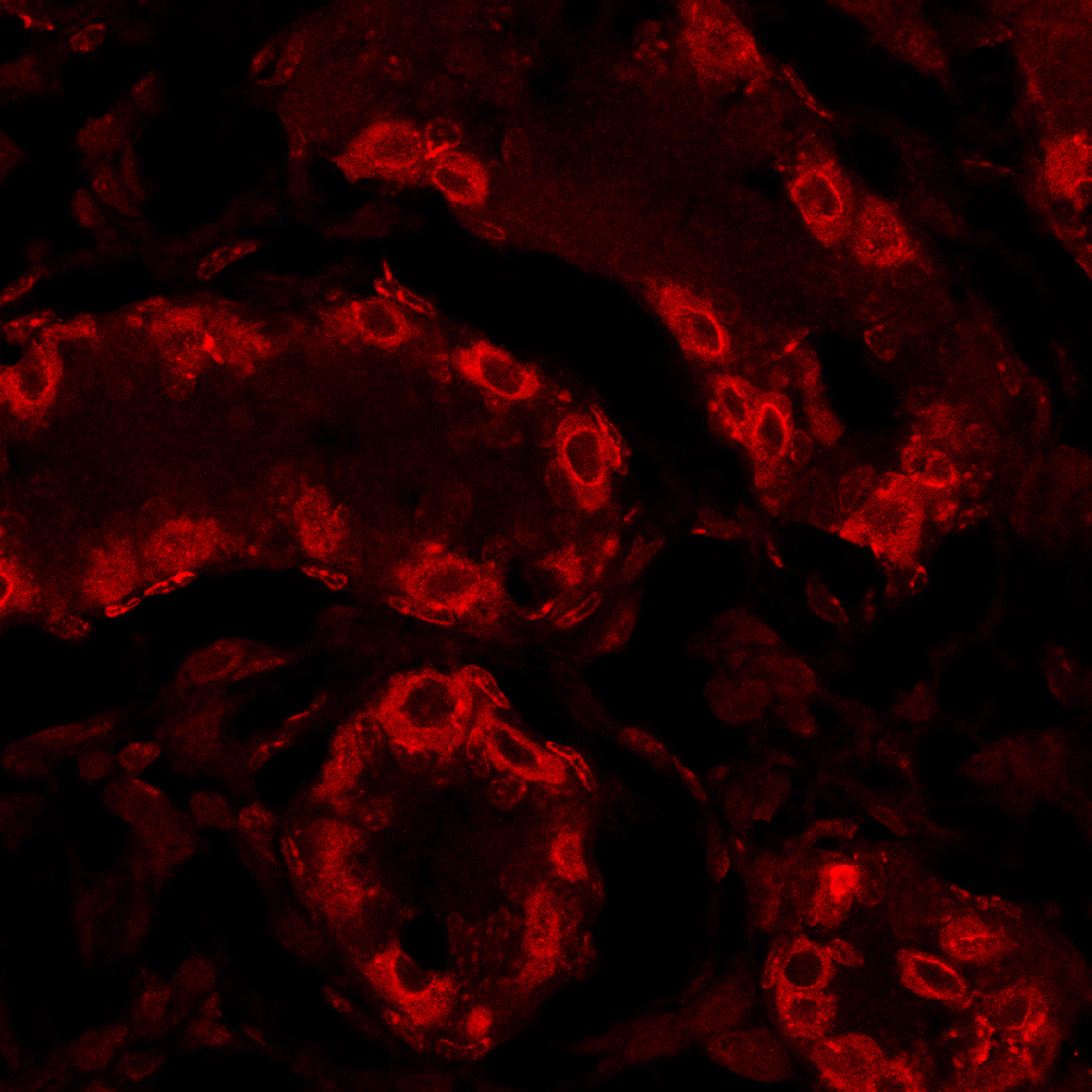

Supplement: Supplementary file 14 — EV and Appendix Figure Source Data [file 44318_2024_203_MOESM14_ESM.zip › Source Data for Expanded View and Appendix/Figure EV2/EV2B/P5-cKO-DDX4.jpg]

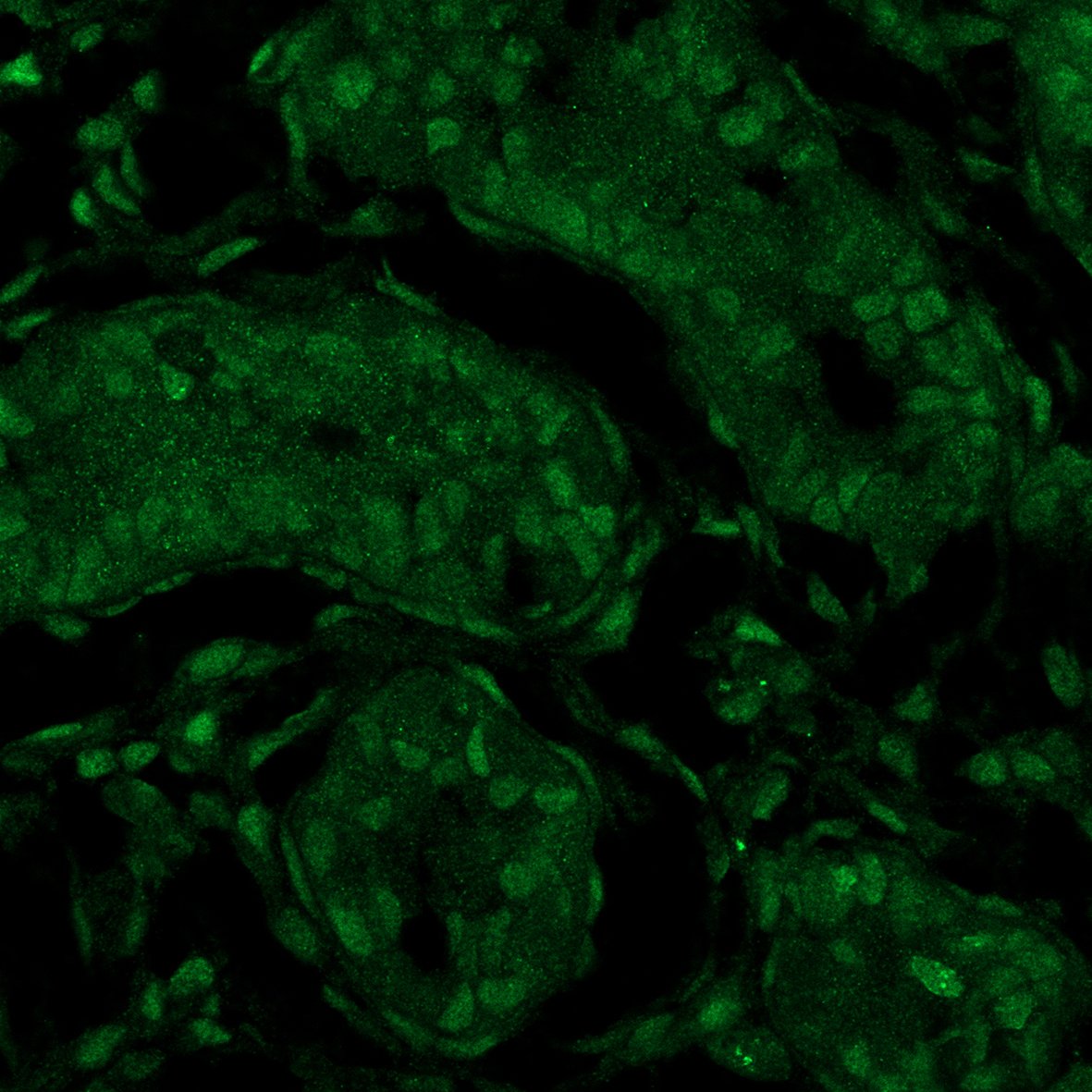

Supplement: Supplementary file 14 — EV and Appendix Figure Source Data [file 44318_2024_203_MOESM14_ESM.zip › Source Data for Expanded View and Appendix/Figure EV2/EV2B/P5-cKO-KDM2A.jpg]

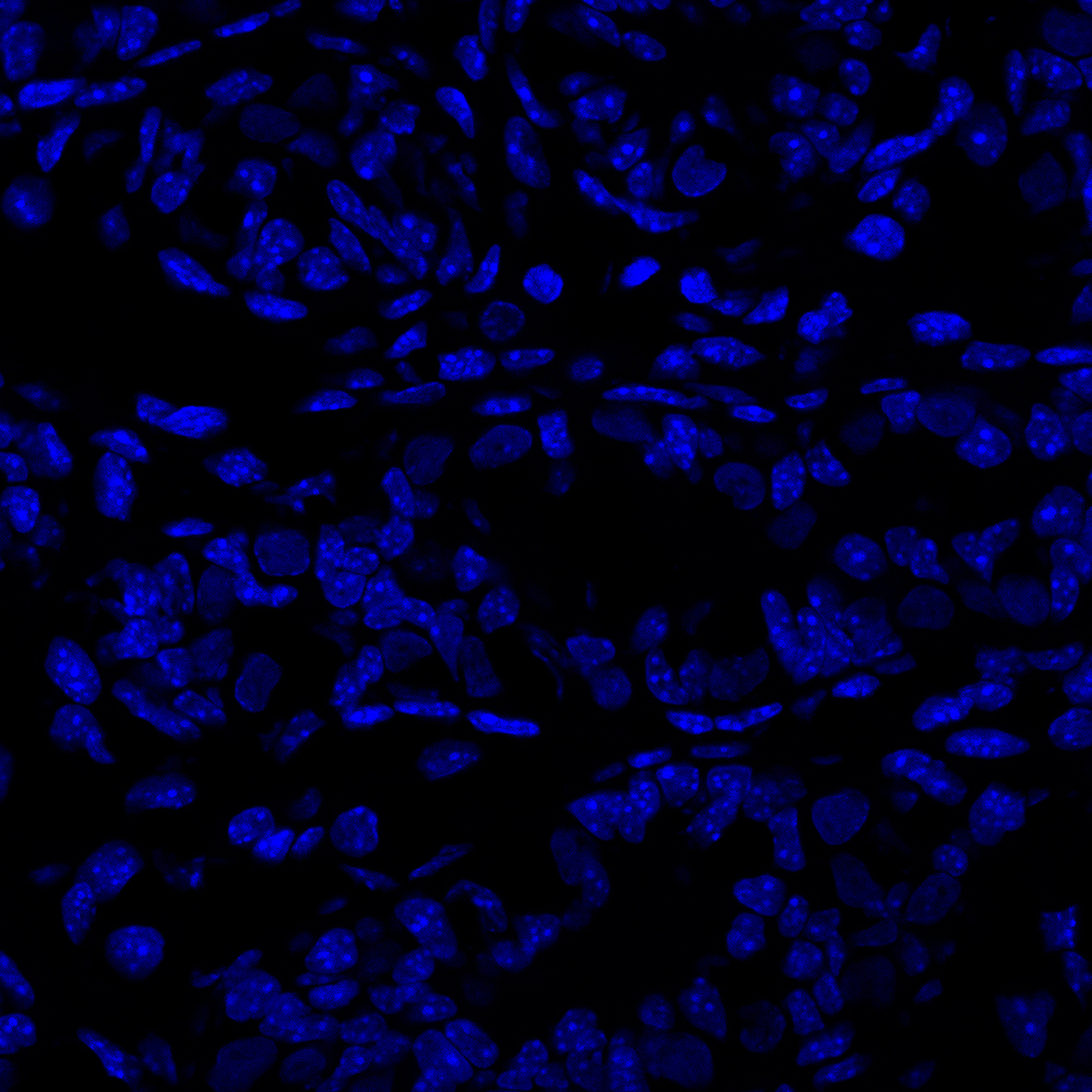

Supplement: Supplementary file 14 — EV and Appendix Figure Source Data [file 44318_2024_203_MOESM14_ESM.zip › Source Data for Expanded View and Appendix/Figure EV2/EV2B/P5-Ctrl-DAPI.jpg]

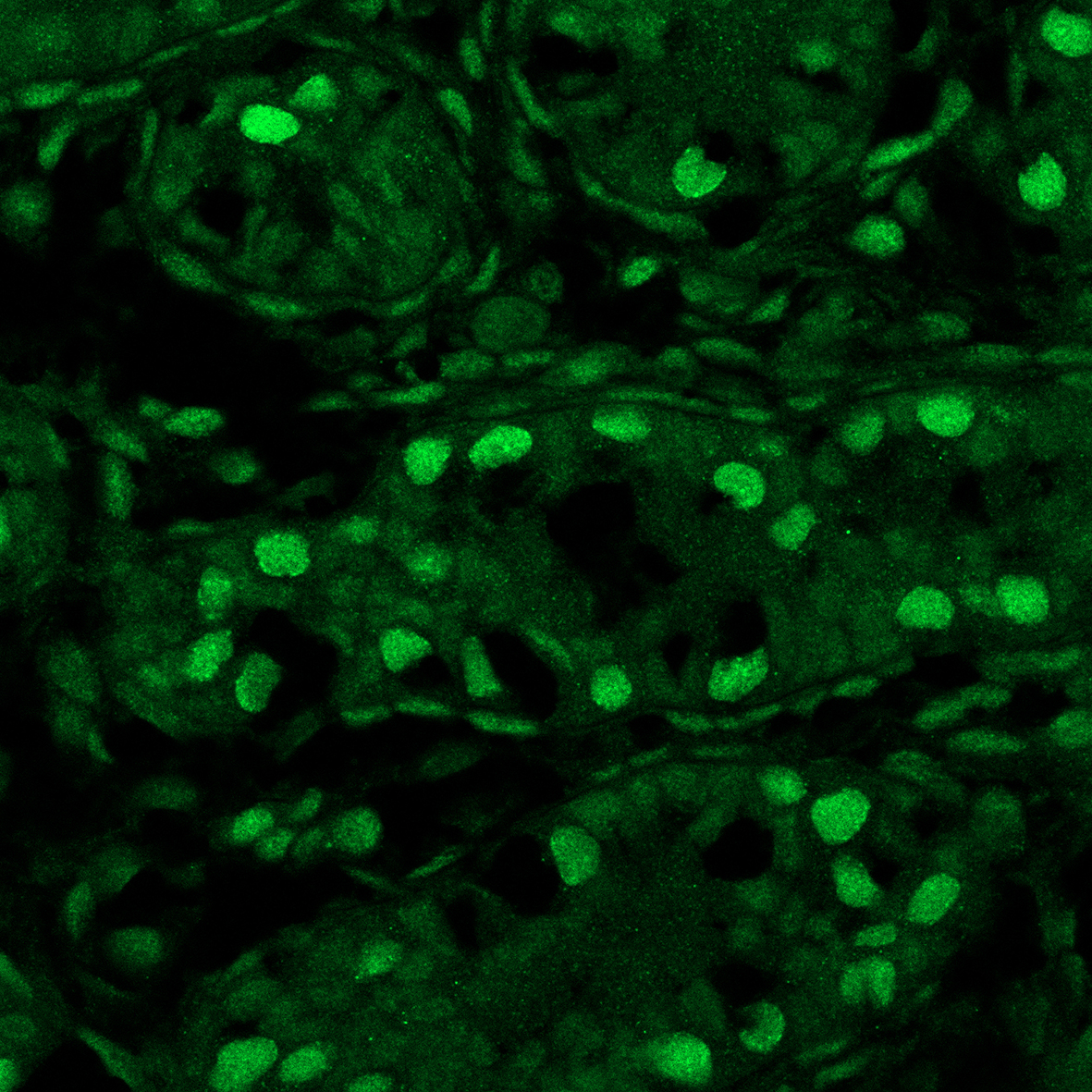

Supplement: Supplementary file 14 — EV and Appendix Figure Source Data [file 44318_2024_203_MOESM14_ESM.zip › Source Data for Expanded View and Appendix/Figure EV2/EV2B/P5-Ctrl-KDM2A.jpg]

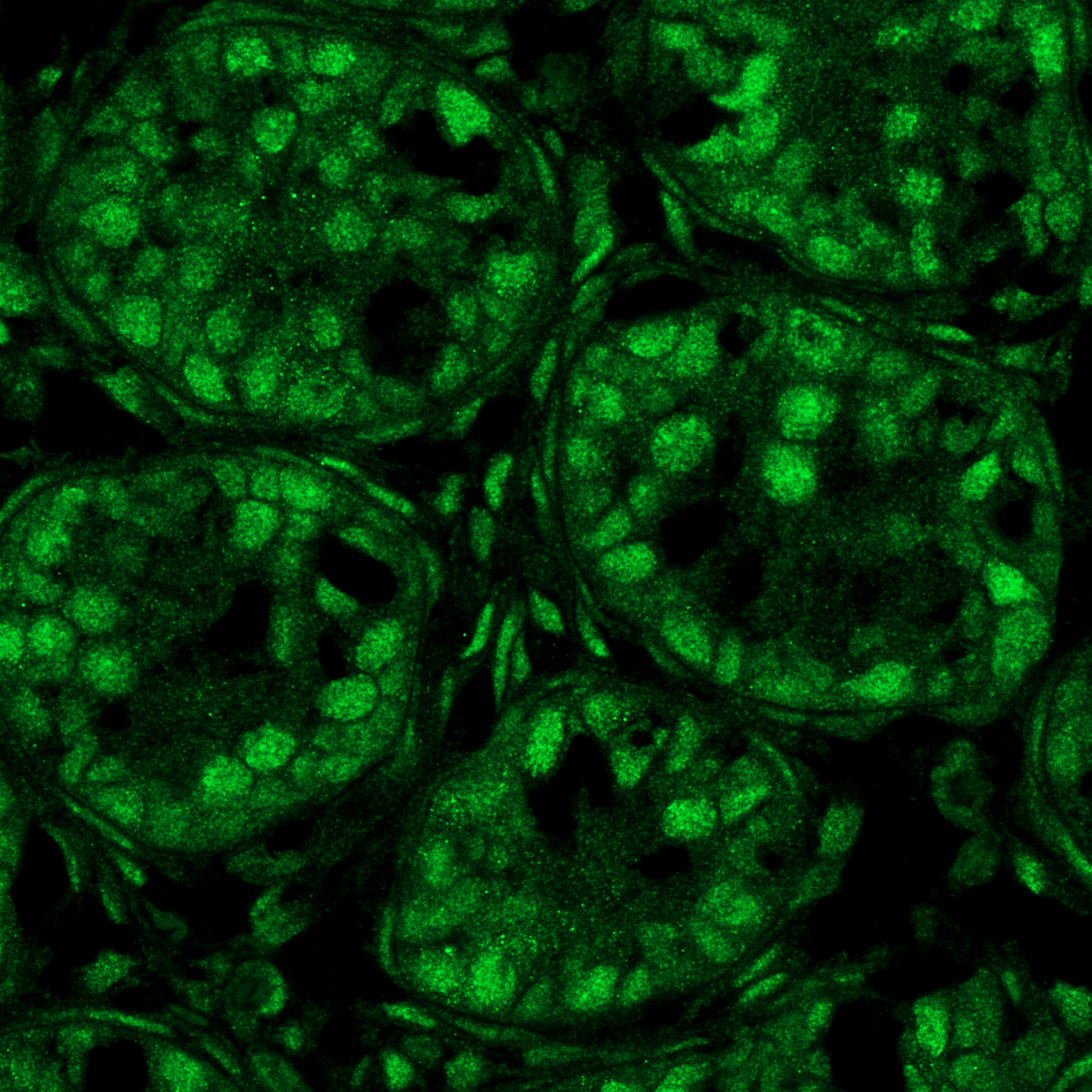

Supplement: Supplementary file 14 — EV and Appendix Figure Source Data [file 44318_2024_203_MOESM14_ESM.zip › Source Data for Expanded View and Appendix/Figure EV2/EV2B/P8-Ctrl-KDM2A.jpg]

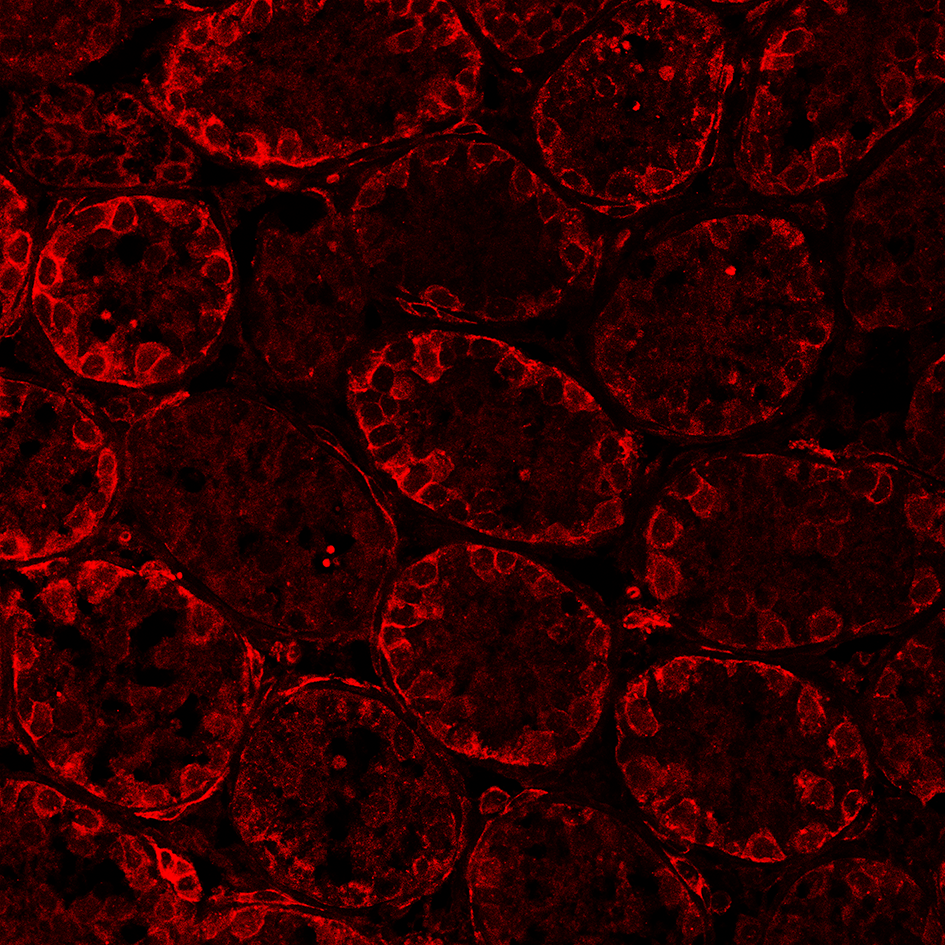

Supplement: Supplementary file 14 — EV and Appendix Figure Source Data [file 44318_2024_203_MOESM14_ESM.zip › Source Data for Expanded View and Appendix/Figure EV2/EV2D/cKO-cKIT.tif]

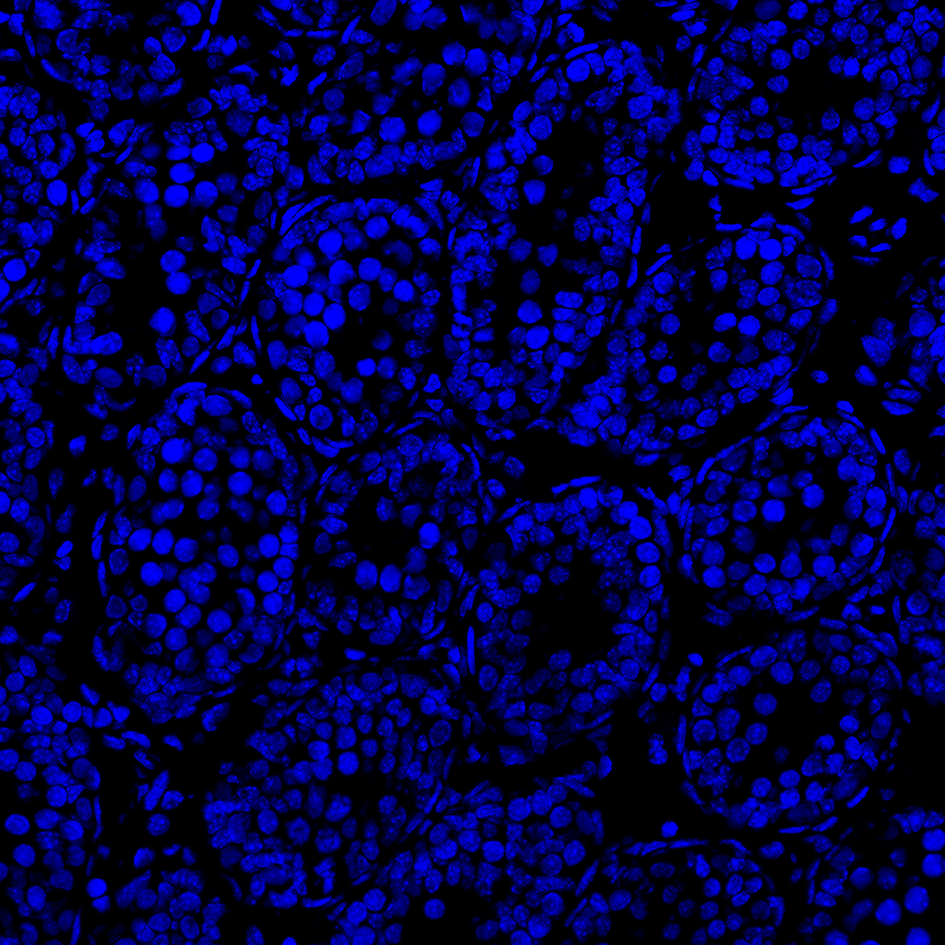

Supplement: Supplementary file 14 — EV and Appendix Figure Source Data [file 44318_2024_203_MOESM14_ESM.zip › Source Data for Expanded View and Appendix/Figure EV2/EV2D/Ctrl-DAPI.tif]

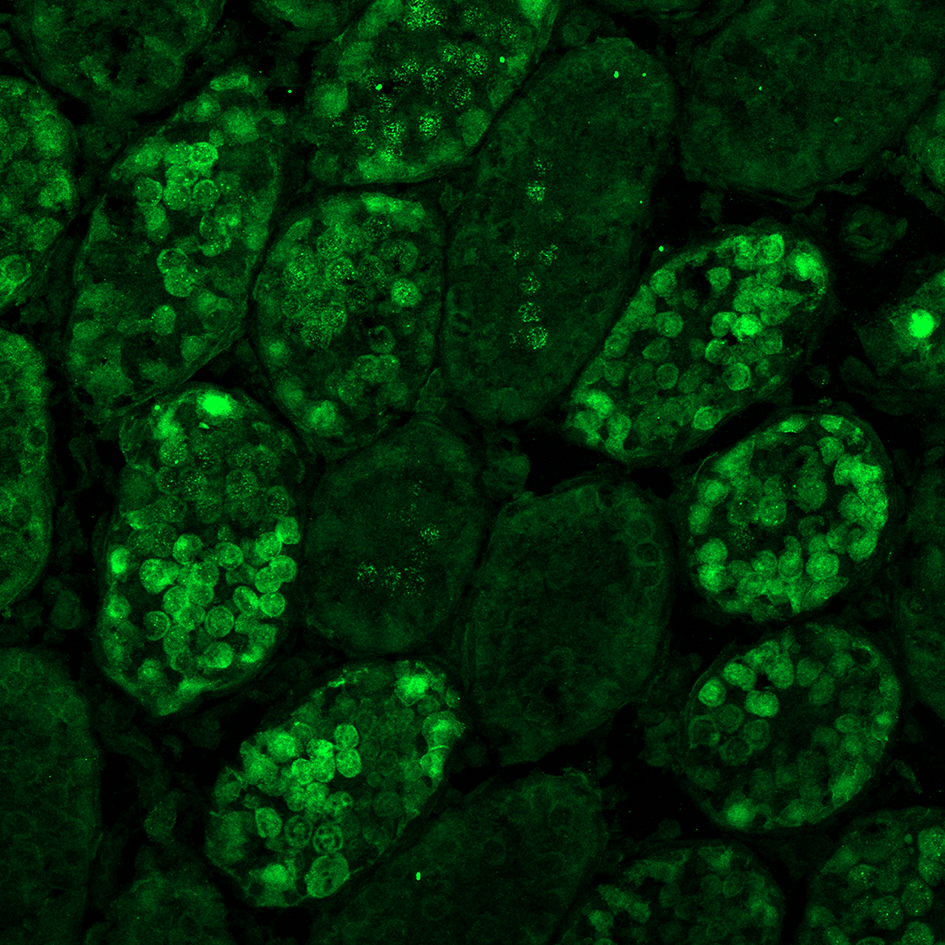

Supplement: Supplementary file 14 — EV and Appendix Figure Source Data [file 44318_2024_203_MOESM14_ESM.zip › Source Data for Expanded View and Appendix/Figure EV2/EV2D/Ctrl-STRA8.tif]

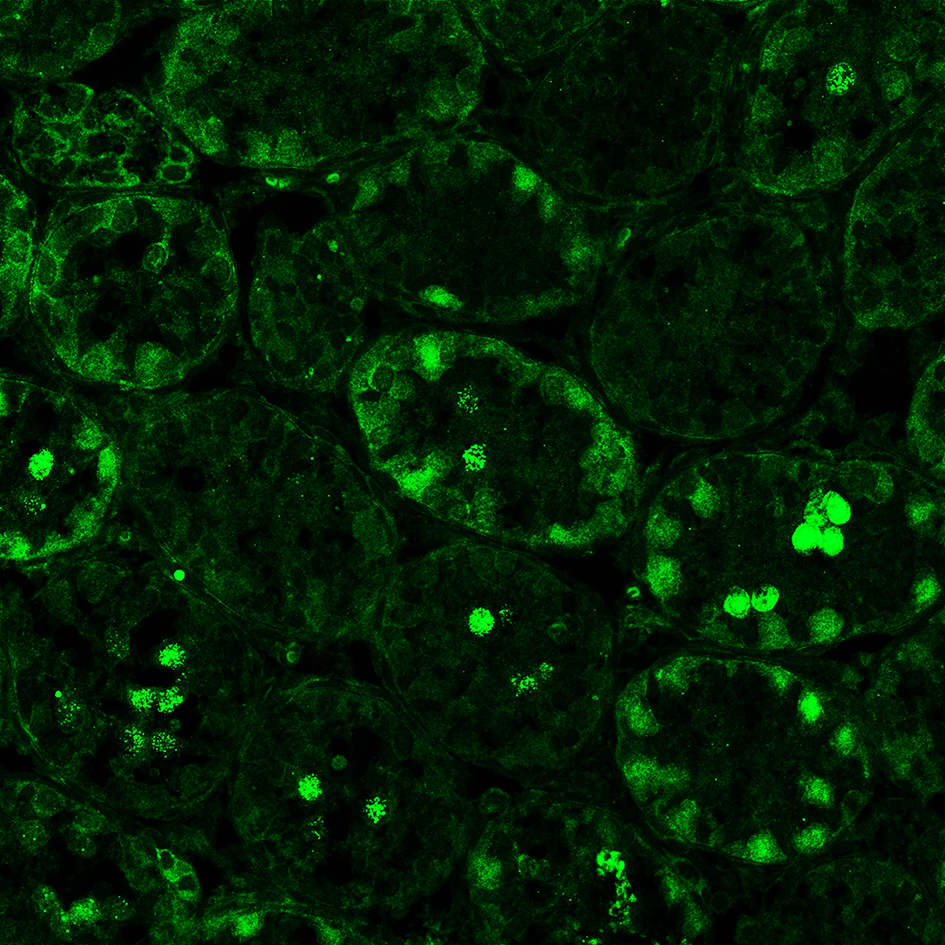

Supplement: Supplementary file 14 — EV and Appendix Figure Source Data [file 44318_2024_203_MOESM14_ESM.zip › Source Data for Expanded View and Appendix/Figure EV2/EV2D/cKO-STRA8.tif]

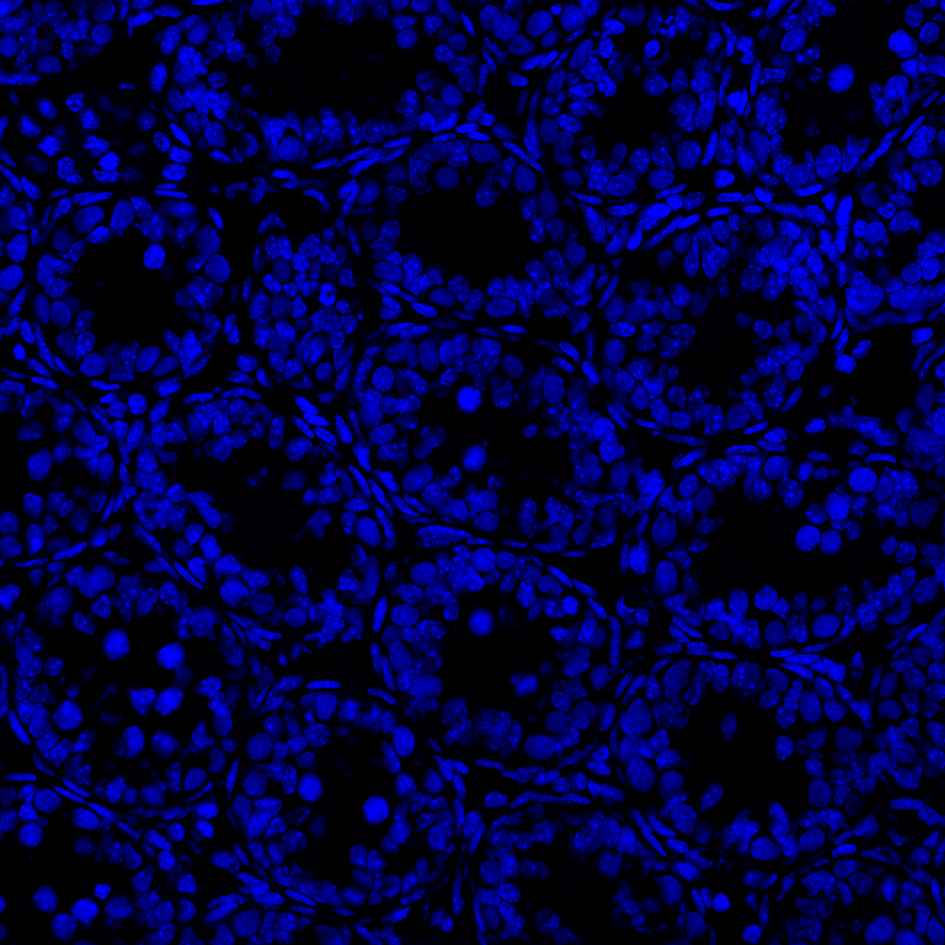

Supplement: Supplementary file 14 — EV and Appendix Figure Source Data [file 44318_2024_203_MOESM14_ESM.zip › Source Data for Expanded View and Appendix/Figure EV2/EV2D/cKO-DAPI.tif]

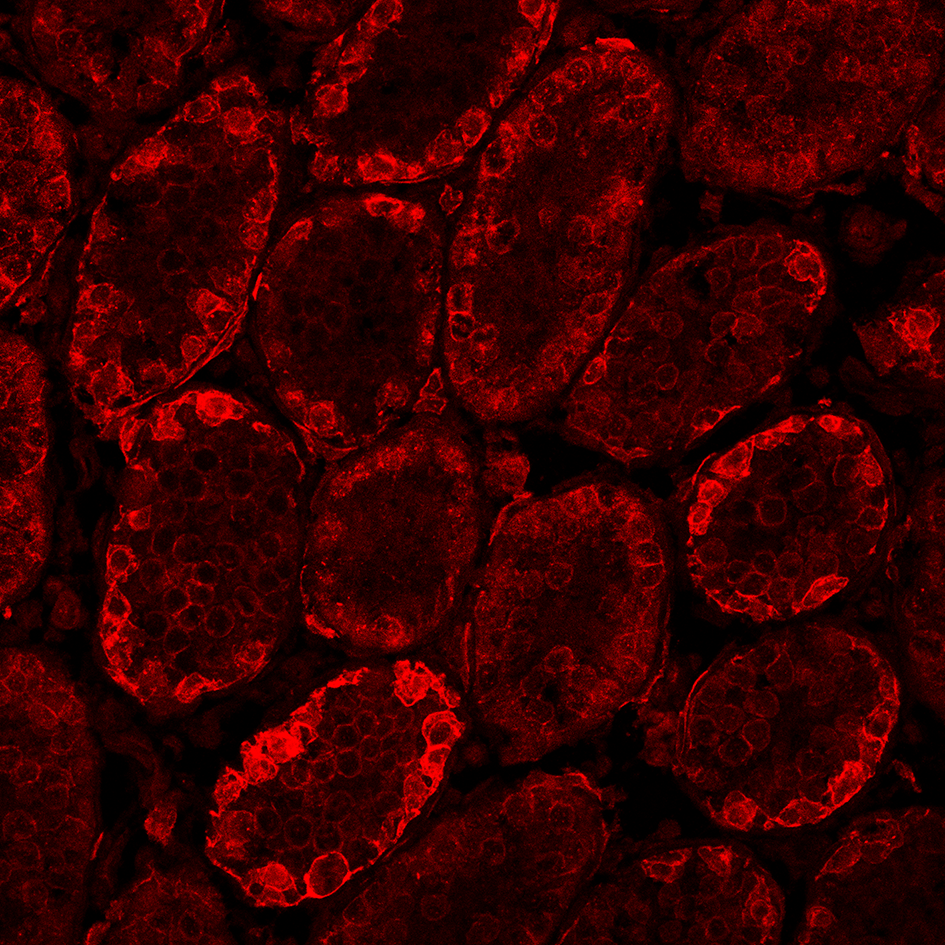

Supplement: Supplementary file 14 — EV and Appendix Figure Source Data [file 44318_2024_203_MOESM14_ESM.zip › Source Data for Expanded View and Appendix/Figure EV2/EV2D/Ctrl-cKIT.tif]

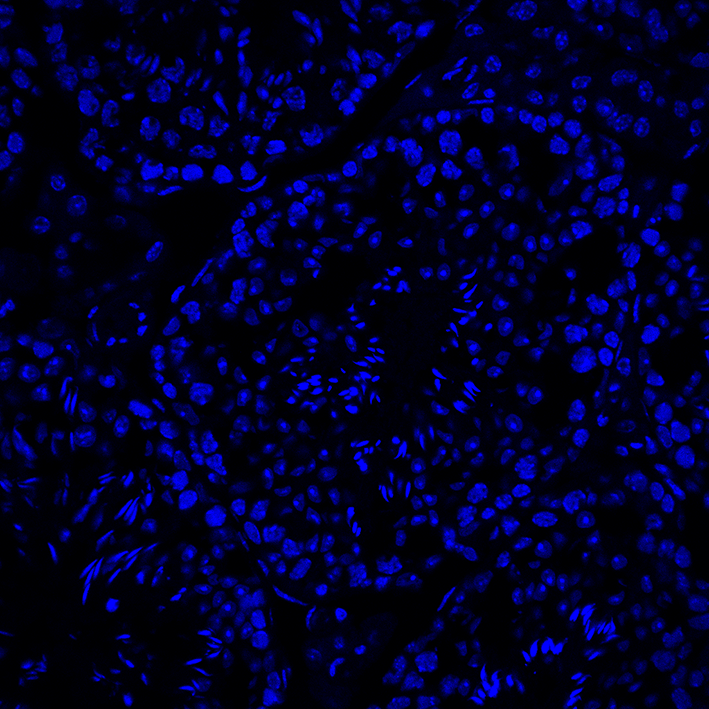

Supplement: Supplementary file 14 — EV and Appendix Figure Source Data [file 44318_2024_203_MOESM14_ESM.zip › Source Data for Expanded View and Appendix/Figure EV2/EV2F/Ctrl-DAPI.tif]

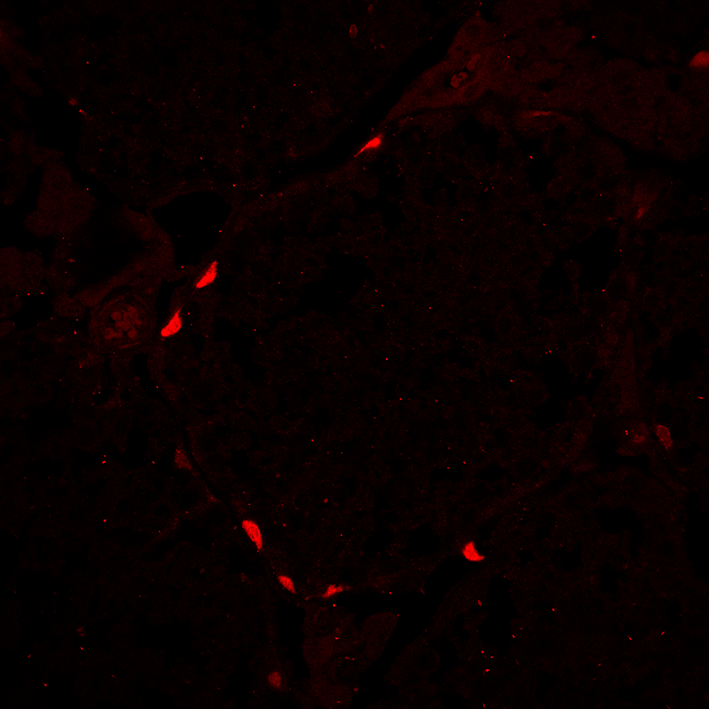

Supplement: Supplementary file 14 — EV and Appendix Figure Source Data [file 44318_2024_203_MOESM14_ESM.zip › Source Data for Expanded View and Appendix/Figure EV2/EV2F/Ctrl-PLZF.tif]

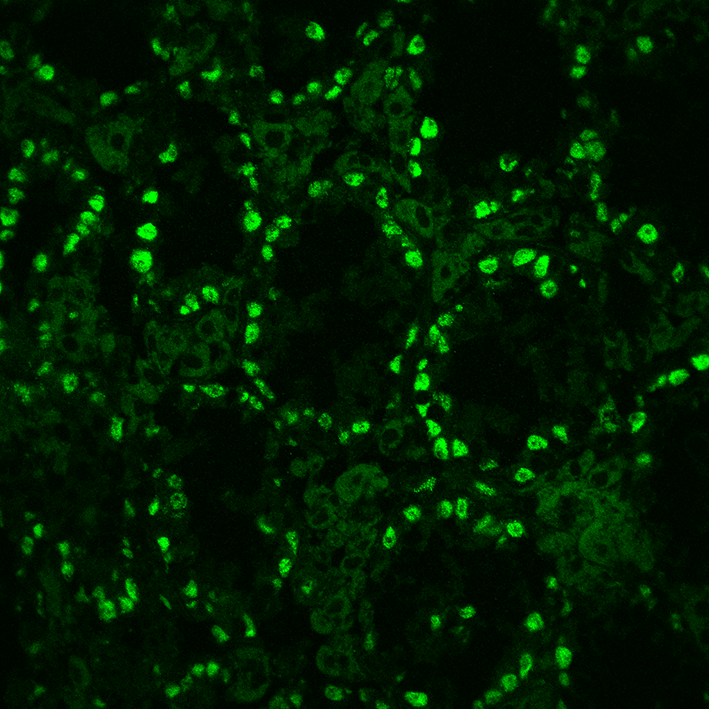

Supplement: Supplementary file 14 — EV and Appendix Figure Source Data [file 44318_2024_203_MOESM14_ESM.zip › Source Data for Expanded View and Appendix/Figure EV2/EV2F/cKO-WT1.tif]

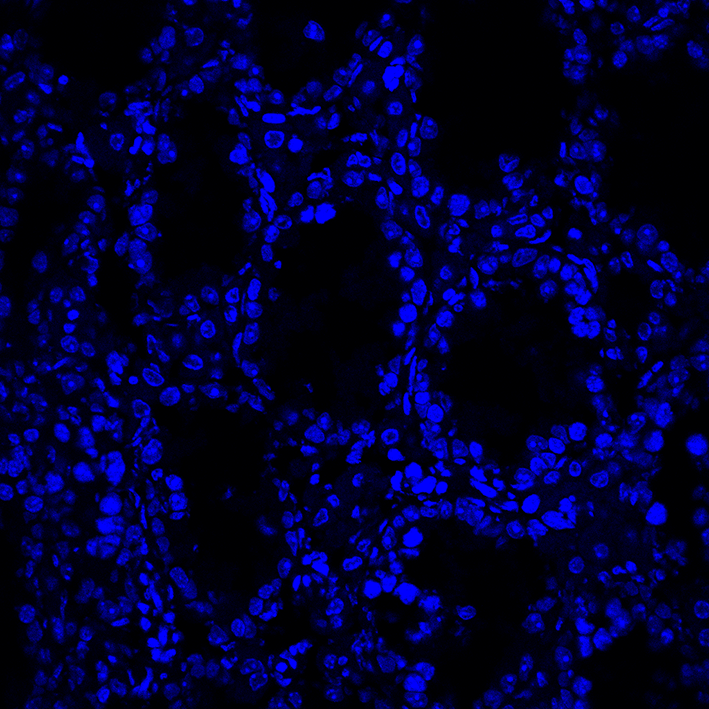

Supplement: Supplementary file 14 — EV and Appendix Figure Source Data [file 44318_2024_203_MOESM14_ESM.zip › Source Data for Expanded View and Appendix/Figure EV2/EV2F/cKO-DAPI.tif]

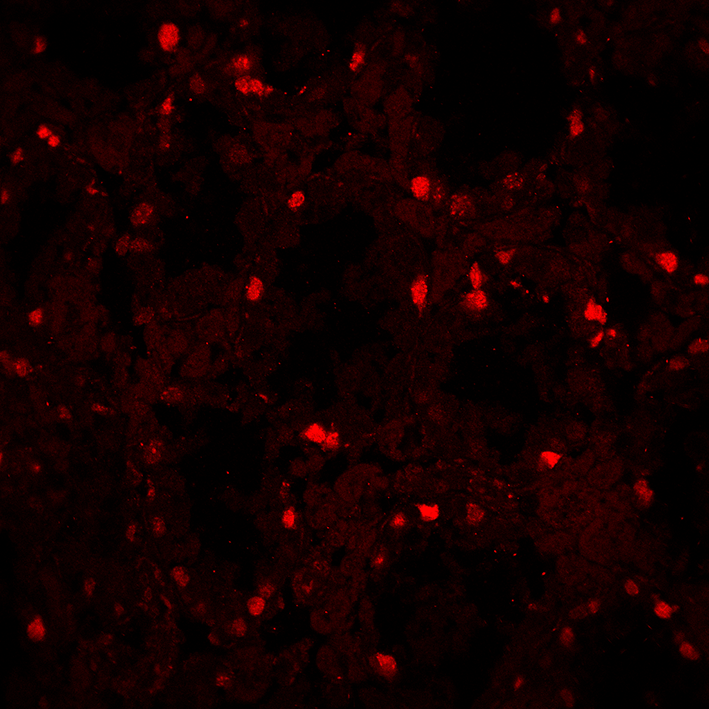

Supplement: Supplementary file 14 — EV and Appendix Figure Source Data [file 44318_2024_203_MOESM14_ESM.zip › Source Data for Expanded View and Appendix/Figure EV2/EV2F/cKO-PLZF.tif]

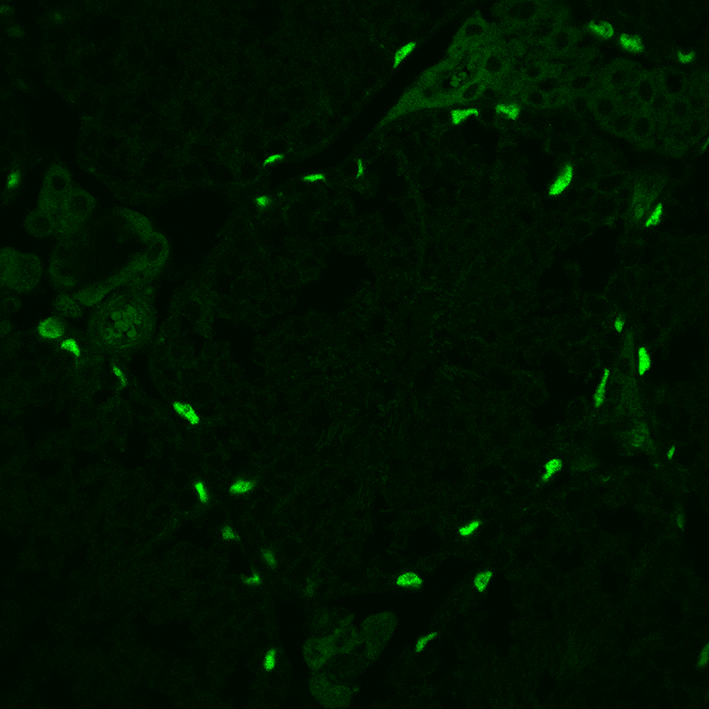

Supplement: Supplementary file 14 — EV and Appendix Figure Source Data [file 44318_2024_203_MOESM14_ESM.zip › Source Data for Expanded View and Appendix/Figure EV2/EV2F/Ctrl-WT1.tif]

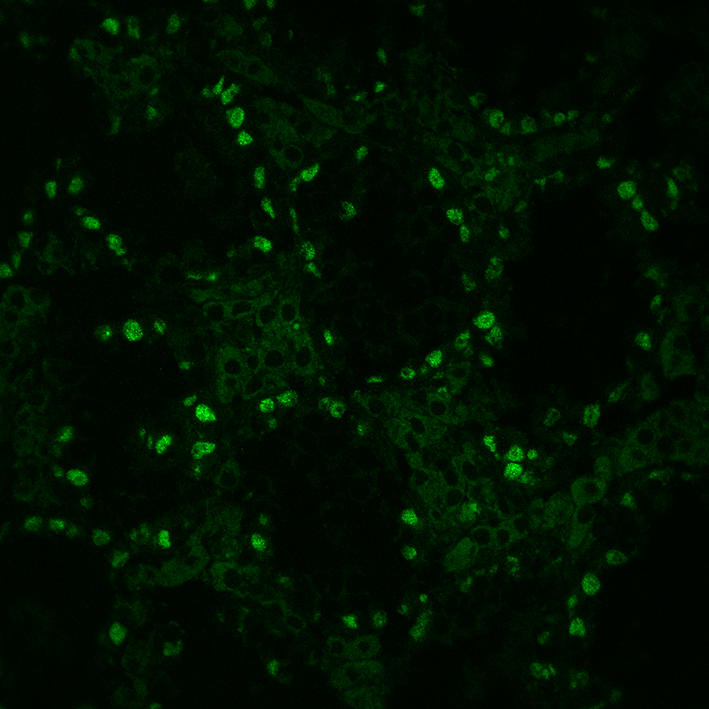

Supplement: Supplementary file 14 — EV and Appendix Figure Source Data [file 44318_2024_203_MOESM14_ESM.zip › Source Data for Expanded View and Appendix/Figure EV2/EV2H/cKO -WT1.tif]

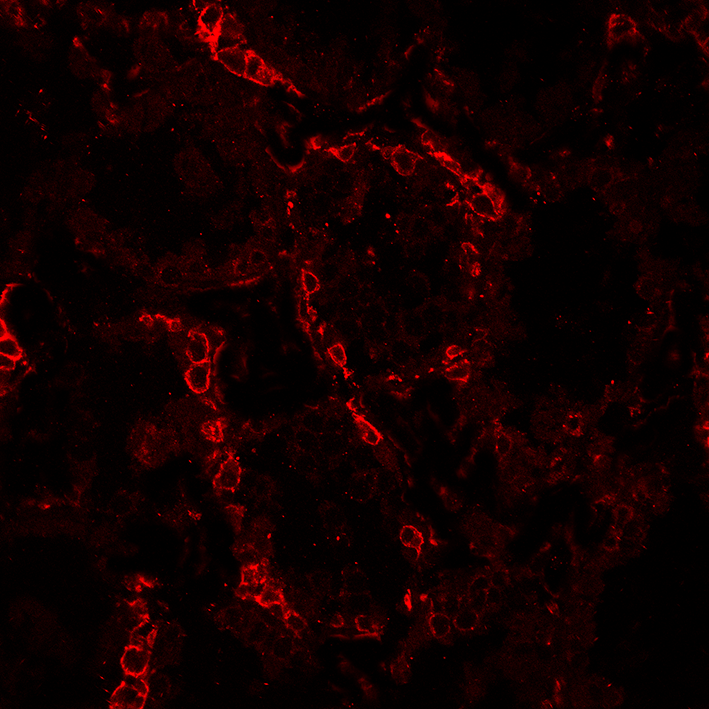

Supplement: Supplementary file 14 — EV and Appendix Figure Source Data [file 44318_2024_203_MOESM14_ESM.zip › Source Data for Expanded View and Appendix/Figure EV2/EV2H/cKO-cKIT.tif]

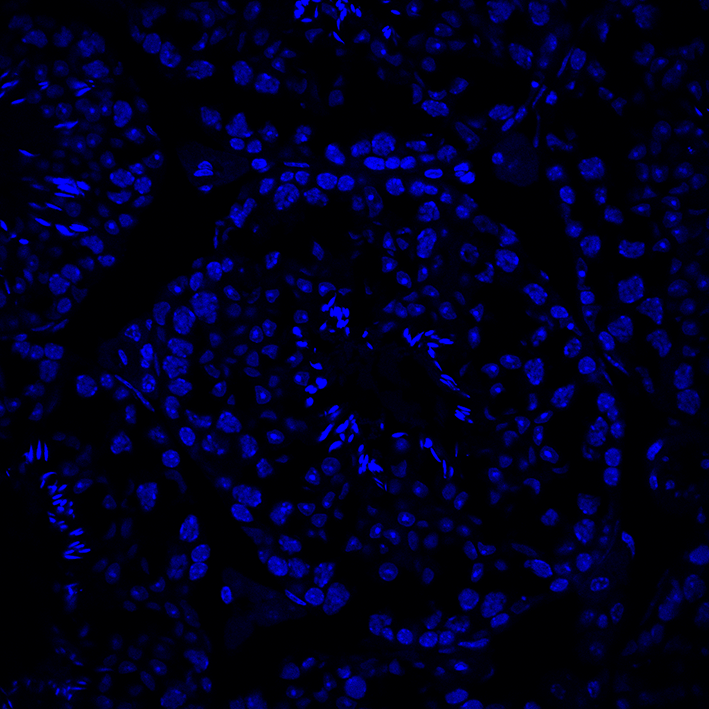

Supplement: Supplementary file 14 — EV and Appendix Figure Source Data [file 44318_2024_203_MOESM14_ESM.zip › Source Data for Expanded View and Appendix/Figure EV2/EV2H/Ctrl-DAPI.tif]

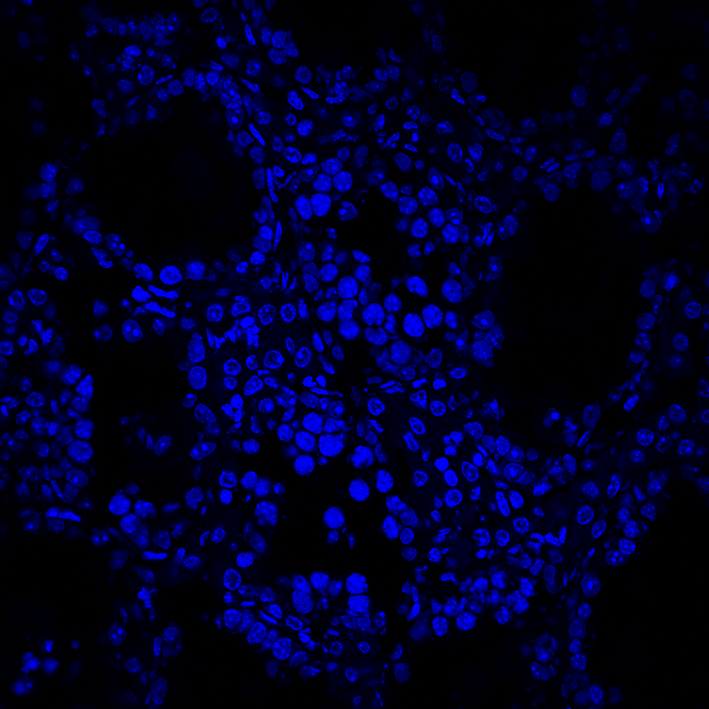

Supplement: Supplementary file 14 — EV and Appendix Figure Source Data [file 44318_2024_203_MOESM14_ESM.zip › Source Data for Expanded View and Appendix/Figure EV2/EV2H/cKO-DAPI.tif]

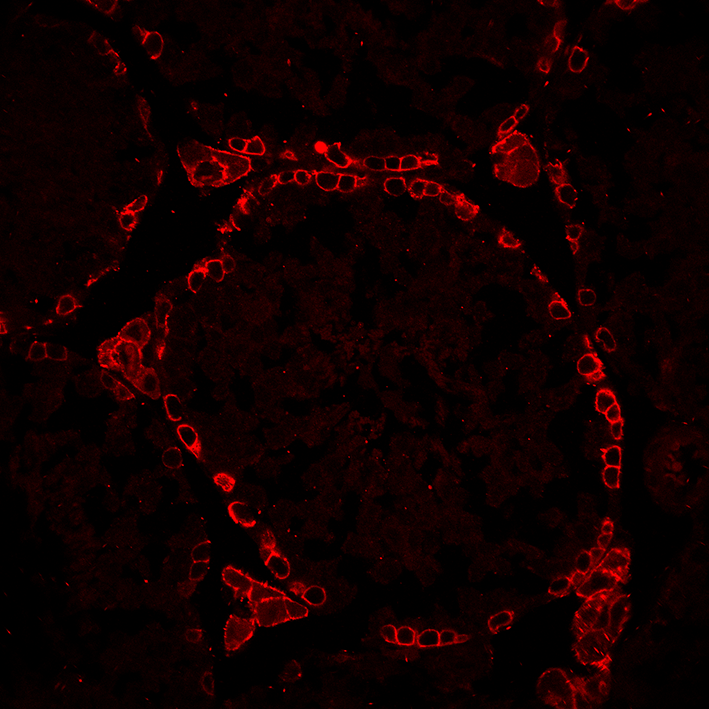

Supplement: Supplementary file 14 — EV and Appendix Figure Source Data [file 44318_2024_203_MOESM14_ESM.zip › Source Data for Expanded View and Appendix/Figure EV2/EV2H/Ctrl-cKIT.tif]

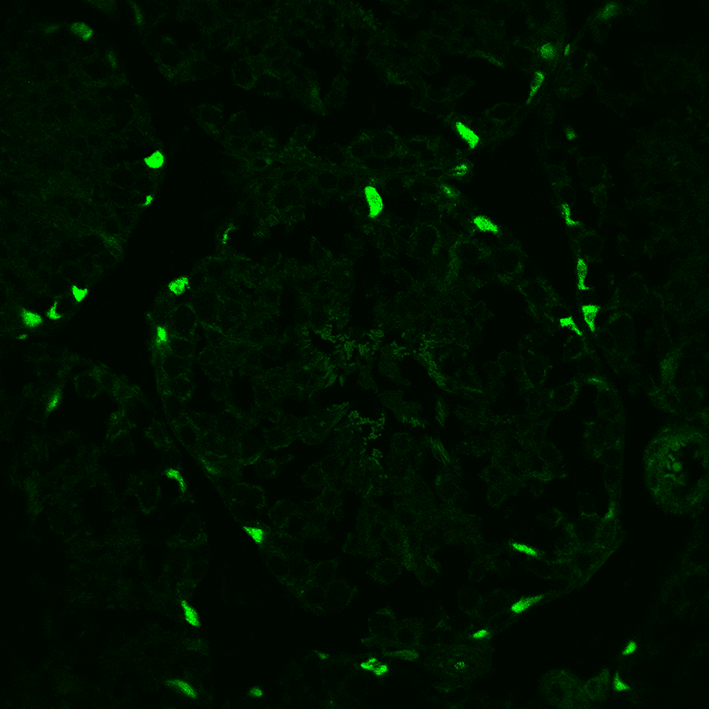

Supplement: Supplementary file 14 — EV and Appendix Figure Source Data [file 44318_2024_203_MOESM14_ESM.zip › Source Data for Expanded View and Appendix/Figure EV2/EV2H/Ctrl-WT1.tif]

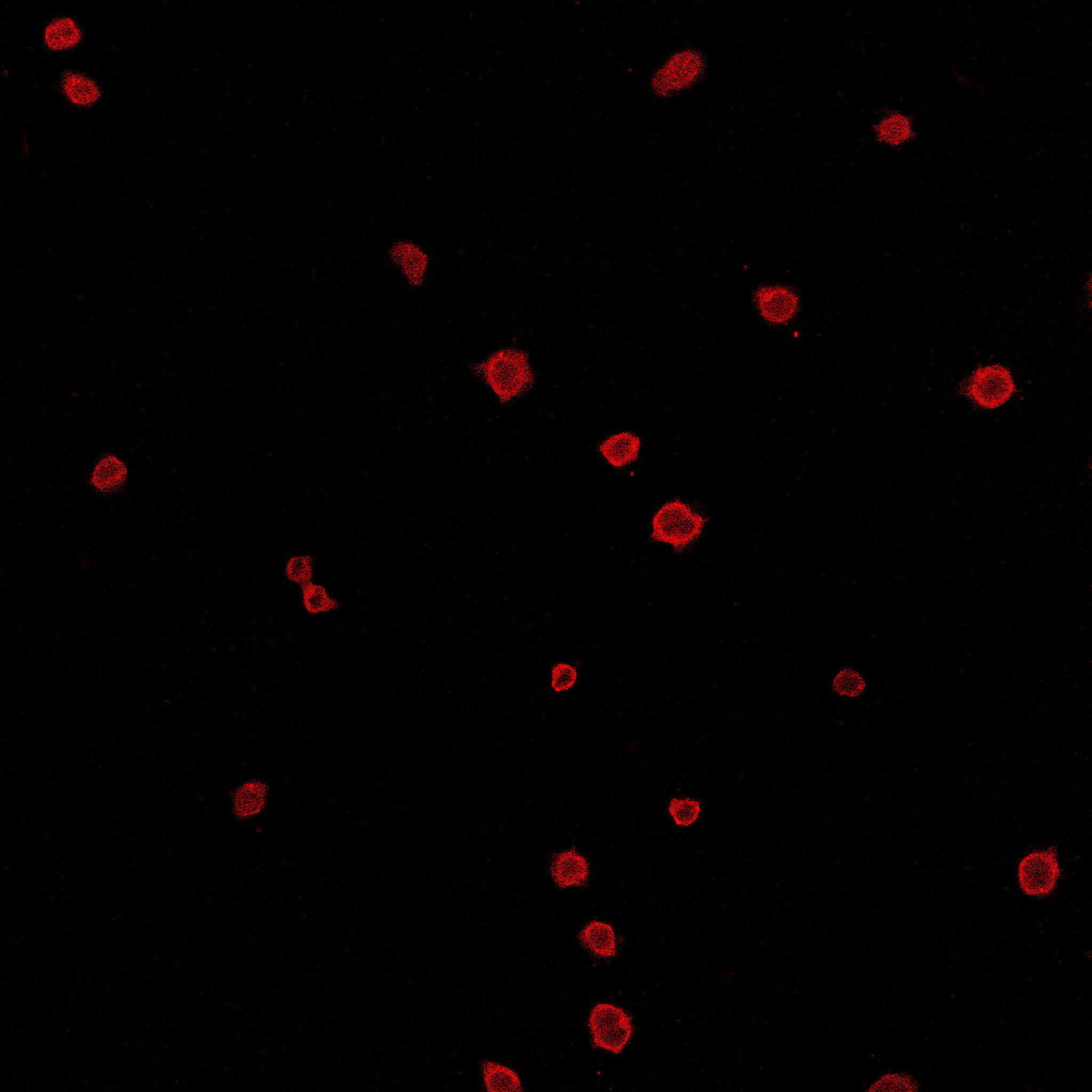

Supplement: Supplementary file 14 — EV and Appendix Figure Source Data [file 44318_2024_203_MOESM14_ESM.zip › Source Data for Expanded View and Appendix/Figure EV5/EV5B/cKO - c-KIT.jpg]

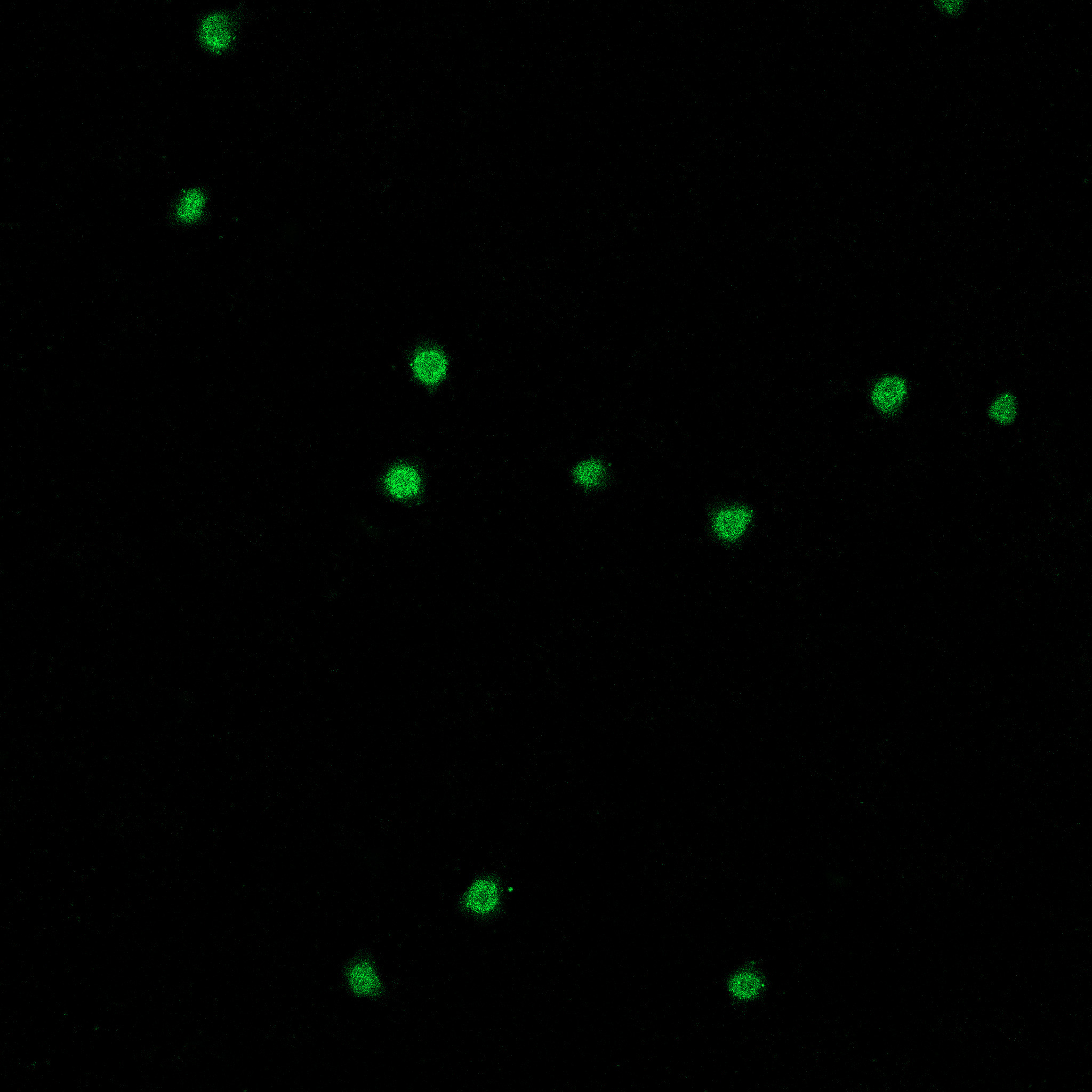

Supplement: Supplementary file 14 — EV and Appendix Figure Source Data [file 44318_2024_203_MOESM14_ESM.zip › Source Data for Expanded View and Appendix/Figure EV5/EV5B/Control-STRA8.jpg]

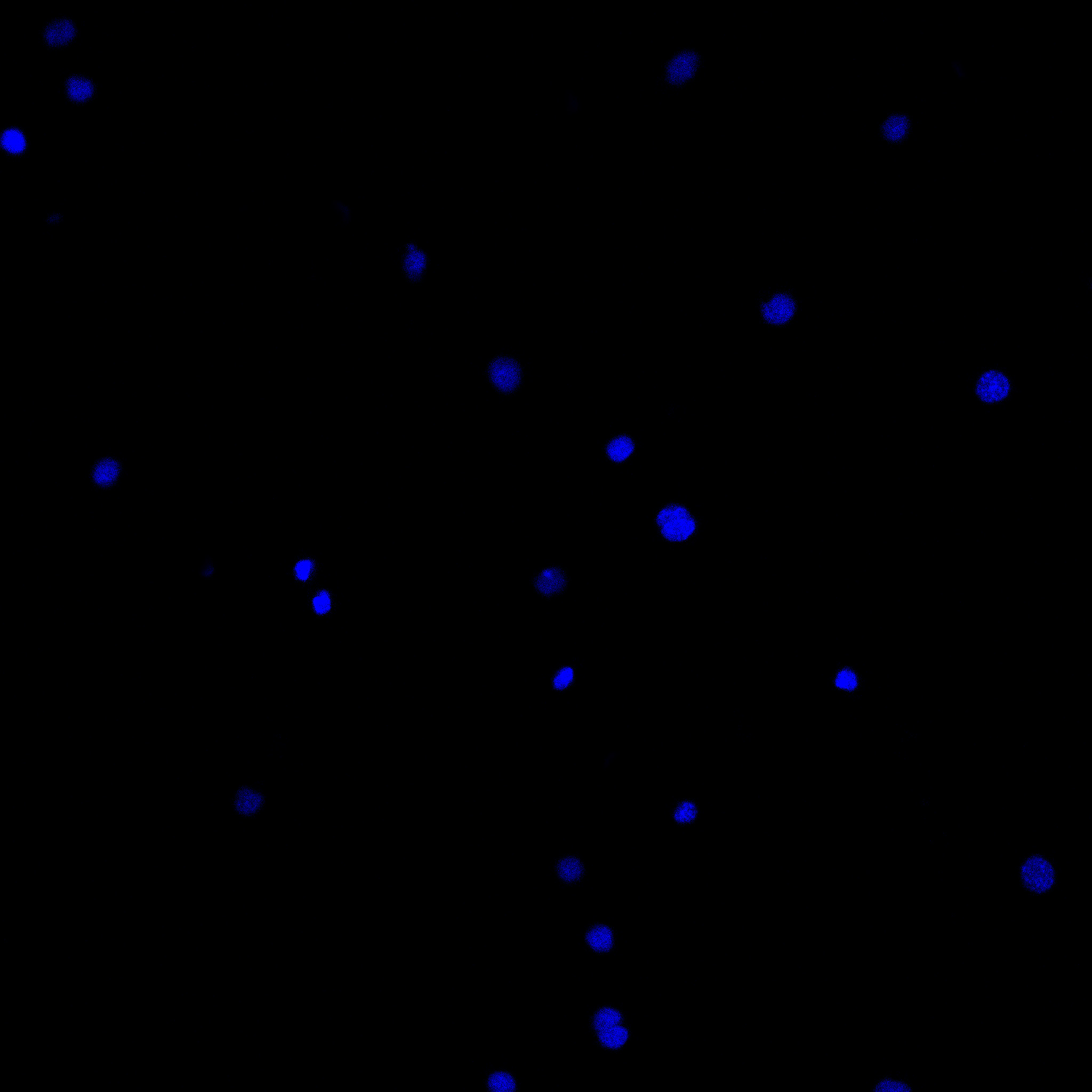

Supplement: Supplementary file 14 — EV and Appendix Figure Source Data [file 44318_2024_203_MOESM14_ESM.zip › Source Data for Expanded View and Appendix/Figure EV5/EV5B/cKO-DAPI.jpg]

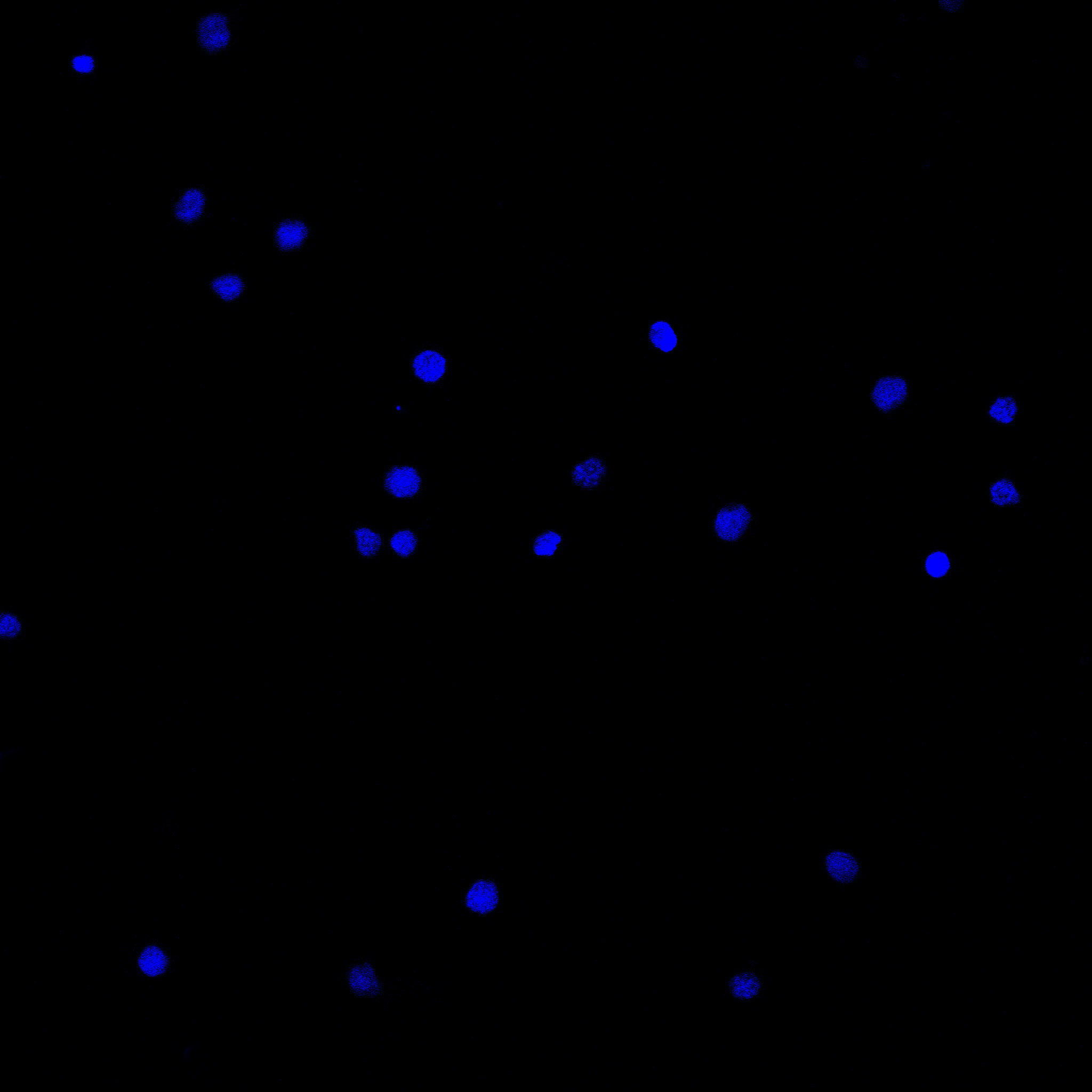

Supplement: Supplementary file 14 — EV and Appendix Figure Source Data [file 44318_2024_203_MOESM14_ESM.zip › Source Data for Expanded View and Appendix/Figure EV5/EV5B/Control-DAPI.jpg]

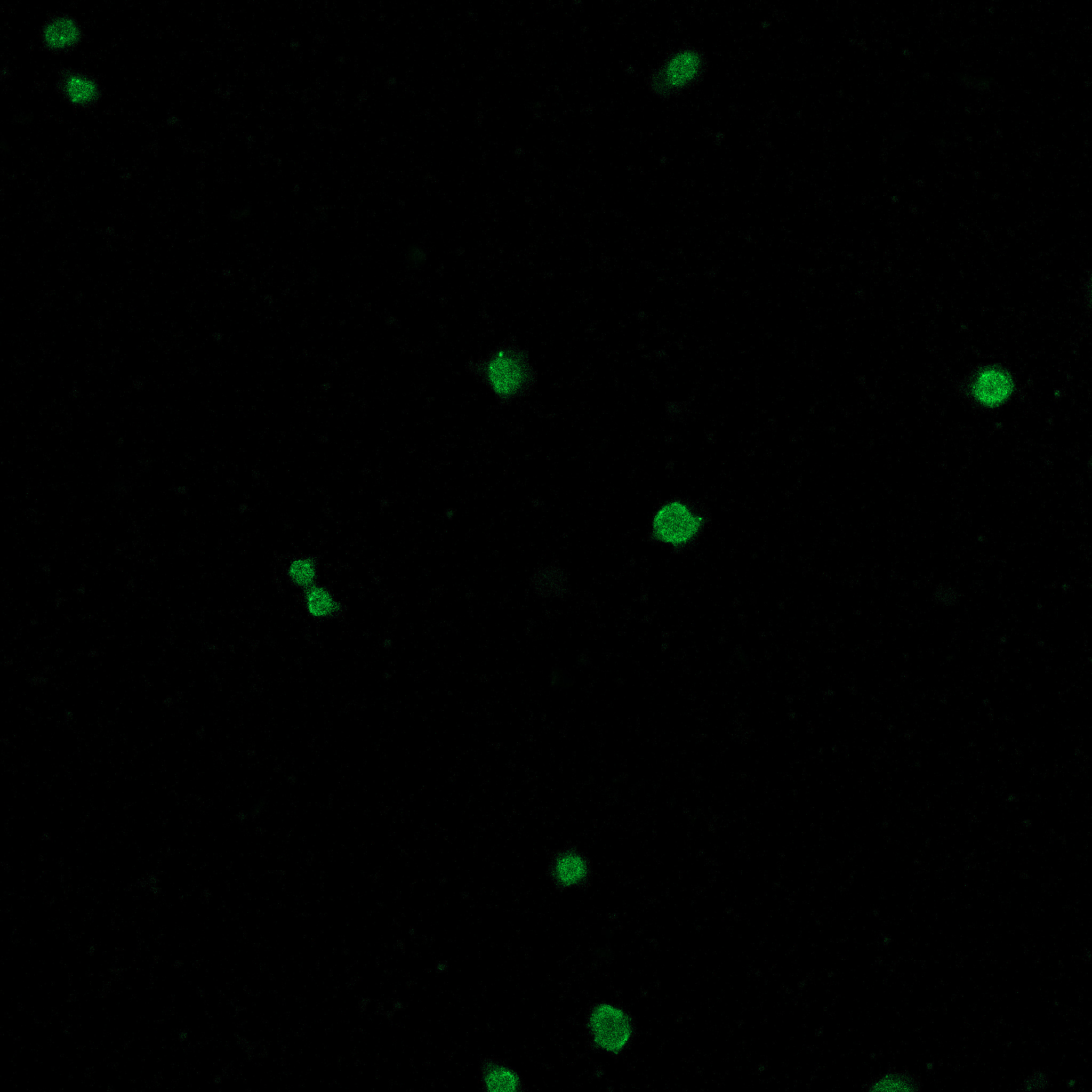

Supplement: Supplementary file 14 — EV and Appendix Figure Source Data [file 44318_2024_203_MOESM14_ESM.zip › Source Data for Expanded View and Appendix/Figure EV5/EV5B/cKO-STRA8.jpg]

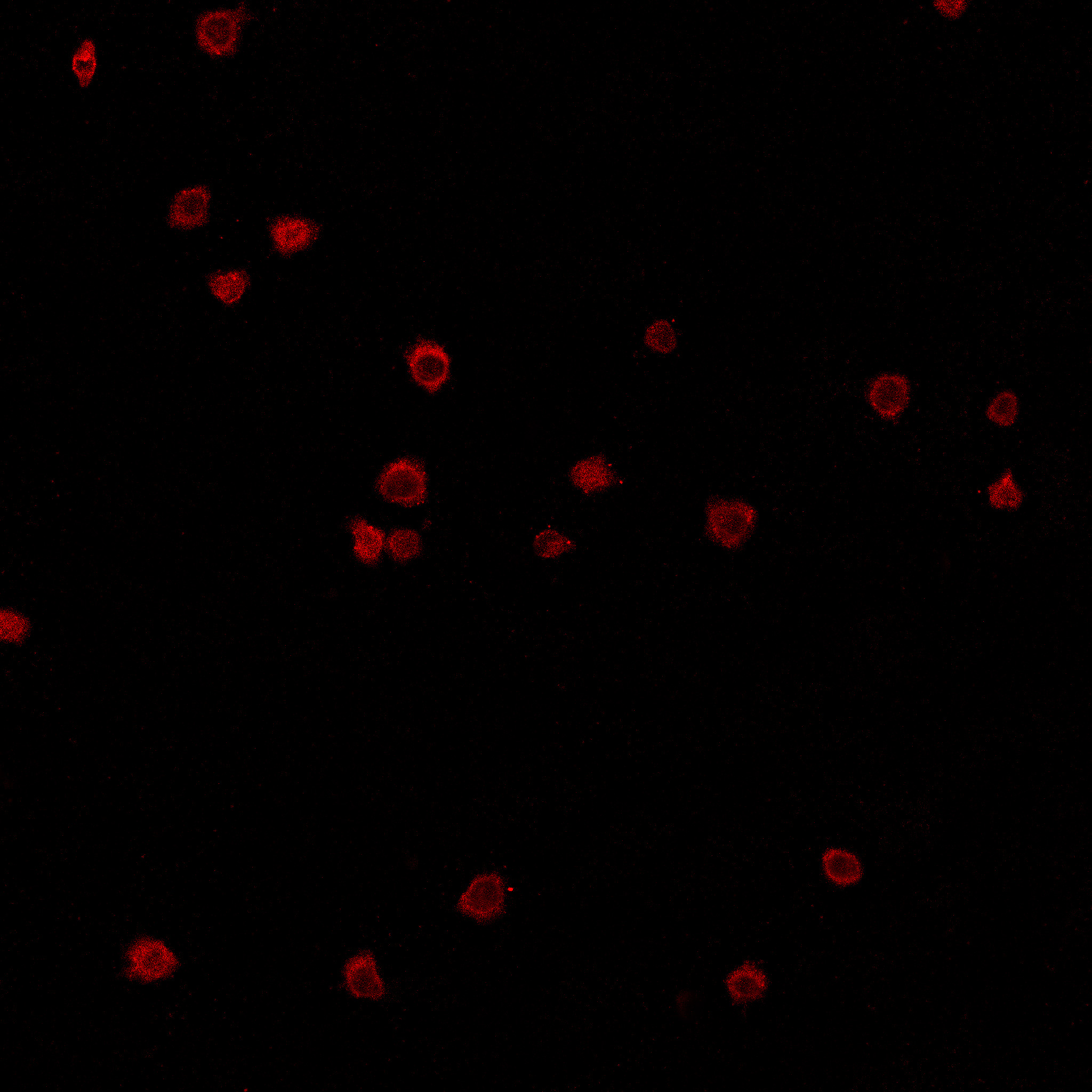

Supplement: Supplementary file 14 — EV and Appendix Figure Source Data [file 44318_2024_203_MOESM14_ESM.zip › Source Data for Expanded View and Appendix/Figure EV5/EV5B/Control-c-KIT.jpg]

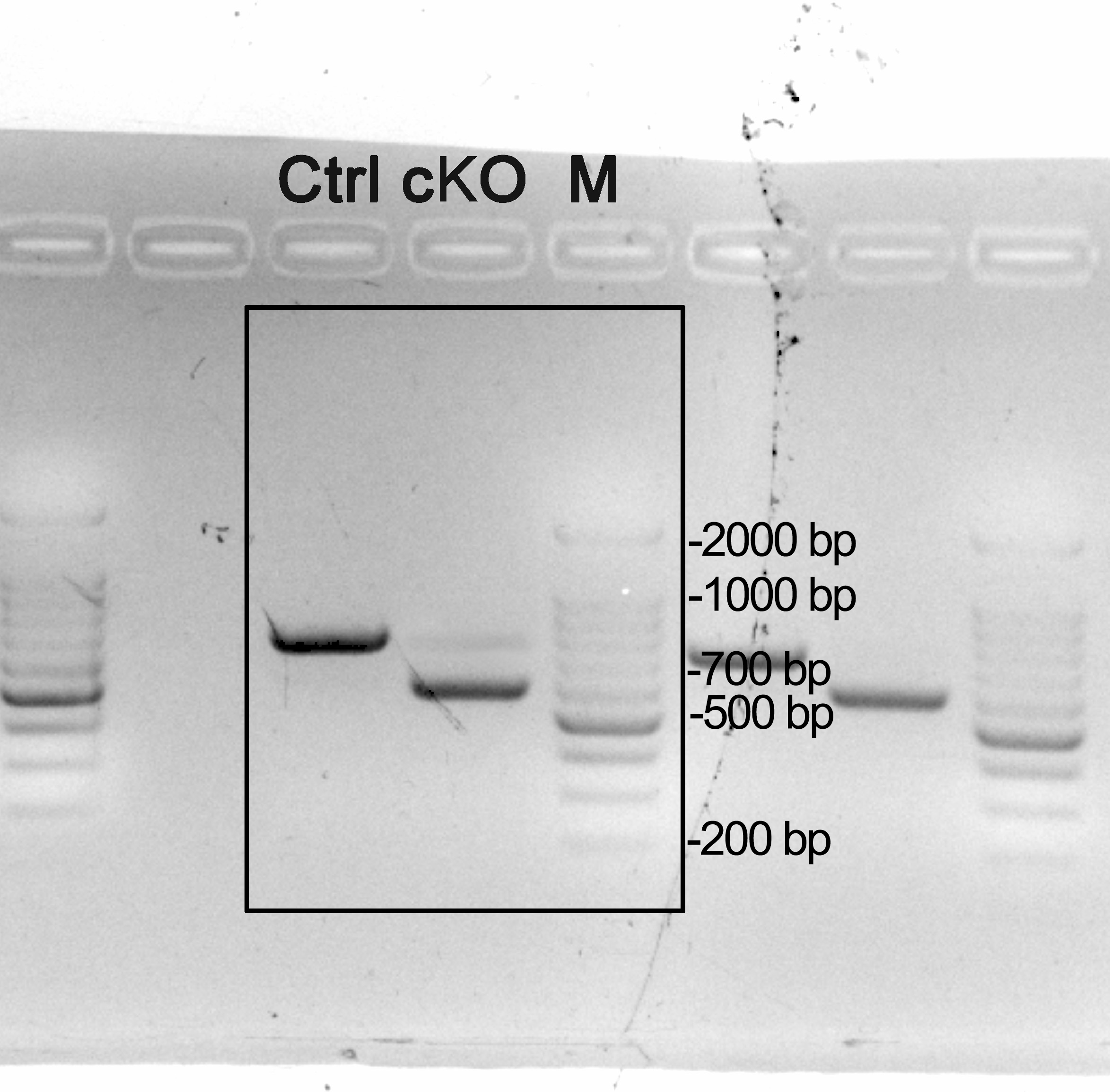

Supplement: Supplementary file 14 — EV and Appendix Figure Source Data [file 44318_2024_203_MOESM14_ESM.zip › Source Data for Expanded View and Appendix/Figure EV5/EV5F/RT-PCR KDM2A.jpg]

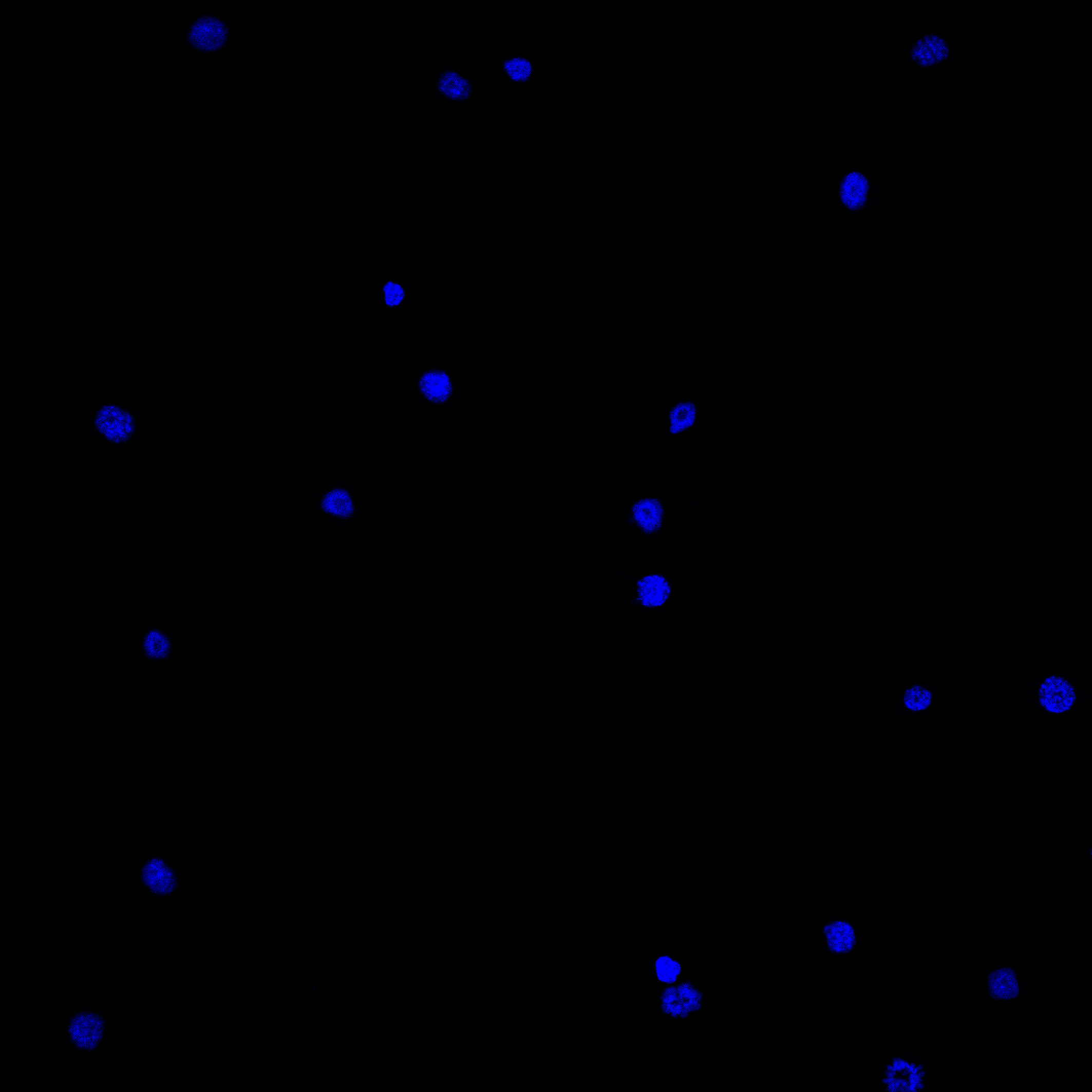

Supplement: Supplementary file 14 — EV and Appendix Figure Source Data [file 44318_2024_203_MOESM14_ESM.zip › Source Data for Expanded View and Appendix/Figure EV5/EV5A/Ctrl-DAPI.jpg]

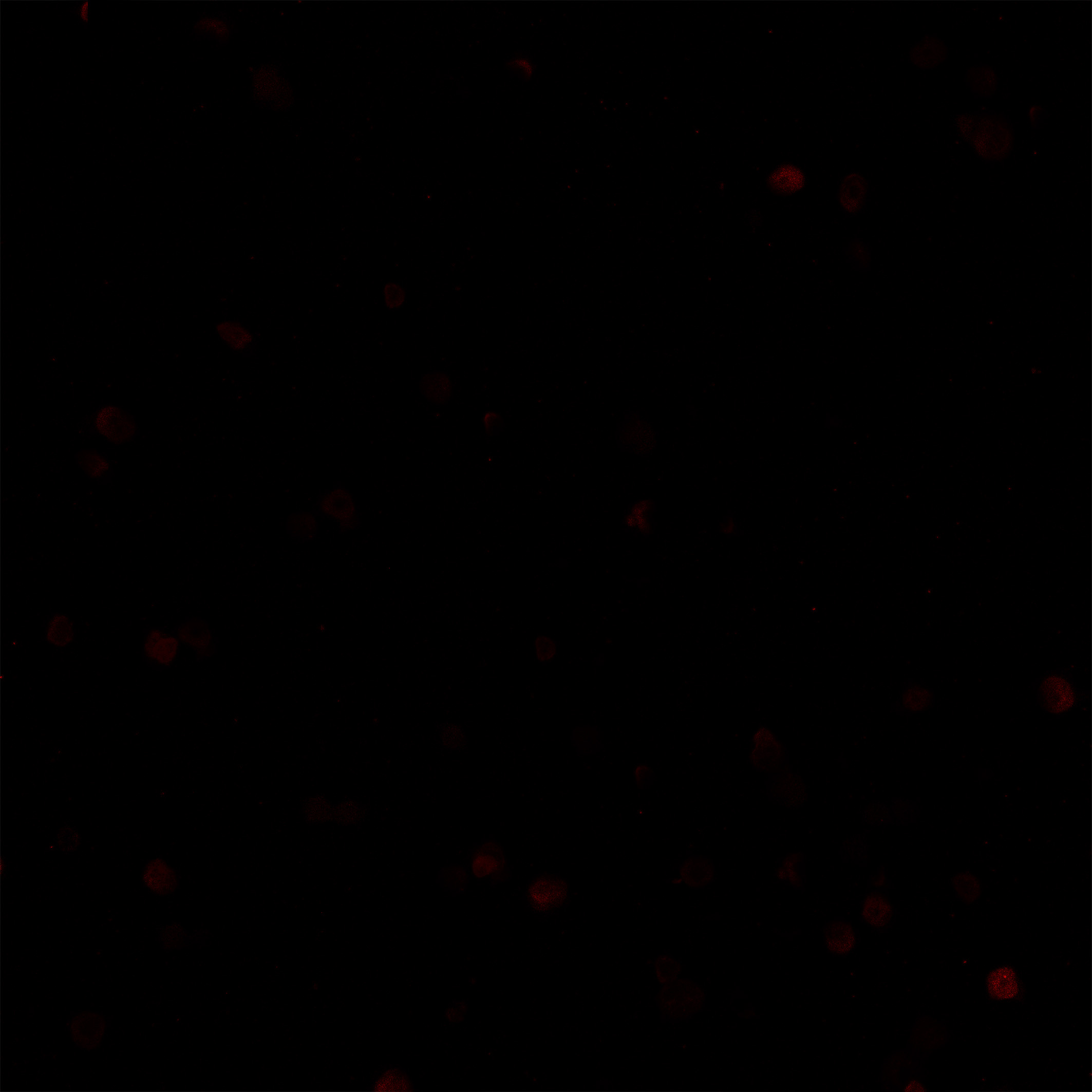

Supplement: Supplementary file 14 — EV and Appendix Figure Source Data [file 44318_2024_203_MOESM14_ESM.zip › Source Data for Expanded View and Appendix/Figure EV5/EV5A/cKO-KDM2A.jpg]

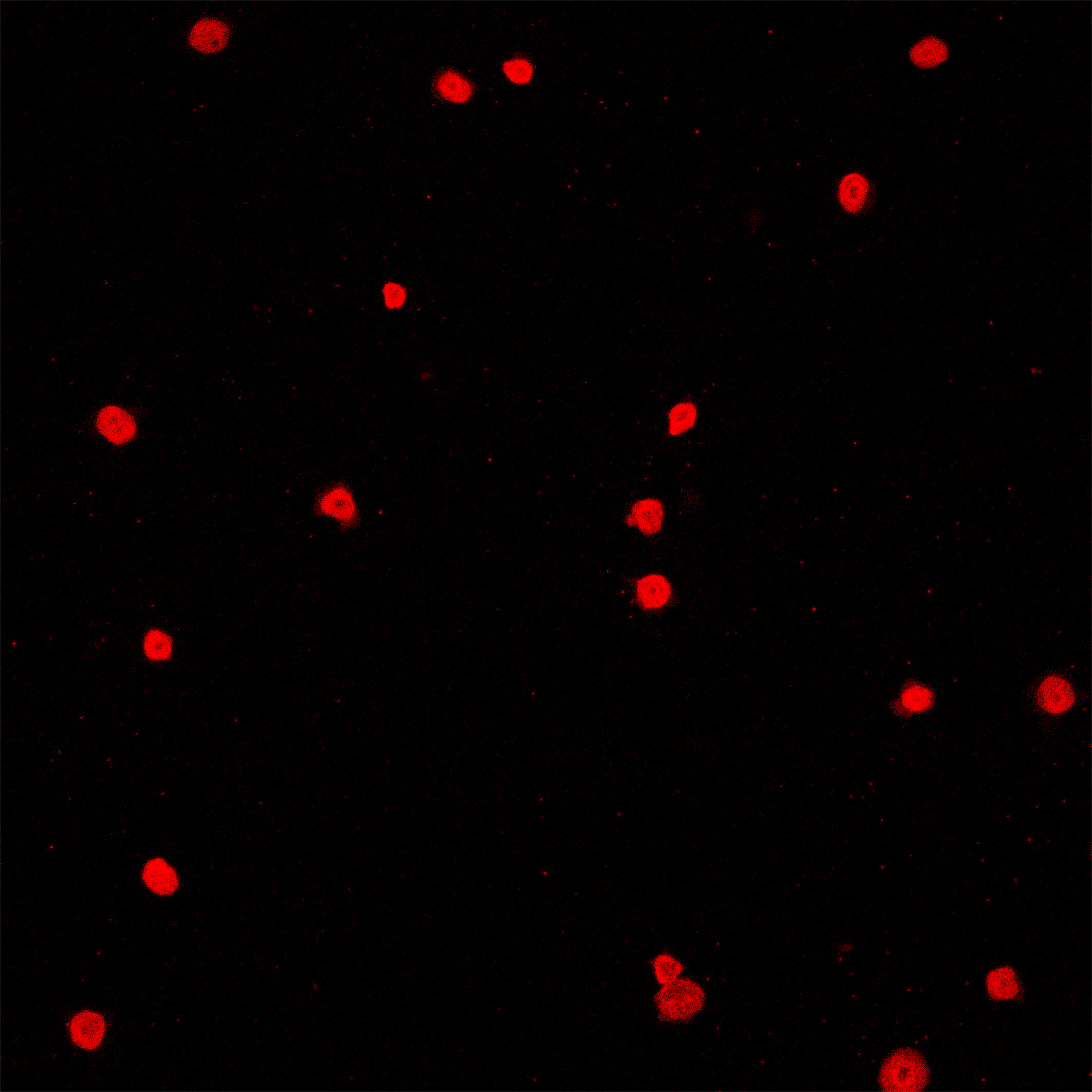

Supplement: Supplementary file 14 — EV and Appendix Figure Source Data [file 44318_2024_203_MOESM14_ESM.zip › Source Data for Expanded View and Appendix/Figure EV5/EV5A/Ctrl-KDM2A.jpg]

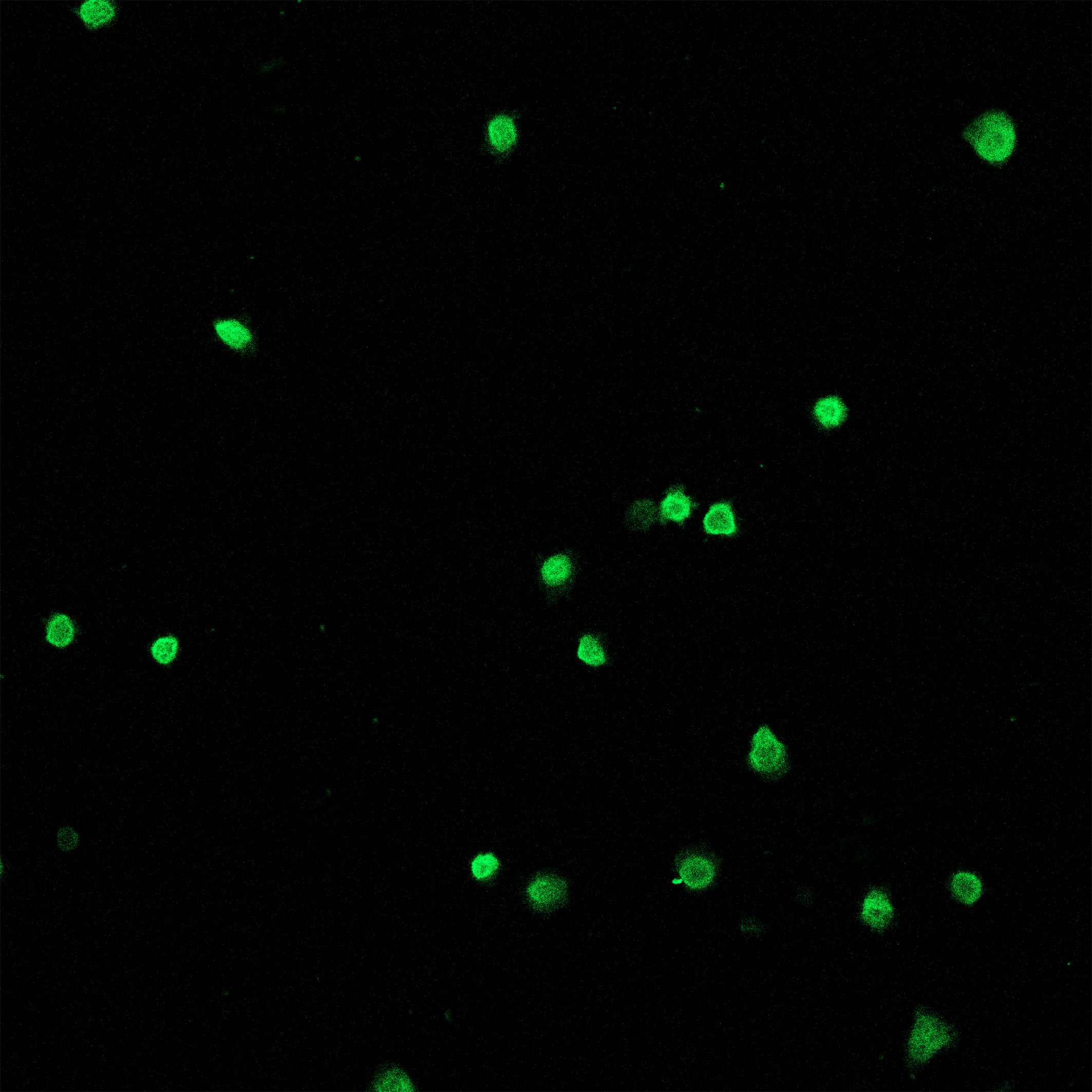

Supplement: Supplementary file 14 — EV and Appendix Figure Source Data [file 44318_2024_203_MOESM14_ESM.zip › Source Data for Expanded View and Appendix/Figure EV5/EV5A/cKO-cKIT.jpg]

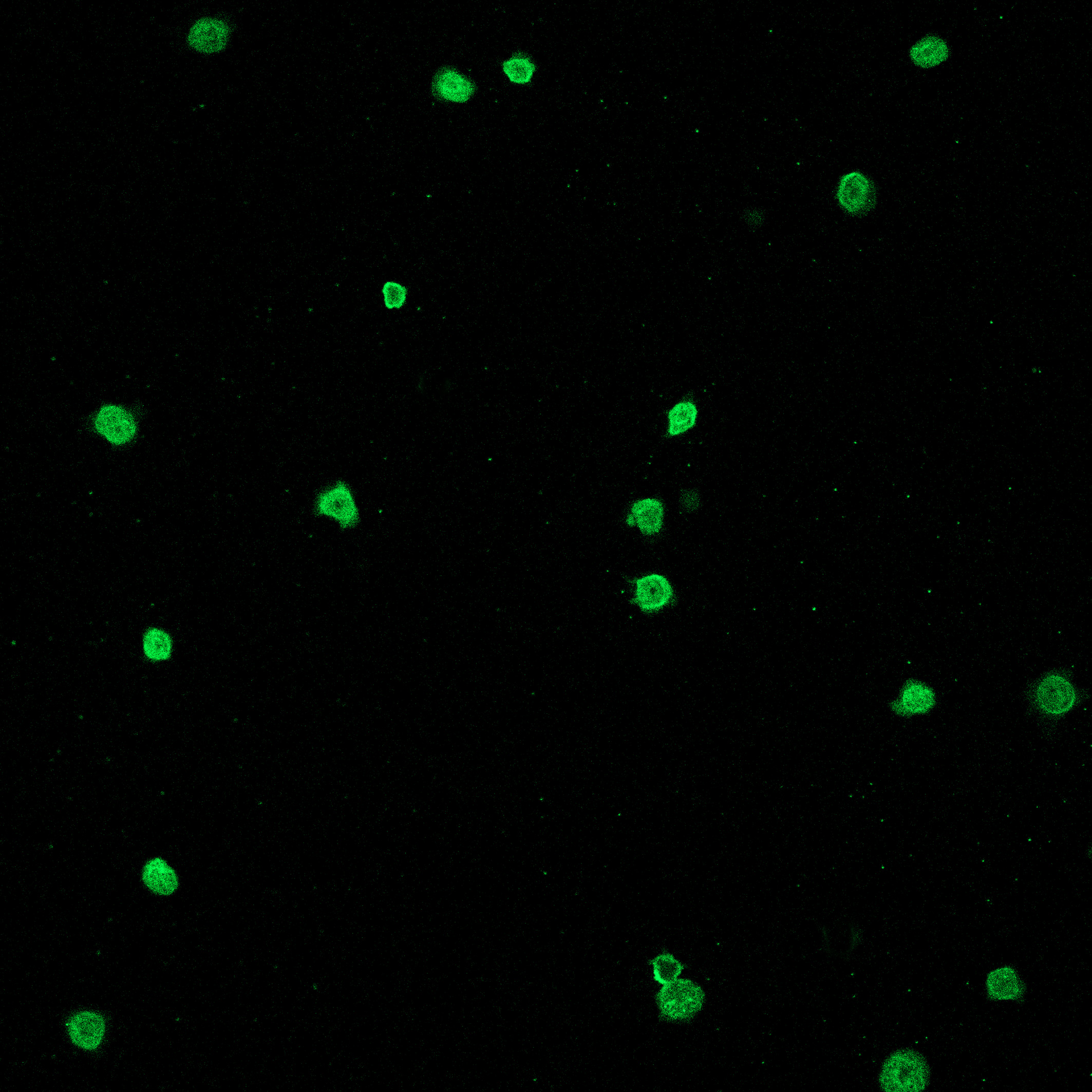

Supplement: Supplementary file 14 — EV and Appendix Figure Source Data [file 44318_2024_203_MOESM14_ESM.zip › Source Data for Expanded View and Appendix/Figure EV5/EV5A/Ctrl-cKIT.jpg]

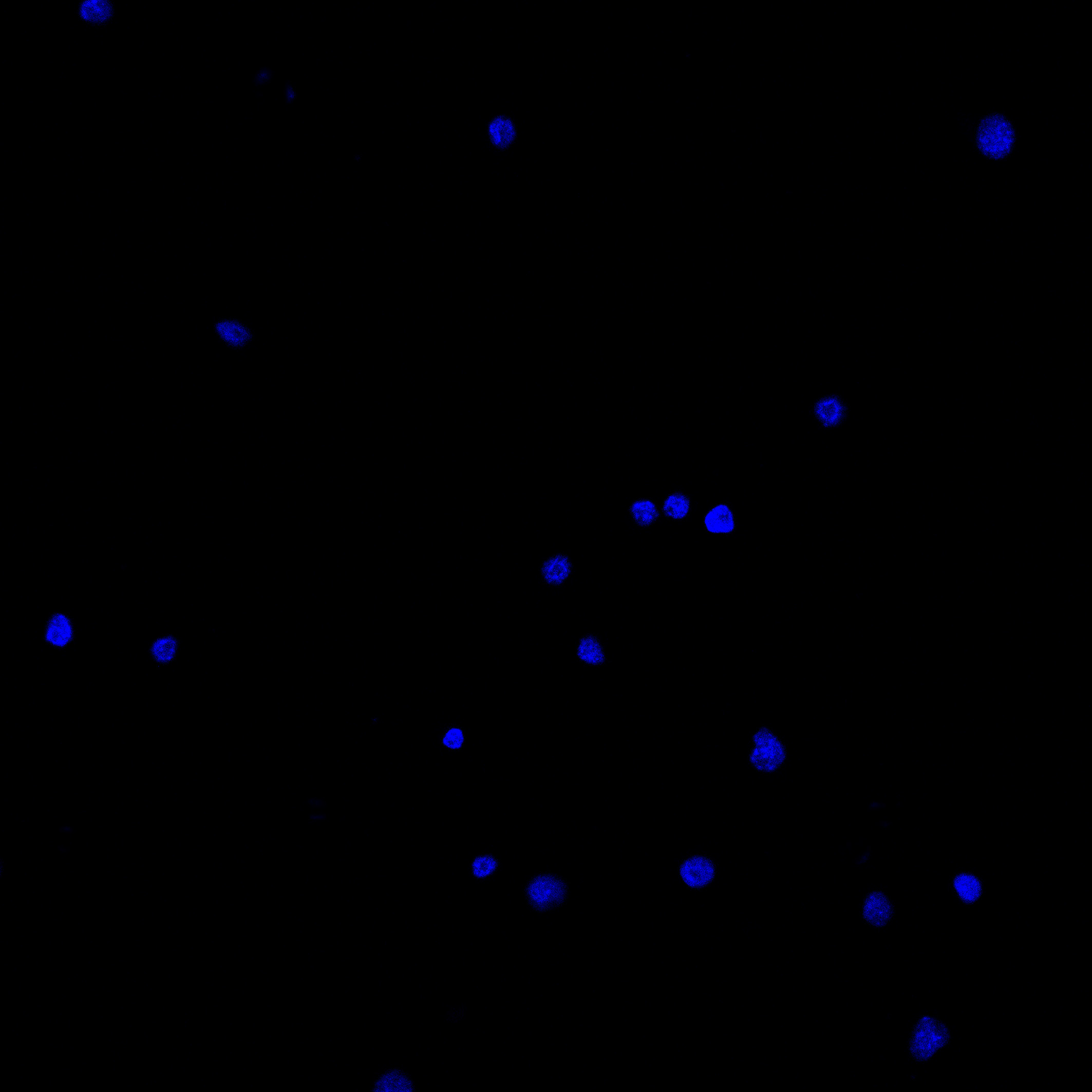

Supplement: Supplementary file 14 — EV and Appendix Figure Source Data [file 44318_2024_203_MOESM14_ESM.zip › Source Data for Expanded View and Appendix/Figure EV5/EV5A/cKO-DAPI.jpg]

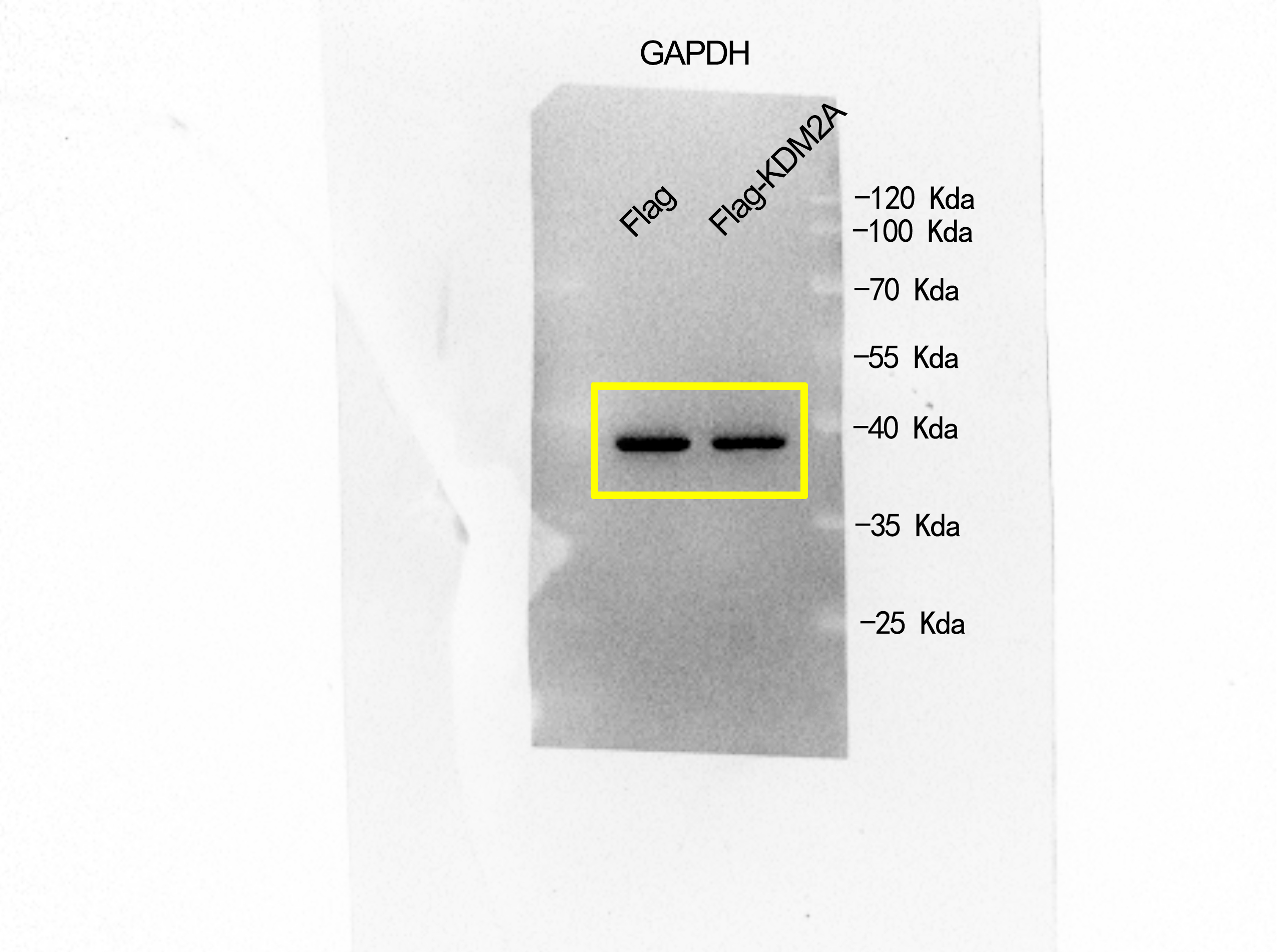

Supplement: Supplementary file 14 — EV and Appendix Figure Source Data [file 44318_2024_203_MOESM14_ESM.zip › Source Data for Expanded View and Appendix/Appendix Figure S4/S4D/WB-GAPDH.jpg]

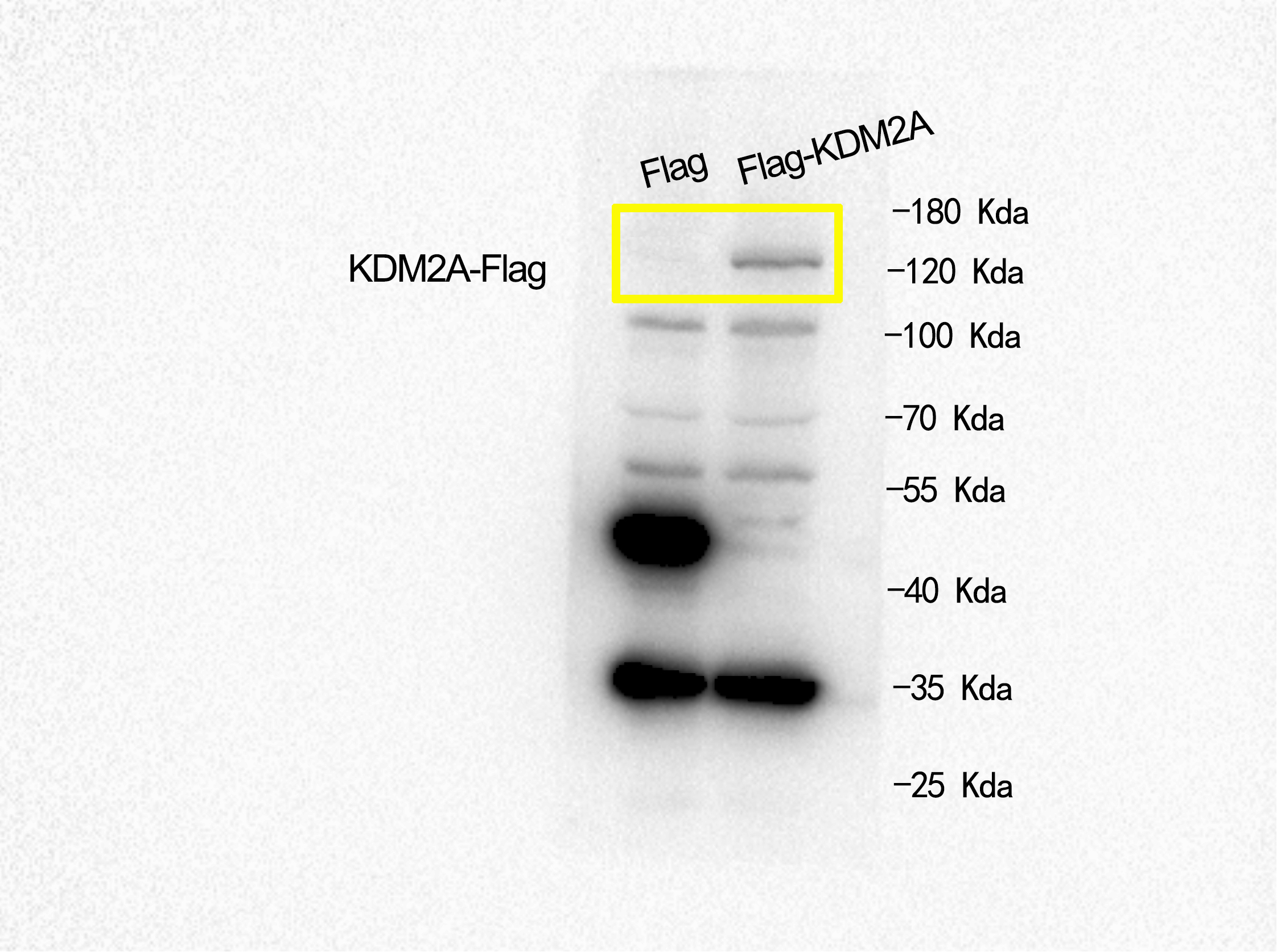

Supplement: Supplementary file 14 — EV and Appendix Figure Source Data [file 44318_2024_203_MOESM14_ESM.zip › Source Data for Expanded View and Appendix/Appendix Figure S4/S4D/WB-flag.jpg]

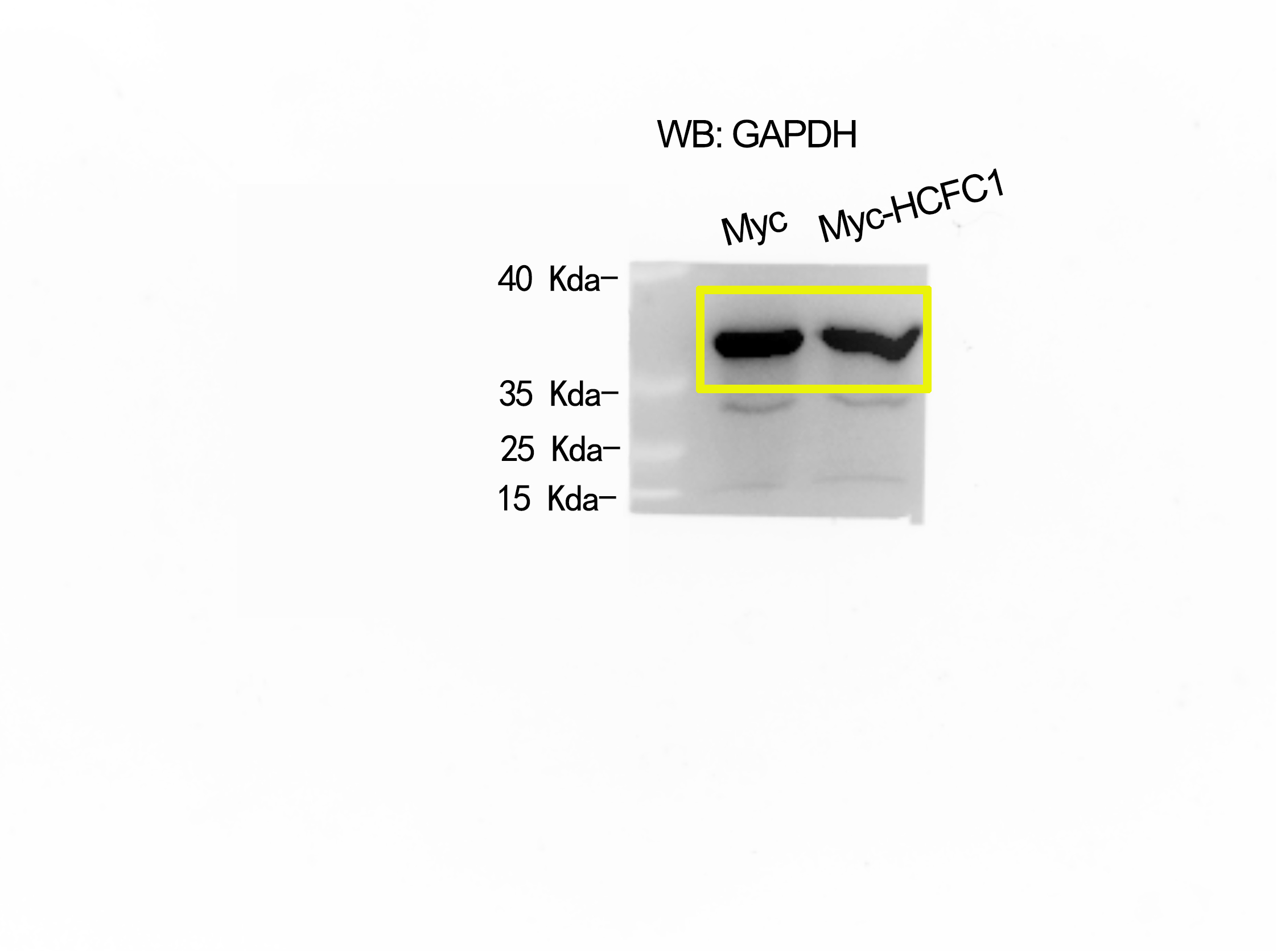

Supplement: Supplementary file 14 — EV and Appendix Figure Source Data [file 44318_2024_203_MOESM14_ESM.zip › Source Data for Expanded View and Appendix/Appendix Figure S4/S4E/WB-GAPDH.jpg]

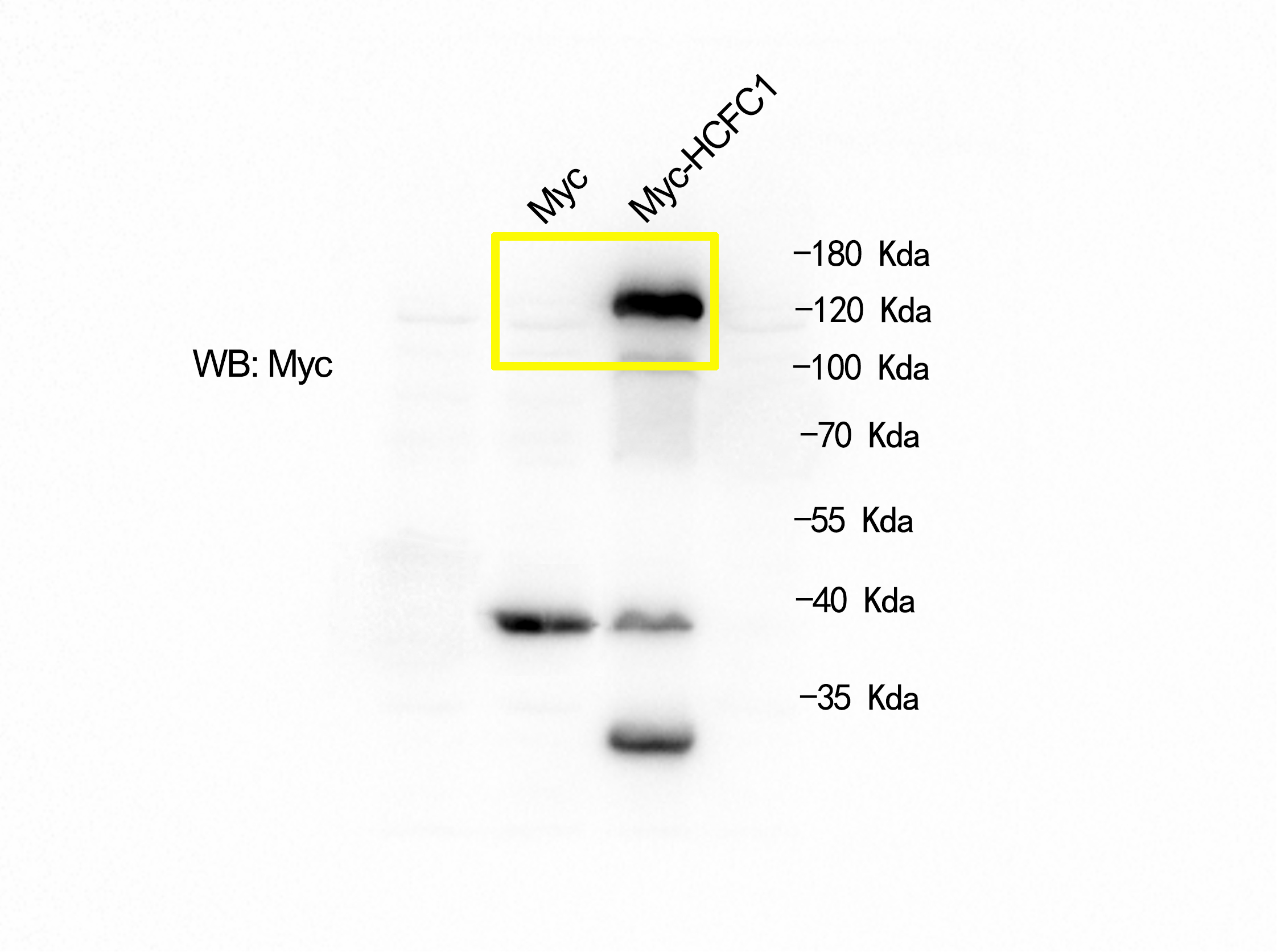

Supplement: Supplementary file 14 — EV and Appendix Figure Source Data [file 44318_2024_203_MOESM14_ESM.zip › Source Data for Expanded View and Appendix/Appendix Figure S4/S4E/WB-Myc.jpg]

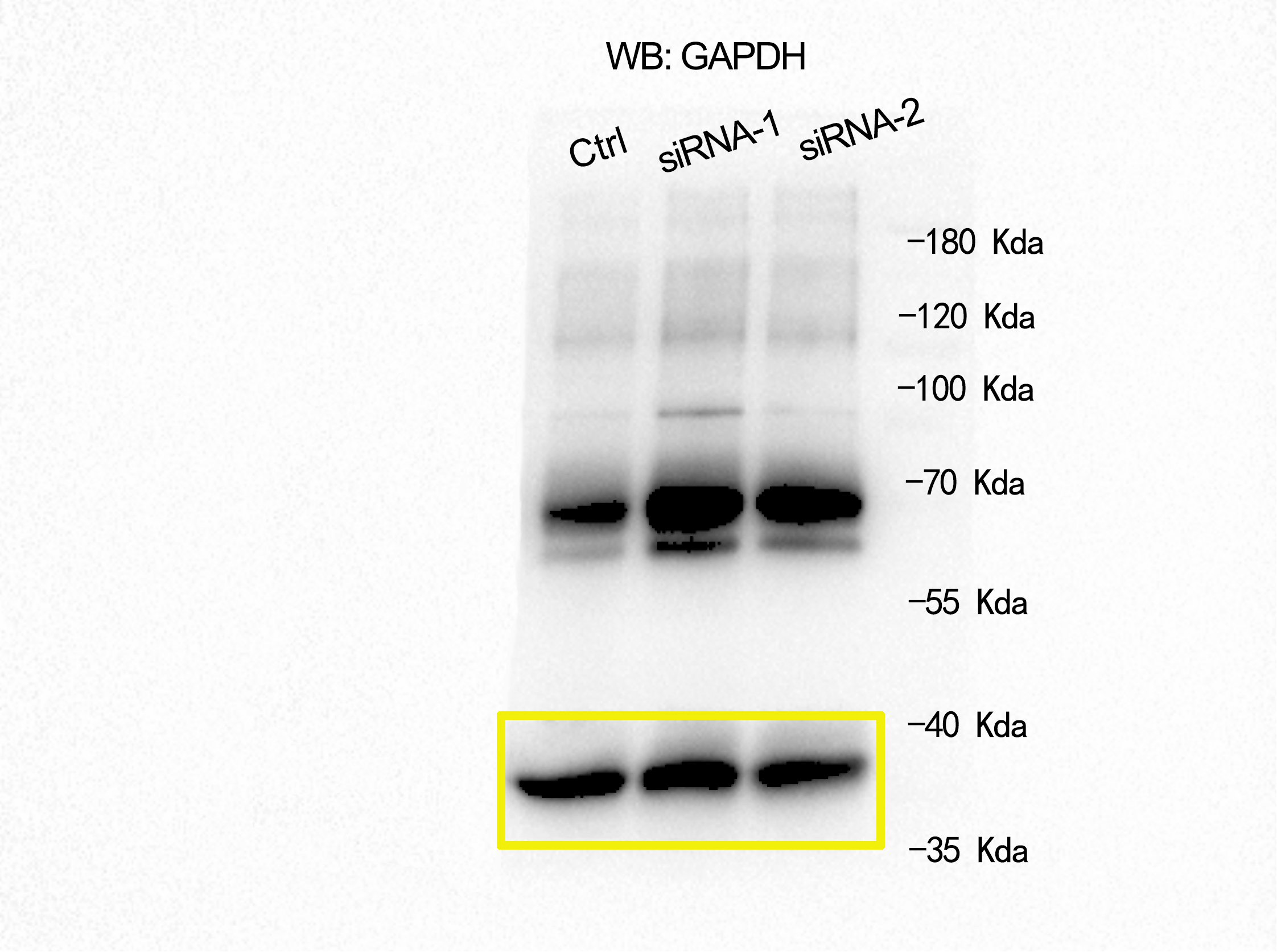

Supplement: Supplementary file 14 — EV and Appendix Figure Source Data [file 44318_2024_203_MOESM14_ESM.zip › Source Data for Expanded View and Appendix/Appendix Figure S4/S4G/WB-GAPDH.jpg]

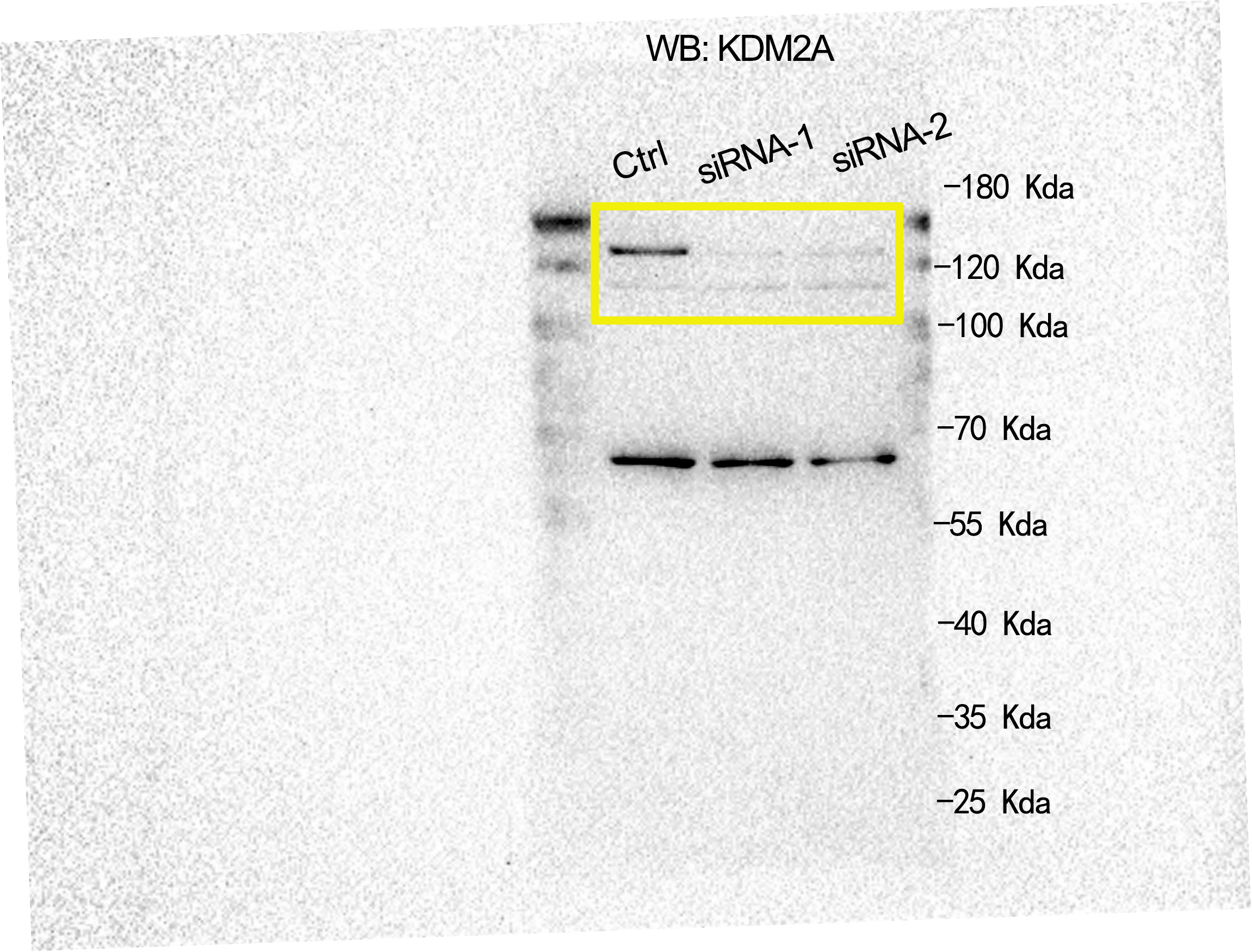

Supplement: Supplementary file 14 — EV and Appendix Figure Source Data [file 44318_2024_203_MOESM14_ESM.zip › Source Data for Expanded View and Appendix/Appendix Figure S4/S4G/WB-KDM2A.jpg]
