# Supplementary material for: Histone demethylase KDM2A recruits HCFC1 and E2F1 to orchestrate male germ cell meiotic entry and progression
Source: EMBO J. 2024 Aug 19;43(19):4197–227. doi: 10.1038/s44318-024-00203-4 (PMC11448500; doi:10.1038/s44318-024-00203-4)
Supplement: Supplementary file 15 — Expanded View Figures [file 44318_2024_203_MOESM15_ESM.pdf]

Expanded View Figures

Figure EV1. The expression pattern of KDM2A and H3K36me1/2/3 during spermatogenesis.

(**A**) RT-qPCR analyses of *Kdm2a* mRNA levels in various organs from wild-type (WT) adult mice. $n = 3$ (three biological replicates). Data are presented as the mean ± SD. (**B**) WB analyses the expression of KDM2A protein levels in multiple organs from WT adult mice. GAPDH was used as a loading control. Biologically independent mice ($n = 3$) performed three independent experiments. Data are presented as the mean ± SD. (**C**) RT-qPCR analyses of *Kdm2a* mRNA levels in WT testes at different developmental stages including postnatal day 0 (P0), P7, P14, P21, P28, P35, and adult. $n = 3$ (three biological replicates). (**D**) WB analyses the KDM2A protein levels in developing WT testes. GAPDH served as a loading control. Biologically independent mice ($n = 3$) performed three separate experiments. (**E**) Whole-mount staining of seminiferous tubules from adult WT testis with antibodies against KDM2A and PLZF. Nuclei were stained with DAPI. Scale bars = 50 μm. Examples of A_{single} (As), A_{paired} (Apr), and $A_{aligned}$ (Aal(4), Aal(8)) undifferentiated spermatogonia in adult testes (dotted circles). (**F**) same as (**E**) for adult WT testes, but with antibodies against KDM2A and c-KIT. Nuclei were stained with DAPI. Scale bars = 50 μm. (**G**) Co-Immunofluorescent staining of KDM2A and PLZF on testis sections from WT mice at P14. Nuclei were stained with DAPI. Scale bars = 50 μm. (**H**) Co-Immunofluorescent staining of SALL4 with H3K36me1 (upper), H3K36me2 (middle), and H3K36me3 (lower) on testis sections from WT mice at P14, respectively. Nuclei were stained with DAPI. Scale bars = 50 μm. The white arrows indicate the $SALL4^{+}$ spermatogonia. (**I**) Co-Immunofluorescent staining of SYCP3 with H3K36me1 (upper), H3K36me2 (middle), and H3K36me3 (lower) on surface-spread spermatocytes from WT mice at P21, respectively. Nuclei were stained with DAPI. Leptotene (Lep), Zygotene (Zyg), Pachytene (Pac), and Diplotene (Dip) spermatocytes are shown. Scale bars = 5 μm. (**J**–**L**) Co-Immunofluorescent staining of PLZF with H3K36me1 (**J**), H3K36me2 (**K**), and H3K36me3 (**L**) on whole-mount testes seminiferous tubules from adult WT mice. Nuclei were stained with DAPI. Scale bars = 50 μm. Source data are available online for this figure.

 © The Author(s)

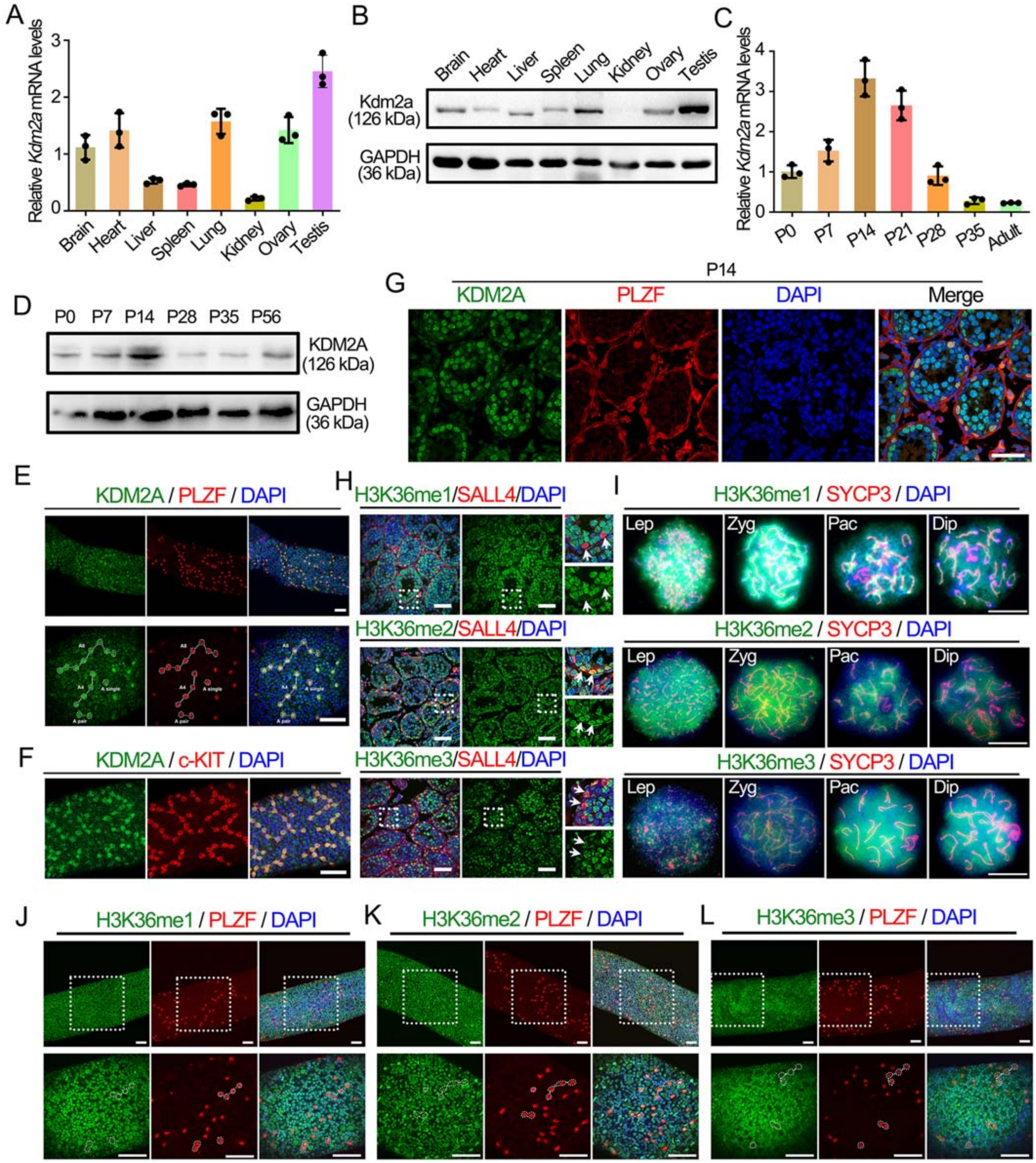
A
Relative Kdm2a mRNA levels
Brain Heart Liver Spleen Lung Kidney Ovary Testis
B
Kdm2a (126 kDa)
GAPDH (36 kDa)
C
P0 P7 P14 P21 P28 P35 Adult
D
P0 P7 P14 P28 P35 P56
KDM2A (126 kDa)
GAPDH (36 kDa)
G
P14
KDM2A PLZF DAPI Merge
E
KDM2A / PLZF / DAPI
F
KDM2A / c-KIT / DAPI
H
H3K36me1/SALL4/DAPI
H3K36me2/SALL4/DAPI
H3K36me3/SALL4/DAPI
I
H3K36me1 / SYCP3 / DAPI
H3K36me2 / SYCP3 / DAPI
H3K36me3 / SYCP3 / DAPI
Lep Zyg Pac Dip
J
H3K36me1 / PLZF / DAPI
K
H3K36me2 / PLZF / DAPI
L
H3K36me3 / PLZF / DAPI

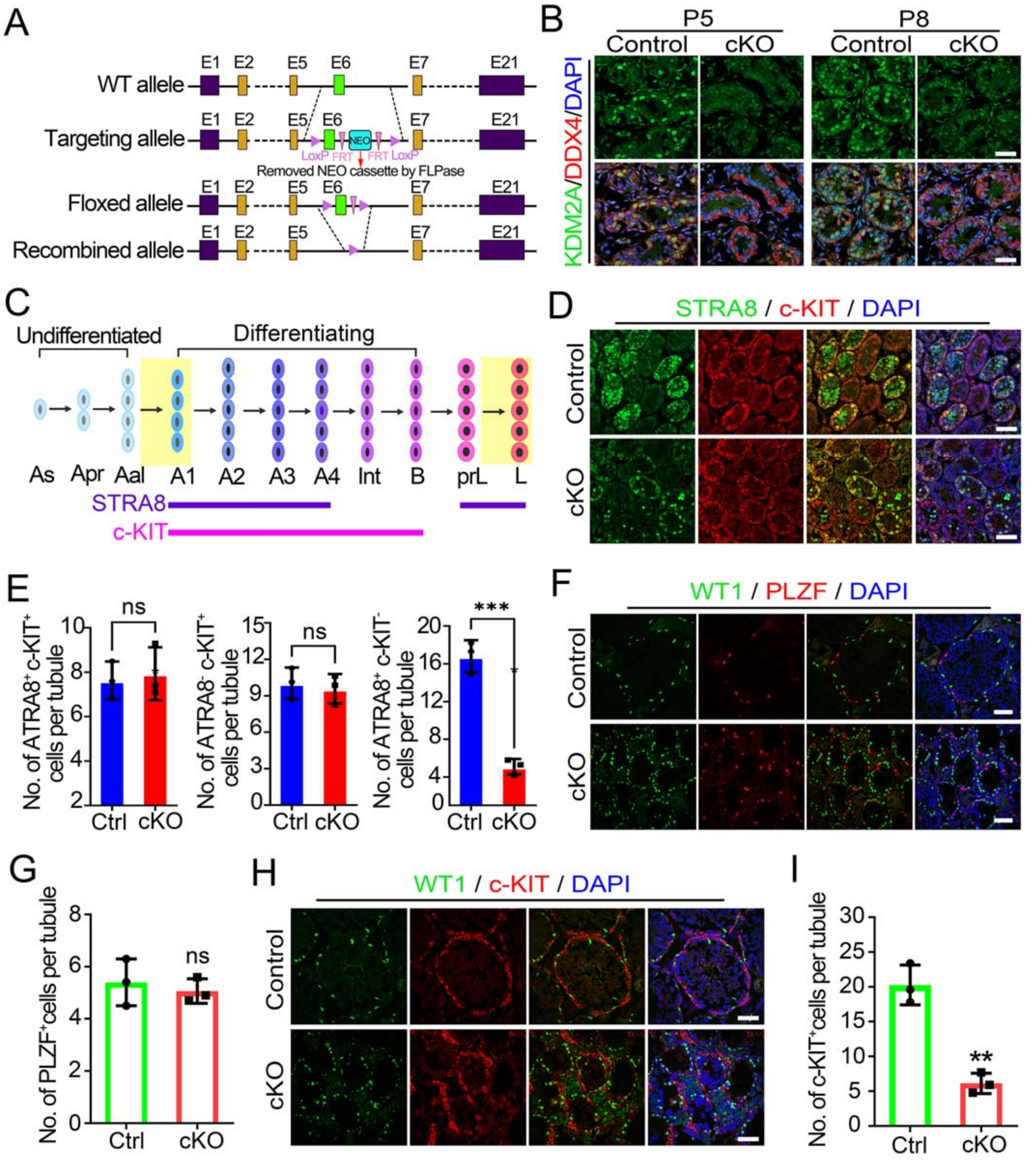
A
E1 E2 E5 E6 E7 E21
WT allele
Targeting allele
NEO
LoxP FRT FRT LoxP
Removed NEO cassette by FLPase
Floxed allele
Recombined allele
B
P5 P8
Control cKO Control cKO
KDM2A/DDX4/DAPI
C
Undifferentiated
Differentiating
As Apr Aal A1 A2 A3 A4 Int B prL L
STRA8
c-KIT
D
STRA8 / c-KIT / DAPI
Control
cKO
E
No. of ATRA8⁺ c-KIT⁺ cells per tubule
No. of ATRA8⁻ c-KIT⁺ cells per tubule
No. of ATRA8⁺ c-KIT⁻ cells per tubule
ns
ns
***
Ctrl cKO
F
WT1 / PLZF / DAPI
Control
cKO
G
No. of PLZF⁺cells per tubule
ns
Ctrl cKO
H
WT1 / c-KIT / DAPI
Control
cKO
I
No. of c-KIT⁺cells per tubule
**
Ctrl cKO

 © The Author(s)

Figure EV2. *Kdm2a* deletion results in defective differentiation of adult spermatogonia.

(**A**) Schematic diagram of the targeting strategy used to generate floxed *Kdm2a* allele by homologous recombination in mouse embryonic stem cells.
(**B**) Immunofluorescence (IF) staining of KDM2A and DDX4 in control and *Kdm2a* cKO testes of P5 and P8 mice. Scale bars = 50 μm. (**C**) The Schematic diagram of spermatogonial differentiation process. As, A-single; Apr, A-paired; Aal, A-aligned; Int, intermediary spermatogonia; B, B spermatogonia; prL, preleptotene spermatocytes; and L, leptotene spermatocytes. (**D**) Co-immunofluorescent staining of STRA8 with c-KIT on testis sections from P10 control and *Kdm2a* cKO mice. Nuclei were stained with DAPI. Scale bars = 50 μm. (**E**) The quantifications of $STRA8^{+}$ $c\text{-}KIT^{+}$ or $STRA8^{-}$ $c\text{-}KIT^{+}$ or $STRA8^{+}$ $c\text{-}KIT^{-}$ cells per tubule. Data were presented as the mean ± SD. $n = 3$ biological replicates. *P* value was calculated using a two-tailed Student's *t*-test. ns, not significant. ***$P = 0.0004$. (**F**) Co-immunofluorescent staining of WT1 with PLZF on testis sections from adult control and *Kdm2a* cKO mice. Nuclei were stained with DAPI. Scale bars = 50 μm. (**G**) Quantification of $PLZF^{+}$ cells per tubule for (**F**). Data were presented as the mean ± SD. $n = 3$ biological replicates. *P* value was calculated using a two-tailed Student's *t*-test. ns, not significant. (**H**) Co-immunofluorescent staining of WT1 with c-KIT on testis sections from adult control and *Kdm2a* cKO mice. Nuclei were stained with DAPI. Scale bars = 50 μm. (**I**) Quantification of $c\text{-}Kit^{+}$ cells per tubule for (**H**). Data were presented as the mean ± SD. $n = 3$ biological replicates. *P* value was calculated using a two-tailed Student's *t*-test. ns, not significant. **$P = 0.0016$. Source data are available online for this figure.

© The Author(s)

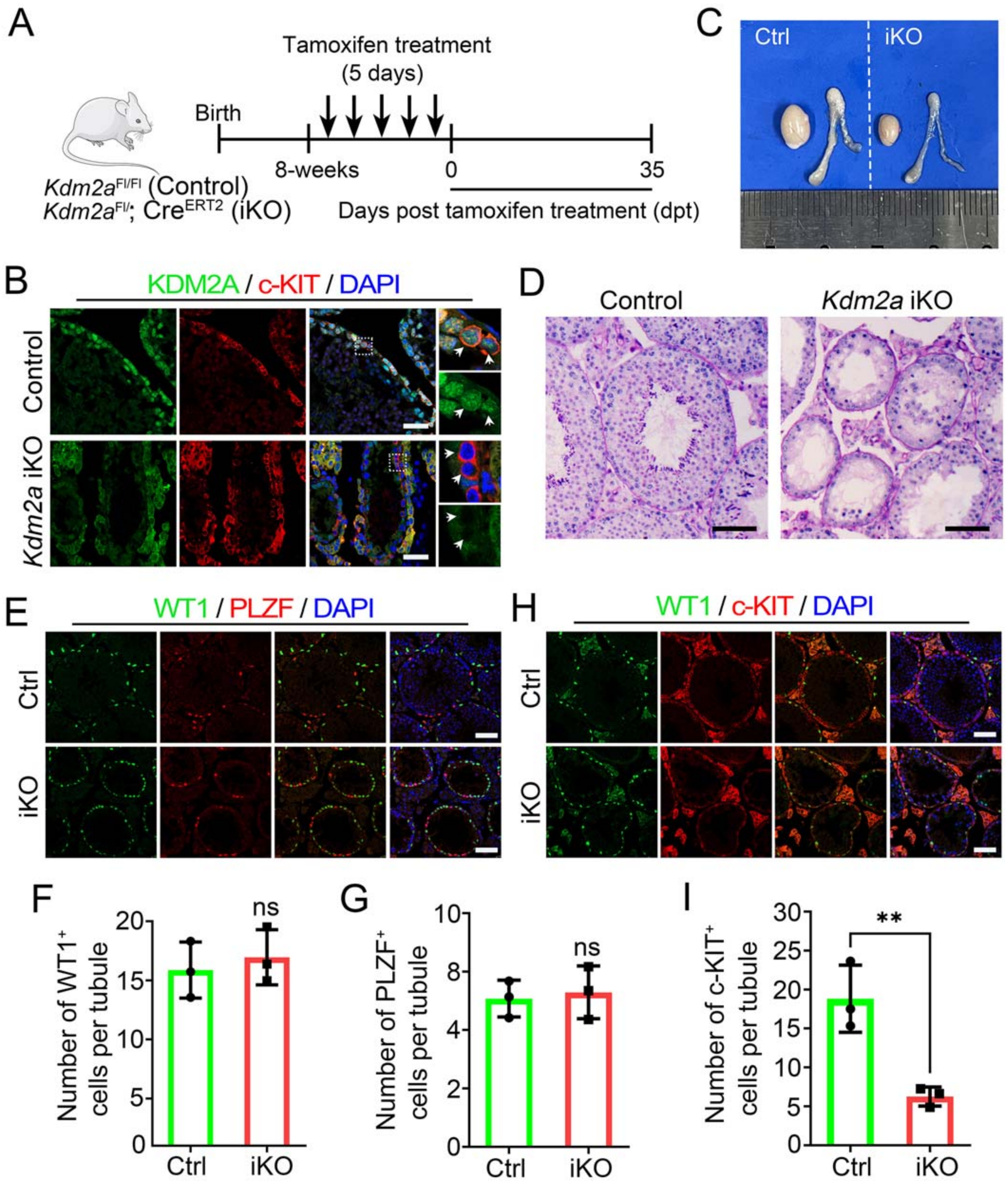
A
Tamoxifen treatment
(5 days)
Birth
8-weeks
0
35
Kdm2a^Fl/Fl (Control)
Kdm2a^Fl/+; Cre^ERT2 (iKO)
Days post tamoxifen treatment (dpt)
C
Ctrl
iKO
B
KDM2A / c-KIT / DAPI
Control
Kdm2a iKO
D
Control
Kdm2a iKO
E
WT1 / PLZF / DAPI
Ctrl
iKO
H
WT1 / c-KIT / DAPI
Ctrl
iKO
F
Number of WT1+ cells per tubule
ns
Ctrl
iKO
G
Number of PLZF+ cells per tubule
ns
Ctrl
iKO
I
Number of c-KIT+ cells per tubule
**
Ctrl
iKO

 © The Author(s)

Figure EV3. Tamoxifen-induced *Kdm2a* deletion in adult mice affects spermatogonial differentiation.

(**A**) Regimen of tamoxifen treatment in adult (2 M) *Kdm2a*$^{fl/-}$ Ddx4-Cre^{ERT2} male mice. (**B**) Immunofluorescence (IF) staining of KDM2A and c-KIT in control and *Kdm2a* iKO testes. Arrowheads indicate KDM2A signals in the c-KIT positive cells. Nuclei were stained with DAPI. Enlarged images are shown in right panels. Scale bars = 50 µm. (**C**) Gross morphology of testes and epididymides from control and *Kdm2a* iKO mice. (**D**) Periodic acid-Schiff (PAS) staining of testes from control and *Kdm2a*-iKO mice. Scale bars = 50 µm. (**E**) Co-immunofluorescent staining of WT1 with PLZF on testis sections from adult control and *Kdm2a* iKO mice. Nuclei were stained with DAPI. Scale bars = 50 µm. (**F**, **G**) Quantification of WT1$^+$ and PLZF$^+$ (**G**) cells per tubule for (**E**). Data were presented as the mean ± SD. $n = 3$ biological replicates. *P* value was calculated using a two-tailed Student's *t*-test. ns, not significant. (**H**) Co-immunofluorescent staining of WT1 with c-KIT on testis sections from adult control and *Kdm2a* iKO mice. Nuclei were stained with DAPI. Scale bars = 50 µm. (**I**) Quantification of c-Kit$^+$ cells per tubule for (**H**). Data were presented as the mean ± SD. $n = 3$ biological replicates. *P* value was calculated using a two-tailed Student's *t*-test. ns, not significant. $^{**}P = 0.0083$. Source data are available online for this figure.

© The Author(s)

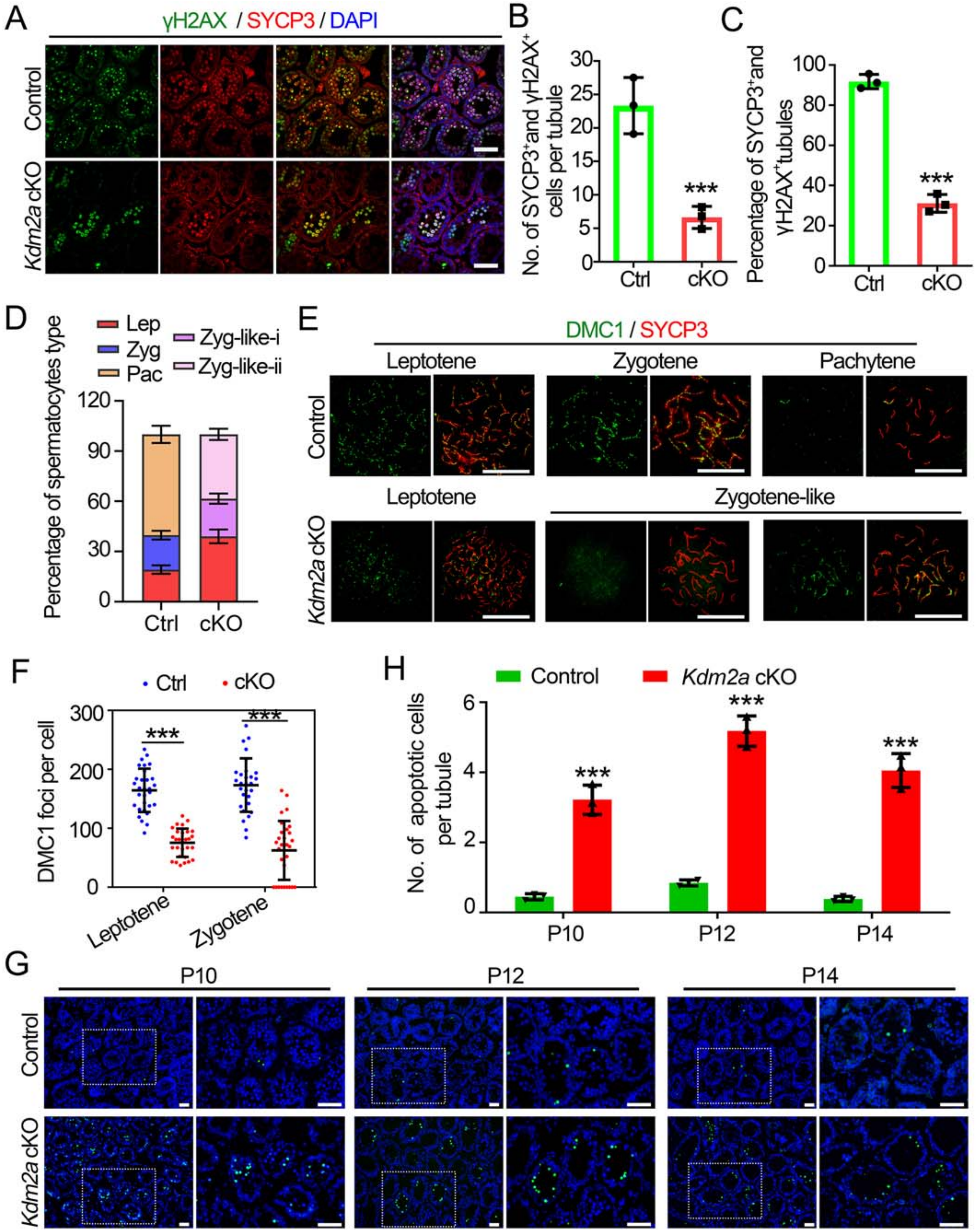
A
γH2AX / SYCP3 / DAPI
Control
Kdm2a cKO
B
No. of SYCP3+and γH2AX+ cells per tubule
30
25
20
15
10
5
0
Ctrl
cKO
***
C
Percentage of SYCP3+and γH2AX+tubules
100
80
60
40
20
0
Ctrl
cKO
***
D
Lep
Zyg
Pac
Zyg-like-i
Zyg-like-ii
Percentage of spermatocytes type
120
90
60
30
0
Ctrl
cKO
E
DMC1 / SYCP3
Leptotene
Zygotene
Pachytene
Control
Leptotene
Zygotene-like
Kdm2a cKO
F
Ctrl
cKO
DMC1 foci per cell
300
200
100
0
***
***
Leptotene
Zygotene
H
Control
Kdm2a cKO
No. of apoptotic cells per tubule
6
4
2
0
***
***
***
P10
P12
P14
G
P10
P12
P14
Control
Kdm2a cKO

Figure EV4. *Kdm2a* deletion results in defective meiosis initiation and progression.

(**A**) Co-immunostaining of SYCP3 with γH2AX in testis sections from control and *Kdm2a* cKO mice at P14. Nuclei were stained with DAPI. Scale bars = 50 μm. (**B**, **C**) The quantifications of $SYCP3^+$ and $γH2AX^+$ cells per tubule (**B**) and the percentage of $SYCP3^+$ and $γH2AX^+$ tubule (**C**) are shown. Data were presented as the mean ± SD. $n = 3$ biological replicates. *P* value was calculated using a two-tailed Student's *t*-test. ns, not significant. For (**B**), $^{***}P = 0.003$; For (**C**), $^{***}P < 0.0001$. (**D**) The percentage of spermatocytes at the leptotene (Lep), zygotene (Zyg), and pachytene (Pac) stages for (Fig. 3D). Data were presented as the mean ± SD. $n = 3$ biological replicates. (**E**) Spermatocyte spreads from control and *Kdm2a* cKO testes at P18 were co-stained SYCP3 and DMC1. Scale bars = 50 μm. (**F**) A scatter plot shows the number of DMC1 foci per cell on SYCP3 axes in leptotene and zygotene spermatocytes from control and *Kdm2a* cKO mice, respectively. Data are presented as the mean ± SD. A total of $n = 30$ control leptotema, $n = 29$ *Kdm2a* cKO leptotema, $n = 29$ control zygonema, and $n = 30$ *Kdm2a* cKO zygonema were counted from three biologically independent mice for each genotype. *P* value was calculated using a two-tailed Mann–Whitney *U*-test. left to right: $^{***}P < 0.0001$, $^{***}P < 0.0001$. (**G**) TUNEL staining of testis sections from control and *Kdm2a* cKO mice at P10, P12, and P14. Nuclei were stained with DAPI. Scale bars = 50 μm. (**H**) Quantification of apoptotic cells per tubule for (**G**). Data were presented as the mean ± SD. $n = 3$ biological replicates. *P* value was calculated using a two-tailed Student's *t*-test. left to right: $^{***}P = 0.0003$, $^{***}P < 0.0001$, $^{***}P = 0.0002$. Source data are available online for this figure.

© The Author(s)

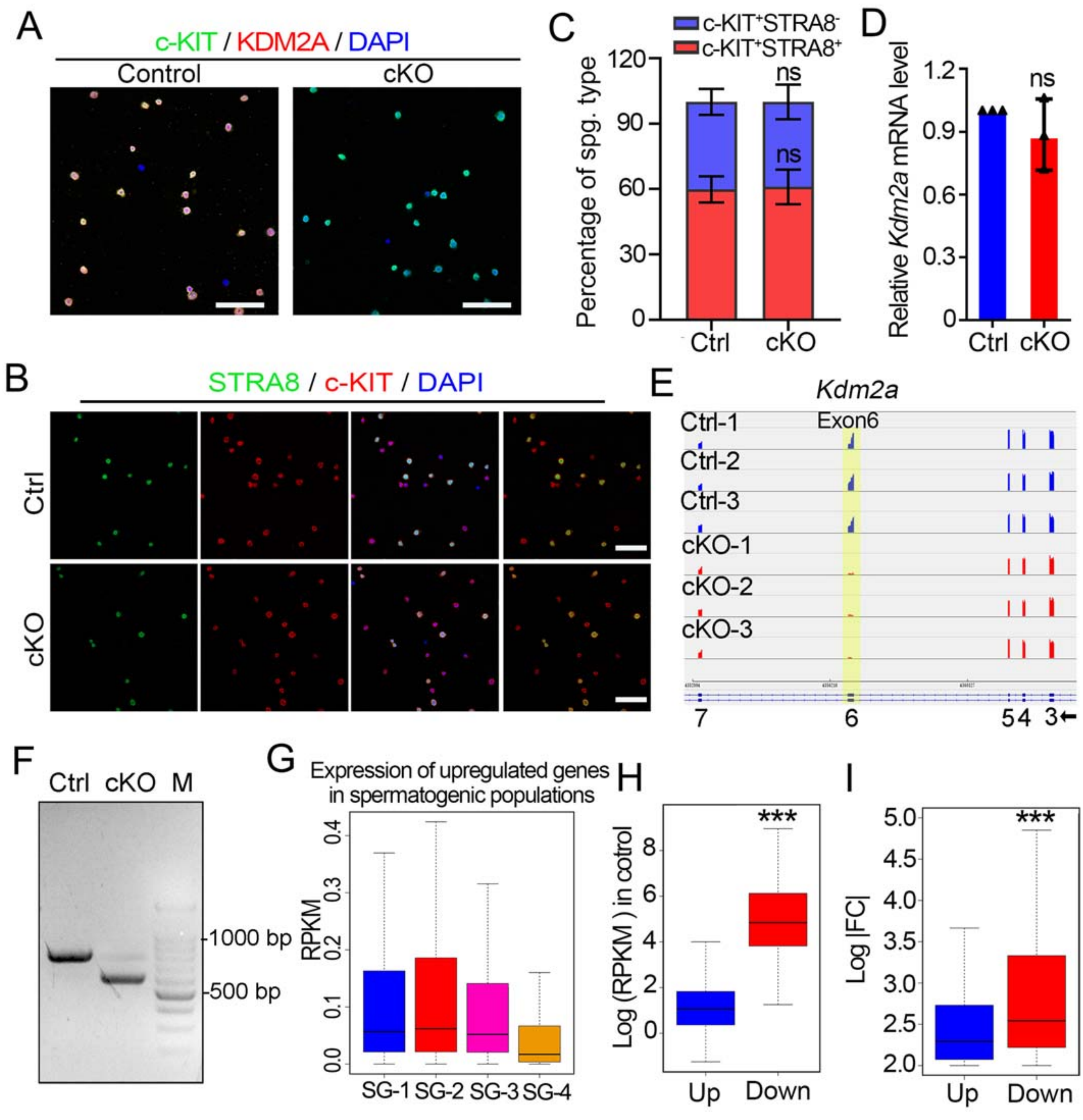
A
c-KIT / KDM2A / DAPI
Control
cKO
C
c-KIT+STRA8-
c-KIT+STRA8+
Percentage of spg. type
120
90
60
30
0
ns
ns
Ctrl
cKO
D
Relative Kdm2a mRNA level
1.2
0.9
0.6
0.3
0
ns
Ctrl cKO
B
STRA8 / c-KIT / DAPI
Ctrl
cKO
E
Kdm2a
Exon6
Ctrl-1
Ctrl-2
Ctrl-3
cKO-1
cKO-2
cKO-3
7
6
54 3←
F
Ctrl cKO M
-1000 bp
-500 bp
G
Expression of upregulated genes in spermatogenic populations
RPKM
0.0
0.1
0.2
0.3
0.4
SG-1 SG-2 SG-3 SG-4
H
Log (RPKM) in cotrol
***
8
6
4
2
0
Up Down
I
Log |FC|
***
5.0
4.5
4.0
3.5
3.0
2.5
2.0
Up Down

 © The Author(s)

Figure EV5. Verification of KDM2A deletion in isolated c-KIT cells from *Kdm2a* cKO testes.

(**A**) Co-immunofluorescence staining of anti-c-KIT with anti-KDM2A on isolated differentiated spermatogonal from control and KDM2A cKO mice. Nuclei were stained with DAPI. Scale bars = 50 µm. (**B**) Co-immunofluorescence staining of anti-STRA8 with anti-c-KIT on isolated differentiated spermatogonal from P10 control and *Kdm2a* cKO mice. Nuclei were stained with DAPI. Scale bars = 50 µm. (**C**) The percentage of c-KIT$^-$ STRA8$^+$ and c-KIT$^+$STRA8$^+$ spermatogonia from control and *Kdm2a* cKO mice. Data were presented as the mean ± SD. $n = 3$ biological replicates. *P* value was calculated using a two-tailed Student's *t*-test. ns, not significant. (**D**) RT-qPCR analyses of *Kdm2a* mRNA levels in isolated differentiated spermatogonal from P10 control and *Kdm2a* cKO mice. Data were presented as the mean ± SD. $n = 3$ biological replicates. *P* value was calculated using a two-tailed Student's *t*-test. ns, not significant. (**E**) Genome browser tracks depicting peaks of the region containing exon6 of Kdm2a based on RNA-seq. (**F**) RT-PCR analyses of *Kdm2a* mRNA in isolated c-KIT spermatogonia from control and *Kdm2a* cKO mice. (**G**) Expression levels (RPKM) of the upregulated 1934 genes in different types of spermatogonia (SG1-4) were reanalyzed with the previously published data of stage-specific bulk RNA-seq ($N = 3$ biologically independent samples). Whiskers indicate min and max. Bounds of box indicate 25th and 75th percentiles quantile with median. SPG 1, 2, 3, 4 correspond to SSCs (spermatogenic stem cells), undifferentiated spermatogonia, early differentiating spermatogonia and late differentiating spermatogonia, respectively. (**H**) Expression levels (RPKM) of the 1934 upregulated and 1922 downregulated genes in control (c-KIT+ cells of P10 control mice) are shown by box-whisker plot (whiskers indicate min and max. Bounds of box indicate 25th and 75th percentiles quantile with median). Data were presented as the mean ± SD. *P* value was calculated using an unpaired two-tailed Student's *t*-test. ***$P < 0.0001$. (**I**) Fold change of the 1934 upregulated and 1922 downregulated genes from RNA-seq are shown by box-whisker plot (whiskers indicate min and max. Bounds of box indicate 25th and 75th percentiles quantile with median). Data were presented as the mean ± SD. *P* value was calculated using an unpaired two-tailed Student's *t*-test. ***$P < 0.0001$. Source data are available online for this figure.

© The Author(s)
